# Pattern Recognition in Pulmonary Tuberculosis Defined by High Content Peptide Microarray Chip Analysis Representing 61 Proteins from *M. tuberculosis*

**DOI:** 10.1371/journal.pone.0003840

**Published:** 2008-12-09

**Authors:** Simani Gaseitsiwe, Davide Valentini, Shahnaz Mahdavifar, Isabelle Magalhaes, Daniel F. Hoft, Johannes Zerweck, Mike Schutkowski, Jan Andersson, Marie Reilly, Markus J. Maeurer

**Affiliations:** 1 Department of Microbiology, Tumor and Cell Biology Center (MTC), Karolinska Institutet Stockholm, Stockholm, Sweden; 2 Department of Medical Epidemiology and Biostatistics, Karolinska Institutet, Stockholm, Sweden; 3 The Swedish Institute for Infectious Disease Control (SMI), Stockholm, Sweden; 4 Division of Immunobiology, Departments of Internal Medicine & Molecular Microbiology, Saint Louis University Medical Center, Saint Louis, Missouri, United States of America; 5 JPT, Berlin, Germany; 6 Department of Medicine, Karolinska Institutet, Karolinska University Hospital Huddinge, Stockholm, Sweden; New York University School of Medicine, United States of America

## Abstract

**Background:**

Serum antibody-based target identification has been used to identify tumor-associated antigens (TAAs) for development of anti-cancer vaccines. A similar approach can be helpful to identify biologically relevant and clinically meaningful targets in *M.tuberculosis* (MTB) infection for diagnosis or TB vaccine development in clinically well defined populations.

**Method:**

We constructed a high-content peptide microarray with 61 M.tuberculosis proteins as linear 15 aa peptide stretches with 12 aa overlaps resulting in 7446 individual peptide epitopes. Antibody profiling was carried with serum from 34 individuals with active pulmonary TB and 35 healthy individuals in order to obtain an unbiased view of the MTB epitope pattern recognition pattern. Quality data extraction was performed, data sets were analyzed for significant differences and patterns predictive of TB+/−.

**Findings:**

Three distinct patterns of IgG reactivity were identified: 89/7446 peptides were differentially recognized (in 34/34 TB+ patients and in 35/35 healthy individuals) and are highly predictive of the division into TB+ and TB−, other targets were exclusively recognized in all patients with TB (e.g. sigmaF) but not in any of the healthy individuals, and a third peptide set was recognized exclusively in healthy individuals (35/35) but no in TB+ patients. The segregation between TB+ and TB− does not cluster into specific recognition of distinct MTB proteins, but into specific peptide epitope ‘hotspots’ at different locations within the same protein. Antigen recognition pattern profiles in serum from TB+ patients from Armenia vs. patients recruited in Sweden showed that IgG-defined MTB epitopes are very similar in individuals with different genetic background.

**Conclusions:**

A uniform target MTB IgG-epitope recognition pattern exists in pulmonary tuberculosis. Unbiased, high-content peptide microarray chip-based testing of clinically well-defined populations allows to visualize biologically relevant targets useful for development of novel TB diagnostics and vaccines.

## Introduction

Serum antibody-based target identification has been extensively used to identify tumor-associated antigens (TAAs) for development of anti-cancer vaccines and early diagnostic markers. cDNA tumor expression libraries (SEREX, serological analysis of recombinant cDNA expression libraries) were instrumental in identifying humoral targets which were further tested for T-cell recognition in patients with cancer [1]. B-cell antigens, and humoral and cellular targets appeared to be closely linked in malignant disease: the majority of TAAs have been identified using SEREX and proved to be indicative of CD4+ and CD8+ T-cell responses [2], [3], [4]. A similar approach can be helpful to identify biologically relevant and clinically meaningful targets in *M.tuberculosis* infection for diagnosis or TB vaccine development [5]. Comprehensive testing of immune recognition in arrayed MTB antigens in a clinically well defined population will help to reveal the profile of a successful protective immune response, most likely associated with CD4+ and CD8+ anti-MTB responses [6], [7], [8], [9], [10] in individuals capable of containing MTB infection. More recent studies have emphasized the usefulness of antibody-based diagnostics in TB and although these have been extensively tested in low-income countries, they did not deliver sufficient accuracy and sensitivity since humoral immune responses may depend on the individual and test sensitivity can vary [11], [12], [13]. In most cases, these tests gauge antibody responses using single recombinant TB antigens. The remedy to limited MTB target testing would be the implementation of protein arrays, as recently reported for autoantigens recognized by sera from patients suffering from autoimmune diseases [14] . Expression of recombinant antigens is time-consuming and challenged by the need for correct folding of the target antigen. An alternative approach represents the construction of a high-content peptide microarray which displays a comprehensive set of MTB antigens in the form of linear peptide stretches without ‘pre-meditated’ target-selection. This approach enables a detailed epitope profiling of the humoral immune response and defines ‘hotspots’ of antibody recognition in clinically well defined patient cohorts. Since T-cells are instrumental in mediating anti-MTB responses, we examined IgG responses, whose presence implies T-cell recognition.

## Results

### Serum profile using MTB peptide microarray analysis: differential target recognition

**Figure 1:**
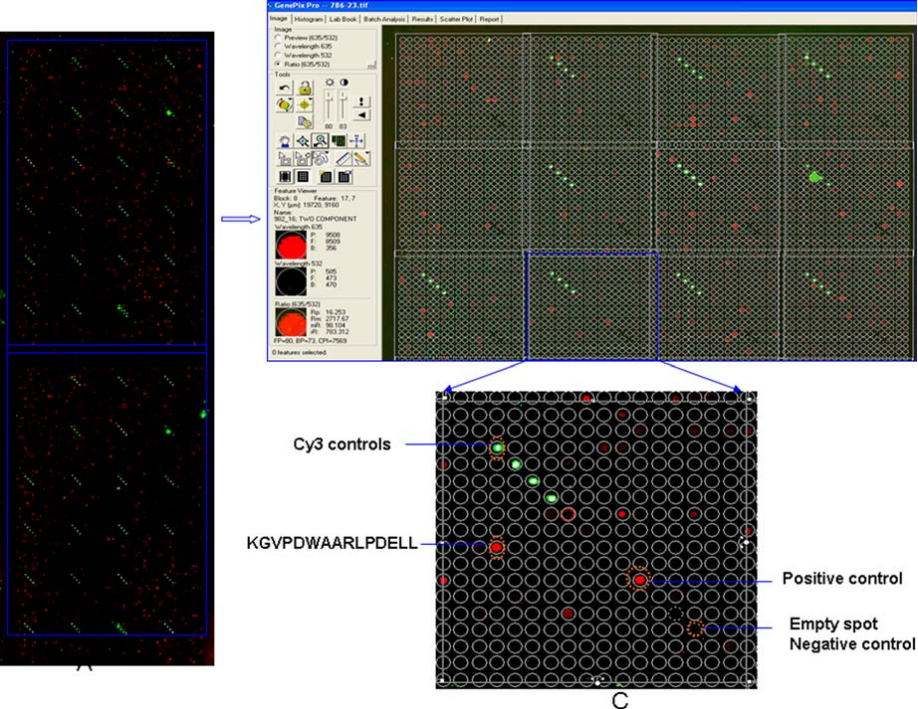
Overview of peptide microarry chip analysis. The analysis platform (left) consists of two identical sub-arrays, each with 7776 spots arranged in 24 blocks, each block has 324 spots, and these are arranged in columns and rows of 18. The 7776 peptide spots represent 7446 unique peptides, 153 negative control spots, 96 Cy3 controls for GAL file orientation and 24 positive controls spots (4 repetitions each of IgG, IgA, IgM, IgE). Plasma is tested for IgG binding to peptides and the slide is scanned with the GenPix 4000B microarray scanner (Axon Instruments with the features described in the method section. A magnification (right) shows the Cy3 controls in order to orient the Gal file (mask) which allows the identification of the aa sequence of each peptide printed in a designated location. Positive controls serve to detect the function of the secondary reagent, empty spots are devoid of peptides, serum antibody-peptide antigen complexes are visualized using the appropriate secondary reagent. The plot shows a representative positive result with the target epitope sequence identified.

**Figure 2:**
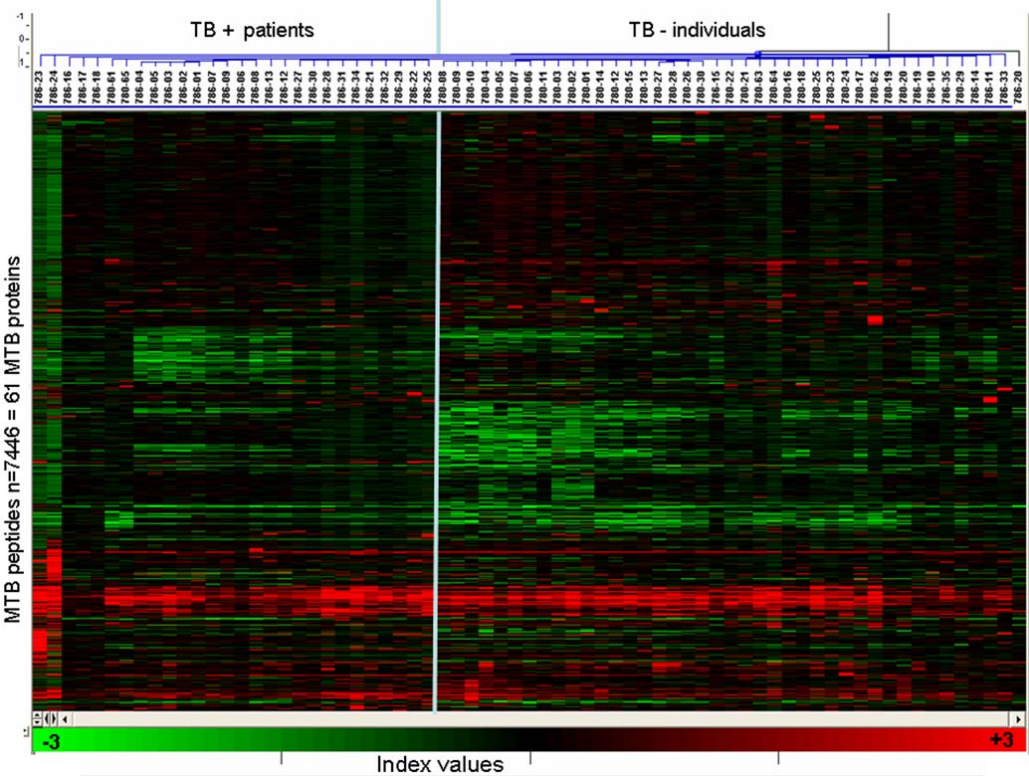
Cluster analysis of the normalized IgG responses from TB+ and TB− individuals Spots flagged as “bad” and false positive responses were excluded.

**Figure 3:**
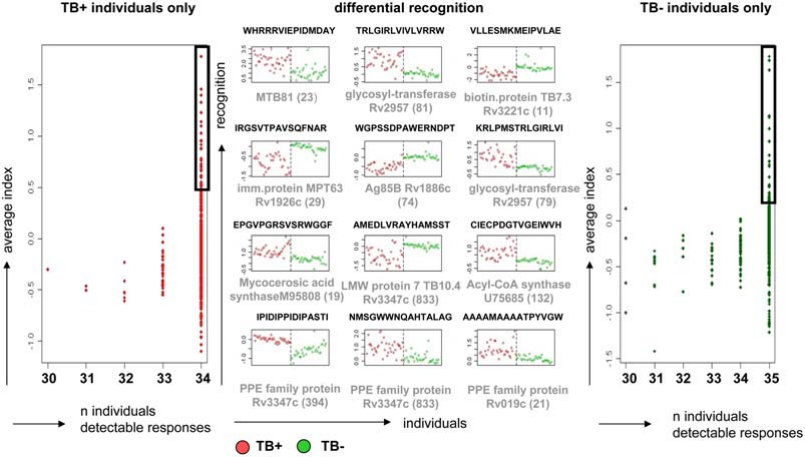
IgG recognition of MTB epitopes segregates TB+ (marked in red) and TB− (marked in green) individuals. The left panel shows the peptides that gave a detectable response for 34/34 individuals with TB but were never recognized in any sample from 35 TB− individuals. Conversely, the right panel shows the responses of peptide epitopes that were recognized by 35/35 TB− individuals but not recognized by any of the 34 TB+ individuals. A predictive analysis using PAM found 89 peptides differentially recognized by TB+ and TB− individuals with a classification error of approximately zero. The center panel plots the ‘top 12’ most strongly predictive of these: the header of each plot shows the peptide sequence, and the corresponding protein with the accession number and the location of the peptide (given in brackets) within the protein is listed under each plot. A compilation of the ‘top 24’ responses (boxed) of the peptides recognized exclusively by TB+ or TB− individuals (left and right panel) segregated by proteins are compiled in Supplementary Table S4 online. Segregation between TB+ and TB− does not cluster into specific recognition of certain MTB *proteins,* but rather into specific peptide epitopes at different locations within the same protein.

**Figure 4:**
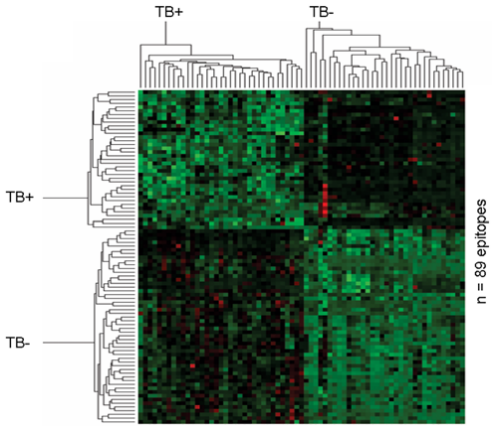
Prediction of TB+ using pattern analysis. Differential IgG responses to 89 peptides (listed in Supplementary Table S2 online) identified by PAM analysis segregate TB+ from TB− individuals. The amino acid sequences of the 89 peptides are listed individually with the corresponding index in the TB+ / TB− groups in the supplementary Table S2.

Sera from 34 individuals with sputum, acid-fast positive, pulmonary TB as well as 35 sera from healthy participants were tested for recognition of 61 MTB proteins (listed with details and segregated according to the MTB life cycle in the Supplementary Table S1 online) in the form of single peptide epitopes. Each peptide was 15aa and showed a 12aa overlap resulting in 7776 epitope spots arranged in 24 blocks on the microarray slide. After incubation with serum, antibody binding to individual peptides was identified (Figure 1), and a cluster analysis of the two groups (TB+ and TB− individuals) was carried out (Figure 2). Quality data was extracted as described in materials and methods, and for each group we normalized the responses of all peptides that were recognized in at least one of the samples. Three individual patterns emerged: Peptides recognized i) exclusively in TB+ individuals ii) exclusively in TB− individuals and iii) peptides which are differentially recognized in the two groups. We identified 1089 peptides that were exclusively recognized in the TB+ group (Figure 3, left panel), 1001 in the TB− group (Figure 3, right panel) and 89 common peptides that were predictive of the groups by PAM analysis (see cluster image in Figure 4) with an classification error of approximately zero. The ‘top 12’ most strongly predictive peptides are shown in Figure 3 (centre panel), and the whole list is provided in Supplementary Table S2 online. These 89 peptides all appeared among the 172 peptides identified by SAM analysis as having significantly higher response in TB+ individuals, and also among the 301 with significantly lower response (Supplementary Table S3 online).

### Cluster analysis of MTB epitopes segregates TB− and TB− individuals

A compilation of the ‘top 24’ peptides recognized in serum from TB+ individuals but not in any sample from TB− individuals are segregated by proteins and compiled in the supplementary Table S4 online. Of note, the segregation between TB+ and TB− does not cluster into specific recognition of certain MTB *proteins,* but rather into specific peptide epitopes at different locations within the same protein. For instance, the highly immunogenic Ag85B mycolylstransferase protein Rv1888c is recognized in different groups: the peptide epitope QSSFYSDWYSPACGK is *exclusively* recognized in 34/34 of the TB+ individuals (and *not* in any of the 35 healthy TB− individuals), while YNGWDINTPAFEWYY and SPACGKAGCQTYKWE are exclusively recognized in 35/35 of the TB− group (Supplementary Tables S2, S3 and S4 online) and WGPSSDPAWERNTDPT is strongly recognized in healthy individuals but only weakly in individuals with acid-fast+ TB (Figure 3, middle panel). Among the strongly recognized linear peptide epitopes are peptides from protein antigens which have been described in the past, e.g. Ag85B, or isocytrate dehydrogenase [15]. We identified additional target peptides from proteins involved in cellular metabolism (glycosyl transferase), lipid-degradation (acyl-CoA Synthase) or lipid formation (e.g. cyclopropane-fatty-acyl-phospholipid synthetase). The latter enzyme is also present in MOTT (mycobacteria other than tuberculosis) and could therefore be recognized in sera from healthy individuals, exposed to MOTT.

### Uniform Ig-recognition pattern in pulmonary TB

**Figure 5:**
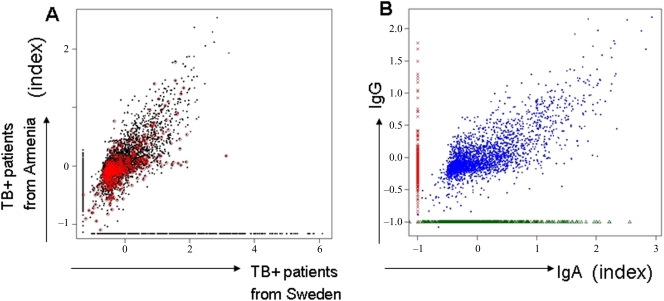
Ig-recognition: Genetic background and IgA/IgG focus. (A) Genetic background of the test population. Normalized responses of 5864 peptides detected in all 34 patients with TB+ from Armenia patients and 4733 peptides detected in all 6 TB+ patients from Sweden. Peptides recognized by one patient group but not the other are assigned the minimum detectable response (approximately −1.0). Peptides that were not recognized in any of the 35 healthy controls are highlighted in red. (B) Differential epitope recognition in IgA and IgG responses in patients with TB. Material from 6 individuals with TB, recruited in Stockholm, was tested for IgA and IgG responses directed against the MTB peptide library. Three groups of peptides are apparent (supplementary Table S6a–c online): those recognized by both IgG and IgA (n = 2544 peptide species, supplementary Table S6a) only by IgG (n = 1703 peptides, supplementary Table S6b), or only by IgA (n = 845 peptides, supplementary Table S6c). For peptides not recognized by IgG, we assigned the minimum detectable response (approximately −1.0) on the plot, and similarly for IgA. Peptides recognized by both IgG and IgA are strongly correlated (r = 0.75).

The formation of IgG is dependent on T-cell help, which is determined by MHC class II-restricted presentation of antigenic peptides. It may very well be that differences in the T-cell ‘immunome’, and the IgG B-cell recognition patterns the TB+ and TB− subjects presented in the current study is due to their different genetic background and different exposure to environmental bacterial species. We therefore obtained an additional 6 serum samples from Swedish individuals who presented with pulmonary (acid-fast stain positive) TB in Stockholm, and compared these with the 35 serum samples from the TB− individuals and the 34 samples from the Armenian patients. The average responses of peptides that were detectable in all patients in either the two TB+ groups are presented in Figure 5A, where it can be seen that (i) for peptides that are detected in both groups, the magnitude of the responses are strongly correlated, (ii) this correlation remains high when we limit to peptides that are never recognized in the 35 healthy controls (iii) there is a group of peptides exclusively defined by IgG in patients from Sweden, but not from Armenia, and vice versa, and (iv) approximately half of these exclusive peptides in each group are not recognized in any of the 35 healthy controls. The sets of peptides defined by IgG reactivity in patients (from Armenia and from Sweden), but not in healthy controls have considerable overlap: from among the 100 top peptides exclusively recognized by IgG in TB+ individuals from Armenia, but not in 35/35 TB-negative individuals, we found 60 that were also recognized in the additional six TB+ patients from Sweden (Supplementary Table S5 online). The responses in each patient group were highly coherent: most of the peptides that had a response for *any* patient in a group had a response for all patients in the group: this was true for 5864/5999 peptides for the 34 TB+ patients from Armenia and for 4733/4748 peptides for the 6 patients recruited in Sweden.

The IgA immune responses in the sera from the 6 additional patients showed a different profile than the IgG ‘immunome’: a specific set of peptides were exclusively recognized by IgA and not by IgG and vice versa, but for peptides recognized by both, the responses were strongly correlated (r = 0.75) (Figure 5B and Supplementary Table S6 online).

## Discussion

We studied the serum IgG recognition pattern in patients with TB and used as a paradigm a ‘high content chip’ peptide microarray with 61 MTB proteins as linear peptide stretches. This excludes the identification of conformation-dependent epitopes and non-protein targets, e.g. carbohydrates, glycolipids and fatty acids which may also serve as biologically relevant targets for humoral and cellular immune responses [16]. A recent survey examining TB-related epitope data revealed that 65 percent of the known TB epitopes derive from the top 30 most studied protein antigens, and that 357 humoral responses have been identified [17]. Until now, the most frequent target proteins for T- and B-cell responses are associated with either the cell wall (i.e. Ag85b, PE/PPE) or MTB pathogenicity [17]. Indeed, Ag85B and PPE-protein family members were frequently detected in the three different categories of recognition in our study: (i) exclusively recognized in the TB+ group, (ii) exclusively recognized in the TB− group and (iii) differentially recognized in TB+ vs. TB− individuals (Fig. 3, middle panel). The 89 differentially responding peptides were highly predictive, with a classification error of approximately zero. The immune focus on these proteins may, in part, be biased from the work in the pre-genomic area of MTB research which targeted proteins secreted into culture medium, or proteins which have been extensively used in vaccine trials, e.g. the proteins Esat-6, Ag85B or TB10.4 are more easily available to the research community since they have been expressed as recombinant proteins.

Some of the top 100 peptide epitopes identified by antibody profiling belong to surface-associated MTB proteins (Supplementary tables S2, S3 and S4 online), other targets belong to factors associated with subcellular organization, i.e. epitopes from the RNA polymerase sigma factor F (CAB07069) as well as the Acyl-CoA Synthase which are recognized in TB+ individuals. These targets are interesting in the dormant phase of MTB infection for rational drug design, for example the MTB sigma factor F (sigF) is responsible for transcriptional initiation [18], [19] and Acetyl-CoA represents the central intermediate in the TCA cycle and in fatty and amino acid biosynthesis. The use of carbon through Ac-CoA is critical for survival of non-replicating bacteria, since fatty acids (and not carbohydrates) present the primary source for carbon in granulomas. Targeting drugs to this pathway has been suggested to disrupt the carbon flux necessary for MTB survival [20]. Not a single individual among the 35 healthy subjects recognized any of 100 epitopes from the sigma factor. In contrast, sigF was frequently recognized in serum from the 34 individuals with tuberculosis. Proteins involved in lipid generation and modification, such as the cyclopropane fatty-acyl phospholipid synthase which provides targets for IgG (Fig 3, Supplementary tables S2 and S3 online) and T-cells (defined by IL-2, TNFα and IFNγ production in intracellular cytokine staining, our unpublished data) may add to the list of immune targets associated with MTB pathogenesis. Mycolic acids, modified by cyclopropane synthease, provide major components of the mycobacterial cell wall involved in MTB persistence. Mycolic acids protect mycobateria against injuries, decrease permeability for antibiotics and affect MTB survival within the host phagolysosome [21]. Cyclopropane-mediated alterations of trehalose dimycolate (cord factor) is responsible for proinflammatory reactions in early mycobacterial infection [22].

Most immunology studies are undertaken with the aim of defining diagnostic markers and identifying new MTB vaccines [11], [23], [24]. The cornerstone of studies that investigate the association between immune-profiling and clinical events is a well-defined study population. This is very challenging in the context of TB: although patients with clinical TB were characterized by pulmonary tests (acid-fast stain positive TB), the history of the infection could differ from patient to patient. The positive stain could represent a recently acquired infection or activation of an infection aquired in the past. Exposure to environmental bacteria as well as BCG vaccination may also alter the response to MTB proteins. The same is true for TB− individuals: in our study, these had not received BCG vaccination, had tested negative in the tuberculin skin test (TST) and the Quantiferon test. However, we cannot exclude exposure to environmental mycobacterial species, e.g. *M. fortuitum, M smegmatis.* Formally, we also cannot exclude previous exposure to MTB which may have resulted in a clearance of the infection (the most likely case in immune-competent individuals) in the absence of a positive TST and Quantiferon test at the time of the blood draw. We performed a Blast search of the top 12 peptides exclusively recognized in TB-negative individuals. If greater variation with the query peptides was allowed concerning the peptide length, matches with peptides derived from non-mycobacterial species were obtained. This would suggest that cross-recognition would account for anti-peptide specific humoral immune responses in the TB− cohort, it could also imply that ‘heterologous immunization’ may contribute for the epitope recognition pattern in TB− individuals, which is absent in the TB+ cohort. Antigen recognition signatures in a healthy population will also define individuals who have been exposed to MTB but who were able to fight off the infection, worthwhile study subjects for vaccine developers.

A significant difference between our 2 main groups (TB+ and TB− individuals) is not only the geographic location (Armenia and the US), but also the genetic background. Considering these caveats, it is surprising that additional serum samples obtained from individuals testing positive for TB in Sweden shared 60/100 peptide epitopes which were exclusively recognized in 34/34 sera from the patients with TB from Armenia (supplementary Table S5 online). It is also important to note that none of the peptide epitopes which are exclusively recognized in the TB-negative population were recognized by 6/6 serum samples from patients with TB-infection recruited in Stockholm. In addition, most of the peptides recognized by any of the patients in a group were recognized by all. This suggests that TB is most likely associated with a rather uniform epitope target recognition. In order to develop the peptide chip described in the current report for diagnostic purposes, appropriate clinically well defined cohorts need to be analyzed. For instance, the control sera are from PPD negative, quantiferon- negative subjects. The use of sera from absolutely ‘clean controls’ from a non-endemic country and advanced TB patients has been the major source of problems in previous efforts to devise a diagnostic assay for TB. The PPD+ subjects from the same geographical area as the patients will certainly aid to define clinically meaningful biomarkers. However, the fact that PPD-, ‘clean’, individuals exhibit a uniform recognition pattern of a defined set of ‘MTB epitopes’, suggests that ‘cross-recognition’, exposure to MOTT and to other bacterial or viral species (see supplementary Table S7 online) shapes the immune recognition profile even in low endemic environments, and most likely also the immune response to BCG and MTB.

The biological underpinning of differential peptide epitope recognition could be twofold: first, differential recognition of MTB-associated proteins may be dependent on antigen accessibility and the nature of presentation. MTB has recently been shown to access the cytosol of host cells, while BCG or MOTT are unable to do so [25]. This may in part explain why certain immune epitopes from the *same protein* are recognized exclusively in TB+ and not in TB− individuals. Second, there is a vast literature concerning ‘crossreactive’ antibodies that may recognize very similar epitopes from unrelated targets [26], [27] or from closely related proteins, e.g. TB10.3 and TB12.9 in the case of peptides derived from TB10.4 [28]. Differential profiling of IgA and IgG, due to the different half life of the immunoglobulin (IgA has a half life of 6 days) may aid in dissecting the nature and specificity of the immune response at the time of the blood draw (Fig. 5*B* and Supplementary table S6 online). IgA-mediated immune recognition of MTB target proteins may also be helpful in designing assays to gauge anti-MTB recognition profiles in sputum, or to design MTB vaccines targeting protective immune responses on mucosal surfaces. In conclusion, high content peptide microarray antibody profiling represents a powerful tool to visualize the global B-cell response for diagnostics and vaccine candidates.

## Materials and Methods

### Patients and slide preparation

Slide production has recently been reported in detail [29]. Slides with MTB epitopes in the current study were manufactured by JPT, Germany and consist of two identical subarrays, each with 7776 spots arranged in 24 blocks of 324 spots arranged in columns and rows of 18. The 7776 peptide spots represent 7446 unique peptides, 153 negative control spots, 96 Cy3 controls for GAL file orientation and 24 positive controls spots (4 repetitions each of IgG, IgA, IgM, IgE). A single slide was prepared using serum from each of the study subjects; 34 patients who tested positive for active pulmonary TB (defined by acid fast stain, AFS, in sputum) from the Medical Yerevan State university hospital in Armenia who received routine BCG vaccination in childhood, 6 patients with TB (defined by AFS in sputum and MTB culture) from the Karolinska Hospital Huddinge and 35 healthy individuals prior to BCG vaccination (testing negative in the tuberculin skin test (TST) and the Quantiferon-test) at Saint Louis University, USA. Ethical approval was obtained from the Stockholm South ethical committee (Dnr 238/02) for use of the specimens from the Karolinska Hospital Huddinge, and from St Louis University, USA (number 12968), for use of the samples provided from healthy individuals. Ethical approval for the analysis of serum samples from the Armenian patients is filed at the University of Mainz, Germany (837.327.99-2271). A further 13 slides (7 from the batch used for the TB patients, and six from the batch used for the healthy subjects) were prepared using only buffer and secondary antibody, in order to help identify peptides giving a ‘false positive’ response, which were removed for further analysis (see below). Serum obtained from patients with TB or from the healthy controls was diluted 1∶100 using a buffer consisting of PBS, 3% FCS and 0,5% Tween and pipetted onto the slide (300 microliter), on which the incubation area was defined using a liquid blocker pen before the slide was covered with a cover slip. Plasma and serum yielded identical results (data not shown). The slide was incubated at 4°C in a humid chamber for 16 hours. On day 2, the cover slip was removed and the slide washed five times (two times rotating in washing solution for 5 minutes, two times rotating in sterile water for 5 minutes, and finally one rotation in filtered Milli Q water for 5 minutes). The slide was tapped on dry tissue to remove droplets and 300 µl of the polyclonal goat anti-human IgG, heavy and light chain specific, affinity purified Cy5-labeled secondary reagent (Abcam, cat no: 6561-100, diluted to 1∶500) was pipetted at one end of the frame and a cover slip was carefully applied. Anti-human IgA was a human IgA alpha chain specific, affinity purified rabbit secondary reagent labeled with Cy3 (Jackson ImmunoResearch, USA, cat no 309-165-01) and diluted 1∶500. After this step, all work was performed in the dark. Incubation with the secondary reagent took place for 1 hour at room temperature in a humid chamber, the 5 washing steps were repeated, and the slide was dried using a slide centrifuge (Euro Tech, UK) for 10 seconds.

### Scanning and analysis

Each slide was scanned with the GenPix 4000B microarray scanner (Axon Instruments) at two wavelengths, 532 and 635 nm, and the images were saved in TIFF and JPG formats.

Image analysis was performed utilizing the circular feature alignment of the GenePix Pro 6.0 software and Genepix Array List (GAL) files and the following criteria were used to flag spots with non-uniform foreground or background signal for IgG detection:

([F635 Mean]>(1.5*[F635 Median])) AND ([F635 Median]>40)

OR

([B635 Mean]>(1.5*[B635 Median])) AND ([B635 Median]>40)

For IgA detection, these same criteria were applied, but with 635 replaced by 532. In addition to these ‘bad’ spots, GenePix also flagged spots as “not-found” and “empty”, resulting in four types of spots: ‘good’ or ‘non-flagged’ spots (labelled as ‘0’), ‘bad spots’ (labelled as ‘−100’), not-found spots (labelled as ‘−50’), and empty spots (labelled as ‘−75’). The digitized image from each sub-array was saved as a GenePix Result (GPR) file and the median foreground and background intensities for the 635nm wavelength from individual peptide spots were used for further analysis of the IgG responses, and the median foreground and background intensities of the 532nm wavelength were used for the IgA responses. All GPR files were saved in a common folder and imported into R/Bioconductor using the *read.GenePix* function from the *marray* R/Bioconductor package.

### Quality data extraction

To examine the quality of the data, we examined the distribution of the flags. We performed this quality control exercise for each group, first by combining the data from all the subarrays and inspecting all spots regardless of whether they were from control or peptide spots, and subsequently stratifying by the type of feature. The individual subarray images, produced with the *Image* function in Bioconductor, were also visually inspected to check whether there were any strange or aberrant subarrays that should not be included in the analysis. The ratio of median foreground to background (on a log scale) was chosen as the measure of the strength of the response. The values of this response index were computed for all spots with background greater than zero (those with zero background, and thus undefined index, were noted and excluded). The data for each of the seven groups of slides (IgG responses from TB+ patients from Armenia, IgG and IgA from TB+ patients from Sweden, IgG from healthy subjects, and IgG from three groups of control slides as listed above) were arranged in a large matrix with identifiers for slide, subarray, and block, and these master datasets used in all analyses described below.

### Data reduction

The volume of data was reduced while maintaining all the important information. Removing spots flagged as ‘not found’ would result in a drastic reduction of the data volume, as many spots have intensity values not substantially different from experimental noise. However, low responding spots on some slides can be informative if they represent peptides that have a high response on one or more of the other slides. Thus, we removed only the “not-found” spots with high intensity and the spots with no ‘detectable’ response on any slide, where the distribution of the negative controls was used to define a cut-off for a detectable response as follows: for each slide, we examined first the negative control responses on a scatterplot of the index vs. the log-background[29] to identify and eliminate any outliers. The negative control responses on all slides in a group were then normalized to remove the effects of slide, sub-array, and block. We performed this normalization using the simple linear model: Y_ijk_ = slide_i_+subarr_j_+block_k_ where Y_ijk_ is the response (i.e. the index) for block *k* in sub-array *j* on slide *i*. The model was run using the *lm* function in R, and from the mean and SD of the normalized values (i.e. the residuals from the regression model) we defined a threshold for a detectable normalized response as t = mean+2SD. Every spot with at least one detectable response on one slide was retained in the analysis.

### Identification of false positive peptide responses

Since no peptides are expected to give a detectable response on a slide with only buffer and secondary antibody, the responding peptides on the buffer slides were considered as false positives. We normalized all the valid (i.e. unflagged) peptide responses on these slides using the same linear model as above applied to each group in turn. From a scatter plot of the normalized indices vs. normalised background, the false positives were identified and excluded in the analysis of the patient data.

### Analysis of peptide responses

For each group of patient slides, we used the cut-off from the negative controls to select all detectable responses for all unflagged peptides on each slide. If a peptide had no detectable response on any slide it was excluded from further analysis. All other peptides had all their responses included i.e. any peptide that had a detectable response on at least one slide had its responses from all slides analyzed, whether or not these were above or below the cut-off. Any peptides defined as false positive by the analysis of the buffer slides were excluded, and the remaining peptide responses were normalized using the model Y_ijk_ = slide_i_+subarr_j_+block_k_ where Y*ijk* is the response (i.e. the index) from block *k* in sub-array *j* on slide *i*. This model was fit using the *biglm* R-package to accommodate the much larger dimension of the data. Since inclusion of a ‘peptide effect’ term in the model was computationally intractable, we estimated the peptide effects as the differences between the observed responses and the responses estimated by the model shown (i.e. the residuals). Since the systematic effects of slide, subarray, and block have been removed, we refer to these as the ‘normalized responses’, and we use them as input data for further analysis (differential expression or predictive analysis). For the peptides that had replications, their normalized values were averaged to produce a list of unique peptides with their normalized values for each slide. The normalized values for each peptide and slide were stored in one expression matrix for each group.

### Significance and predictive analysis

For each of the groups in the following comparisons, we identified the peptides that had a normalized response on each slide in the group, and the peptides that were not common to the two groups: a) Armenian TB+ vs. controls (IgG), b) Swedish TB+ vs. controls (IgG), c) Swedish TB IgG vs. Swedish TB IgA. For a) and b), we identified the common peptides for differential expression analysis, and a two-group comparison was then carried out using the SAM library in R[30], and a parallel predictivity analysis was performed using the PAM library[31]. The proteins from which the peptides are derived were identified using the GAL file, although peptide sequences could also be submitted to any online data bank. For all three comparisons (a, b, c) peptides present in only one of the groups were identified and ranked by the strength of their responses and the number of replications of the peptide in the group, and these two quantities illustrated on a plot. For comparison of IgG and IgA responses (IgG responses using the red channel, and IgA responses using the green channel) we present the normalized index values in each group on a scatter plot (Fig 5(b)) to identify strongly recognized IgA and IgG target epitopes.

## Supporting Information

Table S1 Compilation of MTB proteins displayed as linear peptide stretches on a peptide microarray chip
(0.09 MB PDF)

Table S2 89 peptides predictive of TB+ (n = 34) vs TB− (n = 35) by PAM analysis
(0.02 MB PDF)

Table S3 Peptides predictive of TB+ (n = 34) vs TB− (n = 35) by SAM analysis
(0.08 MB PDF)

Table S4 Top 24 peptides recognized by TB+ patients(n = 35) but not by TB− controls (n = 34) and vice versa
(0.02 MB PDF)

Table S5 Comparison of MTB peptides defined by IgG in patients from Armenia and from Sweden.
(0.19 MB PDF)

Table S6 S6a: MTB Peptides recognized by both IgG and IgA, S6b: MTB Peptides recognized by IgG and not by IgA, S6c: MTB Peptides recognized by IgA and not by IgG.
(0.75 MB PDF)

Table S7 Blast search of the top 12 peptide exclusively recognized in healthy, PPD-, Quantiferon-negative individuals. Blast search of the top 12 peptides exclusively recognized in TB-negative individuals. The peptide amino acid sequence and peptide number as well as the protein ID and Rv numbers are provided. The search allowed for at most two amino acids variation from the query peptide except in some few cases highlighted with a star (greater variation as compared with the query peptide). If greater variation with the query peptides was allowed concerning the peptide length, more matches with peptides derived from non-mycobacterial species were obtained. Amino acid differences are marked in red. A detailed blast search covering all possible permutations of these peptides, followed by targeted amino acid substitutions and subsequent serum recognition analysis will aid to define immunogenicity.
(0.02 MB PDF)

## References

[1] Lee SY, Jeoung D (2007). The reverse proteomics for identification of tumor antigens.. J Microbiol Biotechnol.

[2] Jager D, Karbach J, Pauligk C, Seil I, Frei C (2005). Humoral and cellular immune responses against the breast cancer antigen NY-BR-1: definition of two HLA-A2 restricted peptide epitopes.. Cancer Immun.

[3] Odunsi K, Qian F, Matsuzaki J, Mhawech-Fauceglia P, Andrews C (2007). Vaccination with an NY-ESO-1 peptide of HLA class I/II specificities induces integrated humoral and T cell responses in ovarian cancer.. Proc Natl Acad Sci U S A.

[4] Odunsi K, Old LJ (2007). Tumor infiltrating lymphocytes: indicators of tumor-related immune responses.. Cancer Immun.

[5] Kaufmann SH, Parida SK (2008). Tuberculosis in Africa: learning from pathogenesis for biomarker identification.. Cell Host Microbe.

[6] Tully G, Kortsik C, Hohn H, Zehbe I, Hitzler WE (2005). Highly focused T cell responses in latent human pulmonary Mycobacterium tuberculosis infection.. J Immunol.

[7] Mueller H, Detjen AK, Schuck SD, Gutschmidt A, Wahn U (2008). Mycobacterium tuberculosis-specific CD4(+), IFNgamma(+), and TNFalpha(+) multifunctional memory T cells coexpress GM-CSF.. Cytokine.

[8] Jacobsen M, Detjen AK, Mueller H, Gutschmidt A, Leitner S (2007). Clonal expansion of CD8+ effector T cells in childhood tuberculosis.. J Immunol.

[9] Andersen P, Smedegaard B (2000). CD4(+) T-cell subsets that mediate immunological memory to Mycobacterium tuberculosis infection in mice.. Infect Immun.

[10] Mueller H, Detjen AK, Schuck SD, Gutschmidt A, Wahn U (2008). Mycobacterium tuberculosis-specific CD4+, IFNgamma+, and TNFalpha+ multifunctional memory T cells coexpress GM-CSF.. Cytokine.

[11] Andersen P, Munk ME, Pollock JM, Doherty TM (2000). Specific immune-based diagnosis of tuberculosis.. Lancet.

[12] Lyashchenko K, Manca C, Colangeli R, Heijbel A, Williams A (1998). Use of Mycobacterium tuberculosis complex-specific antigen cocktails for a skin test specific for tuberculosis.. Infect Immun.

[13] Pottumarthy S, Wells VC, Morris AJ (2000). A comparison of seven tests for serological diagnosis of tuberculosis.. J Clin Microbiol.

[14] Robinson WH, DiGennaro C, Hueber W, Haab BB, Kamachi M (2002). Autoantigen microarrays for multiplex characterization of autoantibody responses.. Nat Med.

[15] Banerjee S, Nandyala A, Podili R, Katoch VM, Murthy KJ (2004). Mycobacterium tuberculosis (Mtb) isocitrate dehydrogenases show strong B cell response and distinguish vaccinated controls from TB patients.. Proc Natl Acad Sci U S A.

[16] Meena LS, Goel S, Sharma SK, Jain NK, Banavaliker JN (2002). Comparative study of three different mycobacterial antigens with a novel lipopolysaccharide antigen for the serodiagnosis of tuberculosis.. J Clin Lab Anal.

[17] Blythe MJ, Zhang Q, Vaughan K, de Castro R, Salimi N (2007). An analysis of the epitope knowledge related to Mycobacteria.. Immunome Res.

[18] Lee JH, Geiman DE, Bishai WR (2008). Role of stress response sigma factor SigG in Mycobacterium tuberculosis.. J Bacteriol.

[19] Lee JH, Karakousis PC, Bishai WR (2008). Roles of SigB and SigF in the Mycobacterium tuberculosis sigma factor network.. J Bacteriol.

[20] Michele TM, Ko C, Bishai WR (1999). Exposure to antibiotics induces expression of the Mycobacterium tuberculosis sigF gene: implications for chemotherapy against mycobacterial persistors.. Antimicrob Agents Chemother.

[21] Barry CE, Lee RE, Mdluli K, Sampson AE, Schroeder BG (1998). Mycolic acids: structure, biosynthesis and physiological functions.. Prog Lipid Res.

[22] Rao V, Fujiwara N, Porcelli SA, Glickman MS (2005). Mycobacterium tuberculosis controls host innate immune activation through cyclopropane modification of a glycolipid effector molecule.. J Exp Med.

[23] Andersen P, Doherty TM (2005). The success and failure of BCG - implications for a novel tuberculosis vaccine.. Nat Rev Microbiol.

[24] Kaufmann SH, Baumann S, Nasser Eddine A (2006). Exploiting immunology and molecular genetics for rational vaccine design against tuberculosis.. Int J Tuberc Lung Dis.

[25] van der Wel N, Hava D, Houben D, Fluitsma D, van Zon M (2007). M. tuberculosis and M. leprae translocate from the phagolysosome to the cytosol in myeloid cells.. Cell.

[26] Predki PF, Mattoon D, Bangham R, Schweitzer B, Michaud G (2005). Protein microarrays: a new tool for profiling antibody cross-reactivity.. Hum Antibodies.

[27] Michaud GA, Salcius M, Zhou F, Bangham R, Bonin J (2003). Analyzing antibody specificity with whole proteome microarrays.. Nat Biotechnol.

[28] Skjot RL, Brock I, Arend SM, Munk ME, Theisen M (2002). Epitope mapping of the immunodominant antigen TB10.4 and the two homologous proteins TB10.3 and TB12.9, which constitute a subfamily of the esat-6 gene family.. Infect Immun.

[29] Nahtman T, Jernberg A, Mahdavifar S, Zerweck J, Schutkowski M (2007). Validation of peptide epitope microarray experiments and extraction of quality data.. J Immunol Methods.

[30] Tusher VG, Tibshirani R, Chu G (2001). Significance analysis of microarrays applied to the ionizing radiation response.. Proc Natl Acad Sci U S A.

[31] Tibshirani R, Hastie T, Narasimhan B, Chu G (2002). Diagnosis of multiple cancer types by shrunken centroids of gene expression.. Proc Natl Acad Sci U S A.

