# Supplementary material for: Pattern Recognition in Pulmonary Tuberculosis Defined by High Content Peptide Microarray Chip Analysis Representing 61 Proteins from M. tuberculosis
Source: PLoS One. 2008 Dec 9;3(12):e3840. doi: 10.1371/journal.pone.0003840 (PMC2588537; doi:10.1371/journal.pone.0003840)
Supplement: Table S1 — Compilation of MTB proteins displayed as linear peptide stretches on a peptide microarray chip (0.09 MB PDF) [file pone.0003840.s001.pdf]

Supplementary Table 1. MTB target proteins associated with the Mtb life cycle.

Protein Group	Protein name	Protein Function and Immunology
Dormancy and stress related	HEAT SHOCK PROTEIN HSP CAA17343 Rv0251c	Thought to be involved in the initiation step of translation at high temperature. Bounded to 30s ribosomal subunit. Possibly a molecular chaperone.
	HEAT SHOCK PROTEIN HSPX CAA17245 Rv2031c	Stress protein induced by anoxia. Has a proposed role in maintenance of long-term viability during latent, asymptomatic infections, and a proposed role in replication during initial infection. Belongs to the small heat shock protein (hsp20) family[13].
	60 KDA CHAPERONIN 2 P0A521 Mb0448	Prevents misfolding and promotes the refolding and proper assembly of unfolded polypeptides generated under stress conditions.<br>This protein is one of the major immunoreactive proteins of the mycobacteria, contains epitopes that are common to various species of mycobacteria.
	LOW MOLECULAR WEIGHT T-CELL ANTIGEN TB8.4 NP_215690 Rv1174c	Unknown, suggested to be involved in reactivation of dormant mycobacteria[16]. May also be involved in mechanisms of survival against intracellular killing and in adaptation to hypoxic conditions[17].
	HYPOTHETICAL PROTEIN NP_217139 RV2623	Unknown<br>a small-molecular-weight chaperonin capable of protecting proteins from heat-induced denaturation and known to be associated with cell wall thickening, may protect essential *M. tuberculosis* functions from host insult[14].
	HYPOTHETICAL PROTEIN NP_217142 RV2626C	Unknown<br>One of the Mtb proteins more abundant under low-oxygen conditions [13]
	CONSERVED HYPOTHETICAL PROTEIN CAB08634 Rv2629	Unknown<br>One of the MTB proteins identified to be more abundant during anaerobic conditions[36]
Cell wall - associated	SECRETED L-ALANINE DEHYDROGENASE ALD CAA15575 Rv2780	May play a role in cell wall synthesis as l-alanine is an important constituent of the peptidoglycan layer.
	SECRETED ANTIGEN 85-B FBPB (85B) CAB10044 Rv1886c	Involved in cell wall mycoloylation. Proteins of the antigen 85 complex are responsible for the high affinity of mycobacteria to fibronectin. Possesses amycoly ltransferase activity required for the biogenesis of trehalose dimycolate (cord factor), a dominant structure necessary for maintaining cell wall integrity.<br>Is immunogenic, and induces expansion and differentiation of TCRVbeta11(+)CD4(+) T cells to IFN-gamma-producing cells in C57BL/6 (I-A(b)) mice[33].<br>Peptide-25 (aa240-254) of Ag85B is a major Th1 cell epitope in I-A(b) mice. Active immunization of C57BL/6 mice with Peptide-25 can induce the development of CDT4(+) TCRVbeta11(+) and CDT4(+) TCRVbeta11(-)Th1 cells that produce IFN-gamma- and TNF-alpha, and protects against subsequent infection with live Mycobacterium tuberculosis H37Rv IFN-gamma[34].
	IRON-REGULATED CONSERVED HYPOTHETICAL PROTEIN CAB08889 Rv1636	Unknown<br>Present in cell wall preparations of *M. tuberculosis* proteins and found to be a good seroantigen[9].
Lipid and fat metabolism	MYCOBACTERIUM BOVIS ACYL-COA SYNTHASE GENE; U75685	Unknown, but involved in lipid degradation[20].
	MYCOBACTERIUM BOVIS MYCOCEROSIC ACID SYNTHASE GENE M95808	Catalyzes the elongation of n-fatty acyl-coa with methylamalonyl-coa (not malonyl-coa) as the elongating agent to form mycocerosyl lipids[21].
	PUTATIVE CYCLOPROPANE-FATTY-ACYL-PHOSPHOLIPID SYNTHASE UFAA1 NP_854118 Mb0455c	Transfers a methylene group froms-adenosyl-l-methionine to the cis double bond of an unsaturated fatty acid chain resulting in the replacement of the double bond with a methylene bridge [catalytic activity: S-adenosyl-L-methionine + phospholipid olefinicfatty

		acid = S-adenosyl-L-homocysteine + phospholipidcyclopropane fatty acid].
	3-OXOACYL-[ACYL-CARRIER PROTEIN] SYNTHASE 2 KASB CAA94642 Rv2246	Involved in fatty acid biosynthesis (mycolic acids synthesis); involved in meromycolate extension. Catalyzes the condensation reaction of fatty acid.
Cellular processes / housekeeping	ALTERNATE RNA POLYMERASE SIGMA FACTOR SIGF CAB07069 Rv3286c	Is an initiation factor that promotes attachment of the RNA polymerase to specific initiation sites and then is released. Thought to be involved in survival and proliferation in lung granulomas during infection. Thought to be involved in virulence and persistence processes. Induced by a number of in vitro stress conditions, including temperature, oxidative, and stationary-phase stress[5].
	10 KDA CHAPERONIN GROES CAB01005 Rv3418c	Binds to CPN60 in the presence of Mg-ATP and suppresses the ATPase activity of the latter.
	PERIPLASMIC PHOSPHATE-BINDING LIPOPROTEIN PSTS1 YP_177770 Rv0934	Involved in active transport of in organic phosphate across the membrane (import). This is one of the proteins required for binding-protein-mediated phosphate transport. recognized only by cavitary TB patients' sera, thus providing several antigens that may be useful in demarcating cavitary and noncavitary TB patients[22].
	PERIPLASMIC PHOSPHATE-BINDING LIPOPROTEIN PSTS2 YP_177769 Rv0932c	Involved in active transport of inorganic phosphate across the membrane (import). This is one of the proteins required for binding-protein-mediated phosphate transport. One of the most immunogenic lipoproteins found in culture filtrate gauged by antibodies response from Tb infected patients[23].
	PERIPLASMIC PHOSPHATE-BINDING LIPOPROTEIN PSTS3 YP_177768 Rv0928	Involved in active transport of inorganic phosphate across the membrane (import). This is one of the proteins required for binding-protein-mediated phosphate transport.
	POSSIBLE GLYCOSYL TRANSFERASE CAB05418 Rv2958c	Unknown<br>Probably involved in cellular metabolism. Possibly involved in resistance to killing by human macrophages.
	POSSIBLE GLYCOSYL TRANSFERASE CAB05419 Rv2957	Unknown<br>Probably involved in cellular metabolism
	POSSIBLE GLYCOSYL TRANSFERASE CAB05415 Rv2962c	Unknown<br>Probably involved in cellular metabolism. Possibly involved in resistance to killing by human macrophages
	PROBABLE 50S RIBOSOMAL PROTEIN L7/L12 RPLL CAB07109 Rv0652	Involved in translation mechanisms: seems to be the binding site for several of the factors involved in protein synthesis and appears to be essential for accurate translation.
	PROBABLE CUTINASE PRECURSOR CFP21 NP_216500 Rv1984c	Hydrolyzes cutin<br>Immunological very active[28].
	PROBABLE ISOCITRATE DEHYDROGENASE CAA17111 Rv3339c	Involved in the krebs cycle [catalytic activity: isocitrate + NADP(+) = 2-OXOGLUTARATE + CO(2) + NADPH][29].<br>Elicit a strong B cell response in TB-infected populations and can differentiate between healthy BCG-vaccinated populations and those with TB[30].
	PROBABLE ISOCITRATE DEHYDROGENASE CAA16247 Rv0066c	Involved in the krebs cycle [catalytic activity: isocitrate + NADP(+) = 2-OXOGLUTARATE + CO(2) + NADPH][29].<br>Elicit a strong B cell response in TB-infected populations and can differentiate between healthy BCG-vaccinated populations and those with TB[30].
	PROBABLE LIPOPROTEIN LPRJ CAB10947 Rv1690	Unknown. Contains possible signal sequence and PS00013 Prokaryotic membrane lipoprotein lipid attachment site.
	PROBABLE MOLYBDOPTERIN-GUANINE DINUCLEOTIDE BIOSYNTHESIS PROTEIN CAA16030 Rv2453c	Ivolved in molybdenum cofactor biosynthesis. Links a guanosine 5'-phosphate to molydopterin (MPT)forming molybdopterin guanine dinucleotide (MGD)
	PROBABLE SERINE PROTEASE PEPA	Unknown; possibly hydrolyzes peptides and or proteins (seems to cleave preferentially

	CAB09453 Rv0125	after serine residues).
	PROBABLE SERINE PROTEASE PEPD CAA17582 Rv0983	Unknown; possibly hydrolyzes peptides and/or proteins (seems to cleave preferentially after serine residues.
	RNA POLYMERASE BETA-SUBUNIT AAA21416 L27989	Catalyzes the transcription of DNA into RNA using the four ribonucleoside triphosphates as substrates [catalytic activity: n nucleoside triphosphate = n diphosphate + [31](n)]. 127-135 area is a CD8 CTL epitope binding to HLA A*0201[32].
	RNA POLYMERASE BETA'-SUBUNIT AAA21417 L27989	Catalyzes the transcription of DNA into rna using the four ribonucleoside triphosphates as substrates [catalytic activity: n nucleoside triphosphate = n diphosphate + [31](n)]."
	TRANSMEMBRANE SERINE/THREONINE-PROTEIN KINASE D PKND NP_215446 Rv0931c	Involved in signal transduction (via phosphorylation). Thought to regulate phosphate transport. Can phosphorylate the peptide substrate myelin basic protein (mbp) at serine and threonine residues. Can be autophosphorylated on threonine residues [catalytic activity: atp + a protein = adp + a phosphoprotein]."
	TWO COMPONENT TRANSCRIPTIONAL REGULATORY PROTEIN DEVR NP_217649 Rv3133c	Regulator part of the two component regulatory system devr/devs. Controls hspx|rv2031|acr expression.
Secreted antigens and immunogenic proteins of unknown function	BIOTINYLATED PROTEIN TB7.3 CAB08316 Rv3221c	Unknown<br>Recognised at a low level by pbmcs of TB infected patients[6].
	CELL SURFACE LIPOPROTEIN MPT83 CAB08316 Rv2873	unknown<br>Vaccination with DNA or RNA constructs expressing the *M. tuberculosis* MPT83 antigen are capable of inducing specific humoral and T-cell immune responses and confer modest but significant protection against *M. tuberculosis* challenge in mice[7].
	10 KDA CULTURE FILTRATE ANTIGEN ESXB CAA17966 Rv3874	Unknown. Expression in BCG together with ESAT 6 resulted in enhanced protection against Tb[1]. CFP10 distinguished TB patients from *Mycobacterium bovis* BCG-vaccinated donors[2].
	IMMUNOGENIC PROTEIN MPT63 CAB06500 Rv1926c	Unknown<br>Elicits specific humoral immune responses in humans with TB[3].<br>T-cell epitope mapping showed that MPT63 contained a highly immunodominant region within the first 30 residues of the amino-terminal of the mature protein[4]
	ESAT-6 LIKE PROTEIN ESXQ CAA16102 Rv3017c	Unknown<br>ESAT-6 like protein, possibly secreted protein, Belongs to the ESAT6 family. Note, previously known as TB12.9[12].
	IMMUNOGENIC PROTEIN MPT64 CAA98382 Rv1980c	UNKNOWN<br>This antigen has not been detected in non-tuberculous mycobacteria. Immunohistochemistry using anti-MPT64 is a simple and sensitive technique for establishing an early and specific diagnosis of *M. tuberculosis* infection and one that can easily be incorporated into routine histopathology laboratories[15].
	MAJOR SECRETED IMMUNOGENIC PROTEIN CAA98373 Rv2875	Unknown<br>Major secreted immunogenic protein MPT70 precursor. Belongs to the mpt70 / mpt83 family. Generally found as a monomer; homodimer in culture fluids.
	MCE-FAMILY PROTEIN MCE1A YP_177701 Rv0169	Unknown, but thought to be involved in host cell invasion (entry and survival inside macrophages).
	MTB48 AAK31576 AY029285	Unknown<br>The inclusion of recombinant MTB48 in a prototype serodiagnostic test increases assay sensitivity for M. tuberculosis infection when it is combined with other known immunodominant antigens, such as the 38-kDa antigen[18].
	MTB81)	Unknown<br>Mtb81 reacted with 26 of 37 HIV(+) TB(+) sera (70%), compared to 2 of 37 (5%) that

Conserved hypothetical proteins of ill-defined function		reacted with the 38-kDa antigen, Mtb81 may be a promising complementary antigen for the serodiagnosis of TB in HIV-1 and TB coinfected patients[19].
	POSSIBLE HEMOLYSIN CAA16235 Rv3922c	Not known<br>Possible hemolysin, highly similar to Q9L7M0|YIDD_MYCPA HYPOTHETICAL 12.4 KDA PROTEIN from Mycobacterium paratuberculosis.
	POSSIBLE HEMOLYSIN-LIKE PROTEIN CAA17201 Rv1085c	Not known, but supposed involved in virulence
	LIPOPROTEIN LPQH PRECURSOR P0A5J0 Rv3763	unknown<br>Possibly attached to the membrane by a lipid anchor similar to other mycobacterium 19 kda antigen. Contains PS00013 Prokaryotic membrane lipoprotein lipid attachment site.
	CONSERVED HYPOTHETICAL PROTEIN CFP17 CAB01474 Rv1827	unknown<br>Immunologically very active and induce either a high IFN-γ release from murine memory effector cells or a pronounced DTH reaction[8].
	CONSERVED HYPOTHETICAL PROTEIN TB16.3CAD97060 Mb2207c	unknown<br>Had a serological sensitivity of 48 to 55% with samples from Danish resident TB patients and a sensitivity of 88 to 98% with samples from African TB patients. Importantly, the TB16.3 antigens were recognized by more than 85% of the samples from TB patients coinfected with human immunodeficiency virus, a patient group for which it is in general difficult to detect *M. tuberculosis*-specific antibodies[9].
	CONSERVED HYPOTHETICAL PROTEIN TB18.5CAD93033 Mb0169	Unknown<br>Induced significant production of IFN-γ and interleukin (IL)-12p40 in peripheral blood mononuclear cells from healthy tuberculin reactors[10].
	CONSERVED HYPOTHETICAL PROTEIN TB9.8 CAD93159 Mb0295	unknown<br>PE-family related protein; distant member of the Mycobacterium tuberculosis PE family[11].
PPE/PE family	PPE FAMILY PROTEIN CAE55371 Rv1196	Not known<br>Found to induce in vitro gamma interferon responses in infected or BCG-vaccinated calves[24].
	PPE FAMILY PROTEIN YP 177963 Rv3347c	Unknown<br>Antibodies to the C-terminal, approximately 100-kDa fragment of the protein were detectable in sera from 29/30 (97%) human immunodeficiency virus-negative/TB-positive (HIV(-) TB(+)) patients and 17/24 (71%) HIV(+) TB(+) patients tested but not in sera from healthy controls, suggesting that the in vivo expression of the protein correlates with active M. tuberculosis infection[25].
	PPE FAMILY PROTEIN CAE55334 Rv0915c	Unknown. Possibly a protective antigen involved with the early control of infection.
	PPE FAMILY PROTEIN CAE55489 Rv2430c	Unknown.<br>Member of the Mycobacterium tuberculosis PPE family. Lacks a transmembrane domain and is therefore likely to be cytosolic or secretory in localization, is an immunodominant B-cell target antigen with apparent diagnostic potential[26].
	PPE FAMILY PROTEIN CAE55504 Rv2608	Unknown<br>Member of the Mycobacterium tuberculosis PPE family.<br>Elicit a high humoral and a low T cell response[27].
	PE FAMILY PROTEIN CAE55335 Rv0916c	PE family. This family named after a PE motif near to the amino terminus of the domain. The PE family of proteins all contain an amino-terminal region of about 110 amino acids.
ESAT-6 family	6 KDA EARLY SECRETORY ANTIGENIC TARGET ESXA (ESAT-6) CAE55648 Rv3875	Unknown.<br>Elicits high level of IFN-gamma from memory effector cells during the first phase of a protective immune response[37, 38].

	SECRETED ESAT-6 LIKE PROTEIN ESXR CAA16104 Rv3019c	Unknown<br>Secreted ESAT-6 like protein. Belongs to the ESAT6 family. Member of a three-member subfamily within the large 23 protein *esat-6* gene family. As well as the immunogenic and protective ESAT-6 protein, the family contains a number of other immunodominant proteins recognised by the immune systems of both humans and cattle. Detailed analysis has revealed Rv3019c also contains several unique T cell epitopes strongly recognised by tuberculosis patients, BCG vaccinees, and infected cattle[35].<br>Note previously known as TB10.3.
	PUTATIVE ESAT-6 LIKE PROTEIN ESXN YP_177838 Rv1793	Unknown<br>ESAT-6 like protein , almost identical to several mycobacterial proteins of the ESAT-6-likefamily including
	LOW MOLECULAR WEIGHT PROTEIN ANTIGEN 7 ESXH TB10.4 CAA17363 Rv0288	Unknown, may be involved in virulence. Belongs to the ESAT 6 family

References

1. Pym, A.S., et al., *Recombinant BCG exporting ESAT-6 confers enhanced protection against tuberculosis.* Nat Med, 2003. **9**(5): p. 533-9.
2. Michel, T., *Targeting and translocation of endothelial nitric oxide synthase.* Braz J Med Biol Res, 1999. **32**(11): p. 1361-6.
3. Lyashchenko, K.P., et al., *Diversity of antigen recognition by serum antibodies in experimental bovine tuberculosis.* Infect Immun, 1998. **66**(11): p. 5344-9.
4. Lee, B.Y. and M.A. Horwitz, *T-cell epitope mapping of the three most abundant extracellular proteins of Mycobacterium tuberculosis in outbred guinea pigs.* Infect Immun, 1999. **67**(5): p. 2665-70.
5. Chen, P., et al., *Construction and characterization of a Mycobacterium tuberculosis mutant lacking the alternate sigma factor gene, sigF.* Infect Immun, 2000. **68**(10): p. 5575-80.
6. Skjot, R.L., et al., *Comparative evaluation of low-molecular-mass proteins from Mycobacterium tuberculosis identifies members of the ESAT-6 family as immunodominant T-cell antigens.* Infect Immun, 2000. **68**(1): p. 214-20.
7. Xue, T., et al., *RNA encoding the MPT83 antigen induces protective immune responses against Mycobacterium tuberculosis infection.* Infect Immun, 2004. **72**(11): p. 6324-9.
8. Weldingh, K., et al., *Two-dimensional electrophoresis for analysis of Mycobacterium tuberculosis culture filtrate and purification and characterization of six novel proteins.* Infect Immun, 1998. **66**(8): p. 3492-500.
9. Weldingh, K., et al., *Assessing the serodiagnostic potential of 35 Mycobacterium tuberculosis proteins and identification of four novel serological antigens.* J Clin Microbiol, 2005. **43**(1): p. 57-65.
10. Lim, J.H., et al., *Identification of the new T-cell-stimulating antigens from Mycobacterium tuberculosis culture filtrate.* FEMS Microbiol Lett, 2004. **232**(1): p. 51-9.
11. Garnier, T., et al., *The complete genome sequence of Mycobacterium bovis.* Proc Natl Acad Sci U S A, 2003. **100**(13): p. 7877-82.
12. Skjot, R.L., et al., *Epitope mapping of the immunodominant antigen TB10.4 and the two homologous proteins TB10.3 and TB12.9, which constitute a subfamily of the esat-6 gene family.* Infect Immun, 2002. **70**(10): p. 5446-53.

13. Rosenkrands, I., et al., *Hypoxic response of Mycobacterium tuberculosis studied by metabolic labeling and proteome analysis of cellular and extracellular proteins.* J Bacteriol, 2002. **184**(13): p. 3485-91.
14. Shi, L., et al., *Expression of Th1-mediated immunity in mouse lungs induces a Mycobacterium tuberculosis transcription pattern characteristic of nonreplicating persistence.* Proc Natl Acad Sci U S A, 2003. **100**(1): p. 241-6.
15. Purohit, M.R., et al., *Immunohistochemical diagnosis of abdominal and lymph node tuberculosis by detecting Mycobacterium tuberculosis complex specific antigen MPT64.* Diagn Pathol, 2007. **2**: p. 36.
16. Zhang, Y., et al., *Resuscitation of dormant Mycobacterium tuberculosis by phospholipids or specific peptides.* Biochem Biophys Res Commun, 2001. **284**(2): p. 542-7.
17. Bottai, D., et al., *The secretion antigen SA5K has a role in the adaptation of Mycobacterium bovis bacillus Calmette-Guerin to intracellular stress and hypoxia.* Microbes Infect, 2006. **8**(8): p. 2254-61.
18. Lodes, M.J., et al., *Serological expression cloning and immunological evaluation of MTB48, a novel Mycobacterium tuberculosis antigen.* J Clin Microbiol, 2001. **39**(7): p. 2485-93.
19. Hendrickson, R.C., et al., *Mass spectrometric identification of mtb81, a novel serological marker for tuberculosis.* J Clin Microbiol, 2000. **38**(6): p. 2354-61.
20. Fitzmaurice, A.M. and P.E. Kolattukudy, *Open reading frame 3, which is adjacent to the mycocerosic acid synthase gene, is expressed as an acyl coenzyme A synthase in Mycobacterium bovis BCG.* J Bacteriol, 1997. **179**(8): p. 2608-15.
21. Mathur, M. and P.E. Kolattukudy, *Molecular cloning and sequencing of the gene for mycocerosic acid synthase, a novel fatty acid elongating multifunctional enzyme, from Mycobacterium tuberculosis var. bovis Bacillus Calmette-Guerin.* J Biol Chem, 1992. **267**(27): p. 19388-95.
22. Sartain, M.J., et al., *Disease state differentiation and identification of tuberculosis biomarkers via native antigen array profiling.* Mol Cell Proteomics, 2006. **5**(11): p. 2102-13.
23. Malen, H., T. Softeland, and H.G. Wiker, *Antigen analysis of Mycobacterium tuberculosis H37Rv culture filtrate proteins.* Scand J Immunol, 2008. **67**(3): p. 245-52.
24. Mustafa, A.S., et al., *Immunogenicity of Mycobacterium tuberculosis antigens in Mycobacterium bovis BCG-vaccinated and M. bovis-infected cattle.* Infect Immun, 2006. **74**(8): p. 4566-72.
25. Singh, K.K., et al., *Immunogenicity of the Mycobacterium tuberculosis PPE55 (Rv3347c) protein during incipient and clinical tuberculosis.* Infect Immun, 2005. **73**(8): p. 5004-14.
26. Choudhary, R.K., et al., *PPE antigen Rv2430c of Mycobacterium tuberculosis induces a strong B-cell response.* Infect Immun, 2003. **71**(11): p. 6338-43.
27. Chakhaiyar, P., et al., *Regions of high antigenicity within the hypothetical PPE major polymorphic tandem repeat open-reading frame, Rv2608, show a differential humoral response and a low T cell response in various categories of patients with tuberculosis.* J Infect Dis, 2004. **190**(7): p. 1237-44.
28. Wang, B.L., et al., *Antibody response to four secretory proteins from Mycobacterium tuberculosis and their complex antigen in TB patients.* Int J Tuberc Lung Dis, 2005. **9**(12): p. 1327-34.
29. Banerjee, S., et al., *Comparison of Mycobacterium tuberculosis isocitrate dehydrogenases (ICD-1 and ICD-2) reveals differences in coenzyme affinity,*

oligomeric state, pH tolerance and phylogenetic affiliation. BMC Biochem, 2005. **6**: p. 20.

30. Banerjee, S., et al., *Mycobacterium tuberculosis (Mtb) isocitrate dehydrogenases show strong B cell response and distinguish vaccinated controls from TB patients.* Proc Natl Acad Sci U S A, 2004. **101**(34): p. 12652-7.
31. Correa, R., et al., *Functional patterns of HIV-1-specific CD4 T-cell responses in children are influenced by the extent of virus suppression and exposure.* Aids, 2007. **21**(1): p. 23-30.
32. Cho, S., et al., *Antimicrobial activity of MHC class I-restricted CD8+ T cells in human tuberculosis.* Proc Natl Acad Sci U S A, 2000. **97**(22): p. 12210-5.
33. Kariyone, A., et al., *Immunogenicity of Peptide-25 of Ag85B in Th1 development: role of IFN-gamma.* Int Immunol, 2003. **15**(10): p. 1183-94.
34. Takatsu, K. and A. Kariyone, *The immunogenic peptide for Th1 development.* Int Immunopharmacol, 2003. **3**(6): p. 783-800.
35. Hogarth, P.J., et al., *Protective immunity against Mycobacterium bovis induced by vaccination with Rv3109c--a member of the esat-6 gene family.* Vaccine, 2005. **23**(20): p. 2557-64.
36. Starck, J., et al., *Comparative proteome analysis of Mycobacterium tuberculosis grown under aerobic and anaerobic conditions.* Microbiology, 2004. **150**(Pt 11): p. 3821-9.
37. Cole, S.T., et al., *Deciphering the biology of Mycobacterium tuberculosis from the complete genome sequence.* Nature, 1998. **393**(6685): p. 537-44.
38. Camus, J.C., et al., *Re-annotation of the genome sequence of Mycobacterium tuberculosis H37Rv.* Microbiology, 2002. **148**(Pt 10): p. 2967-73.
