# Supplementary material for: Pattern Recognition in Pulmonary Tuberculosis Defined by High Content Peptide Microarray Chip Analysis Representing 61 Proteins from M. tuberculosis
Source: PLoS One. 2008 Dec 9;3(12):e3840. doi: 10.1371/journal.pone.0003840 (PMC2588537; doi:10.1371/journal.pone.0003840)
Supplement: Table S2 — 89 peptides predictive of TB+ (n = 34) vs TB− (n = 35) by PAM analysis (0.02 MB PDF) [file pone.0003840.s002.pdf]

Supplementary Table 2. 89 peptides predictive of TB+ (n=34) vs TB- (n=35) by PAM analysis

Peptide ID	Protein	Healthy-score	TB - score
VLLESMKMEIPVLAE	CAB08316.1; BIOTINYLATED PROTEIN TB7.3	-0,2588	0,2664
IRGSVTPAVSQFNAR	CAB06500.1; IMMUNOGENIC PROTEIN MPT63	-0,2535	0,2609
WGPSSDPAWERNDPT	CAB10044.1; SECRETED ANTIGEN 85-B FBPB (85B)	-0,2346	0,2415
AMEDLVRAYHAMSST	CAA17363.1; LOW MOLECULAR WEIGHT PROTEIN ANTIGEN 7 ESXH	-0,2123	0,2186
GFSEIMRSTLEKDNT	RNA POLYMERASE BETA-SUBUNIT AAA21416	-0,1427	0,1469
EGWGKSPGFGTTVDF	PERIPLASMIC PHOSPHATE-BINDING LIPOPROTEIN PSTS1 YP_177770	-0,1226	0,1262
ACGGGTNSSSSGAGG	PERIPLASMIC PHOSPHATE-BINDING LIPOPROTEIN PSTS2 YP_177769	-0,1217	0,1252
GDLPTIGTAVSARNT	CAE55335.1; PE FAMILY PROTEIN	-0,1073	0,1104
FEETFEVTAAAPVAV	CAB07109.1; PROBABLE 50S RIBOSOMAL PROTEIN L7/L12 RPLL	-0,0993	0,1022
QDPEGWGKSPGFGTT	PERIPLASMIC PHOSPHATE-BINDING LIPOPROTEIN PSTS1 YP_177770	-0,0917	0,0944
NGEEYLILSARDVLA	CAB01005.1; 10 KDA CHAPERONIN GROES	-0,0905	0,0932
LGGGVGAFLNALFAG	YP_177963; PPE FAMILY PROTEIN	-0,0702	0,0723
VFGGAASCAAPIQAD	CAB10947.1; PROBABLE LIPOPROTEIN LPRJ	-0,0655	0,0675
AAAGDLVGPGCAEYA	CAA98373.1; MAJOR SECRETED IMMUNOGENIC PROTEIN	-0,0592	0,0609
TNDGVIFFFNPGELL	CAA98382.1; IMMUNOGENIC PROTEIN MPT64	-0,0568	0,0585
ENYIAQTRDKFLSAA	CAA98382.1; IMMUNOGENIC PROTEIN MPT64	-0,0526	0,0542
DEMTRVIWKLIKDML	CAA17111.1; PROBABLE ISOCITRATE DEHYDROGENASE	-0,0501	0,0516
SAAAKNTPGSITYNE	PERIPLASMIC PHOSPHATE-BINDING LIPOPROTEIN PSTS3 YP_177768	-0,0486	0,0501
GPGQEGLDQYGSIPL	PERIPLASMIC PHOSPHATE-BINDING LIPOPROTEIN PSTS2 YP_177769	-0,044	0,0453
LCKTTSNFIWGQLLL	CAB06237.1; CONSERVED HYPOTHETICAL PROTEIN	-0,0395	0,0407
PVAGQVWEATATVNA	CAB06500.1; IMMUNOGENIC PROTEIN MPT63	-0,0385	0,0397
GVIFFFNPGELLPEA	CAA98382.1; IMMUNOGENIC PROTEIN MPT64	-0,0383	0,0394
MTLLELSDFVKKFEE	CAB07109.1; PROBABLE 50S RIBOSOMAL PROTEIN L7/L12 RPLL	-0,038	0,0391
SVPANVSRRAKVDVL	CAB08889.1; IRON-REGULATED CONSERVED HYPOTHETICAL PROTEIN	-0,0328	0,0338
GFLMPPSDGSGVTPG	MTB48 AAK31576	-0,0316	0,0325
KTLDAAIGKLLDNDK	PROBABLE ISOCITRATE DEHYDROGENASE CAA16247	-0,0284	0,0293
YEEQMQDAFETGVMF	PROBABLE ISOCITRATE DEHYDROGENASE CAA16247	-0,0251	0,0259
AEGDTVIYSKYGGTE	CAB01005.1; 10 KDA CHAPERONIN GROES	-0,0244	0,0251
LPLLEKVIGAGKPLL	60 kDa chaperonin 2 P0A521	-0,0227	0,0234
TSELPQWLSANRAVK	CAB10044.1; SECRETED ANTIGEN 85-B FBPB (85B)	-0,022	0,0227
PGGTTTLVEHMVGIL	CAA16030.1; PROBABLE MOLYBDOPTERIN-GUANINE DINUCLEOTIDE BIOSYNTHESIS PROTEIN	-0,0217	0,0223
LSEGDMAAHKGLMNI	PERIPLASMIC PHOSPHATE-BINDING LIPOPROTEIN PSTS1 YP_177770	-0,0122	0,0125
TIEQLLTIPLAKELA	MTB81	-0,0078	0,0081
AKVRRERMGHIELAA	RNA POLYMERASE BETA'-SUBUNIT AAA21417	-0,0064	0,0066
RGEHRDEHTQDAGDK	CAA17343.1; HEAT SHOCK PROTEIN HSP	-0,0052	0,0054
QVVLGWKVSDLKSST	CAB06500.1; IMMUNOGENIC PROTEIN MPT63	-0,0028	0,0029
SPHPLLTHAVEQTGR	MYCOBACTERIUM BOVIS MYCOCEROSIC ACID SYNTHASE GENE; M95808	-0,0015	0,0015
VDVFAHWPRAQGLPV	MYCOBACTERIUM BOVIS MYCOCEROSIC ACID SYNTHASE GENE; M95808	0,0059	-0,006
GDVGTSVLSWLSDHQ	MYCOBACTERIUM BOVIS MYCOCEROSIC ACID SYNTHASE GENE; M95808	0,0059	-0,0061
ATMPYVLELADHGWR	SECRETED L-ALANINE DEHYDROGENASE ALD CAA15575	0,0059	-0,0061
RGLERGLNALADAVK	60 kDa chaperonin 2 P0A521	0,007	-0,0072
NEDVIVRELTEVQGE	PROBABLE ISOCITRATE DEHYDROGENASE CAA16247	0,0147	-0,0151
VLDDLNSRMPQSRHD	MCE-FAMILY PROTEIN MCE1AYP_177701	0,0188	-0,0194
GAGSSGWWNQAHAAL	YP_177963; PPE FAMILY PROTEIN	0,0203	-0,0209
ASTGQRSRGPSKFRM	MYCOBACTERIUM BOVIS MYCOCEROSIC ACID SYNTHASE GENE; M95808	0,0227	-0,0234
VPGRSVSRWGGFLDD	MYCOBACTERIUM BOVIS MYCOCEROSIC ACID SYNTHASE GENE; M95808	0,0234	-0,024
YVIAGDIPLINIPPT	YP_177963; PPE FAMILY PROTEIN	0,0237	-0,0244
TDEQAITAATAHATA	MYCOBACTERIUM BOVIS MYCOCEROSIC ACID SYNTHASE GENE; M95808	0,0288	-0,0296
LGGGVAANLGRAASV	CAE55371.1; PPE FAMILY PROTEIN	0,0325	-0,0334
PMSTRLGIRLVIVLV	POSSIBLE GLYCOSYL TRANSFERASE CAB05419	0,0332	-0,0342
GIRLVIVLVRRWPKV	POSSIBLE GLYCOSYL TRANSFERASE CAB05419	0,0338	-0,0348
ERYPEWNEGVKGAWV	CONSERVED HYPOTHETICAL PROTEIN TB18.5CAD93033	0,0339	-0,0349
FAHWPRAQGLPVSAI	MYCOBACTERIUM BOVIS MYCOCEROSIC ACID SYNTHASE GENE; M95808	0,0368	-0,0379
GEIWVHGDNVGNGYW	MYCOBACTERIUM BOVIS ACYL-COA SYNTHASE GENE; U75685	0,0381	-0,0392
SDALRQTARQLATWV	MYCOBACTERIUM BOVIS MYCOCEROSIC ACID SYNTHASE GENE; M95808	0,0386	-0,0397
GTMKSQPWILAYEDH	MTB81	0,039	-0,0401
MLEMRTHVETETGIR	MYCOBACTERIUM BOVIS MYCOCEROSIC ACID SYNTHASE GENE; M95808	0,0392	-0,0403
DELIDRDWSPKVFGS	MYCOBACTERIUM BOVIS MYCOCEROSIC ACID SYNTHASE GENE; M95808	0,0446	-0,0459

LIATGEQVLVNEHLI	CONSERVED HYPOTHETICAL PROTEIN CAB08634	0,0452	-0,0465
VLWSPDVKPPTWWHS	POSSIBLE GLYCOSYL TRANSFERASE CAB05418	0,0549	-0,0565
SQVGTAVKAFLQSTI	PERIPLASMIC PHOSPHATE-BINDING LIPOPROTEIN PSTS3 YP_177768	0,0636	-0,0655
FGANMSGWWNQAHTA	YP_177963; PPE FAMILY PROTEIN	0,0645	-0,0663
GTAVLAGASLVAVSW	POSSIBLE HEMOLYSIN-LIKE PROTEIN CAA17201	0,0663	-0,0682
PPIVFLPSMTIGGQT	YP_177963; PPE FAMILY PROTEIN	0,0668	-0,0688
TLTDALKSHGPQGTE	MYCOBACTERIUM BOVIS MYCOCEROSIC ACID SYNTHASE GENE; M95808	0,068	-0,07
VGWANMGLGNIGFGN	YP_177963; PPE FAMILY PROTEIN	0,0755	-0,0777
RSVSRWGGFLDDVAG	MYCOBACTERIUM BOVIS MYCOCEROSIC ACID SYNTHASE GENE; M95808	0,0765	-0,0787
IDKWHRRRVIEPIDM	MTB81	0,0827	-0,0851
ANHEYLGPVLWSPAG	POSSIBLE GLYCOSYL TRANSFERASE CAB05415	0,0961	-0,0989
EGVTGHQVGDRVGGF	MYCOBACTERIUM BOVIS MYCOCEROSIC ACID SYNTHASE GENE; M95808	0,1032	-0,1062
GTPCALGSAKSNMGH	MYCOBACTERIUM BOVIS MYCOCEROSIC ACID SYNTHASE GENE; M95808	0,1115	-0,1148
LALRDQVIPPTLNLV	3-OXOACYL-[ACYL-CARRIER PROTEIN] SYNTHASE 2 KASB CAA94642	0,1121	-0,1154
SNAVDTPDMLASTLT	MYCOBACTERIUM BOVIS MYCOCEROSIC ACID SYNTHASE GENE; M95808	0,1142	-0,1175
AEHTGKLVLDVPRSG	MYCOBACTERIUM BOVIS MYCOCEROSIC ACID SYNTHASE GENE; M95808	0,1144	-0,1177
GFGNFGAGSSGWWNQ	YP_177963; PPE FAMILY PROTEIN	0,1191	-0,1226
GFGPIAVPSVTIPAL	YP_177963; PPE FAMILY PROTEIN	0,1311	-0,135
FGNFGANMSGWWNQA	YP_177963; PPE FAMILY PROTEIN	0,1314	-0,1353
APGCWQPITRDLWPA	MCE-FAMILY PROTEIN MCE1AYP_177701	0,1422	-0,1463
GNFGAGSSGWWNQAH	YP_177963; PPE FAMILY PROTEIN	0,1442	-0,1484
CAMFTDLLAQPTPAW	PUTATIVE CYCLOPROPANE-FATTY-ACYL-PHOSPHOLIPID SYNTHASE UFAA1 NP_854118	0,1475	-0,1518
IQVAIPLTSATLGGL	YP_177963; PPE FAMILY PROTEIN	0,16	-0,1647
AAAAMAAAATPYVGW	PPE FAMILY PROTEIN CAE55334	0,1614	-0,1662
NMSGWWNQAHTALAG	YP_177963; PPE FAMILY PROTEIN	0,1817	-0,1871
IPIDIPPIDIPASTI	YP_177963; PPE FAMILY PROTEIN	0,1909	-0,1966
CIECPDGTVGEIWVH	MYCOBACTERIUM BOVIS ACYL-COA SYNTHASE GENE; U75685	0,1933	-0,199
EPGVPGRSVSRWGGF	MYCOBACTERIUM BOVIS MYCOCEROSIC ACID SYNTHASE GENE; M95808	0,2218	-0,2284
KRLPMSTRLGIRLVI	POSSIBLE GLYCOSYL TRANSFERASE CAB05419	0,2232	-0,2298
TRLGIRLVIVLVRRW	POSSIBLE GLYCOSYL TRANSFERASE CAB05419	0,3283	-0,3379
WHRRRVIEPIDMDAY	MTB81	0,395	-0,4067
