# Supplementary material for: Pattern Recognition in Pulmonary Tuberculosis Defined by High Content Peptide Microarray Chip Analysis Representing 61 Proteins from M. tuberculosis
Source: PLoS One. 2008 Dec 9;3(12):e3840. doi: 10.1371/journal.pone.0003840 (PMC2588537; doi:10.1371/journal.pone.0003840)
Supplement: Table S3 — Peptides predictive of TB+ (n = 34) vs TB− (n = 35) by SAM analysis (0.08 MB PDF) [file pone.0003840.s003.pdf]

Supplementary Table 3. Peptides predictive of TB+ (n=34) vs TB- (n=35) by SAM analysis

Peptide ID	Protein	Score(d)	Fold Change
WGPSSDPAWERNDPT	CAB10044.1; SECRETED ANTIGEN 85-B FBPB (85B)	7,543056466	1,492452594
IRGSVTPAVSQFNAR	CAB06500.1; IMMUNOGENIC PROTEIN MPT63	7,226410972	1,605744998
VLLESMKMEIPVLAE	CAB08316.1; BIOTINYLATED PROTEIN TB7.3	6,395170976	2,659637684
AMEDLVRAYHAMSST	CAA17363.1; LOW MOLECULAR WEIGHT PROTEIN ANTIGEN 7 ESXH	6,359877451	1,64631432
EGWGKSPGFGTTVDF	PERIPLASMIC PHOSPHATE-BINDING LIPOPROTEIN PSTS1 YP_177770	5,952928524	1,453083374
GFSEIMRSTLEKDNT	RNA POLYMERASE BETA-SUBUNIT AAA21416	5,824780797	1,49666428
ACGGGTNSSSSGAGG	PERIPLASMIC PHOSPHATE-BINDING LIPOPROTEIN PSTS2 YP_177769	5,630151939	1,487331017
FEETFEVTAAAPVAV	CAB07109.1; PROBABLE 50S RIBOSOMAL PROTEIN L7/L12 RPLL	5,397598823	1,428631429
LGGGVGAFLNALFAG	YP_177963; PPE FAMILY PROTEIN	5,249389006	1,373591334
QDPEGWGKSPGFGTT	PERIPLASMIC PHOSPHATE-BINDING LIPOPROTEIN PSTS1 YP_177770	5,164393514	1,535252696
SAAAKNTPGSITYNE	PERIPLASMIC PHOSPHATE-BINDING LIPOPROTEIN PSTS3 YP_177768	5,073107234	1,340551209
NGEEYLILSARDVLA	CAB01005.1; 10 KDA CHAPERONIN GROES	5,046942792	1,57280672
GDLPTIGTAVSARNT	CAE55335.1; PE FAMILY PROTEIN	5,019799543	1,549150717
VFGGAASCAAPIQAD	CAB10947.1; PROBABLE LIPOPROTEIN LPRJ	4,996426873	1,431101124
GPGQEGLDQYGSIPL	PERIPLASMIC PHOSPHATE-BINDING LIPOPROTEIN PSTS2 YP_177769	4,95040986	1,343962311
ENYIAQTRDKFLSAA	CAA98382.1; IMMUNOGENIC PROTEIN MPT64	4,913217198	1,391534609
AAAGDLVGPGCAEYA	CAA98373.1; MAJOR SECRETED IMMUNOGENIC PROTEIN	4,838836065	1,424093299
PVAGQVWEATATVNA	CAB06500.1; IMMUNOGENIC PROTEIN MPT63	4,805006274	1,379400624
LSEGDMAAHKGLMNI	PERIPLASMIC PHOSPHATE-BINDING LIPOPROTEIN PSTS1 YP_177770	4,787581389	1,291054396
GFLMPPSDGSGVTPG	MTB48 AAK31576	4,612601363	1,360779775
DEMTRVIWKLIKDML	CAA17111.1; PROBABLE ISOCITRATE DEHYDROGENASE	4,557135764	1,493835585
KTLDAAIGKLLDNDK	PROBABLE ISOCITRATE DEHYDROGENASE CAA16247	4,543138388	1,43383417
MTLLELSDFVKKFEE	CAB07109.1; PROBABLE 50S RIBOSOMAL PROTEIN L7/L12 RPLL	4,529431708	1,480964789
SVPANVSRRAKVDVL	CAB08889.1; IRON-REGULATED CONSERVED HYPOTHETICAL PROTEIN	4,525562693	1,37587643
AEGDTVIYSKYGGTE	CAB01005.1; 10 KDA CHAPERONIN GROES	4,506372376	1,39241218
AKVRRERMGHIELAA	RNA POLYMERASE BETA'-SUBUNIT AAA21417	4,504619992	1,317442718
TSELPQWLSANRAVK	CAB10044.1; SECRETED ANTIGEN 85-B FBPB (85B)	4,419395015	1,37466625
CGGVSTANATVYMID	CAA98373.1; MAJOR SECRETED IMMUNOGENIC PROTEIN	4,382030526	1,289270173
QVVLGWKVSDLKSST	CAB06500.1; IMMUNOGENIC PROTEIN MPT63	4,36325164	1,324089833
RKVVVTKDETTIVEG	60 kDa chaperonin 2 P0A521	4,346190145	1,249218162
TGGGGGSGFSNSGSG	YP_177963; PPE FAMILY PROTEIN	4,340725478	1,277469926
GDDDRLHGMLTDRDI	HYPOTHETICAL PROTEIN RV2626C NP_217142	4,321666353	1,30931065
HRSDGSGDTFLFTQY	PERIPLASMIC PHOSPHATE-BINDING LIPOPROTEIN PSTS1 YP_177770	4,295379775	1,264835122
LEAAGDKKIGVIKVV	CAB07109.1; PROBABLE 50S RIBOSOMAL PROTEIN L7/L12 RPLL	4,282202095	1,281240425
RGEHRDEHTQDAGDK	CAA17343.1; HEAT SHOCK PROTEIN HSP	4,278446467	1,327223015
WISEYKEVEILEADD	CONSERVED HYPOTHETICAL PROTEIN TB16.3CAD97060	4,276759181	1,308237261
PGGTTTLVEHMVGIL	CAA16030.1; PROBABLE MOLYBDOPTERIN-GUANINE DINUCLEOTIDE BIOSYNTHESIS PROTEIN	4,26813859	1,355496021
TLQAFLHWAITDGNK	PERIPLASMIC PHOSPHATE-BINDING LIPOPROTEIN PSTS1 YP_177770	4,260838908	1,31242242
YEEQMQDAFETGVMF	PROBABLE ISOCITRATE DEHYDROGENASE CAA16247	4,221681026	1,702239193
AMMARDTAEAAKWGG	CAA17363.1; LOW MOLECULAR WEIGHT PROTEIN ANTIGEN 7 ESXH	4,199424311	1,27612978
RGLRAAAEAGVRLAF	CAA16030.1; PROBABLE MOLYBDOPTERIN-GUANINE DINUCLEOTIDE BIOSYNTHESIS PROTEIN	4,186145299	1,292019249
TNDGVIFFFNPGELL	CAA98382.1; IMMUNOGENIC PROTEIN MPT64	4,166409029	2,874073623
GVIFFFNPGELLPEA	CAA98382.1; IMMUNOGENIC PROTEIN MPT64	4,141959035	1,847326701
LPGVSEHLKLNGKVL	PERIPLASMIC PHOSPHATE-BINDING LIPOPROTEIN PSTS1 YP_177770	4,133968498	1,32288704
TIEQLLTIPLAKELA	MTB81	4,129069409	1,452230375
LCKTTSNFIWGQLLL	CAB06237.1; CONSERVED HYPOTHETICAL PROTEIN	4,128641073	2,84585048
SPHPLLTHAVEQTGR	MYCOBACTERIUM BOVIS MYCOCEROSIC ACID SYNTHASE GENE; M95808	4,053212382	1,36357465
IADIEAYPQWISEYK	CONSERVED HYPOTHETICAL PROTEIN TB16.3CAD97060	4,049527406	1,57286585
SKVSTVKDLLPLLEK	60 kDa chaperonin 2 P0A521	4,020404419	1,301176314
LFSRVEDVLGLPQNT	MTB81	4,020399162	1,251941384
MWELYLAYSEAGFRS	PUTATIVE CYCLOPROPANE-FATTY-ACYL-PHOSPHOLIPID SYNTHASE UFAA1 NP_854118	4,014670768	1,301045967
AAHKGLMNIALAISA	PERIPLASMIC PHOSPHATE-BINDING LIPOPROTEIN PSTS1 YP_177770	4,011051567	1,292531068
DTLNGGEYTVFAPTN	CAA98350.1; CELL SURFACE LIPOPROTEIN MPT83	3,98438077	1,306070988
YDREKLQERLAKLAG	60 kDa chaperonin 2 P0A521	3,968710251	1,355865331
VRERMTTQDVEAITP	RNA POLYMERASE BETA-SUBUNIT AAA21416	3,92827977	1,35216364
GDLVGPGCAEYAAAN	CAA98373.1; MAJOR SECRETED IMMUNOGENIC PROTEIN	3,913998348	1,346280476
ANRAVKPTGSAAIGL	CAB10044.1; SECRETED ANTIGEN 85-B FBPB (85B)	3,908915102	1,254393918
MSFVTIQPVVLAAAT	CAE55335.1; PE FAMILY PROTEIN	3,847796095	1,323958723

LPLLEKVIGAGKPLL	60 kDa chaperonin 2 P0A521	3,799506967	5,966619148
EDEMKEGRYEVRAEL	CAA17245.1; HEAT SHOCK PROTEIN HSPX	3,794948053	1,233490479
PPGYDITALLQAHPH	MYCOBACTERIUM BOVIS MYCOCEROSIC ACID SYNTHASE GENE; M95808	3,770023725	1,258515759
VGPGRWDEDGEKRIP	CAB01005.1; 10 KDA CHAPERONIN GROES	3,73008544	1,219942489
LGWKVSDLKSSTAVI	CAB06500.1; IMMUNOGENIC PROTEIN MPT63	3,728183404	1,691886699
AAASLAAIAIAFLAG	CAA98350.1; CELL SURFACE LIPOPROTEIN MPT83	3,726944335	1,218451245
AGTVNIGASDAYLSE	PERIPLASMIC PHOSPHATE-BINDING LIPOPROTEIN PSTS1 YP_177770	3,718066062	1,261432497
QGQWRGAAGTAAQAA	CAA17966.1; 10 KDA CULTURE FILTRATE ANTIGEN ESXB	3,714141845	1,266013899
VGQVTATMGQLQQLV	CAB06237.1; CONSERVED HYPOTHETICAL PROTEIN	3,69540828	1,239482592
AGGYKAADMWGPSSD	CAB10044.1; SECRETED ANTIGEN 85-B FBPB (85B)	3,685083925	1,22459453
PGQGAYAAANSWVDV	MYCOBACTERIUM BOVIS MYCOCEROSIC ACID SYNTHASE GENE; M95808	3,678353448	1,311166881
QVGSQFGPYQLLRLL	TRANSMEMBRANE SERINE/THREONINE-PROTEIN KINASE D PKND NP_215446	3,648920557	1,972064128
RIFYKDAFAKHQELF	PROBABLE ISOCITRATE DEHYDROGENASE CAA16247	3,641917277	2,174705555
TCELFSRVEDVLGLP	MTB81	3,635983241	1,244140777
DVGSLNGTYVNREPV	CAB01474.1; CONSERVED HYPOTHETICAL PROTEIN CFP17	3,635236196	1,330963304
SEFDVILEAAGDKKI	CAB07109.1; PROBABLE 50S RIBOSOMAL PROTEIN L7/L12 RPLL	3,629166901	1,763418706
LGENGNGGMVTGCAE	PERIPLASMIC PHOSPHATE-BINDING LIPOPROTEIN PSTS1 YP_177770	3,595085324	1,198416921
TSEQIVERFGFSEIM	RNA POLYMERASE BETA-SUBUNIT AAA21416	3,593269783	1,441432449
EAEHQAIVRDVLAAG	PUTATIVE ESAT-6 LIKE PROTEIN ESXN YP_177838	3,580808637	1,260995712
YIGISFLDQASQRGL	PERIPLASMIC PHOSPHATE-BINDING LIPOPROTEIN PSTS1 YP_177770	3,553659689	1,239501957
RGLGEAQLGNSSGNF	PERIPLASMIC PHOSPHATE-BINDING LIPOPROTEIN PSTS1 YP_177770	3,544484337	1,229393291
LSIVMPVGGQSSFYS	CAB10044.1; SECRETED ANTIGEN 85-B FBPB (85B)	3,543904042	1,279160099
VALFGEPSSGFSSML	PROBABLE CUTINASE PRECURSOR CFP21 NP_216500	3,533941308	2,016206769
TWQTKSGGTRTGNVT	CAB09453.1; PROBABLE SERINE PROTEASE PEPA	3,525451301	1,244619216
TGNMISNQAKYVSDT	CAB06237.1; CONSERVED HYPOTHETICAL PROTEIN	3,516965266	1,285557713
LASTLTDALKSHGPQ	MYCOBACTERIUM BOVIS MYCOCEROSIC ACID SYNTHASE GENE; M95808	3,512910002	1,219803845
SAAIGLSMAGSSAMI	CAB10044.1; SECRETED ANTIGEN 85-B FBPB (85B)	3,5118702	1,202126625
ESGTTDNFQRYLQAA	PERIPLASMIC PHOSPHATE-BINDING LIPOPROTEIN PSTS3 YP_177768	3,508094545	1,236890001
HVIAGQASPSRIDGT	CAA98350.1; CELL SURFACE LIPOPROTEIN MPT83	3,487395491	1,219266395
CVAYIGISFLDQASQ	PERIPLASMIC PHOSPHATE-BINDING LIPOPROTEIN PSTS1 YP_177770	3,455200032	1,237933076
PETINYRTLKPEKDG	RNA POLYMERASE BETA'-SUBUNIT AAA21417	3,43476377	1,218566129
FPSFAGLRPTFDTRL	CAA17245.1; HEAT SHOCK PROTEIN HSPX	3,414098855	3,988954005
VSVAVSEGKPTEKHI	CAA17245.1; HEAT SHOCK PROTEIN HSPX	3,39206489	1,22861781
NTPAIAVNEAEYGEM	CAE55371.1; PPE FAMILY PROTEIN	3,37521295	1,205161367
HQGQGGAKSKGSQQE	MTB48 AAK31576	3,370006917	1,270127225
PDRSAERCGSPAWDL	PERIPLASMIC PHOSPHATE-BINDING LIPOPROTEIN PSTS2 YP_177769	3,357336369	1,306068486
EKVAKEAADEAKAKL	CAB07109.1; PROBABLE 50S RIBOSOMAL PROTEIN L7/L12 RPLL	3,355732495	1,293919875
PLDTPPAPAPPPFRL	YP_177963; PPE FAMILY PROTEIN	3,340030267	1,538360375
QAQLISSQAQQGGQQ	CAB06237.1; CONSERVED HYPOTHETICAL PROTEIN	3,339075582	1,236887404
LQAHPHAVNGTAMRE	MYCOBACTERIUM BOVIS MYCOCEROSIC ACID SYNTHASE GENE; M95808	3,337000624	1,357036006
PGYPVAGQVWEATAT	CAB06500.1; IMMUNOGENIC PROTEIN MPT63	3,32944389	1,225800503
MWLSPNGTIRNILGG	CAA17111.1; PROBABLE ISOCITRATE DEHYDROGENASE	3,327732928	1,362672629
LAPDTVPLAGVVLAG	CAA16030.1; PROBABLE MOLYBDOPTERIN-GUANINE DINUCLEOTIDE BIOSYNTHESIS PROTEIN	3,30935192	1,257127895
LGDVGEAFVDSLTSQ	PROBABLE CUTINASE PRECURSOR CFP21 NP_216500	3,307123473	1,596342397
IGLSMAGSSAMILAA	CAB10044.1; SECRETED ANTIGEN 85-B FBPB (85B)	3,301643613	1,201574684
LPADTAAQLTSAGRE	MTB48 AAK31576	3,290558179	1,273146998
TAQTLQAFLHWAITD	PERIPLASMIC PHOSPHATE-BINDING LIPOPROTEIN PSTS1 YP_177770	3,267335637	1,258025181
FGLQLELTEGMRFDK	60 kDa chaperonin 2 P0A521	3,258657142	1,238047797
LMILIATNLLGQNTP	CAE55371.1; PPE FAMILY PROTEIN	3,234562697	1,308607794
DGYPIINYEYAIVNN	PERIPLASMIC PHOSPHATE-BINDING LIPOPROTEIN PSTS1 YP_177770	3,205355936	1,182148257
FLGGFAGLPSLGFGN	CAB06237.1; CONSERVED HYPOTHETICAL PROTEIN	3,203788718	1,24289223
ADEEQQQALSSQMGF	CAA17966.1; 10 KDA CULTURE FILTRATE ANTIGEN ESXB	3,201730961	1,216068513
WLSANRAVKPTGSAA	CAB10044.1; SECRETED ANTIGEN 85-B FBPB (85B)	3,188504025	1,201013879
ITSAGRHPDSDIFLD	CAB01474.1; CONSERVED HYPOTHETICAL PROTEIN CFP17	3,178927462	1,205186836
VTVTGQGNSLKVGNA	CAA98373.1; MAJOR SECRETED IMMUNOGENIC PROTEIN	3,175083854	1,220301187
AFLHWAITDGNKASF	PERIPLASMIC PHOSPHATE-BINDING LIPOPROTEIN PSTS1 YP_177770	3,172954376	1,231614409
VLCNGGSPTTQQALA	POSSIBLE GLYCOSYL TRANSFERASE CAB05418	3,161437936	1,167273448
ASFLDQVHFQPLPPA	PERIPLASMIC PHOSPHATE-BINDING LIPOPROTEIN PSTS1 YP_177770	3,157875045	1,2905779
LLDIYRKLRPGEPPT	RNA POLYMERASE BETA-SUBUNIT AAA21416	3,148263079	1,327553186
QNAGGTHPTTTYKAF	CAA98382.1; IMMUNOGENIC PROTEIN MPT64	3,128768113	2,131662258
AQAQHLTMANIVTSA	PERIPLASMIC PHOSPHATE-BINDING LIPOPROTEIN PSTS3 YP_177768	3,125150591	1,195761817
LEPGVVAEKVRNLPA	60 kDa chaperonin 2 P0A521	3,111776807	1,229392988
GVPSRLGYLLDLAPK	RNA POLYMERASE BETA'-SUBUNIT AAA21417	3,110843745	2,243846408
FPHHPIHTVPSEEVL	POSSIBLE GLYCOSYL TRANSFERASE CAB05418	3,110761094	1,413178556

IAFLAGCSSTKPVSQ	CAA98350.1; CELL SURFACE LIPOPROTEIN MPT83	3,106179311	1,235403909
PPTPISVIFRSDKSG	PERIPLASMIC PHOSPHATE-BINDING LIPOPROTEIN PSTS2 YP_177769	3,097047657	1,187984157
QSGGNNSPAVYLLDG	CAB10044.1; SECRETED ANTIGEN 85-B FBPB (85B)	3,093192001	1,178910156
QSGLGDNGYIPIPDE	PERIPLASMIC PHOSPHATE-BINDING LIPOPROTEIN PSTS3 YP_177768	3,082781854	1,199339641
LTTYILLPSQDLPLL	PPE FAMILY PROTEIN CAE55504	3,076699053	2,270394617
LILTSYTSDEAMLDA	TWO COMPONENT TRANSCRIPTIONAL REGULATORY PROTEIN DEVR NP_217649	3,076065886	1,211003625
GNKASFLDQVHFQPL	PERIPLASMIC PHOSPHATE-BINDING LIPOPROTEIN PSTS1 YP_177770	3,065912657	1,272041941
SAFAAKAGLMRHTIG	CONSERVED HYPOTHETICAL PROTEIN TB9.8 CAD93159	3,053479759	1,158366214
SAERCGSPAWDLPTV	PERIPLASMIC PHOSPHATE-BINDING LIPOPROTEIN PSTS2 YP_177769	3,027222134	1,298749663
KGLMNIALAISAQQV	PERIPLASMIC PHOSPHATE-BINDING LIPOPROTEIN PSTS1 YP_177770	3,023121017	1,163210728
QSVCPILAEPGGSFN	CONSERVED HYPOTHETICAL PROTEIN CAA15739	3,018149855	1,204792503
VCAVDMPYLTVELIE	CAA16030.1; PROBABLE MOLYBDOPTERIN-GUANINE DINUCLEOTIDE BIOSYNTHESIS PROTEIN	3,016545971	1,561980797
DVVQHVARRPGESPP	MYCOBACTERIUM BOVIS ACYL-COA SYNTHASE GENE; U75685	3,015671163	1,278025275
GYPVHKPVTAGWNGY	CONSERVED HYPOTHETICAL PROTEIN CAB08634	3,012111853	1,296232943
QEAGNFERISGDLKT	CAA17966.1; 10 KDA CULTURE FILTRATE ANTIGEN ESXB	3,007125532	1,181144787
PVIAENRAELMILIA	CAE55371.1; PPE FAMILY PROTEIN	3,005612498	1,173936112
ISMIDGPAPDGYPII	PERIPLASMIC PHOSPHATE-BINDING LIPOPROTEIN PSTS1 YP_177770	3,005418763	1,221429185
TDVIPETDGAEKGPT	MTB81	3,002446553	1,349117711
YHVVAGQTSPANVVG	CAA98373.1; MAJOR SECRETED IMMUNOGENIC PROTEIN	2,998347757	1,158727608
ILKDESYKVTGTAPI	CAB08889.1; IRON-REGULATED CONSERVED HYPOTHETICAL PROTEIN	2,995338559	1,216757313
HGTCANQCPIVDAGD	RNA POLYMERASE BETA-SUBUNIT AAA21416	2,988744517	1,270881516
NAFLTALTNAGIAYD	CAB10947.1; PROBABLE LIPOPROTEIN LPRJ	2,985448864	1,235463484
DSIYYVDANASIQEM	HYPOTHETICAL PROTEIN RV2626C NP_217142	2,978585453	1,186318964
AAQAVQTAAQNGVRA	CAE55371.1; PPE FAMILY PROTEIN	2,968766547	1,226101594
PAEFLENFVRSSNLK	CAB10044.1; SECRETED ANTIGEN 85-B FBPB (85B)	2,963694769	1,208556386
TPPAPAPPPFRLPLL	YP_177963; PPE FAMILY PROTEIN	2,962157075	5,003206502
KTINLCAPDDPICTG	PROBABLE CUTINASE PRECURSOR CFP21 NP_216500	2,954247002	1,175523886
YNANGSGAGVTQFLN	PERIPLASMIC PHOSPHATE-BINDING LIPOPROTEIN PSTS2 YP_177769	2,952168417	1,160137969
RSCPGYTLDYNANGS	PERIPLASMIC PHOSPHATE-BINDING LIPOPROTEIN PSTS2 YP_177769	2,950124484	1,184885013
AGAGTVATTPASSPV	PERIPLASMIC PHOSPHATE-BINDING LIPOPROTEIN PSTS1 YP_177770	2,947458296	1,219207153
IWKLIKDMLILPYLD	CAA17111.1; PROBABLE ISOCITRATE DEHYDROGENASE	2,939266202	1,26928518
AYVITSVDEEMRHNE	RNA POLYMERASE BETA'-SUBUNIT AAA21417	2,931773398	1,211631869
DRFADFPALPLDPSA	CAB09453.1; PROBABLE SERINE PROTEASE PEPA	2,928754666	1,182858938
VGNVGLSTIAGRLLG	CAB08889.1; IRON-REGULATED CONSERVED HYPOTHETICAL PROTEIN	2,928257998	1,219082627
FGDVDAHGAMIRAQA	PUTATIVE ESAT-6 LIKE PROTEIN ESXN YP_177838	2,923999807	1,185013956
VHNVAALPGAAYCEM	MYCOBACTERIUM BOVIS MYCOCEROSIC ACID SYNTHASE GENE; M95808	2,923964425	1,154112332
TAVVLLCCSGVATAA	CAA98382.1; IMMUNOGENIC PROTEIN MPT64	2,920743115	1,206995716
LAALPDFLGGFAGLP	CAB06237.1; CONSERVED HYPOTHETICAL PROTEIN	2,917871383	1,288715308
TVIAATAGRNHLKNV	POSSIBLE GLYCOSYL TRANSFERASE CAB05418	2,912023904	1,192106149
IVMSTNNDDPAGACR	3-OXOACYL-[ACYL-CARRIER PROTEIN] SYNTHASE 2 KASB CAA94642	2,89912101	1,20047952
DPRYNQLLGPLPFRH	POSSIBLE GLYCOSYL TRANSFERASE CAB05415	2,883700431	1,179960428
MNIALAISAQQVNYN	PERIPLASMIC PHOSPHATE-BINDING LIPOPROTEIN PSTS1 YP_177770	2,879796278	1,177018598
WSFAVGKQLNMAQII	PERIPLASMIC PHOSPHATE-BINDING LIPOPROTEIN PSTS2 YP_177769	2,879236187	1,196378184
YTFKEDEYPSTAYLQ	MYCOBACTERIUM BOVIS ACYL-COA SYNTHASE GENE; U75685	2,879175615	1,299438787
GASVTVTGQGNSLKV	CAA98373.1; MAJOR SECRETED IMMUNOGENIC PROTEIN	2,879140981	1,163393009
ATIPQGEQSTGKIYF	CAB06500.1; IMMUNOGENIC PROTEIN MPT63	2,874760041	1,191895829
TAALRAAEDDCPPPG	MYCOBACTERIUM BOVIS MYCOCEROSIC ACID SYNTHASE GENE; M95808	2,871508551	1,146331184
AGFTAPATTLLSALG	YP_177963; PPE FAMILY PROTEIN	2,863206106	1,469672565
MAEDVRAEIVASVLE	CAB08316.1; BIOTINYLATED PROTEIN TB7.3	2,862835875	1,235696419
ALTGLGDKFGESIVN	MCE-FAMILY PROTEIN MCE1AYP_177701	2,860193555	1,300904653
WHRRRVIEPIDMDAY	MTB81 (publication)	-7,977690023	0,393926473
TRLGIRLVIVLVRRW	POSSIBLE GLYCOSYL TRANSFERASE CAB05419	-7,878118104	0,522570785
IPIDIPPIDIPASTI	YP_177963; PPE FAMILY PROTEIN	-6,85023435	0,686345944
KRLPMSTRLGIRLVI	POSSIBLE GLYCOSYL TRANSFERASE CAB05419	-6,741303355	0,574535454
EPGVPGRSVSRWGGF	MYCOBACTERIUM BOVIS MYCOCEROSIC ACID SYNTHASE GENE; M95808	-6,697487634	0,580394207
IQVAIPLTSATLGGL	YP_177963; PPE FAMILY PROTEIN	-6,581101808	0,707024085
CIECPDGTVGEIWVH	MYCOBACTERIUM BOVIS ACYL-COA SYNTHASE GENE; U75685	-6,358414308	0,583806535
LALRDQVIPPTLNLV	3-OXOACYL-[ACYL-CARRIER PROTEIN] SYNTHASE 2 KASB CAA94642	-6,174626856	0,732640634
NMSGWWNQAHTALAG	YP_177963; PPE FAMILY PROTEIN	-6,009783795	0,560224326
AAAAMAAAATPYVGW	PPE FAMILY PROTEIN CAE55334	-5,899224666	0,572044128
GFGPIAVPSVTIPAL	YP_177963; PPE FAMILY PROTEIN	-5,872792378	0,697076072
PPIVFLPSMTIGGQT	YP_177963; PPE FAMILY PROTEIN	-5,844150874	0,773709564
GNFGAGSSGWWNQAH	YP_177963; PPE FAMILY PROTEIN	-5,701177999	0,625028336
SQVGTAVKAFLQSTI	PERIPLASMIC PHOSPHATE-BINDING LIPOPROTEIN PSTS3 YP_177768	-5,588282549	0,75128722

VGWANMGLGNIGFGN	YP_177963; PPE FAMILY PROTEIN	-5,47613507	0,722184313
APGCWQPITRDLWPA	MCE-FAMILY PROTEIN MCE1AYP_177701	-5,47423812	0,58374356
CAMFTDLLAQPTPAW	PUTATIVE CYCLOPROPANE-FATTY-ACYL-PHOSPHOLIPID SYNTHASE UFAA1 NP_854118	-5,425741858	0,529908514
AEHTGKLVLDVPRSG	MYCOBACTERIUM BOVIS MYCOCEROSIC ACID SYNTHASE GENE; M95808	-5,306983761	0,634503903
FGNFGANMSGWWNQA	YP_177963; PPE FAMILY PROTEIN	-5,23042175	0,590999338
EGVTGHQVGDRVGGF	MYCOBACTERIUM BOVIS MYCOCEROSIC ACID SYNTHASE GENE; M95808	-5,207458819	0,637440969
GFGNFGAGSSGWWNQ	YP_177963; PPE FAMILY PROTEIN	-5,165235502	0,53674499
FAHWPRAQGLPVSAI	MYCOBACTERIUM BOVIS MYCOCEROSIC ACID SYNTHASE GENE; M95808	-5,127146231	0,752019111
GTPCALGSAKSNMGH	MYCOBACTERIUM BOVIS MYCOCEROSIC ACID SYNTHASE GENE; M95808	-5,12271031	0,545474831
SNAVDTPDMLASTLT	MYCOBACTERIUM BOVIS MYCOCEROSIC ACID SYNTHASE GENE; M95808	-5,105028429	0,524860921
IDKWHRRRVIEPIDM	MTB81 (publication)	-5,062134837	0,628037561
NEDVIVRELTEVQGE	PROBABLE ISOCITRATE DEHYDROGENASE CAA16247	-5,031957593	0,785590376
RSVSRWGGFLDDVAG	MYCOBACTERIUM BOVIS MYCOCEROSIC ACID SYNTHASE GENE; M95808	-4,989951623	0,646631039
SDALRQTARQLATWV	MYCOBACTERIUM BOVIS MYCOCEROSIC ACID SYNTHASE GENE; M95808	-4,982332949	0,732383702
LGGGVAANLGRAASV	CAE55371.1; PPE FAMILY PROTEIN	-4,977263706	0,752498409
YVIAGDIPLINIPPT	YP_177963; PPE FAMILY PROTEIN	-4,972580266	0,763786885
ANHEYLGPVLWSPAG	POSSIBLE GLYCOSYL TRANSFERASE CAB05415	-4,922874027	0,587678958
VDVFAHWPRAQGLPV	MYCOBACTERIUM BOVIS MYCOCEROSIC ACID SYNTHASE GENE; M95808	-4,89373758	0,78158946
MLEMRTHVETETGIR	MYCOBACTERIUM BOVIS MYCOCEROSIC ACID SYNTHASE GENE; M95808	-4,889360013	0,735019525
ISIPNNPLALLASAS	YP_177963; PPE FAMILY PROTEIN	-4,889140073	0,802321204
DELIDRDWSPKVFGS	MYCOBACTERIUM BOVIS MYCOCEROSIC ACID SYNTHASE GENE; M95808	-4,879828288	0,709308043
GTAVLAGASLVAVSW	POSSIBLE HEMOLYSIN-LIKE PROTEIN CAA17201	-4,866213262	0,65133847
LIATGEQVLVNEHLI	CONSERVED HYPOTHETICAL PROTEIN CAB08634	-4,726528798	0,66499837
PMSTRLGIRLVIVLV	POSSIBLE GLYCOSYL TRANSFERASE CAB05419	-4,685977343	0,690026531
GIRLVIVLVRRWPKV	POSSIBLE GLYCOSYL TRANSFERASE CAB05419	-4,641480239	0,684143195
VLWSPDVKPPTWWHS	POSSIBLE GLYCOSYL TRANSFERASE CAB05418	-4,638316745	0,659365874
TLTDALKSHGPQGTE	MYCOBACTERIUM BOVIS MYCOCEROSIC ACID SYNTHASE GENE; M95808	-4,60526696	0,587346652
LTNATMPYVLELADH	SECRETED L-ALANINE DEHYDROGENASE ALD CAA15575	-4,559720896	0,75694855
TRQAQIVKPHDSGER	MYCOBACTERIUM BOVIS MYCOCEROSIC ACID SYNTHASE GENE; M95808	-4,5398754	0,816310403
KLTADGVLTAPQCTH	MYCOBACTERIUM BOVIS MYCOCEROSIC ACID SYNTHASE GENE; M95808	-4,534385515	0,78368222
TDEQAITAATAHATA	MYCOBACTERIUM BOVIS MYCOCEROSIC ACID SYNTHASE GENE; M95808	-4,51808827	0,664164467
GAGSSGWWNQAHAAL	YP_177963; PPE FAMILY PROTEIN	-4,48663606	0,703376824
ASTGQRSRGPSKFRM	MYCOBACTERIUM BOVIS MYCOCEROSIC ACID SYNTHASE GENE; M95808	-4,48614906	0,744503526
FGANMSGWWNQAHTA	YP_177963; PPE FAMILY PROTEIN	-4,467565814	0,595440925
VPGRSVSRWGGFLDD	MYCOBACTERIUM BOVIS MYCOCEROSIC ACID SYNTHASE GENE; M95808	-4,460064573	0,689737768
GEIWVHGDNVGNGYW	MYCOBACTERIUM BOVIS ACYL-COA SYNTHASE GENE; U75685	-4,457694816	0,658389207
QIDIPALNPNVTGSV	YP_177963; PPE FAMILY PROTEIN	-4,454495314	0,786259657
GTMKSQPWILAYEDH	MTB81 (publication)	-4,410186348	0,592277135
RGLERGLNALADAVK	60 kDa chaperonin 2 P0A521	-4,333916983	0,748260341
SFNTGIGNGGTGSTG	YP_177963; PPE FAMILY PROTEIN	-4,332885809	0,815750498
VLDDLNSRMPQSRHD	MCE-FAMILY PROTEIN MCE1AYP_177701	-4,277221669	0,689118528
ERYPEWNEGVKGAWV	CONSERVED HYPOTHETICAL PROTEIN TB18.5CAD93033	-4,269413392	0,60265187
DEVFARMWELYLAYS	PUTATIVE CYCLOPROPANE-FATTY-ACYL-PHOSPHOLIPID SYNTHASE UFAA1 NP_854118	-4,258584888	0,726920542
TPIGDPIEYRSLARV	MYCOBACTERIUM BOVIS MYCOCEROSIC ACID SYNTHASE GENE; M95808	-4,250415609	0,745217404
YVAADAAAASTYTGF	CONSERVED HYPOTHETICAL PROTEIN TB9.8 CAD93159	-4,217274253	0,687545434
ISYMLPRSPIVRIVD	MYCOBACTERIUM BOVIS ACYL-COA SYNTHASE GENE; U75685	-4,206825327	0,753331902
INSVLMYSGAGSSPL	YP_177963; PPE FAMILY PROTEIN	-4,18717109	0,766572955
LSGSEEDETAWRNGD	MYCOBACTERIUM BOVIS MYCOCEROSIC ACID SYNTHASE GENE; M95808	-4,17239579	0,763738891
DLPANHEYLGPVLWS	POSSIBLE GLYCOSYL TRANSFERASE CAB05415	-4,161997667	0,732910409
VGTPGATIPAITFPE	YP_177963; PPE FAMILY PROTEIN	-4,160236437	0,799829856
LTIPGLSLDAAVSEI	YP_177963; PPE FAMILY PROTEIN	-4,155198128	0,82945517
QASSPMVGTLSGLGN	YP_177963; PPE FAMILY PROTEIN	-4,124039724	0,738438182
AANLGDLNLGLGNIG	YP_177963; PPE FAMILY PROTEIN	-4,119920787	0,824807034
GDVGTSVLSWLSDHQ	MYCOBACTERIUM BOVIS MYCOCEROSIC ACID SYNTHASE GENE; M95808	-4,118100841	0,681292571
ATMPYVLELADHGWR	SECRETED L-ALANINE DEHYDROGENASE ALD CAA15575	-4,113085135	0,653979296
EQVLAERLAGWQERY	HYPOTHETICAL PROTEIN RV2623 NP_217139	-4,106338391	0,70789415
AVRWPDPWPTTFGYH	POSSIBLE HEMOLYSIN-LIKE PROTEIN CAA17201	-4,061143621	0,712233687
EYVVAFLGALQAGRI	MYCOBACTERIUM BOVIS ACYL-COA SYNTHASE GENE; U75685	-4,037209623	0,798570489
EALPFAAIAVGAALV	CONSERVED HYPOTHETICAL PROTEIN CAB08634	-4,009837033	0,760790007
DRIPVELHASTTIGP	YP_177963; PPE FAMILY PROTEIN	-3,98171913	0,733567849
AGPINISIIDIPALP	YP_177963; PPE FAMILY PROTEIN	-3,953655312	0,793180911
LDAVERREATWRDVR	CONSERVED HYPOTHETICAL PROTEIN CAB08634	-3,94501151	0,745610463
SLPQDEWAGRLRRLL	MYCOBACTERIUM BOVIS MYCOCEROSIC ACID SYNTHASE GENE; M95808	-3,941861887	0,820437085
WPRAQGLPVSAIAWG	MYCOBACTERIUM BOVIS MYCOCEROSIC ACID SYNTHASE GENE; M95808	-3,932855663	0,789734482

PVLAINAHLTGNAAM	YP_177963; PPE FAMILY PROTEIN	-3,924762633	0,746625593
APPPIAHSTVLVAPV	PPE FAMILY PROTEIN CAE55489	-3,889123749	0,82262788
ANMGLGNIGFGNTGT	YP_177963; PPE FAMILY PROTEIN	-3,887586504	0,836958406
VVADKPEKEKASVPG	60 kDa chaperonin 2 P0A521	-3,883446387	0,809213459
SRWGGFLDDVAGFDA	MYCOBACTERIUM BOVIS MYCOCEROSIC ACID SYNTHASE GENE; M95808	-3,87959803	0,772067915
CWAMQAYGAAYTLQE	RNA POLYMERASE BETA-SUBUNIT AAA21416	-3,860537931	0,758720787
RRVIEPIDMDAYRQF	MTB81 (publication)	-3,855791074	0,679812234
AISDPLLGSASGFAN	YP_177963; PPE FAMILY PROTEIN	-3,849649955	0,763441076
PQSTVIGGTSDTVRD	MYCOBACTERIUM BOVIS MYCOCEROSIC ACID SYNTHASE GENE; M95808	-3,824229017	0,831646335
SRGPSKFRMELLSLP	MYCOBACTERIUM BOVIS MYCOCEROSIC ACID SYNTHASE GENE; M95808	-3,823956285	0,758113449
PEVATWLEVPLPPGV	HYPOTHETICAL PROTEIN RV2623 NP_217139	-3,806931281	0,765064016
RFGEMECWAMQAYGA	RNA POLYMERASE BETA-SUBUNIT AAA21416	-3,805095458	0,677584646
YIVKPKMHGPAEVAF	MTB81 (publication)	-3,804251642	0,696314142
EFPIHWTSTGGIGPI	YP_177963; PPE FAMILY PROTEIN	-3,790421089	0,750093863
YTPFALLALPAHDGR	POSSIBLE HEMOLYSIN-LIKE PROTEIN CAA17201	-3,781084082	0,730068036
FRIEPIDVPAVVLDI	YP_177963; PPE FAMILY PROTEIN	-3,769208893	0,785093984
AIPIGLELNGGVGPI	YP_177963; PPE FAMILY PROTEIN	-3,768761582	0,796780928
SGLYNTAIVGLGTPA	YP_177963; PPE FAMILY PROTEIN	-3,763699999	0,615548474
RTIDADRSFIEYGLD	MYCOBACTERIUM BOVIS MYCOCEROSIC ACID SYNTHASE GENE; M95808	-3,761252796	0,770145603
PVTAGWNGYGDFQHT	CONSERVED HYPOTHETICAL PROTEIN CAB08634	-3,735927147	0,716207526
AGMGNIGLGNAGSGN	YP_177963; PPE FAMILY PROTEIN	-3,735741874	0,823867314
LNLGAANIGDVNLGF	YP_177963; PPE FAMILY PROTEIN	-3,723509207	0,826511729
LPVSAIAWGAWGEVG	MYCOBACTERIUM BOVIS MYCOCEROSIC ACID SYNTHASE GENE; M95808	-3,721255739	0,788442057
HDTLDAVERREATWR	CONSERVED HYPOTHETICAL PROTEIN CAB08634	-3,698316163	0,577199554
GPVLWSPAGKPPTWW	POSSIBLE GLYCOSYL TRANSFERASE CAB05415	-3,688163603	0,688874078
TGSIALAITNTARPW	PPE FAMILY PROTEIN CAE55334	-3,686084576	0,755231612
LVHAVSPEVATWLEV	HYPOTHETICAL PROTEIN RV2623 NP_217139	-3,682993475	0,836598483
PLTLVHAVSPEVATW	HYPOTHETICAL PROTEIN RV2623 NP_217139	-3,682478837	0,84240897
SGLGALPGIGNIGLG	YP_177963; PPE FAMILY PROTEIN	-3,674644112	0,845111103
LWSPAGKPPTWWDSL	POSSIBLE GLYCOSYL TRANSFERASE CAB05415	-3,657732882	0,699884776
LMSVTQPDRVRELLA	MYCOBACTERIUM BOVIS MYCOCEROSIC ACID SYNTHASE GENE; M95808	-3,647438973	0,680060513
LAHLGFDEVFARMWE	PUTATIVE CYCLOPROPANE-FATTY-ACYL-PHOSPHOLIPID SYNTHASE UFAA1 NP_854118	-3,629301324	0,697474586
QLLTIPLAKELAWAP	MTB81 (publication)	-3,603599133	0,772774144
ASVILRRTIDADRSF	MYCOBACTERIUM BOVIS MYCOCEROSIC ACID SYNTHASE GENE; M95808	-3,584639086	0,793443532
NLGLADVGSVNVGFG	YP_177963; PPE FAMILY PROTEIN	-3,571369491	0,845321123
TGHQVGDRVGGFSEG	MYCOBACTERIUM BOVIS MYCOCEROSIC ACID SYNTHASE GENE; M95808	-3,566179562	0,711955944
CPDGTVGEIWVHGDN	MYCOBACTERIUM BOVIS ACYL-COA SYNTHASE GENE; U75685	-3,563738637	0,645718137
PNDQAGLEKLGSQLR	MYCOBACTERIUM BOVIS MYCOCEROSIC ACID SYNTHASE GENE; M95808	-3,551896943	0,792664934
NIISGIPTLSTTGPV	YP_177963; PPE FAMILY PROTEIN	-3,539531845	0,824648824
TGKLVLDVPRSGRRS	MYCOBACTERIUM BOVIS MYCOCEROSIC ACID SYNTHASE GENE; M95808	-3,53948196	0,808785429
PYAVEYVWGRQVGDN	MCE-FAMILY PROTEIN MCE1AYP_177701	-3,531853784	0,776077832
IIPDTTILPPIHLGL	YP_177963; PPE FAMILY PROTEIN	-3,520255727	0,800513737
GSIYIVKPKMHGPAE	MTB81 (publication)	-3,519690205	0,692864136
SGTANFGLFNSGSYN	YP_177963; PPE FAMILY PROTEIN	-3,512158326	0,850074279
SGIAMAVVGGALLYL	CAB06237.1; CONSERVED HYPOTHETICAL PROTEIN	-3,512045996	0,750934617
LEEFDHQLTRIELRR	3-OXOACYL-[ACYL-CARRIER PROTEIN] SYNTHASE 2 KASB CAA94642	-3,5109865	0,78742779
PITRDLWPAPYLVMD	MCE-FAMILY PROTEIN MCE1AYP_177701	-3,485875774	0,715819981
LNSGVLNVGSGISGL	YP_177963; PPE FAMILY PROTEIN	-3,484476542	0,81301396
TIPLAKELAWAPDEI	MTB81 (publication)	-3,473145011	0,837246828
AETTWKLLLDRQSGI	3-OXOACYL-[ACYL-CARRIER PROTEIN] SYNTHASE 2 KASB CAA94642	-3,470564498	0,867400783
AASVGATSEIEPFIV	YP_177963; PPE FAMILY PROTEIN	-3,469337406	0,821376338
QAGLEKLGSQLRGRD	MYCOBACTERIUM BOVIS MYCOCEROSIC ACID SYNTHASE GENE; M95808	-3,463759968	0,739666898
ATLAQLPPGILSDIL	PPE FAMILY PROTEIN CAE55334	-3,455996475	0,702505753
IEPFIVWTSSGAIGP	YP_177963; PPE FAMILY PROTEIN	-3,451124196	0,842272705
RPAVYAPKSALGHSV	3-OXOACYL-[ACYL-CARRIER PROTEIN] SYNTHASE 2 KASB CAA94642	-3,444563885	0,820134662
GPIIIPDTTILPPIH	YP_177963; PPE FAMILY PROTEIN	-3,428421435	0,83422836
TAQENAMEQFVYAYV	PERIPLASMIC PHOSPHATE-BINDING LIPOPROTEIN PSTS2 YP_177769	-3,424520118	0,707454626
KSHGPQGTECASLSW	MYCOBACTERIUM BOVIS MYCOCEROSIC ACID SYNTHASE GENE; M95808	-3,423423527	0,721930646
TDSFEWLIGSPRWRE	RNA POLYMERASE BETA-SUBUNIT AAA21416	-3,420992251	0,783092146
PSIPLGFAAIGHIGP	YP_177963; PPE FAMILY PROTEIN	-3,411144805	0,601587185
KSFQAKLAAAVNAIS	PERIPLASMIC PHOSPHATE-BINDING LIPOPROTEIN PSTS2 YP_177769	-3,401878841	0,789566805
QVGDRVGGFSEGGCW	MYCOBACTERIUM BOVIS MYCOCEROSIC ACID SYNTHASE GENE; M95808	-3,401437497	0,75570118
DEDELPAGVNELVRV	RNA POLYMERASE BETA-SUBUNIT AAA21416	-3,401332371	0,860608022
NVGFGNSGLGAALGI	YP_177963; PPE FAMILY PROTEIN	-3,398533577	0,84967499
IVGASNSTGMSGGFV	YP_177963; PPE FAMILY PROTEIN	-3,397731326	0,836129271

LPTVVIDRIPVELHA	YP_177963; PPE FAMILY PROTEIN	-3,385460538	0,830259135
PFAAIAVGAALVRDD	CONSERVED HYPOTHETICAL PROTEIN CAB08634	-3,385116365	0,82250264
ERLLRAIFGEKAREV	RNA POLYMERASE BETA-SUBUNIT AAA21416	-3,381286707	0,758733301
MRTHVETETGIRLTP	MYCOBACTERIUM BOVIS MYCOCEROSIC ACID SYNTHASE GENE; M95808	-3,372194997	0,818432223
HVLEIGTGWGELCIR	PUTATIVE CYCLOPROPANE-FATTY-ACYL-PHOSPHOLIPID SYNTHASE UFAA1 NP_854118	-3,368540712	0,848464499
PLGFAAIGHIGPISI	YP_177963; PPE FAMILY PROTEIN	-3,363981649	0,836774407
TVEVTADAASIMAIV	CONSERVED HYPOTHETICAL PROTEIN TB18.5CAD93033	-3,362388462	0,794266407
TVIGGTSDTVRDLIA	MYCOBACTERIUM BOVIS MYCOCEROSIC ACID SYNTHASE GENE; M95808	-3,35506598	0,793227731
EGPNIGLIGSLSVYA	RNA POLYMERASE BETA-SUBUNIT AAA21416	-3,350285965	0,780174935
MVAPDPNGERAGHAI	3-OXOACYL-[ACYL-CARRIER PROTEIN] SYNTHASE 2 KASB CAA94642	-3,345854553	0,653023489
GGPVAIQAITMPHHR	PUTATIVE CYCLOPROPANE-FATTY-ACYL-PHOSPHOLIPID SYNTHASE UFAA1 NP_854118	-3,337308215	0,71422506
PGATIPAITFPEIPA	YP_177963; PPE FAMILY PROTEIN	-3,326350355	0,812299069
ETVGVVEAHGTGTPI	MYCOBACTERIUM BOVIS MYCOCEROSIC ACID SYNTHASE GENE; M95808	-3,32316333	0,840622835
NPAKRAMLRDMGVEH	MYCOBACTERIUM BOVIS MYCOCEROSIC ACID SYNTHASE GENE; M95808	-3,321655881	0,664767512
RAAVAAFEAALAATV	YP_177963; PPE FAMILY PROTEIN	-3,320911006	0,856207142
VLNVGTLNSGVLNVG	YP_177963; PPE FAMILY PROTEIN	-3,316904482	0,828835136
SLMFVRNVGHLMTND	MTB81 (publication)	-3,312815623	0,816963737
HETMVIPPIVFLPSM	YP_177963; PPE FAMILY PROTEIN	-3,304522698	0,599950009
PGNVNTGWFNAGHTN	YP_177963; PPE FAMILY PROTEIN	-3,273753908	0,712488405
QQDHGRHLIDDALKV	HYPOTHETICAL PROTEIN RV2623 NP_217139	-3,267030283	0,866011087
DVQTDSFEWLIGSPR	RNA POLYMERASE BETA-SUBUNIT AAA21416	-3,263731604	0,861151219
GGEVSILQPFTVAPI	YP_177963; PPE FAMILY PROTEIN	-3,251171831	0,813372569
PRLFVVTRQAQIVKP	MYCOBACTERIUM BOVIS MYCOCEROSIC ACID SYNTHASE GENE; M95808	-3,249078609	0,831953815
TIIYTLTDEAPLLAT	PROBABLE ISOCITRATE DEHYDROGENASE CAA16247	-3,246615631	0,650493733
TIPAITFPEIPANAD	YP_177963; PPE FAMILY PROTEIN	-3,238956164	0,829179187
VNDALSGLGLPPPWQ	PPE FAMILY PROTEIN CAE55504	-3,238233783	0,686932316
ARRRRILFVAEAVTL	POSSIBLE GLYCOSYL TRANSFERASE CAB05415	-3,220465919	0,794249534
GGLALPLQQTIDAIE	YP_177963; PPE FAMILY PROTEIN	-3,218599282	0,818680452
TWWDSLPTDRPIVYA	POSSIBLE GLYCOSYL TRANSFERASE CAB05415	-3,208029692	0,773045154
FPVGPFFGLGVVNIS	YP_177963; PPE FAMILY PROTEIN	-3,201569019	0,817496876
MARGIDDVVVSVVAS	MYCOBACTERIUM BOVIS MYCOCEROSIC ACID SYNTHASE GENE; M95808	-3,198353224	0,783189197
RLVDAPLPAWTHARL	MYCOBACTERIUM BOVIS MYCOCEROSIC ACID SYNTHASE GENE; M95808	-3,196809999	0,851601152
PFGLFPDVDWAEVAA	PPE FAMILY PROTEIN CAE55504	-3,192899778	0,781884133
SLVAHMKPGAVLVDI	SECRETED L-ALANINE DEHYDROGENASE ALD CAA15575	-3,192365202	0,878677187
WQGDTGITYQGWQTQ	CAA16104.1; SECRETED ESAT-6 LIKE PROTEIN ESXR	-3,180591721	0,729294636
TGNFSNGILWRGNYE	YP_177963; PPE FAMILY PROTEIN	-3,180220613	0,785885828
GGFLDDVAGFDAEFF	MYCOBACTERIUM BOVIS MYCOCEROSIC ACID SYNTHASE GENE; M95808	-3,170309916	0,72840058
AGLTTLAYTAATITM	POSSIBLE HEMOLYSIN-LIKE PROTEIN CAA17201	-3,161428215	0,73203082
QPLDWFCLFSSGAAL	MYCOBACTERIUM BOVIS MYCOCEROSIC ACID SYNTHASE GENE; M95808	-3,160807238	0,872516081
AVVLDIHETMVIPPI	YP_177963; PPE FAMILY PROTEIN	-3,159279248	0,782915368
ALSGLGLPPPWQPAL	PPE FAMILY PROTEIN CAE55504	-3,155771415	0,771416738
PGPESMLAAAAAWDG	PPE FAMILY PROTEIN CAE55334	-3,154145803	0,863168367
GNTTTVPSSGFFNTG	YP_177963; PPE FAMILY PROTEIN	-3,149960583	0,85226891
VLIAFGRFPIIDDRE	MYCOBACTERIUM BOVIS MYCOCEROSIC ACID SYNTHASE GENE; M95808	-3,137678059	0,878219814
LPIVRAFAEPAGIKI	PROBABLE ISOCITRATE DEHYDROGENASE CAA16247	-3,134148491	0,86853694
RACACLQPHDPAFTF	MYCOBACTERIUM BOVIS ACYL-COA SYNTHASE GENE; U75685	-3,130895034	0,723351513
LPRVNARGGPGGAPG	MCE-FAMILY PROTEIN MCE1AYP_177701	-3,115862551	0,869292104
FDPREQPVCDGAYWV	MYCOBACTERIUM BOVIS MYCOCEROSIC ACID SYNTHASE GENE; M95808	-3,111285466	0,78825179
KLAGRHGNKGVIGKI	RNA POLYMERASE BETA-SUBUNIT AAA21416	-3,110418544	0,873121778
AWGAWGEVGRATFLA	MYCOBACTERIUM BOVIS MYCOCEROSIC ACID SYNTHASE GENE; M95808	-3,102833563	0,872328753
IALYIASIATGSIAL	PPE FAMILY PROTEIN CAE55334	-3,099916767	0,821647855
RQATTLLALLAGVFG	CAB10947.1; PROBABLE LIPOPROTEIN LPRJ	-3,095099211	0,84924794
YPLAEAADAIRAMSN	MYCOBACTERIUM BOVIS MYCOCEROSIC ACID SYNTHASE GENE; M95808	-3,092450842	0,679699496
KPPTWWDSLPTDRPI	POSSIBLE GLYCOSYL TRANSFERASE CAB05415	-3,076596906	0,771761944
TIPEFPIHWTSTGGI	YP_177963; PPE FAMILY PROTEIN	-3,074332658	0,748139712
ATLFDPREQPVCDGA	MYCOBACTERIUM BOVIS MYCOCEROSIC ACID SYNTHASE GENE; M95808	-3,070611364	0,758716368
RRILFVAEAVTLAHV	POSSIBLE GLYCOSYL TRANSFERASE CAB05415	-3,066118742	0,820522898
IVIPTPIGELDVIAL	PPE FAMILY PROTEIN CAE55334	-3,061426739	0,797548048
APQCTHYPLAEAADA	MYCOBACTERIUM BOVIS MYCOCEROSIC ACID SYNTHASE GENE; M95808	-3,059236362	0,829602017
AELDSALLAAAGFGN	MCE-FAMILY PROTEIN MCE1AYP_177701	-3,059031463	0,820990245
IPIPAIHIDFDGAAD	YP_177963; PPE FAMILY PROTEIN	-3,049875639	0,814075489
FPEIPANADGELYVI	YP_177963; PPE FAMILY PROTEIN	-3,049474623	0,823497914
MREHDIGALPICGDD	HYPOTHETICAL PROTEIN RV2626C NP_217142	-3,049339391	0,737781004
SINFADVLIAFGRFP	MYCOBACTERIUM BOVIS MYCOCEROSIC ACID SYNTHASE GENE; M95808	-3,046802306	0,844460122

AVSPEVATWLEVPLP	HYPOTHETICAL PROTEIN RV2623 NP_217139	-3,046130652	0,820995476
GAAQRAGLELLPFGG	MYCOBACTERIUM BOVIS MYCOCEROSIC ACID SYNTHASE GENE; M95808	-3,045909749	0,809419367
GTAFLRVLNRDRNYT	MTB81	-3,03974115	0,777029731
DALKSHGPQGTECAS	MYCOBACTERIUM BOVIS MYCOCEROSIC ACID SYNTHASE GENE; M95808	-3,039540242	0,792844761
GNVGDFNLGAANLGD	YP_177963; PPE FAMILY PROTEIN	-3,038461686	0,831214255
CSKVPDIHDVALMED	MTB81	-3,029235697	0,83199424
GILWRGNYEGLAGFS	YP_177963; PPE FAMILY PROTEIN	-3,029144077	0,817167163
LHALHYHQVDVAAVQ	MTB81 (publication)	-3,029022556	0,875760866
ISNFHSPSDVIVDAS	PROBABLE ISOCITRATE DEHYDROGENASE CAA16247	-3,027880335	0,877600593
WVFSGQGSQWAAMGT	MYCOBACTERIUM BOVIS MYCOCEROSIC ACID SYNTHASE GENE; M95808	-3,026061966	0,731992904
WQGDTGITYQAWQAQ	CAA17363.1; LOW MOLECULAR WEIGHT PROTEIN ANTIGEN 7 ESXH	-3,020949729	0,820481894
DELQAQIDKWHRRRV	MTB81	-3,017902997	0,817201513
DILVGKVTPKGETEL	RNA POLYMERASE BETA-SUBUNIT AAA21416	-3,017197581	0,800332091
PFDLPTVVIDRIPVE	YP_177963; PPE FAMILY PROTEIN	-3,008423826	0,833102289
HQIPLGFQVPGSIDA	YP_177963; PPE FAMILY PROTEIN	-3,003339412	0,871580282
LHSVKVIPSRGAWLE	RNA POLYMERASE BETA-SUBUNIT AAA21416	-3,002009962	0,801639394
FSNGILWRGNYEGLA	YP_177963; PPE FAMILY PROTEIN	-2,99853645	0,793885605
GQPVSRLVIRGEHRD	CAA17343.1; HEAT SHOCK PROTEIN HSP	-2,995767003	0,861172447
ANSGNVDTGALMSGN	YP_177963; PPE FAMILY PROTEIN	-2,995338631	0,876255969
DADRSFIEYGLDSLG	MYCOBACTERIUM BOVIS MYCOCEROSIC ACID SYNTHASE GENE; M95808	-2,986803333	0,879494501
LVTGKAFPYVVVTGI	3-OXOACYL-[ACYL-CARRIER PROTEIN] SYNTHASE 2 KASB CAA94642	-2,983513149	0,768702173
VAAAGFGHRVEIDLC	PUTATIVE CYCLOPROPANE-FATTY-ACYL-PHOSPHOLIPID SYNTHASE UFAA1 NP_854118	-2,98274266	0,844885109
RKHGLSSLGWDLCRI	POSSIBLE GLYCOSYL TRANSFERASE CAB05418	-2,974585333	0,593443274
QRAGLELLPFGGRFV	MYCOBACTERIUM BOVIS MYCOCEROSIC ACID SYNTHASE GENE; M95808	-2,968527236	0,860280542
LGSQLRGRDGVVIVY	MYCOBACTERIUM BOVIS MYCOCEROSIC ACID SYNTHASE GENE; M95808	-2,967713369	0,799805226
DAEFCGRIHTRYSSA	SECRETED L-ALANINE DEHYDROGENASE ALD CAA15575	-2,964818972	0,838367424
EFDLPVRIGGHLLEE	3-OXOACYL-[ACYL-CARRIER PROTEIN] SYNTHASE 2 KASB CAA94642	-2,95765668	0,864267002
NAGDVAYRPMAPNFD	MTB81	-2,953293108	0,86873004
TGDIGPATIIPPIHI	YP_177963; PPE FAMILY PROTEIN	-2,951860987	0,877164132
ITGTRIDPIPLNFDV	YP_177963; PPE FAMILY PROTEIN	-2,946548793	0,860420859
RNWLGLNKGDLAAAV	MTB81	-2,945377828	0,841053286
PGFTIPTGPIHIGLP	YP_177963; PPE FAMILY PROTEIN	-2,942366696	0,845243696
DAAKGVPDWAARLPD	RNA POLYMERASE BETA-SUBUNIT AAA21416	-2,941656386	0,892461976
VLNSLTGAAQRAGLE	MYCOBACTERIUM BOVIS MYCOCEROSIC ACID SYNTHASE GENE; M95808	-2,930937479	0,845699732
GTGTPIGDPIEYRSL	MYCOBACTERIUM BOVIS MYCOCEROSIC ACID SYNTHASE GENE; M95808	-2,926924819	0,787648857
PPPQRAAMAAQLQAV	LOW MOLECULAR WEIGHT T-CELL ANTIGEN TB8.4 NP_215690	-2,911737914	0,840604462
FALLALPAHDGRVVL	POSSIBLE HEMOLYSIN-LIKE PROTEIN CAA17201	-2,906493991	0,823730751
AGSPEVDTNRLMVSI	3-OXOACYL-[ACYL-CARRIER PROTEIN] SYNTHASE 2 KASB CAA94642	-2,902112966	0,874942999
NTRIVLGGYSQGATV	PROBABLE CUTINASE PRECURSOR CFP21 NP_216500	-2,901489482	0,884338297
ANRSRLMSLVAANIL	PPE FAMILY PROTEIN CAE55334	-2,88702449	0,805486857
TAVAAICHYIAMWFV	POSSIBLE HEMOLYSIN-LIKE PROTEIN CAA17201	-2,884487775	0,784671686
VLADWDFNIRCFSNP	POSSIBLE GLYCOSYL TRANSFERASE CAB05419	-2,882895966	0,735910945
FLALGAGFEDIGIKT	PROBABLE ISOCITRATE DEHYDROGENASE CAA16247	-2,879490031	0,864893835
GYVVRWVDQGVGCSK	MTB81	-2,874252389	0,782502817
TVTGIDKVQPAVFAV	MYCOBACTERIUM BOVIS MYCOCEROSIC ACID SYNTHASE GENE; M95808	-2,870184496	0,833750751
GTGNIGFFNSGTGNW	YP_177963; PPE FAMILY PROTEIN	-2,869443987	0,849980214
MAPNFDDSIAFLAAQ	MTB81	-2,854075636	0,843307029
LPLGLGLSGGTPPFD	YP_177963; PPE FAMILY PROTEIN	-2,850171103	0,849764137
NTGGFNPGSTNTGWF	YP_177963; PPE FAMILY PROTEIN	-2,850042114	0,86480222
FAVGPDGMPGGEVSI	YP_177963; PPE FAMILY PROTEIN	-2,848596564	0,848204503
CWTLVSSRSARASSR	MYCOBACTERIUM BOVIS MYCOCEROSIC ACID SYNTHASE GENE; M95808	-2,848569275	0,789816579
LVIVLVRRWPKVISR	POSSIBLE GLYCOSYL TRANSFERASE CAB05419	-2,836868054	0,821151497
TIGGQTYTIPLDTPP	YP_177963; PPE FAMILY PROTEIN	-2,832010431	0,822025173
ATGFTVQDGQLVVAL	MTB81	-2,823693246	0,849406137
FLLDQAITSAGRHPD	CAB01474.1; CONSERVED HYPOTHETICAL PROTEIN CFP17	-2,821870586	0,822659656
GFAVPDIPIPAIHID	YP_177963; PPE FAMILY PROTEIN	-2,814319606	0,809103725
AQGLPVSAIAWGAWG	MYCOBACTERIUM BOVIS MYCOCEROSIC ACID SYNTHASE GENE; M95808	-2,811294741	0,856839955
PAGRLVDAPLPAWTH	MYCOBACTERIUM BOVIS MYCOCEROSIC ACID SYNTHASE GENE; M95808	-2,811132491	0,82653376
WAQELAAQTDDQQLA	PROBABLE ISOCITRATE DEHYDROGENASE CAA16247	-2,800095559	0,869593256
LGSPGQGAYAAANSW	MYCOBACTERIUM BOVIS MYCOCEROSIC ACID SYNTHASE GENE; M95808	-2,799631118	0,791273241
GPQGTECASLSWSVQ	MYCOBACTERIUM BOVIS MYCOCEROSIC ACID SYNTHASE GENE; M95808	-2,799597152	0,798137416
MNCRAVADHLTRLVD	CONSERVED HYPOTHETICAL PROTEIN CAB08634	-2,793288989	0,778448266
GVLNSGVDISGMFNV	YP_177963; PPE FAMILY PROTEIN	-2,792326279	0,877373241
GIVRIGAEVRDGDIL	RNA POLYMERASE BETA-SUBUNIT AAA21416	-2,790902181	0,73182861
PYNHMEVGSPYAVEY	MCE-FAMILY PROTEIN MCE1AYP_177701	-2,790349876	0,742528254

EITGSTLVDINETLN	YP_177963; PPE FAMILY PROTEIN	-2,788763747	0,711488252
PTPIGELDVIALYIA	PPE FAMILY PROTEIN CAE55334	-2,786721116	0,831422713
TPGSITYNEWSFAQA	PERIPLASMIC PHOSPHATE-BINDING LIPOPROTEIN PSTS3 YP_177768	-2,77675836	0,739815139
DPDSFWAGVDKVVAD	MTB81	-2,776748082	0,745378443
SGSTAQENAMEQFVY	PERIPLASMIC PHOSPHATE-BINDING LIPOPROTEIN PSTS2 YP_177769	-2,775316035	0,748105541
TSTGGIGPIIIPDTT	YP_177963; PPE FAMILY PROTEIN	-2,775197692	0,864121774
FSFFAVGPDGMPGGE	YP_177963; PPE FAMILY PROTEIN	-2,765628636	0,822297957
SEGGCWRTFLTCDAN	MYCOBACTERIUM BOVIS MYCOCEROSIC ACID SYNTHASE GENE; M95808	-2,758393326	0,865358021
GINYRVLWQAAGPDT	CAB06500.1; IMMUNOGENIC PROTEIN MPT63	-2,752265033	0,836818079
NADGELYVIAGDIPL	YP_177963; PPE FAMILY PROTEIN	-2,743611558	0,789817279
YLQSLPAHLRPGLDE	PPE FAMILY PROTEIN CAE55504	-2,743170572	0,868746704
RSGRRSVAVTPEQRP	MYCOBACTERIUM BOVIS MYCOCEROSIC ACID SYNTHASE GENE; M95808	-2,741850957	0,864278325
SLAHFADGWNTFNLT	MTB48 AAK31576	-2,737532922	0,790848624
PLTIDIDGQIDGFST	YP_177963; PPE FAMILY PROTEIN	-2,733579275	0,856021963
QDEWAGRLRRLLVEQ	MYCOBACTERIUM BOVIS MYCOCEROSIC ACID SYNTHASE GENE; M95808	-2,728793042	0,869269784
DTPPNDQAGLEKLGS	MYCOBACTERIUM BOVIS MYCOCEROSIC ACID SYNTHASE GENE; M95808	-2,726771959	0,816248238
LAKELAWAPDEIREE	MTB81	-2,724639047	0,867206115
REQPVCDGAYWVDNL	MYCOBACTERIUM BOVIS MYCOCEROSIC ACID SYNTHASE GENE; M95808	-2,717266352	0,878766459
DYSALYPAGRLVDAP	MYCOBACTERIUM BOVIS MYCOCEROSIC ACID SYNTHASE GENE; M95808	-2,716185962	0,809328202
MSGGFVTAPTQGILI	YP_177963; PPE FAMILY PROTEIN	-2,713502485	0,855242486
VEHVYDSRSVEFAEQ	MYCOBACTERIUM BOVIS MYCOCEROSIC ACID SYNTHASE GENE; M95808	-2,711688174	0,877841057
WMHLMASDFHAFSAA	MYCOBACTERIUM BOVIS ACYL-COA SYNTHASE GENE; U75685	-2,709535161	0,815426548
AGVNELVRVYVAQKR	RNA POLYMERASE BETA-SUBUNIT AAA21416	-2,708687035	0,866858883
DVPRSGRRSVAVTPE	MYCOBACTERIUM BOVIS MYCOCEROSIC ACID SYNTHASE GENE; M95808	-2,702215351	0,861398978
GDVLVDADGKAMLFD	RNA POLYMERASE BETA-SUBUNIT AAA21416	-2,698728865	0,845657119
MRESFAERGVTLGAA	MYCOBACTERIUM BOVIS MYCOCEROSIC ACID SYNTHASE GENE; M95808	-2,680320564	0,858515307
FAPNLGERLIIHRDT	POSSIBLE GLYCOSYL TRANSFERASE CAB05419	-2,676180043	0,83333613
DVLAAGDFWGGAGSV	PUTATIVE ESAT-6 LIKE PROTEIN ESXN YP_177838	-2,673770913	0,815865195
YAEYQQRSEKVLTEY	MTB48 AAK31576	-2,67201692	0,565350017
