# Supplementary material for: Pattern Recognition in Pulmonary Tuberculosis Defined by High Content Peptide Microarray Chip Analysis Representing 61 Proteins from M. tuberculosis
Source: PLoS One. 2008 Dec 9;3(12):e3840. doi: 10.1371/journal.pone.0003840 (PMC2588537; doi:10.1371/journal.pone.0003840)
Supplement: Table S4 — Top 24 peptides recognized by TB+ patients(n = 35) but not by TB− controls (n = 34) and vice versa (0.02 MB PDF) [file pone.0003840.s004.pdf]

Supplementary Table 4. Top 24 peptides recognized by TB+ patients(n=35) but not by TB- controls (n=34) and vice versa

Target Protein	Only in TB + individuals<br>aa sequence and peptide location	Only in TB- individuals<br>aa sequence and peptide location
SECRETED ANTIGEN 85-B FBPB (85B) CAB10044 Rv1886c	QSSFYSDWYSPACGK 39	YNGWDINTPAFEWYY 030<br>SPACGKAGCQTYKWE 042
PROBABLE LIPOPROTEIN LPRJ CAB10947 Rv1690	MTAHTHDGTRTWRTG 1	
POSSIBLE GLYCOSYL TRANSFERASE CAB05415 Rv2962c	YRLERPLLFALQCMP 204<br>VRRRHGLSSLGWNLC 210<br>RHGLSSLGWNLCRIF 211	
POSSIBLE GLYCOSYL TRANSFERASE CAB05418 Rv2958c	AIANAYWSPQARRRF 50<br>RRFPLPDVPWTRFFG 54	PLIFALYCLPLNWLR 063
POSSIBLE GLYCOSYL TRANSFERASE CAB05419 Rv2957	AFDLDRLLFKRNICH 52	
M. BOVIS MYCOCEROSIC ACID SYNTHASE GENE M95808	GYIPILGAPWLADLV 660<br>PILGAPWLADLVRRS 661<br>FRMELLSLPQDEWAG 672	TVFKLTADGVLTAPQ 056<br>SSGAALLGSPGQGAY 063<br>VHPLLGSHVRLTEEP 029
RNA POLYMERASE BETA-SUBUNIT AAA21416 L27989	KAMLFDGRSGEPFPY 336	
POSSIBLE HEMOLYSIN CAA16235, Rv3922c	IRLAKCGPWHRGGWD 24<br>LRPASCRFVPTCSQY 13	AVDALTEYGLLRGSW 018
PPE FAMILY PROTEIN YP_177963 (Rv3347c)	ALMSGNFSNGILWRG 333<br>PFNVNLKLQFLHDAF 373	AAAAMAAAAAPYAGW 021<br>FRLPLLFVNALGDNW 578<br>NIGNANVGFGNIGHG 674
PPE FAMILY ROTEIN CAE55489 (Rv2430c)	WRSLDVEMTAVQRSV 011	NRTLLSLMDAWAGPV 016<br>AMTRAASPYVGWLNT 022
TRANSMEMBRANE SERINE/THREONINE-PROTEIN KINASE D PKND NP_215446 Rv0931c	YRADSVERLIAAHLM 072	APWGIAVDEAGTVYV 199
PROBABLE ISOCITRATE DEHYDROGENASE CAA16247 Rv0066c	GELDNRGSQFYLAMY 219	RMCIVKDAPIRDWVK 154
PUTATIVE CYCLOPROPANE-FATTY-ACYL-PHOSPHOLIPID-SYNTHASE NP_854118 Mb0455c	HWLRPITPTFRPSWP 042<br>EMIEAVGYRSWPRYF 094	LGFDEVFARMWELYL 130
CONSERVED HYPOTHETICAL PROTEIN CAB06237 Rv3864	GVYKVCKGLEKIPLL 038	
MTB48 AAK31576 AY029285	QAQYVAQLHVWARRE 070	
SECRETED L-ALANINE DEHYDROGENASE ALD CAA15575 Rv2780	FCGRIHTRYSSAYEL 071	
IMMUNOGENIC PROTEIN MPT64 CAA98382 Rv1980c		GGTHPTTTYKAFDWD 090<br>TTYKAFDWDQAYRKP 092
SECRETED ESAT6-LIKE PROTEIN ESXR CAA16104 (Rv3019c) = TB10.3		IMYNYPAMMAHAGDM 002
LOW MOLECULAR WEIGHT PROTEIN ANTIGEN 7 ESXH CAA17363 Rv0288		IMYNYPAMLGHAGDM 002
PROBABLE CUTINASE PRECURSOR CFP21 NP_216500		GFSSMLWGGGSLPTI 051

Rv1984c		
CONSERVED HYPOTHETICAL PROTEIN CAB08634 Rv2629		AIAVGAALVRDDNRI 113
6 KDA EARLY SECRETORY ANTIGENIC TARGET ESXA (ESAT-6) CAE55648 Rv3875		AAASAIQGNVTSIHS 005

Listing of the peptides was defined by the index. Note that IgG target epitope recognition segregates not into distinct proteins, yet the recognition pattern segregates into specific, different locations on proteins.
