# Supplementary material for: Pattern Recognition in Pulmonary Tuberculosis Defined by High Content Peptide Microarray Chip Analysis Representing 61 Proteins from M. tuberculosis
Source: PLoS One. 2008 Dec 9;3(12):e3840. doi: 10.1371/journal.pone.0003840 (PMC2588537; doi:10.1371/journal.pone.0003840)
Supplement: Table S5 — Comparison of MTB peptides defined by IgG in patients from Armenia and from Sweden. (0.19 MB PDF) [file pone.0003840.s005.pdf]

Supplementary Table 5. Comparison of MTB peptides defined by IgG in patients from Armenia and from Sweden.

Peptides	Protein	Index value in 34 TB+ patients from Armenia		Peptides	Protein	Index value in 6 TB+ patients from Sweden
QSSFYSDWYSPACGK	CAB10044.1; SECRETED ANTIGEN 85-B FBPB (85B)	1,7776		MGCRLPGGINSPDKL	MYCOBACTERIUM BOVIS MYCOCEROSIC ACID SYNTHASE GENE; M95808	3,2663
MTAHTHDGTRTWRTG	CAB10947.1; PROBABLE LIPOPROTEIN LPRJ	1,4567		WLADLVRRSPWGEMF	MYCOBACTERIUM BOVIS MYCOCEROSIC ACID SYNTHASE GENE; M95808	2,0054
VRRRHGLSSLGWNLC	POSSIBLE GLYCOSYL TRANSFERASE CAB05415	1,3994		TGRSLDMSVAALAGM	MYCOBACTERIUM BOVIS MYCOCEROSIC ACID SYNTHASE GENE; M95808	1,9423
PILGAPWLADLVRRS	MYCOBACTERIUM BOVIS MYCOCEROSIC ACID SYNTHASE GENE; M95808	1,3324		PILGAPWLADLVRRS	MYCOBACTERIUM BOVIS MYCOCEROSIC ACID SYNTHASE GENE; M95808	1,8986
KAMLFDGRSGEPFPY	RNA POLYMERASE BETA-SUBUNIT AAA21416	1,2314		KAMLFDGRSGEPFPY	RNA POLYMERASE BETA-SUBUNIT AAA21416	1,8126
IRLAKCGPWHRGGWD	POSSIBLE HEMOLYSIN CAA16235	1,141		VKAPGFGDRRKAMLQ	60 kDa chaperonin 2 P0A521	1,7968
ALMSGNFSNGILWRG	YP_177963; PPE FAMILY PROTEIN	1,0973		PTIMGEVRRHFRDNS	ALTERNATE RNA POLYMERASE SIGMA FACTOR SIGF CAB07069	1,7872
YRADSVERLIAAHLM	TRANSMEMBRANE SERINE/THREONINE-PROTEIN KINASE D PKND NP_215446	1,0307		GMYNFKESIRDFARA	CAA17111.1; PROBABLE ISOCITRATE DEHYDROGENASE	1,7756
GYIPILGAPWLADLV	MYCOBACTERIUM BOVIS MYCOCEROSIC ACID SYNTHASE GENE; M95808	0,956		GELDNRGSQFYLAMY	PROBABLE ISOCITRATE DEHYDROGENASE CAA16247	1,7505
FRMELLSLPQDEWAG	MYCOBACTERIUM BOVIS MYCOCEROSIC ACID SYNTHASE GENE; M95808	0,9553		LTAANTKKVAMAAPM	POSSIBLE GLYCOSYL TRANSFERASE CAB05419	1,7085
AFDLDRLLFKRNICH	POSSIBLE GLYCOSYL TRANSFERASE CAB05419	0,9176		AFDLDRLLFKRNICH	POSSIBLE GLYCOSYL TRANSFERASE CAB05419	1,6717
GELDNRGSQFYLAMY	PROBABLE ISOCITRATE DEHYDROGENASE CAA16247	0,8795		MTAHTHDGTRTWRTG	CAB10947.1; PROBABLE LIPOPROTEIN LPRJ	1,6451
YRLERPLLFALQCMP	POSSIBLE GLYCOSYL TRANSFERASE CAB05415	0,8753		QSSFYSDWYSPACGK	CAB10044.1; SECRETED ANTIGEN 85-B FBPB (85B)	1,6311
HWLRPITPTFRPSWP	PUTATIVE CYCLOPROPANE-FATTY-ACYL-	0,783		VIPTMTPPPGMVRQR	CAA17582.1; PROBABLE SERINE PROTEASE PEPD	1,5876

	PHOSPHOLIPID SYNTHASE UFAA1 NP_854118					
PFNVNLKLQFLHDAF	YP_177963; PPE FAMILY PROTEIN	0,7652		LKMCWPAAPRSVGVP	POSSIBLE HEMOLYSIN-LIKE PROTEIN CAA17201	1,5409
AIANAYWSPQARRRF	POSSIBLE GLYCOSYL TRANSFERASE CAB05418	0,7299		RSLHWLRPITPTFRP	PUTATIVE CYCLOPROPANE-FATTY-ACYL-PHOSPHOLIPID SYNTHASE UFAA1 NP_854118	1,5054
WRSLDVEMTAVQRSF	PPE FAMILY PROTEIN CAE55489	0,7127		ALMSGNFSNGILWRG	YP_177963; PPE FAMILY PROTEIN	1,4496
RRFPLPDVPWTRFFG	POSSIBLE GLYCOSYL TRANSFERASE CAB05418	0,6965		YRADSVERLIAAHLM	TRANSMEMBRANE SERINE/THREONINE-PROTEIN KINASE D PKND NP_215446	1,432
GVYKVCKGLEKIPLL	CAB06237.1; CONSERVED HYPOTHETICAL PROTEIN	0,6757		FCGRIHTRYSSAYEL	SECRETED L-ALANINE DEHYDROGENASE ALD CAA15575	1,4222
EMIEAVGYRSWPRYF	PUTATIVE CYCLOPROPANE-FATTY-ACYL-PHOSPHOLIPID SYNTHASE UFAA1 NP_854118	0,6748		GLRGLLRVISSEHPM	MYCOBACTERIUM BOVIS MYCOCEROSIC ACID SYNTHASE GENE; M95808	1,3622
QAQYVAQLHVWARRE	MTB48 AAK31576	0,6624		HWLRPITPTFRPSWP	PUTATIVE CYCLOPROPANE-FATTY-ACYL-PHOSPHOLIPID SYNTHASE UFAA1 NP_854118	1,3283
FCGRIHTRYSSAYEL	SECRETED L-ALANINE DEHYDROGENASE ALD CAA15575	0,6143		GYIPILGAPWLADLV	MYCOBACTERIUM BOVIS MYCOCEROSIC ACID SYNTHASE GENE; M95808	1,27
LRPASCRFVPTCSQY	POSSIBLE HEMOLYSIN CAA16235	0,6097		WGGAFDLDRLLFKRN	POSSIBLE GLYCOSYL TRANSFERASE CAB05419	1,2687
RHGLSSLGWNLCRIF	POSSIBLE GLYCOSYL TRANSFERASE CAB05415	0,5962		AMRTLRKYVEADLRV	POSSIBLE GLYCOSYL TRANSFERASE CAB05415	1,2657
WGGAFDLDRLLFKRN	POSSIBLE GLYCOSYL TRANSFERASE CAB05419	0,5502		LSPLRPASCRFVPTC	POSSIBLE HEMOLYSIN CAA16235	1,2146
RLVKLLYRLERPLLF	POSSIBLE GLYCOSYL TRANSFERASE CAB05415	0,5502		LRPASCRFVPTCSQY	POSSIBLE HEMOLYSIN CAA16235	1,2138
ARASFSYGLNAKWPV	CAA17111.1; PROBABLE ISOCITRATE DEHYDROGENASE	0,5338		YCGKYKRVRFKGIIC	RNA POLYMERASE BETA'-SUBUNIT AAA21417	1,1718
DVPWTRFFGVRPVSI	POSSIBLE GLYCOSYL TRANSFERASE CAB05418	0,5333		PKVISRAMVMRTVIS	POSSIBLE GLYCOSYL TRANSFERASE CAB05419	1,1702
WLADLVRRSPWGEMF	MYCOBACTERIUM BOVIS MYCOCEROSIC ACID SYNTHASE GENE; M95808	0,5301		PEFQRHRDKIVQRCL	ALTERNATE RNA POLYMERASE SIGMA FACTOR SIGF CAB07069	1,1516
PWDGRNHYLAAVYRT	CAA16030.1;	0,5262		LRLMPENRPRTWLAS	POSSIBLE	1,125

	PROBABLE MOLYBDOPTERIN-GUANINE DINUCLEOTIDE BIOSYNTHESIS PROTEIN				GLYCOSYL TRANSFERASE CAB05415	
LVVKRGPNAGSRFLL	CAB01474.1; CONSERVED HYPOTHETICAL PROTEIN CFP17	0,5166		PVTHIWYFKGVPSRL	RNA POLYMERASE BETA'-SUBUNIT AAA21417	1,0922
GVRLAFVCAVDMPYL	CAA16030.1; PROBABLE MOLYBDOPTERIN-GUANINE DINUCLEOTIDE BIOSYNTHESIS PROTEIN	0,5089		IRLAKCGPWHRGGWD	POSSIBLE HEMOLYSIN CAA16235	1,0881
LSPLRPASCRFVPTC	POSSIBLE HEMOLYSIN CAA16235	0,4868		AVAVGCAQPACLRAY	MYCOBACTERIUM BOVIS MYCOCEROSIC ACID SYNTHASE GENE; M95808	1,0307
PVTHIWYFKGVPSRL	RNA POLYMERASE BETA'-SUBUNIT AAA21417	0,4781		YRLERPLLFALQCMP	POSSIBLE GLYCOSYL TRANSFERASE CAB05415	1,0282
VKGAWVLARYDDGRP	CONSERVED HYPOTHETICAL PROTEIN TB18.5CAD93033	0,4771		DVPWTRFFGVRPVSI	POSSIBLE GLYCOSYL TRANSFERASE CAB05418	1,0253
AITNTARPWHIGLYG	PPE FAMILY PROTEIN CAE55334	0,4769		NMREYLAAGAKERQR	MTB48 AAK31576	1,001
FRHHAIHTIPSERFF	POSSIBLE GLYCOSYL TRANSFERASE CAB05415	0,465		GVRLAFVCAVDMPYL	CAA16030.1; PROBABLE MOLYBDOPTERIN-GUANINE DINUCLEOTIDE BIOSYNTHESIS PROTEIN	0,9976
FNIRCFSNPALVTRY	POSSIBLE GLYCOSYL TRANSFERASE CAB05419	0,4586		VRRRHGLSSLGWNLC	POSSIBLE GLYCOSYL TRANSFERASE CAB05415	0,9946
PSERFFGNLTQGRFY	POSSIBLE GLYCOSYL TRANSFERASE CAB05415	0,4571		PVAVIGMGCRLPGGI	MYCOBACTERIUM BOVIS MYCOCEROSIC ACID SYNTHASE GENE; M95808	0,9943
PTIMGEVRRHFRDNS	ALTERNATE RNA POLYMERASE SIGMA FACTOR SIGF CAB07069	0,4569		WRSLDVEMTAVQRSF	PPE FAMILY PROTEIN CAE55489	0,9935
FTLPGRSLMFVRNVG	MTB81 (publication)	0,4539		AIANAYWSPQARRRF	POSSIBLE GLYCOSYL TRANSFERASE CAB05418	0,9873
SQFGPYQLLRLLGRG	TRANSMEMBRANE SERINE/THREONINE-PROTEIN KINASE D PKND NP_215446	0,451		FRMELLSLPQDEWAG	MYCOBACTERIUM BOVIS MYCOCEROSIC ACID SYNTHASE GENE; M95808	0,9579
PDFKWPPTPGSPLFP	CAB06237.1; CONSERVED HYPOTHETICAL PROTEIN	0,4411		RMRKFARSNHGTCAN	RNA POLYMERASE BETA-SUBUNIT AAA21416	0,9493
RTLRKYIAADRKILN	POSSIBLE GLYCOSYL TRANSFERASE CAB05418	0,4273		GVYKVCKGLEKIPLL	CAB06237.1; CONSERVED HYPOTHETICAL PROTEIN	0,9396

IQMSDPAYNINISLP	CAA98382.1; IMMUNOGENIC PROTEIN MPT64	0,4247		QAQYVAQLHVWARRE	MTB48 AAK31576	0,8876
NYRYAINNSFGFGGH	3-OXOACYL-[ACYL-CARRIER PROTEIN] SYNTHASE 2 KASB CAA94642	0,4105		FACDPRFNKLLGPLP	POSSIBLE GLYCOSYL TRANSFERASE CAB05418	0,8726
SFYRPKRPGSYPIVL	PERIPLASMIC PHOSPHATE-BINDING LIPOPROTEIN PSTS3 YP_177768	0,407		LVVKRGPNAGSRFLL	CAB01474.1; CONSERVED HYPOTHETICAL PROTEIN CFP17	0,869
AATIKRFADRFARFN	MYCOBACTERIUM BOVIS ACYL-COA SYNTHASE GENE; U75685	0,4066		HPIVFGHAVRIFYKD	PROBABLE ISOCITRATE DEHYDROGENASE CAA16247	0,8627
LRISSQLLANWLRHG	MTB81 (publication)	0,4058		KIHARSTGPYSMITQ	RNA POLYMERASE BETA-SUBUNIT AAA21416	0,855
YNQLLGPLPFRHHAI	POSSIBLE GLYCOSYL TRANSFERASE CAB05415	0,4034		SRTGSIYIVKPKMHG	MTB81 (publication)	0,8511
FTLPGERIHVVFRSD	PERIPLASMIC PHOSPHATE-BINDING LIPOPROTEIN PSTS3 YP_177768	0,392		RRFPLPDVPWTRFFG	POSSIBLE GLYCOSYL TRANSFERASE CAB05418	0,8376
HPIVFGHAVRIFYKD	PROBABLE ISOCITRATE DEHYDROGENASE CAA16247	0,3856		PDFKWPPTPGSPLFP	CAB06237.1; CONSERVED HYPOTHETICAL PROTEIN	0,8202
RSLHWLRPITPTFRP	PUTATIVE CYCLOPROPANE-FATTY-ACYL-PHOSPHOLIPID SYNTHASE UFAA1 NP_854118	0,3799		RQRVAAAGFGHRVEI	PUTATIVE CYCLOPROPANE-FATTY-ACYL-PHOSPHOLIPID SYNTHASE UFAA1 NP_854118	0,7801
MLATRHTQTWIQKYI	PUTATIVE CYCLOPROPANE-FATTY-ACYL-PHOSPHOLIPID SYNTHASE UFAA1 NP_854118	0,3777		ARASFSYGLNAKWPV	CAA17111.1; PROBABLE ISOCITRATE DEHYDROGENASE	0,7763
SCVYATASRWGGPPV	POSSIBLE GLYCOSYL TRANSFERASE CAB05415	0,3697		RCLPLADHIARRFEG	ALTERNATE RNA POLYMERASE SIGMA FACTOR SIGF CAB07069	0,7725
VRPVSILYRLYRPLI	POSSIBLE GLYCOSYL TRANSFERASE CAB05418	0,3589		AVLDPDHDGMRVQVR	MYCOBACTERIUM BOVIS MYCOCEROSIC ACID SYNTHASE GENE; M95808	0,7721
FYRRGLFGTIGPYNL	POSSIBLE GLYCOSYL TRANSFERASE CAB05419	0,3525		MLATRHTQTWIQKYI	PUTATIVE CYCLOPROPANE-FATTY-ACYL-PHOSPHOLIPID SYNTHASE UFAA1 NP_854118	0,7599
TRYSSAYELEGAVKR	SECRETED L-ALANINE DEHYDROGENASE ALD CAA15575	0,3507		SCVYATASRWGGPPV	POSSIBLE GLYCOSYL TRANSFERASE CAB05415	0,7582
AVAVGCAQPACLRAY	MYCOBACTERIUM BOVIS MYCOCEROSIC ACID SYNTHASE GENE; M95808	0,3436		FTLPGRSLMFVRNVG	MTB81 (publication)	0,747
RYARCLGSAVNPVLR	PROBABLE	0,3428		EMIEAVGYRSWPRYF	PUTATIVE	0,7395

	ISOCITRATE DEHYDROGENASE CAA16247				CYCLOPROPANE-FATTY-ACYL-PHOSPHOLIPID SYNTHASE UFAA1 NP_854118	
QAIFYRRGLFGTIGP	POSSIBLE GLYCOSYL TRANSFERASE CAB05419	0,3288		PWDGRNHYLAAVYRT	CAA16030.1; PROBABLE MOLYBDOPTERIN-GUANINE DINUCLEOTIDE BIOSYNTHESIS PROTEIN	0,7307
EAVGYRSWPRYFAAL	PUTATIVE CYCLOPROPANE-FATTY-ACYL-PHOSPHOLIPID SYNTHASE UFAA1 NP_854118	0,3231		MIDGTSLRALLKQYG	TRANSMEMBRANE SERINE/THREONINE-PROTEIN KINASE D PKND NP_215446	0,7116
RSEYRQAAARLADAF	POSSIBLE GLYCOSYL TRANSFERASE CAB05415	0,3201		SPAMVAANRTRLASL	PPE FAMILY PROTEIN CAE55504	0,7048
RKYIAADRKILNEIA	POSSIBLE GLYCOSYL TRANSFERASE CAB05418	0,3156		EQAGLRGLLRVISSE	MYCOBACTERIUM BOVIS MYCOCEROSIC ACID SYNTHASE GENE; M95808	0,6865
LFIHRPDALARRIGR	PUTATIVE CYCLOPROPANE-FATTY-ACYL-PHOSPHOLIPID SYNTHASE UFAA1 NP_854118	0,3114		KYKRVRFKGIICERC	RNA POLYMERASE BETA'-SUBUNIT AAA21417	0,6861
NGDEVQIGKFRLVFL	CAB01474.1; CONSERVED HYPOTHETICAL PROTEIN CFP17	0,2974		MSVAALAGMRREQPL	MYCOBACTERIUM BOVIS MYCOCEROSIC ACID SYNTHASE GENE; M95808	0,6787
FGVRLVKLLYRLERP	POSSIBLE GLYCOSYL TRANSFERASE CAB05415	0,2921		RTLRKYIAADRKILN	POSSIBLE GLYCOSYL TRANSFERASE CAB05418	0,6733
RVNPFGFIETPYRKV	RNA POLYMERASE BETA-SUBUNIT AAA21416	0,2893		RVNPFGFIETPYRKV	RNA POLYMERASE BETA-SUBUNIT AAA21416	0,6619
GMYNFKESIRDFARA	CAA17111.1; PROBABLE ISOCITRATE DEHYDROGENASE	0,2874		GDFYAGEKSMTLDRA	PROBABLE ISOCITRATE DEHYDROGENASE CAA16247	0,6608
FRSGYLDVYQWTLIR	PUTATIVE CYCLOPROPANE-FATTY-ACYL-PHOSPHOLIPID SYNTHASE UFAA1 NP_854118	0,2852		RGPGQMLGGLPVGQM	CAE55371.1; PPE FAMILY PROTEIN	0,6593
YRATNFKVDQPGTVT	CAA17111.1; PROBABLE ISOCITRATE DEHYDROGENASE	0,2784		DLVRRSPWGEMFAST	MYCOBACTERIUM BOVIS MYCOCEROSIC ACID SYNTHASE GENE; M95808	0,6586
FGTIGPYNLRYRVLA	POSSIBLE GLYCOSYL TRANSFERASE CAB05419	0,2738		RVGFPQHVENALRLM	POSSIBLE GLYCOSYL TRANSFERASE CAB05415	0,6453
TGDEVTYRADIYALA	TRANSMEMBRANE SERINE/THREONINE-PROTEIN KINASE D PKND NP_215446	0,2714		KIRAWGRRLMIGTAA	CAB10044.1; SECRETED ANTIGEN 85-B FBPB (85B)	0,6345
PLNWLRRKHGLSSLG	POSSIBLE	0,2405		AITNTARPWHIGLYG	PPE FAMILY	0,6268

	GLYCOSYL TRANSFERASE CAB05418				PROTEIN CAE55334	
WSVKVPRRLKELHLR	ALTERNATE RNA POLYMERASE SIGMA FACTOR SIGF CAB07069	0,2347		PPSLVAANRSRLMSL	PPE FAMILY PROTEIN CAE55334	0,6126
KAVKEYARKHPHSMG	PROBABLE ISOCITRATE DEHYDROGENASE CAA16247	0,2334		DPTQQIPKLVANNTR	CAB10044.1; SECRETED ANTIGEN 85-B FBPB (85B)	0,5906
AAIANRLLRRTATHL	PUTATIVE CYCLOPROPANE-FATTY-ACYL-PHOSPHOLIPID SYNTHASE UFAA1 NP_854118	0,2259		QRVAVRVSQLHAGPR	CONSERVED HYPOTHETICAL PROTEIN CAB08634	0,585
WPVYLSTKNTILKAY	CAA17111.1; PROBABLE ISOCITRATE DEHYDROGENASE	0,2243		TRYSSAYELEGAVKR	SECRETED L-ALANINE DEHYDROGENASE ALD CAA15575	0,5797
EVQIGKFRLVFLTGP	CAB01474.1; CONSERVED HYPOTHETICAL PROTEIN CFP17	0,2181		WSVKVPRRLKELHLR	ALTERNATE RNA POLYMERASE SIGMA FACTOR SIGF CAB07069	0,5761
YGSFRRSFRLPAHVT	CAA17343.1; HEAT SHOCK PROTEIN HSP	0,2164		AATIKRFADRFARFN	MYCOBACTERIUM BOVIS ACYL-COA SYNTHASE GENE; U75685	0,5613
IEYRSLARVYGAGTP	MYCOBACTERIUM BOVIS MYCOCEROSIC ACID SYNTHASE GENE; M95808	0,208		DAARVICRRSKLMTR	MYCOBACTERIUM BOVIS MYCOCEROSIC ACID SYNTHASE GENE; M95808	0,5602
RDFARASFSYGLNAK	CAA17111.1; PROBABLE ISOCITRATE DEHYDROGENASE	0,2078		VYVTSEGMYGRVVKL	TRANSMEMBRANE SERINE/THREONINE-PROTEIN KINASE D PKND NP_215446	0,5358
IKRFADRFARFNLQE	MYCOBACTERIUM BOVIS ACYL-COA SYNTHASE GENE; U75685	0,2071		LLEQAAAVEEASDTA	CAE55371.1; PPE FAMILY PROTEIN	0,534
LKIAQGRLFYNTRTL	POSSIBLE GLYCOSYL TRANSFERASE CAB05418	0,1868		FYRRGLFGTIGPYNL	POSSIBLE GLYCOSYL TRANSFERASE CAB05419	0,5246
DGRTLREIRYGSFRR	CAA17343.1; HEAT SHOCK PROTEIN HSP	0,1839		SQMHVSRLLAKSLAR	ALTERNATE RNA POLYMERASE SIGMA FACTOR SIGF CAB07069	0,5245
VGDPDEHSLLAGREQ	MYCOBACTERIUM BOVIS MYCOCEROSIC ACID SYNTHASE GENE; M95808	0,1807		CIRAAARGAHIRSVT	PUTATIVE CYCLOPROPANE-FATTY-ACYL-PHOSPHOLIPID SYNTHASE UFAA1 NP_854118	0,5159
FTAHTKHYRVVSKPA	CAE55335.1; PE FAMILY PROTEIN	0,1789		ERCGVEVTRAKVRRE	RNA POLYMERASE BETA'-SUBUNIT AAA21417	0,5031
KADVYGNTRLGLFPF	MYCOBACTERIUM BOVIS MYCOCEROSIC ACID SYNTHASE GENE; M95808	0,1781		GGATSLISYMLPRSP	MYCOBACTERIUM BOVIS ACYL-COA SYNTHASE GENE; U75685	0,5025
LRLMPENRPRTWLAS	POSSIBLE GLYCOSYL TRANSFERASE CAB05415	0,1776		AGAVMQVISRSEYRQ	POSSIBLE GLYCOSYL TRANSFERASE CAB05415	0,4996
GPMVRKGTMKSQPWI	MTB81 (publication)	0,1774		VKGAWVLARYDDGRP	CONSERVED	0,499

					HYPOTHETICAL PROTEIN TB18.5CAD93033	
WDLTSAEFTRRRYAE	CONSERVED HYPOTHETICAL PROTEIN CAB08634	0,1748		LFIHRPDALARRIGR	PUTATIVE CYCLOPROPANE-FATTY-ACYL-PHOSPHOLIPID SYNTHASE UFAA1 NP_854118	0,4989
FFGVRPVSILYRLYR	POSSIBLE GLYCOSYL TRANSFERASE CAB05418	0,1699		TELAAAQRRKIDRLL	PUTATIVE CYCLOPROPANE-FATTY-ACYL-PHOSPHOLIPID SYNTHASE UFAA1 NP_854118	0,495
ANRLLRRTATHLPLR	PUTATIVE CYCLOPROPANE-FATTY-ACYL-PHOSPHOLIPID SYNTHASE UFAA1 NP_854118	0,1695		NYRYAINNSFGFGGH	3-OXOACYL-[ACYL-CARRIER PROTEIN] SYNTHASE 2 KASB CAA94642	0,4885
FACDPRFNKLLGPLP	POSSIBLE GLYCOSYL TRANSFERASE CAB05418	0,1662		RLARQRVAAAGFGHR	PUTATIVE CYCLOPROPANE-FATTY-ACYL-PHOSPHOLIPID SYNTHASE UFAA1 NP_854118	0,4731
EIGKADVYGNTRLGL	MYCOBACTERIUM BOVIS MYCOCEROSIC ACID SYNTHASE GENE; M95808	0,1651		RRERMGHIELAAPVT	RNA POLYMERASE BETA'-SUBUNIT AAA21417	0,473
VRPFVLARSLDPSRY	POSSIBLE GLYCOSYL TRANSFERASE CAB05418	0,1648		RSEYRQAAARLADAF	POSSIBLE GLYCOSYL TRANSFERASE CAB05415	0,4708
RMRKFARSNHGTCAN	RNA POLYMERASE BETA-SUBUNIT AAA21416	0,1633		DIILNTHGVPRRMNI	RNA POLYMERASE BETA-SUBUNIT AAA21416	0,4596
MGCRLPGGINSPDKL	MYCOBACTERIUM BOVIS MYCOCEROSIC ACID SYNTHASE GENE; M95808	0,1605		KAVKEYARKHPHSMG	PROBABLE ISOCITRATE DEHYDROGENASE CAA16247	0,4521
YCGKYKRVRFKGIIC	RNA POLYMERASE BETA'-SUBUNIT AAA21417	0,1555		RKYIAADRKILNEIA	POSSIBLE GLYCOSYL TRANSFERASE CAB05418	0,4468
ASDFAYLVDFGIARA	TRANSMEMBRANE SERINE/THREONINE-PROTEIN KINASE D PKND NP_215446	0,1554		NRAAAALMAKLRGAA	TWO COMPONENT TRANSCRIPTIONAL REGULATORY PROTEIN DEVR NP_217649	0,4113
TIRVGSFRGRWLDPR	CAA16102.1; ESAT-6 LIKE PROTEIN ESXQ	0,1474		RVGRYKVNKKLGLHV	RNA POLYMERASE BETA-SUBUNIT AAA21416	0,4071
SRTGSIYIVKPKMHG	MTB81 (publication)	0,1458		RLVKLLYRLERPLLF	POSSIBLE GLYCOSYL TRANSFERASE CAB05415	0,404
VAITLTWSQLYRRTL	MYCOBACTERIUM BOVIS ACYL-COA SYNTHASE GENE; U75685	0,1403		KGSQQEDEALYTEDR	MTB48 AAK31576	0,4011
GAAFGGLTTAHTPRR	MYCOBACTERIUM BOVIS MYCOCEROSIC ACID SYNTHASE GENE; M95808	0,1389		WDLTSAEFTRRRYAE	CONSERVED HYPOTHETICAL PROTEIN CAB08634	0,3953

DLVRRSPWGEMFAST	MYCOBACTERIUM BOVIS MYCOCEROSIC ACID SYNTHASE GENE; M95808	0,1389		RDFARASFSYGLNAK	CAA17111.1; PROBABLE ISOCITRATE DEHYDROGENASE	0,3855
GRAGGGAALGGGGMG	MTB48 AAK31576	0,1355		SFYRPKRPGSYPIVL	PERIPLASMIC PHOSPHATE-BINDING LIPOPROTEIN PSTS3 YP_177768	0,3787
YVAWMSVTAGQAELT	CAE55371.1; PPE FAMILY PROTEIN	0,1354		MTSAGGLLEQAAAVE	CAE55371.1; PPE FAMILY PROTEIN	0,3707
TILKAYDGMFKDEFE	CAA17111.1; PROBABLE ISOCITRATE DEHYDROGENASE	0,1256		DSGFVTDGKMFIIGR	MYCOBACTERIUM BOVIS ACYL-COA SYNTHASE GENE; U75685	0,3603
AAAQFNASPVAQSYL	LOW MOLECULAR WEIGHT T-CELL ANTIGEN TB8.4 NP_215690	0,1232		GPMVRKGTMKSQPWI	MTB81 (publication)	0,3553
PPSLVAANRSRLMSL	PPE FAMILY PROTEIN CAE55334	0,1203		WPVYLSTKNTILKAY	CAA17111.1; PROBABLE ISOCITRATE DEHYDROGENASE	0,3529
DPTQQIPKLVANNTR	CAB10044.1; SECRETED ANTIGEN 85-B FBPB (85B)	0,1202		FRHHAIHTIPSERFF	POSSIBLE GLYCOSYL TRANSFERASE CAB05415	0,3521
FGGLTTAHTPRREPR	MYCOBACTERIUM BOVIS MYCOCEROSIC ACID SYNTHASE GENE; M95808	0,1178		VGDPDEHSLLAGREQ	MYCOBACTERIUM BOVIS MYCOCEROSIC ACID SYNTHASE GENE; M95808	0,3507
RAPTESTRRCWTLVS	MYCOBACTERIUM BOVIS MYCOCEROSIC ACID SYNTHASE GENE; M95808	0,1167		RFARFNLQERVIRPS	MYCOBACTERIUM BOVIS ACYL-COA SYNTHASE GENE; U75685	0,3472
AFAMTVPPSLVAANR	PPE FAMILY PROTEIN CAE55334	0,1164		TATLLPFEEAPEMTS	CAE55371.1; PPE FAMILY PROTEIN	0,3462
DARAADILKDESYKV	CAB08889.1; IRON-REGULATED CONSERVED HYPOTHETICAL PROTEIN	0,1123		PSERFFGNLTQGRFY	POSSIBLE GLYCOSYL TRANSFERASE CAB05415	0,3446
SSGLLRHAHCPVVII	HYPOTHETICAL PROTEIN RV2623 NP_217139	0,1105		PSVMADVASGNLPAL	CONSERVED HYPOTHETICAL PROTEIN CAA15739	0,3374
MSVAALAGMRREQPL	MYCOBACTERIUM BOVIS MYCOCEROSIC ACID SYNTHASE GENE; M95808	0,1071		RVTPVAVIGMGCRLP	MYCOBACTERIUM BOVIS MYCOCEROSIC ACID SYNTHASE GENE; M95808	0,3348
RLARQRVAAAGFGHR	PUTATIVE CYCLOPROPANE-FATTY-ACYL-PHOSPHOLIPID SYNTHASE UFAA1 NP_854118	0,1052		IKRFADRFARFNLQE	MYCOBACTERIUM BOVIS ACYL-COA SYNTHASE GENE; U75685	0,3329
RRSGSSRAPTESTRR	MYCOBACTERIUM BOVIS MYCOCEROSIC ACID SYNTHASE GENE; M95808	0,1045		QAAAVEEASDTAAAN	CAE55371.1; PPE FAMILY PROTEIN	0,319
QRVAVRVSQLHAGPR	CONSERVED HYPOTHETICAL PROTEIN CAB08634	0,1039		IAAAAKPPLGSPPPK	CAA17582.1; PROBABLE SERINE PROTEASE PEPD	0,3094
TRHTQTWIQKYIFPG	PUTATIVE CYCLOPROPANE-	0,1036		MMGNAFLTALTNAGI	CAB10947.1; PROBABLE	0,3017

	FATTY-ACYL-PHOSPHOLIPID SYNTHASE UFAA1 NP_854118				LIPOPROTEIN LPRJ	
AEDDCPPPGYDITAL	MYCOBACTERIUM BOVIS MYCOCEROSIC ACID SYNTHASE GENE; M95808	0,1033		NGIELCRDLLSRMPD	TWO COMPONENT TRANSCRIPTIONAL REGULATORY PROTEIN DEVR NP_217649	0,3012
KDTKAVNPESTFSRI	PROBABLE ISOCITRATE DEHYDROGENASE CAA16247	0,1023		RRSGSSRAPTESTRR	MYCOBACTERIUM BOVIS MYCOCEROSIC ACID SYNTHASE GENE; M95808	0,2936
AGAVMQVISRSEYRQ	POSSIBLE GLYCOSYL TRANSFERASE CAB05415	0,0998		FGGLTTAHTPRREPR	MYCOBACTERIUM BOVIS MYCOCEROSIC ACID SYNTHASE GENE; M95808	0,2863
AMSLTVGAGVASADP	LOW MOLECULAR WEIGHT T-CELL ANTIGEN TB8.4 NP_215690	0,0961		DGRTLREIRYGSFRR	CAA17343.1; HEAT SHOCK PROTEIN HSP	0,2843
TELAAAQRRKIDRLL	PUTATIVE CYCLOPROPANE-FATTY-ACYL-PHOSPHOLIPID SYNTHASE UFAA1 NP_854118	0,0959		RHGLSSLGWNLCRIF	POSSIBLE GLYCOSYL TRANSFERASE CAB05415	0,284
DLVYGDVIMRSTNFR	POSSIBLE GLYCOSYL TRANSFERASE CAB05419	0,0952		ATATATLLPFEEAPE	CAE55371.1; PPE FAMILY PROTEIN	0,2801
MIDGTSLRALLKQYG	TRANSMEMBRANE SERINE/THREONINE-PROTEIN KINASE D PKND NP_215446	0,0949		ATVSPAMVAANRTRL	PPE FAMILY PROTEIN CAE55504	0,2754
SADNMREYLAAGAKE	MTB48 AAK31576	0,0933		PLNWLRRKHGLSSLG	POSSIBLE GLYCOSYL TRANSFERASE CAB05418	0,2735
WSQLYRRTLNVAREL	MYCOBACTERIUM BOVIS ACYL-COA SYNTHASE GENE; U75685	0,0842		MAKTIAYDEEARRGL	60 kDa chaperonin 2 P0A521	0,2711
VKKPETINYRTLKPE	RNA POLYMERASE BETA'-SUBUNIT AAA21417	0,0832		YTEPILHRRRREFKA	MTB81 (publication)	0,27
RQRVAAAGFGHRVEI	PUTATIVE CYCLOPROPANE-FATTY-ACYL-PHOSPHOLIPID SYNTHASE UFAA1 NP_854118	0,0809		AGAATEVELKERKHR	60 kDa chaperonin 2 P0A521	0,2637
PVAVIGMGCRLPGGI	MYCOBACTERIUM BOVIS MYCOCEROSIC ACID SYNTHASE GENE; M95808	0,0773		FNIRCFSNPALVTRY	POSSIBLE GLYCOSYL TRANSFERASE CAB05419	0,2446
RVGRYKVNKKLGLHV	RNA POLYMERASE BETA-SUBUNIT AAA21416	0,0751		FGTIGPYNLRYRVLA	POSSIBLE GLYCOSYL TRANSFERASE CAB05419	0,239
RAPKAVKEYARKHPH	PROBABLE ISOCITRATE DEHYDROGENASE CAA16247	0,0748		AERVGISQMHVSRLL	ALTERNATE RNA POLYMERASE SIGMA FACTOR SIGF CAB07069	0,2359
WSPQARRRFPLPDVP	POSSIBLE GLYCOSYL	0,0744		AFAMTVPPSLVAANR	PPE FAMILY PROTEIN CAE55334	0,2342

	TRANSFERASE CAB05418					
PEFQRHRDKIVQRCL	ALTERNATE RNA POLYMERASE SIGMA FACTOR SIGF CAB07069	0,0739		TTLALVSAPAGGRAA	PROBABLE CUTINASE PRECURSOR CFP21 NP_216500	0,2311
EKAIKERYARCLGSA	PROBABLE ISOCITRATE DEHYDROGENASE CAA16247	0,0737		DFTPAAEIVKDGDDA	CAA17343.1; HEAT SHOCK PROTEIN HSP	0,2213
FRGEFTPKTQLTMLS	MCE-FAMILY PROTEIN MCE1AYP_177701	0,0736		AASAAPSIPAANMPP	CAA17582.1; PROBABLE SERINE PROTEASE PEPD	0,2175
MDFGLLPPEVNSSRM	PPE FAMILY PROTEIN CAE55334	0,0725		STGMSGGFVTAPTQG	YP_177963; PPE FAMILY PROTEIN	0,2166
GEAQLGNSSGNFLLP	PERIPLASMIC PHOSPHATE-BINDING LIPOPROTEIN PSTS1 YP_177770	0,0724		INAFSVGSGQTYGVD	CAB09453.1; PROBABLE SERINE PROTEASE PEPA	0,2139
VLAGASLVAVSWAVG	POSSIBLE HEMOLYSIN-LIKE PROTEIN CAA17201	0,0714		WDATATELNNALQNL	CAE55648	0,2136
QNTMKIGIMDEERRT	MTB81 (publication)	0,0685		YGRLRHGQILFTFLH	SECRETED L-ALANINE DEHYDROGENASE ALD CAA15575	0,2054
KYKRVRFKGIICERC	RNA POLYMERASE BETA'-SUBUNIT AAA21417	0,0685		ENPSARDQILPVYAE	MTB48 AAK31576	0,1932
YGRLRHGQILFTFLH	SECRETED L-ALANINE DEHYDROGENASE ALD CAA15575	0,0667		AEAADAIRAMSNAEH	MYCOBACTERIUM BOVIS MYCOCEROSIC ACID SYNTHASE GENE; M95808	0,1922
TGRSLDMSVAALAGM	MYCOBACTERIUM BOVIS MYCOCEROSIC ACID SYNTHASE GENE; M95808	0,0666		TATELNNALQNLART	CAE55648	0,1917
VEYVPSSEVDYMDVS	RNA POLYMERASE BETA-SUBUNIT AAA21416	0,0662		YNQLLGPLPFRHHAI	POSSIBLE GLYCOSYL TRANSFERASE CAB05415	0,1824
IDGPAPDGYPIINYE	PERIPLASMIC PHOSPHATE-BINDING LIPOPROTEIN PSTS1 YP_177770	0,065		DANRALMGANMQRQA	RNA POLYMERASE BETA-SUBUNIT AAA21416	0,1811
GDFYAGEKSMTLDRA	PROBABLE ISOCITRATE DEHYDROGENASE CAA16247	0,0626		MNPDIEKDQTSDEVT	CAB01474.1; CONSERVED HYPOTHETICAL PROTEIN CFP17	0,177
HSLLAGREQVRHLVR	MYCOBACTERIUM BOVIS MYCOCEROSIC ACID SYNTHASE GENE; M95808	0,0603		TILKAYDGMFKDEFE	CAA17111.1; PROBABLE ISOCITRATE DEHYDROGENASE	0,1768
YTEPILHRRRREFKA	MTB81 (publication)	0,0597		WSQLYRRTLNVAREL	MYCOBACTERIUM BOVIS ACYL-COA SYNTHASE GENE; U75685	0,1726
VRGDKVIAYARKFLD	MTB81 (publication)	0,0593		AAGFSGRAQVGKGMW	MTB81 (publication)	0,1691
VIPTMTPPPGMVRQR	CAA17582.1; PROBABLE SERINE PROTEASE PEPD	0,0591		GAAFGGLTTAHTPRR	MYCOBACTERIUM BOVIS MYCOCEROSIC ACID SYNTHASE GENE; M95808	0,1685
DIILNTHGVPRRMNI	RNA POLYMERASE	0,0587		ERNDPTQQIPKLVAN	CAB10044.1;	0,1631

	BETA-SUBUNIT AAA21416				SECRETED ANTIGEN 85-B FBPB (85B)	
DLATGTWLLFLGADD	POSSIBLE GLYCOSYL TRANSFERASE CAB05419	0,0571		ANRLLRRTATHLPLR	PUTATIVE CYCLOPROPANE-FATTY-ACYL-PHOSPHOLIPID SYNTHASE UFAA1 NP_854118	0,1621
DVILESAITTIMDFE	MTB81 (publication)	0,0561		LDRLLFKRNICHQAI	POSSIBLE GLYCOSYL TRANSFERASE CAB05419	0,1618
GRGGYAGMLVGSVGE	HYPOTHETICAL PROTEIN RV2623 NP_217139	0,0558		GVVLAGGESRRMGRD	CAA16030.1; PROBABLE MOLYBDOPTERIN-GUANINE DINUCLEOTIDE BIOSYNTHESIS PROTEIN	0,1615
MAKTIAYDEEARRGL	60 kDa chaperonin 2 P0A521	0,0544		RAPTESTRRCWTLVS	MYCOBACTERIUM BOVIS MYCOCEROSIC ACID SYNTHASE GENE; M95808	0,1573
GLRGLLRVISSEHPM	MYCOBACTERIUM BOVIS MYCOCEROSIC ACID SYNTHASE GENE; M95808	0,0526		NNKAALEPVNPPKPP	MTB48 AAK31576	0,1496
GLGLFFASKLAAAGC	MYCOBACTERIUM BOVIS MYCOCEROSIC ACID SYNTHASE GENE; M95808	0,0511		VAAMFGYHSAASAVA	PPE FAMILY PROTEIN CAE55504	0,1483
EAAAARSAVVLCNGG	POSSIBLE GLYCOSYL TRANSFERASE CAB05418	0,0484		DVTVSRRHAEFRLEN	CAB01474.1; CONSERVED HYPOTHETICAL PROTEIN CFP17	0,1367
TESTRRCWTLVSSRS	MYCOBACTERIUM BOVIS MYCOCEROSIC ACID SYNTHASE GENE; M95808	0,047		VGNGYWQKPDESERT	MYCOBACTERIUM BOVIS ACYL-COA SYNTHASE GENE; U75685	0,1357
GGQFTLPGRSLMFVR	MTB81 (publication)	0,0468		TPGTGMPAAPMVPPT	MTB48 AAK31576	0,128
PTTYDHPTFAVHDTL	SECRETED L-ALANINE DEHYDROGENASE ALD CAA15575	0,0461		WSPQARRRFPLPDVP	POSSIBLE GLYCOSYL TRANSFERASE CAB05418	0,1277
TIKAERTEQKDFDGR	CAA17245.1; HEAT SHOCK PROTEIN HSPX	0,0436		FRGEFTPKTQLTMLS	MCE-FAMILY PROTEIN MCE1AYP_177701	0,1248
ANRTRLASLVAANLL	PPE FAMILY PROTEIN CAE55504	0,0417		AMAAQLQAVPGAAQY	LOW MOLECULAR WEIGHT T-CELL ANTIGEN TB8.4 NP_215690	0,1226
HDEDSVMPHPQQAPV	HYPOTHETICAL PROTEIN RV2623 NP_217139	0,0413		VRPFVLARSLDPSRY	POSSIBLE GLYCOSYL TRANSFERASE CAB05418	0,1202
IAKEIELEDPYEKIG	60 kDa chaperonin 2 P0A521	0,041		FKEMTLLELSDFVKK	CAB07109.1; PROBABLE 50S RIBOSOMAL PROTEIN L7/L12 RPLL	0,1107
GDGGVSGFGNFGAGS	YP_177963; PPE FAMILY PROTEIN	0,0408		AAPSIPAANMPPGSV	CAA17582.1; PROBABLE SERINE PROTEASE PEPD	0,1072
EFEGELPRLFVVTRQ	MYCOBACTERIUM BOVIS	0,0399		YPADPKTDQEKAIKE	PROBABLE ISOCITRATE	0,0996

	MYCOCEROSIC ACID SYNTHASE GENE; M95808				DEHYDROGENASE CAA16247	
GGATSLISYMLPRSP	MYCOBACTERIUM BOVIS ACYL-COA SYNTHASE GENE; U75685	0,0382		SQFGPYQLLRLLGRG	TRANSMEMBRANE SERINE/THREONINE-PROTEIN KINASE D PKND NP_215446	0,095
RCLPLADHIARRFEG	ALTERNATE RNA POLYMERASE SIGMA FACTOR SIGF CAB07069	0,0376		DVVVLLESMKMEIPV	CAB08316.1; BIOTINYLATED PROTEIN TB7.3	0,0858
VAAMFGYHSAASAVA	PPE FAMILY PROTEIN CAE55504	0,0371		YQAGKPTSTNPIASI	CAA17111.1; PROBABLE ISOCITRATE DEHYDROGENASE	0,0794
LTAANTKKVAMAAPM	POSSIBLE GLYCOSYL TRANSFERASE CAB05419	0,0368		SYNTGSFNVGDTNTG	YP_177963; PPE FAMILY PROTEIN	0,0779
GNRGDANIGIGNIGD	PPE FAMILY PROTEIN CAE55504	0,0363		ADDEGYPKRARMLMD	CONSERVED HYPOTHETICAL PROTEIN TB16.3CAD97060	0,0626
TLTWSQLYRRTLNVA	MYCOBACTERIUM BOVIS ACYL-COA SYNTHASE GENE; U75685	0,0341		SSGLLRHAHCPVVII	HYPOTHETICAL PROTEIN RV2623 NP_217139	0,0544
SPAMVAANRTRLASL	PPE FAMILY PROTEIN CAE55504	0,0339		PDSDMTTAVMRPSKT	PROBABLE ISOCITRATE DEHYDROGENASE CAA16247	0,045
ERNDPTQQIPKLVAN	CAB10044.1; SECRETED ANTIGEN 85-B FBPB (85B)	0,0337		NELVRVYVAQKRKIS	RNA POLYMERASE BETA-SUBUNIT AAA21416	0,0405
AEFPDYLTEEQRVPD	PROBABLE ISOCITRATE DEHYDROGENASE CAA16247	0,0334		VPPPVIAENRAELMI	CAE55371.1; PPE FAMILY PROTEIN	0,0383
PKVISRAMVMRTVIS	POSSIBLE GLYCOSYL TRANSFERASE CAB05419	0,0334		LPDFLGGFAGLPSLG	CAB06237.1; CONSERVED HYPOTHETICAL PROTEIN	0,0381
EGYPKRARMLMDAAI	CONSERVED HYPOTHETICAL PROTEIN TB16.3CAD97060	0,0327		EAVGYRSWPRYFAAL	PUTATIVE CYCLOPROPANE-FATTY-ACYL-PHOSPHOLIPID SYNTHASE UFAA1 NP_854118	0,0362
LYLTIMTLMNATNLR	CAB06237.1; CONSERVED HYPOTHETICAL PROTEIN	0,0318		TIRVGSFRGRWLDPR	CAA16102.1; ESAT-6 LIKE PROTEIN ESXQ	0,0323
RVGFPQHVENALRLM	POSSIBLE GLYCOSYL TRANSFERASE CAB05415	0,0312		KADVYGNTRLGLFPF	MYCOBACTERIUM BOVIS MYCOCEROSIC ACID SYNTHASE GENE; M95808	0,0238
EGVARVLTKPRFRGW	POSSIBLE HEMOLYSIN-LIKE PROTEIN CAA17201	0,0306		LDEHGTVLLAVRGLR	MYCOBACTERIUM BOVIS MYCOCEROSIC ACID SYNTHASE GENE; M95808	0,0238
NELVRVYVAQKRKIS	RNA POLYMERASE BETA-SUBUNIT AAA21416	0,03		YELEGAVKRADLVIG	SECRETED L-ALANINE DEHYDROGENASE ALD CAA15575	0,0127
TLAKIFNGSITQWNN	PERIPLASMIC PHOSPHATE-BINDING	0,0295		NSGVSMTNTLSSMLK	CAE55371.1; PPE FAMILY PROTEIN	0,0126

	LIPOPROTEIN PSTS3 YP_177768					
VIGVAGNLDQHLNME	POSSIBLE GLYCOSYL TRANSFERASE CAB05415	0,0293		FRSGYLDVYQWTLIR	PUTATIVE CYCLOPROPANE-FATTY-ACYL-PHOSPHOLIPID SYNTHASE UFAA1 NP_854118	0,0088
VYVTSEGMYGRVVKL	TRANSMEMBRANE SERINE/THREONINE-PROTEIN KINASE D PKND NP_215446	0,0282		VVAVGPGRWDEDGEK	CAB01005.1; 10 KDA CHAPERONIN GROES	0,0012
GLFNSGSFNTGIGNS	YP_177963; PPE FAMILY PROTEIN	0,0253		VEYVPSSEVDYMDVS	RNA POLYMERASE BETA-SUBUNIT AAA21416	-0,0021
LFNVGGFNTGVANVG	YP_177963; PPE FAMILY PROTEIN	0,0246		GKSPGFGTTVDFPAV	PERIPLASMIC PHOSPHATE-BINDING LIPOPROTEIN PSTS1 YP_177770	-0,0087
DGVGALLRYAATNRL	CONSERVED HYPOTHETICAL PROTEIN CAB08634	0,0241		TRIEAVPIAGFAQMR	3-OXOACYL-[ACYL-CARRIER PROTEIN] SYNTHASE 2 KASB CAA94642	-0,0095
RFARFNLQERVIRPS	MYCOBACTERIUM BOVIS ACYL-COA SYNTHASE GENE; U75685	0,0232		VGISQMHVSRLLAKS	ALTERNATE RNA POLYMERASE SIGMA FACTOR SIGF CAB07069	-0,0119
GGLNSGTGNIGLFNS	YP_177963; PPE FAMILY PROTEIN	0,0221		SADNMREYLAAGAKE	MTB48 AAK31576	-0,0198
LDRLLFKRNICHQAI	POSSIBLE GLYCOSYL TRANSFERASE CAB05419	0,0219		AEFPDYLTEEQRVPD	PROBABLE ISOCITRATE DEHYDROGENASE CAA16247	-0,0231
NANIGFGNRGDANIG	PPE FAMILY PROTEIN CAE55504	0,0201		VAASDLAYTLARGRR	MYCOBACTERIUM BOVIS MYCOCEROSIC ACID SYNTHASE GENE; M95808	-0,0241
PPQEQGLIPGFLMPP	MTB48 AAK31576	0,0196		FTLPGERIHVVFRSD	PERIPLASMIC PHOSPHATE-BINDING LIPOPROTEIN PSTS3 YP_177768	-0,0268
MLVTAVVLLCCSGVA	CAA98382.1; IMMUNOGENIC PROTEIN MPT64	0,0196		SKLMTRIAGAGAMGS	MYCOBACTERIUM BOVIS MYCOCEROSIC ACID SYNTHASE GENE; M95808	-0,0279
SRPQSSSNNSVPGAP	RNA POLYMERASE BETA-SUBUNIT AAA21416	0,0171		DMEGTFARPMTELVT	3-OXOACYL-[ACYL-CARRIER PROTEIN] SYNTHASE 2 KASB CAA94642	-0,0285
DMEGTFARPMTELVT	3-OXOACYL-[ACYL-CARRIER PROTEIN] SYNTHASE 2 KASB CAA94642	0,0169		APEMTSAGGLLEQAA	CAE55371.1; PPE FAMILY PROTEIN	-0,0288
TGNLGLFNSGTGNIG	YP_177963; PPE FAMILY PROTEIN	0,0167		TMPHHRMLATRHTQT	PUTATIVE CYCLOPROPANE-FATTY-ACYL-PHOSPHOLIPID SYNTHASE UFAA1 NP_854118	-0,0291
MSGQADTATTAEART	POSSIBLE HEMOLYSIN-LIKE PROTEIN CAA17201	0,0157		ILTVSVAVSEGKPTE	CAA17245.1; HEAT SHOCK PROTEIN HSPX	-0,03
VNIGASDAYLSEGDM	PERIPLASMIC PHOSPHATE-BINDING	0,0146		VRDLIARWEQRDVMA	MYCOBACTERIUM BOVIS MYCOCEROSIC ACID	-0,031

	LIPOPROTEIN PSTS1 YP_177770				SYNTHASE GENE; M95808	
SAITDADFKAAGAQL	SECRETED L-ALANINE DEHYDROGENASE ALD CAA15575	0,0142		YVAWMSVTAGQAELT	CAE55371.1; PPE FAMILY PROTEIN	-0,0315
NNSFGFGGHNVAIAF	3-OXOACYL-[ACYL-CARRIER PROTEIN] SYNTHASE 2 KASB CAA94642	0,0138		RMTSGSSSGFKVRPS	YP_177963; PPE FAMILY PROTEIN	-0,0345
GGNLGGQNLGLGNLG	YP_177963; PPE FAMILY PROTEIN	0,0129		QRHRDKIVQRCLPLA	ALTERNATE RNA POLYMERASE SIGMA FACTOR SIGF CAB07069	-0,0419
VGASGNNGTSALLQT	PERIPLASMIC PHOSPHATE-BINDING LIPOPROTEIN PSTS2 YP_177769	0,0127		DAQSIQAAAAGFASK	PERIPLASMIC PHOSPHATE-BINDING LIPOPROTEIN PSTS1 YP_177770	-0,0453
QLATWVEEHQDCVAA	MYCOBACTERIUM BOVIS MYCOCEROSIC ACID SYNTHASE GENE; M95808	0,0122		ANRTRLASLVAANLL	PPE FAMILY PROTEIN CAE55504	-0,0461
FNSGDGGVSGFGNFG	YP_177963; PPE FAMILY PROTEIN	0,0115		RYARCLGSAVNPVLR	PROBABLE ISOCITRATE DEHYDROGENASE CAA16247	-0,0467
NNQTVLPFDGLNYPE	TRANSMEMBRANE SERINE/THREONINE-PROTEIN KINASE D PKND NP_215446	0,0113		ELAIPMSGIAMAVVG	CAB06237.1; CONSERVED HYPOTHETICAL PROTEIN	-0,0479
VEQAALQSAWQGDTG	CAA17363.1; LOW MOLECULAR WEIGHT PROTEIN ANTIGEN 7 ESXH	0,0106		TSYTSDEAMLDAILA	TWO COMPONENT TRANSCRIPTIONAL REGULATORY PROTEIN DEVR NP_217649	-0,0482
ATATATLLPFEEAPE	CAE55371.1; PPE FAMILY PROTEIN	0,0102		QNTMKIGIMDEERRT	MTB81 (publication)	-0,0484
AMNRGVDLATGTWLL	POSSIBLE GLYCOSYL TRANSFERASE CAB05419	0,01		PLSGLTHKRRLSALG	RNA POLYMERASE BETA-SUBUNIT AAA21416	-0,0499
NALFAGPAKMLRLNA	YP_177963; PPE FAMILY PROTEIN	0,01		TESTRRCWTLVSSRS	MYCOBACTERIUM BOVIS MYCOCEROSIC ACID SYNTHASE GENE; M95808	-0,0518
AFLAAQELILSGAQQ	MTB81 (publication)	0,0096		AAAQFNASPVAQSYL	LOW MOLECULAR WEIGHT T-CELL ANTIGEN TB8.4 NP_215690	-0,0531
DSALLAAAGFGNTTA	MCE-FAMILY PROTEIN MCE1AYP_177701	0,009		EGVARVLTKPRFRGW	POSSIBLE HEMOLYSIN-LIKE PROTEIN CAA17201	-0,0553
GSTAQANAMTRFVNV	PERIPLASMIC PHOSPHATE-BINDING LIPOPROTEIN PSTS3 YP_177768	0,0085		ILVQANEAETTTASG	CAB01005.1; 10 KDA CHAPERONIN GROES	-0,0559
LKMCWPAAPRSVGVP	POSSIBLE HEMOLYSIN-LIKE PROTEIN CAA17201	0,0085		GAPNRVSFAKLREPL	RNA POLYMERASE BETA-SUBUNIT AAA21416	-0,0561
GKLKDTKAVNPESTF	PROBABLE ISOCITRATE DEHYDROGENASE CAA16247	0,0076		VRPVSILYRLYRPLI	POSSIBLE GLYCOSYL TRANSFERASE CAB05418	-0,0571
RVAGAYKAPAETQAQ		0,0075		TRHTQTWIQKYIFPG		-0,0574
QGYGSLGLMTSVLMT		0,0074		DVDKDVNVELDPGQP		-0,0588

DAFPNEFPDPTISVQ		0,0064		GLGLFFASKLAAAGC		-0,0622
VKAPGFGDRRKAMLQ		0,0053		YPEGLAVDTQGAVYV		-0,0637
AARSAVVLCNGGSPT		0,0048		PIPEGLTTGRSCQTD		-0,0647
DANRALMGANMQRQA		0,0047		RVSVGNLRIARVLYD		-0,0674
YTSGSTRTPAGVVMS		0,0042		TWKLLLDRQSGIRTL		-0,0678
VVAVGPGRWDEDGEK		0,0027		LERPVGDSSDCGTIR		-0,0698
AAGFSGRAQVGKGMW		0,0026		TGNLGLFNSGTGNIG		-0,072
QKVQAAGNNMAQTDS		0,0018		TGMELRAAIDAATSS		-0,073
ARTISEAGQAMASTE		-0,0013		GRAGGGAALGGGGMG		-0,0739
VGAGERKMSALVDAS		-0,0013		PLDDGDVIDSMFMSK		-0,0752
PGEPPTKESAQTLLE		-0,0017		ARDADMLSELGEPVD		-0,0787
FVTDGKMFIIGRIKD		-0,0018		RVQAATIKRFADRFA		-0,0789
VGSWIGSSAGLMVAA		-0,0023		YQQRSEKVLTEYNNK		-0,0794
KIHARSTGPYSMITQ		-0,0029		PRREPRRCWPRSRCP		-0,0801
CAPVFVMAAPGQPLP		-0,0031		FTAHTKHYRVVSKPA		-0,0829
AERVGISQMHVSRLL		-0,0043		AGNLGLFNSGTGNIG		-0,0878
QEITRGRCAAISVPG		-0,0044		RKIPLTLVHAVSPEV		-0,0882
YELEGAVKRADLVIG		-0,0049		TRGRCAAISVPGDRR		-0,0902
SRMYSGPGPESMLAA		-0,0052		FVTDGKMFIIGRIKD		-0,0914
FAGYTGAAESPTSVL		-0,0055		NRANEVEAPMADPPT		-0,0921
DSGFVTDGKMFIIGR		-0,0077		VGAQNLGAANAGSGN		-0,0981
LEKDNTVGTDEALLD		-0,0084		LVDFGIARAASDPGL		-0,1036
PNGYTFKEDEYPSTA		-0,0089		AAERGPGQMLGGLPV		-0,105
NYNLPGVSEHLKLNG		-0,0098		ASGLVIPDTAKEKPQ		-0,1059
GAGTVYVTDFNNRVV		-0,0102		PGAARWSPGDSATVA		-0,1066
SGMTSQAATFAANRL		-0,0118		LLKGAKEVETKEQIA		-0,1082
WDGVAITLTWSQLYR		-0,012		HSLLAGREQVRHLVR		-0,1083
AHMKPGAVLVDIAID		-0,0124		PLAGVVLAGGESRRM		-0,1106
DAARVICRRSKLMTR		-0,0127		NTMAMMARDTAEAAK		-0,1117
WDATATELNNALQNL		-0,0129		SSDLRVGQPVLAIGS		-0,1136
KIDPPPPPQEQGLIP		-0,0131		PELVPTYNLPANHRY		-0,1203
AMRTLRKYVEADLRV		-0,0144		EIGKADVYGNTRLGL		-0,1232
VAPVSPSTASSRTDT		-0,0144		LLRYAATNRLGSHRS		-0,1234
VLSACGNDDNVTGGG		-0,015		MLEGCILADSRQSKT		-0,1262
QTYGVDVVGYDRTQD		-0,0151		MEAGPMVRKGTMKSQ		-0,1268
TGQHTGLRIVDAASL		-0,0153		AGVLLRTERLKSQRV		-0,1296
VAASDLAYTLARGRR		-0,0153		VSEHLKLNGKVLAAM		-0,132
VDGAPINSATAMADA		-0,0154		TGDEVTYRADIYALA		-0,1361
LVVGNGGPGVTALVM		-0,0159		FFGVRPVSILYRLYR		-0,1365
GQRCAPVFVMAAPGQ		-0,016		NSTELPSSGFFNTGG		-0,1409
SPSRKTGELDNRGSQ		-0,0163		SEAIAASYDAGVLTV		-0,1433
DVTVSRRHAEFRLEN		-0,0164		AQAASLEAEHQAIVR		-0,1444
QAAAVEEASDTAAAN		-0,0168		VNIGASDAYLSEGDM		-0,1456
QNAASIAGLFLTTEA		-0,0169		QTYGVDVVGYDRTQD		-0,1478
ALGDNWIVGASNSTG		-0,0172		TTVALGRSVCPMVVA		-0,1489
ERCGVEVTRAKVRRE		-0,0172		VFRARMQREADTAGR		-0,1505
NNKAALEPVNPPKPP		-0,0174		DNQSGIGGLNSGTGN		-0,1507
EFRLENNEFNVVDVG		-0,0179		LSSMLKGFAPAAAAQ		-0,1519
EIELEDPYEKIGAEL		-0,0182		NALFAGPAKMLRLNA		-0,1523
FGKPANPDVLVVGNG		-0,0183		LRISSQLLANWLRHG		-0,1594
APAQANPVDDAFIAA		-0,0187		QLAEHFASLADVLTK		-0,1596
APSLFIHRPDALARR		-0,02		QPVVLAAATGDLPTI		-0,1604
LNSLTYDVSVAQGVT		-0,0205		DSVAAVDAADKVLGY		-0,1628
IAAAAKPPLGSPPPK		-0,0206		LIPGFLMPPSDGSGV		-0,1634
NGTPNELGGANIPAE		-0,0206		TLTWSQLYRRTLNVA		-0,1657
MVDFGALPPEINSAR		-0,0221		GPYLQRGVADLVPTA		-0,1676
AAGTAAQAAVVRFQE		-0,0226		KDTKAVNPESTFSRI		-0,1685
HSTVLVAPVSPSTAS		-0,0228		EFRLENNEFNVVDVG		-0,1718
SGFFNSGDGGVSGFG		-0,0231		YVAQKRKISDGDKLA		-0,174
GADGKLKDTKAVNPE		-0,0231		TGMPAAPMVPPTGSP		-0,1747
DVDKDVNVELDPGQP		-0,0232		HDEDSVMPHPQQAPV		-0,1762
DKGDVVVLLESMKME		-0,0237		EIVKDGDDAVVRLEL		-0,1775
AHVVRPFALAQSLDP		-0,0242		TAAQNGVRAMSSLGS		-0,1784
SMTNSGVSMTNTLSS		-0,0242		PAVFAVQVALAATME		-0,179
GNIGLGNAGSGNVGF		-0,0243		EKAIKERYARCLGSA		-0,1812
CIRAAARGAHIRSVT		-0,0244		RNFLAAPPPQRAAMA		-0,1822

TANGSGAGISEFNGN		-0,0246		LGGQNLGLGNLGDGN		-0,1837
PTQYRQPYEALGGTR		-0,0252		HEGQTTMTVPGGVEV		-0,1873
NVTGGGATTGQASAK		-0,0255		DLARVGRYKVNKKLG		-0,1902
DAQSIQAAAAGFASK		-0,0265		GQARLAASAFEATLA		-0,1934
NGTSALLQTTDGSIT		-0,0271		LKIAQGRLFYNTRTL		-0,1937
KRADHSMIFVFIAGS		-0,0281		KGAGKSFQGGVGEGA		-0,1942
AHTPRREPRRCWPRS		-0,0282		GTAVSARNTAVCAPT		-0,1972
LVDAADPEVVFVSGE		-0,0283		PILDDLAAALADIAP		-0,1978
GLPQNTMKIGIMDEE		-0,0287		AHTPRREPRRCWPRS		-0,1988
DYMDVSPRQMVSVAT		-0,0289		NPGELLPEAAGPTQV		-0,1989
AESNNQVVLPFTDIT		-0,0291		MVDFGALPPEINSAR		-0,2008
AVLDPDHDGMRVQVR		-0,0293		WDGVAITLTWSQLYR		-0,201
ELTRVLTVLAGSVDE		-0,0296		FGVRLVKLLYRLERP		-0,2049
LVAANRSRLMSLVAA		-0,03		IEYRSLARVYGAGTP		-0,2061
RSNPALAKGLSTHEG		-0,0304		RVVRVTGRASARGLI		-0,2062
RVVRVTGRASARGLI		-0,0307		VGADEDDIKATYDKG		-0,2072
DSVAAVDAADKVLGY		-0,0314		APSLFIHRPDALARR		-0,2087
NVVDVGSLNGTYVNR		-0,0316		GLFNSGSFNTGIGNS		-0,2096
MEAGPMVRKGTMKSQ		-0,0322		HEHRPELAMVDSARG		-0,2098
GQARLAASAFEATLA		-0,0322		YPITGKLGSELTMTD		-0,2108
VGAGTGIVIDPNGVV		-0,0325		GLNYPEGLAVDTQGA		-0,2116
MMGNAFLTALTNAGI		-0,0329		AHLRPGLDELSFTLT		-0,2124
ADPGEVMKAIADIEA		-0,033		AGSWLLIDTSNAVDT		-0,2127
SDGTATGAADPRAPS		-0,0331		GVDVVGYDRTQDVAV		-0,2128
ARQTCGDFELVLVDG		-0,0334		EPVVAMGNSGGQGGT		-0,214
SYNTGSFNVGDTNTG		-0,0336		AENRAELMILIATNL		-0,2146
GLGDKFGESIVNANT		-0,0342		EFPAIPGFPALPGLP		-0,2165
PDTISGATIPQGEQS		-0,0344		MDFGLLPPEVNSSRM		-0,2173
TILRRGDNATLLATP		-0,0348		PFNVNLKLQFLHDAF		-0,2216
RGGPGGAPGCWQPIT		-0,0348		ALGQTVQASDSLTGA		-0,2222
TSSSQESGVIEEVSA		-0,0349		APTTGVLPPAANDVS		-0,2238
AQAASLEAEHQAIVR		-0,0353		TSSSQESGVIEEVSA		-0,2249
PDEHSLLAGREQVRH		-0,0355		ITTTAGPQLVVPVLN		-0,2272
GLGNAGSSNYGLANL		-0,0356		NRVSFAKLREPLEVP		-0,2275
WGGPPVASEVRGDAA		-0,036		EIELEDPYEKIGAEL		-0,2279
KLRPGEPPTKESAQT		-0,0365		ARWSPGDSATVAGPL		-0,2296
LGIGNIGLGNAGSTN		-0,037		LPDIIDGLWPPKLPD		-0,2312
LLRYAATNRLGSHRS		-0,0371		LPQWLSANRAVKPTG		-0,232
VRDLIARWEQRDVMA		-0,0371		AVCAPTTGVLPPAAN		-0,2324
GITGNGQIGFGKPAN		-0,0377		PAATIDQLKTDAKLL		-0,236
DFAGSDVPLNPSTGQ		-0,0378		DDLMVNNAGLVCGGV		-0,2369
LLEQAAAVEEASDTA		-0,0388		SLIGLAMGDAGGYKA		-0,2375
EDYLVLTTTAGGLAS		-0,0392		LVAANRSRLMSLVAA		-0,2414
QSGLSIVMPVGGQSS		-0,0402		EAVIGNRRRQDSKES		-0,2421
PRREPRRCWPRSRCP		-0,0402		PAVPGALGENGNGGM		-0,2496
GLGGLGLFFASKLAA		-0,041		IRGTFKSVAVKAPGF		-0,2509
DGPTLAKIFNGSITQ		-0,041		GLYNTGGLPPGTPAV		-0,2514
NNAVGAGTGIVIDPN		-0,0415		YTSGSTRTPAGVVMS		-0,2519
ATLAHVVRPFVLARS		-0,0416		LLKQYGPLTPARAVA		-0,2531
NAGSSNYGLANLGVG		-0,0417		QAIFYRRGLFGTIGP		-0,2543
ERLIIHRDTDQGVYD		-0,0422		WLLIDTSNAVDTPDM		-0,2552
AATQAAGAGAVADAQ		-0,0423		LEGTYRLAPKGSGTE		-0,2553
FAGAGLGPMLAAASA		-0,0432		IKGMELARAVKDVGA		-0,2591
AANMPPGSVEQVAAK		-0,0434		HGMLTDRDIVIKGLA		-0,2598
VFAPTNAAFDKLPAA		-0,0434		GIRLTPKVIATNKTA		-0,2609
GDVITAVDGAPINSA		-0,0435		GLGGLGLFFASKLAA		-0,2621
GSGAGISEFNGNQTD		-0,0437		RRLKELHLRLGTATA		-0,2621
VQAKPAAAASLAAIA		-0,044		TILRRGDNATLLATP		-0,2621
TSVLMTADGKTVEAE		-0,0446		LIATNLLGQNTPAIA		-0,2657
ARMQREADTAGRLTE		-0,0459		TPADGSAPIVHEMVS		-0,2658
ADDEGYPKRARMLMD		-0,0459		AARSAVVLCNGGSPT		-0,2658
TAAQNGVRAMSSLGS		-0,0462		DESERTFGGKIVTPS		-0,2666
AMFGYAAATATATAT		-0,0465		ASDFAYLVDFGIARA		-0,2669
QAAGNNMAQTDSAVG		-0,0471		PLPTLPVPVLRDELP		-0,2679
GTGSTGLFNAGNFNT		-0,0476		FAWTRGLQHRGKLDG		-0,2701
DRAGVKDVILESAIT		-0,0477		QQVSIAPNAGLDPVN		-0,2706

HPDSDIFLDDVTVSR		-0,0482		HIGLYGNAGGLGPTQ		-0,2709
AADILKDESYKVTGT		-0,0487		PDEHSLLAGREQVRH		-0,2743
GSDVPLNPSTGQPDR		-0,0489		RAGAGVLLRTERLKS		-0,2746
VGGGNVGFGNIGDAN		-0,0489		MYCPSVMADVASGNL		-0,275
QVRRPGDLQTLEFVA		-0,0491		AIPPRGTQAVVLKVY		-0,2751
GDVQSDTVAQGYGSL		-0,0492		PPQEQGLIPGFLMPP		-0,2753
GVAVDSAGNVYVTSE		-0,05		PTTYDHPTFAVHDTL		-0,2754
TRFVNVFEQACPGQT		-0,05		LESSSLLKSLEGTYR		-0,2782
DIGIKTGNERAKLLG		-0,0504		VGADPTSDIAVVRVQ		-0,2795
EVLLKIAQGRLFYNT		-0,0506		NAGSTNVGLANMGVG		-0,2821
TSAGREAAALSGDVA		-0,0507		VAITLTWSQLYRRTL		-0,2826
FGMSGTNVHAIVEEA		-0,051		PELDVVGEAGSVAEA		-0,2832
GQMGARAGGGLSGVL		-0,0513		YAGLTAANTKKVAMA		-0,2845
EDKILVQANEAETTT		-0,0514		RVAGAYKAPAETQAQ		-0,286
AGAGEGSAITDADFK		-0,0516		GLREVADGDALYDAA		-0,2876
YAGLTAANTKKVAMA		-0,0519		VGGGNVGFGNIGDAN		-0,2884
LVLSADNMREYLAAG		-0,052		KHRIEDAVRNAKAAV		-0,2896
DRAAQIAGADAKLII		-0,0521		VPDNLAELGRLTQLP		-0,2901
PAAEIVKDGDDAVVR		-0,0525		HFRDNSWSVKVPRRL		-0,2918
PLPATGRGLRAAAEA		-0,0526		VDMANPMSPVNKSFE		-0,2918
PDSDMTTAVMRPSKT		-0,053		STNNDDPAGACRPFD		-0,2929
TVIGARDDLMVNNAG		-0,0536		QEITRGRCAAISVPG		-0,2944
FGSGVSGLYNTGGLP		-0,0541		LDPSQGMGPSLIGLA		-0,2945
TQYLSKQDPEGWGKS		-0,0542		NSGSFNTGIGNGGTG		-0,295
PISVIFRSDKSGTSD		-0,0545		AKERAHNAGAKNVEE		-0,2951
GASVTVGPILINGVN		-0,0547		AGVTCVGEHETLTAA		-0,2957
VTSALSSSHGLSVAD		-0,0548		VLAGASLVAVSWAVG		-0,296
REPIVISNVPRLVPG		-0,0552		IQMSDPAYNINISLP		-0,2967
LVAGVPVIGVAGNLD		-0,0554		AESNNQVVLPFTDIT		-0,2971
INTGWFNTGNANTGI		-0,0562		NDDPAGACRPFDRDR		-0,2979
MKFARSGAAVSLLAA		-0,0564		DAMARLGAIKREVTS		-0,2991
GALLRYAATNRLGSH		-0,0566		DGVGALLRYAATNRL		-0,3007
GLTVPPPVIAENRAE		-0,0566		ILELGTSAKMLSVVP		-0,3017
TPANQAISMIDGPAP		-0,0569		ARGANILARIMGASI		-0,3019
SLIGLAMGDAGGYKA		-0,0569		LLPFEEAPEMTSAGG		-0,3026
VSVDIPAVTITGTRI		-0,0571		NGDEVQIGKFRLVFL		-0,3037
PGIGNTTTVPSSGFF		-0,0574		VTLGPKGRNVVLEKK		-0,3043
GTAVKAFLQSTIGAG		-0,0575		EIVCSKYPDATTGTA		-0,3047
KIRAWGRRLMIGTAA		-0,0578		DKRDTVGVRIDRKRR		-0,3069
TGPGSVAGMAQDPVA		-0,0587		LVVGNGGPGVTALVM		-0,3075
LEDPYEKIGAELVKE		-0,0592		IAGAKIMGQGNDLVL		-0,3077
ELKGTDTGQACQIQM		-0,0594		GQPVLAIGSPLGLEG		-0,308
HAVFNAASQAFNTPA		-0,0594		TPDAAPGLAARRRRI		-0,309
GNVDTGALMSGNFSN		-0,0595		FGSGVSGLYNTGGLP		-0,3102
TAPFTVVGADPTSDI		-0,0598		RVQGVSGLTPISLGS		-0,3104
HACVAASAQGMLSST		-0,0599		PANAFVADYLPGEAA		-0,3125
QQALVAGVPVIGVAG		-0,0604		IGKLLDNDKSPSRKT		-0,3138
DSGTTSIAYETVQTA		-0,061		RAVDRAAQIAGADAK		-0,3151
GLLPPEVNSSRMYSG		-0,0611		LAQIKAGDKVLIHSA		-0,3154
AGNLGLFNSGTGNIG		-0,0613		DEDDIKATYDKGILT		-0,3169
MRVQVRRPGDLQTLE		-0,0615		GTGSTGLFNAGNFNT		-0,319
NNGNGARVQRVVGSA		-0,0618		RGGPGGAPGCWQPIT		-0,3194
LGSSSDLRVGQPVLA		-0,0619		ADFPALPLDPSAMVA		-0,3205
VRAMSSLGSSLGSSG		-0,0622		PDTAKEKPQEGTVVA		-0,3206
VTVGPILINGVNIPA		-0,0623		AMFGYAAATATATAT		-0,3213
ATVSPAMVAANRTRL		-0,0625		RAPKAVKEYARKHPH		-0,3235
LSSMLKGFAPAAAAQ		-0,0626		DNQTGIGGLNSGAGN		-0,3239
GGLNSGTGNLGLFNS		-0,063		GKLKDTKAVNPESTF		-0,3249
ALVREGLRNVAAGAN		-0,0632		VFAPTNAAFDKLPAA		-0,3254
RVTPVAVIGMGCRLP		-0,0634		GLERHAKAGVMTPVS		-0,3261
APPLAVAANLAGALP		-0,0635		HACVAASAQGMLSST		-0,3263
TPDAAPGLAARRRRI		-0,0638		GTAVKAFLQSTIGAG		-0,3267
IGNIGDRNLGIGNTG		-0,0639		DRARNVRMELLAKSG		-0,3275
FSIIIPTLNVAAVLP		-0,0642		EIVCSKYPDSQVGTA		-0,3291
GLNTPLAVAVDSDRT		-0,0644		HGTVLLAVRGLRMGT		-0,3303
VFRARMQREADTAGR		-0,0645		YGSFRRSFRLPAHVT		-0,3321

TATLLPFEEAPEMTS		-0,0648		NMQRQAVPLVRSEAP		-0,333
TPADGSAPIVHEMVS		-0,0652		AFGRFPIIDDREPQL		-0,3332
TSGKVRRGACVEQYR		-0,0654		SGIPTLSTTGPVHAV		-0,3342
RPGRVPPALDQVIAK		-0,0658		MLSSTSSDALRQTAR		-0,3346
AVQTAAQNGVRAMSS		-0,0658		GAAEKQDPLSGLTDQ		-0,3346
MPVLSKTVEVTADAA		-0,0658		SDQDAMARLGAIKRE		-0,3356
EFPAIPGFPALPGLP		-0,066		RRCRRALRQIGVLER		-0,3356
QNQALLNARDELQAQ		-0,0661		NISVPDSSVPIIIVP		-0,3363
VNINTKLGYNNAVGA		-0,0661		AGSNNQTVLPFDGLN		-0,3371
RVQAATIKRFADRFA		-0,0661		VKKPETINYRTLKPE		-0,3372
WYVARLTPSPLGHEE		-0,0676		VGAPVDALVNLADEE		-0,3402
GFTLSGATPADAYPT		-0,0676		AGSPEFQRHRDKIVQ		-0,3402
SSSAGADPTALNGMP		-0,0687		LTSAAERGPGQMLGG		-0,3406
LDQASQRGLGEAQLG		-0,0689		NNQTVLPFDGLNYPE		-0,3412
GKSPGFGTTVDFPAV		-0,069		GLGDKFGESIVNANT		-0,3422
GAAASLVGFNRAPAG		-0,069		CAPVFVMAAPGQPLP		-0,343
RLLAKSLARLRDQLE		-0,0691		LPNISASVPQLVAAI		-0,3431
AGSPEFQRHRDKIVQ		-0,0692		SMTNSGVSMTNTLSS		-0,3433
KHLESRDAKQELIDS		-0,0693		AAIDAATSSSQESGV		-0,3433
VIGAGKPLLIIAEDV		-0,0694		GPGVTALVMGGTDSL		-0,3438
PILHRRRREFKARAA		-0,0696		GGLNSGTGNLGLFNS		-0,345
MGATVTVLDINIDKL		-0,0698		GAYHLMRTQGGRGVL		-0,3453
QRHRDKIVQRCLPLA		-0,0701		QSESGIAGAGTGPPT		-0,3465
VGAQNLGAANAGSGN		-0,0703		TLPVPVLRDELPGLG		-0,3469
PLSGLTHKRRLSALG		-0,0706		ATLAHVVRPFVLARS		-0,3473
ELTSAAVSYGSVVST		-0,0708		GITGNGQIGFGKPAN		-0,3475
AGSNNQTVLPFDGLN		-0,0708		HVSRLLAKSLARLRD		-0,3482
GVPVIGVAGNLDQHL		-0,0709		VPVLRDELPGLGPLP		-0,3485
EAYPPEVNSANIYAG		-0,0713		VARAQGMSQDMAQTF		-0,3487
GPKVVIDGKDQNVTG		-0,0715		SRPQSSSNNSVPGAP		-0,351
IRGTFKSVAVKAPGF		-0,0716		AITRVVVRDQPARQL		-0,3512
GIMDEERRTTVNLKA		-0,072		MSAYKTVVVGTDGSD		-0,3516
QNPTGPGSVAGMAQD		-0,0721		MADVASGNLPALPDM		-0,3516
QVGPQVVNINTKLGY		-0,0723		GEAQLGNSSGNFLLP		-0,3522
DGKDQNVTGSVVCTT		-0,0724		GQRCAPVFVMAAPGQ		-0,3529
DLARVGRYKVNKKLG		-0,0725		EGYPKRARMLMDAAI		-0,3534
AADPEVVFVSGEVRS		-0,0728		GRGCAQYAAQNPTGP		-0,3536
PVTVIAATAGRSDLK		-0,0729		TKDGSHYKITGTATG		-0,3546
VAAELTSAAVSYGSV		-0,0732		CAQYAAQNPTGPGSV		-0,3556
HFRDNSWSVKVPRRL		-0,0736		TFESITSRMAEINGM		-0,3557
IVGLERLYAENPSAR		-0,0738		DYMDVSPRQMVSVAT		-0,3584
GTTGGGGTRSGTSTD		-0,0738		SALLDPSQGMGPSLI		-0,3587
AYTLARGRRHRPVRT		-0,074		DKPEKEKASVPGGGD		-0,3599
QSESGIAGAGTGPPT		-0,074		LFNVGGFNTGVANVG		-0,361
TGIGGLNSGTGNLGL		-0,0741		TSVAGDPGPDAGTST		-0,3611
YPADPKTDQEKAIKE		-0,0742		VSGLGLKEAKDLVDG		-0,3616
PNGYTEPILHRRRRE		-0,0745		AAIANRLLRRTATHL		-0,3627
KLNPDVNLVDTLNGG		-0,0748		DIMVRDGQLTIKAER		-0,363
AGVQYSRADEEQQQA		-0,0751		EKEKASVPGGGDMGG		-0,3632
TAPTQGILIHTGPSS		-0,0751		HLMRTQGGRGVLMGG		-0,3637
TVTVLDINIDKLRQL		-0,0753		TIKAERTEQKDFDGR		-0,3668
PAVFAVQVALAATME		-0,0754		GLILTNNHVIAAAAK		-0,3683
FKEMTLLELSDFVKK		-0,0755		VASPQSTVIGGTSDT		-0,3692
NQNTVLDAIQTDAAI		-0,0758		AFELAARRTTDDDMA		-0,3695
AGLMRHTIGQAEQAA		-0,076		KTDDVAGDGTTTATV		-0,3696
NAGSYNTGSFNVGDT		-0,0762		WNQAQTEVAGAGSGF		-0,3697
GNAGTASTGLFNVGG		-0,0764		TQVLVPRSAIDSMLA		-0,3701
RAVDRAAQIAGADAK		-0,0765		YRTDLADRVDTLVGA		-0,3702
ELAIPMSGIAMAVVG		-0,0766		FSVGSGQTYGVDVVG		-0,3703
LGSELTMTDTVGQVV		-0,0768		NSEEFLDAIADNLEK		-0,3705
SNGAWGKGAGKSFQG		-0,0768		TIFPNGISIPNNPLA		-0,371
GTDTGQACQIQMSDP		-0,0769		VITSADVRASLERMA		-0,3711
YPITGKLGSELTMTD		-0,0771		CQGDLGMSHQDWQAQ		-0,3722
LKASDVNGPLINSRT		-0,0775		AATQAAGAGAVADAQ		-0,3742
ISFLDQASQRGLGEA		-0,0775		NQNTVLDAIQTDAAI		-0,3742
GAAHQGQGGAKSKGS		-0,0786		GGQFTLPGRSLMFVR		-0,3753

DVVVLLESMKMEIPV		-0,0786		VRAMSSLGSSLGSSG		-0,3755
GNWPSQTGHSPAVPN		-0,0786		VKRADLVIGAVLVPG		-0,3756
EIVCSKYPDATTGTA		-0,0792		LTSLTSAAERGPGQM		-0,3758
QNLARTISEAGQAMA		-0,0795		ARLFIDDDGQEQRAQ		-0,3761
MGVPPLAGASRTDME		-0,08		LVLSADNMREYLAAG		-0,3771
VSQLHAGPRKSALDE		-0,0801		SDGSGVTPGTGMPAA		-0,3773
SEAIAASYDAGVLTV		-0,0804		VGSWIGSSAGLMVAA		-0,3778
QVVKLLAGSTTSTVL		-0,0805		NVTGGGATTGQASAK		-0,3783
AAKAGLMRHTIGQAE		-0,0805		VLSWLSDHQVHNVAA		-0,3791
PTFSSSAGADPTALN		-0,0806		LTVRVAGAYKAPAET		-0,3802
ALSGQLNPQVNLVDT		-0,0806		PAFSPPAQALGGGVG		-0,3814
GSVAEAMARVPAARP		-0,0811		AQDPVATAASNNPML		-0,3817
SLAAIAIAFLAGCSS		-0,0813		KERKHRIEDAVRNAK		-0,3834
AAPRSVGVPLYLLLG		-0,0821		FGGRFVEIGKADVYG		-0,3836
TIFPNGISIPNNPLA		-0,0825		GAAHQGQGGAKSKGS		-0,3839
GTVTRHYRQYQAGKP		-0,0826		RLLAKSLARLRDQLE		-0,3839
FMQAAIGPGQEGLDQ		-0,0827		GQMGARAGGGLSGVL		-0,3848
RGAHIRSVTLSVEQQ		-0,0827		PGVDPDKDVDIMVRD		-0,3849
DPDKDVDIMVRDGQL		-0,0828		IVGLERLYAENPSAR		-0,3855
VTLGAAFGGLTTAHT		-0,0833		TLARVAAFIGEHEPS		-0,3857
VARAQGMSQDMAQTF		-0,0835		MAEMKTDAATLAQEA		-0,3866
TTIVEGAGDTDAIAG		-0,0836		VGAGERKMSALVDAS		-0,3869
TTGSGETTTAAGTTA		-0,0838		QNQALLNARDELQAQ		-0,3869
GEHEPSDLVYGDVIM		-0,084		DPDKDVDIMVRDGQL		-0,3876
DDRPINSADALVAAV		-0,0845		GNAGTASTGLFNVGG		-0,3881
TAVVPLHRSDGSGDT		-0,0855		QALNSGTDLPPTPIS		-0,3883
VDPVKLNLTLSAAAE		-0,0858		QSGLSIVMPVGGQSS		-0,3888
HGLHIEILIDPESQV		-0,0859		SGMTSQAATFAANRL		-0,3899
YPEGLAVDTQGAVYV		-0,0868		AYTLARGRRHRPVRT		-0,3911
YVAQKRKISDGDKLA		-0,0874		GNPNRPDGGILTRFG		-0,3928
VNAIRGSVTPAVSQF		-0,0876		QNAASIAGLFLTTEA		-0,3929
HLMRTQGGRGVLMGG		-0,0876		LAQPTQGTTPSSKLG		-0,3929
NGNQTDFGGSDVPLS		-0,0876		AALPAFSPPAQALGG		-0,3929
MTSAGGLLEQAAAVE		-0,0877		QGELSKQTGQQVSIA		-0,393
QRRKIDRLLDVAGVQ		-0,0883		ASLVGFNRAPAGPSG		-0,3932
GLRMGTGTSERDERD		-0,0883		PGEPPTKESAQTLLE		-0,3938
LLRTERLKSQRVAGA		-0,0883		ARLGAIKREVTSALS		-0,395
FAWTRGLQHRGKLDG		-0,089		AYAIKKHGVGVKCAT		-0,3952
DPNGVVLTNNHVIAG		-0,0895		AVQTAAQNGVRAMSS		-0,3961
AFELAARRTTDDDMA		-0,0899		FMQAAIGPGQEGLDQ		-0,3961
PGVDPDKDVDIMVRD		-0,09		KVNTLLDVAQANLGE		-0,3967
CLFSSGAALLGSPGQ		-0,0901		RRSPWGEMFASTGQR		-0,3983
TAALAKETATQARAA		-0,0902		RQRPRAGMLAIGAVT		-0,3991
YCEELKGTDTGQACQ		-0,0907		EFEGELPRLFVVTRQ		-0,3995
TLGYTSGTGQGNASA		-0,0909		AAHDALTTSEQHQAT		-0,4
VGNGYWQKPDESERT		-0,091		LTNNHVIAGATDINA		-0,4002
LTNNHVIAGATDINA		-0,0911		ASQRGLGEAQLGNSS		-0,4006
VYVADRGNNRVVKLA		-0,0914		ARGTHQASGLGDVGE		-0,4011
QPRAGASTAWVPSPT		-0,0917		FVVTRQAQIVKPHDS		-0,4015
PGGVEVPVETDDIDH		-0,0917		PKTQLTMLSARAGLV		-0,4016
GIRLTPKVIATNKTA		-0,0917		FEATLAATVSPAMVA		-0,4029
GGAAPVSAGVGHAAL		-0,0923		GSDVPLNPSTGQPDR		-0,4033
AFVDSLTSQVGGRSI		-0,0925		LRHGQILFTFLHLAA		-0,4041
RLAGWQERYPNVAIT		-0,0928		SDLAYTLARGRRHRP		-0,4047
GIMDALFTGLIAIHG		-0,093		AMSNLLGQNAAAIAA		-0,4047
VTGKARTTVARDADM		-0,093		HWAITDGNKASFLDQ		-0,4053
PNAGSRFLLDQAITS		-0,0932		RLAGWQERYPNVAIT		-0,4056
EAVIGNRRRQDSKES		-0,0934		TRFVNVFEQACPGQT		-0,4071
EAAEEQSEFDVILEA		-0,0935		AGMAQDPVATAASNN		-0,4075
LPDFLGGFAGLPSLG		-0,0948		HLVEGVARVLTKPRF		-0,408
NDDNVTGGGATTGQA		-0,0948		LMDAWAGPVVMQLME		-0,4089
TPGCVAYIGISFLDQ		-0,0954		DGTRTWRTGRQATTL		-0,4092
PTQGHPLSSATDEPE		-0,0955		TIDAIELPAISFSQS		-0,4096
AEAYLNQNIAQQLRA		-0,0956		HPMLRTTLIDVDEHT		-0,4098
TMPHHRMLATRHTQT		-0,0959		SEEGSGIILSAEGLI		-0,4103
LIARWEQRDVMAREV		-0,0959		KDQTSDEVTVETTSV		-0,4105

AIDQGGCFEGSRPTT		-0,096		AEAERAPIDAGTAAS		-0,412
MGTVPNVGLMAQQAE		-0,0961		FGMSGTNVHAIVEEA		-0,4128
GLNYPEGLAVDTQGA		-0,0962		NALGGNRPAVYAPKS		-0,4131
VPLNPSTGQPDRSAE		-0,0964		GVAVDSAGNVYVTSE		-0,4132
NSEEFLDAIADNLEK		-0,0965		SSSAGADPTALNGMP		-0,4136
AGDTDAIAGRVAQIR		-0,0966		LSTDELLDAFKEMTL		-0,4162
TRGRCAAISVPGDRR		-0,0967		AVDGAGTVYYVTDFNN		-0,4172
QPATTVALGRSVCPM		-0,0968		GGCAVLLEPHACVAA		-0,4173
LENNEFNVVDVGSLN		-0,0971		DQQEILNRANEVEAP		-0,4176
LVSGAGNVGQQLSGV		-0,0972		QGYGSLGLMTSVLMT		-0,4186
DRARNVRMELLAKSG		-0,0974		GSKPPSGSPETGAGA		-0,419
VVRGTATNQDGRTET		-0,0975		RSTLEKDNTVGTDEA		-0,4191
GQLNPQVNLVDTLNS		-0,0981		KAVEKVTETLLKGAK		-0,4193
ISGATIPQGEQSTGK		-0,0983		GNVDTGALMSGNFSN		-0,4194
ALAVAVSPPAAAGDL		-0,0993		VEERPIVGAPVDALV		-0,4204
NSGVSMTNTLSSMLK		-0,1		MDYEQDWDGVAITLT		-0,4214
DKRDTVGVRIDRKRR		-0,1002		KSLENYIAQTRDKFL		-0,4215
MSNAPKIKVSGPVVE		-0,1002		IGVLERPVGDSSDCG		-0,4217
AQVGAYHLMRTQGGR		-0,1004		AGADPTALNGMPAGL		-0,4218
SVAVKAPGFGDRRKA		-0,1005		AEAMARVPAARPDVA		-0,4224
KGSQQEDEALYTEDR		-0,1006		VGPGCAEYAAANPTG		-0,4226
QLAEHFASLADVLTK		-0,1006		SVAVKAPGFGDRRKA		-0,4245
TLDYNANGSGAGVTQ		-0,1011		ALPGIVGGAPNPYTY		-0,4255
GTTDRAGVKDVILES		-0,1011		NAGSYNTGSFNVGDT		-0,4277
KQKQELDEISTNIRQ		-0,1015		LGSELTMTDTVGQVV		-0,4284
PVAITTESVGKTIAG		-0,1015		PGALGENGNGGMVTG		-0,4285
TTGEAGNQNTVLDAI		-0,1016		TPANQAISMIDGPAP		-0,4286
SGIPTLSTTGPVHAV		-0,1038		GNWPSQTGHSPAVPN		-0,4293
QVTNDKDTLGAKIVE		-0,104		PVTVIAATAGRSDLK		-0,4299
AMAAQLQAVPGAAQY		-0,1044		MTVPGGVEVPVETDD		-0,4302
KDEAAAAQRRCGSPA		-0,1046		AADILKDESYKVTGT		-0,4322
LEGTYRLAPKGSGTE		-0,1047		PALDQVIAKGMAKNP		-0,4329
IPALNPNVTGSVGFG		-0,1048		VVPSVVMLETDLGRQ		-0,4336
RKIPLTLVHAVSPEV		-0,105		KVPRRLKELHLRLGT		-0,4344
LYGNAGGLGPTQGHP		-0,1055		MGTVPNVGLMAQQAE		-0,4348
EQGLIPGFLMPPSDG		-0,106		QLVAAGGGPSQLASM		-0,4355
VGEQLSGLSSAGTAL		-0,1067		DAFPNEFPDPTISVQ		-0,4361
MSSLGSSLGSSGLGG		-0,1073		NANIGFGNRGDANIG		-0,4391
RRTTVNLKACIKAAA		-0,1075		EHTDVERVAQQLLSG		-0,4406
KVNTLLDVAQANLGE		-0,1077		GAGSGFANFGSLGSG		-0,4408
ITTTAGPQLVVPVLN		-0,1077		VPLNPSTGQPDRSAE		-0,4411
EPVDSAVLANGDEVQ		-0,1078		YRATNFKVDQPGTVT		-0,4422
AQTDDQQLAEHFASL		-0,1079		QNIAQQLRAQVMGDL		-0,4424
LVAGLIAPPLAVAAN		-0,1086		DVVVSVVASPQSTVI		-0,4427
LSVEQQRLARQRVAA		-0,1086		PVDALVNLADEEKAD		-0,4427
SFRLPAHVTSEAIAA		-0,1088		GWRAACRSNPALAKG		-0,4427
ATVDPAAVAVNRMAM		-0,109		VAPVSPSTASSRTDT		-0,4435
PVISEHRLVGIVTEA		-0,1092		DLVYGDVIMRSTNFR		-0,4442
DDLMVNNAGLVCGGV		-0,1096		DLQTLEFVASDRVPP		-0,4446
TKRRITPKDVIDVRS		-0,1097		AVGEPVVAMGNSGGQ		-0,4475
EAETTTASGLVIPDT		-0,1105		DDRPINSADALVAAV		-0,4479
GVNLPGTAVVPLHRS		-0,1105		TSVLMTADGKTVEAE		-0,4482
INAFSVGSGQTYGVD		-0,1106		DGSAPIVHEMVSIPE		-0,4494
RVQGVSGLTPISLGS		-0,111		ARIFAGAGLGPMLAA		-0,4495
NTGNWNIGIGITGNG		-0,1115		EVLLKIAQGRLFYNT		-0,4504
VVAGEPRPGNYRYAI		-0,1117		AVVLPGLVGLAGGAA		-0,4519
NSGLEPGVVAEKVRN		-0,1123		PMLSTLTSALSGKLN		-0,4531
ILTVSVAVSEGKPTE		-0,1125		QNPTGPGSVAGMAQD		-0,4534
RFNLQERVIRPSYGL		-0,1129		NTAASDNFQLSQGGQ		-0,4534
VSGIGNVGEQLSGLS		-0,1132		APKIKVSGPVVELDG		-0,4564
VGADEDDIKATYDKG		-0,1138		RTVSLPVGADEDDIK		-0,4573
IPETDGAEKGPTYNK		-0,1138		LKRGIEKAVEKVTET		-0,4576
LAKLAGGVAVIKAGA		-0,115		VKAFLQSTIGAGQSG		-0,4576
DGQIDGFSTPPITID		-0,1156		GDTNTGGFNPGSTNT		-0,4583
DGALPLLAPMSEVAG		-0,1158		RPIVGAPVDALVNLA		-0,4597
QPVVLAAATGDLPTI		-0,1159		TGVANSGNVDTGALM		-0,4597

NRAAAALMAKLRGAA		-0,116		AQQLRAQVMGDLDKL		-0,4607
GPYLQRGVADLVPTA		-0,116		GALLRYAATNRLGSH		-0,4609
IAGAKIMGQGNDLVL		-0,1161		LALLAGVFGGAASCA		-0,4611
PDKSTGLANPGQFAG		-0,1161		VTIPTITTSPIPLKI		-0,4619
FEAALAATVDPAAVA		-0,1161		PAIKIDPPPPPQEQG		-0,4627
DVVVSVVASPQSTVI		-0,1165		DKLPAATIDQLKTDA		-0,4637
GLAAGLDPNTATAGE		-0,1166		SRQSKTAASPSPSRP		-0,465
VVVNEGDQIDKGDVV		-0,1167		TGIGGLNSGTGNLGL		-0,4684
LSGAQQPNGYTEPIL		-0,1169		AGDTDAIAGRVAQIR		-0,4698
TVLPFTGLNTPLAVA		-0,117		VTYELAVDLAVPMIG		-0,4699
QATPTFSSSAGADPT		-0,117		AATALDNDGEGTVQA		-0,4701
AVDVASHSPQVDPIL		-0,1171		WTHARLFIDDDGQEQ		-0,4713
QLMNNVPQALQQLAQ		-0,1176		DALRIVMADSRPLTN		-0,4722
AAPSIPAANMPPGSV		-0,1176		LRALLKQYGPLTPAR		-0,4731
LVYSDGTATGAADPR		-0,1176		ASNNPELTTLTAALS		-0,4744
DLQTLEFVASDRVPP		-0,1178		DAKQELIDSLEEAVR		-0,4751
AGVNYGDPVDAKALG		-0,1179		EQRVPDNLAELGRLT		-0,4767
LNRPVSTTGEAGNQN		-0,1181		IAFNSGLEPGVVAEK		-0,4808
IAFNSGLEPGVVAEK		-0,1182		DLAAAVDKDGTAFLR		-0,4819
GTRTGNVTLAEGPPA		-0,1186		PQNAIPLTIDASGVL		-0,4835
VKRADLVIGAVLVPG		-0,1188		RSNHGTCANQCPIVD		-0,4842
AIAIAFLAGCSSTKP		-0,1189		GLSDLYSKIESLPAS		-0,4859
VHEMVSIPEDGGVVL		-0,1189		ATVDPAAVAVNRMAM		-0,4862
QTGQQVSIAPNAGLD		-0,1191		RLNTEGVAAAVKQVL		-0,4882
TSYTSDEAMLDAILA		-0,1192		QATPTFSSSAGADPT		-0,4928
CERLVRGLDTIAATG		-0,1193		TGVLPPAANDVSVLT		-0,4945
PELVPTYNLPANHRY		-0,1193		LLSFASLPTVGQVTA		-0,4965
AGSLASGVLNSGVDI		-0,1195		GEVDEEAATALDNDG		-0,4976
QMLLLDEQTPIDAVA		-0,1204		IDTSNAVDTPDMLAS		-0,4978
LQSLGAEIAVEQAAL		-0,1214		MLSARAGLVMDPGSK		-0,4989
YRTDLADRVDTLVGA		-0,1214		GNPLADLIQPDLRVL		-0,5
DIDHVNAHATGTQVG		-0,1215		GIPDPGDIFNTGSSL		-0,5011
GFGNDTTAPSSGFFN		-0,1216		VLAIGSPLGLEGTVT		-0,503
TTTAAGTTASPGAAS		-0,122		DLADRVDTLVGAGER		-0,5044
RNFLAAPPPQRAAMA		-0,1221		GKSFQGGVGEGARGN		-0,5121
VPGVEPADVVVIGAG		-0,1221		PIAHSTVLVAPVSPS		-0,5184
HEGQTTMTVPGGVEV		-0,1227		NATNLRGILGRLIEM		-0,5236
AYAIKKHGVGVKCAT		-0,1238		ILSGSERVQAATIKR		-0,5412
VSEHLKLNGKVLAAM		-0,1238		VVIDRIPVELHASTT		-0,5428
IEEHEIDARDTKLGA		-0,1238		GFTIPGGTLIPQLPL		-0,5441
DFTPAAEIVKDGDDA		-0,1239		NKGDLAAAVDKDGTA		-0,5484
TNDAIVDTDGSEVFE		-0,125		AARALPLTSLTSAAE		-0,5494
TGVLPPAANDVSVLT		-0,1259		QSIGDLIAEAMDKVG		-0,5737
GRSLLDNRAAAALMA		-0,1259		AEINGMSRDMASTFT		-0,5843
MTVETSQTPSAAIDS		-0,1274		VGEQLSGLSSAGTAL		-0,5899
PILDDLAAALADIAP		-0,1276		VEIDLCDYRDVDGQY		-0,5976
EEAPAEASAPESSPG		-0,1276		QLVVALPDKSTGLAN		-0,6021
NDDPAGACRPFDRDR		-0,1277		ADHLTRLVDAADPEV		-0,6202
SALLQTTDGSITYNE		-0,128		DVRAEIVASVLEVVV		-0,6216
PALDQVIAKGMAKNP		-0,1282		PLLEKVAKEAADEAK		-0,6468
IAQTRDKFLSAATSS		-0,1288		RAELMILIATNLLGQ		-0,6511
GHGNVGFGNSGLGAA		-0,129		LDELKLEGDEATGAN		-0,6551
MSAYKTVVVGTDGSD		-0,1291		TIDQLKTDAKLLSSI		-0,6871
GCAEYAAANPTGPAS		-0,1298		NLAELGRLTQLPDTN		-0,6927
AENRAELMILIATNL		-0,1299		NVLEHLAENLKQRAE		-0,7053
CQGDLGMSHQDWQAQ		-0,1303		LAVAANLAGALPGIV		-0,7506
AGSWLLIDTSNAVDT		-0,1305		LPSLPDIIDGLWPPK		-0,7516
ENSDSDYDREKLQER		-0,1313		VASHSPQVDPILDDL		-0,8035
LLPFEEAPEMTSAGG		-0,1314		LIDGALKDLKKRVEG		-0,8127
EEITRDIPNISDEVL		-0,1319		TVELIEDLARRAVQT		-0,8261
QYSRADEEQQQALSS		-0,1319		VPEMFRELVGLPAGS		-0,8474
SNVGQNPITIVNIGL		-0,1324		LENADLSLLGKARKV		-0,8481
QLTMLSARAGLVMDP		-0,1333		GYGVDIVLNSLTGAA		-0,8482
VLDINIDKLRQLDAE		-0,1347		DAAIGKLLDNDKSPS		-0,8638
DAKALGQSVCPILAE		-0,1348		LQQTIDAIELPAISF		-0,8819
QIAAALDAAHANGVT		-0,1349		GPYQLLRLLGRGGMG		-0,9103

DALRIVMADSRPLTN		-0,1361		VGLSTIAGRLLGSVP		-0,9129
PLGLEGTVTTGIVSA		-0,1365		ATVALTFQDPSGGSR		-0,9209
LTGDNQTGIGGLNSG		-0,1366		AGKPLLIIAEDVEGE		-0,9913
QGELSKQTGQQVSIA		-0,137		GILGRLIEMLTTLPK		-1,1355
SGVDAEITTTAGPQL		-0,1382		LPPAANDVSVLTAAR		-1,1472
SAPAGGRAAHADPCS		-0,1383		MLKRKAERRLIDGAL		-0,1864
PDTAKEKPQEGTVVA		-0,1386		LGGANIPAEFLENFV		-0,4329
PQGGVTDERSDSVLS		-0,1387				
VRDGQLTIKAERTEQ		-0,1391				
SAGTVGLIKAILSLR		-0,1393				
IVDSDTCIECPDGTV		-0,1396				
PKTQLTMLSARAGLV		-0,1397				
HIGLYGNAGGLGPTQ		-0,1398				
TTVALGRSVCPMVVA		-0,14				
LQLRGAGGLPSAAIG		-0,1405				
THQASGLGDVGEAFV		-0,1406				
NEVEAPMADPPTDVP		-0,1411				
ADFPALPLDPSAMVA		-0,1412				
TWKLLLDRQSGIRTL		-0,1414				
PTITNDGVSIAKEIE		-0,1414				
GAYHLMRTQGGRGVL		-0,1419				
HWAITDGNKASFLDQ		-0,1422				
GIVGGAPNPYTYPEN		-0,1424				
SERTAPSRACQGDLG		-0,1425				
NLADEEKADLLVVGN		-0,1433				
HLIPANVNADIKATT		-0,1436				
KERKHRIEDAVRNAK		-0,1438				
AEINGMSRDMASTFT		-0,1439				
TGMPAAPMVPPTGSP		-0,1441				
ARWSPGDSATVAGPL		-0,1443				
RGDSDQDAMARLGAI		-0,1445				
RLSALGPGGLSRERA		-0,1448				
ATADLSQRLGRAPSA		-0,1456				
TLARVAAFIGEHEPS		-0,1459				
GVVAEKVRNLPAGHG		-0,146				
VRELLATVFKLTADG		-0,1461				
QEGLDQYGSIPLPKS		-0,1463				
VDCGGKKTLKASGST		-0,1476				
SDLAYTLARGRRHRP		-0,1484				
KTARALAQYLADTLA		-0,1485				
GTAVSARNTAVCAPT		-0,1485				
AAIGPGQEGLDQYGS		-0,1486				
RMTSGSSSGFKVRPS		-0,1492				
AIPPRGTQAVVLKVY		-0,1502				
RAHNAGAKNVEERPI		-0,1518				
HQTLQGADLTVIGAR		-0,1519				
DERGIVRIGAEVRDG		-0,152				
DLGIEHRDATDDQVT		-0,1525				
SQMHVSRLLAKSLAR		-0,1525				
DKGILTVSVAVSEGK		-0,1526				
VASVLEVVVNEGDQI		-0,1535				
MTVPGGVEVPVETDD		-0,1537				
SSDLRVGQPVLAIGS		-0,1543				
VGAPVDALVNLADEE		-0,1551				
STGMSGGFVTAPTQG		-0,1553				
ADHVAAVALFGEPSS		-0,1555				
DQAITSAGRHPDSDI		-0,1556				
RVSVGNLRIARVLYD		-0,1559				
TSTAPNAAPEPVARR		-0,1568				
APKIKVSGPVVELDG		-0,1569				
LLKGAKEVETKEQIA		-0,1573				
RTERLNTEGVAAAVK		-0,158				
EETLNGLIQFDAAIQ		-0,1581				
MLEGCILADSRQSKT		-0,1581				
DGTRTWRTGRQATTL		-0,1587				
SLPSYYPDQKSLENY		-0,1593				

GESAAAVVAGALSLE		-0,16				
ASQRGLGEAQLGNSS		-0,1616				
ESAGAVGGDSSAELT		-0,1634				
ANAGNVNTGALITGN		-0,164				
DDARAITDTLGDVDA		-0,1642				
AAAVVAGALSLEDAA		-0,1645				
AANKQKQELDEISTN		-0,1647				
VGTADQVWADADLLL		-0,1662				
LTPQNQALLNARDEL		-0,1662				
PTTLHFPGFTIPTGP		-0,1682				
LQGADLTVIGARDDL		-0,1683				
EINTLFQTLTSIAEK		-0,1685				
AALPAFSPPAQALGG		-0,1688				
GNNRVVKLAAGSKTQ		-0,1689				
VEIDLCDYRDVDGQY		-0,169				
KDQTSDEVTVETTSV		-0,1696				
TPGTGMPAAPMVPPT		-0,1697				
SIDSGGGSDDDARAI		-0,1704				
GSLQGQWRGAAGTAA		-0,1711				
MSLLDAHIPQLVASQ		-0,1721				
PAVPGALGENGNGGM		-0,1722				
ILTGGQVISEEVGLT		-0,1727				
LALLAGVFGGAASCA		-0,1739				
QVESTAGSLQGQWRG		-0,1745				
LTVRVAGAYKAPAET		-0,1762				
AQQAELDSALLAAAG		-0,1767				
IGVLERPVGDSSDCG		-0,177				
ATVLAQALVREGLRN		-0,178				
DTVPLAGVVLAGGES		-0,1787				
EQAGLRGLLRVISSE		-0,1791				
AGASTAWVPSPTAAT		-0,1791				
AVIGNRRRQDSKESK		-0,181				
NSGSFNTGIGNGGTG		-0,1813				
CTDDGEMALGKNLLV		-0,1815				
STSSDALRQTARQLA		-0,1818				
VTYELAVDLAVPMIG		-0,1828				
INISLPSYYPDQKSL		-0,1837				
LLKQYGPLTPARAVA		-0,1852				
ESRDAKQELIDSLEE		-0,1855				
LRALLKQYGPLTPAR		-0,1868				
GGYSQGATVIDLSTS		-0,1896				
GMDFVGVVTAVGEGV		-0,1897				
SYYPDQKSLENYIAQ		-0,1914				
NVGQQLSGLLFAGTG		-0,1919				
VVPSVVMLETDLGRQ		-0,1922				
KVAVSVGDVIQAGDL		-0,1927				
NETDFAGSDVPLNPS		-0,1928				
EAEAAHGTVTRHYRQ		-0,1932				
SAAERGDVNPVGGLE		-0,1933				
AASAAPSIPAANMPP		-0,1935				
FEATLAATVSPAMVA		-0,1938				
DERSDSVLSDSSPVA		-0,1943				
AITFPEIPANADGEL		-0,1945				
EQQRLARQRVAAAGF		-0,1962				
YQQRSEKVLTEYNNK		-0,1962				
VPDNLAELGRLTQLP		-0,1963				
AEAERAPIDAGTAAS		-0,198				
LFFASKLAAAGCGRI		-0,1981				
KSLENYIAQTRDKFL		-0,1983				
APEMTSAGGLLEQAA		-0,1983				
KLREPLEVPGLLDVQ		-0,1985				
QLVVALPDKSTGLAN		-0,2				
VKLNLTLSAAAEALT		-0,2001				
ARIANGMGATVTVLD		-0,202				
HLVEGVARVLTKPRF		-0,2024				
VEFAEQIRRDTDGYG		-0,2025				

DPRHAGPATAADAGD		-0,2032				
NKGDLAAAVDKDGTA		-0,2032				
GEVDEEAATALDNDG		-0,2036				
FVNNEALPGTDIDPD		-0,2052				
LAQPTQGTTPSSKLG		-0,2052				
VAAVALFGEPSSGFS		-0,2058				
DSSDCGTIRVGSFRG		-0,2093				
VKDVGAGRSLLDNRA		-0,2095				
DKVVADLTPQNQALL		-0,2109				
GGSASRANEYADVPE		-0,2116				
LAASRACTDALLDSG		-0,2126				
AVVLPGLVGLAGGAA		-0,2132				
FSIPQLGFTLSGATP		-0,2135				
ELPGLGPLPATGRGL		-0,2137				
NSTELPSSGFFNTGG		-0,2138				
SSTAVIPGYPVAGQV		-0,2142				
AAVYRTDLADRVDTL		-0,2149				
CSKYPDSQVGTAVKA		-0,2155				
SSMRAVDRAAQIAGA		-0,2155				
VELGYDRTAHQDVPS		-0,2162				
RRSPWGEMFASTGQR		-0,2166				
TLVVNKIRGTFKSVA		-0,217				
SDQDAMARLGAIKRE		-0,2174				
LVEHMVGILGQRCAP		-0,2183				
TAAAVVLPGLVGLAG		-0,2192				
NPGELLPEAAGPTQV		-0,2194				
AANPTGPASVQGMSQ		-0,2203				
STNNDDPAGACRPFD		-0,2233				
HVSRLLAKSLARLRD		-0,2237				
AMDKVGNEGVITVEE		-0,2253				
ASGLVIPDTAKEKPQ		-0,2258				
PHENELIKKVKTYLK		-0,2258				
GLSDLYSKIESLPAS		-0,2272				
GFMNVGQQLSGLLFA		-0,2276				
LGAEIAVEQAALQSA		-0,2284				
GPGVTALVMGGTDSL		-0,2313				
ERAPIDAGTAASQRG		-0,2319				
VGPGCAEYAAANPTG		-0,2325				
LKRGIEKAVEKVTET		-0,2326				
APTTGVLPPAANDVS		-0,2336				
VLSWLSDHQVHNVAA		-0,2338				
YPDSQVGTAVKAFLQ		-0,2363				
ANSPIDADGRFVEPR		-0,2365				
RGPGQMLGGLPVGQM		-0,239				
RTVSLPVGADEDDIK		-0,2393				
MDYEQDWDGVAITLT		-0,2398				
LESSSLLKSLEGTYR		-0,2408				
ARDADMLSELGEPVD		-0,2412				
IVKGENIPEPGIPES		-0,2432				
TTTASGLVIPDTAKE		-0,2464				
LPVEYLQVPSPSMGR		-0,2482				
LPTVGQVTATMGQLQ		-0,2502				
GLAAGLAGMGNIGLG		-0,2507				
DSATVAGPLAADSRG		-0,2512				
NTGGLPPGTPAVVSG		-0,2513				
EKEKASVPGGGDMGG		-0,2533				
GDTNTGGFNPGSTNT		-0,2534				
AGSKTQTVLPFTGLN		-0,2535				
LIPGFLMPPSDGSGV		-0,2559				
PQNAIPLTIDASGVL		-0,256				
EQRVPDNLAELGRLT		-0,2572				
WLLIDTSNAVDTPDM		-0,2625				
HGMLTDRDIVIKGLA		-0,2625				
PAVGQRGRALIATGE		-0,2638				
LNLGSGLSGIYNTST		-0,2639				
STGKASHASLGVQVT		-0,267				

FLRVLNRDRNYTAPG		-0,2676				
GEAGSVAEAMARVPA		-0,2678				
GKSFQGGVGEGARGN		-0,2678				
SKLMTRIAGAGAMGS		-0,2691				
DLADRVDTLVGAGER		-0,2691				
EYAAANPTGPASVQG		-0,2713				
ILELGTSAKMLSVVP		-0,2737				
GVVNISGIEIKDLAG		-0,274				
DLDVVAGEPRPGNYR		-0,2767				
NQSLIINLGLADVGS		-0,2791				
PAATIDQLKTDAKLL		-0,2806				
AVLVPGAKAPKLVSN		-0,2815				
PVATAASNNPMLSTL		-0,2825				
LPQWLSANRAVKPTG		-0,2848				
MLKRKAERRLIDGAL		-0,285				
VRAFAEPAGIKIEAS		-0,286				
AEAADAIRAMSNAEH		-0,2926				
LVLRPSWSPTQASGQ		-0,2947				
GNPLADLIQPDLRVL		-0,2967				
QSIGDLIAEAMDKVG		-0,2969				
DVRAEIVASVLEVVV		-0,2975				
GIPDPGDIFNTGSSL		-0,2981				
RIGAEVRDGDILVGK		-0,2989				
VPPPVIAENRAELMI		-0,2992				
TIDAIELPAISFSQS		-0,3001				
VAVSPPAAAGDLVGP		-0,3004				
ILSGSERVQAATIKR		-0,3004				
SIIDIPALPGFGNST		-0,3022				
QLPDTNIIKLPNISA		-0,3029				
AGVTCVGEHETLTAA		-0,3029				
DTESLSAGHAKPCAG		-0,3031				
DLLAAGVADPVKVTR		-0,3033				
LTSLTSAAERGPGQM		-0,3044				
TRIEAVPIAGFAQMR		-0,3063				
SLCLNVEVLSSDGAA		-0,3071				
TPEERLLRAIFGEKA		-0,3073				
GLYNTGGLPPGTPAV		-0,3078				
SAGTALNQSLIINLG		-0,311				
NISVPDSSVPIIIVP		-0,3113				
GMLVGSVGETVAQLA		-0,3131				
PIPEGLTTGRSCQTD		-0,3135				
GLTHKRRLSALGPGG		-0,3141				
FKQISDKMGLAIPGT		-0,3153				
AFGRFPIIDDREPQL		-0,3202				
GVDVVGYDRTQDVAV		-0,3221				
DIEETIQEITRGRCA		-0,3222				
VRDQPARQLVQRSEE		-0,3223				
GAPNRVSFAKLREPL		-0,3254				
NGVTLGYTSGTGQGN		-0,3256				
YPASDDYRASASNGS		-0,3264				
LSGIYNTSTLPLGTP		-0,3275				
LPDIIDGLWPPKLPD		-0,3277				
PGDSGGPVVNGLGQV		-0,3286				
AGAATEVELKERKHR		-0,3355				
GAAEKQDPLSGLTDQ		-0,3374				
LQELLTIKSDDTVGR		-0,3388				
DIASERTAPSRACQG		-0,3412				
TLPNRDGDVLVDADG		-0,3429				
TIDQLKTDAKLLSSI		-0,3452				
AGVLLRTERLKSQRV		-0,3502				
ASLVGFNRAPAGPSG		-0,3504				
VDMPYLTVELIEDLA		-0,3518				
QTSPANVVGTRQTLQ		-0,361				
LKTQIDQVESTAGSL		-0,362				
ARGANILARIMGASI		-0,3622				
NATNLRGILGRLIEM		-0,3629				

GGNRPAVYAPKSALG		-0,3672					
QTDFGGSDVPLSKDE		-0,3699					
NRANEVEAPMADPPT		-0,3754					
MLSSTSSDALRQTAR		-0,376					
IGSPLGLEGTVTTGI		-0,3771					
NALGGNRPAVYAPKS		-0,3814					
ARSQPNPKARQTIEG		-0,3825					
AGMAQDPVATAASNN		-0,3833					
LDELKLEGDEATGAN		-0,3906					
LAGCSSTKPVSQDTS		-0,3926					
LTDLFPILELGTSAK		-0,3946					
AMSNLLGQNAAAIAA		-0,395					
VGISQMHVSRLLAKS		-0,395					
FQDPSGGSRTVQVTL		-0,3974					
LGFAIPVDQAKRIAD		-0,3978					
VPVLRDELPGLGPLP		-0,3996					
PIAHSTVLVAPVSPS		-0,4013					
EVLADLDERGIVRIG		-0,4029					
TTTLVEHMVGILGQR		-0,4039					
VTQPDRVRELLATVF		-0,4067					
DEDDIKATYDKGILT		-0,4076					
FDAAIQPGDSGGPVV		-0,4109					
LLRGDDLVTEIPPDR		-0,4111					
ARPDVAVLDVRLPDG		-0,4136					
KQLNMAQIITSAGPD		-0,4148					
TRGLQHRGKLDGTPE		-0,4168					
IGPATIIPPIHIPSI		-0,4169					
AIPLTSATLGGLALP		-0,4189					
EVRAELPGVDPDKDV		-0,4217					
LERPVGDSSDCGTIR		-0,4233					
PEVDTNRLMVSIGTG		-0,4241					
VGLSTIAGRLLGSVP		-0,425					
LALNISVPDSSVPII		-0,4327					
TTLLALLAGVFGGAA		-0,4384					
NVARELSRCGSTGDR		-0,4395					
KFLSAATSSTPREAP		-0,4434					
STSAMPPAVADHVAA		-0,4585					
VDISGMFNVSTLGSA		-0,4619					
LDEHGTVLLAVRGLR		-0,4627					
NMAQIITSAGPDPVA		-0,4628					
VASHSPQVDPILDDL		-0,4652					
PDRVRELLATVFKLT		-0,4679					
PTNAAFDKLPAATID		-0,4691					
QGAVQGVNDALSGLG		-0,4719					
NMQRQAVPLVRSEAP		-0,4719					
LPSLPDIIDGLWPPK		-0,4763					
GFTIPGGTLIPQLPL		-0,4797					
AAKVVPSVVMLETDL		-0,4838					
LELPGIDVDKDVNVE		-0,4846					
AQEAELQGLLSCTLP		-0,4849					
LGADIASERTAPSRA		-0,4921					
DRQNAGDVAYRPMAP		-0,4924					
LPPAANDVSVLTAAR		-0,5001					
GYGVDIVLNSLTGAA		-0,5022					
VITSADVRASLERMA		-0,5023					
LTSAAERGPGQMLGG		-0,5073					
DGPCTDDGEMALGKN		-0,5112					
AGVDKVVADLTPQNQ		-0,5134					
EQVAAKVVPSVVMLE		-0,5221					
SADVRASLERMAPLV		-0,5231					
RAELMILIATNLLGQ		-0,5259					
PPITIDRIPLNLGAS		-0,5291					
VDMANPMSPVNKSFE		-0,5417					
PIDAGTAASQRGQEG		-0,5469					
SALLDPSQGMGPSLI		-0,5515					
IGKLLDNDKSPSRKT		-0,5561					

QQVSIAPNAGLDPVN		-0,5571				
QGLLSCTLPNRDGDV		-0,561				
DKPEKEKASVPGGGD		-0,5642				
TQQALAAGVPVIGLP		-0,5709				
YQAGKPTSTNPIASI		-0,5761				
AGADPTALNGMPAGL		-0,5782				
DAKQELIDSLEEAVR		-0,5811				
ILSDILSALAANADP		-0,5885				
LRVLDEIAPDLVVGD		-0,5894				
GPDGMPGGEVSILQP		-0,5913				
EIVKDGDDAVVRLEL		-0,5921				
PITLTIPGLSLDAAV		-0,6155				
LNALADAVKVTLGPK		-0,6202				
AANADPLTSGLLGIA		-0,6346				
ALACVLGECLTGAPP		-0,6376				
PPGILSDILSALAAN		-0,6464				
LIATNLLGQNTPAIA		-0,6471				
LENADLSLLGKARKV		-0,6647				
VKMLLNNVLEHLAEN		-0,6658				
VSGLGLKEAKDLVDG		-0,6661				
SVPQLVAAIKELQDK		-0,6786				
NLRGILGRLIEMLTT		-0,6892				
ATVALTFQDPSGGSR		-0,728				
DILSALAANADPLTS		-0,7457				
SVVMLETDLGRQSEE		-0,7471				
SVQDTPPNDQAGLEK		-0,7487				
IKGMELARAVKDVGA		-0,7558				
EKLQERLAKLAGGVA		-0,7585				
AGKPLLIIAEDVEGE		-0,7703				
STIAGRLLGSVPANV		-0,782				
DAAIGKLLDNDKSPS		-0,8085				
LIDGALKDLKKRVEG		-0,8086				
PVDALVNLADEEKAD		-0,8292				
MSGVLNAISDPLLGS		-0,8474				
GILGRLIEMLTTLPK		-0,9109				
TVELIEDLARRAVQT		-0,9572				
AEIVASVLEVVVNEG		-0,9657				
STVKDLLPLLEKVIG		-1,0346				
PLLEKVAKEAADEAK		-1,0988				
PELDVVGEAGSVAEA		0,1002				
NMREYLAAGAKERQR		0,0362				
AQDPVATAASNNPML		-0,0356				
LGGANIPAEFLENFV		-0,1184				
RPIVGAPVDALVNLA		-0,1383				
FVPQAVTPWPNGNDH		-0,1695				
IDTSNAVDTPDMLAS		-0,1783				
AAGVADPVKVTRSAL		-0,2049				
PLPTLPVPVLRDELP		-0,2552				
DTKLGAEEITRDIPN		-0,2644				
PLDDGDVIDSMFMSK		-0,2686				
VEERPIVGAPVDALV		-0,2848				
DGSAPIVHEMVSIPE		-0,3052				
AAGTTASPGAASGPK		-0,3347				
MAEMKTDAATLAQEA		-0,3455				
AVCAPTTGVLPPAAN		-0,3682				
KKELHSSGSTAQENA		-0,4079				
TQVLVPRSAIDSMLA		-0,4171				
DKLPAATIDQLKTDA		-0,4355				
SQGATVIDLSTSAMP		-0,4514				
VTIPTITTSPIPLKI		-0,455				
LSTDELLDAFKEMTL		-0,4623				
NGIELCRDLLSRMPD		-0,4827				
NLAELGRLTQLPDTN		-0,5231				
FVVTRQAQIVKPHDS		-0,5445				
NRVSFAKLREPLEVP		-0,5502				
AKNVEERPIVGAPVD		-0,2301				

IDLSTSAMPPAVADH		-0,4124				
DAMARLGAIKREVTS		-0,4153				
TLPVPVLRDELPGLG		-0,5232				
MNPDIEKDQTSDEVT		-0,5293				
AQQLRAQVMGDLDKL		-0,5766				
VLAAATGDLPTIGTA		-0,6067				
AAERGPGQMLGGLPV		-0,462				
DVQVTMPVPEPMVKM		-0,501				
GLREVADGDALYDAA		-0,2997				
ALAAGVPVIGLPSNM		-0,6886				

The top 100 recognized epitopes, defined by the index values, are listed with the respective MTB protein and accession number.
