# Supplementary material for: Pattern Recognition in Pulmonary Tuberculosis Defined by High Content Peptide Microarray Chip Analysis Representing 61 Proteins from M. tuberculosis
Source: PLoS One. 2008 Dec 9;3(12):e3840. doi: 10.1371/journal.pone.0003840 (PMC2588537; doi:10.1371/journal.pone.0003840)
Supplement: Table S6 — S6a: MTB Peptides recognized by both IgG and IgA, S6b: MTB Peptides recognized by IgG and not by IgA, S6c: MTB Peptides recognized by IgA and not by IgG. (0.75 MB PDF) [file pone.0003840.s006.pdf]

Supplementary Table 6a. Peptides recognized by both IgG and IgA

Peptide ID	Protein	IgA Average Index	IgG Average Index
WSRPVWDVEPWDRWL	CAA17343.1; HEAT SHOCK PROTEIN HSP	2.175	2.938
LALWSRPVWDVEPWD	CAA17343.1; HEAT SHOCK PROTEIN HSP	1.678	2.839
PVWDVEPWDRWLRDF	CAA17343.1; HEAT SHOCK PROTEIN HSP	1.961	2.760
WEYWGAQLNAMKGDL	CAB10044.1; SECRETED ANTIGEN 85-B FBPB (85B)	2.133	2.524
LADIAPMTPKVPYYS	MYCOBACTERIUM BOVIS MYCOCEROSIC ACID SYNTHASE GENE; M95808	0.439	2.343
GEMWAQDAAAMFGYA	CAE55371.1; PPE FAMILY PROTEIN	1.297	2.329
WSWELAIPMSGIAMA	CAB06237.1; CONSERVED HYPOTHETICAL PROTEIN	1.263	2.318
THSWEYWGAQLNAMK	CAB10044.1; SECRETED ANTIGEN 85-B FBPB (85B)	1.765	2.313
EYEQIWAQDVAAMFG	PPE FAMILY PROTEIN CAE55504	1.807	2.297
TNDGVIFFFNPGELL	CAA98382.1; IMMUNOGENIC PROTEIN MPT64	1.549	2.262
GTAVGTYNYMAPERF	TRANSMEMBRANE SERINE/THREONINE-PROTEIN KINASE D PKND NP_215446	1.138	2.259
EYAEMWAQDAAVMYS	PPE FAMILY PROTEIN CAE55334	1.035	2.249
DVEPWDRWLRDFFGP	CAA17343.1; HEAT SHOCK PROTEIN HSP	1.696	2.248
SAAIAATQAEYAEMW	PPE FAMILY PROTEIN CAE55334	0.868	2.156
DWDFNIRCFSNPALV	POSSIBLE GLYCOSYL TRANSFERASE CAB05419	1.584	2.102
DYITVMHDNGTRRTY	RNA POLYMERASE BETA-SUBUNIT AAA21416	0.661	2.078
MKAIADIEAYPQWIS	CONSERVED HYPOTHETICAL PROTEIN TB16.3CAD97060	0.893	2.072
EKIFGPTRDWECYCG	RNA POLYMERASE BETA'-SUBUNIT AAA21417	1.197	2.071
VGYMYIMKLHHLVDD	RNA POLYMERASE BETA-SUBUNIT AAA21416	1.209	2.062
YKAADMWGPSSDPAW	CAB10044.1; SECRETED ANTIGEN 85-B FBPB (85B)	1.969	2.032
MKLHHLVDDKIHARS	RNA POLYMERASE BETA-SUBUNIT AAA21416	1.148	2.030
LETHLGWCAHSGWKV	RNA POLYMERASE BETA-SUBUNIT AAA21416	0.850	2.027
GYVVKDIKGMELARA	TWO COMPONENT TRANSCRIPTIONAL REGULATORY PROTEIN DEVR NP_217649	0.015	2.020
QAQWNQAMEDLVRAY	CAA17363.1; LOW MOLECULAR WEIGHT PROTEIN ANTIGEN 7 ESXH	1.365	2.016
AQFGGQRFGEMECWA	RNA POLYMERASE BETA-SUBUNIT AAA21416	1.117	2.016
VAMNNGMEDLLIWEP	CAB06500.1; IMMUNOGENIC PROTEIN MPT63	0.670	1.991
GHLWSWELAIPMSGI	CAB06237.1; CONSERVED HYPOTHETICAL PROTEIN	1.085	1.953
EIVRAYEWAHHDMVP	PPE FAMILY PROTEIN CAE55489	0.818	1.951
FGEPSSGFSSMLWGG	PROBABLE CUTINASE PRECURSOR CFP21 NP_216500	0.725	1.950
FKDTLIMSYEWPEDR	CONSERVED HYPOTHETICAL PROTEIN TB16.3CAD97060	1.724	1.948
TGRSLDMSVAALAGM	MYCOBACTERIUM BOVIS MYCOCEROSIC ACID SYNTHASE GENE; M95808	0.030	1.942
DPAFTFMDYEQDWDG	MYCOBACTERIUM BOVIS ACYL-COA SYNTHASE GENE; U75685	0.966	1.913
PILGAPWLADLVRRS	MYCOBACTERIUM BOVIS MYCOCEROSIC ACID SYNTHASE GENE; M95808	1.277	1.899
PARWMHLMASDFHAF	MYCOBACTERIUM BOVIS ACYL-COA SYNTHASE GENE; U75685	0.958	1.888
HGLIGFGESYMAGEW	PUTATIVE CYCLOPROPANE-FATTY-ACYL-PHOSPHOLIPID SYNTHASE UFAA1 NP_854118	1.124	1.884
RAQVGKGMWTMTELM	MTB81	1.181	1.882
FWDEDGEVMRDYRLR	PPE FAMILY PROTEIN CAE55489	2.145	1.867
SYDDMRARGMKAVSP	3-OXOACYL-[ACYL-CARRIER PROTEIN] SYNTHASE 2 KASB CAA94642	0.253	1.860
NFPPNGTHSWEYWGA	CAB10044.1; SECRETED ANTIGEN 85-B FBPB (85B)	1.201	1.855
KGVPDWAARLPDELL	RNA POLYMERASE BETA-SUBUNIT AAA21416	1.152	1.827
VQAAMEDGYRVFADV	MYCOBACTERIUM BOVIS MYCOCEROSIC ACID SYNTHASE GENE; M95808	0.712	1.826
KAMLFDGRSGEPFPY	RNA POLYMERASE BETA-SUBUNIT AAA21416	0.862	1.813
AGGGSGFGNFGANMS	YP_177963; PPE FAMILY PROTEIN	0.482	1.811
VKAPGFGDRRKAMLQ	60 kDa chaperonin 2 P0A521	1.175	1.797
FTFMDYEQDWDGVAI	MYCOBACTERIUM BOVIS ACYL-COA SYNTHASE GENE; U75685	0.915	1.794
FGNFGANMSGWWNQA	YP_177963; PPE FAMILY PROTEIN	0.964	1.793
LCKTTSNFIWGQLLL	CAB06237.1; CONSERVED HYPOTHETICAL PROTEIN	1.555	1.788
PTIMGEVRRHFRDNS	ALTERNATE RNA POLYMERASE SIGMA FACTOR SIGF CAB07069	0.660	1.787
WPDPWPTTFGYHEFF	POSSIBLE HEMOLYSIN-LIKE PROTEIN CAA17201	0.876	1.782
GMYNFKESIRDFARA	CAA17111.1; PROBABLE ISOCITRATE DEHYDROGENASE	0.681	1.776
PNGTHSWEYWGAQLN	CAB10044.1; SECRETED ANTIGEN 85-B FBPB (85B)	1.400	1.775
RCGSPAWDLPTVFGP	PERIPLASMIC PHOSPHATE-BINDING LIPOPROTEIN PSTS2 YP_177769	0.447	1.757
NYPAMLGHAGDMAGY	CAA17363.1; LOW MOLECULAR WEIGHT PROTEIN ANTIGEN 7 ESXH	0.814	1.755
KWETFLTSELPQWLS	CAB10044.1; SECRETED ANTIGEN 85-B FBPB (85B)	0.477	1.727
EKIPLLGHLWSWELA	CAB06237.1; CONSERVED HYPOTHETICAL PROTEIN	1.225	1.725
WNQAMEDLVRAYHAM	CAA17363.1; LOW MOLECULAR WEIGHT PROTEIN ANTIGEN 7 ESXH	1.186	1.704
GLMVAAASPYVAWMS	CAE55371.1; PPE FAMILY PROTEIN	0.178	1.694
ITYQAWQAQWNQAME	CAA17363.1; LOW MOLECULAR WEIGHT PROTEIN ANTIGEN 7 ESXH	0.886	1.692

AQIADLDQEYDDFWD	PPE FAMILY PROTEIN CAE55489	0.833	1.691
WHRRRVIEPIDMDAY	MTB81	1.231	1.690
MTEQQWNFAGIEAAA	CAE55648	0.260	1.690
SMLWGGGSLPTIGPL	PROBABLE CUTINASE PRECURSOR CFP21 NP_216500	0.252	1.682
GPVLWSPAGKPPTWW	POSSIBLE GLYCOSYL TRANSFERASE CAB05415	0.919	1.651
MTAHTHDGTRTWRTG	CAB10947.1; PROBABLE LIPOPROTEIN LPRJ	1.910	1.645
QSSFYSDWYSPACGK	CAB10044.1; SECRETED ANTIGEN 85-B FBPB (85B)	1.352	1.631
GLATAEDIRQWSYGE	RNA POLYMERASE BETA'-SUBUNIT AAA21417	0.872	1.611
MASGSGLCKTTSNFI	CAB06237.1; CONSERVED HYPOTHETICAL PROTEIN	0.748	1.609
CAMFTDLLAQPTPAW	PUTATIVE CYCLOPROPANE-FATTY-ACYL-PHOSPHOLIPID SYNTHASE UFAA1 NP_854118	0.793	1.593
VSYGSVVSTLIVEPW	PPE FAMILY PROTEIN CAE55334	0.721	1.591
KTSTYALTNATMPYV	SECRETED L-ALANINE DEHYDROGENASE ALD CAA15575	-0.024	1.589
VIPTMTPPPGMVRQR	CAA17582.1; PROBABLE SERINE PROTEASE PEPD	-0.017	1.588
ATFEMRYLQSLPAHL	PPE FAMILY PROTEIN CAE55504	0.497	1.581
PYNHMEVGSPYAVEY	MCE-FAMILY PROTEIN MCE1AYP_177701	0.476	1.580
KYGGTEIKYNGEEYL	CAB01005.1; 10 KDA CHAPERONIN GROES	1.369	1.567
KALCDFYEEQMQDAF	PROBABLE ISOCITRATE DEHYDROGENASE CAA16247	1.112	1.564
VAHMRHGDFYAGEKS	PROBABLE ISOCITRATE DEHYDROGENASE CAA16247	0.953	1.557
HPTTTYKAFDWDQAY	CAA98382.1; IMMUNOGENIC PROTEIN MPT64	0.831	1.553
GGTSDTVRDLIARWE	MYCOBACTERIUM BOVIS MYCOCEROSIC ACID SYNTHASE GENE; M95808	0.811	1.546
KAFDWDQAYRKPITY	CAA98382.1; IMMUNOGENIC PROTEIN MPT64	0.631	1.535
EMECWAMQAYGAAYT	RNA POLYMERASE BETA-SUBUNIT AAA21416	0.593	1.530
GLEVRDVHPSHYGRM	RNA POLYMERASE BETA-SUBUNIT AAA21416	0.586	1.528
HPHSMGEWSMASRTH	PROBABLE ISOCITRATE DEHYDROGENASE CAA16247	1.341	1.509
DGDVIDSMFMSKKAL	PROBABLE ISOCITRATE DEHYDROGENASE CAA16247	0.273	1.507
RDFFGPAATTDWYRP	CAA17343.1; HEAT SHOCK PROTEIN HSP	0.775	1.507
MSHQDWQAQWNQAME	CAA16102.1; ESAT-6 LIKE PROTEIN ESXQ	0.876	1.504
FGANMSGWWNQAHTA	YP_177963; PPE FAMILY PROTEIN	0.648	1.503
AIAVNEAEYGEMWAQ	CAE55371.1; PPE FAMILY PROTEIN	0.579	1.496
EYAARFITPVHPGYT	PPE FAMILY PROTEIN CAE55504	0.922	1.494
ADMWGPSSDPAWERN	CAB10044.1; SECRETED ANTIGEN 85-B FBPB (85B)	1.161	1.493
LGPVLWSPDVKPPTW	POSSIBLE GLYCOSYL TRANSFERASE CAB05418	0.893	1.483
AAIFKDTLIMSYEWP	CONSERVED HYPOTHETICAL PROTEIN TB16.3CAD97060	0.612	1.482
GVLWRGDYEGLWGLS	YP_177963; PPE FAMILY PROTEIN	0.832	1.477
MTPKVPYYSATLFDP	MYCOBACTERIUM BOVIS MYCOCEROSIC ACID SYNTHASE GENE; M95808	0.188	1.477
LHAWSDVDVSEWPGI	HYPOTHETICAL PROTEIN RV2623 NP_217139	0.768	1.472
WYYQSGLSIVMPVGG	CAB10044.1; SECRETED ANTIGEN 85-B FBPB (85B)	-0.096	1.465
TAGAFSRPGLPVEYL	CAB10044.1; SECRETED ANTIGEN 85-B FBPB (85B)	0.407	1.458
IGFGESYMAGEWSSK	PUTATIVE CYCLOPROPANE-FATTY-ACYL-PHOSPHOLIPID SYNTHASE UFAA1 NP_854118	1.010	1.450
ALMSGNFSNGILWRG	YP_177963; PPE FAMILY PROTEIN	0.723	1.450
GSQFYLAMYWAQELA	PROBABLE ISOCITRATE DEHYDROGENASE CAA16247	1.044	1.438
LVCGGVHTANATVYM	CAA98350.1; CELL SURFACE LIPOPROTEIN MPT83	0.526	1.433
YRADSVERLIAAHLM	TRANSMEMBRANE SERINE/THREONINE-PROTEIN KINASE D PKND NP_215446	0.409	1.432
LPLLEKVIGAGKPLL	60 kDa chaperonin 2 P0A521	-0.455	1.429
SMFMSKKALCDFYEE	PROBABLE ISOCITRATE DEHYDROGENASE CAA16247	1.043	1.427
KRAMLRDMGVEHVYD	MYCOBACTERIUM BOVIS MYCOCEROSIC ACID SYNTHASE GENE; M95808	0.947	1.425
FCGRIHTRYSSAYEL	SECRETED L-ALANINE DEHYDROGENASE ALD CAA15575	0.583	1.422
FAAFLDETMTYSCAM	PUTATIVE CYCLOPROPANE-FATTY-ACYL-PHOSPHOLIPID SYNTHASE UFAA1 NP_854118	0.128	1.422
DLPSFPGFPGFPEFP	CAB06237.1; CONSERVED HYPOTHETICAL PROTEIN	0.539	1.414
LWSPAGKPPTWWDSL	POSSIBLE GLYCOSYL TRANSFERASE CAB05415	0.917	1.412
IAPMTPKVPYYSATL	MYCOBACTERIUM BOVIS MYCOCEROSIC ACID SYNTHASE GENE; M95808	0.185	1.397
TLIMSYEWPEDRQSL	CONSERVED HYPOTHETICAL PROTEIN TB16.3CAD97060	0.779	1.393
FYSDWYSPACGKAGC	CAB10044.1; SECRETED ANTIGEN 85-B FBPB (85B)	0.899	1.376
CEASLDQQRQWILHM	MTB48 AAK31576	0.639	1.376
HMEVGSPYAVEYVWG	MCE-FAMILY PROTEIN MCE1AYP_177701	0.297	1.370
HLGWCAHSGWKVDAA	RNA POLYMERASE BETA-SUBUNIT AAA21416	0.311	1.361
TPPAPAPPPFRLPLL	YP_177963; PPE FAMILY PROTEIN	1.133	1.357
CWQPITRDLWPAPYL	MCE-FAMILY PROTEIN MCE1AYP_177701	0.812	1.356
SGFGNFGAGSSGWWN	YP_177963; PPE FAMILY PROTEIN	0.767	1.350
ILRRTIDADRSFIEY	MYCOBACTERIUM BOVIS MYCOCEROSIC ACID SYNTHASE GENE; M95808	0.227	1.338
SPHRSPISNMVSMAN	CAE55371.1; PPE FAMILY PROTEIN	-0.095	1.337
FDPREQPVCDGAYWV	MYCOBACTERIUM BOVIS MYCOCEROSIC ACID SYNTHASE GENE; M95808	0.438	1.333

AIMPWEGHNYEDAII	RNA POLYMERASE BETA-SUBUNIT AAA21416	0.534	1.329
HWLRPITPTFRPSWP	PUTATIVE CYCLOPROPANE-FATTY-ACYL-PHOSPHOLIPID SYNTHASE UFAA1 NP_854118	0.649	1.328
FLDETMTYSCAMFTD	PUTATIVE CYCLOPROPANE-FATTY-ACYL-PHOSPHOLIPID SYNTHASE UFAA1 NP_854118	0.650	1.328
PGPDSMLAAARAWRS	PPE FAMILY PROTEIN CAE55489	0.564	1.318
MRHGDFYAGEKSMTL	PROBABLE ISOCITRATE DEHYDROGENASE CAA16247	0.560	1.317
QIHEIVRAYEWAHHD	PPE FAMILY PROTEIN CAE55489	0.536	1.315
GPWHRGGWDPIPEGL	POSSIBLE HEMOLYSIN CAA16235	1.023	1.312
MNIGQILETHLGWCA	RNA POLYMERASE BETA-SUBUNIT AAA21416	0.935	1.298
VGTYNYMAPERFTGD	TRANSMEMBRANE SERINE/THREONINE-PROTEIN KINASE D PKND NP_215446	0.882	1.289
LFPDLPSFPGFPGFP	CAB06237.1; CONSERVED HYPOTHETICAL PROTEIN	0.430	1.285
YYSATLFDPREQPVC	MYCOBACTERIUM BOVIS MYCOCEROSIC ACID SYNTHASE GENE; M95808	0.336	1.285
AAAVQAAMEDGYRVF	MYCOBACTERIUM BOVIS MYCOCEROSIC ACID SYNTHASE GENE; M95808	0.558	1.282
DEIVYLTADEEDRHV	RNA POLYMERASE BETA-SUBUNIT AAA21416	0.728	1.277
TALLQAHPHAVNGTA	MYCOBACTERIUM BOVIS MYCOCEROSIC ACID SYNTHASE GENE; M95808	0.645	1.276
RWLRDFFGPAATTDW	CAA17343.1; HEAT SHOCK PROTEIN HSP	0.519	1.272
GYIPILGAPWLADLV	MYCOBACTERIUM BOVIS MYCOCEROSIC ACID SYNTHASE GENE; M95808	0.838	1.270
WGGAFDLDRLLFKRN	POSSIBLE GLYCOSYL TRANSFERASE CAB05419	0.373	1.269
RNTVQFAAAVQAAME	MYCOBACTERIUM BOVIS MYCOCEROSIC ACID SYNTHASE GENE; M95808	0.400	1.268
AMRTLRKYVEADLRV	POSSIBLE GLYCOSYL TRANSFERASE CAB05415	0.837	1.266
MYETGAGGSAPKHVK	PROBABLE ISOCITRATE DEHYDROGENASE CAA16247	0.853	1.262
ENVEAGDIWRMCIVK	PROBABLE ISOCITRATE DEHYDROGENASE CAA16247	1.039	1.257
DAEVVLPWDGRNHYL	CAA16030.1; PROBABLE MOLYBDOPTERIN-GUANINE DINUCLEOTIDE BIOSYNTHESIS PROTEIN	0.405	1.254
SMAEQVLAERLAGWQ	HYPOTHETICAL PROTEIN RV2623 NP_217139	0.867	1.253
LAHLGFDEVFARMWE	PUTATIVE CYCLOPROPANE-FATTY-ACYL-PHOSPHOLIPID SYNTHASE UFAA1 NP_854118	0.489	1.253
GSSAMILAAYHPQQF	CAB10044.1; SECRETED ANTIGEN 85-B FBPB (85B)	0.829	1.246
ARPMTELVTGKAFPY	3-OXOACYL-[ACYL-CARRIER PROTEIN] SYNTHASE 2 KASB CAA94642	0.333	1.240
LMWAADVAAMAGYHS	YP_177963; PPE FAMILY PROTEIN	0.917	1.240
AMMAHAGDMAGYAGT	CAA16104.1; SECRETED ESAT-6 LIKE PROTEIN ESXR	0.567	1.236
RACACLQPHDPAFTF	MYCOBACTERIUM BOVIS ACYL-COA SYNTHASE GENE; U75685	0.627	1.236
ANEYADVPEMFRELV	ALTERNATE RNA POLYMERASE SIGMA FACTOR SIGF CAB07069	1.026	1.236
DTGITYQGWQTQWNQ	CAA16104.1; SECRETED ESAT-6 LIKE PROTEIN ESXR	0.462	1.229
APGCWQPITRDLWPA	MCE-FAMILY PROTEIN MCE1AYP_177701	0.943	1.229
QAQWNQAMEALARAY	CAA16102.1; ESAT-6 LIKE PROTEIN ESXQ	-0.016	1.227
PWDRWLRDFFGPAAT	CAA17343.1; HEAT SHOCK PROTEIN HSP	1.331	1.224
MEQFVYAYVRSCPGY	PERIPLASMIC PHOSPHATE-BINDING LIPOPROTEIN PSTS2 YP_177769	0.276	1.223
PLLGHLWSWELAIPM	CAB06237.1; CONSERVED HYPOTHETICAL PROTEIN	0.814	1.216
LSPLRPASCRFVPTC	POSSIBLE HEMOLYSIN CAA16235	0.083	1.215
LLTENVEAGDIWRMC	PROBABLE ISOCITRATE DEHYDROGENASE CAA16247	0.399	1.214
CDFYEEQMQDAFETG	PROBABLE ISOCITRATE DEHYDROGENASE CAA16247	0.288	1.213
GGQVISEEVGLTLEN	60 kDa chaperonin 2 P0A521	0.788	1.213
MADPPTDVPITPCEL	MTB48 AAK31576	-0.008	1.203
QAWQAQWNQAMEDLV	CAA17363.1; LOW MOLECULAR WEIGHT PROTEIN ANTIGEN 7 ESXH	0.741	1.199
YEEQMQDAFETGVMF	PROBABLE ISOCITRATE DEHYDROGENASE CAA16247	0.622	1.197
YDDFWDEDGEVMRDY	PPE FAMILY PROTEIN CAE55489	0.636	1.189
LNVMEEHQVRRVPVI	HYPOTHETICAL PROTEIN RV2626C NP_217142	-0.015	1.188
AVRWPDPWPTTFGYH	POSSIBLE HEMOLYSIN-LIKE PROTEIN CAA17201	0.770	1.188
AACLKWEGGYVWACK	CAA17111.1; PROBABLE ISOCITRATE DEHYDROGENASE	1.008	1.187
SFAKLREPLEVPGLL	RNA POLYMERASE BETA-SUBUNIT AAA21416	0.599	1.184
TSEQIVERFGFSEIM	RNA POLYMERASE BETA-SUBUNIT AAA21416	0.201	1.174
TDSFEWLIGSPRWRE	RNA POLYMERASE BETA-SUBUNIT AAA21416	0.779	1.173
PKVISRAMVMRTVIS	POSSIBLE GLYCOSYL TRANSFERASE CAB05419	-0.017	1.170
PLPDVPWTRFFGVRP	POSSIBLE GLYCOSYL TRANSFERASE CAB05418	0.472	1.170
DSMLAAARAWRSLDV	PPE FAMILY PROTEIN CAE55489	0.702	1.168
DWDQAYRKPITYDTL	CAA98382.1; IMMUNOGENIC PROTEIN MPT64	0.222	1.167
YCPAVIAPLMPNRLQ	CAB10947.1; PROBABLE LIPOPROTEIN LPRJ	0.191	1.162
VLADWDFNIRCFSNP	POSSIBLE GLYCOSYL TRANSFERASE CAB05419	1.120	1.161
GLAMGDAGGYKAADM	CAB10044.1; SECRETED ANTIGEN 85-B FBPB (85B)	0.415	1.158
DMTTAVMRPSKTFNA	PROBABLE ISOCITRATE DEHYDROGENASE CAA16247	0.101	1.152
PEFQRHRDKIVQRCL	ALTERNATE RNA POLYMERASE SIGMA FACTOR SIGF CAB07069	0.410	1.152
CTHYPLAEAADAIRA	MYCOBACTERIUM BOVIS MYCOCEROSIC ACID SYNTHASE GENE; M95808	0.612	1.147
VERQIHEIVRAYEWA	PPE FAMILY PROTEIN CAE55489	0.718	1.144
FFVEMRMIDGTSLRA	TRANSMEMBRANE SERINE/THREONINE-PROTEIN KINASE D PKND	0.016	1.141

	NP_215446		
LFNLWGPAFHERYPN	PERIPLASMIC PHOSPHATE-BINDING LIPOPROTEIN PSTS1 YP_177770	0.609	1.140
PEQDWLNSEEFLDAI	CAA17111.1; PROBABLE ISOCITRATE DEHYDROGENASE	0.211	1.140
QGASLAHFADGWNTF	MTB48 AAK31576	0.787	1.137
AMAAAATPYVGWLAA	PPE FAMILY PROTEIN CAE55334	0.153	1.137
PKVFGSWNLHRATLG	MYCOBACTERIUM BOVIS MYCOCEROSIC ACID SYNTHASE GENE; M95808	0.878	1.136
LMEAAKPFVRWLTDL	PPE FAMILY PROTEIN CAE55489	0.281	1.135
ITPEEGAYAFETLVR	MYCOBACTERIUM BOVIS MYCOCEROSIC ACID SYNTHASE GENE; M95808	0.322	1.134
EGTYIHAVYYPGENQ	CONSERVED HYPOTHETICAL PROTEIN TB18.5CAD93033	0.473	1.132
ENAMEQFVYAYVRSC	PERIPLASMIC PHOSPHATE-BINDING LIPOPROTEIN PSTS2 YP_177769	0.158	1.132
GFGNATATPSSGFFN	YP_177963; PPE FAMILY PROTEIN	0.041	1.131
MWLSPNGTIRNILGG	CAA17111.1; PROBABLE ISOCITRATE DEHYDROGENASE	0.245	1.126
LRLMPENRPRTWLAS	POSSIBLE GLYCOSYL TRANSFERASE CAB05415	0.190	1.125
YADVPEMFRELVGLP	ALTERNATE RNA POLYMERASE SIGMA FACTOR SIGF CAB07069	0.021	1.119
GPRKSALDEEEIWDL	CONSERVED HYPOTHETICAL PROTEIN CAB08634	0.665	1.119
LGLPPPWQPALPRLF	PPE FAMILY PROTEIN CAE55504	0.840	1.114
AVFNFPPNGTHSWEY	CAB10044.1; SECRETED ANTIGEN 85-B FBPB (85B)	0.451	1.113
LPDELSDVETGLFVP	MYCOBACTERIUM BOVIS MYCOCEROSIC ACID SYNTHASE GENE; M95808	-0.252	1.104
GFGNFGAGSSGWWNQ	YP_177963; PPE FAMILY PROTEIN	0.707	1.101
VLWSPDVKPPTWWHS	POSSIBLE GLYCOSYL TRANSFERASE CAB05418	0.871	1.096
VANNTRLWVYCGNGT	CAB10044.1; SECRETED ANTIGEN 85-B FBPB (85B)	0.625	1.095
PVTHIWYFKGVPSRL	RNA POLYMERASE BETA'-SUBUNIT AAA21417	0.686	1.092
SGNFSNGVLWRGDYE	YP_177963; PPE FAMILY PROTEIN	0.936	1.091
IRLAKCGPWHRGGWD	POSSIBLE HEMOLYSIN CAA16235	0.901	1.088
VHPSHYGRMCPIETP	RNA POLYMERASE BETA-SUBUNIT AAA21416	0.195	1.086
PFGLFPDVDWAEVAA	PPE FAMILY PROTEIN CAE55504	-0.067	1.082
KTVSPHRSPISNMVS	CAE55371.1; PPE FAMILY PROTEIN	-0.165	1.082
PWPTTFGYHEFFHAC	POSSIBLE HEMOLYSIN-LIKE PROTEIN CAA17201	0.829	1.081
QTQWNQALEDLVRAY	CAA16104.1; SECRETED ESAT-6 LIKE PROTEIN ESXR	0.555	1.074
VLFWLDPYRPHENEL	PROBABLE ISOCITRATE DEHYDROGENASE CAA16247	0.642	1.073
NLLGQNAPAIAAAEA	PPE FAMILY PROTEIN CAE55504	-0.127	1.070
WTELAALPDFLGGFA	CAB06237.1; CONSERVED HYPOTHETICAL PROTEIN	0.500	1.064
ATMPYVLELADHGWR	SECRETED L-ALANINE DEHYDROGENASE ALD CAA15575	0.460	1.063
ERFGFSEIMRSTLEK	RNA POLYMERASE BETA-SUBUNIT AAA21416	0.482	1.063
SPTAATLHALHYHQV	MTB81 (publication)	0.502	1.062
ELMPTYDLPANHEYL	POSSIBLE GLYCOSYL TRANSFERASE CAB05415	0.818	1.061
GRGGMGEVYEAEDTR	TRANSMEMBRANE SERINE/THREONINE-PROTEIN KINASE D PKND NP_215446	0.351	1.059
PTWWHSLPTDRPIIY	POSSIBLE GLYCOSYL TRANSFERASE CAB05418	0.683	1.057
QQEDEALYTEDRAWT	MTB48 AAK31576	0.501	1.055
GAPWLADLVRRSPWG	MYCOBACTERIUM BOVIS MYCOCEROSIC ACID SYNTHASE GENE; M95808	0.505	1.053
YNLPANHRYLGPVLW	POSSIBLE GLYCOSYL TRANSFERASE CAB05418	0.578	1.050
WVFSGQGSQWAAMGT	MYCOBACTERIUM BOVIS MYCOCEROSIC ACID SYNTHASE GENE; M95808	0.479	1.049
GVSGFGNFGAGSSGW	YP_177963; PPE FAMILY PROTEIN	0.298	1.044
IADIEAYPQWISEYK	CONSERVED HYPOTHETICAL PROTEIN TB16.3CAD97060	0.681	1.042
PSSGFSSMLWGGGSL	PROBABLE CUTINASE PRECURSOR CFP21 NP_216500	0.448	1.038
MAPERFTGDEVTYRA	TRANSMEMBRANE SERINE/THREONINE-PROTEIN KINASE D PKND NP_215446	0.332	1.036
RSFNRTLLSLMDAWA	PPE FAMILY PROTEIN CAE55489	0.623	1.036
TQAEYAEMWAQDAAV	PPE FAMILY PROTEIN CAE55334	-0.096	1.035
ARKHPHSMGEWSMAS	PROBABLE ISOCITRATE DEHYDROGENASE CAA16247	1.139	1.032
AVAVGCAQPACLRAY	MYCOBACTERIUM BOVIS MYCOCEROSIC ACID SYNTHASE GENE; M95808	0.086	1.031
FGALPPEINSARMYA	CAE55371.1; PPE FAMILY PROTEIN	0.265	1.027
DVPWTRFFGVRPVSI	POSSIBLE GLYCOSYL TRANSFERASE CAB05418	0.438	1.025
RAYEWAHHDMVPLAQ	PPE FAMILY PROTEIN CAE55489	0.730	1.021
PTCSQYAVDALTEYG	POSSIBLE HEMOLYSIN CAA16235	0.544	1.020
GGGMYETGAGGSAPK	PROBABLE ISOCITRATE DEHYDROGENASE CAA16247	0.825	1.020
MTLDRARNVRMELLA	PROBABLE ISOCITRATE DEHYDROGENASE CAA16247	-0.064	1.020
DNRGSQFYLAMYWAQ	PROBABLE ISOCITRATE DEHYDROGENASE CAA16247	0.894	1.019
ATACEASLDQQRQWI	MTB48 AAK31576	0.575	1.017
AAAIAAVEAEYELMW	YP_177963; PPE FAMILY PROTEIN	0.352	1.015
DVKRFRGFDNWEGDA	MTB48 AAK31576	0.767	1.015
ANHEYLGPVLWSPAG	POSSIBLE GLYCOSYL TRANSFERASE CAB05415	0.751	1.013
YPLAEAADAIRAMSN	MYCOBACTERIUM BOVIS MYCOCEROSIC ACID SYNTHASE GENE; M95808	0.870	1.011
VGGQSSFYSDWYSPA	CAB10044.1; SECRETED ANTIGEN 85-B FBPB (85B)	0.448	1.010
MTDTVGQVVLGWKVS	CAB06500.1; IMMUNOGENIC PROTEIN MPT63	-0.044	1.009

GFPGFPEFPAIPGFP	CAB06237.1; CONSERVED HYPOTHETICAL PROTEIN	0.252	1.009
GSPLFPDLPSFPGFP	CAB06237.1; CONSERVED HYPOTHETICAL PROTEIN	0.208	1.008
AEYGEMWAQDAAAMF	CAE55371.1; PPE FAMILY PROTEIN	0.504	1.008
KAYDGMFKDEFERVY	CAA17111.1; PROBABLE ISOCITRATE DEHYDROGENASE	0.806	1.008
AGSVDELVPRSLHWL	PUTATIVE CYCLOPROPANE-FATTY-ACYL-PHOSPHOLIPID SYNTHASE UFAA1 NP_854118	0.537	1.005
ATLFDPREQPVCDGA	MYCOBACTERIUM BOVIS MYCOCEROSIC ACID SYNTHASE GENE; M95808	0.538	1.005
PGQGAYAAANSWVDV	MYCOBACTERIUM BOVIS MYCOCEROSIC ACID SYNTHASE GENE; M95808	0.684	1.003
GVRLAFVCAVDMPYL	CAA16030.1; PROBABLE MOLYBDOPTERIN-GUANINE DINUCLEOTIDE BIOSYNTHESIS PROTEIN	0.210	0.998
EHRLIDDMVAACLKW	CAA17111.1; PROBABLE ISOCITRATE DEHYDROGENASE	0.485	0.996
DEARVEEFNLKKMWL	CAA17111.1; PROBABLE ISOCITRATE DEHYDROGENASE	0.881	0.995
VRRRHGLSSLGWNLC	POSSIBLE GLYCOSYL TRANSFERASE CAB05415	0.850	0.995
PVAVIGMGCRLPGGI	MYCOBACTERIUM BOVIS MYCOCEROSIC ACID SYNTHASE GENE; M95808	0.039	0.994
WRSLDVEMTAVQRSF	PPE FAMILY PROTEIN CAE55489	0.771	0.994
FAAFPSFAGLRPTFD	CAA17245.1; HEAT SHOCK PROTEIN HSPX	-0.179	0.991
EWAHHDMVPLAQIYN	PPE FAMILY PROTEIN CAE55489	0.640	0.991
ATWLEVPLPPGVLRW	HYPOTHETICAL PROTEIN RV2623 NP_217139	0.276	0.988
LIGPEQDWLNSEEFL	CAA17111.1; PROBABLE ISOCITRATE DEHYDROGENASE	0.866	0.987
AIANAYWSPQARRRF	POSSIBLE GLYCOSYL TRANSFERASE CAB05418	0.344	0.987
DIRQWSYGEVKKPET	RNA POLYMERASE BETA'-SUBUNIT AAA21417	0.162	0.983
FEWLIGSPRWRESAA	RNA POLYMERASE BETA-SUBUNIT AAA21416	0.545	0.972
VVLPWDGRNHYLAAV	CAA16030.1; PROBABLE MOLYBDOPTERIN-GUANINE DINUCLEOTIDE BIOSYNTHESIS PROTEIN	0.128	0.968
IAATQAEYAEMWAQD	PPE FAMILY PROTEIN CAE55334	0.674	0.966
QPHDPAFTFMDYEQD	MYCOBACTERIUM BOVIS ACYL-COA SYNTHASE GENE; U75685	0.558	0.965
AMAAAAAPYAGWLGS	YP_177963; PPE FAMILY PROTEIN	0.247	0.963
FRMELLSLPQDEWAG	MYCOBACTERIUM BOVIS MYCOCEROSIC ACID SYNTHASE GENE; M95808	0.682	0.958
EGLTNKQIADRMFLA	TWO COMPONENT TRANSCRIPTIONAL REGULATORY PROTEIN DEVR NP_217649	-0.050	0.957
AKCGPWHRGGWDPIP	POSSIBLE HEMOLYSIN CAA16235	0.528	0.947
LMASDFHAFSAAPNF	MYCOBACTERIUM BOVIS ACYL-COA SYNTHASE GENE; U75685	0.398	0.946
NNMAQTDSAVGSSWA	PUTATIVE ESAT-6 LIKE PROTEIN ESXN YP_177838	0.208	0.943
GVYKVCKGLEKIPLL	CAB06237.1; CONSERVED HYPOTHETICAL PROTEIN	0.164	0.940
EGVTGHQVGDRVGGF	MYCOBACTERIUM BOVIS MYCOCEROSIC ACID SYNTHASE GENE; M95808	0.481	0.934
MSTVLSRRLWENAGS	3-OXOACYL-[ACYL-CARRIER PROTEIN] SYNTHASE 2 KASB CAA94642	0.278	0.928
TDLCVQLSEVERQIH	PPE FAMILY PROTEIN CAE55489	0.673	0.927
AVPQVGSQFGPYQLL	TRANSMEMBRANE SERINE/THREONINE-PROTEIN KINASE D PKND NP_215446	0.415	0.926
WRGDYEGLWGLSGGS	YP_177963; PPE FAMILY PROTEIN	0.615	0.919
MRLEDEMKEGRYEVR	CAA17245.1; HEAT SHOCK PROTEIN HSPX	0.127	0.915
RYRVLADWDFNIRCF	POSSIBLE GLYCOSYL TRANSFERASE CAB05419	1.286	0.915
QIWAQDVAAMFGYHS	PPE FAMILY PROTEIN CAE55504	0.270	0.912
NARTADGINYRVLWQ	CAB06500.1; IMMUNOGENIC PROTEIN MPT63	0.402	0.909
YAQGQQQTYSQQFDW	CAA17582.1; PROBABLE SERINE PROTEASE PEPD	0.794	0.908
GFPEFPAIPGFPALP	CAB06237.1; CONSERVED HYPOTHETICAL PROTEIN	-0.014	0.904
HPTYEDIVGLERLYA	MTB48 AAK31576	0.085	0.900
SPYAQRRFPLPDVIW	POSSIBLE GLYCOSYL TRANSFERASE CAB05415	0.591	0.896
HDYGEINGQFFVEMR	TRANSMEMBRANE SERINE/THREONINE-PROTEIN KINASE D PKND NP_215446	0.239	0.894
RIFYKDAFAKHQELF	PROBABLE ISOCITRATE DEHYDROGENASE CAA16247	0.972	0.891
LAAPVTHIWYFKGVP	RNA POLYMERASE BETA'-SUBUNIT AAA21417	0.321	0.891
QAITAATAHATAWYG	MYCOBACTERIUM BOVIS MYCOCEROSIC ACID SYNTHASE GENE; M95808	0.501	0.890
QAQYVAQLHVWARRE	MTB48 AAK31576	0.265	0.888
DEALYTEDRAWTEAV	MTB48 AAK31576	0.270	0.885
LQGDVKRFRGFDNWE	MTB48 AAK31576	0.707	0.884
SMYSYPAMTANVGDM	CAA16102.1; ESAT-6 LIKE PROTEIN ESXQ	0.107	0.884
PSKFRMELLSLPQDE	MYCOBACTERIUM BOVIS MYCOCEROSIC ACID SYNTHASE GENE; M95808	0.908	0.884
IDMDAYRQFLTEIGY	MTB81 (publication)	-0.107	0.883
GPVVMQLMEAAKPFV	PPE FAMILY PROTEIN CAE55489	-0.490	0.883
IHELVNIGPFRIEPI	YP_177963; PPE FAMILY PROTEIN	-0.067	0.882
LNGKVLAAMYQGTIK	PERIPLASMIC PHOSPHATE-BINDING LIPOPROTEIN PSTS1 YP_177770	0.270	0.876
FACDPRFNKLLGPLP	POSSIBLE GLYCOSYL TRANSFERASE CAB05418	0.204	0.873
DWYSPACGKAGCQTY	CAB10044.1; SECRETED ANTIGEN 85-B FBPB (85B)	0.388	0.872
QGWQTQWNQALEDLV	CAA16104.1; SECRETED ESAT-6 LIKE PROTEIN ESXR	0.262	0.871
LVVKRGPNAGSRFLL	CAB01474.1; CONSERVED HYPOTHETICAL PROTEIN CFP17	0.932	0.869
LVALHAWSDVDVSEW	HYPOTHETICAL PROTEIN RV2623 NP_217139	0.460	0.868

PVIGLPSNMDQHLNM	POSSIBLE GLYCOSYL TRANSFERASE CAB05418	0.275	0.866
ITYQGWQTQWNQALE	CAA16104.1; SECRETED ESAT-6 LIKE PROTEIN ESXR	0.701	0.866
TLTDALKSHGPQGTE	MYCOBACTERIUM BOVIS MYCOCEROSIC ACID SYNTHASE GENE; M95808	0.732	0.863
HPIVFGHAVRIFYKD	PROBABLE ISOCITRATE DEHYDROGENASE CAA16247	0.203	0.863
AQDVAAMFGYHSAAS	PPE FAMILY PROTEIN CAE55504	0.051	0.859
DIPGMSLFDIHELVN	YP_177963; PPE FAMILY PROTEIN	0.592	0.856
KIHARSTGPYSMITQ	RNA POLYMERASE BETA-SUBUNIT AAA21416	-0.161	0.855
IAAVEAEYELMWAAD	YP_177963; PPE FAMILY PROTEIN	0.296	0.854
QGIEGTYIHAVYYPG	CONSERVED HYPOTHETICAL PROTEIN TB18.5CAD93033	0.046	0.853
AQQLVLSADNMREYL	MTB48 AAK31576	0.181	0.848
KDLVDGAPKPLLEKV	CAB07109.1; PROBABLE 50S RIBOSOMAL PROTEIN L7/L12 RPLL	-0.182	0.842
YQEIINFCKTNGQFD	PROBABLE ISOCITRATE DEHYDROGENASE CAA16247	0.674	0.839
KPEKDGLFCEKIFGP	RNA POLYMERASE BETA'-SUBUNIT AAA21417	0.518	0.839
VWYTATILHNAGVTA	POSSIBLE HEMOLYSIN-LIKE PROTEIN CAA17201	-0.086	0.839
EIMITPEEGAYAFET	MYCOBACTERIUM BOVIS MYCOCEROSIC ACID SYNTHASE GENE; M95808	0.222	0.838
RRFPLPDVPWTRFFG	POSSIBLE GLYCOSYL TRANSFERASE CAB05418	0.709	0.838
DWLNSEEFLDAIADN	CAA17111.1; PROBABLE ISOCITRATE DEHYDROGENASE	-0.052	0.827
TAVQGIEGTYIHAVY	CONSERVED HYPOTHETICAL PROTEIN TB18.5CAD93033	-0.084	0.825
LTTYILLPSQDLPLL	PPE FAMILY PROTEIN CAE55504	0.064	0.823
AFEWYYQSGLSIVMP	CAB10044.1; SECRETED ANTIGEN 85-B FBPB (85B)	-0.019	0.821
PDFKWPPTPGSPLFP	CAB06237.1; CONSERVED HYPOTHETICAL PROTEIN	-0.017	0.820
NTPAFEWYYQSGLSI	CAB10044.1; SECRETED ANTIGEN 85-B FBPB (85B)	0.288	0.819
ADLDQEYDDFWDEDG	PPE FAMILY PROTEIN CAE55489	0.811	0.815
QQRQWILHMAKLSAA	MTB48 AAK31576	-0.126	0.814
IGRHGLIGFGESYMA	PUTATIVE CYCLOPROPANE-FATTY-ACYL-PHOSPHOLIPID SYNTHASE UFAA1 NP_854118	0.209	0.809
SDIFLDDVTVSRRHA	CAB01474.1; CONSERVED HYPOTHETICAL PROTEIN CFP17	-0.048	0.809
AEGDTVIYSKYGGTE	CAB01005.1; 10 KDA CHAPERONIN GROES	0.255	0.806
YNEWSFAQAQHLTMA	PERIPLASMIC PHOSPHATE-BINDING LIPOPROTEIN PSTS3 YP_177768	0.122	0.803
AFNSGSFNNGALWTG	YP_177963; PPE FAMILY PROTEIN	0.148	0.797
EFNLKKMWLSPNGTI	CAA17111.1; PROBABLE ISOCITRATE DEHYDROGENASE	0.052	0.793
SMAGSSAMILAAYHP	CAB10044.1; SECRETED ANTIGEN 85-B FBPB (85B)	0.330	0.787
LFCEKIFGPTRDWEC	RNA POLYMERASE BETA'-SUBUNIT AAA21417	0.183	0.787
SAERCGSPAWDLPTV	PERIPLASMIC PHOSPHATE-BINDING LIPOPROTEIN PSTS2 YP_177769	0.132	0.781
IWKLIKDMLILPYLD	CAA17111.1; PROBABLE ISOCITRATE DEHYDROGENASE	0.190	0.778
LPTWTELAALPDFLG	CAB06237.1; CONSERVED HYPOTHETICAL PROTEIN	0.071	0.778
GFFNTGAGGGSGFGN	YP_177963; PPE FAMILY PROTEIN	-0.052	0.776
ARASFSYGLNAKWPV	CAA17111.1; PROBABLE ISOCITRATE DEHYDROGENASE	0.478	0.776
PANHRYLGPVLWSPD	POSSIBLE GLYCOSYL TRANSFERASE CAB05418	0.522	0.776
SDTVRDLIARWEQRD	MYCOBACTERIUM BOVIS MYCOCEROSIC ACID SYNTHASE GENE; M95808	0.000	0.774
RCLPLADHIARRFEG	ALTERNATE RNA POLYMERASE SIGMA FACTOR SIGF CAB07069	0.174	0.773
SFPGFPGFPEFPAIP	CAB06237.1; CONSERVED HYPOTHETICAL PROTEIN	0.062	0.771
LSSATDEPEPHWGPF	PPE FAMILY PROTEIN CAE55334	0.443	0.770
FKDEFERVYEEEFKA	CAA17111.1; PROBABLE ISOCITRATE DEHYDROGENASE	0.552	0.767
HLKLNGKVLAAMYQG	PERIPLASMIC PHOSPHATE-BINDING LIPOPROTEIN PSTS1 YP_177770	0.206	0.766
SSGLMAVHLACRSLH	MYCOBACTERIUM BOVIS MYCOCEROSIC ACID SYNTHASE GENE; M95808	0.001	0.763
VGWANMGLGNIGFGN	YP_177963; PPE FAMILY PROTEIN	0.861	0.763
AFAKHQELFDDLGVN	PROBABLE ISOCITRATE DEHYDROGENASE CAA16247	0.635	0.761
NFRWGGAFDLDRLLF	POSSIBLE GLYCOSYL TRANSFERASE CAB05419	0.363	0.760
MLATRHTQTWIQKYI	PUTATIVE CYCLOPROPANE-FATTY-ACYL-PHOSPHOLIPID SYNTHASE UFAA1 NP_854118	-0.143	0.760
DMAQTFTSIAISMYC	CONSERVED HYPOTHETICAL PROTEIN CAA15739	0.273	0.759
SCVYATASRWGGPPV	POSSIBLE GLYCOSYL TRANSFERASE CAB05415	0.179	0.758
QLKTDAKLLSSILTY	CAA98350.1; CELL SURFACE LIPOPROTEIN MPT83	-0.236	0.756
DAATAQTLQAFLHWA	PERIPLASMIC PHOSPHATE-BINDING LIPOPROTEIN PSTS1 YP_177770	0.607	0.754
VVELDGDEMTRVIWK	CAA17111.1; PROBABLE ISOCITRATE DEHYDROGENASE	0.208	0.753
DGMFKDEFERVYEEE	CAA17111.1; PROBABLE ISOCITRATE DEHYDROGENASE	0.362	0.752
SGNFSNGILWRGNYE	YP_177963; PPE FAMILY PROTEIN	1.150	0.752
QDDYNGWDINTPAFE	CAB10044.1; SECRETED ANTIGEN 85-B FBPB (85B)	0.142	0.750
GPGSASLVAAAQMWD	CAE55371.1; PPE FAMILY PROTEIN	0.179	0.744
GLGLPPPWQPALPRL	PPE FAMILY PROTEIN CAE55504	0.174	0.741
RKPITYDTLWQADTD	CAA98382.1; IMMUNOGENIC PROTEIN MPT64	0.344	0.740
EMIEAVGYRSWPRYF	PUTATIVE CYCLOPROPANE-FATTY-ACYL-PHOSPHOLIPID SYNTHASE UFAA1 NP_854118	0.363	0.739
FPHHPIHTVPSEEVL	POSSIBLE GLYCOSYL TRANSFERASE CAB05418	0.486	0.735
SITYNEWSFAQAQHL	PERIPLASMIC PHOSPHATE-BINDING LIPOPROTEIN PSTS3 YP_177768	0.443	0.734

GMRFDKGYISGYFVT	60 kDa chaperonin 2 P0A521	-0.049	0.733
AYVRSCPGYTLDYNA	PERIPLASMIC PHOSPHATE-BINDING LIPOPROTEIN PSTS2 YP_177769	-0.064	0.729
MGTQLLASEPVFAAT	MYCOBACTERIUM BOVIS MYCOCEROSIC ACID SYNTHASE GENE; M95808	-0.151	0.728
NDHTPKRVAVSSFGM	MYCOBACTERIUM BOVIS MYCOCEROSIC ACID SYNTHASE GENE; M95808	0.024	0.727
HAVRIFYKDAFAKHQ	PROBABLE ISOCITRATE DEHYDROGENASE CAA16247	0.261	0.725
ERYPEWNEGVKGAWV	CONSERVED HYPOTHETICAL PROTEIN TB18.5CAD93033	0.561	0.720
SIMAIVADIERYPEW	CONSERVED HYPOTHETICAL PROTEIN TB18.5CAD93033	0.513	0.717
GQILETHLGWCAHSG	RNA POLYMERASE BETA-SUBUNIT AAA21416	0.350	0.717
SDFVKKFEETFEVTA	CAB07109.1; PROBABLE 50S RIBOSOMAL PROTEIN L7/L12 RPLL	-0.106	0.715
YGHWWPAVAVGCAQP	MYCOBACTERIUM BOVIS MYCOCEROSIC ACID SYNTHASE GENE; M95808	0.028	0.707
GKPTSTNPIASIFAW	CAA17111.1; PROBABLE ISOCITRATE DEHYDROGENASE	-0.165	0.707
GLDQIENREVLRPLL	ALTERNATE RNA POLYMERASE SIGMA FACTOR SIGF CAB07069	0.417	0.705
QDPEGWGKSPGFGTT	PERIPLASMIC PHOSPHATE-BINDING LIPOPROTEIN PSTS1 YP_177770	-0.111	0.705
ASRANEYADVPEMFR	ALTERNATE RNA POLYMERASE SIGMA FACTOR SIGF CAB07069	0.668	0.704
SRACQGDLGMSHQDW	CAA16102.1; ESAT-6 LIKE PROTEIN ESXQ	0.582	0.699
VDDKIHARSTGPYSM	RNA POLYMERASE BETA-SUBUNIT AAA21416	0.136	0.697
FLFTQYLSKQDPEGW	PERIPLASMIC PHOSPHATE-BINDING LIPOPROTEIN PSTS1 YP_177770	0.126	0.694
GSLSALLDPSQGMGP	CAB10044.1; SECRETED ANTIGEN 85-B FBPB (85B)	0.048	0.691
VMQVISRSEYRQAAA	POSSIBLE GLYCOSYL TRANSFERASE CAB05415	0.267	0.691
LYPLFNLWGPAFHER	PERIPLASMIC PHOSPHATE-BINDING LIPOPROTEIN PSTS1 YP_177770	0.297	0.690
AAPGLAARRRRILFV	POSSIBLE GLYCOSYL TRANSFERASE CAB05415	0.373	0.689
YIVKPKMHGPAEVAF	MTB81 (publication)	0.236	0.687
SRRRSLRWSWLLSVL	CAB09453.1; PROBABLE SERINE PROTEASE PEPA	-0.110	0.687
VMFSLHVKATMMKVS	PROBABLE ISOCITRATE DEHYDROGENASE CAA16247	-0.230	0.687
GAYAAANSWVDVFAH	MYCOBACTERIUM BOVIS MYCOCEROSIC ACID SYNTHASE GENE; M95808	0.424	0.687
EQAGLRGLLRVISSE	MYCOBACTERIUM BOVIS MYCOCEROSIC ACID SYNTHASE GENE; M95808	-0.018	0.686
VRVNFEQLMSGYFAD	MYCOBACTERIUM BOVIS ACYL-COA SYNTHASE GENE; U75685	0.445	0.686
RFRGFDNWEGDAATA	MTB48 AAK31576	0.532	0.685
NDLFAAFLDETMTYS	PUTATIVE CYCLOPROPANE-FATTY-ACYL-PHOSPHOLIPID SYNTHASE UFAA1 NP_854118	-0.021	0.684
LSEVERQIHEIVRAY	PPE FAMILY PROTEIN CAE55489	0.296	0.684
SYPAMTANVGDMAGY	CAA16102.1; ESAT-6 LIKE PROTEIN ESXQ	-0.131	0.683
ARFITPVHPGYTATF	PPE FAMILY PROTEIN CAE55504	0.525	0.683
MSVAALAGMRREQPL	MYCOBACTERIUM BOVIS MYCOCEROSIC ACID SYNTHASE GENE; M95808	0.227	0.679
WDGLAEELHAAAGSF	PPE FAMILY PROTEIN CAE55504	0.679	0.678
MSDAVPQVGSQFGPY	TRANSMEMBRANE SERINE/THREONINE-PROTEIN KINASE D PKND NP_215446	0.332	0.676
YKDAFAKHQELFDDL	PROBABLE ISOCITRATE DEHYDROGENASE CAA16247	0.412	0.676
DRVGGFSEGGCWRTF	MYCOBACTERIUM BOVIS MYCOCEROSIC ACID SYNTHASE GENE; M95808	0.413	0.676
GNKASFLDQVHFQPL	PERIPLASMIC PHOSPHATE-BINDING LIPOPROTEIN PSTS1 YP_177770	0.231	0.674
RTLRKYIAADRKILN	POSSIBLE GLYCOSYL TRANSFERASE CAB05418	-0.085	0.673
QLHVWARREHPTYED	MTB48 AAK31576	0.064	0.672
RPWHIGLYGNAGGLG	PPE FAMILY PROTEIN CAE55334	0.232	0.669
GVPSRLGYLLDLAPK	RNA POLYMERASE BETA'-SUBUNIT AAA21417	-0.319	0.666
RMGHIELAAPVTHIW	RNA POLYMERASE BETA'-SUBUNIT AAA21417	0.099	0.665
GHGLNAQTGVYEDLL	60 kDa chaperonin 2 P0A521	0.177	0.664
LAATMEQTYGVRPGA	MYCOBACTERIUM BOVIS MYCOCEROSIC ACID SYNTHASE GENE; M95808	-0.043	0.664
RVNPFGFIETPYRKV	RNA POLYMERASE BETA-SUBUNIT AAA21416	0.334	0.662
IEAYPQWISEYKEVE	CONSERVED HYPOTHETICAL PROTEIN TB16.3CAD97060	0.120	0.662
GDFYAGEKSMTLDRA	PROBABLE ISOCITRATE DEHYDROGENASE CAA16247	0.319	0.661
AGNVYVTSEGMYGRV	TRANSMEMBRANE SERINE/THREONINE-PROTEIN KINASE D PKND NP_215446	-0.077	0.660
RGPGQMLGGLPVGQM	CAE55371.1; PPE FAMILY PROTEIN	-0.095	0.659
DLVRRSPWGEMFAST	MYCOBACTERIUM BOVIS MYCOCEROSIC ACID SYNTHASE GENE; M95808	0.111	0.659
SRLGYLLDLAPKDLE	RNA POLYMERASE BETA'-SUBUNIT AAA21417	-0.156	0.658
TDGSITYNEWSFAVG	PERIPLASMIC PHOSPHATE-BINDING LIPOPROTEIN PSTS2 YP_177769	-0.032	0.654
QPTPAWTELAAAQRR	PUTATIVE CYCLOPROPANE-FATTY-ACYL-PHOSPHOLIPID SYNTHASE UFAA1 NP_854118	0.017	0.650
VYLLDGLRAQDDYNG	CAB10044.1; SECRETED ANTIGEN 85-B FBPB (85B)	0.155	0.649
ALVSWLPFYHDMGLV	MYCOBACTERIUM BOVIS ACYL-COA SYNTHASE GENE; U75685	0.509	0.648
RPGLPVEYLQVPSPS	CAB10044.1; SECRETED ANTIGEN 85-B FBPB (85B)	-0.068	0.648
YEDIVGLERLYAENP	MTB48 AAK31576	0.179	0.648
RVGFPQHVENALRLM	POSSIBLE GLYCOSYL TRANSFERASE CAB05415	-0.026	0.645
EQVLAERLAGWQERY	HYPOTHETICAL PROTEIN RV2623 NP_217139	0.595	0.645
GDFQHTTEEAIRMNC	CONSERVED HYPOTHETICAL PROTEIN CAB08634	0.119	0.640
QLELTEGMRFDKGYI	60 kDa chaperonin 2 P0A521	0.312	0.640

KIRAWGRRLMIGTAA	CAB10044.1; SECRETED ANTIGEN 85-B FBPB (85B)	0.093	0.634
TIPEFPIHWTSTGGI	YP_177963; PPE FAMILY PROTEIN	0.782	0.634
ELSDVETGLFVPQAV	MYCOBACTERIUM BOVIS MYCOCEROSIC ACID SYNTHASE GENE; M95808	-0.213	0.631
QLLRLLGRGGMGEVY	TRANSMEMBRANE SERINE/THREONINE-PROTEIN KINASE D PKND NP_215446	-0.411	0.628
AITNTARPWHIGLYG	PPE FAMILY PROTEIN CAE55334	0.568	0.627
SLAHFADGWNTFNLT	MTB48 AAK31576	0.385	0.625
RVEEFNLKKMWLSPN	CAA17111.1; PROBABLE ISOCITRATE DEHYDROGENASE	0.196	0.622
VHIDQIDIPGMSLFD	YP_177963; PPE FAMILY PROTEIN	0.049	0.622
VFGHAVRIFYKDAFA	PROBABLE ISOCITRATE DEHYDROGENASE CAA16247	0.031	0.621
ILYAVRWPDPWPTTF	POSSIBLE HEMOLYSIN-LIKE PROTEIN CAA17201	0.361	0.620
TRARISGMPVLFWLD	PROBABLE ISOCITRATE DEHYDROGENASE CAA16247	-0.174	0.617
PTPGSPLFPDLPSFP	CAB06237.1; CONSERVED HYPOTHETICAL PROTEIN	-0.168	0.617
IGGILYAVRWPDPWP	POSSIBLE HEMOLYSIN-LIKE PROTEIN CAA17201	0.068	0.615
ALEDLVRAYQSMSGT	CAA16104.1; SECRETED ESAT-6 LIKE PROTEIN ESXR	-0.170	0.615
SLDQQRQWILHMAKL	MTB48 AAK31576	0.256	0.614
APIYEILHDAKERAH	CAB08889.1; IRON-REGULATED CONSERVED HYPOTHETICAL PROTEIN	0.571	0.613
FSNGILWRGNYEGLF	YP_177963; PPE FAMILY PROTEIN	0.780	0.613
LNWVRRRHGLSSLGW	POSSIBLE GLYCOSYL TRANSFERASE CAB05415	0.295	0.613
AVVEDATLTNITDEL	MYCOBACTERIUM BOVIS MYCOCEROSIC ACID SYNTHASE GENE; M95808	0.182	0.613
VDVSEWPGIDWPATQ	HYPOTHETICAL PROTEIN RV2623 NP_217139	0.124	0.613
RVVKLAAGSKTQTVL	TRANSMEMBRANE SERINE/THREONINE-PROTEIN KINASE D PKND NP_215446	-0.219	0.612
LAAAAAWDGLAEELG	YP_177963; PPE FAMILY PROTEIN	0.357	0.607
NITDELIDRDWSPKV	MYCOBACTERIUM BOVIS MYCOCEROSIC ACID SYNTHASE GENE; M95808	0.377	0.606
FACDPRYNQLLGPLP	POSSIBLE GLYCOSYL TRANSFERASE CAB05415	-0.015	0.602
RAYHAMSSTHEANTM	CAA17363.1; LOW MOLECULAR WEIGHT PROTEIN ANTIGEN 7 ESXH	-0.078	0.601
ETMTYSCAMFTDLLA	PUTATIVE CYCLOPROPANE-FATTY-ACYL-PHOSPHOLIPID SYNTHASE UFAA1 NP_854118	0.159	0.598
DVLAAGDFWGGAGSV	PUTATIVE ESAT-6 LIKE PROTEIN ESXN YP_177838	0.461	0.596
QSLTKLAAAWGGSGS	CAE55648	0.232	0.595
FDWRYPPSPPPQPTQ	CAA17582.1; PROBABLE SERINE PROTEASE PEPD	0.074	0.594
LAERLAGWQERYPNV	HYPOTHETICAL PROTEIN RV2623 NP_217139	0.249	0.591
DPTQQIPKLVANNTR	CAB10044.1; SECRETED ANTIGEN 85-B FBPB (85B)	-0.037	0.591
SRYEVHFACDPRFNK	POSSIBLE GLYCOSYL TRANSFERASE CAB05418	0.410	0.590
GERKMSALVDASDAL	CAA16030.1; PROBABLE MOLYBDOPTERIN-GUANINE DINUCLEOTIDE BIOSYNTHESIS PROTEIN	0.289	0.589
FLAEGGEIMITPEEG	MYCOBACTERIUM BOVIS MYCOCEROSIC ACID SYNTHASE GENE; M95808	0.008	0.589
YRPVAGDFTPAAEIV	CAA17343.1; HEAT SHOCK PROTEIN HSP	-0.033	0.589
MVPLAQIYNNRAERQ	PPE FAMILY PROTEIN CAE55489	0.497	0.587
IMGASITSDGFHMVA	3-OXOACYL-[ACYL-CARRIER PROTEIN] SYNTHASE 2 KASB CAA94642	0.203	0.586
NFDDSIAFLAAQELI	MTB81 (publication)	0.588	0.581
NATATPSSGFFNSGA	YP_177963; PPE FAMILY PROTEIN	-0.031	0.581
TRYSSAYELEGAVKR	SECRETED L-ALANINE DEHYDROGENASE ALD CAA15575	0.048	0.580
LSGSEEDETAWRNGD	MYCOBACTERIUM BOVIS MYCOCEROSIC ACID SYNTHASE GENE; M95808	0.481	0.579
SLDMSVAALAGMRRE	MYCOBACTERIUM BOVIS MYCOCEROSIC ACID SYNTHASE GENE; M95808	-0.024	0.578
NFVRSSNLKFQDAYN	CAB10044.1; SECRETED ANTIGEN 85-B FBPB (85B)	0.290	0.578
TPKRVAVSSFGMSGT	MYCOBACTERIUM BOVIS MYCOCEROSIC ACID SYNTHASE GENE; M95808	-0.046	0.577
WSVKVPRRLKELHLR	ALTERNATE RNA POLYMERASE SIGMA FACTOR SIGF CAB07069	0.107	0.576
AAQVRVAAAAYETAY	CAE55371.1; PPE FAMILY PROTEIN	-0.083	0.575
VDELVPRSLHWLRPI	PUTATIVE CYCLOPROPANE-FATTY-ACYL-PHOSPHOLIPID SYNTHASE UFAA1 NP_854118	0.352	0.573
PDIPGLPDFKWPPTP	CAB06237.1; CONSERVED HYPOTHETICAL PROTEIN	-0.127	0.571
NLCRIFTDGDHTLYA	POSSIBLE GLYCOSYL TRANSFERASE CAB05415	0.399	0.568
LRCLILTSYTSDEAM	TWO COMPONENT TRANSCRIPTIONAL REGULATORY PROTEIN DEVR NP_217649	0.050	0.568
DMLILPYLDIRLDYY	CAA17111.1; PROBABLE ISOCITRATE DEHYDROGENASE	0.030	0.567
LMAGGGMYETGAGGS	PROBABLE ISOCITRATE DEHYDROGENASE CAA16247	0.203	0.565
FPMMTEKGTFIINGT	RNA POLYMERASE BETA-SUBUNIT AAA21416	-0.078	0.564
AKFILDVDPRYIHLI	MCE-FAMILY PROTEIN MCE1AYP_177701	0.156	0.563
DAARVICRRSKLMTR	MYCOBACTERIUM BOVIS MYCOCEROSIC ACID SYNTHASE GENE; M95808	0.007	0.560
VIDSMFMSKKALCDF	PROBABLE ISOCITRATE DEHYDROGENASE CAA16247	0.702	0.558
RRLAEAFGPDFAGFP	POSSIBLE GLYCOSYL TRANSFERASE CAB05418	0.353	0.558
LMAVHLACRSLHDGE	MYCOBACTERIUM BOVIS MYCOCEROSIC ACID SYNTHASE GENE; M95808	0.271	0.554
GYTATFLETPSQFFP	PPE FAMILY PROTEIN CAE55504	0.518	0.554
EFERVYEEEFKAQFE	CAA17111.1; PROBABLE ISOCITRATE DEHYDROGENASE	0.346	0.552
VGFGNMGLGNIGFGN	YP_177963; PPE FAMILY PROTEIN	0.927	0.550

ALVHGMFVALPAATA	CAE55335.1; PE FAMILY PROTEIN	0.150	0.548
DELIDRDWSPKVFGS	MYCOBACTERIUM BOVIS MYCOCEROSIC ACID SYNTHASE GENE; M95808	0.356	0.548
PPALSQDRFADFPAL	CAB09453.1; PROBABLE SERINE PROTEASE PEPA	0.210	0.544
GILWRGNYEGLFSYS	YP_177963; PPE FAMILY PROTEIN	0.025	0.542
MSAEQPTIIYTLTDE	PROBABLE ISOCITRATE DEHYDROGENASE CAA16247	-0.061	0.541
VLDVRLPDGNGIELC	TWO COMPONENT TRANSCRIPTIONAL REGULATORY PROTEIN DEVR NP_217649	0.394	0.538
MYSYEGASAAASALP	PPE FAMILY PROTEIN CAE55334	0.086	0.536
AALEPVNPPKPPPAI	MTB48 AAK31576	0.148	0.532
MWDSVASDLFSAASA	CAE55371.1; PPE FAMILY PROTEIN	0.137	0.532
HAFGDQYRATNFKVD	CAA17111.1; PROBABLE ISOCITRATE DEHYDROGENASE	0.298	0.532
DAADKVLGYRNWLGL	MTB81 (publication)	0.057	0.531
HTIPSERFFGNLTQG	POSSIBLE GLYCOSYL TRANSFERASE CAB05415	0.418	0.529
GTSERDERDRLVSER	MYCOBACTERIUM BOVIS MYCOCEROSIC ACID SYNTHASE GENE; M95808	-0.008	0.529
GALWTGDHHGLVGFS	YP_177963; PPE FAMILY PROTEIN	0.560	0.528
YNEWSFAVGKQLNMA	PERIPLASMIC PHOSPHATE-BINDING LIPOPROTEIN PSTS2 YP_177769	0.588	0.525
WVAVWYTATILHNAG	POSSIBLE HEMOLYSIN-LIKE PROTEIN CAA17201	-0.072	0.525
SKTAASPSPSRPQSS	RNA POLYMERASE BETA-SUBUNIT AAA21416	-0.083	0.525
FYRRGLFGTIGPYNL	POSSIBLE GLYCOSYL TRANSFERASE CAB05419	0.544	0.525
SQMHVSRLLAKSLAR	ALTERNATE RNA POLYMERASE SIGMA FACTOR SIGF CAB07069	0.012	0.524
TDGIPPPNSALVSWL	MYCOBACTERIUM BOVIS ACYL-COA SYNTHASE GENE; U75685	0.082	0.524
IVRDVLAAGDFWGGA	PUTATIVE ESAT-6 LIKE PROTEIN ESXN YP_177838	0.302	0.524
LAAASAWDGLAEELH	PPE FAMILY PROTEIN CAE55504	0.928	0.523
RCGSPAWNLPVVFGP	PERIPLASMIC PHOSPHATE-BINDING LIPOPROTEIN PSTS3 YP_177768	0.024	0.523
LDGLRAQDDYNGWDI	CAB10044.1; SECRETED ANTIGEN 85-B FBPB (85B)	0.389	0.523
MAKQAQYVAQLHVWA	MTB48 AAK31576	0.099	0.522
TAGNPAKRAMLRDMG	MYCOBACTERIUM BOVIS MYCOCEROSIC ACID SYNTHASE GENE; M95808	0.422	0.522
IGPYNLRYRVLADWD	POSSIBLE GLYCOSYL TRANSFERASE CAB05419	0.349	0.521
AMYWAQELAAQTDDQ	PROBABLE ISOCITRATE DEHYDROGENASE CAA16247	0.223	0.521
SLFPEFSELFAAFPS	CAA17245.1; HEAT SHOCK PROTEIN HSPX	-0.126	0.520
PWILAYEDHNVDAGL	MTB81 (publication)	0.342	0.518
GEINGQFFVEMRMID	TRANSMEMBRANE SERINE/THREONINE-PROTEIN KINASE D PKND NP_215446	-0.083	0.518
CIRAAARGAHIRSVT	PUTATIVE CYCLOPROPANE-FATTY-ACYL-PHOSPHOLIPID SYNTHASE UFAA1 NP_854118	-0.145	0.516
DESYKVTGTAPIYEI	CAB08889.1; IRON-REGULATED CONSERVED HYPOTHETICAL PROTEIN	-0.048	0.514
AALNATDPGAAAQFN	LOW MOLECULAR WEIGHT T-CELL ANTIGEN TB8.4 NP_215690	-0.051	0.513
YDPEPGVPGRSVSRW	MYCOBACTERIUM BOVIS MYCOCEROSIC ACID SYNTHASE GENE; M95808	0.115	0.511
GINYRVLWQAAGPDT	CAB06500.1; IMMUNOGENIC PROTEIN MPT63	0.217	0.510
LLLLGEGIPDPGDIF	CAB06237.1; CONSERVED HYPOTHETICAL PROTEIN	-0.036	0.510
IGPFRIEPIDVPAVV	YP_177963; PPE FAMILY PROTEIN	-0.553	0.509
VKPPTWWHSLPTDRP	POSSIBLE GLYCOSYL TRANSFERASE CAB05418	0.299	0.507
AGEVEYVPSSEVDYM	RNA POLYMERASE BETA-SUBUNIT AAA21416	-0.105	0.507
PGLPDIPGLPDFKWP	CAB06237.1; CONSERVED HYPOTHETICAL PROTEIN	0.006	0.507
DHTLYADVPELMPTY	POSSIBLE GLYCOSYL TRANSFERASE CAB05415	0.313	0.507
YFAAYVITSVDEEMR	RNA POLYMERASE BETA'-SUBUNIT AAA21417	0.194	0.505
FNRLPDELSDVETGL	MYCOBACTERIUM BOVIS MYCOCEROSIC ACID SYNTHASE GENE; M95808	-0.176	0.505
PLDTPPAPAPPPFRL	YP_177963; PPE FAMILY PROTEIN	1.112	0.505
DALFTGLIAIHGLKA	MTB81 (publication)	0.199	0.502
DVPELMPTYDLPANH	POSSIBLE GLYCOSYL TRANSFERASE CAB05415	0.533	0.501
CPDGTVGEIWVHGDN	MYCOBACTERIUM BOVIS ACYL-COA SYNTHASE GENE; U75685	0.519	0.501
HPRSLFPEFSELFAA	CAA17245.1; HEAT SHOCK PROTEIN HSPX	0.178	0.501
AMMARDTAEAAKWGG	CAA17363.1; LOW MOLECULAR WEIGHT PROTEIN ANTIGEN 7 ESXH	0.084	0.501
VQFAAAVQAAMEDGY	MYCOBACTERIUM BOVIS MYCOCEROSIC ACID SYNTHASE GENE; M95808	-0.049	0.500
AGAVMQVISRSEYRQ	POSSIBLE GLYCOSYL TRANSFERASE CAB05415	0.314	0.500
TAHATAWYGLNDLAQ	MYCOBACTERIUM BOVIS MYCOCEROSIC ACID SYNTHASE GENE; M95808	0.289	0.500
VKGAWVLARYDDGRP	CONSERVED HYPOTHETICAL PROTEIN TB18.5CAD93033	0.292	0.499
LFIHRPDALARRIGR	PUTATIVE CYCLOPROPANE-FATTY-ACYL-PHOSPHOLIPID SYNTHASE UFAA1 NP_854118	0.109	0.499
KPAALVHGMFVALPA	CAE55335.1; PE FAMILY PROTEIN	0.037	0.498
TGHQVGDRVGGFSEG	MYCOBACTERIUM BOVIS MYCOCEROSIC ACID SYNTHASE GENE; M95808	0.467	0.498
PWKAPPPIAHSTVLV	PPE FAMILY PROTEIN CAE55489	-0.104	0.498
EMTAVQRSFNRTLLS	PPE FAMILY PROTEIN CAE55489	-0.099	0.496
DLCDYRDVDGQYDSV	PUTATIVE CYCLOPROPANE-FATTY-ACYL-PHOSPHOLIPID SYNTHASE UFAA1 NP_854118	-0.125	0.495
QEMLNVMEEHQVRRV	HYPOTHETICAL PROTEIN RV2626C NP_217142	0.066	0.492
FFNTGTGNWGLFNSG	YP_177963; PPE FAMILY PROTEIN	0.391	0.491

AVIPGYPVAGQVWEA	CAB06500.1; IMMUNOGENIC PROTEIN MPT63	-0.135	0.491
NYRYAINNSFGFGGH	3-OXOACYL-[ACYL-CARRIER PROTEIN] SYNTHASE 2 KASB CAA94642	0.464	0.488
LGSPGQGAYAAANSW	MYCOBACTERIUM BOVIS MYCOCEROSIC ACID SYNTHASE GENE; M95808	0.231	0.487
DMRARGMKAVSPLTV	3-OXOACYL-[ACYL-CARRIER PROTEIN] SYNTHASE 2 KASB CAA94642	-0.149	0.486
FSNGILWRGNFEGLF	YP_177963; PPE FAMILY PROTEIN	0.483	0.486
AIPVDQAKRIADELI	CAA17582.1; PROBABLE SERINE PROTEASE PEPD	0.325	0.486
TGNFSNGILWRGNYE	YP_177963; PPE FAMILY PROTEIN	1.015	0.485
SVRSLPAALRACACL	MYCOBACTERIUM BOVIS ACYL-COA SYNTHASE GENE; U75685	-0.184	0.485
TYSCAMFTDLLAQPT	PUTATIVE CYCLOPROPANE-FATTY-ACYL-PHOSPHOLIPID SYNTHASE UFAA1 NP_854118	0.224	0.483
RLAGIPYIAIANAYW	POSSIBLE GLYCOSYL TRANSFERASE CAB05415	0.389	0.482
VVPLMAGGGMYETGA	PROBABLE ISOCITRATE DEHYDROGENASE CAA16247	-0.016	0.481
SGQGSQWAAMGTQLL	MYCOBACTERIUM BOVIS MYCOCEROSIC ACID SYNTHASE GENE; M95808	0.182	0.479
DGGVVLGMYNFKESI	CAA17111.1; PROBABLE ISOCITRATE DEHYDROGENASE	0.015	0.478
ARRIGRHGLIGFGES	PUTATIVE CYCLOPROPANE-FATTY-ACYL-PHOSPHOLIPID SYNTHASE UFAA1 NP_854118	0.305	0.477
SELFAAFPSFAGLRP	CAA17245.1; HEAT SHOCK PROTEIN HSPX	-0.242	0.476
HTANATVYMIDTVLM	CAA98350.1; CELL SURFACE LIPOPROTEIN MPT83	-0.208	0.476
TAVAAICHYIAMWFV	POSSIBLE HEMOLYSIN-LIKE PROTEIN CAA17201	0.034	0.476
TDLGVPFTEPASVLA	SECRETED L-ALANINE DEHYDROGENASE ALD CAA15575	-0.068	0.472
NTMAMLARDGAEAAK	CAA16104.1; SECRETED ESAT-6 LIKE PROTEIN ESXR	0.059	0.472
GPMLAAASAWDGLAE	PPE FAMILY PROTEIN CAE55504	-0.095	0.472
RSEYRQAAARLADAF	POSSIBLE GLYCOSYL TRANSFERASE CAB05415	0.413	0.471
TNKQIADRMFLAEKT	TWO COMPONENT TRANSCRIPTIONAL REGULATORY PROTEIN DEVR NP_217649	0.004	0.471
DLNSRMPQSRHDIQQ	MCE-FAMILY PROTEIN MCE1AYP_177701	0.057	0.470
MWELYLAYSEAGFRS	PUTATIVE CYCLOPROPANE-FATTY-ACYL-PHOSPHOLIPID SYNTHASE UFAA1 NP_854118	0.019	0.469
AKVRRERMGHIELAA	RNA POLYMERASE BETA'-SUBUNIT AAA21417	-0.204	0.468
YRVLWQAAGPDTISG	CAB06500.1; IMMUNOGENIC PROTEIN MPT63	0.064	0.468
FVYAYVRSCPGYTLD	PERIPLASMIC PHOSPHATE-BINDING LIPOPROTEIN PSTS2 YP_177769	-0.060	0.468
LGSAEELVFSYDDMR	3-OXOACYL-[ACYL-CARRIER PROTEIN] SYNTHASE 2 KASB CAA94642	-0.013	0.467
WLDPYRPHENELIKK	PROBABLE ISOCITRATE DEHYDROGENASE CAA16247	0.833	0.466
KVCKGLEKIPLLGHL	CAB06237.1; CONSERVED HYPOTHETICAL PROTEIN	0.071	0.466
TYALTNATMPYVLEL	SECRETED L-ALANINE DEHYDROGENASE ALD CAA15575	-0.107	0.465
GVIFFFNPGELLPEA	CAA98382.1; IMMUNOGENIC PROTEIN MPT64	-0.209	0.463
MLSVVPLMAGGGMYE	PROBABLE ISOCITRATE DEHYDROGENASE CAA16247	0.042	0.463
KSQTVFMGDFPMMTE	RNA POLYMERASE BETA-SUBUNIT AAA21416	-0.125	0.462
PVDIGGYYAPDSDMT	PROBABLE ISOCITRATE DEHYDROGENASE CAA16247	0.030	0.461
SNMDQHLNMEALERA	POSSIBLE GLYCOSYL TRANSFERASE CAB05418	0.127	0.461
DIILNTHGVPRRMNI	RNA POLYMERASE BETA-SUBUNIT AAA21416	0.307	0.460
WGAQLNAMKGDLQSS	CAB10044.1; SECRETED ANTIGEN 85-B FBPB (85B)	0.234	0.457
SEEDETAWRNGDWYV	MYCOBACTERIUM BOVIS MYCOCEROSIC ACID SYNTHASE GENE; M95808	0.035	0.455
PYLTVELIEDLARRA	CAA16030.1; PROBABLE MOLYBDOPTERIN-GUANINE DINUCLEOTIDE BIOSYNTHESIS PROTEIN	0.127	0.455
GVPLYLLLGWVAVWY	POSSIBLE HEMOLYSIN-LIKE PROTEIN CAA17201	0.039	0.454
RSPISNMVSMANNHM	CAE55371.1; PPE FAMILY PROTEIN	-0.000	0.453
KAVKEYARKHPHSMG	PROBABLE ISOCITRATE DEHYDROGENASE CAA16247	0.341	0.452
MGDFPMMTEKGTFII	RNA POLYMERASE BETA-SUBUNIT AAA21416	0.063	0.451
ALVNLADEEKADLLV	CAB08889.1; IRON-REGULATED CONSERVED HYPOTHETICAL PROTEIN	0.005	0.450
GYRSWPRYFAALEQL	PUTATIVE CYCLOPROPANE-FATTY-ACYL-PHOSPHOLIPID SYNTHASE UFAA1 NP_854118	0.218	0.449
EENHLRWDSLGEFLA	PROBABLE ISOCITRATE DEHYDROGENASE CAA16247	0.262	0.449
VQRHPRSLFPEFSEL	CAA17245.1; HEAT SHOCK PROTEIN HSPX	0.337	0.449
FQSVVWGLTVGSWIG	CAE55371.1; PPE FAMILY PROTEIN	0.032	0.448
NFGFGNSGLGLPPGM	YP_177963; PPE FAMILY PROTEIN	0.083	0.448
LDPSRYEVHFACDPR	POSSIBLE GLYCOSYL TRANSFERASE CAB05415	0.263	0.447
RKYIAADRKILNEIA	POSSIBLE GLYCOSYL TRANSFERASE CAB05418	0.407	0.447
GESYMAGEWSSKELT	PUTATIVE CYCLOPROPANE-FATTY-ACYL-PHOSPHOLIPID SYNTHASE UFAA1 NP_854118	0.674	0.447
LQAHPHAVNGTAMRE	MYCOBACTERIUM BOVIS MYCOCEROSIC ACID SYNTHASE GENE; M95808	-0.176	0.444
QMQDAFETGVMFSLH	PROBABLE ISOCITRATE DEHYDROGENASE CAA16247	0.524	0.444
PSMTIGGQTYTIPLD	YP_177963; PPE FAMILY PROTEIN	-0.045	0.443
IVLATYEIVCSKYPD	PERIPLASMIC PHOSPHATE-BINDING LIPOPROTEIN PSTS2 YP_177769	0.018	0.441
NYQFGDVDAHGAMIR	PUTATIVE ESAT-6 LIKE PROTEIN ESXN YP_177838	0.153	0.441
GEHETLTAAAQYMRE	HYPOTHETICAL PROTEIN RV2626C NP_217142	-0.113	0.440
YELSPIEDFSGSMSL	RNA POLYMERASE BETA-SUBUNIT AAA21416	0.065	0.439

YPDATTGTAVRAFMQ	PERIPLASMIC PHOSPHATE-BINDING LIPOPROTEIN PSTS2 YP_177769	-0.115	0.439
SAFAAKAGLMRHTIG	CONSERVED HYPOTHETICAL PROTEIN TB9.8 CAD93159	-0.197	0.438
NEGVKGAWVLARYDD	CONSERVED HYPOTHETICAL PROTEIN TB18.5CAD93033	0.379	0.438
YNYMAPERFTGDEVT	TRANSMEMBRANE SERINE/THREONINE-PROTEIN KINASE D PKND NP_215446	0.102	0.436
LTNATMPYVLELADH	SECRETED L-ALANINE DEHYDROGENASE ALD CAA15575	0.138	0.435
PEWNEGVKGAWVLAR	CONSERVED HYPOTHETICAL PROTEIN TB18.5CAD93033	0.677	0.434
RRKAGEVEYVPSSEV	RNA POLYMERASE BETA-SUBUNIT AAA21416	-0.127	0.433
LWVYCGNGTPNELGG	CAB10044.1; SECRETED ANTIGEN 85-B FBPB (85B)	0.132	0.428
KMGLAIPGTNWIGQA	CAB06237.1; CONSERVED HYPOTHETICAL PROTEIN	0.043	0.428
GVYFDETIDKSTDKT	RNA POLYMERASE BETA-SUBUNIT AAA21416	-0.071	0.427
DAAVGHGDRGPVWVF	MYCOBACTERIUM BOVIS MYCOCEROSIC ACID SYNTHASE GENE; M95808	0.211	0.427
AAQMWDSVASDLFSA	CAE55371.1; PPE FAMILY PROTEIN	-0.068	0.424
TWWDSLPTDRPIVYA	POSSIBLE GLYCOSYL TRANSFERASE CAB05415	-0.020	0.422
HDTLDAVERREATWR	CONSERVED HYPOTHETICAL PROTEIN CAB08634	0.288	0.421
VDTLNSGQYTVFAPT	CAA98373.1; MAJOR SECRETED IMMUNOGENIC PROTEIN	0.045	0.419
QIVERFGFSEIMRST	RNA POLYMERASE BETA-SUBUNIT AAA21416	-0.186	0.419
HRYLGPVLWSPDVKP	POSSIBLE GLYCOSYL TRANSFERASE CAB05418	0.370	0.417
LDVAEGDTVIYSKYG	CAB01005.1; 10 KDA CHAPERONIN GROES	0.019	0.414
YDHPTFAVHDTLFYC	SECRETED L-ALANINE DEHYDROGENASE ALD CAA15575	0.222	0.414
EYLGPVLWSPAGKPP	POSSIBLE GLYCOSYL TRANSFERASE CAB05415	-0.007	0.412
NRAAAALMAKLRGAA	TWO COMPONENT TRANSCRIPTIONAL REGULATORY PROTEIN DEVR NP_217649	0.012	0.411
DEALLDIYRKLRPGE	RNA POLYMERASE BETA-SUBUNIT AAA21416	-0.017	0.411
PVETDDIDHFGNRRL	RNA POLYMERASE BETA-SUBUNIT AAA21416	0.505	0.411
CAGGGATSLISYMLP	MYCOBACTERIUM BOVIS ACYL-COA SYNTHASE GENE; U75685	-0.045	0.409
EDGEVMRDYRLRVSD	PPE FAMILY PROTEIN CAE55489	0.419	0.409
RVGRYKVNKKLGLHV	RNA POLYMERASE BETA-SUBUNIT AAA21416	0.025	0.407
SASLVAAAQMWDSVA	CAE55371.1; PPE FAMILY PROTEIN	0.029	0.407
TLPVQRHPRSLFPEF	CAA17245.1; HEAT SHOCK PROTEIN HSPX	0.087	0.407
IYNTSTLPLGTPALV	YP_177963; PPE FAMILY PROTEIN	-0.203	0.405
RLVKLLYRLERPLLF	POSSIBLE GLYCOSYL TRANSFERASE CAB05415	0.039	0.404
KGSQQEDEALYTEDR	MTB48 AAK31576	-0.143	0.401
MIDGVYKVCKGLEKI	CAB06237.1; CONSERVED HYPOTHETICAL PROTEIN	-0.218	0.400
VPTYNLPANHRYLGP	POSSIBLE GLYCOSYL TRANSFERASE CAB05418	0.292	0.399
HYLAAVYRTDLADRV	CAA16030.1; PROBABLE MOLYBDOPTERIN-GUANINE DINUCLEOTIDE BIOSYNTHESIS PROTEIN	0.005	0.398
RLRVSDALSKLTPWK	PPE FAMILY PROTEIN CAE55489	0.014	0.398
YTFKEDEYPSTAYLQ	MYCOBACTERIUM BOVIS ACYL-COA SYNTHASE GENE; U75685	0.213	0.398
ATYEIVCSKYPDATT	PERIPLASMIC PHOSPHATE-BINDING LIPOPROTEIN PSTS2 YP_177769	0.032	0.397
ACLQPHDPAFTFMDY	MYCOBACTERIUM BOVIS ACYL-COA SYNTHASE GENE; U75685	0.164	0.397
HAIHTIPSERFFGNL	POSSIBLE GLYCOSYL TRANSFERASE CAB05415	0.583	0.396
WDLTSAEFTRRRYAE	CONSERVED HYPOTHETICAL PROTEIN CAB08634	0.281	0.395
LGWNLCRIFTDGDHT	POSSIBLE GLYCOSYL TRANSFERASE CAB05415	0.329	0.395
NYGQVVAALNATDPG	LOW MOLECULAR WEIGHT T-CELL ANTIGEN TB8.4 NP_215690	-0.026	0.394
EVMRDYRLRVSDALS	PPE FAMILY PROTEIN CAE55489	0.450	0.391
VCAVDMPYLTVELIE	CAA16030.1; PROBABLE MOLYBDOPTERIN-GUANINE DINUCLEOTIDE BIOSYNTHESIS PROTEIN	0.556	0.391
GTATNQDGRTETLTM	MYCOBACTERIUM BOVIS MYCOCEROSIC ACID SYNTHASE GENE; M95808	-0.013	0.390
EPEPHWGPFGGAAPV	PPE FAMILY PROTEIN CAE55334	-0.070	0.390
NPAKRAMLRDMGVEH	MYCOBACTERIUM BOVIS MYCOCEROSIC ACID SYNTHASE GENE; M95808	-0.111	0.388
GLGNAGSNNVGFGNM	YP_177963; PPE FAMILY PROTEIN	0.084	0.388
PYAVEYVWGRQVGDN	MCE-FAMILY PROTEIN MCE1AYP_177701	0.132	0.386
RDFARASFSYGLNAK	CAA17111.1; PROBABLE ISOCITRATE DEHYDROGENASE	0.144	0.386
EVDLLDLDAPNGYTF	MYCOBACTERIUM BOVIS ACYL-COA SYNTHASE GENE; U75685	-0.047	0.385
ERFTGDEVTYRADIY	TRANSMEMBRANE SERINE/THREONINE-PROTEIN KINASE D PKND NP_215446	0.083	0.384
PTIVAMNNGMEDLLI	CAB06500.1; IMMUNOGENIC PROTEIN MPT63	0.151	0.383
FCKTNGQFDPTTMGT	PROBABLE ISOCITRATE DEHYDROGENASE CAA16247	0.878	0.382
IPPPNSALVSWLPFY	MYCOBACTERIUM BOVIS ACYL-COA SYNTHASE GENE; U75685	0.106	0.382
GYLDVYQWTLIREGP	PUTATIVE CYCLOPROPANE-FATTY-ACYL-PHOSPHOLIPID SYNTHASE UFAA1 NP_854118	0.145	0.381
AGLGPMLAAASAWDG	PPE FAMILY PROTEIN CAE55504	0.120	0.379
FLHDAFPNEFPDPTI	YP_177963; PPE FAMILY PROTEIN	0.006	0.379
SFYRPKRPGSYPIVL	PERIPLASMIC PHOSPHATE-BINDING LIPOPROTEIN PSTS3 YP_177768	0.089	0.379
GDTFLFTQYLSKQDP	PERIPLASMIC PHOSPHATE-BINDING LIPOPROTEIN PSTS1 YP_177770	-0.344	0.378
GSHYKITGTATGVDM	LIPOPROTEIN LPQH PRECURSOR P0A5J0	-0.087	0.375

VPLHRSDGSGDTFLF	PERIPLASMIC PHOSPHATE-BINDING LIPOPROTEIN PSTS1 YP_177770	-0.075	0.375
APAPPPFRLPLLFVN	YP_177963; PPE FAMILY PROTEIN	0.284	0.374
YLDIRLDYYDLGIEH	CAA17111.1; PROBABLE ISOCITRATE DEHYDROGENASE	0.061	0.372
VPSPFGLFPDVDWAE	PPE FAMILY PROTEIN CAE55504	-0.174	0.371
MTSAGGLLEQAAAVE	CAE55371.1; PPE FAMILY PROTEIN	0.559	0.371
GFPALPGLPSIPNLF	CAB06237.1; CONSERVED HYPOTHETICAL PROTEIN	-0.111	0.369
TSTNPIASIFAWTRG	CAA17111.1; PROBABLE ISOCITRATE DEHYDROGENASE	-0.224	0.367
PAWTELAAAQRRKID	PUTATIVE CYCLOPROPANE-FATTY-ACYL-PHOSPHOLIPID SYNTHASE UFAA1 NP_854118	0.031	0.367
LAAARAWRSLDVEMT	PPE FAMILY PROTEIN CAE55489	0.165	0.367
SRYEVHFACDPRYNQ	POSSIBLE GLYCOSYL TRANSFERASE CAB05415	0.310	0.366
EEGAYAFETLVRHDR	MYCOBACTERIUM BOVIS MYCOCEROSIC ACID SYNTHASE GENE; M95808	0.777	0.365
HETMVIPPIVFLPSM	YP_177963; PPE FAMILY PROTEIN	-0.108	0.364
ISMIDGPAPDGYPII	PERIPLASMIC PHOSPHATE-BINDING LIPOPROTEIN PSTS1 YP_177770	-0.072	0.364
NMSGWWNQAHTALAG	YP_177963; PPE FAMILY PROTEIN	0.004	0.363
GRNHYLAAVYRTDLA	CAA16030.1; PROBABLE MOLYBDOPTERIN-GUANINE DINUCLEOTIDE BIOSYNTHESIS PROTEIN	0.120	0.363
ACRSLHDGERDLALA	MYCOBACTERIUM BOVIS MYCOCEROSIC ACID SYNTHASE GENE; M95808	0.030	0.361
AWVLARYDDGRPSQV	CONSERVED HYPOTHETICAL PROTEIN TB18.5CAD93033	-0.041	0.361
DSGFVTDGKMFIIGR	MYCOBACTERIUM BOVIS ACYL-COA SYNTHASE GENE; U75685	-0.188	0.360
YAEYQQRSEKVLTEY	MTB48 AAK31576	0.056	0.360
GNFGAGSSGWWNQAH	YP_177963; PPE FAMILY PROTEIN	0.460	0.359
GTMKSQPWILAYEDH	MTB81 (publication)	0.288	0.359
EDATLTNITDELIDR	MYCOBACTERIUM BOVIS MYCOCEROSIC ACID SYNTHASE GENE; M95808	0.092	0.357
FETLVRHDRAYSGYI	MYCOBACTERIUM BOVIS MYCOCEROSIC ACID SYNTHASE GENE; M95808	-0.224	0.356
GPMVRKGTMKSQPWI	MTB81 (publication)	-0.019	0.355
WPVYLSTKNTILKAY	CAA17111.1; PROBABLE ISOCITRATE DEHYDROGENASE	0.058	0.353
FRHHAIHTIPSERFF	POSSIBLE GLYCOSYL TRANSFERASE CAB05415	0.737	0.352
PAGACRPFDRDRDGF	3-OXOACYL-[ACYL-CARRIER PROTEIN] SYNTHASE 2 KASB CAA94642	0.308	0.352
VGDPDEHSLLAGREQ	MYCOBACTERIUM BOVIS MYCOCEROSIC ACID SYNTHASE GENE; M95808	0.005	0.351
RKHGLSSLGWDLCRI	POSSIBLE GLYCOSYL TRANSFERASE CAB05418	0.180	0.350
QNFNTTFPVGPFFGL	YP_177963; PPE FAMILY PROTEIN	0.243	0.348
RFARFNLQERVIRPS	MYCOBACTERIUM BOVIS ACYL-COA SYNTHASE GENE; U75685	-0.154	0.347
NIGFFNSGTGNWGLF	YP_177963; PPE FAMILY PROTEIN	0.389	0.347
VAAASPYVAWMSVTA	CAE55371.1; PPE FAMILY PROTEIN	-0.203	0.347
TATLLPFEEAPEMTS	CAE55371.1; PPE FAMILY PROTEIN	-0.075	0.346
PSERFFGNLTQGRFY	POSSIBLE GLYCOSYL TRANSFERASE CAB05415	0.586	0.345
FFFNPGELLPEAAGP	CAA98382.1; IMMUNOGENIC PROTEIN MPT64	0.017	0.344
NFQRYLQAASNGAWG	PERIPLASMIC PHOSPHATE-BINDING LIPOPROTEIN PSTS3 YP_177768	0.212	0.343
PRLVPGWTKPIVIGR	CAA17111.1; PROBABLE ISOCITRATE DEHYDROGENASE	0.033	0.342
VSVEMIEAVGYRSWP	PUTATIVE CYCLOPROPANE-FATTY-ACYL-PHOSPHOLIPID SYNTHASE UFAA1 NP_854118	-0.100	0.341
FPAQNFNTTFPVGPF	YP_177963; PPE FAMILY PROTEIN	0.045	0.341
RMELLAKSGKTIVLK	PROBABLE ISOCITRATE DEHYDROGENASE CAA16247	0.315	0.340
IMAHVSYVQSGMTSQ	PROBABLE CUTINASE PRECURSOR CFP21 NP_216500	-0.033	0.340
ARGISNFHSPSDVIV	PROBABLE ISOCITRATE DEHYDROGENASE CAA16247	0.524	0.340
GYVVRWVDQGVGCSK	MTB81 (publication)	0.346	0.340
RTAHQDVPSPFGLFP	PPE FAMILY PROTEIN CAE55504	0.004	0.338
PSVMADVASGNLPAL	CONSERVED HYPOTHETICAL PROTEIN CAA15739	0.182	0.337
VWARREHPTYEDIVG	MTB48 AAK31576	-0.022	0.337
QQKWDATATELNNAL	CAE55648	0.235	0.337
LYADVPELMPTYDLP	POSSIBLE GLYCOSYL TRANSFERASE CAB05415	-0.135	0.336
VDYAFQYDGVNDFPK	PPE FAMILY PROTEIN CAE55504	0.021	0.336
CRIFTDGDYTLYADV	POSSIBLE GLYCOSYL TRANSFERASE CAB05418	0.192	0.335
CPAVIAPLMPNRLQA	CAB10947.1; PROBABLE LIPOPROTEIN LPRJ	-0.018	0.335
RVTPVAVIGMGCRLP	MYCOBACTERIUM BOVIS MYCOCEROSIC ACID SYNTHASE GENE; M95808	-0.249	0.335
CVQLSEVERQIHEIV	PPE FAMILY PROTEIN CAE55489	0.018	0.334
ISDKMGLAIPGTNWI	CAB06237.1; CONSERVED HYPOTHETICAL PROTEIN	-0.040	0.333
AATITMFTVSATYHR	POSSIBLE HEMOLYSIN-LIKE PROTEIN CAA17201	-0.202	0.331
IEMLTTLPKFPGLPG	CAB06237.1; CONSERVED HYPOTHETICAL PROTEIN	0.131	0.329
GWFNAGHTNTGGFNT	YP_177963; PPE FAMILY PROTEIN	0.462	0.329
NTRLWVYCGNGTPNE	CAB10044.1; SECRETED ANTIGEN 85-B FBPB (85B)	0.082	0.329
APKDLEKIIYFAAYV	RNA POLYMERASE BETA'-SUBUNIT AAA21417	-0.019	0.328
GAGSSGWWNQAHAAL	YP_177963; PPE FAMILY PROTEIN	0.058	0.327
LEEFDHQLTRIELRR	3-OXOACYL-[ACYL-CARRIER PROTEIN] SYNTHASE 2 KASB CAA94642	-0.036	0.326
PTPGFGNATATPSSG	YP_177963; PPE FAMILY PROTEIN	-0.199	0.323

DLVRAYHAMSSTHEA	CAA17363.1; LOW MOLECULAR WEIGHT PROTEIN ANTIGEN 7 ESXH	0.202	0.322
DTLWQADTDPLPVVF	CAA98382.1; IMMUNOGENIC PROTEIN MPT64	-0.114	0.322
GRNVVLEKKWGAPTI	60 kDa chaperonin 2 P0A521	-0.024	0.320
FNNGALWTGDHHGLV	YP_177963; PPE FAMILY PROTEIN	0.730	0.320
NTGGFNPGNVNTGWF	YP_177963; PPE FAMILY PROTEIN	0.031	0.320
AANARWGSLYDALYG	MTB81 (publication)	0.020	0.319
QAAAVEEASDTAAAN	CAE55371.1; PPE FAMILY PROTEIN	0.544	0.319
AGELARDSIYYVDAN	HYPOTHETICAL PROTEIN RV2626C NP_217142	-0.004	0.318
TAATAHATAWYGLND	MYCOBACTERIUM BOVIS MYCOCEROSIC ACID SYNTHASE GENE; M95808	0.126	0.317
SPDVKPPTWWHSLPT	POSSIBLE GLYCOSYL TRANSFERASE CAB05418	0.240	0.317
SHYGRMCPIETPEGP	RNA POLYMERASE BETA-SUBUNIT AAA21416	-0.161	0.311
FNKLLGPLPFPHHPI	POSSIBLE GLYCOSYL TRANSFERASE CAB05418	0.286	0.311
VPGRSVSRWGGFLDD	MYCOBACTERIUM BOVIS MYCOCEROSIC ACID SYNTHASE GENE; M95808	0.143	0.309
ELRHLNPLNIVNLGN	YP_177963; PPE FAMILY PROTEIN	-0.192	0.309
YAGPGPDSMLAAARA	PPE FAMILY PROTEIN CAE55489	-0.078	0.308
TAGEPNFMDLKEAAR	MTB48 AAK31576	-0.296	0.308
AVAVVAMAAIATFAA	CAB06500.1; IMMUNOGENIC PROTEIN MPT63	0.009	0.308
FIVWTSSGAIGPTWY	YP_177963; PPE FAMILY PROTEIN	0.079	0.307
QTYKWETFLTSELPQ	CAB10044.1; SECRETED ANTIGEN 85-B FBPB (85B)	-0.152	0.305
HDRAYSGYIPILGAP	MYCOBACTERIUM BOVIS MYCOCEROSIC ACID SYNTHASE GENE; M95808	-0.126	0.305
NPESTFSRIYQEIIN	PROBABLE ISOCITRATE DEHYDROGENASE CAA16247	0.074	0.304
DLVRAYQSMSGTHES	CAA16104.1; SECRETED ESAT-6 LIKE PROTEIN ESXR	0.141	0.304
SRIYQEIINFCKTNG	PROBABLE ISOCITRATE DEHYDROGENASE CAA16247	0.040	0.304
GVATAAPKTYCEELK	CAA98382.1; IMMUNOGENIC PROTEIN MPT64	0.989	0.301
KDFDGRSEFAYGSFV	CAA17245.1; HEAT SHOCK PROTEIN HSPX	0.138	0.300
QLVEENHLRWDSLGE	PROBABLE ISOCITRATE DEHYDROGENASE CAA16247	0.225	0.300
EFFHACTAVAAICHY	POSSIBLE HEMOLYSIN-LIKE PROTEIN CAA17201	0.167	0.300
RVPPRPYVMPHSPAA	CAE55371.1; PPE FAMILY PROTEIN	-0.205	0.300
VFLPSMTIGGQTYTI	YP_177963; PPE FAMILY PROTEIN	-0.091	0.300
NATDPGAAAQFNASP	LOW MOLECULAR WEIGHT T-CELL ANTIGEN TB8.4 NP_215690	-0.056	0.299
TIAYDEEARRGLERG	60 kDa chaperonin 2 P0A521	-0.106	0.298
WNNPAIQALNRDFTL	PERIPLASMIC PHOSPHATE-BINDING LIPOPROTEIN PSTS3 YP_177768	0.186	0.298
KRLPDALRDGNRIFA	MYCOBACTERIUM BOVIS MYCOCEROSIC ACID SYNTHASE GENE; M95808	0.093	0.297
FGNSGLGLPPGMGNI	YP_177963; PPE FAMILY PROTEIN	0.383	0.297
AGGLLEQAAAVEEAS	CAE55371.1; PPE FAMILY PROTEIN	0.524	0.294
WVHGDNVGNGYWQKP	MYCOBACTERIUM BOVIS ACYL-COA SYNTHASE GENE; U75685	0.423	0.294
LRIGLATAEDIRQWS	RNA POLYMERASE BETA'-SUBUNIT AAA21417	0.146	0.294
RLLGRGGMGEVYEAE	TRANSMEMBRANE SERINE/THREONINE-PROTEIN KINASE D PKND NP_215446	0.408	0.290
FGGLTTAHTPRREPR	MYCOBACTERIUM BOVIS MYCOCEROSIC ACID SYNTHASE GENE; M95808	0.197	0.286
TGDQGASLAHFADGW	MTB48 AAK31576	0.110	0.285
DGRTLREIRYGSFRR	CAA17343.1; HEAT SHOCK PROTEIN HSP	-0.071	0.284
RHGLSSLGWNLCRIF	POSSIBLE GLYCOSYL TRANSFERASE CAB05415	0.046	0.284
KASVPGGGDMGGMDF	60 kDa chaperonin 2 P0A521	0.538	0.284
EGTVVAVGPGRWDED	CAB01005.1; 10 KDA CHAPERONIN GROES	0.481	0.283
AIRMNCRAVADHLTR	CONSERVED HYPOTHETICAL PROTEIN CAB08634	-0.196	0.282
LMDAAIFKDTLIMSY	CONSERVED HYPOTHETICAL PROTEIN TB16.3CAD97060	-0.131	0.282
GYPVHKPVTAGWNGY	CONSERVED HYPOTHETICAL PROTEIN CAB08634	0.102	0.281
ATATATLLPFEEAPE	CAE55371.1; PPE FAMILY PROTEIN	-0.236	0.280
TNGQFDPTTMGTVPN	PROBABLE ISOCITRATE DEHYDROGENASE CAA16247	-0.030	0.278
PFGFIETPYRKVVDG	RNA POLYMERASE BETA-SUBUNIT AAA21416	0.693	0.276
ATVSPAMVAANRTRL	PPE FAMILY PROTEIN CAE55504	-0.161	0.275
GMPVLFWLDPYRPHE	PROBABLE ISOCITRATE DEHYDROGENASE CAA16247	0.462	0.275
QVGDRVGGFSEGGCW	MYCOBACTERIUM BOVIS MYCOCEROSIC ACID SYNTHASE GENE; M95808	0.137	0.275
MIFVFIAGSYTPFAL	POSSIBLE HEMOLYSIN-LIKE PROTEIN CAA17201	0.010	0.274
PLNWLRRKHGLSSLG	POSSIBLE GLYCOSYL TRANSFERASE CAB05418	-0.125	0.273
LATSLRNAAKAYGEV	MTB48 AAK31576	-0.161	0.273
TAVFTGLTHEDYLVL	MYCOBACTERIUM BOVIS MYCOCEROSIC ACID SYNTHASE GENE; M95808	0.190	0.272
VNEAEYGEMWAQDAA	CAE55371.1; PPE FAMILY PROTEIN	0.175	0.272
MAKTIAYDEEARRGL	60 kDa chaperonin 2 P0A521	0.145	0.271
NFGAGSSGWWNQAQT	YP_177963; PPE FAMILY PROTEIN	0.090	0.271
MVAPDPNGERAGHAI	3-OXOACYL-[ACYL-CARRIER PROTEIN] SYNTHASE 2 KASB CAA94642	-0.024	0.271
YTEPILHRRRREFKA	MTB81 (publication)	-0.034	0.270
AAAAQRRCGSPAWNL	PERIPLASMIC PHOSPHATE-BINDING LIPOPROTEIN PSTS3 YP_177768	-0.135	0.270
FRADFLSELDAPAQA	CAB01474.1; CONSERVED HYPOTHETICAL PROTEIN CFP17	-0.086	0.270
TRYMHVVVASYNEFG	POSSIBLE GLYCOSYL TRANSFERASE CAB05419	0.015	0.270

PLLEYAARFITPVHP	PPE FAMILY PROTEIN CAE55504	-0.078	0.270
QSMSGTHESNTMAML	CAA16104.1; SECRETED ESAT-6 LIKE PROTEIN ESXR	0.087	0.269
NLCAPDDPICTGGGN	PROBABLE CUTINASE PRECURSOR CFP21 NP_216500	0.353	0.267
MVSMANNHMSMTNSG	CAE55371.1; PPE FAMILY PROTEIN	-0.031	0.267
EWPEDRQSLSWTLES	CONSERVED HYPOTHETICAL PROTEIN TB16.3CAD97060	-0.035	0.267
RFVEIGKADVYGNTR	MYCOBACTERIUM BOVIS MYCOCEROSIC ACID SYNTHASE GENE; M95808	0.162	0.265
TDGDHTLYADVPELM	POSSIBLE GLYCOSYL TRANSFERASE CAB05415	0.348	0.265
GFNPGNVNTGWLNTG	YP_177963; PPE FAMILY PROTEIN	0.031	0.264
IINFCKTNGQFDPTT	PROBABLE ISOCITRATE DEHYDROGENASE CAA16247	0.692	0.262
IAHTLGLHGPAMTFD	MYCOBACTERIUM BOVIS MYCOCEROSIC ACID SYNTHASE GENE; M95808	0.063	0.261
TAEDIRQWSYGEVKK	RNA POLYMERASE BETA'-SUBUNIT AAA21417	0.347	0.260
GSLYDALYGTDVIPE	MTB81 (publication)	-0.286	0.259
KHQELFDDLGVNVNN	PROBABLE ISOCITRATE DEHYDROGENASE CAA16247	0.176	0.258
GMGMPMGAAHQGQGG	MTB48 AAK31576	-0.094	0.258
TPGSITYNEWSFAQA	PERIPLASMIC PHOSPHATE-BINDING LIPOPROTEIN PSTS3 YP_177768	0.164	0.257
LIDDMVAACLKWEGG	CAA17111.1; PROBABLE ISOCITRATE DEHYDROGENASE	-0.010	0.256
LAMVDSARGISNFHS	PROBABLE ISOCITRATE DEHYDROGENASE CAA16247	0.062	0.255
SAATSSTPREAPYEL	CAA98382.1; IMMUNOGENIC PROTEIN MPT64	-0.045	0.255
LNAGNTNTGGFNPGN	YP_177963; PPE FAMILY PROTEIN	0.055	0.251
VNSANIYAGPGPDSM	PPE FAMILY PROTEIN CAE55489	0.067	0.251
LDAFKEMTLLELSDF	CAB07109.1; PROBABLE 50S RIBOSOMAL PROTEIN L7/L12 RPLL	-0.186	0.251
THAVEQTGRSLDMSV	MYCOBACTERIUM BOVIS MYCOCEROSIC ACID SYNTHASE GENE; M95808	-0.095	0.250
VNDFPKYPLNVFATA	PPE FAMILY PROTEIN CAE55504	0.019	0.250
LRAIFGEKAREVRDT	RNA POLYMERASE BETA-SUBUNIT AAA21416	0.052	0.249
TGSLNAGNTNTGGFN	YP_177963; PPE FAMILY PROTEIN	-0.116	0.248
CLGSGRWPGRLLGSV	HYPOTHETICAL PROTEIN RV2623 NP_217139	0.068	0.248
GEPRPGNYRYAINNS	3-OXOACYL-[ACYL-CARRIER PROTEIN] SYNTHASE 2 KASB CAA94642	-0.129	0.248
HIWYFKGVPSRLGYL	RNA POLYMERASE BETA'-SUBUNIT AAA21417	0.191	0.245
FNIRCFSNPALVTRY	POSSIBLE GLYCOSYL TRANSFERASE CAB05419	0.430	0.245
LSQDRFADFPALPLD	CAB09453.1; PROBABLE SERINE PROTEASE PEPA	-0.142	0.244
EYTVFAPTNAAFDKL	CAA98350.1; CELL SURFACE LIPOPROTEIN MPT83	-0.153	0.244
YNLRYRVLADWDFNI	POSSIBLE GLYCOSYL TRANSFERASE CAB05419	0.304	0.243
AGGYKAADMWGPSSD	CAB10044.1; SECRETED ANTIGEN 85-B FBPB (85B)	0.281	0.241
SITYNEWSFAVGKQL	PERIPLASMIC PHOSPHATE-BINDING LIPOPROTEIN PSTS2 YP_177769	0.270	0.241
VGSPYAVEYVWGRQV	MCE-FAMILY PROTEIN MCE1AYP_177701	-0.066	0.240
ESSAAFQAAHARFVA	CONSERVED HYPOTHETICAL PROTEIN TB9.8 CAD93159	0.072	0.240
FGTIGPYNLRYRVLA	POSSIBLE GLYCOSYL TRANSFERASE CAB05419	-0.150	0.239
PKRARMLMDAAIFKD	CONSERVED HYPOTHETICAL PROTEIN TB16.3CAD97060	0.282	0.239
FGFGNIGNANFGFGN	YP_177963; PPE FAMILY PROTEIN	0.865	0.239
AERVGISQMHVSRLL	ALTERNATE RNA POLYMERASE SIGMA FACTOR SIGF CAB07069	-0.022	0.236
VDSARGISNFHSPSD	PROBABLE ISOCITRATE DEHYDROGENASE CAA16247	0.655	0.235
HIELAAPVTHIWYFK	RNA POLYMERASE BETA'-SUBUNIT AAA21417	-0.097	0.235
VGCAQPACLRAYPQC	MYCOBACTERIUM BOVIS MYCOCEROSIC ACID SYNTHASE GENE; M95808	0.027	0.234
FMDQNNPLSGLTHKR	RNA POLYMERASE BETA-SUBUNIT AAA21416	0.136	0.234
ETLTAAAQYMREHDI	HYPOTHETICAL PROTEIN RV2626C NP_217142	0.168	0.233
TDWYRPVAGDFTPAA	CAA17343.1; HEAT SHOCK PROTEIN HSP	-0.068	0.233
QLMSGYFADTDGIPP	MYCOBACTERIUM BOVIS ACYL-COA SYNTHASE GENE; U75685	0.225	0.232
GTPCALGSAKSNMGH	MYCOBACTERIUM BOVIS MYCOCEROSIC ACID SYNTHASE GENE; M95808	0.077	0.231
RRSVAVTPEQRPLYR	MYCOBACTERIUM BOVIS MYCOCEROSIC ACID SYNTHASE GENE; M95808	-0.224	0.230
MTINYQFGDVDAHGA	PUTATIVE ESAT-6 LIKE PROTEIN ESXN YP_177838	-0.035	0.230
NAGSNNVGFGNMGLG	YP_177963; PPE FAMILY PROTEIN	0.147	0.230
LEMRRPTYVFAAVDH	CONSERVED HYPOTHETICAL PROTEIN CAB08634	-0.201	0.230
PNELGGANIPAEFLE	CAB10044.1; SECRETED ANTIGEN 85-B FBPB (85B)	0.144	0.229
DQIDIPGMSLFDIHE	YP_177963; PPE FAMILY PROTEIN	0.589	0.229
KTINLCAPDDPICTG	PROBABLE CUTINASE PRECURSOR CFP21 NP_216500	0.231	0.227
QFNASPVAQSYLRNF	LOW MOLECULAR WEIGHT T-CELL ANTIGEN TB8.4 NP_215690	0.217	0.227
WMHLMASDFHAFSAA	MYCOBACTERIUM BOVIS ACYL-COA SYNTHASE GENE; U75685	0.041	0.226
VGTDEALLDIYRKLR	RNA POLYMERASE BETA-SUBUNIT AAA21416	-0.334	0.226
FNAGNTNTGGFNPGN	YP_177963; PPE FAMILY PROTEIN	0.087	0.225
EAGFRSGYLDVYQWT	PUTATIVE CYCLOPROPANE-FATTY-ACYL-PHOSPHOLIPID SYNTHASE UFAA1 NP_854118	0.158	0.224
GFNPGNVNTGWFNAG	YP_177963; PPE FAMILY PROTEIN	-0.113	0.224
AGFASKTPANQAISM	PERIPLASMIC PHOSPHATE-BINDING LIPOPROTEIN PSTS1 YP_177770	-0.099	0.224
PSDMTNHPRYSPPPQ	CAA17582.1; PROBABLE SERINE PROTEASE PEPD	0.591	0.223
IYSKYGGTEIKYNGE	CAB01005.1; 10 KDA CHAPERONIN GROES	0.102	0.220
KAVNPESTFSRIYQE	PROBABLE ISOCITRATE DEHYDROGENASE CAA16247	0.543	0.219

FGSWNLHRATLGQPL	MYCOBACTERIUM BOVIS MYCOCEROSIC ACID SYNTHASE GENE; M95808	0.030	0.219
FGPAATTDWYRPVAG	CAA17343.1; HEAT SHOCK PROTEIN HSP	-0.187	0.219
DEEKADLLVVGNVGL	CAB08889.1; IRON-REGULATED CONSERVED HYPOTHETICAL PROTEIN	-0.109	0.219
HQTFSLGPLVVPDIV	YP_177963; PPE FAMILY PROTEIN	-0.112	0.218
KVSHPIVFGHAVRIF	PROBABLE ISOCITRATE DEHYDROGENASE CAA16247	-0.064	0.218
AASAAPSIPAANMPP	CAA17582.1; PROBABLE SERINE PROTEASE PEPD	-0.115	0.217
AQRRCGSPAWNLPVV	PERIPLASMIC PHOSPHATE-BINDING LIPOPROTEIN PSTS3 YP_177768	-0.239	0.217
DFWGGAGSVACQEFI	PUTATIVE ESAT-6 LIKE PROTEIN ESXN YP_177838	-0.070	0.216
KNAAQQLVLSADNMR	MTB48 AAK31576	-0.258	0.215
NGYIPIPDEFKSRLS	PERIPLASMIC PHOSPHATE-BINDING LIPOPROTEIN PSTS3 YP_177768	-0.132	0.215
NTPAIAVNEAEYGEM	CAE55371.1; PPE FAMILY PROTEIN	-0.159	0.215
VRHLVRITRELAEFE	MYCOBACTERIUM BOVIS MYCOCEROSIC ACID SYNTHASE GENE; M95808	0.036	0.215
INAFSVGSGQTYGVD	CAB09453.1; PROBABLE SERINE PROTEASE PEPA	-0.248	0.214
FSAASAFQSVVWGLT	CAE55371.1; PPE FAMILY PROTEIN	-0.018	0.214
WDATATELNNALQNL	CAE55648	-0.138	0.214
PLPFRHHAIHTIPSE	POSSIBLE GLYCOSYL TRANSFERASE CAB05415	0.065	0.212
AAPEPVARRQRILFV	POSSIBLE GLYCOSYL TRANSFERASE CAB05418	-0.020	0.212
GNVNTGAFNSGSFNN	YP_177963; PPE FAMILY PROTEIN	-0.143	0.211
QGLAGKRRATIEQLL	MTB81 (publication)	0.031	0.210
VVSDEIVYLTADEED	RNA POLYMERASE BETA-SUBUNIT AAA21416	0.157	0.210
WIQKYIFPGGLLPST	PUTATIVE CYCLOPROPANE-FATTY-ACYL-PHOSPHOLIPID SYNTHASE UFAA1 NP_854118	-0.201	0.210
TAQENAMEQFVYAYV	PERIPLASMIC PHOSPHATE-BINDING LIPOPROTEIN PSTS2 YP_177769	0.016	0.210
STLLYPLFNLWGPAF	PERIPLASMIC PHOSPHATE-BINDING LIPOPROTEIN PSTS1 YP_177770	0.241	0.209
PLLFVNALGDNWIVG	YP_177963; PPE FAMILY PROTEIN	-0.044	0.209
DQILPVYAEYQQRSE	MTB48 AAK31576	-0.185	0.207
WKVDAAKGVPDWAAR	RNA POLYMERASE BETA-SUBUNIT AAA21416	-0.000	0.207
DETAWRNGDWYVARL	MYCOBACTERIUM BOVIS MYCOCEROSIC ACID SYNTHASE GENE; M95808	-0.103	0.206
WENAGSPEVDTNRLM	3-OXOACYL-[ACYL-CARRIER PROTEIN] SYNTHASE 2 KASB CAA94642	0.009	0.206
YDALYGTDVIPETDG	MTB81 (publication)	-0.167	0.205
RFPLPDVIWTRLFGV	POSSIBLE GLYCOSYL TRANSFERASE CAB05415	0.067	0.205
WRGNYEGLFSYSYSL	YP_177963; PPE FAMILY PROTEIN	0.174	0.205
PGNVNTGWFNAGHTN	YP_177963; PPE FAMILY PROTEIN	0.143	0.205
YRPMAPNFDDSIAFL	MTB81 (publication)	0.062	0.202
VMDTGASLAPYNHME	MCE-FAMILY PROTEIN MCE1AYP_177701	0.463	0.202
WLRRKHGLSSLGWDL	POSSIBLE GLYCOSYL TRANSFERASE CAB05418	0.148	0.202
KVLAAMYQGTIKTWD	PERIPLASMIC PHOSPHATE-BINDING LIPOPROTEIN PSTS1 YP_177770	0.015	0.201
RRTTDDDMAGRDLGN	MYCOBACTERIUM BOVIS ACYL-COA SYNTHASE GENE; U75685	-0.037	0.201
RPGNYRYAINNSFGF	3-OXOACYL-[ACYL-CARRIER PROTEIN] SYNTHASE 2 KASB CAA94642	0.133	0.200
LVRHDRAYSGYIPIL	MYCOBACTERIUM BOVIS MYCOCEROSIC ACID SYNTHASE GENE; M95808	-0.119	0.199
ASFLDQVHFQPLPPA	PERIPLASMIC PHOSPHATE-BINDING LIPOPROTEIN PSTS1 YP_177770	-0.205	0.196
YVWACKNYDGDVQSD	CAA17111.1; PROBABLE ISOCITRATE DEHYDROGENASE	0.135	0.196
FNTGNVNTGAFNSGS	YP_177963; PPE FAMILY PROTEIN	0.057	0.195
AEAADAIRAMSNAEH	MYCOBACTERIUM BOVIS MYCOCEROSIC ACID SYNTHASE GENE; M95808	-0.254	0.192
ERLLRAIFGEKAREV	RNA POLYMERASE BETA-SUBUNIT AAA21416	-0.621	0.192
TATELNNALQNLART	CAE55648	-0.366	0.192
GILWRGNFEGLFGLN	YP_177963; PPE FAMILY PROTEIN	0.137	0.190
MHGPAEVAFTCELFS	MTB81 (publication)	-0.074	0.190
IGGYYAPDSDMTTAV	PROBABLE ISOCITRATE DEHYDROGENASE CAA16247	0.239	0.188
GMSLFDIHELVNIGP	YP_177963; PPE FAMILY PROTEIN	-0.155	0.187
PAGKPPTWWDSLPTD	POSSIBLE GLYCOSYL TRANSFERASE CAB05415	-0.075	0.186
WAQDAAAMFGYAAAT	CAE55371.1; PPE FAMILY PROTEIN	-0.213	0.183
YNQLLGPLPFRHHAI	POSSIBLE GLYCOSYL TRANSFERASE CAB05415	0.302	0.182
NILRDYLTDLFPILE	PROBABLE ISOCITRATE DEHYDROGENASE CAA16247	-0.379	0.182
LVHAVSPEVATWLEV	HYPOTHETICAL PROTEIN RV2623 NP_217139	-0.091	0.182
DANRALMGANMQRQA	RNA POLYMERASE BETA-SUBUNIT AAA21416	-0.071	0.181
TARQLATWVEEHQDC	MYCOBACTERIUM BOVIS MYCOCEROSIC ACID SYNTHASE GENE; M95808	0.101	0.181
SIPAANMPPGSVEQV	CAA17582.1; PROBABLE SERINE PROTEASE PEPD	-0.194	0.178
VVRFQEAANKQKQEL	CAA17966.1; 10 KDA CULTURE FILTRATE ANTIGEN ESXB	-0.106	0.177
MNPDIEKDQTSDEVT	CAB01474.1; CONSERVED HYPOTHETICAL PROTEIN CFP17	0.061	0.177
TILKAYDGMFKDEFE	CAA17111.1; PROBABLE ISOCITRATE DEHYDROGENASE	0.260	0.177
YQGTIKTWDDPQIAA	PERIPLASMIC PHOSPHATE-BINDING LIPOPROTEIN PSTS1 YP_177770	-0.088	0.177
GTTVDFPAVPGALGE	PERIPLASMIC PHOSPHATE-BINDING LIPOPROTEIN PSTS1 YP_177770	-0.122	0.176
RSVSRWGGFLDDVAG	MYCOBACTERIUM BOVIS MYCOCEROSIC ACID SYNTHASE GENE; M95808	0.208	0.176
MSLSFSDPRFDDVKA	RNA POLYMERASE BETA-SUBUNIT AAA21416	-0.060	0.176
DPAPQPSQLRPGRVP	TRANSMEMBRANE SERINE/THREONINE-PROTEIN KINASE D PKND	-0.070	0.175

	NP_215446		
GLGNAGSGNVGWANM	YP_177963; PPE FAMILY PROTEIN	-0.163	0.175
GTEIKYNGEEYLILS	CAB01005.1; 10 KDA CHAPERONIN GROES	0.129	0.175
SQSATIATFEMRYLQ	PPE FAMILY PROTEIN CAE55504	-0.002	0.174
VSRRHAEFRLENNEF	CAB01474.1; CONSERVED HYPOTHETICAL PROTEIN CFP17	-0.194	0.173
WSQLYRRTLNVAREL	MYCOBACTERIUM BOVIS ACYL-COA SYNTHASE GENE; U75685	0.226	0.173
AVLSSAWQGDTGITY	CAA16104.1; SECRETED ESAT-6 LIKE PROTEIN ESXR	-0.151	0.172
KLQFLHDAFPNEFPD	YP_177963; PPE FAMILY PROTEIN	0.231	0.172
LSIVMPVGGQSSFYS	CAB10044.1; SECRETED ANTIGEN 85-B FBPB (85B)	-0.121	0.170
VNTGWFNAGHTNTGG	YP_177963; PPE FAMILY PROTEIN	0.195	0.170
NTTFPVGPFFGLGVV	YP_177963; PPE FAMILY PROTEIN	0.568	0.169
AAGFSGRAQVGKGMW	MTB81 (publication)	0.129	0.169
AAANSWVDVFAHWPR	MYCOBACTERIUM BOVIS MYCOCEROSIC ACID SYNTHASE GENE; M95808	0.002	0.169
AGGKMYGADGKLKDT	PROBABLE ISOCITRATE DEHYDROGENASE CAA16247	-0.058	0.168
AKEAADEAKAKLEAA	CAB07109.1; PROBABLE 50S RIBOSOMAL PROTEIN L7/L12 RPLL	0.199	0.168
MLTSGGSGGSGQPSI	YP_177963; PPE FAMILY PROTEIN	-0.067	0.167
RGACVEQYRQDQFAR	MYCOBACTERIUM BOVIS ACYL-COA SYNTHASE GENE; U75685	0.174	0.166
VWQGDVGTSVLSWLS	MYCOBACTERIUM BOVIS MYCOCEROSIC ACID SYNTHASE GENE; M95808	0.584	0.166
YQNFAVTNDGVIFFF	CAA98382.1; IMMUNOGENIC PROTEIN MPT64	0.525	0.166
TEQKDFDGRSEFAYG	CAA17245.1; HEAT SHOCK PROTEIN HSPX	0.208	0.165
VDFPAVPGALGENGN	PERIPLASMIC PHOSPHATE-BINDING LIPOPROTEIN PSTS1 YP_177770	-0.268	0.165
LLDLDAPNGYTFKED	MYCOBACTERIUM BOVIS ACYL-COA SYNTHASE GENE; U75685	-0.116	0.164
FDKGYISGYFVTDPE	60 kDa chaperonin 2 P0A521	0.155	0.163
ERNDPTQQIPKLVAN	CAB10044.1; SECRETED ANTIGEN 85-B FBPB (85B)	-0.314	0.163
ANRLLRRTATHLPLR	PUTATIVE CYCLOPROPANE-FATTY-ACYL-PHOSPHOLIPID SYNTHASE UFAA1 NP_854118	-0.139	0.162
LDRLLFKRNICHQAI	POSSIBLE GLYCOSYL TRANSFERASE CAB05419	-0.073	0.162
MGRDIKVQFQSGGNN	CAB10044.1; SECRETED ANTIGEN 85-B FBPB (85B)	-0.007	0.162
GVVLAGGESRRMGRD	CAA16030.1; PROBABLE MOLYBDOPTERIN-GUANINE DINUCLEOTIDE BIOSYNTHESIS PROTEIN	-0.174	0.161
SDRRAPKAVKEYARK	PROBABLE ISOCITRATE DEHYDROGENASE CAA16247	-0.015	0.161
AMAVVGGALLYLTIM	CAB06237.1; CONSERVED HYPOTHETICAL PROTEIN	-0.158	0.159
TAQTLQAFLHWAITD	PERIPLASMIC PHOSPHATE-BINDING LIPOPROTEIN PSTS1 YP_177770	-0.045	0.159
GTGNIGFFNTGTGNW	YP_177963; PPE FAMILY PROTEIN	0.372	0.158
DVVATIEYLVRLHEG	RNA POLYMERASE BETA-SUBUNIT AAA21416	-0.067	0.158
RAPTESTRRCWTLVS	MYCOBACTERIUM BOVIS MYCOCEROSIC ACID SYNTHASE GENE; M95808	0.014	0.157
IGTYCPAVIAPLMPN	CAB10947.1; PROBABLE LIPOPROTEIN LPRJ	-0.132	0.156
IGICAPILGGYPAVL	MYCOBACTERIUM BOVIS ACYL-COA SYNTHASE GENE; U75685	0.029	0.156
PPQPTQYRQPYEALG	CAA17582.1; PROBABLE SERINE PROTEASE PEPD	-0.264	0.155
ILGGYPAVLTSPVSF	MYCOBACTERIUM BOVIS ACYL-COA SYNTHASE GENE; U75685	0.023	0.154
QVGSQFGPYQLLRLL	TRANSMEMBRANE SERINE/THREONINE-PROTEIN KINASE D PKND NP_215446	-0.175	0.154
DALARRIGRHGLIGF	PUTATIVE CYCLOPROPANE-FATTY-ACYL-PHOSPHOLIPID SYNTHASE UFAA1 NP_854118	-0.025	0.150
VRLPDGNGIELCRDL	TWO COMPONENT TRANSCRIPTIONAL REGULATORY PROTEIN DEVR NP_217649	-0.163	0.150
LPDVIWTRLFGVRLV	POSSIBLE GLYCOSYL TRANSFERASE CAB05415	-0.120	0.150
NNKAALEPVNPPKPP	MTB48 AAK31576	-0.031	0.150
FALAQSLDPSRYEVH	POSSIBLE GLYCOSYL TRANSFERASE CAB05415	-0.241	0.150
LLDNDKSPSRKTGEL	PROBABLE ISOCITRATE DEHYDROGENASE CAA16247	-0.072	0.149
NYEYAIVNNRQKDAA	PERIPLASMIC PHOSPHATE-BINDING LIPOPROTEIN PSTS1 YP_177770	-0.122	0.149
YHPQQFIYAGSLSAL	CAB10044.1; SECRETED ANTIGEN 85-B FBPB (85B)	-0.088	0.149
HTHDGTRTWRTGRQA	CAB10947.1; PROBABLE LIPOPROTEIN LPRJ	0.423	0.147
SYNTGSLNAGNTNTG	YP_177963; PPE FAMILY PROTEIN	0.034	0.146
SLPAALRACACLQPH	MYCOBACTERIUM BOVIS ACYL-COA SYNTHASE GENE; U75685	-0.043	0.144
LRAQDDYNGWDINTP	CAB10044.1; SECRETED ANTIGEN 85-B FBPB (85B)	-0.163	0.144
QHVARRPGESPPSII	MYCOBACTERIUM BOVIS ACYL-COA SYNTHASE GENE; U75685	0.176	0.144
TGIANSGNVDTGALM	YP_177963; PPE FAMILY PROTEIN	-0.266	0.143
ASPYVAWMSVTAGQA	CAE55371.1; PPE FAMILY PROTEIN	-0.030	0.143
TSAEFTRRRYAEITN	CONSERVED HYPOTHETICAL PROTEIN CAB08634	-0.075	0.141
GLNVGITIPEFPIHW	YP_177963; PPE FAMILY PROTEIN	0.061	0.141
LDAVERREATWRDVR	CONSERVED HYPOTHETICAL PROTEIN CAB08634	0.465	0.141
EDLHRCHEHRPELAM	PROBABLE ISOCITRATE DEHYDROGENASE CAA16247	0.095	0.141
AAVDAADKVLGYRNW	MTB81 (publication)	-0.085	0.140
FGLQLELTEGMRFDK	60 kDa chaperonin 2 P0A521	0.161	0.136
QQWNFAGIEAAASAI	CAE55648	-0.112	0.136
VGNGYWQKPDESERT	MYCOBACTERIUM BOVIS ACYL-COA SYNTHASE GENE; U75685	-0.230	0.136

PYRPHENELIKKVKT	PROBABLE ISOCITRATE DEHYDROGENASE CAA16247	0.019	0.134
ADGFVRSEGCAMVLL	MYCOBACTERIUM BOVIS MYCOCEROSIC ACID SYNTHASE GENE; M95808	0.114	0.132
EFPIHWTSTGGIGPI	YP_177963; PPE FAMILY PROTEIN	-0.050	0.132
KWPPTPGSPLFPDLP	CAB06237.1; CONSERVED HYPOTHETICAL PROTEIN	-0.129	0.131
LNMEALERAGAGVLL	POSSIBLE GLYCOSYL TRANSFERASE CAB05418	-0.042	0.131
VSADYITVMHDNGTR	RNA POLYMERASE BETA-SUBUNIT AAA21416	-0.082	0.131
FAHWPRAQGLPVSAI	MYCOBACTERIUM BOVIS MYCOCEROSIC ACID SYNTHASE GENE; M95808	-0.106	0.131
ARVPPYKTAGLGLVL	MCE-FAMILY PROTEIN MCE1AYP_177701	0.067	0.130
PTYDLPANHEYLGPV	POSSIBLE GLYCOSYL TRANSFERASE CAB05415	0.109	0.130
FPSFAGLRPTFDTRL	CAA17245.1; HEAT SHOCK PROTEIN HSPX	0.182	0.129
AQTLLENLFFKEKRY	RNA POLYMERASE BETA-SUBUNIT AAA21416	0.059	0.129
WSPQARRRFPLPDVP	POSSIBLE GLYCOSYL TRANSFERASE CAB05418	-0.016	0.128
PDRSAERCGSPAWDL	PERIPLASMIC PHOSPHATE-BINDING LIPOPROTEIN PSTS2 YP_177769	0.058	0.126
FRGEFTPKTQLTMLS	MCE-FAMILY PROTEIN MCE1AYP_177701	-0.177	0.125
SLAPYNHMEVGSPYA	MCE-FAMILY PROTEIN MCE1AYP_177701	0.171	0.125
EEQPSDMTNHPRYSP	CAA17582.1; PROBABLE SERINE PROTEASE PEPD	0.389	0.125
LTYEHRLIDDMVAAC	CAA17111.1; PROBABLE ISOCITRATE DEHYDROGENASE	-0.235	0.124
PAEFLENFVRSSNLK	CAB10044.1; SECRETED ANTIGEN 85-B FBPB (85B)	-0.334	0.123
AMAAQLQAVPGAAQY	LOW MOLECULAR WEIGHT T-CELL ANTIGEN TB8.4 NP_215690	-0.168	0.123
KEAKDLVDGAPKPLL	CAB07109.1; PROBABLE 50S RIBOSOMAL PROTEIN L7/L12 RPLL	-0.173	0.122
QGNVTSIHSLLDEGK	CAE55648	-0.122	0.121
VRPFVLARSLDPSRY	POSSIBLE GLYCOSYL TRANSFERASE CAB05418	0.051	0.120
LARIMGASITSDGFH	3-OXOACYL-[ACYL-CARRIER PROTEIN] SYNTHASE 2 KASB CAA94642	0.152	0.120
LLVAIMPWEGHNYED	RNA POLYMERASE BETA-SUBUNIT AAA21416	-0.184	0.119
GRLIEMLTTLPKFPG	CAB06237.1; CONSERVED HYPOTHETICAL PROTEIN	-0.512	0.119
SNNVGFGNMGLGNIG	YP_177963; PPE FAMILY PROTEIN	0.327	0.118
VGKGMWTMTELMADM	MTB81 (publication)	-0.166	0.118
GVAANLGRAASVGSL	CAE55371.1; PPE FAMILY PROTEIN	-0.150	0.117
IPEDGGVVLGMYNFK	CAA17111.1; PROBABLE ISOCITRATE DEHYDROGENASE	-0.128	0.117
AAAEVFGEAAEVRDI	MYCOBACTERIUM BOVIS MYCOCEROSIC ACID SYNTHASE GENE; M95808	0.247	0.116
AMLGHAGDMAGYAGT	CAA17363.1; LOW MOLECULAR WEIGHT PROTEIN ANTIGEN 7 ESXH	-0.106	0.116
TWYSVGRIYNAGDLF	YP_177963; PPE FAMILY PROTEIN	0.048	0.114
LSKTVEVTADAASIM	CONSERVED HYPOTHETICAL PROTEIN TB18.5CAD93033	-0.118	0.113
ACRPFDRDRDGFVFG	3-OXOACYL-[ACYL-CARRIER PROTEIN] SYNTHASE 2 KASB CAA94642	0.114	0.112
GYISGYFVTDPERQE	60 kDa chaperonin 2 P0A521	0.024	0.112
NGYGDFQHTTEEAIR	CONSERVED HYPOTHETICAL PROTEIN CAB08634	0.160	0.111
PTFAVHDTLFYCVAN	SECRETED L-ALANINE DEHYDROGENASE ALD CAA15575	-0.027	0.110
EYLVRLHEGQTTMTV	RNA POLYMERASE BETA-SUBUNIT AAA21416	-0.062	0.110
EVHFACDPRYNQLLG	POSSIBLE GLYCOSYL TRANSFERASE CAB05415	0.079	0.110
TAMADALNGHHPGDV	CAB09453.1; PROBABLE SERINE PROTEASE PEPA	0.455	0.110
AEAGVRLAFVCAVDM	CAA16030.1; PROBABLE MOLYBDOPTERIN-GUANINE DINUCLEOTIDE BIOSYNTHESIS PROTEIN	-0.123	0.110
GRWDEDGEKRIPLDV	CAB01005.1; 10 KDA CHAPERONIN GROES	0.256	0.110
AAPSIPAANMPPGSV	CAA17582.1; PROBABLE SERINE PROTEASE PEPD	-0.186	0.107
RDQVIPPTLNLVNLD	3-OXOACYL-[ACYL-CARRIER PROTEIN] SYNTHASE 2 KASB CAA94642	0.149	0.106
RISGMPVLFWLDPYR	PROBABLE ISOCITRATE DEHYDROGENASE CAA16247	-0.154	0.106
TWRDVRKHLESRDAK	CONSERVED HYPOTHETICAL PROTEIN CAB08634	0.438	0.106
GEVLLTENVEAGDIW	PROBABLE ISOCITRATE DEHYDROGENASE CAA16247	0.120	0.104
LELSDFVKKFEETFE	CAB07109.1; PROBABLE 50S RIBOSOMAL PROTEIN L7/L12 RPLL	0.179	0.104
VNLVDTLNSGQYTVF	CAA98373.1; MAJOR SECRETED IMMUNOGENIC PROTEIN	0.127	0.104
FEEAPEMTSAGGLLE	CAE55371.1; PPE FAMILY PROTEIN	0.168	0.103
IAVTYNLNSVSSLNL	PERIPLASMIC PHOSPHATE-BINDING LIPOPROTEIN PSTS3 YP_177768	-0.095	0.102
TSQLSQFMDQNNPLS	RNA POLYMERASE BETA-SUBUNIT AAA21416	-0.095	0.102
ARDIMNAGVTCVGEH	HYPOTHETICAL PROTEIN RV2626C NP_217142	0.109	0.101
AMILAAYHPQQFIYA	CAB10044.1; SECRETED ANTIGEN 85-B FBPB (85B)	-0.184	0.101
EKVAKEAADEAKAKL	CAB07109.1; PROBABLE 50S RIBOSOMAL PROTEIN L7/L12 RPLL	0.249	0.100
YPADPKTDQEKAIKE	PROBABLE ISOCITRATE DEHYDROGENASE CAA16247	-0.161	0.100
KLLSSILTYHVIAGQ	CAA98350.1; CELL SURFACE LIPOPROTEIN MPT83	-0.199	0.099
AGFPQHIESALRLVC	POSSIBLE GLYCOSYL TRANSFERASE CAB05418	-0.316	0.098
CALGSAKSNMGHSTA	MYCOBACTERIUM BOVIS MYCOCEROSIC ACID SYNTHASE GENE; M95808	-0.064	0.097
NLVDTLNGGEYTVFA	CAA98350.1; CELL SURFACE LIPOPROTEIN MPT83	-0.007	0.097
SDALRQTARQLATWV	MYCOBACTERIUM BOVIS MYCOCEROSIC ACID SYNTHASE GENE; M95808	-0.046	0.097
AVHDTLFYCVANMPA	SECRETED L-ALANINE DEHYDROGENASE ALD CAA15575	-0.177	0.095
TAAAQYMREHDIGAL	HYPOTHETICAL PROTEIN RV2626C NP_217142	-0.147	0.095
VTRAKVRRERMGHIE	RNA POLYMERASE BETA'-SUBUNIT AAA21417	0.272	0.095
CATITPDEARVEEFN	CAA17111.1; PROBABLE ISOCITRATE DEHYDROGENASE	0.333	0.095

GLTVAVAGAAILVAG	LIPOPROTEIN LPQH PRECURSOR P0A5J0	0.017	0.095
IDHFGNRRLRTVGEL	RNA POLYMERASE BETA-SUBUNIT AAA21416	0.240	0.094
IAAHLMDPAPQPSQL	TRANSMEMBRANE SERINE/THREONINE-PROTEIN KINASE D PKND NP_215446	-0.091	0.092
QWSYGEVKKPETINY	RNA POLYMERASE BETA'-SUBUNIT AAA21417	-0.174	0.092
IDKWHRRRVIEPIDM	MTB81 (publication)	0.315	0.091
IMNAGVTCVGEHETL	HYPOTHETICAL PROTEIN RV2626C NP_217142	0.446	0.090
LDQYGSIPLPKSFQA	PERIPLASMIC PHOSPHATE-BINDING LIPOPROTEIN PSTS2 YP_177769	-0.150	0.090
QNAGGTHPTTTYKAF	CAA98382.1; IMMUNOGENIC PROTEIN MPT64	0.022	0.090
GLPSIPNLFPGLPGL	CAB06237.1; CONSERVED HYPOTHETICAL PROTEIN	-0.336	0.090
PDWAARLPDELLEAH	RNA POLYMERASE BETA-SUBUNIT AAA21416	-0.025	0.088
VDGANDDWNPASKRG	POSSIBLE HEMOLYSIN CAA16235	-0.072	0.086
LAALPDFLGGFAGLP	CAB06237.1; CONSERVED HYPOTHETICAL PROTEIN	-0.082	0.085
GLGNAGSGNVGFGNM	YP_177963; PPE FAMILY PROTEIN	-0.167	0.085
RRVIEPIDMDAYRQF	MTB81 (publication)	-0.078	0.084
VSTANATVYMIDSVL	CAA98373.1; MAJOR SECRETED IMMUNOGENIC PROTEIN	-0.280	0.082
GTSVLSWLSDHQVHN	MYCOBACTERIUM BOVIS MYCOCEROSIC ACID SYNTHASE GENE; M95808	-0.051	0.082
IGELDVIALYIASIA	PPE FAMILY PROTEIN CAE55334	-0.061	0.080
AGASRTDMEGTFARP	3-OXOACYL-[ACYL-CARRIER PROTEIN] SYNTHASE 2 KASB CAA94642	-0.193	0.080
QDLPLLVPLRAIPLL	PPE FAMILY PROTEIN CAE55504	-0.482	0.078
SYNTGSFNVGDTNTG	YP_177963; PPE FAMILY PROTEIN	-0.108	0.078
NQAISMIDGPAPDGY	PERIPLASMIC PHOSPHATE-BINDING LIPOPROTEIN PSTS1 YP_177770	-0.050	0.078
DTVIYSKYGGTEIKY	CAB01005.1; 10 KDA CHAPERONIN GROES	0.094	0.077
LPVEDMPFLADGTPV	RNA POLYMERASE BETA-SUBUNIT AAA21416	-0.161	0.076
HEGALLSERVATDLG	SECRETED L-ALANINE DEHYDROGENASE ALD CAA15575	-0.231	0.076
RMTTQDVEAITPQTL	RNA POLYMERASE BETA-SUBUNIT AAA21416	-0.001	0.075
YVITSVDEEMRHNEL	RNA POLYMERASE BETA'-SUBUNIT AAA21417	0.070	0.075
YSSKTINLCAPDDPI	PROBABLE CUTINASE PRECURSOR CFP21 NP_216500	-0.080	0.073
SGTHESNTMAMLARD	CAA16104.1; SECRETED ESAT-6 LIKE PROTEIN ESXR	-0.077	0.073
TDQEKAIKERYARCL	PROBABLE ISOCITRATE DEHYDROGENASE CAA16247	0.193	0.072
LGPVHIDQIDIPGMS	YP_177963; PPE FAMILY PROTEIN	-0.190	0.072
NTGGFNPGSINTGWF	YP_177963; PPE FAMILY PROTEIN	-0.036	0.072
GGPVAIQAITMPHHR	PUTATIVE CYCLOPROPANE-FATTY-ACYL-PHOSPHOLIPID SYNTHASE UFAA1 NP_854118	0.052	0.070
AMEDGYRVFADVSPH	MYCOBACTERIUM BOVIS MYCOCEROSIC ACID SYNTHASE GENE; M95808	-0.027	0.069
NTGGFNPGNVNTGWL	YP_177963; PPE FAMILY PROTEIN	0.143	0.069
KYIFPGGLLPSTQAI	PUTATIVE CYCLOPROPANE-FATTY-ACYL-PHOSPHOLIPID SYNTHASE UFAA1 NP_854118	0.056	0.068
EGWGKSPGFGTTVDF	PERIPLASMIC PHOSPHATE-BINDING LIPOPROTEIN PSTS1 YP_177770	-0.156	0.067
FAYLVDFGIARAASD	TRANSMEMBRANE SERINE/THREONINE-PROTEIN KINASE D PKND NP_215446	-0.112	0.066
MTEKGTFIINGTERV	RNA POLYMERASE BETA-SUBUNIT AAA21416	-0.240	0.065
LAEPGGSFNTAVASV	CONSERVED HYPOTHETICAL PROTEIN CAA15739	0.106	0.065
ATGNILRDYLTDLFP	PROBABLE ISOCITRATE DEHYDROGENASE CAA16247	-0.581	0.064
IEEVSADYITVMHDN	RNA POLYMERASE BETA-SUBUNIT AAA21416	-0.045	0.063
ADDEGYPKRARMLMD	CONSERVED HYPOTHETICAL PROTEIN TB16.3CAD97060	0.173	0.063
GFSFGYPIPLFPAVG	YP_177963; PPE FAMILY PROTEIN	-0.094	0.061
PYVLELADHGWRAAC	SECRETED L-ALANINE DEHYDROGENASE ALD CAA15575	0.137	0.061
SPNGTIRNILGGTIF	CAA17111.1; PROBABLE ISOCITRATE DEHYDROGENASE	-0.112	0.061
AMTTALATDAETTWK	3-OXOACYL-[ACYL-CARRIER PROTEIN] SYNTHASE 2 KASB CAA94642	-0.108	0.061
LAGGESRRMGRDKAT	CAA16030.1; PROBABLE MOLYBDOPTERIN-GUANINE DINUCLEOTIDE BIOSYNTHESIS PROTEIN	0.496	0.060
VVADKPEKEKASVPG	60 kDa chaperonin 2 P0A521	-0.209	0.059
LGLFPFRRGLTFYYL	MYCOBACTERIUM BOVIS MYCOCEROSIC ACID SYNTHASE GENE; M95808	-0.088	0.058
YIAIANAYWSPYAQR	POSSIBLE GLYCOSYL TRANSFERASE CAB05415	0.117	0.057
IGRHAFGDQYRATNF	CAA17111.1; PROBABLE ISOCITRATE DEHYDROGENASE	-0.171	0.056
FADVLIAFGRFPIID	MYCOBACTERIUM BOVIS MYCOCEROSIC ACID SYNTHASE GENE; M95808	-0.177	0.056
SSGLLRHAHCPVVII	HYPOTHETICAL PROTEIN RV2623 NP_217139	-0.148	0.054
VISRSEYRQAAARLA	POSSIBLE GLYCOSYL TRANSFERASE CAB05415	0.331	0.053
TEEAIRMNCRAVADH	CONSERVED HYPOTHETICAL PROTEIN CAB08634	-0.102	0.053
RTIDADRSFIEYGLD	MYCOBACTERIUM BOVIS MYCOCEROSIC ACID SYNTHASE GENE; M95808	-0.128	0.052
SGSFNNGALWTGDHH	YP_177963; PPE FAMILY PROTEIN	0.460	0.052
FHSPSDVIVDASMPA	PROBABLE ISOCITRATE DEHYDROGENASE CAA16247	0.152	0.052
GEAATLAHVVRPFVL	POSSIBLE GLYCOSYL TRANSFERASE CAB05418	-0.293	0.052
GDYTLYADVPELVPT	POSSIBLE GLYCOSYL TRANSFERASE CAB05418	-0.138	0.051
EQPTIIYTLTDEAPL	PROBABLE ISOCITRATE DEHYDROGENASE CAA16247	-0.139	0.050
DIKATTVFGGKYVSL	MCE-FAMILY PROTEIN MCE1AYP_177701	0.171	0.050

AMEDLVRAYHAMSST	CAA17363.1; LOW MOLECULAR WEIGHT PROTEIN ANTIGEN 7 ESXH	-0.131	0.049
QEQRAQGACTITVHP	MYCOBACTERIUM BOVIS MYCOCEROSIC ACID SYNTHASE GENE; M95808	0.019	0.049
PITRDLWPAPYLVMD	MCE-FAMILY PROTEIN MCE1AYP_177701	0.062	0.049
VASGNLPALPDMPGL	CONSERVED HYPOTHETICAL PROTEIN CAA15739	-0.149	0.048
WAAMGTQLLASEPVF	MYCOBACTERIUM BOVIS MYCOCEROSIC ACID SYNTHASE GENE; M95808	-0.059	0.047
FNVGDTNTGGFNPGS	YP_177963; PPE FAMILY PROTEIN	-0.150	0.046
VDVFAHWPRAQGLPV	MYCOBACTERIUM BOVIS MYCOCEROSIC ACID SYNTHASE GENE; M95808	-0.088	0.046
PDSDMTTAVMRPSKT	PROBABLE ISOCITRATE DEHYDROGENASE CAA16247	-0.117	0.045
LEVPLPPGVLRWQQD	HYPOTHETICAL PROTEIN RV2623 NP_217139	-0.213	0.044
ILPYLDIRLDYYDLG	CAA17111.1; PROBABLE ISOCITRATE DEHYDROGENASE	-0.167	0.044
FLALGAGFEDIGIKT	PROBABLE ISOCITRATE DEHYDROGENASE CAA16247	0.090	0.042
ALSKLTPWKAPPPIA	PPE FAMILY PROTEIN CAE55489	0.394	0.041
LTNVNSAAGLHAPMQ	CAA16030.1; PROBABLE MOLYBDOPTERIN-GUANINE DINUCLEOTIDE BIOSYNTHESIS PROTEIN	-0.199	0.041
NELVRVYVAQKRKIS	RNA POLYMERASE BETA-SUBUNIT AAA21416	0.296	0.040
HHDMVPLAQIYNNRA	PPE FAMILY PROTEIN CAE55489	0.156	0.039
YLDVYQWTLIREGPP	PUTATIVE CYCLOPROPANE-FATTY-ACYL-PHOSPHOLIPID SYNTHASE UFAA1 NP_854118	-0.131	0.039
VPPPVIAENRAELMI	CAE55371.1; PPE FAMILY PROTEIN	0.132	0.038
AAAAMAAAATPYVGW	PPE FAMILY PROTEIN CAE55334	-0.094	0.037
GDVGTSVLSWLSDHQ	MYCOBACTERIUM BOVIS MYCOCEROSIC ACID SYNTHASE GENE; M95808	0.127	0.036
EAVGYRSWPRYFAAL	PUTATIVE CYCLOPROPANE-FATTY-ACYL-PHOSPHOLIPID SYNTHASE UFAA1 NP_854118	0.079	0.036
SSLFKQISDKMGLAI	CAB06237.1; CONSERVED HYPOTHETICAL PROTEIN	-0.100	0.034
RVAVSSFGMSGTNVH	MYCOBACTERIUM BOVIS MYCOCEROSIC ACID SYNTHASE GENE; M95808	-0.086	0.034
AAALMAKLRGAAEKQ	TWO COMPONENT TRANSCRIPTIONAL REGULATORY PROTEIN DEVR NP_217649	0.008	0.033
GNTNTGGFNPGNVNT	YP_177963; PPE FAMILY PROTEIN	-0.078	0.033
TIRVGSFRGRWLDPR	CAA16102.1; ESAT-6 LIKE PROTEIN ESXQ	-0.121	0.032
TADGINYRVLWQAAG	CAB06500.1; IMMUNOGENIC PROTEIN MPT63	0.050	0.031
AKNTPGSITYNEWSF	PERIPLASMIC PHOSPHATE-BINDING LIPOPROTEIN PSTS3 YP_177768	-0.148	0.030
GSGVLNFGSGVSGLY	YP_177963; PPE FAMILY PROTEIN	0.010	0.030
FAVTNDGVIFFFNPG	CAA98382.1; IMMUNOGENIC PROTEIN MPT64	0.275	0.030
GAANIGDVNLGFGNI	YP_177963; PPE FAMILY PROTEIN	0.434	0.029
FTTGLANAGSYNTGS	YP_177963; PPE FAMILY PROTEIN	-0.056	0.028
TGIGNGGTGSTGLFN	YP_177963; PPE FAMILY PROTEIN	0.453	0.028
AGSSPLLAAAAAWDG	YP_177963; PPE FAMILY PROTEIN	0.019	0.028
TLQAFLHWAITDGNK	PERIPLASMIC PHOSPHATE-BINDING LIPOPROTEIN PSTS1 YP_177770	-0.299	0.027
AAPEIQLAVQATPTF	PPE FAMILY PROTEIN CAE55334	-0.113	0.027
EDRAWTEAVIGNRRR	MTB48 AAK31576	0.103	0.025
DVAQANLGEAAGTYV	CONSERVED HYPOTHETICAL PROTEIN TB9.8 CAD93159	-0.161	0.025
NTGAFISGNFSNGVL	YP_177963; PPE FAMILY PROTEIN	0.107	0.024
KADVYGNTRLGLFPF	MYCOBACTERIUM BOVIS MYCOCEROSIC ACID SYNTHASE GENE; M95808	0.083	0.024
LRGLLTELHRAGAAL	MYCOBACTERIUM BOVIS MYCOCEROSIC ACID SYNTHASE GENE; M95808	-0.031	0.024
AFQYDGVNDFPKYPL	PPE FAMILY PROTEIN CAE55504	0.047	0.023
KADLLVVGNVGLSTI	CAB08889.1; IRON-REGULATED CONSERVED HYPOTHETICAL PROTEIN	-0.026	0.022
MVSVATAMIPFLEHD	RNA POLYMERASE BETA-SUBUNIT AAA21416	-0.270	0.022
ASAWDGLAEELHAAA	PPE FAMILY PROTEIN CAE55504	-0.043	0.022
ALLLIETEEHAKARG	3-OXOACYL-[ACYL-CARRIER PROTEIN] SYNTHASE 2 KASB CAA94642	-0.064	0.020
AQRRFPLPDVIWTRL	POSSIBLE GLYCOSYL TRANSFERASE CAB05415	0.143	0.020
LESAITTIMDFEDSV	MTB81 (publication)	-0.097	0.019
LFDGRSGEPFPYPVT	RNA POLYMERASE BETA-SUBUNIT AAA21416	-0.173	0.017
AQLQAVPGAAQYIGL	LOW MOLECULAR WEIGHT T-CELL ANTIGEN TB8.4 NP_215690	-0.079	0.017
DDPFVEEFDLPVRIG	3-OXOACYL-[ACYL-CARRIER PROTEIN] SYNTHASE 2 KASB CAA94642	0.006	0.017
VDNNCQSILGYVVRW	MTB81 (publication)	0.098	0.017
RSLARVYGAGTPCAL	MYCOBACTERIUM BOVIS MYCOCEROSIC ACID SYNTHASE GENE; M95808	-0.122	0.016
RLPGGINSPDKLWES	MYCOBACTERIUM BOVIS MYCOCEROSIC ACID SYNTHASE GENE; M95808	-0.219	0.015
RSLRWSWLLSVLAAV	CAB09453.1; PROBABLE SERINE PROTEASE PEPA	-0.066	0.014
VYARVNPFGFIETPY	RNA POLYMERASE BETA-SUBUNIT AAA21416	0.271	0.013
KMYGADGKLKDTKAV	PROBABLE ISOCITRATE DEHYDROGENASE CAA16247	-0.090	0.013
DPRAPSLFIHRPDAL	PUTATIVE CYCLOPROPANE-FATTY-ACYL-PHOSPHOLIPID SYNTHASE UFAA1 NP_854118	-0.096	0.013
NSGVSMTNTLSSMLK	CAE55371.1; PPE FAMILY PROTEIN	-0.221	0.013
GNMGLGNIGFGNTGT	YP_177963; PPE FAMILY PROTEIN	0.625	0.012
TLTNITDELIDRDWS	MYCOBACTERIUM BOVIS MYCOCEROSIC ACID SYNTHASE GENE; M95808	-0.041	0.012
AGMGNIGLGNAGSGN	YP_177963; PPE FAMILY PROTEIN	0.124	0.011
HGPASLAMTRAASPY	PPE FAMILY PROTEIN CAE55504	-0.203	0.011

IAAAEAEYEQIWAQD	PPE FAMILY PROTEIN CAE55504	0.058	0.009
FRSGYLDVYQWTLIR	PUTATIVE CYCLOPROPANE-FATTY-ACYL-PHOSPHOLIPID SYNTHASE UFAA1 NP_854118	0.194	0.009
GGFLDDVAGFDAEFF	MYCOBACTERIUM BOVIS MYCOCEROSIC ACID SYNTHASE GENE; M95808	-0.006	0.008
LHAGPRKSALDEEEI	CONSERVED HYPOTHETICAL PROTEIN CAB08634	0.194	0.008
HLMDPAPQPSQLRPG	TRANSMEMBRANE SERINE/THREONINE-PROTEIN KINASE D PKND NP_215446	-0.179	0.008
MQAYGAAYTLQELLT	RNA POLYMERASE BETA-SUBUNIT AAA21416	-0.016	0.008
ATYEIVCSKYPDSQV	PERIPLASMIC PHOSPHATE-BINDING LIPOPROTEIN PSTS3 YP_177768	0.147	0.007
LAQIYNNRAERQILI	PPE FAMILY PROTEIN CAE55489	-0.104	0.005
VPIHDYGEINGQFFV	TRANSMEMBRANE SERINE/THREONINE-PROTEIN KINASE D PKND NP_215446	-0.123	0.003
GNANFGFGNSGLGLP	YP_177963; PPE FAMILY PROTEIN	-0.048	0.001
VVAVGPGRWDEDGEK	CAB01005.1; 10 KDA CHAPERONIN GROES	0.483	0.001
EVTYRADIYALACVL	TRANSMEMBRANE SERINE/THREONINE-PROTEIN KINASE D PKND NP_215446	-0.137	0.001
TGAFGPVVVPPIPVL	YP_177963; PPE FAMILY PROTEIN	-0.401	-0.001
LLGPLPFPHHPIHTV	POSSIBLE GLYCOSYL TRANSFERASE CAB05418	0.109	-0.001
AEELVFSYDDMRARG	3-OXOACYL-[ACYL-CARRIER PROTEIN] SYNTHASE 2 KASB CAA94642	-0.061	-0.002
NTARPWHIGLYGNAG	PPE FAMILY PROTEIN CAE55334	0.076	-0.002
VEYVPSSEVDYMDVS	RNA POLYMERASE BETA-SUBUNIT AAA21416	-0.041	-0.002
ANMGLGNIGFGNTGT	YP_177963; PPE FAMILY PROTEIN	0.298	-0.003
AYSGYIPILGAPWLA	MYCOBACTERIUM BOVIS MYCOCEROSIC ACID SYNTHASE GENE; M95808	-0.248	-0.004
DRVVFINTGFLDRTG	MTB81 (publication)	-0.083	-0.004
DAFETGVMFSLHVKA	PROBABLE ISOCITRATE DEHYDROGENASE CAA16247	0.151	-0.005
FSNGILWRGNYEGLA	YP_177963; PPE FAMILY PROTEIN	0.053	-0.005
GEPFPYPVTVGYMYI	RNA POLYMERASE BETA-SUBUNIT AAA21416	-0.064	-0.006
AMIPFLEHDDANRAL	RNA POLYMERASE BETA-SUBUNIT AAA21416	0.017	-0.007
SGAIGPTWYSVGRIY	YP_177963; PPE FAMILY PROTEIN	0.095	-0.007
ITDGNKASFLDQVHF	PERIPLASMIC PHOSPHATE-BINDING LIPOPROTEIN PSTS1 YP_177770	0.067	-0.007
RDYRLRVSDALSKLT	PPE FAMILY PROTEIN CAE55489	-0.298	-0.008
IRQAGVQYSRADEEQ	CAA17966.1; 10 KDA CULTURE FILTRATE ANTIGEN ESXB	0.156	-0.009
VMSHQNVRVNFEQLM	MYCOBACTERIUM BOVIS ACYL-COA SYNTHASE GENE; U75685	-0.075	-0.009
TRIEAVPIAGFAQMR	3-OXOACYL-[ACYL-CARRIER PROTEIN] SYNTHASE 2 KASB CAA94642	-0.188	-0.010
AAFDKLPAATIDQLK	CAA98350.1; CELL SURFACE LIPOPROTEIN MPT83	-0.047	-0.010
DNAVFRARMQREADT	TRANSMEMBRANE SERINE/THREONINE-PROTEIN KINASE D PKND NP_215446	-0.245	-0.010
NATVYMIDTVLMPPA	CAA98350.1; CELL SURFACE LIPOPROTEIN MPT83	-0.139	-0.010
ANVGDMAGYTGTTQS	CAA16102.1; ESAT-6 LIKE PROTEIN ESXQ	-0.098	-0.012
VGISQMHVSRLLAKS	ALTERNATE RNA POLYMERASE SIGMA FACTOR SIGF CAB07069	-0.366	-0.012
GWDPIPEGLTTGRSC	POSSIBLE HEMOLYSIN CAA16235	-0.014	-0.014
PLPFPHHPIHTVPSE	POSSIBLE GLYCOSYL TRANSFERASE CAB05418	-0.055	-0.016
LLLETSWEAIEHAGL	MYCOBACTERIUM BOVIS MYCOCEROSIC ACID SYNTHASE GENE; M95808	0.016	-0.017
DADADGFVRSEGCAM	MYCOBACTERIUM BOVIS MYCOCEROSIC ACID SYNTHASE GENE; M95808	-0.095	-0.017
ATSPAAPVTTAAMAD	CAA98350.1; CELL SURFACE LIPOPROTEIN MPT83	0.212	-0.017
WLSDHQVHNVAALPG	MYCOBACTERIUM BOVIS MYCOCEROSIC ACID SYNTHASE GENE; M95808	-0.213	-0.019
SADNMREYLAAGAKE	MTB48 AAK31576	-0.173	-0.020
LERLYAENPSARDQI	MTB48 AAK31576	-0.100	-0.020
VGDLGKLPTWTELAA	CAB06237.1; CONSERVED HYPOTHETICAL PROTEIN	-0.010	-0.020
AEFPDYLTEEQRVPD	PROBABLE ISOCITRATE DEHYDROGENASE CAA16247	0.112	-0.023
TGSFNVGDTNTGGFN	YP_177963; PPE FAMILY PROTEIN	-0.149	-0.023
RAYQSMSGTHESNTM	CAA16104.1; SECRETED ESAT-6 LIKE PROTEIN ESXR	-0.078	-0.024
VYDSRSVEFAEQIRR	MYCOBACTERIUM BOVIS MYCOCEROSIC ACID SYNTHASE GENE; M95808	-0.103	-0.024
VAASDLAYTLARGRR	MYCOBACTERIUM BOVIS MYCOCEROSIC ACID SYNTHASE GENE; M95808	-0.192	-0.024
NPIASIFAWTRGLQH	CAA17111.1; PROBABLE ISOCITRATE DEHYDROGENASE	-0.111	-0.026
KPPTWWDSLPTDRPI	POSSIBLE GLYCOSYL TRANSFERASE CAB05415	-0.144	-0.027
FTLPGERIHVVFRSD	PERIPLASMIC PHOSPHATE-BINDING LIPOPROTEIN PSTS3 YP_177768	0.183	-0.027
LETPSQFFPFTGLNS	PPE FAMILY PROTEIN CAE55504	0.030	-0.027
DTYSPELFCTIRNFY	MCE-FAMILY PROTEIN MCE1AYP_177701	-0.067	-0.027
TALATDAETTWKLLL	3-OXOACYL-[ACYL-CARRIER PROTEIN] SYNTHASE 2 KASB CAA94642	-0.061	-0.028
SKLMTRIAGAGAMGS	MYCOBACTERIUM BOVIS MYCOCEROSIC ACID SYNTHASE GENE; M95808	-0.085	-0.028
DMEGTFARPMTELVT	3-OXOACYL-[ACYL-CARRIER PROTEIN] SYNTHASE 2 KASB CAA94642	-0.110	-0.028
APEMTSAGGLLEQAA	CAE55371.1; PPE FAMILY PROTEIN	0.047	-0.029
ARSLDPSRYEVHFAC	POSSIBLE GLYCOSYL TRANSFERASE CAB05418	-0.101	-0.029
TMPHHRMLATRHTQT	PUTATIVE CYCLOPROPANE-FATTY-ACYL-PHOSPHOLIPID SYNTHASE UFAA1 NP_854118	-0.293	-0.029
DIWRMCIVKDAPIRD	PROBABLE ISOCITRATE DEHYDROGENASE CAA16247	0.098	-0.029

ITLFPAQNFNTTFPV	YP_177963; PPE FAMILY PROTEIN	-0.088	-0.030
FLDAIADNLEKELAN	CAA17111.1; PROBABLE ISOCITRATE DEHYDROGENASE	-0.226	-0.031
VRDLIARWEQRDVMA	MYCOBACTERIUM BOVIS MYCOCEROSIC ACID SYNTHASE GENE; M95808	-0.159	-0.031
YVAWMSVTAGQAELT	CAE55371.1; PPE FAMILY PROTEIN	0.357	-0.032
WTGDHHGLVGFSYSI	YP_177963; PPE FAMILY PROTEIN	0.018	-0.032
AFSRPGLPVEYLQVP	CAB10044.1; SECRETED ANTIGEN 85-B FBPB (85B)	-0.129	-0.034
TGTGNWGLFNSGSYN	YP_177963; PPE FAMILY PROTEIN	0.253	-0.034
GHNAVFNFPPNGTHS	CAB10044.1; SECRETED ANTIGEN 85-B FBPB (85B)	-0.080	-0.035
VGHGDRGPVWVFSGQ	MYCOBACTERIUM BOVIS MYCOCEROSIC ACID SYNTHASE GENE; M95808	0.094	-0.035
TGGFNTGNVNTGAFN	YP_177963; PPE FAMILY PROTEIN	-0.110	-0.036
KSGTSDNFQKYLDGA	PERIPLASMIC PHOSPHATE-BINDING LIPOPROTEIN PSTS2 YP_177769	0.546	-0.036
WWPAVAVGCAQPACL	MYCOBACTERIUM BOVIS MYCOCEROSIC ACID SYNTHASE GENE; M95808	-0.140	-0.036
ELMADMVETKIAQPR	MTB81 (publication)	-0.242	-0.036
NAGSGNFGFGNIGNA	YP_177963; PPE FAMILY PROTEIN	0.616	-0.037
VPPRPYVMPHSPAAG	CAE55371.1; PPE FAMILY PROTEIN	-0.103	-0.037
DTLVGAGERKMSALV	CAA16030.1; PROBABLE MOLYBDOPTERIN-GUANINE DINUCLEOTIDE BIOSYNTHESIS PROTEIN	-0.295	-0.039
AQGMSQDMAQTFTSI	CONSERVED HYPOTHETICAL PROTEIN CAA15739	0.170	-0.040
TTAKIFNGTITVWND	PERIPLASMIC PHOSPHATE-BINDING LIPOPROTEIN PSTS2 YP_177769	-0.061	-0.040
GNNSPAVYLLDGLRA	CAB10044.1; SECRETED ANTIGEN 85-B FBPB (85B)	-0.114	-0.041
VKDIKGMELARAVKD	TWO COMPONENT TRANSCRIPTIONAL REGULATORY PROTEIN DEVR NP_217649	-0.171	-0.041
FVADYLPGEAAAARS	POSSIBLE GLYCOSYL TRANSFERASE CAB05415	0.140	-0.042
LLGPLPFRHHAIHTI	POSSIBLE GLYCOSYL TRANSFERASE CAB05415	-0.028	-0.042
LDGDEMTRVIWKLIK	CAA17111.1; PROBABLE ISOCITRATE DEHYDROGENASE	-0.102	-0.043
VDTQGAVYVADRGNN	TRANSMEMBRANE SERINE/THREONINE-PROTEIN KINASE D PKND NP_215446	-0.183	-0.044
GNVGFGNIGDANFGF	YP_177963; PPE FAMILY PROTEIN	0.432	-0.044
FDAEFFGISEREATS	MYCOBACTERIUM BOVIS MYCOCEROSIC ACID SYNTHASE GENE; M95808	-0.063	-0.045
ANRTRLASLVAANLL	PPE FAMILY PROTEIN CAE55504	0.125	-0.046
LQTTDGSITYNEWSF	PERIPLASMIC PHOSPHATE-BINDING LIPOPROTEIN PSTS2 YP_177769	-0.128	-0.046
RYARCLGSAVNPVLR	PROBABLE ISOCITRATE DEHYDROGENASE CAA16247	-0.027	-0.047
AEAVTLAHVVRPFAL	POSSIBLE GLYCOSYL TRANSFERASE CAB05415	-0.205	-0.048
GNIAEPDTADRLVSA	MYCOBACTERIUM BOVIS MYCOCEROSIC ACID SYNTHASE GENE; M95808	-0.158	-0.049
PLSGLTHKRRLSALG	RNA POLYMERASE BETA-SUBUNIT AAA21416	-0.147	-0.050
IDSDRWPAVAKVPRG	PUTATIVE CYCLOPROPANE-FATTY-ACYL-PHOSPHOLIPID SYNTHASE UFAA1 NP_854118	0.012	-0.050
DTLNGGEYTVFAPTN	CAA98350.1; CELL SURFACE LIPOPROTEIN MPT83	-0.120	-0.050
RILAEFPDYLTEEQR	PROBABLE ISOCITRATE DEHYDROGENASE CAA16247	-0.044	-0.051
AAGDFWGGAGSVACQ	PUTATIVE ESAT-6 LIKE PROTEIN ESXN YP_177838	0.316	-0.051
TESTRRCWTLVSSRS	MYCOBACTERIUM BOVIS MYCOCEROSIC ACID SYNTHASE GENE; M95808	-0.096	-0.052
LLENLFFKEKRYDLA	RNA POLYMERASE BETA-SUBUNIT AAA21416	0.043	-0.052
NSWVDVFAHWPRAQG	MYCOBACTERIUM BOVIS MYCOCEROSIC ACID SYNTHASE GENE; M95808	-0.154	-0.052
RNVRMELLAKSGKTI	PROBABLE ISOCITRATE DEHYDROGENASE CAA16247	-0.140	-0.052
TGAGGSAPKHVKQLV	PROBABLE ISOCITRATE DEHYDROGENASE CAA16247	0.036	-0.053
RVYEEEFKAQFEAAG	CAA17111.1; PROBABLE ISOCITRATE DEHYDROGENASE	-0.006	-0.053
KSHGPQGTECASLSW	MYCOBACTERIUM BOVIS MYCOCEROSIC ACID SYNTHASE GENE; M95808	-0.009	-0.053
AAAQFNASPVAQSYL	LOW MOLECULAR WEIGHT T-CELL ANTIGEN TB8.4 NP_215690	-0.098	-0.053
EGVARVLTKPRFRGW	POSSIBLE HEMOLYSIN-LIKE PROTEIN CAA17201	-0.209	-0.055
DPDHDGMRVQVRRPG	MYCOBACTERIUM BOVIS MYCOCEROSIC ACID SYNTHASE GENE; M95808	-0.154	-0.056
DHQVHNVAALPGAAY	MYCOBACTERIUM BOVIS MYCOCEROSIC ACID SYNTHASE GENE; M95808	-0.064	-0.056
GAPNRVSFAKLREPL	RNA POLYMERASE BETA-SUBUNIT AAA21416	-0.213	-0.056
SYNTGSFNAGNTNTG	YP_177963; PPE FAMILY PROTEIN	-0.047	-0.056
PYEKIGAELVKEVAK	60 kDa chaperonin 2 P0A521	-0.152	-0.057
KSGKTIVLKPEVPLD	PROBABLE ISOCITRATE DEHYDROGENASE CAA16247	-0.118	-0.057
SFNTGIGNGGTGSTG	YP_177963; PPE FAMILY PROTEIN	0.437	-0.057
PASQRDEIIEDLHRC	PROBABLE ISOCITRATE DEHYDROGENASE CAA16247	-0.137	-0.058
PPPFRLPLLFVNALG	YP_177963; PPE FAMILY PROTEIN	-0.049	-0.059
DPRYNQLLGPLPFRH	POSSIBLE GLYCOSYL TRANSFERASE CAB05415	0.096	-0.060
IVPPTPGFGNATATP	YP_177963; PPE FAMILY PROTEIN	-0.241	-0.060
HEERRTAVLDPDHDG	MYCOBACTERIUM BOVIS MYCOCEROSIC ACID SYNTHASE GENE; M95808	-0.070	-0.062
EDEMKEGRYEVRAEL	CAA17245.1; HEAT SHOCK PROTEIN HSPX	0.166	-0.062
GLGLFFASKLAAAGC	MYCOBACTERIUM BOVIS MYCOCEROSIC ACID SYNTHASE GENE; M95808	0.014	-0.062
VTPKGETELTPEERL	RNA POLYMERASE BETA-SUBUNIT AAA21416	-0.096	-0.062
QAEEYGSHDKTFEIP	PROBABLE ISOCITRATE DEHYDROGENASE CAA16247	0.097	-0.062
LGDNGYIPIPDEFKS	PERIPLASMIC PHOSPHATE-BINDING LIPOPROTEIN PSTS3 YP_177768	0.180	-0.063

LRPTFDTRLMRLEDE	CAA17245.1; HEAT SHOCK PROTEIN HSPX	0.148	-0.064
FNTGLANAGSYNTGS	YP_177963; PPE FAMILY PROTEIN	-0.197	-0.064
GESRRMGRDKATLPL	CAA16030.1; PROBABLE MOLYBDOPTERIN-GUANINE DINUCLEOTIDE BIOSYNTHESIS PROTEIN	0.359	-0.065
LLDIYRKLRPGEPPT	RNA POLYMERASE BETA-SUBUNIT AAA21416	-0.511	-0.065
TDDQVTIDAAYAIKK	CAA17111.1; PROBABLE ISOCITRATE DEHYDROGENASE	-0.087	-0.065
TVLPFTGLNDPDGVA	TRANSMEMBRANE SERINE/THREONINE-PROTEIN KINASE D PKND NP_215446	-0.144	-0.065
RIGGHLLEEFDHQLT	3-OXOACYL-[ACYL-CARRIER PROTEIN] SYNTHASE 2 KASB CAA94642	-0.073	-0.065
TELTPEERLLRAIFG	RNA POLYMERASE BETA-SUBUNIT AAA21416	-0.543	-0.066
QVVLNALADLPVTVI	POSSIBLE GLYCOSYL TRANSFERASE CAB05418	-0.454	-0.066
GNDLVLDTDSFYRPK	PERIPLASMIC PHOSPHATE-BINDING LIPOPROTEIN PSTS3 YP_177768	-0.214	-0.067
IKYNGEEYLILSARD	CAB01005.1; 10 KDA CHAPERONIN GROES	-0.132	-0.068
ARSTGPYSMITQQPL	RNA POLYMERASE BETA-SUBUNIT AAA21416	-0.094	-0.068
LERPVGDSSDCGTIR	CAA16102.1; ESAT-6 LIKE PROTEIN ESXQ	-0.166	-0.070
SSGFFNTGGGGGSGI	YP_177963; PPE FAMILY PROTEIN	-0.107	-0.071
TTMIKTAVAVVAMAA	CAB06500.1; IMMUNOGENIC PROTEIN MPT63	-0.084	-0.071
TGNLGLFNSGTGNIG	YP_177963; PPE FAMILY PROTEIN	0.347	-0.072
LGKLPTWTELAALPD	CAB06237.1; CONSERVED HYPOTHETICAL PROTEIN	-0.148	-0.072
MTQTQIAERVGISQM	ALTERNATE RNA POLYMERASE SIGMA FACTOR SIGF CAB07069	-0.183	-0.072
RLHTLLAVLTAAPLL	PERIPLASMIC PHOSPHATE-BINDING LIPOPROTEIN PSTS1 YP_177770	0.002	-0.074
GGQGFAIPIGQAMAI	CAB09453.1; PROBABLE SERINE PROTEASE PEPA	0.133	-0.074
QAASNGAWGKGAGKS	PERIPLASMIC PHOSPHATE-BINDING LIPOPROTEIN PSTS3 YP_177768	-0.179	-0.075
RFPIIDDREPQLGMD	MYCOBACTERIUM BOVIS MYCOCEROSIC ACID SYNTHASE GENE; M95808	-0.257	-0.075
PLDDGDVIDSMFMSK	PROBABLE ISOCITRATE DEHYDROGENASE CAA16247	-0.120	-0.075
LYLAYSEAGFRSGYL	PUTATIVE CYCLOPROPANE-FATTY-ACYL-PHOSPHOLIPID SYNTHASE UFAA1 NP_854118	0.026	-0.076
FQVPGSIDAITLFPG	YP_177963; PPE FAMILY PROTEIN	-0.353	-0.076
TIFREPIVISNVPRL	CAA17111.1; PROBABLE ISOCITRATE DEHYDROGENASE	-0.093	-0.077
TPVDIILNTHGVPRR	RNA POLYMERASE BETA-SUBUNIT AAA21416	-0.012	-0.078
TGIANSGNVNTGAFI	YP_177963; PPE FAMILY PROTEIN	-0.137	-0.078
TLLDTYSPELFCTIR	MCE-FAMILY PROTEIN MCE1AYP_177701	-0.255	-0.078
RVQAATIKRFADRFA	MYCOBACTERIUM BOVIS ACYL-COA SYNTHASE GENE; U75685	-0.037	-0.079
YQQRSEKVLTEYNNK	MTB48 AAK31576	-0.072	-0.079
MTLLELSDFVKKFEE	CAB07109.1; PROBABLE 50S RIBOSOMAL PROTEIN L7/L12 RPLL	0.079	-0.080
EMRYLQSLPAHLRPG	PPE FAMILY PROTEIN CAE55504	-0.129	-0.081
GLELLPFGGRFVEIG	MYCOBACTERIUM BOVIS MYCOCEROSIC ACID SYNTHASE GENE; M95808	-0.070	-0.082
GVNVNNGLSDLYSKI	PROBABLE ISOCITRATE DEHYDROGENASE CAA16247	0.215	-0.082
LPGVSEHLKLNGKVL	PERIPLASMIC PHOSPHATE-BINDING LIPOPROTEIN PSTS1 YP_177770	-0.278	-0.082
PGVLRWQQDHGRHLI	HYPOTHETICAL PROTEIN RV2623 NP_217139	0.238	-0.083
ERDRLVSERLLTLGW	MYCOBACTERIUM BOVIS MYCOCEROSIC ACID SYNTHASE GENE; M95808	-0.202	-0.084
MTRIAGAGAMGSVEL	MYCOBACTERIUM BOVIS MYCOCEROSIC ACID SYNTHASE GENE; M95808	-0.270	-0.084
SAKMLSVVPLMAGGG	PROBABLE ISOCITRATE DEHYDROGENASE CAA16247	-0.376	-0.084
RDQARRNIAVHYDLS	PUTATIVE CYCLOPROPANE-FATTY-ACYL-PHOSPHOLIPID SYNTHASE UFAA1 NP_854118	0.180	-0.084
LFPDVDWAEVAADLQ	PPE FAMILY PROTEIN CAE55504	-0.159	-0.084
PAMTFDTACSSGLMA	MYCOBACTERIUM BOVIS MYCOCEROSIC ACID SYNTHASE GENE; M95808	-0.069	-0.085
PTQGTTPSSKLGGLW	CAE55371.1; PPE FAMILY PROTEIN	-0.172	-0.086
ANIYAGPGPDSMLAA	PPE FAMILY PROTEIN CAE55489	-0.158	-0.086
IEPIDMDAYRQFLTE	MTB81 (publication)	-0.119	-0.088
AGNLGLFNSGTGNIG	YP_177963; PPE FAMILY PROTEIN	0.318	-0.088
TKPVSQDTSPKPATS	CAA98350.1; CELL SURFACE LIPOPROTEIN MPT83	-0.200	-0.088
TEEDVVATIEYLVRL	RNA POLYMERASE BETA-SUBUNIT AAA21416	-0.078	-0.089
PIFGFGGAPGFGNDT	YP_177963; PPE FAMILY PROTEIN	-0.141	-0.089
RPSYGLAEATVYVAT	MYCOBACTERIUM BOVIS ACYL-COA SYNTHASE GENE; U75685	-0.203	-0.090
YELNITSATYQSAIP	CAA98382.1; IMMUNOGENIC PROTEIN MPT64	-0.102	-0.091
FVTDGKMFIIGRIKD	MYCOBACTERIUM BOVIS ACYL-COA SYNTHASE GENE; U75685	-0.037	-0.091
ACLRAYPQCPLLLHA	MYCOBACTERIUM BOVIS MYCOCEROSIC ACID SYNTHASE GENE; M95808	-0.265	-0.091
YSPPPQQPGTPGYAQ	CAA17582.1; PROBABLE SERINE PROTEASE PEPD	-0.215	-0.092
MGQGNDLVLDTSSFY	PERIPLASMIC PHOSPHATE-BINDING LIPOPROTEIN PSTS2 YP_177769	-0.151	-0.092
LAFVCAVDMPYLTVE	CAA16030.1; PROBABLE MOLYBDOPTERIN-GUANINE DINUCLEOTIDE BIOSYNTHESIS PROTEIN	-0.275	-0.094
EDVEGEALSTLVVNK	60 kDa chaperonin 2 P0A521	-0.155	-0.094
WDSLGEFLALGAGFE	PROBABLE ISOCITRATE DEHYDROGENASE CAA16247	0.085	-0.094
ALPGLPSIPNLFPGL	CAB06237.1; CONSERVED HYPOTHETICAL PROTEIN	-0.359	-0.094
GARDDLMVNNAGLVC	CAA98350.1; CELL SURFACE LIPOPROTEIN MPT83	-0.196	-0.095
RSTNFRWGGAFDLDR	POSSIBLE GLYCOSYL TRANSFERASE CAB05419	0.140	-0.096

SLTGAAQRAGLELLP	MYCOBACTERIUM BOVIS MYCOCEROSIC ACID SYNTHASE GENE; M95808	-0.058	-0.097
VETTSVFRADFLSEL	CAB01474.1; CONSERVED HYPOTHETICAL PROTEIN CFP17	0.282	-0.097
NTGFLDRTGDEIHTS	MTB81 (publication)	0.194	-0.097
PSPPPQPTQYRQPYE	CAA17582.1; PROBABLE SERINE PROTEASE PEPD	-0.116	-0.097
VGAQNLGAANAGSGN	YP_177963; PPE FAMILY PROTEIN	0.334	-0.098
GLLDVQTDSFEWLIG	RNA POLYMERASE BETA-SUBUNIT AAA21416	-0.078	-0.098
ADPVDAVINTTCNYG	LOW MOLECULAR WEIGHT T-CELL ANTIGEN TB8.4 NP_215690	-0.119	-0.099
YYAPDSDMTTAVMRP	PROBABLE ISOCITRATE DEHYDROGENASE CAA16247	-0.206	-0.099
LMAKLRGAAEKQDPL	TWO COMPONENT TRANSCRIPTIONAL REGULATORY PROTEIN DEVR NP_217649	-0.049	-0.099
IKTAVAVVAMAAIAT	CAB06500.1; IMMUNOGENIC PROTEIN MPT63	-0.028	-0.101
APKPLLEKVAKEAAD	CAB07109.1; PROBABLE 50S RIBOSOMAL PROTEIN L7/L12 RPLL	-0.842	-0.101
TLYADVPELVPTYNL	POSSIBLE GLYCOSYL TRANSFERASE CAB05418	-0.182	-0.101
CPMVVAPGGTFESIT	CAB10947.1; PROBABLE LIPOPROTEIN LPRJ	-0.306	-0.102
VTMPVPEPMVKMLLN	CONSERVED HYPOTHETICAL PROTEIN TB18.5CAD93033	-0.268	-0.102
MAIAGQIRSGGGSPT	CAB09453.1; PROBABLE SERINE PROTEASE PEPA	-0.142	-0.102
FGNTTTAPSSGFFNS	YP_177963; PPE FAMILY PROTEIN	0.080	-0.103
NHLKNVPANAFVADY	POSSIBLE GLYCOSYL TRANSFERASE CAB05418	-0.231	-0.103
LVDFGIARAASDPGL	TRANSMEMBRANE SERINE/THREONINE-PROTEIN KINASE D PKND NP_215446	-0.310	-0.104
GLFNSGSFNTGIGNG	YP_177963; PPE FAMILY PROTEIN	0.177	-0.104
AAERGPGQMLGGLPV	CAE55371.1; PPE FAMILY PROTEIN	-0.185	-0.105
GVGNIGFANTGSNNI	YP_177963; PPE FAMILY PROTEIN	0.376	-0.105
VGITIPEFPIHWTST	YP_177963; PPE FAMILY PROTEIN	-0.204	-0.105
GLAGFSFGYPIPLFP	YP_177963; PPE FAMILY PROTEIN	-0.094	-0.106
ASGLVIPDTAKEKPQ	CAB01005.1; 10 KDA CHAPERONIN GROES	-0.173	-0.106
VLDDLNSRMPQSRHD	MCE-FAMILY PROTEIN MCE1AYP_177701	-0.172	-0.106
YLPQHEDARAADILK	CAB08889.1; IRON-REGULATED CONSERVED HYPOTHETICAL PROTEIN	0.067	-0.107
IQTVMQQGELFAKQE	CONSERVED HYPOTHETICAL PROTEIN TB18.5CAD93033	0.067	-0.107
EAMLDAILAGASGYV	TWO COMPONENT TRANSCRIPTIONAL REGULATORY PROTEIN DEVR NP_217649	0.045	-0.108
AMTANVGDMAGYTGT	CAA16102.1; ESAT-6 LIKE PROTEIN ESXQ	-0.133	-0.108
HSLLAGREQVRHLVR	MYCOBACTERIUM BOVIS MYCOCEROSIC ACID SYNTHASE GENE; M95808	0.023	-0.108
GINLSRNESASFEDL	RNA POLYMERASE BETA-SUBUNIT AAA21416	-0.066	-0.109
EVPGLLDVQTDSFEW	RNA POLYMERASE BETA-SUBUNIT AAA21416	-0.091	-0.109
FLGGFAGLPSLGFGN	CAB06237.1; CONSERVED HYPOTHETICAL PROTEIN	0.136	-0.110
IPIPGLPDIPGLPDF	CAB06237.1; CONSERVED HYPOTHETICAL PROTEIN	-0.322	-0.111
PLAGVVLAGGESRRM	CAA16030.1; PROBABLE MOLYBDOPTERIN-GUANINE DINUCLEOTIDE BIOSYNTHESIS PROTEIN	0.070	-0.111
QGELFAKQEQLFSVV	CONSERVED HYPOTHETICAL PROTEIN TB18.5CAD93033	-0.142	-0.112
NTMAMMARDTAEAAK	CAA17363.1; LOW MOLECULAR WEIGHT PROTEIN ANTIGEN 7 ESXH	-0.016	-0.112
SQQFDWRYPPSPPPQ	CAA17582.1; PROBABLE SERINE PROTEASE PEPD	0.001	-0.113
RGAWLEFDVDKRDTV	RNA POLYMERASE BETA-SUBUNIT AAA21416	-0.057	-0.113
YAINNSFGFGGHNVA	3-OXOACYL-[ACYL-CARRIER PROTEIN] SYNTHASE 2 KASB CAA94642	-0.030	-0.114
HRGGWDPIPEGLTTG	POSSIBLE HEMOLYSIN CAA16235	-0.137	-0.115
ILDVDPRYIHLIPAN	MCE-FAMILY PROTEIN MCE1AYP_177701	0.079	-0.115
DIPALPGFGNSTELP	YP_177963; PPE FAMILY PROTEIN	-0.141	-0.115
TSEGMYGRVVKLATG	TRANSMEMBRANE SERINE/THREONINE-PROTEIN KINASE D PKND NP_215446	-0.091	-0.116
ADVPELVPTYNLPAN	POSSIBLE GLYCOSYL TRANSFERASE CAB05418	-0.110	-0.117
GDLIAEAMDKVGNEG	60 kDa chaperonin 2 P0A521	0.567	-0.117
GPVWVFSGQGSQWAA	MYCOBACTERIUM BOVIS MYCOCEROSIC ACID SYNTHASE GENE; M95808	0.096	-0.118
ISNFHSPSDVIVDAS	PROBABLE ISOCITRATE DEHYDROGENASE CAA16247	-0.055	-0.118
QVTIDAAYAIKKHGV	CAA17111.1; PROBABLE ISOCITRATE DEHYDROGENASE	-0.073	-0.118
VAVRWAARDAELRKI	HYPOTHETICAL PROTEIN RV2623 NP_217139	-0.108	-0.119
DGEAGSWLLIDTSNA	MYCOBACTERIUM BOVIS MYCOCEROSIC ACID SYNTHASE GENE; M95808	-0.115	-0.119
RFVEPRVLVRRKAGE	RNA POLYMERASE BETA-SUBUNIT AAA21416	-0.136	-0.120
PELVPTYNLPANHRY	POSSIBLE GLYCOSYL TRANSFERASE CAB05418	-0.013	-0.120
ATGVDMANPMSPVNK	LIPOPROTEIN LPQH PRECURSOR P0A5J0	-0.098	-0.121
GIAVDEAGTVYVTEH	TRANSMEMBRANE SERINE/THREONINE-PROTEIN KINASE D PKND NP_215446	-0.183	-0.121
PEHAIVQFVKAICSP	HYPOTHETICAL PROTEIN RV2626C NP_217142	-0.286	-0.121
QARRRFPLPDVPWTR	POSSIBLE GLYCOSYL TRANSFERASE CAB05418	-0.142	-0.122
EIIEDLHRCHEHRPE	PROBABLE ISOCITRATE DEHYDROGENASE CAA16247	-0.029	-0.122
SSGFFNTGGGGGSGF	YP_177963; PPE FAMILY PROTEIN	-0.006	-0.122
DPITIFPGGFTIDPL	YP_177963; PPE FAMILY PROTEIN	0.050	-0.123
EIGKADVYGNTRLGL	MYCOBACTERIUM BOVIS MYCOCEROSIC ACID SYNTHASE GENE; M95808	0.027	-0.123

HACTAVAAICHYIAM	POSSIBLE HEMOLYSIN-LIKE PROTEIN CAA17201	-0.214	-0.124
TDEAPLLATYAFLPI	PROBABLE ISOCITRATE DEHYDROGENASE CAA16247	-0.134	-0.125
LRQTARQLATWVEEH	MYCOBACTERIUM BOVIS MYCOCEROSIC ACID SYNTHASE GENE; M95808	-0.085	-0.125
MLEGCILADSRQSKT	RNA POLYMERASE BETA-SUBUNIT AAA21416	-0.146	-0.126
PYIAIANAYWSPQAR	POSSIBLE GLYCOSYL TRANSFERASE CAB05418	0.028	-0.126
MEAGPMVRKGTMKSQ	MTB81 (publication)	-0.162	-0.127
GKILPVEDMPFLADG	RNA POLYMERASE BETA-SUBUNIT AAA21416	-0.154	-0.127
VGLMAQQAEEYGSHD	PROBABLE ISOCITRATE DEHYDROGENASE CAA16247	0.278	-0.128
GFAGLPSLGFGNLLS	CAB06237.1; CONSERVED HYPOTHETICAL PROTEIN	0.061	-0.128
MAPNFDDSIAFLAAQ	MTB81 (publication)	-0.142	-0.128
PAKMLRLNAGLGNVG	YP_177963; PPE FAMILY PROTEIN	0.291	-0.128
QTGVYEDLLAAGVAD	60 kDa chaperonin 2 P0A521	-0.126	-0.128
WGGGSLPTIGPLYSS	PROBABLE CUTINASE PRECURSOR CFP21 NP_216500	-0.075	-0.128
YDGVNDFPKYPLNVF	PPE FAMILY PROTEIN CAE55504	-0.142	-0.128
LNLGAANIGDVNLGF	YP_177963; PPE FAMILY PROTEIN	-0.112	-0.128
SRMERVVRERMTTQD	RNA POLYMERASE BETA-SUBUNIT AAA21416	-0.243	-0.128
SGNFGFGNIGNANFG	YP_177963; PPE FAMILY PROTEIN	0.327	-0.129
AGVLLRTERLKSQRV	POSSIBLE GLYCOSYL TRANSFERASE CAB05415	-0.242	-0.130
ISDEVLADLDERGIV	RNA POLYMERASE BETA-SUBUNIT AAA21416	-0.222	-0.130
ALYPAGRLVDAPLPA	MYCOBACTERIUM BOVIS MYCOCEROSIC ACID SYNTHASE GENE; M95808	-0.151	-0.130
FKARAAEKPAPSDRA	MTB81 (publication)	-0.067	-0.130
SGNVGFGNMGVGNIG	YP_177963; PPE FAMILY PROTEIN	0.322	-0.131
AGVGNIGLGNAGSGN	YP_177963; PPE FAMILY PROTEIN	0.115	-0.131
AGDKKIGVIKVVREI	CAB07109.1; PROBABLE 50S RIBOSOMAL PROTEIN L7/L12 RPLL	-0.288	-0.131
DPERQEAVLEDPYIL	60 kDa chaperonin 2 P0A521	-0.145	-0.132
TDDIDHFGNRRLRTV	RNA POLYMERASE BETA-SUBUNIT AAA21416	0.192	-0.132
HARFVAAAAKVNTLL	CONSERVED HYPOTHETICAL PROTEIN TB9.8 CAD93159	-0.095	-0.132
VSEHLKLNGKVLAAM	PERIPLASMIC PHOSPHATE-BINDING LIPOPROTEIN PSTS1 YP_177770	-0.151	-0.132
DVQTDSFEWLIGSPR	RNA POLYMERASE BETA-SUBUNIT AAA21416	-0.119	-0.132
LYAENPSARDQILPV	MTB48 AAK31576	-0.218	-0.133
QQRLLLETSWEAIEH	MYCOBACTERIUM BOVIS MYCOCEROSIC ACID SYNTHASE GENE; M95808	-0.092	-0.133
RDRDGFVFGEGGALL	3-OXOACYL-[ACYL-CARRIER PROTEIN] SYNTHASE 2 KASB CAA94642	0.156	-0.134
AAVMYSYEGASAAAS	PPE FAMILY PROTEIN CAE55334	-0.088	-0.135
VYLTADEEDRHVVAQ	RNA POLYMERASE BETA-SUBUNIT AAA21416	-0.130	-0.136
TGDEVTYRADIYALA	TRANSMEMBRANE SERINE/THREONINE-PROTEIN KINASE D PKND NP_215446	-0.088	-0.136
PLLLAAAGCGSKPPS	PERIPLASMIC PHOSPHATE-BINDING LIPOPROTEIN PSTS1 YP_177770	-0.162	-0.136
AEIGRGSGLAAQGLA	CONSERVED HYPOTHETICAL PROTEIN CAB08634	-0.047	-0.136
FFGVRPVSILYRLYR	POSSIBLE GLYCOSYL TRANSFERASE CAB05418	-0.112	-0.136
GLWPPKLPDIPIPGL	CAB06237.1; CONSERVED HYPOTHETICAL PROTEIN	-0.097	-0.137
LAALGDVYADAAPDL	MCE-FAMILY PROTEIN MCE1AYP_177701	-0.198	-0.137
NVDLVALHAWSDVDV	HYPOTHETICAL PROTEIN RV2623 NP_217139	-0.136	-0.137
VPGWTKPIVIGRHAF	CAA17111.1; PROBABLE ISOCITRATE DEHYDROGENASE	-0.086	-0.137
GLGNIGNANVGFGNI	YP_177963; PPE FAMILY PROTEIN	0.375	-0.138
KDGLFCEKIFGPTRD	RNA POLYMERASE BETA'-SUBUNIT AAA21417	-0.192	-0.138
SLSVYARVNPFGFIE	RNA POLYMERASE BETA-SUBUNIT AAA21416	-0.030	-0.139
VDGAPKPLLEKVAKE	CAB07109.1; PROBABLE 50S RIBOSOMAL PROTEIN L7/L12 RPLL	-0.150	-0.139
AHVVRPFVLARSLDP	POSSIBLE GLYCOSYL TRANSFERASE CAB05418	-0.203	-0.139
MTTPGKLNKARVPPY	MCE-FAMILY PROTEIN MCE1AYP_177701	0.113	-0.139
SRTDMEGTFARPMTE	3-OXOACYL-[ACYL-CARRIER PROTEIN] SYNTHASE 2 KASB CAA94642	-0.130	-0.140
KVYQNAGGTHPTTTY	CAA98382.1; IMMUNOGENIC PROTEIN MPT64	-0.116	-0.140
LFDIHELVNIGPFRI	YP_177963; PPE FAMILY PROTEIN	-0.122	-0.141
NSTELPSSGFFNTGG	YP_177963; PPE FAMILY PROTEIN	-0.089	-0.141
SYIITGGLGGLGLFF	MYCOBACTERIUM BOVIS MYCOCEROSIC ACID SYNTHASE GENE; M95808	-0.100	-0.141
AQGRLFYNTRTLRKY	POSSIBLE GLYCOSYL TRANSFERASE CAB05418	-0.107	-0.141
PEGPWLRTGDSGFVT	MYCOBACTERIUM BOVIS ACYL-COA SYNTHASE GENE; U75685	-0.154	-0.141
PAPDGYPIINYEYAI	PERIPLASMIC PHOSPHATE-BINDING LIPOPROTEIN PSTS1 YP_177770	-0.221	-0.142
AHAGDMAGYAGTLQS	CAA16104.1; SECRETED ESAT-6 LIKE PROTEIN ESXR	-0.105	-0.142
RTGDSGFVTDGKMFI	MYCOBACTERIUM BOVIS ACYL-COA SYNTHASE GENE; U75685	-0.058	-0.144
AQAASLEAEHQAIVR	PUTATIVE ESAT-6 LIKE PROTEIN ESXN YP_177838	-0.022	-0.144
WEATATVNAIRGSVT	CAB06500.1; IMMUNOGENIC PROTEIN MPT63	0.006	-0.144
ELFDDLGVNVNNGLS	PROBABLE ISOCITRATE DEHYDROGENASE CAA16247	-0.065	-0.145
FLTTEAVVADKPEKE	60 kDa chaperonin 2 P0A521	-0.098	-0.145
LTPKVIATNKTARAL	MYCOBACTERIUM BOVIS MYCOCEROSIC ACID SYNTHASE GENE; M95808	-0.185	-0.146
DNLTGIGGLNSGTGN	YP_177963; PPE FAMILY PROTEIN	0.077	-0.147
CCSGVATAAPKTYCE	CAA98382.1; IMMUNOGENIC PROTEIN MPT64	0.222	-0.148

LQRGVADLVPTATLL	MCE-FAMILY PROTEIN MCE1AYP_177701	-0.099	-0.149
AFVADYLPGEAAAAR	POSSIBLE GLYCOSYL TRANSFERASE CAB05418	-0.189	-0.150
QKPDESERTFGGKIV	MYCOBACTERIUM BOVIS ACYL-COA SYNTHASE GENE; U75685	-0.018	-0.150
ADVSPHPLLTHAVEQ	MYCOBACTERIUM BOVIS MYCOCEROSIC ACID SYNTHASE GENE; M95808	-0.403	-0.150
DNQSGIGGLNSGTGN	YP_177963; PPE FAMILY PROTEIN	0.146	-0.151
IAADRKILNEIAPDV	POSSIBLE GLYCOSYL TRANSFERASE CAB05418	-0.379	-0.151
RHAKAGVMTPVSACA	3-OXOACYL-[ACYL-CARRIER PROTEIN] SYNTHASE 2 KASB CAA94642	-0.057	-0.152
TYVAADAAAASTYTG	CONSERVED HYPOTHETICAL PROTEIN TB9.8 CAD93159	0.134	-0.152
GIPYIAIANAYWSPY	POSSIBLE GLYCOSYL TRANSFERASE CAB05415	0.082	-0.152
NALFAGPAKMLRLNA	YP_177963; PPE FAMILY PROTEIN	-0.211	-0.152
IYFDVTGPSPTIVAM	CAB06500.1; IMMUNOGENIC PROTEIN MPT63	-0.139	-0.153
TRIDPIPLNFDVLSS	YP_177963; PPE FAMILY PROTEIN	-0.402	-0.153
RDATDDQVTIDAAYA	CAA17111.1; PROBABLE ISOCITRATE DEHYDROGENASE	-0.069	-0.154
NFEQLMSGYFADTDG	MYCOBACTERIUM BOVIS ACYL-COA SYNTHASE GENE; U75685	0.063	-0.155
QFDPTTMGTVPNVGL	PROBABLE ISOCITRATE DEHYDROGENASE CAA16247	0.012	-0.156
SFVRTVSLPVGADED	CAA17245.1; HEAT SHOCK PROTEIN HSPX	0.310	-0.156
GNWGLFNSGSYNTGI	YP_177963; PPE FAMILY PROTEIN	0.213	-0.157
MKLTTMIKTAVAVVA	CAB06500.1; IMMUNOGENIC PROTEIN MPT63	-0.133	-0.157
SSLLTSILTYHVVAG	CAA98373.1; MAJOR SECRETED IMMUNOGENIC PROTEIN	-0.364	-0.158
TSALSGKLNPDVNLV	CAA98350.1; CELL SURFACE LIPOPROTEIN MPT83	0.034	-0.158
SSTHEANTMAMMARD	CAA17363.1; LOW MOLECULAR WEIGHT PROTEIN ANTIGEN 7 ESXH	-0.172	-0.158
VAQQFEAEIGRGSGL	CONSERVED HYPOTHETICAL PROTEIN CAB08634	0.159	-0.158
LRISSQLLANWLRHG	MTB81 (publication)	-0.062	-0.159
QLAEHFASLADVLTK	PROBABLE ISOCITRATE DEHYDROGENASE CAA16247	-0.179	-0.160
AFLHWAITDGNKASF	PERIPLASMIC PHOSPHATE-BINDING LIPOPROTEIN PSTS1 YP_177770	0.010	-0.160
VDPRYIHLIPANVNA	MCE-FAMILY PROTEIN MCE1AYP_177701	-0.102	-0.160
GLEEVLYELSPIEDF	RNA POLYMERASE BETA-SUBUNIT AAA21416	-0.190	-0.160
DAWHGPASLAMTRAA	PPE FAMILY PROTEIN CAE55504	-0.149	-0.161
WGPSSDPAWERNDPT	CAB10044.1; SECRETED ANTIGEN 85-B FBPB (85B)	-0.153	-0.163
FVLARSLDPSRYEVH	POSSIBLE GLYCOSYL TRANSFERASE CAB05418	-0.012	-0.163
ARMLMDAAIFKDTLI	CONSERVED HYPOTHETICAL PROTEIN TB16.3CAD97060	-0.145	-0.163
GAANAGSGNFGFGNI	YP_177963; PPE FAMILY PROTEIN	0.618	-0.164
PAFTLFPGGLNIPQN	YP_177963; PPE FAMILY PROTEIN	-0.107	-0.165
GYAVPDYPADPKTDQ	PROBABLE ISOCITRATE DEHYDROGENASE CAA16247	-0.107	-0.165
TLTWSQLYRRTLNVA	MYCOBACTERIUM BOVIS ACYL-COA SYNTHASE GENE; U75685	-0.160	-0.166
STFSRIYQEIINFCK	PROBABLE ISOCITRATE DEHYDROGENASE CAA16247	0.154	-0.166
AYAFETLVRHDRAYS	MYCOBACTERIUM BOVIS MYCOCEROSIC ACID SYNTHASE GENE; M95808	0.094	-0.167
DTDSFYRPKRPGSYP	PERIPLASMIC PHOSPHATE-BINDING LIPOPROTEIN PSTS3 YP_177768	-0.067	-0.167
GKNLLVAIMPWEGHN	RNA POLYMERASE BETA-SUBUNIT AAA21416	-0.242	-0.167
GGSTIPAIPIGLELN	YP_177963; PPE FAMILY PROTEIN	-0.162	-0.168
TLAETGSTLLYPLFN	PERIPLASMIC PHOSPHATE-BINDING LIPOPROTEIN PSTS1 YP_177770	0.054	-0.168
EIAPDVVVGDNRLSL	POSSIBLE GLYCOSYL TRANSFERASE CAB05418	-0.159	-0.168
KDTKAVNPESTFSRI	PROBABLE ISOCITRATE DEHYDROGENASE CAA16247	-0.010	-0.168
SRGPSKFRMELLSLP	MYCOBACTERIUM BOVIS MYCOCEROSIC ACID SYNTHASE GENE; M95808	-0.105	-0.169
FPFRRGLTFYYLDLA	MYCOBACTERIUM BOVIS MYCOCEROSIC ACID SYNTHASE GENE; M95808	-0.135	-0.169
PAGRLVDAPLPAWTH	MYCOBACTERIUM BOVIS MYCOCEROSIC ACID SYNTHASE GENE; M95808	-0.088	-0.170
RDTDQGVYDAMNRGV	POSSIBLE GLYCOSYL TRANSFERASE CAB05419	-0.089	-0.172
SPELFCTIRNFYDAD	MCE-FAMILY PROTEIN MCE1AYP_177701	-0.135	-0.172
CWAMQAYGAAYTLQE	RNA POLYMERASE BETA-SUBUNIT AAA21416	-0.138	-0.172
QHLNMEAVERAGAGV	POSSIBLE GLYCOSYL TRANSFERASE CAB05415	-0.203	-0.173
SSASELATAIAFDEA	HYPOTHETICAL PROTEIN RV2623 NP_217139	-0.080	-0.173
RLVGIVTEADIARHL	HYPOTHETICAL PROTEIN RV2626C NP_217142	-0.195	-0.173
QAAVVRFQEAANKQK	CAA17966.1; 10 KDA CULTURE FILTRATE ANTIGEN ESXB	-0.115	-0.173
YVAQKRKISDGDKLA	RNA POLYMERASE BETA-SUBUNIT AAA21416	0.064	-0.174
GDFNLGAANLGDLNL	YP_177963; PPE FAMILY PROTEIN	0.031	-0.174
AATATATATLLPFEE	CAE55371.1; PPE FAMILY PROTEIN	-0.206	-0.174
RCPRRSGSSRAPTES	MYCOBACTERIUM BOVIS MYCOCEROSIC ACID SYNTHASE GENE; M95808	0.160	-0.175
GFNPGSINTGWFNTG	YP_177963; PPE FAMILY PROTEIN	-0.182	-0.175
VYEDLLAAGVADPVK	60 kDa chaperonin 2 P0A521	0.115	-0.176
MPMGAAHQGQGGAKS	MTB48 AAK31576	0.330	-0.176
DHDTEGLDIQIMSQV	PROBABLE ISOCITRATE DEHYDROGENASE CAA16247	0.003	-0.176
HDEDSVMPHPQQAPV	HYPOTHETICAL PROTEIN RV2623 NP_217139	0.084	-0.176
TLFPGGLNIPQNAIP	YP_177963; PPE FAMILY PROTEIN	-0.204	-0.176
ALITGNFSNGILWRG	YP_177963; PPE FAMILY PROTEIN	-0.136	-0.176
FTDLLAQPTPAWTEL	PUTATIVE CYCLOPROPANE-FATTY-ACYL-PHOSPHOLIPID SYNTHASE UFAA1 NP_854118	0.143	-0.177

WRGNYEGLAGFSFGY	YP_177963; PPE FAMILY PROTEIN	0.096	-0.177
QVDPILDDLAAALAD	MYCOBACTERIUM BOVIS MYCOCEROSIC ACID SYNTHASE GENE; M95808	-0.227	-0.178
NFKESIRDFARASFS	CAA17111.1; PROBABLE ISOCITRATE DEHYDROGENASE	0.023	-0.178
PNAGLDPVNYQNFAV	CAA98382.1; IMMUNOGENIC PROTEIN MPT64	0.049	-0.178
AYNINISLPSYYPDQ	CAA98382.1; IMMUNOGENIC PROTEIN MPT64	-0.047	-0.179
HRPDALARRIGRHGL	PUTATIVE CYCLOPROPANE-FATTY-ACYL-PHOSPHOLIPID SYNTHASE UFAA1 NP_854118	-0.117	-0.179
GEIKSQTVFMGDFPM	RNA POLYMERASE BETA-SUBUNIT AAA21416	-0.220	-0.179
EGGEIMITPEEGAYA	MYCOBACTERIUM BOVIS MYCOCEROSIC ACID SYNTHASE GENE; M95808	-0.130	-0.179
GEIWVHGDNVGNGYW	MYCOBACTERIUM BOVIS ACYL-COA SYNTHASE GENE; U75685	0.089	-0.180
TGQACQIQMSDPAYN	CAA98382.1; IMMUNOGENIC PROTEIN MPT64	-0.178	-0.180
EFITQLGRNFQVIYE	PUTATIVE ESAT-6 LIKE PROTEIN ESXN YP_177838	-0.062	-0.181
AATLHALHYHQVDVA	MTB81 (publication)	-0.056	-0.183
QFTAQIADLDQEYDD	PPE FAMILY PROTEIN CAE55489	-0.048	-0.183
LGGQNLGLGNLGDGN	YP_177963; PPE FAMILY PROTEIN	0.174	-0.184
GTGSTGLFNAGGFTT	YP_177963; PPE FAMILY PROTEIN	0.292	-0.185
FFNSGTGNWGLFNSG	YP_177963; PPE FAMILY PROTEIN	-0.008	-0.185
MLKRKAERRLIDGAL	CONSERVED HYPOTHETICAL PROTEIN TB16.3CAD97060	-0.219	-0.186
ETSWEAIEHAGLDPA	MYCOBACTERIUM BOVIS MYCOCEROSIC ACID SYNTHASE GENE; M95808	0.159	-0.186
SNTFGLQLELTEGMR	60 kDa chaperonin 2 P0A521	-0.207	-0.186
LGLFNSGTGNIGFFN	YP_177963; PPE FAMILY PROTEIN	0.164	-0.188
GNVGFGNIGDANFGL	YP_177963; PPE FAMILY PROTEIN	0.215	-0.189
SCPNTRIVLGGYSQG	PROBABLE CUTINASE PRECURSOR CFP21 NP_216500	-0.076	-0.190
DLARVGRYKVNKKLG	RNA POLYMERASE BETA-SUBUNIT AAA21416	-0.163	-0.190
SLMFVRNVGHLMTND	MTB81 (publication)	-0.113	-0.190
NVLAQLASGNLGSGN	PPE FAMILY PROTEIN CAE55504	0.303	-0.191
AGYLVLRPSWSPTQA	TRANSMEMBRANE SERINE/THREONINE-PROTEIN KINASE D PKND NP_215446	-0.117	-0.193
GQARLAASAFEATLA	PPE FAMILY PROTEIN CAE55504	-0.150	-0.193
TDVIPETDGAEKGPT	MTB81 (publication)	0.008	-0.194
LKIAQGRLFYNTRTL	POSSIBLE GLYCOSYL TRANSFERASE CAB05418	-0.136	-0.194
AIVQFVKAICSPMAL	HYPOTHETICAL PROTEIN RV2626C NP_217142	0.002	-0.194
GGTDSLLPLPNIPLL	PPE FAMILY PROTEIN CAE55504	-0.614	-0.195
AQPACLRAYPQCPLL	MYCOBACTERIUM BOVIS MYCOCEROSIC ACID SYNTHASE GENE; M95808	-0.195	-0.195
LSRERAGLEVRDVHP	RNA POLYMERASE BETA-SUBUNIT AAA21416	-0.100	-0.195
GSSRAPTESTRRCWT	MYCOBACTERIUM BOVIS MYCOCEROSIC ACID SYNTHASE GENE; M95808	-0.076	-0.195
IIYATLGSSGGKNLL	POSSIBLE GLYCOSYL TRANSFERASE CAB05418	-0.207	-0.195
FVTDPERQEAVLEDP	60 kDa chaperonin 2 P0A521	-0.184	-0.195
ILTRFGFSIPQLGFT	PPE FAMILY PROTEIN CAE55504	-0.179	-0.196
PASVQGMSQDPVAVA	CAA98373.1; MAJOR SECRETED IMMUNOGENIC PROTEIN	-0.222	-0.196
GDPIEYRSLARVYGA	MYCOBACTERIUM BOVIS MYCOCEROSIC ACID SYNTHASE GENE; M95808	-0.083	-0.196
LGNVGGGNVGFGNIG	YP_177963; PPE FAMILY PROTEIN	0.250	-0.197
IKIEASDISVAARIL	PROBABLE ISOCITRATE DEHYDROGENASE CAA16247	-0.107	-0.198
GVGNIGFANTGTNNL	YP_177963; PPE FAMILY PROTEIN	0.299	-0.198
NPGELLPEAAGPTQV	CAA98382.1; IMMUNOGENIC PROTEIN MPT64	-0.103	-0.199
VIAAESGFSVTEAIT	MYCOBACTERIUM BOVIS MYCOCEROSIC ACID SYNTHASE GENE; M95808	-0.196	-0.199
ALSAGVGAVAMSLTV	LOW MOLECULAR WEIGHT T-CELL ANTIGEN TB8.4 NP_215690	0.034	-0.199
SQFNARTADGINYRV	CAB06500.1; IMMUNOGENIC PROTEIN MPT63	-0.030	-0.200
SGNVGWANMGLGNIG	YP_177963; PPE FAMILY PROTEIN	0.347	-0.200
MVDFGALPPEINSAR	CAE55371.1; PPE FAMILY PROTEIN	0.072	-0.201
RSDKSGTSDNFQKYL	PERIPLASMIC PHOSPHATE-BINDING LIPOPROTEIN PSTS2 YP_177769	0.590	-0.201
ATIPQGEQSTGKIYF	CAB06500.1; IMMUNOGENIC PROTEIN MPT63	-0.067	-0.201
WDGVAITLTWSQLYR	MYCOBACTERIUM BOVIS ACYL-COA SYNTHASE GENE; U75685	-0.080	-0.201
ALSTLVVNKIRGTFK	60 kDa chaperonin 2 P0A521	-0.249	-0.201
LYSIGGILYAVRWPD	POSSIBLE HEMOLYSIN-LIKE PROTEIN CAA17201	-0.084	-0.201
PAWERNDPTQQIPKL	CAB10044.1; SECRETED ANTIGEN 85-B FBPB (85B)	-0.186	-0.202
AGMRREQPLPHGLRG	MYCOBACTERIUM BOVIS MYCOCEROSIC ACID SYNTHASE GENE; M95808	0.230	-0.202
ITGGLGGLGLFFASK	MYCOBACTERIUM BOVIS MYCOCEROSIC ACID SYNTHASE GENE; M95808	-0.283	-0.203
LGAGNLGNLNLGGGN	YP_177963; PPE FAMILY PROTEIN	0.044	-0.204
TTCNYGQVVAALNAT	LOW MOLECULAR WEIGHT T-CELL ANTIGEN TB8.4 NP_215690	-0.098	-0.204
FGVRLVKLLYRLERP	POSSIBLE GLYCOSYL TRANSFERASE CAB05415	-0.187	-0.205
WLSANRAVKPTGSAA	CAB10044.1; SECRETED ANTIGEN 85-B FBPB (85B)	-0.342	-0.205
KIVTPSPGTPEGPWL	MYCOBACTERIUM BOVIS ACYL-COA SYNTHASE GENE; U75685	-0.174	-0.205
GLMTSVLMTADGKTV	CAA17111.1; PROBABLE ISOCITRATE DEHYDROGENASE	-0.340	-0.206
IEYRSLARVYGAGTP	MYCOBACTERIUM BOVIS MYCOCEROSIC ACID SYNTHASE GENE; M95808	0.015	-0.206
IVGASNSTGMSGGFV	YP_177963; PPE FAMILY PROTEIN	-0.190	-0.206

KVRRGACVEQYRQDQ	MYCOBACTERIUM BOVIS ACYL-COA SYNTHASE GENE; U75685	-0.048	-0.207
LGEFLALGAGFEDIG	PROBABLE ISOCITRATE DEHYDROGENASE CAA16247	-0.021	-0.207
LGSQLRGRDGVVIVY	MYCOBACTERIUM BOVIS MYCOCEROSIC ACID SYNTHASE GENE; M95808	-0.060	-0.207
ERTVLVLRFFDSMTQ	ALTERNATE RNA POLYMERASE SIGMA FACTOR SIGF CAB07069	-0.251	-0.207
GGLPAFTLFPGGLNI	YP_177963; PPE FAMILY PROTEIN	-0.325	-0.208
SRIDGTHQTLQGADL	CAA98350.1; CELL SURFACE LIPOPROTEIN MPT83	-0.203	-0.208
GPQGTECASLSWSVQ	MYCOBACTERIUM BOVIS MYCOCEROSIC ACID SYNTHASE GENE; M95808	0.151	-0.209
GLFNSGSFNTGIGNS	YP_177963; PPE FAMILY PROTEIN	-0.082	-0.210
MTELVTGKAFPYVVV	3-OXOACYL-[ACYL-CARRIER PROTEIN] SYNTHASE 2 KASB CAA94642	-0.135	-0.210
HEHRPELAMVDSARG	PROBABLE ISOCITRATE DEHYDROGENASE CAA16247	-0.040	-0.210
NQAKYVSDTRDVLRA	CAB06237.1; CONSERVED HYPOTHETICAL PROTEIN	-0.260	-0.210
YPTVDYAFQYDGVND	PPE FAMILY PROTEIN CAE55504	0.267	-0.211
YPITGKLGSELTMTD	CAB06500.1; IMMUNOGENIC PROTEIN MPT63	-0.126	-0.211
LYRPLIFALYCLPLN	POSSIBLE GLYCOSYL TRANSFERASE CAB05418	-0.112	-0.211
NYEGLAGFSFGYPIP	YP_177963; PPE FAMILY PROTEIN	-0.172	-0.211
CWTLVSSRSARASSR	MYCOBACTERIUM BOVIS MYCOCEROSIC ACID SYNTHASE GENE; M95808	0.005	-0.212
PQCPLLLHAVDQGLQ	MYCOBACTERIUM BOVIS MYCOCEROSIC ACID SYNTHASE GENE; M95808	0.006	-0.213
RWLTDLCVQLSEVER	PPE FAMILY PROTEIN CAE55489	0.038	-0.213
PEVPLDDGDVIDSMF	PROBABLE ISOCITRATE DEHYDROGENASE CAA16247	-0.047	-0.214
ILAGASGYVVKDIKG	TWO COMPONENT TRANSCRIPTIONAL REGULATORY PROTEIN DEVR NP_217649	0.160	-0.214
GNVGDFNLGAANLGD	YP_177963; PPE FAMILY PROTEIN	-0.026	-0.215
GYAAATATATATLLP	CAE55371.1; PPE FAMILY PROTEIN	-0.183	-0.215
AKLGMERRTQAAVFA	TWO COMPONENT TRANSCRIPTIONAL REGULATORY PROTEIN DEVR NP_217649	-0.129	-0.215
KPQEGTVVAVGPGRW	CAB01005.1; 10 KDA CHAPERONIN GROES	-0.231	-0.215
PVTTAAMADPAADLI	CAA98350.1; CELL SURFACE LIPOPROTEIN MPT83	0.021	-0.216
TKDLAILIGPEQDWL	CAA17111.1; PROBABLE ISOCITRATE DEHYDROGENASE	-0.179	-0.216
AVVKLSDALIATISS	PERIPLASMIC PHOSPHATE-BINDING LIPOPROTEIN PSTS1 YP_177770	-0.065	-0.216
APIVHEMVSIPEDGG	CAA17111.1; PROBABLE ISOCITRATE DEHYDROGENASE	-0.201	-0.216
GLPAGSPEFQRHRDK	ALTERNATE RNA POLYMERASE SIGMA FACTOR SIGF CAB07069	-0.033	-0.216
SLHDGERDLALAGGC	MYCOBACTERIUM BOVIS MYCOCEROSIC ACID SYNTHASE GENE; M95808	-0.065	-0.216
APLLATYAFLPIVRA	PROBABLE ISOCITRATE DEHYDROGENASE CAA16247	-0.341	-0.217
RREHPTYEDIVGLER	MTB48 AAK31576	-0.145	-0.217
EPRVLVRRKAGEVEY	RNA POLYMERASE BETA-SUBUNIT AAA21416	-0.117	-0.217
ADGRFVEPRVLVRRK	RNA POLYMERASE BETA-SUBUNIT AAA21416	0.058	-0.217
AAKPPLGSPPPKTTV	CAA17582.1; PROBABLE SERINE PROTEASE PEPD	-0.183	-0.217
TIGGQTYTIPLDTPP	YP_177963; PPE FAMILY PROTEIN	-0.143	-0.217
FSFFAVGPDGMPGGE	YP_177963; PPE FAMILY PROTEIN	-0.163	-0.219
ATDAETTWKLLLDRQ	3-OXOACYL-[ACYL-CARRIER PROTEIN] SYNTHASE 2 KASB CAA94642	0.097	-0.219
FVNALGDNWIVGASN	YP_177963; PPE FAMILY PROTEIN	-0.180	-0.220
LKIDVSGGLPAFTLF	YP_177963; PPE FAMILY PROTEIN	0.030	-0.221
MLKGFAPAAAAQAVQ	CAE55371.1; PPE FAMILY PROTEIN	-0.062	-0.221
VSTLNLDGPTTAKIF	PERIPLASMIC PHOSPHATE-BINDING LIPOPROTEIN PSTS2 YP_177769	-0.019	-0.221
WHSLPTDRPIIYATL	POSSIBLE GLYCOSYL TRANSFERASE CAB05418	-0.216	-0.221
DLKTVPANAFVADYL	POSSIBLE GLYCOSYL TRANSFERASE CAB05415	-0.232	-0.222
PFNVNLKLQFLHDAF	YP_177963; PPE FAMILY PROTEIN	0.037	-0.222
LDLAPKDLEKIIYFA	RNA POLYMERASE BETA'-SUBUNIT AAA21417	-0.159	-0.222
VRPFALAQSLDPSRY	POSSIBLE GLYCOSYL TRANSFERASE CAB05415	-0.124	-0.222
ESVGKTIAGAKIMGQ	PERIPLASMIC PHOSPHATE-BINDING LIPOPROTEIN PSTS2 YP_177769	-0.087	-0.222
ERDERDRLVSERLLT	MYCOBACTERIUM BOVIS MYCOCEROSIC ACID SYNTHASE GENE; M95808	-0.128	-0.223
RYPPSPPPQPTQYRQ	CAA17582.1; PROBABLE SERINE PROTEASE PEPD	-0.158	-0.223
LAAYHPQQFIYAGSL	CAB10044.1; SECRETED ANTIGEN 85-B FBPB (85B)	-0.179	-0.223
TNLLGQNTPAIAVNE	CAE55371.1; PPE FAMILY PROTEIN	-0.275	-0.223
LGFTIPGFTIPAAPL	YP_177963; PPE FAMILY PROTEIN	-0.207	-0.224
DNQTGFGGLNSGAGN	YP_177963; PPE FAMILY PROTEIN	0.381	-0.224
GILWRGNYEGLAGFS	YP_177963; PPE FAMILY PROTEIN	-0.102	-0.224
APTTGVLPPAANDVS	CAE55335.1; PE FAMILY PROTEIN	-0.007	-0.224
QPLDWFCLFSSGAAL	MYCOBACTERIUM BOVIS MYCOCEROSIC ACID SYNTHASE GENE; M95808	-0.035	-0.224
FYCVANMPASVPKTS	SECRETED L-ALANINE DEHYDROGENASE ALD CAA15575	-0.125	-0.224
RAAHADPCSDIAVVF	PROBABLE CUTINASE PRECURSOR CFP21 NP_216500	-0.138	-0.225
VDGSSASELATAIAF	HYPOTHETICAL PROTEIN RV2623 NP_217139	-0.181	-0.225
GMLSSTGRCHSFDAD	MYCOBACTERIUM BOVIS MYCOCEROSIC ACID SYNTHASE GENE; M95808	-0.259	-0.225
GDRGPVWVFSGQGSQ	MYCOBACTERIUM BOVIS MYCOCEROSIC ACID SYNTHASE GENE; M95808	0.059	-0.225
DGPTTAKIFNGTITV	PERIPLASMIC PHOSPHATE-BINDING LIPOPROTEIN PSTS2 YP_177769	-0.213	-0.226
GLTFPANSLLNLDVF	YP_177963; PPE FAMILY PROTEIN	-0.105	-0.226

VTADAASIMAIVADI	CONSERVED HYPOTHETICAL PROTEIN TB18.5CAD93033	-0.117	-0.226
YGLNAKWPVYLSTKN	CAA17111.1; PROBABLE ISOCITRATE DEHYDROGENASE	0.183	-0.226
EEQSEFDVILEAAGD	CAB07109.1; PROBABLE 50S RIBOSOMAL PROTEIN L7/L12 RPLL	-0.079	-0.226
IGPTWYSVGRIYNAG	YP_177963; PPE FAMILY PROTEIN	-0.086	-0.226
ITLFPGGLTFPANSL	YP_177963; PPE FAMILY PROTEIN	-0.420	-0.227
GNSGTGSTGLFNAGS	YP_177963; PPE FAMILY PROTEIN	0.116	-0.228
EIELEDPYEKIGAEL	60 kDa chaperonin 2 P0A521	-0.107	-0.228
GTTPSSKLGGLWKTV	CAE55371.1; PPE FAMILY PROTEIN	-0.151	-0.228
SDFHAFSAAPNFAFE	MYCOBACTERIUM BOVIS ACYL-COA SYNTHASE GENE; U75685	-0.100	-0.230
QAQIDKWHRRRVIEP	MTB81 (publication)	-0.068	-0.230
VAGQTSPANVVGTRQ	CAA98373.1; MAJOR SECRETED IMMUNOGENIC PROTEIN	-0.078	-0.230
SGYFVTDPERQEAVL	60 kDa chaperonin 2 P0A521	-0.170	-0.230
PPSGSPETGAGAGTV	PERIPLASMIC PHOSPHATE-BINDING LIPOPROTEIN PSTS1 YP_177770	-0.198	-0.230
KDAVLMVVGCLGSGR	HYPOTHETICAL PROTEIN RV2623 NP_217139	-0.130	-0.231
TAAPLLLAAAGCGSK	PERIPLASMIC PHOSPHATE-BINDING LIPOPROTEIN PSTS1 YP_177770	-0.132	-0.231
LPQWLSANRAVKPTG	CAB10044.1; SECRETED ANTIGEN 85-B FBPB (85B)	-0.018	-0.232
AVCAPTTGVLPPAAN	CAE55335.1; PE FAMILY PROTEIN	-0.070	-0.232
DRLHGMLTDRDIVIK	HYPOTHETICAL PROTEIN RV2626C NP_217142	-0.146	-0.232
YATASRWGGPPVASE	POSSIBLE GLYCOSYL TRANSFERASE CAB05415	-0.230	-0.233
GPGQIEVAVSMSSIN	MYCOBACTERIUM BOVIS MYCOCEROSIC ACID SYNTHASE GENE; M95808	-0.134	-0.233
VTLAHVVRPFALAQS	POSSIBLE GLYCOSYL TRANSFERASE CAB05415	-0.227	-0.234
LWGPAFHERYPNVTI	PERIPLASMIC PHOSPHATE-BINDING LIPOPROTEIN PSTS1 YP_177770	-0.192	-0.234
RSVCPMVVAPGGTFE	CAB10947.1; PROBABLE LIPOPROTEIN LPRJ	-0.405	-0.234
RDGLAHLGFDEVFAR	PUTATIVE CYCLOPROPANE-FATTY-ACYL-PHOSPHOLIPID SYNTHASE UFAA1 NP_854118	-0.039	-0.235
NIGFANTGSNNIGIG	YP_177963; PPE FAMILY PROTEIN	0.137	-0.235
DKVLGYRNWLGLNKG	MTB81 (publication)	-0.151	-0.236
AGRLTEPHIVPIHDY	TRANSMEMBRANE SERINE/THREONINE-PROTEIN KINASE D PKND NP_215446	-0.073	-0.236
PAATIDQLKTDAKLL	CAA98350.1; CELL SURFACE LIPOPROTEIN MPT83	0.015	-0.236
MADPAADLIGRGCAQ	CAA98350.1; CELL SURFACE LIPOPROTEIN MPT83	-0.100	-0.236
SCAAPIQADMMGNAF	CAB10947.1; PROBABLE LIPOPROTEIN LPRJ	0.021	-0.236
SSNYGLANLGVGNIG	YP_177963; PPE FAMILY PROTEIN	0.236	-0.237
SGVEGLPPGSALLVV	CAB01474.1; CONSERVED HYPOTHETICAL PROTEIN CFP17	-0.477	-0.237
QNLGAANAGSGNFGF	YP_177963; PPE FAMILY PROTEIN	0.177	-0.237
SLIGLAMGDAGGYKA	CAB10044.1; SECRETED ANTIGEN 85-B FBPB (85B)	0.020	-0.238
HQNVRVNFEQLMSGY	MYCOBACTERIUM BOVIS ACYL-COA SYNTHASE GENE; U75685	-0.176	-0.238
AGLTTLAYTAATITM	POSSIBLE HEMOLYSIN-LIKE PROTEIN CAA17201	0.022	-0.238
NVGLFNSGTGNFGLF	YP_177963; PPE FAMILY PROTEIN	0.052	-0.239
AAIKELQDKGYAVPD	PROBABLE ISOCITRATE DEHYDROGENASE CAA16247	-0.111	-0.240
PLGLKRGIEKAVEKV	60 kDa chaperonin 2 P0A521	-0.096	-0.240
FGDVDAHGAMIRAQA	PUTATIVE ESAT-6 LIKE PROTEIN ESXN YP_177838	-0.008	-0.241
TGLFVPQAVTPWPNG	MYCOBACTERIUM BOVIS MYCOCEROSIC ACID SYNTHASE GENE; M95808	-0.013	-0.241
PLPPGVLRWQQDHGR	HYPOTHETICAL PROTEIN RV2623 NP_217139	-0.121	-0.241
TPGFGNTTTAPSSGF	YP_177963; PPE FAMILY PROTEIN	-0.185	-0.242
EAVIGNRRRQDSKES	MTB48 AAK31576	0.621	-0.242
MSSGNSSLGIIVGID	HYPOTHETICAL PROTEIN RV2623 NP_217139	0.082	-0.242
LDPGQPVSRLVIRGE	CAA17343.1; HEAT SHOCK PROTEIN HSP	-0.349	-0.242
DKLTGNMISNQAKYV	CAB06237.1; CONSERVED HYPOTHETICAL PROTEIN	-0.033	-0.243
KIFNGSITQWNNPAI	PERIPLASMIC PHOSPHATE-BINDING LIPOPROTEIN PSTS3 YP_177768	-0.074	-0.244
ITPDEARVEEFNLKK	CAA17111.1; PROBABLE ISOCITRATE DEHYDROGENASE	-0.134	-0.245
HRCHEHRPELAMVDS	PROBABLE ISOCITRATE DEHYDROGENASE CAA16247	-0.029	-0.246
PMSTRLGIRLVIVLV	POSSIBLE GLYCOSYL TRANSFERASE CAB05419	-0.153	-0.246
PIVIGRHAFGDQYRA	CAA17111.1; PROBABLE ISOCITRATE DEHYDROGENASE	-0.107	-0.246
IDAAIAELRHLNPLN	YP_177963; PPE FAMILY PROTEIN	-0.360	-0.247
NVGDFNLGAANIGDL	YP_177963; PPE FAMILY PROTEIN	-0.170	-0.247
QAGLEKLGSQLRGRD	MYCOBACTERIUM BOVIS MYCOCEROSIC ACID SYNTHASE GENE; M95808	-0.117	-0.247
VATSKPGQPPETVDF	MYCOBACTERIUM BOVIS ACYL-COA SYNTHASE GENE; U75685	-0.229	-0.247
LAPKGSGTEVTYELA	CONSERVED HYPOTHETICAL PROTEIN TB16.3CAD97060	-0.014	-0.248
GGLNSGAGNLGLFNS	YP_177963; PPE FAMILY PROTEIN	-0.198	-0.248
HTSMEAGPMVRKGTM	MTB81 (publication)	-0.114	-0.249
VGPGRWDEDGEKRIP	CAB01005.1; 10 KDA CHAPERONIN GROES	-0.044	-0.249
PAVPGALGENGNGGM	PERIPLASMIC PHOSPHATE-BINDING LIPOPROTEIN PSTS1 YP_177770	-0.165	-0.250
AANLGDLNLGLGNIG	YP_177963; PPE FAMILY PROTEIN	-0.042	-0.250
GDNVGNGYWQKPDES	MYCOBACTERIUM BOVIS ACYL-COA SYNTHASE GENE; U75685	-0.103	-0.250
ARWGSLYDALYGTDV	MTB81 (publication)	-0.109	-0.250

DFAGFPQHIESALRL	POSSIBLE GLYCOSYL TRANSFERASE CAB05418	-0.066	-0.250
GFVTAPTQGILIHTG	YP_177963; PPE FAMILY PROTEIN	-0.308	-0.252
PIIIVPPTPGFGNAT	YP_177963; PPE FAMILY PROTEIN	-0.261	-0.252
IDSLEEAVRDSRPAV	CONSERVED HYPOTHETICAL PROTEIN CAB08634	-0.189	-0.253
LLKQYGPLTPARAVA	TRANSMEMBRANE SERINE/THREONINE-PROTEIN KINASE D PKND NP_215446	-0.304	-0.253
ASVILRRTIDADRSF	MYCOBACTERIUM BOVIS MYCOCEROSIC ACID SYNTHASE GENE; M95808	-0.090	-0.254
PTTMGTVPNVGLMAQ	PROBABLE ISOCITRATE DEHYDROGENASE CAA16247	-0.110	-0.255
WLLIDTSNAVDTPDM	MYCOBACTERIUM BOVIS MYCOCEROSIC ACID SYNTHASE GENE; M95808	-0.301	-0.255
DTAAANQLMNNVPQA	CAE55371.1; PPE FAMILY PROTEIN	-0.191	-0.256
MVSIPEDGGVVLGMY	CAA17111.1; PROBABLE ISOCITRATE DEHYDROGENASE	-0.218	-0.256
FGNSGLGALPGIGNI	YP_177963; PPE FAMILY PROTEIN	0.279	-0.256
VGEAFVDSLTSQVGG	PROBABLE CUTINASE PRECURSOR CFP21 NP_216500	-0.183	-0.257
AGNIGLFNSGTGNVG	YP_177963; PPE FAMILY PROTEIN	-0.066	-0.257
DGRSEFAYGSFVRTV	CAA17245.1; HEAT SHOCK PROTEIN HSPX	-0.094	-0.259
YSMITQQPLGGKAQF	RNA POLYMERASE BETA-SUBUNIT AAA21416	-0.113	-0.260
DVERVAQQLLSGSEE	MYCOBACTERIUM BOVIS MYCOCEROSIC ACID SYNTHASE GENE; M95808	-0.092	-0.260
ADHGWRAACRSNPAL	SECRETED L-ALANINE DEHYDROGENASE ALD CAA15575	0.108	-0.261
ALNAANARWGSLYDA	MTB81 (publication)	-0.057	-0.261
FPVGPFFGLGVVNIS	YP_177963; PPE FAMILY PROTEIN	0.251	-0.261
LVIPDTAKEKPQEGT	CAB01005.1; 10 KDA CHAPERONIN GROES	-0.174	-0.261
QLFSVVATGAASLLT	CONSERVED HYPOTHETICAL PROTEIN TB18.5CAD93033	-0.122	-0.261
YKVTGTAPIYEILHD	CAB08889.1; IRON-REGULATED CONSERVED HYPOTHETICAL PROTEIN	-0.327	-0.262
GLGGLGLFFASKLAA	MYCOBACTERIUM BOVIS MYCOCEROSIC ACID SYNTHASE GENE; M95808	-0.073	-0.262
TILRRGDNATLLATP	TRANSMEMBRANE SERINE/THREONINE-PROTEIN KINASE D PKND NP_215446	-0.201	-0.262
AGVLTVRVAGAYKAP	CAA17343.1; HEAT SHOCK PROTEIN HSP	0.033	-0.262
GASDAYLSEGDMAAH	PERIPLASMIC PHOSPHATE-BINDING LIPOPROTEIN PSTS1 YP_177770	0.066	-0.263
FGFGGHNVAIAFGRY	3-OXOACYL-[ACYL-CARRIER PROTEIN] SYNTHASE 2 KASB CAA94642	-0.105	-0.263
IIKLPNISASVPQLV	PROBABLE ISOCITRATE DEHYDROGENASE CAA16247	-0.568	-0.263
GGAGSVACQEFITQL	PUTATIVE ESAT-6 LIKE PROTEIN ESXN YP_177838	-0.078	-0.263
AYNAAGGHNAVFNFP	CAB10044.1; SECRETED ANTIGEN 85-B FBPB (85B)	-0.092	-0.263
RLFMLSSTSSDALRQ	MYCOBACTERIUM BOVIS MYCOCEROSIC ACID SYNTHASE GENE; M95808	0.146	-0.264
ADYLPGEAAAARSAV	POSSIBLE GLYCOSYL TRANSFERASE CAB05418	-0.176	-0.264
IHWTSTGGIGPIIIP	YP_177963; PPE FAMILY PROTEIN	0.022	-0.264
DRFADFPALPLDPSA	CAB09453.1; PROBABLE SERINE PROTEASE PEPA	-0.258	-0.264
PAVSQFNARTADGIN	CAB06500.1; IMMUNOGENIC PROTEIN MPT63	-0.215	-0.264
CIKAAADRVVFINTG	MTB81 (publication)	-0.008	-0.264
IGLFNSGTGNVGLFN	YP_177963; PPE FAMILY PROTEIN	-0.192	-0.264
AQYLADTLAEEQAAA	MYCOBACTERIUM BOVIS MYCOCEROSIC ACID SYNTHASE GENE; M95808	-0.162	-0.264
VQFQSGGNNSPAVYL	CAB10044.1; SECRETED ANTIGEN 85-B FBPB (85B)	-0.112	-0.265
LIATNLLGQNTPAIA	CAE55371.1; PPE FAMILY PROTEIN	-0.752	-0.266
GHRVEIDLCDYRDVD	PUTATIVE CYCLOPROPANE-FATTY-ACYL-PHOSPHOLIPID SYNTHASE UFAA1 NP_854118	-0.193	-0.266
TPADGSAPIVHEMVS	CAA17111.1; PROBABLE ISOCITRATE DEHYDROGENASE	0.323	-0.266
AARSAVVLCNGGSPT	POSSIBLE GLYCOSYL TRANSFERASE CAB05418	-0.172	-0.266
ADAYPTVDYAFQYDG	PPE FAMILY PROTEIN CAE55504	-0.093	-0.266
PDYLTEEQRVPDNLA	PROBABLE ISOCITRATE DEHYDROGENASE CAA16247	-0.137	-0.266
MPDLRCLILTSYTSD	TWO COMPONENT TRANSCRIPTIONAL REGULATORY PROTEIN DEVR NP_217649	-0.199	-0.267
DESERTFGGKIVTPS	MYCOBACTERIUM BOVIS ACYL-COA SYNTHASE GENE; U75685	-0.052	-0.267
ATIEYLVRLHEGQTT	RNA POLYMERASE BETA-SUBUNIT AAA21416	-0.198	-0.267
ASDFAYLVDFGIARA	TRANSMEMBRANE SERINE/THREONINE-PROTEIN KINASE D PKND NP_215446	-0.082	-0.267
GAVGAVESILTVLAL	3-OXOACYL-[ACYL-CARRIER PROTEIN] SYNTHASE 2 KASB CAA94642	-0.124	-0.267
PFLEHDDANRALMGA	RNA POLYMERASE BETA-SUBUNIT AAA21416	-0.167	-0.267
AVPTLVDMSKDAVLM	HYPOTHETICAL PROTEIN RV2623 NP_217139	-0.196	-0.267
PLPTLPVPVLRDELP	CAA16030.1; PROBABLE MOLYBDOPTERIN-GUANINE DINUCLEOTIDE BIOSYNTHESIS PROTEIN	-0.217	-0.268
FGNIGGGNVGFGNIG	YP_177963; PPE FAMILY PROTEIN	0.276	-0.268
GPLVVPDIVIPAFGG	YP_177963; PPE FAMILY PROTEIN	-0.220	-0.269
MLETDLGRQSEEGSG	CAA17582.1; PROBABLE SERINE PROTEASE PEPD	-0.164	-0.269
NIGFANTGTNNLGIG	YP_177963; PPE FAMILY PROTEIN	0.003	-0.269
AGAGTVATTPASSPV	PERIPLASMIC PHOSPHATE-BINDING LIPOPROTEIN PSTS1 YP_177770	-0.115	-0.270
TIDKSTDKTLHSVKV	RNA POLYMERASE BETA-SUBUNIT AAA21416	-0.239	-0.270
FLDRTGDEIHTSMEA	MTB81 (publication)	-0.210	-0.270
LKSLEGTYRLAPKGS	CONSERVED HYPOTHETICAL PROTEIN TB16.3CAD97060	-0.127	-0.270

FAWTRGLQHRGKLDG	CAA17111.1; PROBABLE ISOCITRATE DEHYDROGENASE	-0.158	-0.270
SNAVDTPDMLASTLT	MYCOBACTERIUM BOVIS MYCOCEROSIC ACID SYNTHASE GENE; M95808	-0.464	-0.270
SGIAMAVVGGALLYL	CAB06237.1; CONSERVED HYPOTHETICAL PROTEIN	-0.130	-0.270
PPPPPQEQGLIPGFL	MTB48 AAK31576	-0.171	-0.271
MGPAAAAMAAAATPY	PPE FAMILY PROTEIN CAE55334	-0.100	-0.271
HIGLYGNAGGLGPTQ	PPE FAMILY PROTEIN CAE55334	-0.124	-0.271
NIGDVNLGFGNIGDV	YP_177963; PPE FAMILY PROTEIN	0.094	-0.271
EASDISVAARILAEF	PROBABLE ISOCITRATE DEHYDROGENASE CAA16247	-0.181	-0.272
ISPITLFPAQNFNTT	YP_177963; PPE FAMILY PROTEIN	0.022	-0.272
VFLVDDHEVVRRGLV	TWO COMPONENT TRANSCRIPTIONAL REGULATORY PROTEIN DEVR NP_217649	-0.219	-0.273
FGPDFAGFPQHIESA	POSSIBLE GLYCOSYL TRANSFERASE CAB05418	-0.189	-0.273
VASADPVDAVINTTC	LOW MOLECULAR WEIGHT T-CELL ANTIGEN TB8.4 NP_215690	-0.258	-0.273
LMTADGKTVEAEAAH	CAA17111.1; PROBABLE ISOCITRATE DEHYDROGENASE	-0.129	-0.274
PDEHSLLAGREQVRH	MYCOBACTERIUM BOVIS MYCOCEROSIC ACID SYNTHASE GENE; M95808	-0.093	-0.274
GDTNTGGFNPGSINT	YP_177963; PPE FAMILY PROTEIN	-0.090	-0.275
AESGFSVTEAITAQQ	MYCOBACTERIUM BOVIS MYCOCEROSIC ACID SYNTHASE GENE; M95808	-0.170	-0.275
MYCPSVMADVASGNL	CONSERVED HYPOTHETICAL PROTEIN CAA15739	-0.089	-0.275
VGFGNMGVGNIGFGN	YP_177963; PPE FAMILY PROTEIN	0.275	-0.275
TEYNNKAALEPVNPP	MTB48 AAK31576	0.062	-0.277
ESIRDFARASFSYGL	CAA17111.1; PROBABLE ISOCITRATE DEHYDROGENASE	-0.147	-0.277
KTAGLGLVLVFALVV	MCE-FAMILY PROTEIN MCE1AYP_177701	-0.112	-0.277
GVNIPATPGFGNTTT	YP_177963; PPE FAMILY PROTEIN	-0.249	-0.277
STGLFNAGSFNTGLA	YP_177963; PPE FAMILY PROTEIN	-0.078	-0.278
LESSSLLKSLEGTYR	CONSERVED HYPOTHETICAL PROTEIN TB16.3CAD97060	-0.336	-0.278
NEGVITVEESNTFGL	60 kDa chaperonin 2 P0A521	-0.198	-0.278
SSGFFNSGAGGVSGF	YP_177963; PPE FAMILY PROTEIN	-0.148	-0.278
PAGAAVEAAEEQSEF	CAB07109.1; PROBABLE 50S RIBOSOMAL PROTEIN L7/L12 RPLL	-0.066	-0.279
GAGVASADPVDAVIN	LOW MOLECULAR WEIGHT T-CELL ANTIGEN TB8.4 NP_215690	-0.018	-0.279
VGADPTSDIAVVRVQ	CAA17582.1; PROBABLE SERINE PROTEASE PEPD	-0.241	-0.280
AGNIGLFNSGTGNIG	YP_177963; PPE FAMILY PROTEIN	0.153	-0.280
DVETGLFVPQAVTPW	MYCOBACTERIUM BOVIS MYCOCEROSIC ACID SYNTHASE GENE; M95808	-0.221	-0.280
HIPQLVASQSAFAAK	CONSERVED HYPOTHETICAL PROTEIN TB9.8 CAD93159	-0.069	-0.280
NELIKKVKTYLKDHD	PROBABLE ISOCITRATE DEHYDROGENASE CAA16247	-0.307	-0.281
GTGSTGLFNAGSFNT	YP_177963; PPE FAMILY PROTEIN	0.048	-0.281
HTNTGGFNTGNVNTG	YP_177963; PPE FAMILY PROTEIN	0.140	-0.282
VAITLTWSQLYRRTL	MYCOBACTERIUM BOVIS ACYL-COA SYNTHASE GENE; U75685	-0.126	-0.283
HHPGDVISVTWQTKS	CAB09453.1; PROBABLE SERINE PROTEASE PEPA	-0.078	-0.283
PELDVVGEAGSVAEA	TWO COMPONENT TRANSCRIPTIONAL REGULATORY PROTEIN DEVR NP_217649	0.014	-0.283
GRNFQVIYEQANAHG	PUTATIVE ESAT-6 LIKE PROTEIN ESXN YP_177838	-0.050	-0.283
LTFTPADGSAPIVHE	CAA17111.1; PROBABLE ISOCITRATE DEHYDROGENASE	0.144	-0.284
LIETEEHAKARGANI	3-OXOACYL-[ACYL-CARRIER PROTEIN] SYNTHASE 2 KASB CAA94642	0.117	-0.284
AGLFLTTEAVVADKP	60 kDa chaperonin 2 P0A521	0.127	-0.284
DGGILTRFGFSIPQL	PPE FAMILY PROTEIN CAE55504	0.067	-0.284
IVKDAPIRDWVKLAV	PROBABLE ISOCITRATE DEHYDROGENASE CAA16247	-0.165	-0.284
QATTILRRGDNATLL	TRANSMEMBRANE SERINE/THREONINE-PROTEIN KINASE D PKND NP_215446	-0.048	-0.284
TVEVTADAASIMAIV	CONSERVED HYPOTHETICAL PROTEIN TB18.5CAD93033	-0.166	-0.285
GEWSSKELTRVLTVL	PUTATIVE CYCLOPROPANE-FATTY-ACYL-PHOSPHOLIPID SYNTHASE UFAA1 NP_854118	-0.247	-0.285
LTGDNLTGIGGLNSG	YP_177963; PPE FAMILY PROTEIN	-0.370	-0.285
SGPVVELDGDEMTRV	CAA17111.1; PROBABLE ISOCITRATE DEHYDROGENASE	-0.185	-0.285
RVAGAYKAPAETQAQ	CAA17343.1; HEAT SHOCK PROTEIN HSP	-0.037	-0.286
FADRFARFNLQERVI	MYCOBACTERIUM BOVIS ACYL-COA SYNTHASE GENE; U75685	-0.125	-0.286
FVEEFDLPVRIGGHL	3-OXOACYL-[ACYL-CARRIER PROTEIN] SYNTHASE 2 KASB CAA94642	0.056	-0.287
LPGVGDLGKLPTWTE	CAB06237.1; CONSERVED HYPOTHETICAL PROTEIN	-0.256	-0.287
EHLAENLKQRAEQLA	CONSERVED HYPOTHETICAL PROTEIN TB18.5CAD93033	-0.088	-0.287
DPRFDDVKAPVDECK	RNA POLYMERASE BETA-SUBUNIT AAA21416	-0.223	-0.288
NRPDGGILTRFGFSI	PPE FAMILY PROTEIN CAE55504	0.081	-0.288
AQSLDPSRYEVHFAC	POSSIBLE GLYCOSYL TRANSFERASE CAB05415	-0.109	-0.288
AHGQKVQAAGNNMAQ	PUTATIVE ESAT-6 LIKE PROTEIN ESXN YP_177838	0.221	-0.288
DGYPIINYEYAIVNN	PERIPLASMIC PHOSPHATE-BINDING LIPOPROTEIN PSTS1 YP_177770	-0.103	-0.288
VGGGNVGFGNIGDAN	YP_177963; PPE FAMILY PROTEIN	0.196	-0.288
LQHRGKLDGTPEVID	CAA17111.1; PROBABLE ISOCITRATE DEHYDROGENASE	-0.203	-0.288
GAAQRAGLELLPFGG	MYCOBACTERIUM BOVIS MYCOCEROSIC ACID SYNTHASE GENE; M95808	-0.134	-0.289

PVTAGWNGYGDFQHT	CONSERVED HYPOTHETICAL PROTEIN CAB08634	-0.078	-0.289
TGIGNSGTGSTGLFN	YP_177963; PPE FAMILY PROTEIN	0.054	-0.289
KHRIEDAVRNAKAAV	60 kDa chaperonin 2 P0A521	-0.140	-0.290
PKSFQAKLAAAVNAI	PERIPLASMIC PHOSPHATE-BINDING LIPOPROTEIN PSTS2 YP_177769	0.033	-0.290
IGIRVFSREDEDELP	RNA POLYMERASE BETA-SUBUNIT AAA21416	-0.125	-0.291
PPQQPGTPGYAQGQQ	CAA17582.1; PROBABLE SERINE PROTEASE PEPD	-0.135	-0.291
TTGLAGDAWHGPASL	PPE FAMILY PROTEIN CAE55504	0.016	-0.291
NLLQVVLNALADLPV	POSSIBLE GLYCOSYL TRANSFERASE CAB05418	-0.540	-0.292
HFRDNSWSVKVPRRL	ALTERNATE RNA POLYMERASE SIGMA FACTOR SIGF CAB07069	-0.140	-0.292
DSPAAQVAVRWAARD	HYPOTHETICAL PROTEIN RV2623 NP_217139	-0.176	-0.292
GNLNIDTRINQSFPV	YP_177963; PPE FAMILY PROTEIN	0.236	-0.292
NTGNTNTGIANSGNV	YP_177963; PPE FAMILY PROTEIN	-0.065	-0.292
TTGRSCQTDVDGAND	POSSIBLE HEMOLYSIN CAA16235	0.164	-0.293
LLGWVAVWYTATILH	POSSIBLE HEMOLYSIN-LIKE PROTEIN CAA17201	-0.206	-0.293
KATLPLPGGTTTLVE	CAA16030.1; PROBABLE MOLYBDOPTERIN-GUANINE DINUCLEOTIDE BIOSYNTHESIS PROTEIN	-0.031	-0.293
ASGLGDVGEAFVDSL	PROBABLE CUTINASE PRECURSOR CFP21 NP_216500	-0.234	-0.293
GLSGGSTIPAIPIGL	YP_177963; PPE FAMILY PROTEIN	-0.363	-0.294
NSGSFNTGIGNGGTG	YP_177963; PPE FAMILY PROTEIN	-0.135	-0.295
GLDPVNYQNFAVTND	CAA98382.1; IMMUNOGENIC PROTEIN MPT64	0.008	-0.295
AKERAHNAGAKNVEE	CAB08889.1; IRON-REGULATED CONSERVED HYPOTHETICAL PROTEIN	0.044	-0.295
VPNVGLMAQQAEEYG	PROBABLE ISOCITRATE DEHYDROGENASE CAA16247	0.209	-0.295
VPPLLHFNRLPDELS	MYCOBACTERIUM BOVIS MYCOCEROSIC ACID SYNTHASE GENE; M95808	0.065	-0.295
VLAGASLVAVSWAVG	POSSIBLE HEMOLYSIN-LIKE PROTEIN CAA17201	0.036	-0.296
TLGTSGGRNLLQLVL	POSSIBLE GLYCOSYL TRANSFERASE CAB05415	-0.070	-0.298
AGIPYIAIANAYWSP	POSSIBLE GLYCOSYL TRANSFERASE CAB05418	-0.103	-0.298
GIGLTGDNLTGIGGL	YP_177963; PPE FAMILY PROTEIN	-0.175	-0.298
LVQEEQPSDMTNHPR	CAA17582.1; PROBABLE SERINE PROTEASE PEPD	-0.160	-0.301
DGVGALLRYAATNRL	CONSERVED HYPOTHETICAL PROTEIN CAB08634	-0.207	-0.301
AEAFGPDFAGFPQHI	POSSIBLE GLYCOSYL TRANSFERASE CAB05418	-0.060	-0.301
TGVGNAGTASTGLFN	YP_177963; PPE FAMILY PROTEIN	-0.050	-0.301
ILELGTSAKMLSVVP	PROBABLE ISOCITRATE DEHYDROGENASE CAA16247	-0.352	-0.302
GLGNLGDGNVGFGNL	YP_177963; PPE FAMILY PROTEIN	0.093	-0.302
GFGNVGDFNLGAANI	YP_177963; PPE FAMILY PROTEIN	-0.130	-0.302
RDKIVQRCLPLADHI	ALTERNATE RNA POLYMERASE SIGMA FACTOR SIGF CAB07069	-0.187	-0.303
GLADVGNYNLGLGNV	YP_177963; PPE FAMILY PROTEIN	0.132	-0.303
NFIWGQLLLLGEGIP	CAB06237.1; CONSERVED HYPOTHETICAL PROTEIN	-0.275	-0.303
VVAMAAIATFAAPVA	CAB06500.1; IMMUNOGENIC PROTEIN MPT63	-0.061	-0.303
GNIGNANFGFGNSGL	YP_177963; PPE FAMILY PROTEIN	0.458	-0.304
SPAWNLPVVFGPIAV	PERIPLASMIC PHOSPHATE-BINDING LIPOPROTEIN PSTS3 YP_177768	0.086	-0.304
NSGSYNTGVGNAGTA	YP_177963; PPE FAMILY PROTEIN	-0.174	-0.305
EIVCSKYPDATTGTA	PERIPLASMIC PHOSPHATE-BINDING LIPOPROTEIN PSTS2 YP_177769	-0.051	-0.305
LNKARVPPYKTAGLG	MCE-FAMILY PROTEIN MCE1AYP_177701	0.018	-0.306
LGLGNVGDFNLGAAN	YP_177963; PPE FAMILY PROTEIN	0.060	-0.306
PFLADGTPVDIILNT	RNA POLYMERASE BETA-SUBUNIT AAA21416	-0.130	-0.306
LNLGLGNIGNANVGF	YP_177963; PPE FAMILY PROTEIN	0.071	-0.307
IGTLGFTIPGFTIPA	YP_177963; PPE FAMILY PROTEIN	-0.411	-0.307
LVVGNGGPGVTALVM	PPE FAMILY PROTEIN CAE55504	-0.150	-0.308
FRGRWLDPRHAGPAT	CAA16102.1; ESAT-6 LIKE PROTEIN ESXQ	-0.138	-0.308
DEDGEKRIPLDVAEG	CAB01005.1; 10 KDA CHAPERONIN GROES	-0.010	-0.308
QVNSELMARGIDDVV	MYCOBACTERIUM BOVIS MYCOCEROSIC ACID SYNTHASE GENE; M95808	-0.087	-0.308
GQPVLAIGSPLGLEG	CAA17582.1; PROBABLE SERINE PROTEASE PEPD	-0.200	-0.308
FGNSGLGAALGIGNI	YP_177963; PPE FAMILY PROTEIN	0.140	-0.309
TPDAAPGLAARRRRI	POSSIBLE GLYCOSYL TRANSFERASE CAB05415	0.023	-0.309
AHFTGAFGPVVVPPI	YP_177963; PPE FAMILY PROTEIN	-0.148	-0.309
TGFGGLNSGAGNLGL	YP_177963; PPE FAMILY PROTEIN	-0.106	-0.309
DAAKGVPDWAARLPD	RNA POLYMERASE BETA-SUBUNIT AAA21416	-0.108	-0.310
GGIGPIIIPDTTILP	YP_177963; PPE FAMILY PROTEIN	0.064	-0.310
FGSGVSGLYNTGGLP	YP_177963; PPE FAMILY PROTEIN	-0.196	-0.310
TFDTACSSGLMAVHL	MYCOBACTERIUM BOVIS MYCOCEROSIC ACID SYNTHASE GENE; M95808	-0.022	-0.310
LVRGLDTIAATGNIL	PROBABLE ISOCITRATE DEHYDROGENASE CAA16247	-0.130	-0.311
ANFGLFNSGSYNTGI	YP_177963; PPE FAMILY PROTEIN	-0.090	-0.311
GMERRTQAAVFATEL	TWO COMPONENT TRANSCRIPTIONAL REGULATORY PROTEIN DEVR NP_217649	0.317	-0.311
RKVVDGVVSDEIVYL	RNA POLYMERASE BETA-SUBUNIT AAA21416	-0.274	-0.311
VSSLNLDGPTLAKIF	PERIPLASMIC PHOSPHATE-BINDING LIPOPROTEIN PSTS3 YP_177768	-0.189	-0.311

DVNPVGGLEEVLYEL	RNA POLYMERASE BETA-SUBUNIT AAA21416	-0.075	-0.311
GTGNIGFFNSGTGNW	YP_177963; PPE FAMILY PROTEIN	0.114	-0.312
TALNQSLIINLGLAD	YP_177963; PPE FAMILY PROTEIN	-0.208	-0.313
GLGNVGNYNVGLGNV	YP_177963; PPE FAMILY PROTEIN	0.148	-0.313
GFNPGSTNTGWFNTG	YP_177963; PPE FAMILY PROTEIN	-0.012	-0.313
GLGNIGFGNTGTNNL	YP_177963; PPE FAMILY PROTEIN	0.057	-0.313
SGFANFGSLGSGVLN	YP_177963; PPE FAMILY PROTEIN	-0.138	-0.313
HATLVSDKKEDEEGV	CAB06237.1; CONSERVED HYPOTHETICAL PROTEIN	0.253	-0.314
IGKLLDNDKSPSRKT	PROBABLE ISOCITRATE DEHYDROGENASE CAA16247	-0.117	-0.314
TPTFRPSWPDHSRDQ	PUTATIVE CYCLOPROPANE-FATTY-ACYL-PHOSPHOLIPID SYNTHASE UFAA1 NP_854118	-0.126	-0.314
FTGLIAIHGLKASDV	MTB81 (publication)	-0.224	-0.314
MHVVVASYNEFGGLS	POSSIBLE GLYCOSYL TRANSFERASE CAB05419	0.023	-0.314
GPVAASAAPSIPAAN	CAA17582.1; PROBABLE SERINE PROTEASE PEPD	-0.096	-0.314
FMDLKEAARKLETGD	MTB48 AAK31576	-0.216	-0.315
RAVDRAAQIAGADAK	CAB08889.1; IRON-REGULATED CONSERVED HYPOTHETICAL PROTEIN	-0.069	-0.315
LGFGNIGDVNLGFGN	YP_177963; PPE FAMILY PROTEIN	0.249	-0.315
GVGNIGFGNTGTNNL	YP_177963; PPE FAMILY PROTEIN	0.344	-0.315
LAQIKAGDKVLIHSA	MYCOBACTERIUM BOVIS MYCOCEROSIC ACID SYNTHASE GENE; M95808	-0.013	-0.315
NEALPGTDIDPDSFW	MTB81 (publication)	-0.220	-0.316
EINSARMYAGPGSAS	CAE55371.1; PPE FAMILY PROTEIN	-0.184	-0.316
GNGGTGSTGLFNAGN	YP_177963; PPE FAMILY PROTEIN	0.020	-0.316
GGGVTLLQAAPTLDE	60 kDa chaperonin 2 P0A521	-0.278	-0.316
VIIHDEDSVMPHPQQ	HYPOTHETICAL PROTEIN RV2623 NP_217139	-0.051	-0.316
CPILAEPGGSFNTAV	CONSERVED HYPOTHETICAL PROTEIN CAA15739	-0.098	-0.316
FGTAFAMTVPPSLVA	PPE FAMILY PROTEIN CAE55334	-0.340	-0.316
IPATPGFGNTTTAPS	YP_177963; PPE FAMILY PROTEIN	-0.240	-0.317
HVKQLVEENHLRWDS	PROBABLE ISOCITRATE DEHYDROGENASE CAA16247	-0.105	-0.317
DEDDIKATYDKGILT	CAA17245.1; HEAT SHOCK PROTEIN HSPX	-0.192	-0.317
WDADDYYDPEPGVPG	MYCOBACTERIUM BOVIS MYCOCEROSIC ACID SYNTHASE GENE; M95808	-0.122	-0.317
NLGFGNIGGGNVGFG	YP_177963; PPE FAMILY PROTEIN	-0.000	-0.318
TPIGDPIEYRSLARV	MYCOBACTERIUM BOVIS MYCOCEROSIC ACID SYNTHASE GENE; M95808	-0.164	-0.318
RRLWENAGSPEVDTN	3-OXOACYL-[ACYL-CARRIER PROTEIN] SYNTHASE 2 KASB CAA94642	-0.066	-0.318
AAYCEMALAAAAEVF	MYCOBACTERIUM BOVIS MYCOCEROSIC ACID SYNTHASE GENE; M95808	-0.077	-0.319
GTGSTGLFNAGNFNT	YP_177963; PPE FAMILY PROTEIN	0.138	-0.319
VNTGWLNTGNTNTGI	YP_177963; PPE FAMILY PROTEIN	0.219	-0.319
IAIGGAATGIAAVLT	LIPOPROTEIN LPQH PRECURSOR P0A5J0	-0.203	-0.319
RGGPGGAPGCWQPIT	MCE-FAMILY PROTEIN MCE1AYP_177701	-0.163	-0.319
LKLISPQYSDNAVFR	TRANSMEMBRANE SERINE/THREONINE-PROTEIN KINASE D PKND NP_215446	0.010	-0.319
RADEEQQQALSSQMG	CAA17966.1; 10 KDA CULTURE FILTRATE ANTIGEN ESXB	-0.175	-0.319
ASRWGGPPVASEVRG	POSSIBLE GLYCOSYL TRANSFERASE CAB05415	-0.105	-0.320
KISDGDKLAGRHGNK	RNA POLYMERASE BETA-SUBUNIT AAA21416	-0.220	-0.320
RSLVRIVGVVVATTL	PROBABLE CUTINASE PRECURSOR CFP21 NP_216500	-0.108	-0.320
FKAQFEAAGLTYEHR	CAA17111.1; PROBABLE ISOCITRATE DEHYDROGENASE	-0.056	-0.320
AVVAANLPELVEGLR	MYCOBACTERIUM BOVIS MYCOCEROSIC ACID SYNTHASE GENE; M95808	-0.268	-0.320
LVTASDFAYLVDFGI	TRANSMEMBRANE SERINE/THREONINE-PROTEIN KINASE D PKND NP_215446	-0.059	-0.320
NTGAFNSGSFNNGAL	YP_177963; PPE FAMILY PROTEIN	0.210	-0.320
LLPFGGRFVEIGKAD	MYCOBACTERIUM BOVIS MYCOCEROSIC ACID SYNTHASE GENE; M95808	-0.051	-0.320
GALPICGDDDRLHGM	HYPOTHETICAL PROTEIN RV2626C NP_217142	-0.047	-0.321
RPELAMVDSARGISN	PROBABLE ISOCITRATE DEHYDROGENASE CAA16247	-0.135	-0.321
TMEQTYGVRPGAVVG	MYCOBACTERIUM BOVIS MYCOCEROSIC ACID SYNTHASE GENE; M95808	-0.214	-0.321
MTPRSLVRIVGVVVA	PROBABLE CUTINASE PRECURSOR CFP21 NP_216500	-0.039	-0.321
AAAGSFASVTTGLAG	PPE FAMILY PROTEIN CAE55504	-0.126	-0.322
SAPQGLEYVVAFLGA	MYCOBACTERIUM BOVIS ACYL-COA SYNTHASE GENE; U75685	-0.000	-0.322
KRLPMSTRLGIRLVI	POSSIBLE GLYCOSYL TRANSFERASE CAB05419	-0.270	-0.323
LPKFPGLPGLPSLPD	CAB06237.1; CONSERVED HYPOTHETICAL PROTEIN	-0.248	-0.323
LNIHQTFSLGPLVVP	YP_177963; PPE FAMILY PROTEIN	-0.215	-0.323
GTFARPMTELVTGKA	3-OXOACYL-[ACYL-CARRIER PROTEIN] SYNTHASE 2 KASB CAA94642	-0.018	-0.323
SWLPFYHDMGLVIGI	MYCOBACTERIUM BOVIS ACYL-COA SYNTHASE GENE; U75685	-0.154	-0.323
LGNVGSYNLGFANVG	YP_177963; PPE FAMILY PROTEIN	-0.006	-0.323
RAPKAVKEYARKHPH	PROBABLE ISOCITRATE DEHYDROGENASE CAA16247	-0.085	-0.323
NIGFFNSGTGNFGVF	YP_177963; PPE FAMILY PROTEIN	-0.051	-0.324
VYGNTRLGLFPFRRG	MYCOBACTERIUM BOVIS MYCOCEROSIC ACID SYNTHASE GENE; M95808	-0.200	-0.324
DNQTGIGGLNSGAGN	YP_177963; PPE FAMILY PROTEIN	0.321	-0.324

ALYDAAVGHGDRGPV	MYCOBACTERIUM BOVIS MYCOCEROSIC ACID SYNTHASE GENE; M95808	-0.298	-0.324
TEVQGEPVDIGGYYA	PROBABLE ISOCITRATE DEHYDROGENASE CAA16247	-0.234	-0.324
FEQACPGQTLNYTAN	PERIPLASMIC PHOSPHATE-BINDING LIPOPROTEIN PSTS3 YP_177768	-0.041	-0.325
RSPGVYFDETIDKST	RNA POLYMERASE BETA-SUBUNIT AAA21416	-0.115	-0.325
YTIPLDTPPAPAPPP	YP_177963; PPE FAMILY PROTEIN	-0.052	-0.325
HEIDARDTKLGAEEI	RNA POLYMERASE BETA-SUBUNIT AAA21416	-0.114	-0.325
GKLKDTKAVNPESTF	PROBABLE ISOCITRATE DEHYDROGENASE CAA16247	-0.062	-0.325
GSVSSGLLRHAHCPV	HYPOTHETICAL PROTEIN RV2623 NP_217139	-0.123	-0.325
SGLGLPPGMGNIGLG	YP_177963; PPE FAMILY PROTEIN	-0.154	-0.325
VGNVGLSTIAGRLLG	CAB08889.1; IRON-REGULATED CONSERVED HYPOTHETICAL PROTEIN	-0.694	-0.325
TSIAISMYCPSVMAD	CONSERVED HYPOTHETICAL PROTEIN CAA15739	-0.175	-0.326
PESFKVLLKELQSLC	RNA POLYMERASE BETA-SUBUNIT AAA21416	-0.426	-0.326
SPAVYLLDGLRAQDD	CAB10044.1; SECRETED ANTIGEN 85-B FBPB (85B)	-0.551	-0.326
GLVGFSYSIEITGST	YP_177963; PPE FAMILY PROTEIN	-0.168	-0.326
DTDPLPVVFPIVQGE	CAA98382.1; IMMUNOGENIC PROTEIN MPT64	-0.264	-0.327
SGMFNVSTLGSAPAV	YP_177963; PPE FAMILY PROTEIN	-0.321	-0.327
GTAVKAFLQSTIGAG	PERIPLASMIC PHOSPHATE-BINDING LIPOPROTEIN PSTS3 YP_177768	-0.189	-0.327
VKPHDSGERANLEQA	MYCOBACTERIUM BOVIS MYCOCEROSIC ACID SYNTHASE GENE; M95808	-0.123	-0.327
RIPLDVAEGDTVIYS	CAB01005.1; 10 KDA CHAPERONIN GROES	-0.175	-0.327
VIVRELTEVQGEPVD	PROBABLE ISOCITRATE DEHYDROGENASE CAA16247	-0.253	-0.328
QFEAEIGRGSGLAAQ	CONSERVED HYPOTHETICAL PROTEIN CAB08634	-0.187	-0.328
VPDIHDVALMEDRAT	MTB81 (publication)	-0.206	-0.328
RRMGRDKATLPLPGG	CAA16030.1; PROBABLE MOLYBDOPTERIN-GUANINE DINUCLEOTIDE BIOSYNTHESIS PROTEIN	0.242	-0.328
VPHGESGKVIGIRVF	RNA POLYMERASE BETA-SUBUNIT AAA21416	-0.231	-0.329
SSLLKSLEGTYRLAP	CONSERVED HYPOTHETICAL PROTEIN TB16.3CAD97060	-0.250	-0.330
PFDLPTVVIDRIPVE	YP_177963; PPE FAMILY PROTEIN	-0.215	-0.330
PYLVMDTGASLAPYN	MCE-FAMILY PROTEIN MCE1AYP_177701	-0.362	-0.330
NSGSYNTGIGNSGTG	YP_177963; PPE FAMILY PROTEIN	0.009	-0.330
TETGIRLTPKVIATN	MYCOBACTERIUM BOVIS MYCOCEROSIC ACID SYNTHASE GENE; M95808	-0.186	-0.331
PLPVVFPIVQGELSK	CAA98382.1; IMMUNOGENIC PROTEIN MPT64	-0.085	-0.331
FPGGLTFPANSLLNL	YP_177963; PPE FAMILY PROTEIN	-0.264	-0.331
NIVTSAGGDPVAITI	PERIPLASMIC PHOSPHATE-BINDING LIPOPROTEIN PSTS3 YP_177768	-0.156	-0.333
RQKDAATAQTLQAFL	PERIPLASMIC PHOSPHATE-BINDING LIPOPROTEIN PSTS1 YP_177770	-0.172	-0.334
DALKSHGPQGTECAS	MYCOBACTERIUM BOVIS MYCOCEROSIC ACID SYNTHASE GENE; M95808	-0.106	-0.334
DNNALGQFTAQIADL	PPE FAMILY PROTEIN CAE55489	-0.079	-0.334
IVNANTVLDDLNSRM	MCE-FAMILY PROTEIN MCE1AYP_177701	-0.063	-0.334
GAAEKQDPLSGLTDQ	TWO COMPONENT TRANSCRIPTIONAL REGULATORY PROTEIN DEVR NP_217649	-0.145	-0.335
SDQDAMARLGAIKRE	MYCOBACTERIUM BOVIS ACYL-COA SYNTHASE GENE; U75685	-0.090	-0.336
FANTGTNNLGIGLTG	YP_177963; PPE FAMILY PROTEIN	-0.101	-0.336
SSVPIIIVPPTPGFG	YP_177963; PPE FAMILY PROTEIN	-0.278	-0.336
GTECASLSWSVQDTP	MYCOBACTERIUM BOVIS MYCOCEROSIC ACID SYNTHASE GENE; M95808	-0.037	-0.336
QALNRDFTLPGERIH	PERIPLASMIC PHOSPHATE-BINDING LIPOPROTEIN PSTS3 YP_177768	0.294	-0.336
PQGEQSTGKIYFDVT	CAB06500.1; IMMUNOGENIC PROTEIN MPT63	-0.280	-0.337
SGAGIAQAAAGTVNI	PERIPLASMIC PHOSPHATE-BINDING LIPOPROTEIN PSTS1 YP_177770	-0.178	-0.337
VKKPETINYRTLKPE	RNA POLYMERASE BETA'-SUBUNIT AAA21417	0.234	-0.337
VRVYVAQKRKISDGD	RNA POLYMERASE BETA-SUBUNIT AAA21416	-0.124	-0.337
RSCQTDVDGANDDWN	POSSIBLE HEMOLYSIN CAA16235	0.040	-0.338
ALGRSVCPMVVAPGG	CAB10947.1; PROBABLE LIPOPROTEIN LPRJ	-0.194	-0.338
MHFEAYPPEVNSANI	PPE FAMILY PROTEIN CAE55489	-0.112	-0.338
TWVEEHQDCVAASDL	MYCOBACTERIUM BOVIS MYCOCEROSIC ACID SYNTHASE GENE; M95808	0.037	-0.339
TGIGGLNSGAGNIGL	YP_177963; PPE FAMILY PROTEIN	-0.099	-0.339
FSLGPLVVPDIVIPA	YP_177963; PPE FAMILY PROTEIN	-0.300	-0.339
VLLCCSGVATAAPKT	CAA98382.1; IMMUNOGENIC PROTEIN MPT64	-0.195	-0.340
VGAPVDALVNLADEE	CAB08889.1; IRON-REGULATED CONSERVED HYPOTHETICAL PROTEIN	0.058	-0.340
AGSPEFQRHRDKIVQ	ALTERNATE RNA POLYMERASE SIGMA FACTOR SIGF CAB07069	-0.102	-0.340
PSSKLGGLWKTVSPH	CAE55371.1; PPE FAMILY PROTEIN	-0.182	-0.340
QGVQQKWDATATELN	CAE55648	-0.011	-0.340
LGWQQRALPEVGDGE	MYCOBACTERIUM BOVIS MYCOCEROSIC ACID SYNTHASE GENE; M95808	-0.119	-0.341
RPITPTFRPSWPDHS	PUTATIVE CYCLOPROPANE-FATTY-ACYL-PHOSPHOLIPID SYNTHASE UFAA1 NP_854118	-0.156	-0.341
LTSAAERGPGQMLGG	CAE55371.1; PPE FAMILY PROTEIN	-0.219	-0.341
VAVAGAAILVAGLSG	LIPOPROTEIN LPQH PRECURSOR P0A5J0	-0.018	-0.341
VNDALSGLGLPPPWQ	PPE FAMILY PROTEIN CAE55504	-0.600	-0.341
QDKGYAVPDYPADPK	PROBABLE ISOCITRATE DEHYDROGENASE CAA16247	-0.087	-0.341

FFNSGTANFGLFNSG	YP_177963; PPE FAMILY PROTEIN	-0.123	-0.341
APSSGFFNSGDGGVS	YP_177963; PPE FAMILY PROTEIN	-0.132	-0.341
ANTKKVAMAAPMFSI	POSSIBLE GLYCOSYL TRANSFERASE CAB05419	0.019	-0.342
VRSEAPLVGTGMELR	RNA POLYMERASE BETA-SUBUNIT AAA21416	0.187	-0.342
GLGDKFGESIVNANT	MCE-FAMILY PROTEIN MCE1AYP_177701	-0.074	-0.342
AVRGLRMGTGTSERD	MYCOBACTERIUM BOVIS MYCOCEROSIC ACID SYNTHASE GENE; M95808	-0.039	-0.342
PGPESMLAAAAAWDG	PPE FAMILY PROTEIN CAE55334	0.019	-0.342
LIIASAYLPQHEDAR	CAB08889.1; IRON-REGULATED CONSERVED HYPOTHETICAL PROTEIN	-0.276	-0.343
GDKVLIHSATGGVGQ	MYCOBACTERIUM BOVIS MYCOCEROSIC ACID SYNTHASE GENE; M95808	-0.098	-0.343
GTSLRALLKQYGPLT	TRANSMEMBRANE SERINE/THREONINE-PROTEIN KINASE D PKND NP_215446	0.175	-0.343
GPGVTALVMGGTDSL	PPE FAMILY PROTEIN CAE55504	-0.295	-0.344
AAHKGLMNIALAISA	PERIPLASMIC PHOSPHATE-BINDING LIPOPROTEIN PSTS1 YP_177770	-0.085	-0.344
NWMKRADHSMIFVFI	POSSIBLE HEMOLYSIN-LIKE PROTEIN CAA17201	-0.227	-0.344
FFNSGTGNFGVFNSG	YP_177963; PPE FAMILY PROTEIN	-0.116	-0.344
PPAVVKLSDALIATI	PERIPLASMIC PHOSPHATE-BINDING LIPOPROTEIN PSTS1 YP_177770	-0.201	-0.345
HRATLGQPLDWFCLF	MYCOBACTERIUM BOVIS MYCOCEROSIC ACID SYNTHASE GENE; M95808	-0.155	-0.345
GGLNSGTGNLGLFNS	YP_177963; PPE FAMILY PROTEIN	-0.149	-0.345
EVYEAEDTRKHRVVA	TRANSMEMBRANE SERINE/THREONINE-PROTEIN KINASE D PKND NP_215446	0.189	-0.345
EPVNPPKPPPAIKID	MTB48 AAK31576	-0.163	-0.345
GTAVLAGASLVAVSW	POSSIBLE HEMOLYSIN-LIKE PROTEIN CAA17201	-0.155	-0.346
APGGGQFTLPGRSLM	MTB81 (publication)	0.051	-0.346
ATLAHVVRPFVLARS	POSSIBLE GLYCOSYL TRANSFERASE CAB05418	-0.168	-0.347
TGSFNAGNTNTGGFN	YP_177963; PPE FAMILY PROTEIN	-0.090	-0.347
GITGNGQIGFGKPAN	PPE FAMILY PROTEIN CAE55504	-0.231	-0.347
QVMGDLDKLTGNMIS	CAB06237.1; CONSERVED HYPOTHETICAL PROTEIN	-0.280	-0.348
SGSTAQENAMEQFVY	PERIPLASMIC PHOSPHATE-BINDING LIPOPROTEIN PSTS2 YP_177769	-0.106	-0.348
DYLPGEAAAARSAVV	POSSIBLE GLYCOSYL TRANSFERASE CAB05415	-0.083	-0.348
SALAANADPLTSGLL	PPE FAMILY PROTEIN CAE55334	0.151	-0.348
QAMASTEGNVTGMFA	CAE55648	-0.125	-0.348
HVSRLLAKSLARLRD	ALTERNATE RNA POLYMERASE SIGMA FACTOR SIGF CAB07069	-0.350	-0.348
ELRAAIDAATSSSQE	RNA POLYMERASE BETA-SUBUNIT AAA21416	-0.146	-0.348
IAATSFAAAGLAALA	CAA98373.1; MAJOR SECRETED IMMUNOGENIC PROTEIN	-0.063	-0.349
VARAQGMSQDMAQTF	CONSERVED HYPOTHETICAL PROTEIN CAA15739	-0.062	-0.349
YRADIYALACVLGEC	TRANSMEMBRANE SERINE/THREONINE-PROTEIN KINASE D PKND NP_215446	-0.242	-0.349
FNTGVANVGSYNTGS	YP_177963; PPE FAMILY PROTEIN	-0.114	-0.349
ANDDWNPASKRGERE	POSSIBLE HEMOLYSIN CAA16235	-0.034	-0.349
SFNTGIGNSGTGSTG	YP_177963; PPE FAMILY PROTEIN	0.070	-0.349
EALPFAAIAVGAALV	CONSERVED HYPOTHETICAL PROTEIN CAB08634	-0.149	-0.349
MLRLNAGLGNVGNYN	YP_177963; PPE FAMILY PROTEIN	-0.240	-0.350
GTVGEIWVHGDNVGN	MYCOBACTERIUM BOVIS ACYL-COA SYNTHASE GENE; U75685	0.165	-0.350
CIECPDGTVGEIWVH	MYCOBACTERIUM BOVIS ACYL-COA SYNTHASE GENE; U75685	-0.008	-0.350
LADVGSVNVGFGNVG	YP_177963; PPE FAMILY PROTEIN	0.050	-0.350
ILEADDEGYPKRARM	CONSERVED HYPOTHETICAL PROTEIN TB16.3CAD97060	-0.222	-0.350
YDDGRPSQVRLDTAV	CONSERVED HYPOTHETICAL PROTEIN TB18.5CAD93033	-0.215	-0.350
VAAIKEFFGTSQLSQ	RNA POLYMERASE BETA-SUBUNIT AAA21416	-0.136	-0.350
AITRVVVRDQPARQL	HYPOTHETICAL PROTEIN RV2623 NP_217139	-0.127	-0.351
MELARAVKDVGAGRS	TWO COMPONENT TRANSCRIPTIONAL REGULATORY PROTEIN DEVR NP_217649	-0.225	-0.351
MSAYKTVVVGTDGSD	CAB08889.1; IRON-REGULATED CONSERVED HYPOTHETICAL PROTEIN	-0.111	-0.352
GSTRTPAGVVMSHQN	MYCOBACTERIUM BOVIS ACYL-COA SYNTHASE GENE; U75685	-0.189	-0.352
MADVASGNLPALPDM	CONSERVED HYPOTHETICAL PROTEIN CAA15739	-0.198	-0.352
VLVRRKAGEVEYVPS	RNA POLYMERASE BETA-SUBUNIT AAA21416	-0.294	-0.352
VAGFDAEFFGISERE	MYCOBACTERIUM BOVIS MYCOCEROSIC ACID SYNTHASE GENE; M95808	-0.215	-0.352
NEGDQIDKGDVVVLL	CAB08316.1; BIOTINYLATED PROTEIN TB7.3	-0.117	-0.352
PPEINSVLMYSGAGS	YP_177963; PPE FAMILY PROTEIN	-0.240	-0.353
WSFAVGKQLNMAQII	PERIPLASMIC PHOSPHATE-BINDING LIPOPROTEIN PSTS2 YP_177769	-0.186	-0.353
VKNYVSRLLAKLGME	TWO COMPONENT TRANSCRIPTIONAL REGULATORY PROTEIN DEVR NP_217649	-0.301	-0.353
APPPIAHSTVLVAPV	PPE FAMILY PROTEIN CAE55489	-0.244	-0.353
LGQGRAGGGAALGGG	MTB48 AAK31576	-0.095	-0.353
RHLPEHAIVQFVKAI	HYPOTHETICAL PROTEIN RV2626C NP_217142	-0.138	-0.353
EGYPKRARMLMDAAI	CONSERVED HYPOTHETICAL PROTEIN TB16.3CAD97060	-0.193	-0.353
GTYVNREPVDSAVLA	CAB01474.1; CONSERVED HYPOTHETICAL PROTEIN CFP17	-0.342	-0.354
LLAASAGILSAAAFA	CONSERVED HYPOTHETICAL PROTEIN CAA15739	-0.137	-0.354

VPSSEVDYMDVSPRQ	RNA POLYMERASE BETA-SUBUNIT AAA21416	0.014	-0.354
GFGNIGDANFGFGNS	YP_177963; PPE FAMILY PROTEIN	0.040	-0.354
PIHLGLTGQANYGFA	YP_177963; PPE FAMILY PROTEIN	-0.091	-0.354
AVLEDPYILLVSSKV	60 kDa chaperonin 2 P0A521	-0.096	-0.355
KKVAMAAPMFSIIIP	POSSIBLE GLYCOSYL TRANSFERASE CAB05419	-0.236	-0.355
VNARGGPGGAPGCWQ	MCE-FAMILY PROTEIN MCE1AYP_177701	-0.087	-0.355
RKAMLQDMAILTGGQ	60 kDa chaperonin 2 P0A521	-0.246	-0.355
SAVVLCNGGSPTTQQ	POSSIBLE GLYCOSYL TRANSFERASE CAB05418	-0.203	-0.355
IPNISDEVLADLDER	RNA POLYMERASE BETA-SUBUNIT AAA21416	-0.613	-0.356
RLVSERLLTLGWQQR	MYCOBACTERIUM BOVIS MYCOCEROSIC ACID SYNTHASE GENE; M95808	-0.471	-0.357
VINTTCNYGQVVAAL	LOW MOLECULAR WEIGHT T-CELL ANTIGEN TB8.4 NP_215690	-0.172	-0.357
QANLGEAAGTYVAAD	CONSERVED HYPOTHETICAL PROTEIN TB9.8 CAD93159	-0.139	-0.357
LDGTPEVIDFAHKLE	CAA17111.1; PROBABLE ISOCITRATE DEHYDROGENASE	-0.130	-0.358
DYMDVSPRQMVSVAT	RNA POLYMERASE BETA-SUBUNIT AAA21416	-0.144	-0.358
TLAAGSNNQTVLPFD	TRANSMEMBRANE SERINE/THREONINE-PROTEIN KINASE D PKND NP_215446	-0.153	-0.359
DCPPPGYDITALLQA	MYCOBACTERIUM BOVIS MYCOCEROSIC ACID SYNTHASE GENE; M95808	0.039	-0.359
AKGLSTHEGALLSER	SECRETED L-ALANINE DEHYDROGENASE ALD CAA15575	-0.161	-0.359
IPMSGIAMAVVGGAL	CAB06237.1; CONSERVED HYPOTHETICAL PROTEIN	-0.064	-0.359
AVSPEVATWLEVPLP	HYPOTHETICAL PROTEIN RV2623 NP_217139	-0.199	-0.360
DKPEKEKASVPGGGD	60 kDa chaperonin 2 P0A521	-0.106	-0.360
TTLAYTAATITMFTV	POSSIBLE HEMOLYSIN-LIKE PROTEIN CAA17201	-0.228	-0.360
KVQPAVFAVQVALAA	MYCOBACTERIUM BOVIS MYCOCEROSIC ACID SYNTHASE GENE; M95808	-0.226	-0.360
GWQERYPNVAITRVV	HYPOTHETICAL PROTEIN RV2623 NP_217139	-0.234	-0.361
ANSGNVNTGAFISGN	YP_177963; PPE FAMILY PROTEIN	-0.102	-0.361
AEVRDITFEQMLLLD	MYCOBACTERIUM BOVIS MYCOCEROSIC ACID SYNTHASE GENE; M95808	-0.153	-0.361
ANFGSLGSGVLNFGS	YP_177963; PPE FAMILY PROTEIN	-0.205	-0.362
GNAGLAAGLAGVGNI	YP_177963; PPE FAMILY PROTEIN	0.127	-0.362
NRFGAAVGVLAAGAL	PERIPLASMIC PHOSPHATE-BINDING LIPOPROTEIN PSTS3 YP_177768	-0.043	-0.362
AAGGGPSQLASMGSQ	CAB06237.1; CONSERVED HYPOTHETICAL PROTEIN	-0.193	-0.363
QLSGLSSAGTALNQS	YP_177963; PPE FAMILY PROTEIN	-0.320	-0.363
CWPAAPRSVGVPLYL	POSSIBLE HEMOLYSIN-LIKE PROTEIN CAA17201	-0.177	-0.363
PAVISGFGNLGNHVS	YP_177963; PPE FAMILY PROTEIN	-0.122	-0.363
GLFNSGSYNTGIGNS	YP_177963; PPE FAMILY PROTEIN	-0.140	-0.363
FAPNLGERLIIHRDT	POSSIBLE GLYCOSYL TRANSFERASE CAB05419	-0.142	-0.364
SERLLTLGWQQRALP	MYCOBACTERIUM BOVIS MYCOCEROSIC ACID SYNTHASE GENE; M95808	-0.201	-0.364
PIDAVASIDAPGVVN	MYCOBACTERIUM BOVIS MYCOCEROSIC ACID SYNTHASE GENE; M95808	-0.042	-0.364
YTPFALLALPAHDGR	POSSIBLE HEMOLYSIN-LIKE PROTEIN CAA17201	-0.206	-0.364
DVNLGFGNIGDVNLG	YP_177963; PPE FAMILY PROTEIN	-0.093	-0.364
AWTEAVIGNRRRQDS	MTB48 AAK31576	-0.084	-0.366
STASAGTVGLIKAIL	MYCOBACTERIUM BOVIS MYCOCEROSIC ACID SYNTHASE GENE; M95808	-0.161	-0.366
GAATAGAFSRPGLPV	CAB10044.1; SECRETED ANTIGEN 85-B FBPB (85B)	-0.238	-0.366
WRGAAGTAAQAAVVR	CAA17966.1; 10 KDA CULTURE FILTRATE ANTIGEN ESXB	-0.083	-0.367
TIKAERTEQKDFDGR	CAA17245.1; HEAT SHOCK PROTEIN HSPX	-0.077	-0.367
QQRALPEVGDGEAGS	MYCOBACTERIUM BOVIS MYCOCEROSIC ACID SYNTHASE GENE; M95808	-0.103	-0.367
GIDDSPAAQVAVRWA	HYPOTHETICAL PROTEIN RV2623 NP_217139	-0.235	-0.367
AGGGLSGVLRVPPRP	CAE55371.1; PPE FAMILY PROTEIN	-0.092	-0.367
KIFNGTITVWNDPQI	PERIPLASMIC PHOSPHATE-BINDING LIPOPROTEIN PSTS2 YP_177769	-0.129	-0.367
NYGDPVDAKALGQSV	CONSERVED HYPOTHETICAL PROTEIN CAA15739	-0.178	-0.368
HTKHYRVVSKPAALV	CAE55335.1; PE FAMILY PROTEIN	0.319	-0.368
LPGAAYCEMALAAAA	MYCOBACTERIUM BOVIS MYCOCEROSIC ACID SYNTHASE GENE; M95808	-0.169	-0.368
GISEREATSIDPQQR	MYCOBACTERIUM BOVIS MYCOCEROSIC ACID SYNTHASE GENE; M95808	-0.077	-0.368
GLILTNNHVIAAAAK	CAA17582.1; PROBABLE SERINE PROTEASE PEPD	0.078	-0.368
EDPYILLVSSKVSTV	60 kDa chaperonin 2 P0A521	-0.135	-0.369
EGGALLLIETEEHAK	3-OXOACYL-[ACYL-CARRIER PROTEIN] SYNTHASE 2 KASB CAA94642	-0.152	-0.369
RGLGEAQLGNSSGNF	PERIPLASMIC PHOSPHATE-BINDING LIPOPROTEIN PSTS1 YP_177770	-0.155	-0.369
ETNRDGETTRHATAA	MYCOBACTERIUM BOVIS MYCOCEROSIC ACID SYNTHASE GENE; M95808	-0.262	-0.369
GVGVGASGNNGTSAL	PERIPLASMIC PHOSPHATE-BINDING LIPOPROTEIN PSTS2 YP_177769	0.064	-0.369
KTDDVAGDGTTTATV	60 kDa chaperonin 2 P0A521	-0.109	-0.370
WNQAQTEVAGAGSGF	YP_177963; PPE FAMILY PROTEIN	-0.241	-0.370
GRSDLKTVPANAFVA	POSSIBLE GLYCOSYL TRANSFERASE CAB05415	-0.143	-0.370
LINGVNIPATPGFGN	YP_177963; PPE FAMILY PROTEIN	-0.262	-0.370
NSEEFLDAIADNLEK	CAA17111.1; PROBABLE ISOCITRATE DEHYDROGENASE	-0.182	-0.371
GSPETGAGAGTVATT	PERIPLASMIC PHOSPHATE-BINDING LIPOPROTEIN PSTS1 YP_177770	-0.208	-0.371
GQVWEATATVNAIRG	CAB06500.1; IMMUNOGENIC PROTEIN MPT63	-0.116	-0.372
GDDDRLHGMLTDRDI	HYPOTHETICAL PROTEIN RV2626C NP_217142	-0.200	-0.372

MTNHPRYSPPPQQPG	CAA17582.1; PROBABLE SERINE PROTEASE PEPD	-0.062	-0.372
CQGDLGMSHQDWQAQ	CAA16102.1; ESAT-6 LIKE PROTEIN ESXQ	-0.108	-0.372
ASAFQSVVWGLTVGS	CAE55371.1; PPE FAMILY PROTEIN	-0.185	-0.372
CAPILGGYPAVLTSP	MYCOBACTERIUM BOVIS ACYL-COA SYNTHASE GENE; U75685	0.093	-0.372
KTIVLKPEVPLDDGD	PROBABLE ISOCITRATE DEHYDROGENASE CAA16247	-0.149	-0.373
NAKWPVYLSTKNTIL	CAA17111.1; PROBABLE ISOCITRATE DEHYDROGENASE	-0.087	-0.373
GAYKAPAETQAQRIA	CAA17343.1; HEAT SHOCK PROTEIN HSP	0.011	-0.373
AGRHPDSDIFLDDVT	CAB01474.1; CONSERVED HYPOTHETICAL PROTEIN CFP17	-0.110	-0.373
APTLDELKLEGDEAT	60 kDa chaperonin 2 P0A521	-0.235	-0.374
GSIYIVKPKMHGPAE	MTB81 (publication)	-0.198	-0.374
TGLPLRGVLHSAAVV	MYCOBACTERIUM BOVIS MYCOCEROSIC ACID SYNTHASE GENE; M95808	-0.139	-0.374
GFGPIAVPSVTIPAL	YP_177963; PPE FAMILY PROTEIN	-0.292	-0.375
FFNTGGGGGSGFSNS	YP_177963; PPE FAMILY PROTEIN	-0.088	-0.375
GGQFTLPGRSLMFVR	MTB81 (publication)	0.092	-0.375
GVFNSGSYNTGVGNA	YP_177963; PPE FAMILY PROTEIN	-0.144	-0.376
LTSLTSAAERGPGQM	CAE55371.1; PPE FAMILY PROTEIN	-0.227	-0.376
QHEDARAADILKDES	CAB08889.1; IRON-REGULATED CONSERVED HYPOTHETICAL PROTEIN	0.149	-0.376
AVADAQATLAQLPPG	PPE FAMILY PROTEIN CAE55334	-0.228	-0.376
LTTLPKFPGLPGLPS	CAB06237.1; CONSERVED HYPOTHETICAL PROTEIN	-0.285	-0.376
VNPVLRQGNSDRRAP	PROBABLE ISOCITRATE DEHYDROGENASE CAA16247	-0.243	-0.376
IDPQQRLLLETSWEA	MYCOBACTERIUM BOVIS MYCOCEROSIC ACID SYNTHASE GENE; M95808	-0.254	-0.377
RLDTAVQGIEGTYIH	CONSERVED HYPOTHETICAL PROTEIN TB18.5CAD93033	-0.169	-0.377
QTDVDGANDDWNPAS	POSSIBLE HEMOLYSIN CAA16235	0.118	-0.377
TWQTKSGGTRTGNVT	CAB09453.1; PROBABLE SERINE PROTEASE PEPA	0.108	-0.377
PAVTIFPNGISIPNN	YP_177963; PPE FAMILY PROTEIN	-0.219	-0.377
IYRKLRPGEPPTKES	RNA POLYMERASE BETA-SUBUNIT AAA21416	-0.207	-0.378
KESAQTLLENLFFKE	RNA POLYMERASE BETA-SUBUNIT AAA21416	-0.060	-0.379
VLSWLSDHQVHNVAA	MYCOBACTERIUM BOVIS MYCOCEROSIC ACID SYNTHASE GENE; M95808	-0.099	-0.379
AASVGATSEIEPFIV	YP_177963; PPE FAMILY PROTEIN	-0.194	-0.380
LPADTAAQLTSAGRE	MTB48 AAK31576	-0.246	-0.380
LTVRVAGAYKAPAET	CAA17343.1; HEAT SHOCK PROTEIN HSP	-0.160	-0.380
REQVRHLVRITRELA	MYCOBACTERIUM BOVIS MYCOCEROSIC ACID SYNTHASE GENE; M95808	-0.156	-0.380
TGNIGLFNSGTGNIG	YP_177963; PPE FAMILY PROTEIN	0.022	-0.380
WRGNFEGLFGLNVGI	YP_177963; PPE FAMILY PROTEIN	-0.169	-0.381
YPNVTITAQGTGSGA	PERIPLASMIC PHOSPHATE-BINDING LIPOPROTEIN PSTS1 YP_177770	-0.200	-0.381
TGPYSMITQQPLGGK	RNA POLYMERASE BETA-SUBUNIT AAA21416	-0.160	-0.381
AQDPVATAASNNPML	CAA98350.1; CELL SURFACE LIPOPROTEIN MPT83	-0.155	-0.382
IAFLAGCSSTKPVSQ	CAA98350.1; CELL SURFACE LIPOPROTEIN MPT83	-0.186	-0.382
IDGFSTPPITIDRIP	YP_177963; PPE FAMILY PROTEIN	-0.315	-0.382
LAAAAAWDGVAAELT	PPE FAMILY PROTEIN CAE55334	-0.135	-0.383
KERKHRIEDAVRNAK	60 kDa chaperonin 2 P0A521	-0.198	-0.383
GSTGDRVVISAPQGL	MYCOBACTERIUM BOVIS ACYL-COA SYNTHASE GENE; U75685	-0.054	-0.383
RLLAKSLARLRDQLE	ALTERNATE RNA POLYMERASE SIGMA FACTOR SIGF CAB07069	-0.253	-0.384
IFAVVRGTATNQDGR	MYCOBACTERIUM BOVIS MYCOCEROSIC ACID SYNTHASE GENE; M95808	-0.096	-0.384
AAANQAVTPAARALP	CAE55371.1; PPE FAMILY PROTEIN	-0.154	-0.384
EEEFKAQFEAAGLTY	CAA17111.1; PROBABLE ISOCITRATE DEHYDROGENASE	-0.186	-0.385
MLPRSPIVRIVDSDT	MYCOBACTERIUM BOVIS ACYL-COA SYNTHASE GENE; U75685	-0.147	-0.385
TLARVAAFIGEHEPS	POSSIBLE GLYCOSYL TRANSFERASE CAB05419	-0.297	-0.386
SHDKTFEIPEDGVAN	PROBABLE ISOCITRATE DEHYDROGENASE CAA16247	-0.263	-0.386
PGIPESFKVLLKELQ	RNA POLYMERASE BETA-SUBUNIT AAA21416	0.241	-0.387
KLTPWKAPPPIAHST	PPE FAMILY PROTEIN CAE55489	0.163	-0.387
AAASLAAIAIAFLAG	CAA98350.1; CELL SURFACE LIPOPROTEIN MPT83	-0.109	-0.388
GNAGTASTGLFNVGG	YP_177963; PPE FAMILY PROTEIN	-0.095	-0.388
LAAAGCGSKPPSGSP	PERIPLASMIC PHOSPHATE-BINDING LIPOPROTEIN PSTS1 YP_177770	-0.148	-0.388
KLGGLWKTVSPHRSP	CAE55371.1; PPE FAMILY PROTEIN	-0.146	-0.389
NDTTAPSSGFFNTGG	YP_177963; PPE FAMILY PROTEIN	-0.132	-0.389
AWGKGAGKSFQGGVG	PERIPLASMIC PHOSPHATE-BINDING LIPOPROTEIN PSTS3 YP_177768	-0.186	-0.389
STGLFNAGGFTTGLA	YP_177963; PPE FAMILY PROTEIN	-0.169	-0.389
GNIGLGNAGSNNVGF	YP_177963; PPE FAMILY PROTEIN	0.001	-0.390
DNTVGTDEALLDIYR	RNA POLYMERASE BETA-SUBUNIT AAA21416	-0.077	-0.390
SGMTSQAATFAANRL	PROBABLE CUTINASE PRECURSOR CFP21 NP_216500	0.091	-0.390
EGTVQAESAGAVGGD	MTB48 AAK31576	0.048	-0.390
QAAGQARLAASAFEA	PPE FAMILY PROTEIN CAE55504	-0.013	-0.391
GNSLKVGNADVVCGG	CAA98373.1; MAJOR SECRETED IMMUNOGENIC PROTEIN	-0.073	-0.391
AYTLARGRRHRPVRT	MYCOBACTERIUM BOVIS MYCOCEROSIC ACID SYNTHASE GENE; M95808	-0.001	-0.391
GQYDSVVSVEMIEAV	PUTATIVE CYCLOPROPANE-FATTY-ACYL-PHOSPHOLIPID SYNTHASE UFAA1	-0.063	-0.392

	NP_854118		
RLVDAPLPAWTHARL	MYCOBACTERIUM BOVIS MYCOCEROSIC ACID SYNTHASE GENE; M95808	0.065	-0.392
RLAASAFEATLAATV	PPE FAMILY PROTEIN CAE55504	-0.108	-0.392
GNFGVFNSGSYNTGV	YP_177963; PPE FAMILY PROTEIN	0.006	-0.392
GNPNRPDGGILTRFG	PPE FAMILY PROTEIN CAE55504	0.010	-0.393
QGELSKQTGQQVSIA	CAA98382.1; IMMUNOGENIC PROTEIN MPT64	-0.222	-0.393
ASLVGFNRAPAGPSG	CAA17582.1; PROBABLE SERINE PROTEASE PEPD	-0.208	-0.393
ITSAGRHPDSDIFLD	CAB01474.1; CONSERVED HYPOTHETICAL PROTEIN CFP17	0.163	-0.393
PGEPPTKESAQTLLE	RNA POLYMERASE BETA-SUBUNIT AAA21416	-0.195	-0.394
VGSAQPIVIPTPIGE	PPE FAMILY PROTEIN CAE55334	-0.442	-0.394
TVTLTFTPADGSAPI	CAA17111.1; PROBABLE ISOCITRATE DEHYDROGENASE	0.161	-0.394
GFANVGDVNLGAGNL	YP_177963; PPE FAMILY PROTEIN	0.068	-0.394
AYAIKKHGVGVKCAT	CAA17111.1; PROBABLE ISOCITRATE DEHYDROGENASE	-0.097	-0.395
GSAVNPVLRQGNSDR	PROBABLE ISOCITRATE DEHYDROGENASE CAA16247	-0.233	-0.395
ALSSSHGLSVADLVL	MYCOBACTERIUM BOVIS ACYL-COA SYNTHASE GENE; U75685	-0.155	-0.395
AERTEQKDFDGRSEF	CAA17245.1; HEAT SHOCK PROTEIN HSPX	-0.083	-0.396
TAALRAAEDDCPPPG	MYCOBACTERIUM BOVIS MYCOCEROSIC ACID SYNTHASE GENE; M95808	-0.080	-0.396
FMQAAIGPGQEGLDQ	PERIPLASMIC PHOSPHATE-BINDING LIPOPROTEIN PSTS2 YP_177769	-0.067	-0.396
LLDEGKQSLTKLAAA	CAE55648	-0.223	-0.396
VRASLERMAPLVDRQ	MTB81 (publication)	-0.062	-0.396
MDPGSKVTYNGVEIG	MCE-FAMILY PROTEIN MCE1AYP_177701	-0.117	-0.397
MPHPQQAPVLVGVDG	HYPOTHETICAL PROTEIN RV2623 NP_217139	-0.166	-0.398
ELAAQTDDQQLAEHF	PROBABLE ISOCITRATE DEHYDROGENASE CAA16247	-0.165	-0.398
RIFTDGDHTLYADVP	POSSIBLE GLYCOSYL TRANSFERASE CAB05415	-0.137	-0.399
SPSGVAVDSAGNVYV	TRANSMEMBRANE SERINE/THREONINE-PROTEIN KINASE D PKND NP_215446	0.312	-0.399
QHLTMANIVTSAGGD	PERIPLASMIC PHOSPHATE-BINDING LIPOPROTEIN PSTS3 YP_177768	-0.094	-0.399
RALAQYLADTLAEEQ	MYCOBACTERIUM BOVIS MYCOCEROSIC ACID SYNTHASE GENE; M95808	-0.220	-0.399
SALQNAASIAGLFLT	60 kDa chaperonin 2 P0A521	-0.117	-0.399
RHDIQQLAALGDVYA	MCE-FAMILY PROTEIN MCE1AYP_177701	-0.209	-0.399
VRAFMQAAIGPGQEG	PERIPLASMIC PHOSPHATE-BINDING LIPOPROTEIN PSTS2 YP_177769	-0.191	-0.400
PAVADHVAAVALFGE	PROBABLE CUTINASE PRECURSOR CFP21 NP_216500	-0.159	-0.400
SIDAPGVVNFTVETN	MYCOBACTERIUM BOVIS MYCOCEROSIC ACID SYNTHASE GENE; M95808	-0.017	-0.401
LPLGTPALVSGLGNV	YP_177963; PPE FAMILY PROTEIN	-0.253	-0.401
ARGTHQASGLGDVGE	PROBABLE CUTINASE PRECURSOR CFP21 NP_216500	-0.173	-0.401
IALAITNTARPWHIG	PPE FAMILY PROTEIN CAE55334	-0.174	-0.403
FEATLAATVSPAMVA	PPE FAMILY PROTEIN CAE55504	-0.066	-0.403
RGKLDGTPEVIDFAH	CAA17111.1; PROBABLE ISOCITRATE DEHYDROGENASE	-0.180	-0.403
GHPLSSATDEPEPHW	PPE FAMILY PROTEIN CAE55334	-0.265	-0.403
LVDINETLNLGPVHI	YP_177963; PPE FAMILY PROTEIN	-0.202	-0.404
ANSGNVDTGALMSGN	YP_177963; PPE FAMILY PROTEIN	-0.149	-0.404
NFQLSQGGQGFAIPI	CAB09453.1; PROBABLE SERINE PROTEASE PEPA	-0.122	-0.405
SDLAYTLARGRRHRP	MYCOBACTERIUM BOVIS MYCOCEROSIC ACID SYNTHASE GENE; M95808	-0.099	-0.405
AMSNLLGQNAAAIAA	YP_177963; PPE FAMILY PROTEIN	-0.252	-0.405
GSAKSNMGHSTASAG	MYCOBACTERIUM BOVIS MYCOCEROSIC ACID SYNTHASE GENE; M95808	-0.099	-0.405
MALASLAARGTTGGG	PPE FAMILY PROTEIN CAE55334	-0.090	-0.405
HWAITDGNKASFLDQ	PERIPLASMIC PHOSPHATE-BINDING LIPOPROTEIN PSTS1 YP_177770	-0.161	-0.405
IGLFNSGTGNIGFFN	YP_177963; PPE FAMILY PROTEIN	-0.083	-0.405
LSKQTGQQVSIAPNA	CAA98382.1; IMMUNOGENIC PROTEIN MPT64	-0.259	-0.406
QTLTLRLLAASAGIL	CONSERVED HYPOTHETICAL PROTEIN CAA15739	-0.193	-0.406
LSVPHSWTTAAPEIQ	PPE FAMILY PROTEIN CAE55334	-0.212	-0.406
LGLTGQANYGFAVPD	YP_177963; PPE FAMILY PROTEIN	-0.114	-0.407
VGTLNSGVLNVGSGI	YP_177963; PPE FAMILY PROTEIN	-0.075	-0.407
ISEAGQAMASTEGNV	CAE55648	-0.199	-0.407
HLVEGVARVLTKPRF	POSSIBLE HEMOLYSIN-LIKE PROTEIN CAA17201	-0.432	-0.408
EEVGLTLENADLSLL	60 kDa chaperonin 2 P0A521	-0.155	-0.408
LRAQVMGDLDKLTGN	CAB06237.1; CONSERVED HYPOTHETICAL PROTEIN	-0.491	-0.409
VITVEESNTFGLQLE	60 kDa chaperonin 2 P0A521	-0.185	-0.409
TIDAIELPAISFSQS	YP_177963; PPE FAMILY PROTEIN	-0.229	-0.410
GFGNIGDANFGLGNA	YP_177963; PPE FAMILY PROTEIN	0.137	-0.410
NAGHTNTGGFNTGNV	YP_177963; PPE FAMILY PROTEIN	0.104	-0.410
CTGGGNIMAHVSYVQ	PROBABLE CUTINASE PRECURSOR CFP21 NP_216500	-0.172	-0.410
SEEGSGIILSAEGLI	CAA17582.1; PROBABLE SERINE PROTEASE PEPD	-0.166	-0.410
PGGSFNTAVASVVAR	CONSERVED HYPOTHETICAL PROTEIN CAA15739	-0.155	-0.410
NAGLVCGGVHTANAT	CAA98350.1; CELL SURFACE LIPOPROTEIN MPT83	-0.115	-0.410
KDQTSDEVTVETTSV	CAB01474.1; CONSERVED HYPOTHETICAL PROTEIN CFP17	-0.138	-0.410

PDRWDADDYYDPEPG	MYCOBACTERIUM BOVIS MYCOCEROSIC ACID SYNTHASE GENE; M95808	-0.261	-0.411
YLQYTSGSTRTPAGV	MYCOBACTERIUM BOVIS ACYL-COA SYNTHASE GENE; U75685	-0.194	-0.411
ATPSSGFFNSGAGGV	YP_177963; PPE FAMILY PROTEIN	-0.260	-0.411
FFNSGAGGVSGFGNF	YP_177963; PPE FAMILY PROTEIN	-0.174	-0.411
VTYNGVEIGRVDTIS	MCE-FAMILY PROTEIN MCE1AYP_177701	-0.076	-0.412
TSTGGIGPIIIPDTT	YP_177963; PPE FAMILY PROTEIN	0.123	-0.412
QIDQVESTAGSLQGQ	CAA17966.1; 10 KDA CULTURE FILTRATE ANTIGEN ESXB	-0.216	-0.413
NALGGNRPAVYAPKS	3-OXOACYL-[ACYL-CARRIER PROTEIN] SYNTHASE 2 KASB CAA94642	-0.125	-0.413
GVAVDSAGNVYVTSE	TRANSMEMBRANE SERINE/THREONINE-PROTEIN KINASE D PKND NP_215446	-0.113	-0.413
SGLYNTAIVGLGTPA	YP_177963; PPE FAMILY PROTEIN	-0.462	-0.413
AADAGFTAPATTLLS	YP_177963; PPE FAMILY PROTEIN	-0.280	-0.414
MAELAPDTVPLAGVV	CAA16030.1; PROBABLE MOLYBDOPTERIN-GUANINE DINUCLEOTIDE BIOSYNTHESIS PROTEIN	-0.498	-0.415
LPTDRPIIYATLGSS	POSSIBLE GLYCOSYL TRANSFERASE CAB05418	-0.340	-0.415
SQESGVIEEVSADYI	RNA POLYMERASE BETA-SUBUNIT AAA21416	-0.139	-0.417
DAATLAQEAGNFERI	CAA17966.1; 10 KDA CULTURE FILTRATE ANTIGEN ESXB	-0.175	-0.417
AINAHLTGNAAMGAF	YP_177963; PPE FAMILY PROTEIN	-0.183	-0.417
AVDGAGTVYVTDFNN	TRANSMEMBRANE SERINE/THREONINE-PROTEIN KINASE D PKND NP_215446	-0.225	-0.417
AAEYGRLRHGQILFT	SECRETED L-ALANINE DEHYDROGENASE ALD CAA15575	-0.229	-0.417
DSVERLIAAHLMDPA	TRANSMEMBRANE SERINE/THREONINE-PROTEIN KINASE D PKND NP_215446	-0.601	-0.417
GGLNSGAGNIGLFNS	YP_177963; PPE FAMILY PROTEIN	-0.144	-0.418
DQQEILNRANEVEAP	MTB48 AAK31576	-0.159	-0.418
GNGQIGFGKPANPDV	PPE FAMILY PROTEIN CAE55504	-0.163	-0.419
LYRRDGSYIITGGLG	MYCOBACTERIUM BOVIS MYCOCEROSIC ACID SYNTHASE GENE; M95808	-0.120	-0.419
VGFGNIGHGNVGFGN	YP_177963; PPE FAMILY PROTEIN	-0.002	-0.419
GCILADSRQSKTAAS	RNA POLYMERASE BETA-SUBUNIT AAA21416	-0.086	-0.419
YIHAVYYPGENQIQT	CONSERVED HYPOTHETICAL PROTEIN TB18.5CAD93033	0.163	-0.419
EFRVAITPAGVAELT	SECRETED L-ALANINE DEHYDROGENASE ALD CAA15575	-0.154	-0.419
NCQSILGYVVRWVDQ	MTB81 (publication)	-0.098	-0.419
GSRGRGGYAGMLVGS	HYPOTHETICAL PROTEIN RV2623 NP_217139	-0.199	-0.420
CVEQYRQDQFARLDA	MYCOBACTERIUM BOVIS ACYL-COA SYNTHASE GENE; U75685	-0.134	-0.420
TAPSRACQGDLGMSH	CAA16102.1; ESAT-6 LIKE PROTEIN ESXQ	-0.088	-0.421
SILGYVVRWVDQGVG	MTB81 (publication)	-0.255	-0.421
IGGGNVGFGNIGDAN	YP_177963; PPE FAMILY PROTEIN	0.038	-0.422
ILTYHVVAGQTSPAN	CAA98373.1; MAJOR SECRETED IMMUNOGENIC PROTEIN	-0.292	-0.422
FNTGVANAGSYNTGS	YP_177963; PPE FAMILY PROTEIN	-0.199	-0.422
LTEIGYLLPEPDDFT	MTB81 (publication)	-0.131	-0.422
DAAICGGVETRIEAV	3-OXOACYL-[ACYL-CARRIER PROTEIN] SYNTHASE 2 KASB CAA94642	-0.201	-0.422
TFDTRLMRLEDEMKE	CAA17245.1; HEAT SHOCK PROTEIN HSPX	-0.105	-0.422
YLILSARDVLAVVSK	CAB01005.1; 10 KDA CHAPERONIN GROES	-0.008	-0.423
RQRLATSLRNAAKAY	MTB48 AAK31576	-0.246	-0.423
ETSQTPSAAIDSDRW	PUTATIVE CYCLOPROPANE-FATTY-ACYL-PHOSPHOLIPID SYNTHASE UFAA1 NP_854118	-0.113	-0.423
GQLTIKAERTEQKDF	CAA17245.1; HEAT SHOCK PROTEIN HSPX	-0.082	-0.424
RQLVQRSEEAQLVVV	HYPOTHETICAL PROTEIN RV2623 NP_217139	-0.150	-0.424
LGSASGFANFGTQLS	YP_177963; PPE FAMILY PROTEIN	-0.181	-0.424
FDETIDKSTDKTLHS	RNA POLYMERASE BETA-SUBUNIT AAA21416	-0.064	-0.424
SVAVKAPGFGDRRKA	60 kDa chaperonin 2 P0A521	-0.071	-0.425
SGQPSIIDAAIAELR	YP_177963; PPE FAMILY PROTEIN	0.193	-0.426
QLQQLVAAGGGPSQL	CAB06237.1; CONSERVED HYPOTHETICAL PROTEIN	-0.332	-0.426
LLGSHVRLTEEPERH	MYCOBACTERIUM BOVIS MYCOCEROSIC ACID SYNTHASE GENE; M95808	-0.167	-0.426
RMAPLVDRQNAGDVA	MTB81 (publication)	0.048	-0.427
NAGSYNTGSFNVGDT	YP_177963; PPE FAMILY PROTEIN	-0.154	-0.428
DTSPKPATSPAAPVT	CAA98350.1; CELL SURFACE LIPOPROTEIN MPT83	-0.327	-0.428
SAAVSYGSVVSTLIV	PPE FAMILY PROTEIN CAE55334	-0.374	-0.428
LGSELTMTDTVGQVV	CAB06500.1; IMMUNOGENIC PROTEIN MPT63	-0.293	-0.428
PGALGENGNGGMVTG	PERIPLASMIC PHOSPHATE-BINDING LIPOPROTEIN PSTS1 YP_177770	-0.188	-0.429
PGGAPGCWQPITRDL	MCE-FAMILY PROTEIN MCE1AYP_177701	-0.154	-0.429
QQVNYNLPGVSEHLK	PERIPLASMIC PHOSPHATE-BINDING LIPOPROTEIN PSTS1 YP_177770	-0.203	-0.430
FGNTGTNNLGIGLTG	YP_177963; PPE FAMILY PROTEIN	-0.152	-0.430
PVTVIAATAGRSDLK	POSSIBLE GLYCOSYL TRANSFERASE CAB05415	-0.243	-0.430
LSGKLNPDVNLVDTL	CAA98350.1; CELL SURFACE LIPOPROTEIN MPT83	-0.245	-0.431
ACGNDDNVTGGGATT	PERIPLASMIC PHOSPHATE-BINDING LIPOPROTEIN PSTS3 YP_177768	-0.118	-0.432
LVMGGTDSLLPLPNI	PPE FAMILY PROTEIN CAE55504	-0.311	-0.432

VVPSVVMLETDLGRQ	CAA17582.1; PROBABLE SERINE PROTEASE PEPD	0.075	-0.434
LDVEMTAVQRSFNRT	PPE FAMILY PROTEIN CAE55489	-0.281	-0.434
SQVGTAVKAFLQSTI	PERIPLASMIC PHOSPHATE-BINDING LIPOPROTEIN PSTS3 YP_177768	-0.296	-0.435
ALTFQDPSGGSRTVQ	CAA17582.1; PROBABLE SERINE PROTEASE PEPD	-0.149	-0.435
MRSERLRWLVAAEGP	CONSERVED HYPOTHETICAL PROTEIN CAB08634	-0.123	-0.436
QLVAAGGGPSQLASM	CAB06237.1; CONSERVED HYPOTHETICAL PROTEIN	-0.321	-0.436
SGGSGQPSIIDAAIA	YP_177963; PPE FAMILY PROTEIN	-0.021	-0.436
ARRFEGRGEPRDDLI	ALTERNATE RNA POLYMERASE SIGMA FACTOR SIGF CAB07069	-0.136	-0.436
DAFPNEFPDPTISVQ	YP_177963; PPE FAMILY PROTEIN	-0.278	-0.436
LVGLAGGAATAGAFS	CAB10044.1; SECRETED ANTIGEN 85-B FBPB (85B)	-0.165	-0.436
AWRNGDWYVARLTPS	MYCOBACTERIUM BOVIS MYCOCEROSIC ACID SYNTHASE GENE; M95808	-0.192	-0.437
AIELPAISFSQSIPI	YP_177963; PPE FAMILY PROTEIN	-0.249	-0.438
NVFATANAIAGILFL	PPE FAMILY PROTEIN CAE55504	-0.098	-0.438
DITAPWGIAVDEAGT	TRANSMEMBRANE SERINE/THREONINE-PROTEIN KINASE D PKND NP_215446	-0.120	-0.438
GNSGLAAGLAGMGNI	YP_177963; PPE FAMILY PROTEIN	0.204	-0.438
LLAMLTSGGSGGSGQ	YP_177963; PPE FAMILY PROTEIN	-0.280	-0.438
EFRQAARRLAEAFGP	POSSIBLE GLYCOSYL TRANSFERASE CAB05418	-0.314	-0.439
EQTPIDAVASIDAPG	MYCOBACTERIUM BOVIS MYCOCEROSIC ACID SYNTHASE GENE; M95808	-0.222	-0.439
NANIGFGNRGDANIG	PPE FAMILY PROTEIN CAE55504	-0.097	-0.439
TAAQAAVVRFQEAAN	CAA17966.1; 10 KDA CULTURE FILTRATE ANTIGEN ESXB	-0.019	-0.439
AISAGDQSIGDLIAE	60 kDa chaperonin 2 P0A521	-0.223	-0.440
DEASRRNVDLVALHA	HYPOTHETICAL PROTEIN RV2623 NP_217139	-0.129	-0.440
TIIYTLTDEAPLLAT	PROBABLE ISOCITRATE DEHYDROGENASE CAA16247	-0.802	-0.441
EHTDVERVAQQLLSG	MYCOBACTERIUM BOVIS MYCOCEROSIC ACID SYNTHASE GENE; M95808	0.162	-0.441
GAGSGFANFGSLGSG	YP_177963; PPE FAMILY PROTEIN	-0.199	-0.441
RATLRISSQLLANWL	MTB81 (publication)	-0.225	-0.441
GIRLVIVLVRRWPKV	POSSIBLE GLYCOSYL TRANSFERASE CAB05419	-0.092	-0.442
YRATNFKVDQPGTVT	CAA17111.1; PROBABLE ISOCITRATE DEHYDROGENASE	-0.169	-0.442
VESGKMTKDLAILIG	CAA17111.1; PROBABLE ISOCITRATE DEHYDROGENASE	0.088	-0.442
QNIAQQLRAQVMGDL	CAB06237.1; CONSERVED HYPOTHETICAL PROTEIN	-0.221	-0.442
AGISEFNGNQTDFGG	PERIPLASMIC PHOSPHATE-BINDING LIPOPROTEIN PSTS3 YP_177768	-0.089	-0.442
GDLNVGLGNVGGGNV	YP_177963; PPE FAMILY PROTEIN	-0.003	-0.443
VSVVASPQSTVIGGT	MYCOBACTERIUM BOVIS MYCOCEROSIC ACID SYNTHASE GENE; M95808	-0.316	-0.443
PVDALVNLADEEKAD	CAB08889.1; IRON-REGULATED CONSERVED HYPOTHETICAL PROTEIN	-0.792	-0.443
GWRAACRSNPALAKG	SECRETED L-ALANINE DEHYDROGENASE ALD CAA15575	-0.194	-0.443
ILKDESYKVTGTAPI	CAB08889.1; IRON-REGULATED CONSERVED HYPOTHETICAL PROTEIN	-0.251	-0.444
GPFFGLGVVNISGIE	YP_177963; PPE FAMILY PROTEIN	0.042	-0.444
PGGLSRERAGLEVRD	RNA POLYMERASE BETA-SUBUNIT AAA21416	-0.156	-0.444
DLQTLEFVASDRVPP	MYCOBACTERIUM BOVIS MYCOCEROSIC ACID SYNTHASE GENE; M95808	-0.160	-0.445
GSTLVDINETLNLGP	YP_177963; PPE FAMILY PROTEIN	-0.178	-0.445
GIGLTGDNQTGIGGL	YP_177963; PPE FAMILY PROTEIN	-0.233	-0.445
ESGTTDNFQRYLQAA	PERIPLASMIC PHOSPHATE-BINDING LIPOPROTEIN PSTS3 YP_177768	-0.191	-0.446
GNGGPGVTALVMGGT	PPE FAMILY PROTEIN CAE55504	-0.146	-0.449
DGSAPIVHEMVSIPE	CAA17111.1; PROBABLE ISOCITRATE DEHYDROGENASE	-0.417	-0.449
YQSAIPPRGTQAVVL	CAA98382.1; IMMUNOGENIC PROTEIN MPT64	-0.244	-0.450
SEFNGNQTDFGGSDV	PERIPLASMIC PHOSPHATE-BINDING LIPOPROTEIN PSTS3 YP_177768	0.090	-0.452
VRSRTDLLSTLPQRV	CONSERVED HYPOTHETICAL PROTEIN CAB08634	-0.257	-0.453
LAAYPITGKLGSELT	CAB06500.1; IMMUNOGENIC PROTEIN MPT63	-0.071	-0.454
TQASGQTVLPFTGID	TRANSMEMBRANE SERINE/THREONINE-PROTEIN KINASE D PKND NP_215446	-0.191	-0.454
AIVADIERYPEWNEG	CONSERVED HYPOTHETICAL PROTEIN TB18.5CAD93033	-0.133	-0.454
GMGEVYEAEDTRKHR	TRANSMEMBRANE SERINE/THREONINE-PROTEIN KINASE D PKND NP_215446	0.001	-0.455
NTNTGIANSGNVNTG	YP_177963; PPE FAMILY PROTEIN	-0.113	-0.455
ERTFGGKIVTPSPGT	MYCOBACTERIUM BOVIS ACYL-COA SYNTHASE GENE; U75685	0.048	-0.455
IPIPDEFKSRLSTAV	PERIPLASMIC PHOSPHATE-BINDING LIPOPROTEIN PSTS3 YP_177768	-0.177	-0.455
TGTNNLGIGLTGDNQ	YP_177963; PPE FAMILY PROTEIN	-0.237	-0.455
IGFGNRGDANIGIGN	PPE FAMILY PROTEIN CAE55504	-0.230	-0.456
NIGFFNSGTANFGLF	YP_177963; PPE FAMILY PROTEIN	-0.109	-0.457
RTVSLPVGADEDDIK	CAA17245.1; HEAT SHOCK PROTEIN HSPX	-0.261	-0.457
LKRGIEKAVEKVTET	60 kDa chaperonin 2 P0A521	-0.195	-0.458
VKAFLQSTIGAGQSG	PERIPLASMIC PHOSPHATE-BINDING LIPOPROTEIN PSTS3 YP_177768	-0.476	-0.458
TGIGNSGVASTGLVN	YP_177963; PPE FAMILY PROTEIN	-0.299	-0.458
KAYGEVDEEAATALD	MTB48 AAK31576	-0.290	-0.458
SGLGNVGDHLSGLLA	YP_177963; PPE FAMILY PROTEIN	-0.464	-0.459

TGVANSGNVDTGALM	YP_177963; PPE FAMILY PROTEIN	-0.184	-0.460
AQQLRAQVMGDLDKL	CAB06237.1; CONSERVED HYPOTHETICAL PROTEIN	-0.412	-0.461
DIVVECGNIAEPDTA	MYCOBACTERIUM BOVIS MYCOCEROSIC ACID SYNTHASE GENE; M95808	-0.064	-0.461
PAIKIDPPPPPQEQG	MTB48 AAK31576	-0.129	-0.463
DKLPAATIDQLKTDA	CAA98350.1; CELL SURFACE LIPOPROTEIN MPT83	-0.360	-0.464
EIPEDGVANIVDVAT	PROBABLE ISOCITRATE DEHYDROGENASE CAA16247	-0.210	-0.464
SLYEADTLARVAAFI	POSSIBLE GLYCOSYL TRANSFERASE CAB05419	-0.200	-0.465
QPLPPAVVKLSDALI	PERIPLASMIC PHOSPHATE-BINDING LIPOPROTEIN PSTS1 YP_177770	-0.225	-0.465
QYGPLTPARAVAIVR	TRANSMEMBRANE SERINE/THREONINE-PROTEIN KINASE D PKND NP_215446	-0.176	-0.465
GMPAGLLSGMALASL	PPE FAMILY PROTEIN CAE55334	-0.330	-0.466
DASGVLDPITIFPGG	YP_177963; PPE FAMILY PROTEIN	-0.305	-0.467
LVLDTDSFYRPKRPG	PERIPLASMIC PHOSPHATE-BINDING LIPOPROTEIN PSTS3 YP_177768	-0.261	-0.468
TGIGGLNSGTGNLGL	YP_177963; PPE FAMILY PROTEIN	-0.246	-0.468
LGDLNLGLGNIGNAN	YP_177963; PPE FAMILY PROTEIN	-0.336	-0.469
VRRGLVDLLGADPEL	TWO COMPONENT TRANSCRIPTIONAL REGULATORY PROTEIN DEVR NP_217649	-0.574	-0.469
FKVLLKELQSLCLNV	RNA POLYMERASE BETA-SUBUNIT AAA21416	-0.242	-0.470
AGDTDAIAGRVAQIR	60 kDa chaperonin 2 P0A521	-0.127	-0.470
IDAAYAIKKHGVGVK	CAA17111.1; PROBABLE ISOCITRATE DEHYDROGENASE	-0.123	-0.470
IHIPSIPLGFAAIGH	YP_177963; PPE FAMILY PROTEIN	-0.255	-0.470
IVMSTNNDDPAGACR	3-OXOACYL-[ACYL-CARRIER PROTEIN] SYNTHASE 2 KASB CAA94642	-0.144	-0.470
GNFLLPDAQSIQAAA	PERIPLASMIC PHOSPHATE-BINDING LIPOPROTEIN PSTS1 YP_177770	-0.109	-0.471
YGAGTPCALGSAKSN	MYCOBACTERIUM BOVIS MYCOCEROSIC ACID SYNTHASE GENE; M95808	-0.131	-0.471
WTHARLFIDDDGQEQ	MYCOBACTERIUM BOVIS MYCOCEROSIC ACID SYNTHASE GENE; M95808	-0.254	-0.471
DALRIVMADSRPLTN	CAA16030.1; PROBABLE MOLYBDOPTERIN-GUANINE DINUCLEOTIDE BIOSYNTHESIS PROTEIN	-0.310	-0.472
TITVHPLLGSHVRLT	MYCOBACTERIUM BOVIS MYCOCEROSIC ACID SYNTHASE GENE; M95808	-0.422	-0.472
LRALLKQYGPLTPAR	TRANSMEMBRANE SERINE/THREONINE-PROTEIN KINASE D PKND NP_215446	-0.300	-0.473
NSPDKLWESLLRGDD	MYCOBACTERIUM BOVIS MYCOCEROSIC ACID SYNTHASE GENE; M95808	-0.205	-0.473
EPMVKMLLNNVLEHL	CONSERVED HYPOTHETICAL PROTEIN TB18.5CAD93033	-0.626	-0.475
YAIVNNRQKDAATAQ	PERIPLASMIC PHOSPHATE-BINDING LIPOPROTEIN PSTS1 YP_177770	-0.278	-0.476
STNVGLANMGVGNIG	YP_177963; PPE FAMILY PROTEIN	-0.019	-0.476
DYYDLGIEHRDATDD	CAA17111.1; PROBABLE ISOCITRATE DEHYDROGENASE	-0.153	-0.479
GSAPAVISGFGNLGN	YP_177963; PPE FAMILY PROTEIN	0.196	-0.480
LRISLSVSARLAGIP	POSSIBLE GLYCOSYL TRANSFERASE CAB05415	-0.481	-0.481
KTTQTIYIDADPGEV	CONSERVED HYPOTHETICAL PROTEIN TB16.3CAD97060	-0.408	-0.483
SGLGALPGIGNIGLG	YP_177963; PPE FAMILY PROTEIN	-0.271	-0.486
ALAAAGVQPETVGVV	MYCOBACTERIUM BOVIS MYCOCEROSIC ACID SYNTHASE GENE; M95808	-0.083	-0.486
ASRMPVLSKTVEVTA	CONSERVED HYPOTHETICAL PROTEIN TB18.5CAD93033	-0.235	-0.486
VGLGNVNGVTLGYTS	LIPOPROTEIN LPQH PRECURSOR P0A5J0	-0.385	-0.487
FVALPAATADAYATT	CAE55335.1; PE FAMILY PROTEIN	-0.150	-0.488
KLGLHVGEPITSSTL	RNA POLYMERASE BETA-SUBUNIT AAA21416	-0.301	-0.489
TGSNNIGIGLTGDNL	YP_177963; PPE FAMILY PROTEIN	-0.164	-0.489
KTVEAEAAHGTVTRH	CAA17111.1; PROBABLE ISOCITRATE DEHYDROGENASE	-0.182	-0.489
RGLERGLNALADAVK	60 kDa chaperonin 2 P0A521	-0.584	-0.489
LNSGVLNVGSGISGL	YP_177963; PPE FAMILY PROTEIN	-0.134	-0.490
GKTIAGAKIMGQGND	PERIPLASMIC PHOSPHATE-BINDING LIPOPROTEIN PSTS2 YP_177769	-0.150	-0.490
LLASASIGTLGFTIP	YP_177963; PPE FAMILY PROTEIN	-0.257	-0.491
DRLVSAATATGLPLR	MYCOBACTERIUM BOVIS MYCOCEROSIC ACID SYNTHASE GENE; M95808	-0.264	-0.493
TGVLPPAANDVSVLT	CAE55335.1; PE FAMILY PROTEIN	-0.257	-0.495
NTIVDKEFLKRLPMS	POSSIBLE GLYCOSYL TRANSFERASE CAB05419	-0.239	-0.496
GEVDEEAATALDNDG	MTB48 AAK31576	-0.053	-0.498
PRWEADLDVLDEHGT	MYCOBACTERIUM BOVIS MYCOCEROSIC ACID SYNTHASE GENE; M95808	-0.195	-0.498
ELLPEAAGPTQVLVP	CAA98382.1; IMMUNOGENIC PROTEIN MPT64	-0.285	-0.498
QLVASQSAFAAKAGL	CONSERVED HYPOTHETICAL PROTEIN TB9.8 CAD93159	-0.248	-0.500
LRIARVLYDFVNNEA	MTB81	-0.343	-0.500
ASGFANFGTQLSGIL	YP_177963; PPE FAMILY PROTEIN	-0.276	-0.501
GIPDPGDIFNTGSSL	CAB06237.1; CONSERVED HYPOTHETICAL PROTEIN	-0.195	-0.501
PQSTVIGGTSDTVRD	MYCOBACTERIUM BOVIS MYCOCEROSIC ACID SYNTHASE GENE; M95808	-0.210	-0.502
DLADRVDTLVGAGER	CAA16030.1; PROBABLE MOLYBDOPTERIN-GUANINE DINUCLEOTIDE BIOSYNTHESIS PROTEIN	-0.279	-0.504
DLSQRLGRAPSASEL	ALTERNATE RNA POLYMERASE SIGMA FACTOR SIGF CAB07069	-0.054	-0.506
SIGLGFAIPVDQAKR	CAA17582.1; PROBABLE SERINE PROTEASE PEPD	-0.274	-0.508
SDIAVVRVQGVSGLT	CAA17582.1; PROBABLE SERINE PROTEASE PEPD	-0.595	-0.509
FLLDQAITSAGRHPD	CAB01474.1; CONSERVED HYPOTHETICAL PROTEIN CFP17	-0.771	-0.509

DSLTGAEETLNGLIQ	CAB09453.1; PROBABLE SERINE PROTEASE PEPA	-0.322	-0.513
GSLGSGVLNFGSGVS	YP_177963; PPE FAMILY PROTEIN	-0.223	-0.513
AGYAGTLQSLGADIA	CAA16104.1; SECRETED ESAT-6 LIKE PROTEIN ESXR	-0.267	-0.530
VPDIPIPAIHIDFDG	YP_177963; PPE FAMILY PROTEIN	-0.329	-0.545
LGAANIGDLNVGLGN	YP_177963; PPE FAMILY PROTEIN	-0.464	-0.547
LGGGVAANLGRAASV	CAE55371.1; PPE FAMILY PROTEIN	-0.397	-0.548
LAGAGSGIANVGTLH	YP_177963; PPE FAMILY PROTEIN	-0.238	-0.553
ATLAQLPPGILSDIL	PPE FAMILY PROTEIN CAE55334	-0.830	-0.553
ISGFGNLGNHVSGVS	YP_177963; PPE FAMILY PROTEIN	-0.186	-0.558
EFLDAIADNLEKELA	CAA17111.1; PROBABLE ISOCITRATE DEHYDROGENASE	-0.338	-0.558
LGAGFEDIGIKTGNE	PROBABLE ISOCITRATE DEHYDROGENASE CAA16247	-0.114	-0.570
QSIGDLIAEAMDKVG	60 kDa chaperonin 2 P0A521	-0.375	-0.574
VGEQLSGLSSAGTAL	YP_177963; PPE FAMILY PROTEIN	-0.240	-0.590
VEIDLCDYRDVDGQY	PUTATIVE CYCLOPROPANE-FATTY-ACYL-PHOSPHOLIPID SYNTHASE UFAA1 NP_854118	-0.312	-0.598
IKDLAGNVTLQLGNL	YP_177963; PPE FAMILY PROTEIN	-0.242	-0.598
RPGDLQTLEFVASDR	MYCOBACTERIUM BOVIS MYCOCEROSIC ACID SYNTHASE GENE; M95808	-0.330	-0.608
TGDIGPATIIPPIHI	YP_177963; PPE FAMILY PROTEIN	-0.477	-0.610
GIASTLNPQVGSAQP	PPE FAMILY PROTEIN CAE55334	-0.195	-0.611
IKTGNERAKLLGKTL	PROBABLE ISOCITRATE DEHYDROGENASE CAA16247	-0.708	-0.628
GNVNTGAFISGNFSN	YP_177963; PPE FAMILY PROTEIN	-0.316	-0.634
TIPTGPIHIGLPLSL	YP_177963; PPE FAMILY PROTEIN	-0.622	-0.643
PLLEKVAKEAADEAK	CAB07109.1; PROBABLE 50S RIBOSOMAL PROTEIN L7/L12 RPLL	-1.083	-0.647
LDELKLEGDEATGAN	60 kDa chaperonin 2 P0A521	-0.391	-0.655
VAAAVKQVLSGAEFR	POSSIBLE GLYCOSYL TRANSFERASE CAB05418	-0.606	-0.679
TIDQLKTDAKLLSSI	CAA98350.1; CELL SURFACE LIPOPROTEIN MPT83	-0.414	-0.687
NLAELGRLTQLPDTN	PROBABLE ISOCITRATE DEHYDROGENASE CAA16247	-0.627	-0.693
HVSGVSIDGLLAMLT	YP_177963; PPE FAMILY PROTEIN	-0.429	-0.705
AQLPPGILSDILSAL	PPE FAMILY PROTEIN CAE55334	-0.747	-0.720
TSTLPLGTPALVSGL	YP_177963; PPE FAMILY PROTEIN	-0.668	-0.736
LAVAANLAGALPGIV	MCE-FAMILY PROTEIN MCE1AYP_177701	-0.270	-0.751
PAIQALNRDFTLPGE	PERIPLASMIC PHOSPHATE-BINDING LIPOPROTEIN PSTS3 YP_177768	-0.587	-0.783
VASHSPQVDPILDDL	MYCOBACTERIUM BOVIS MYCOCEROSIC ACID SYNTHASE GENE; M95808	-0.834	-0.804
AELDSALLAAAGFGN	MCE-FAMILY PROTEIN MCE1AYP_177701	-0.634	-0.859
KTLDAAIGKLLDNDK	PROBABLE ISOCITRATE DEHYDROGENASE CAA16247	-0.515	-0.916
ATVALTFQDPSGGSR	CAA17582.1; PROBABLE SERINE PROTEASE PEPD	-0.628	-0.921
EKTVKNYVSRLLAKL	TWO COMPONENT TRANSCRIPTIONAL REGULATORY PROTEIN DEVR NP_217649	-0.894	-0.988

Supplementary Table 6b. List of peptides only recognized by IgG and not by IgA

Peptides	Protein	Average Index
NYPAMMAHAGDMAGY	CAA16104.1; SECRETED ESAT-6 LIKE PROTEIN ESXR	3,357498292
MGCRLPGGINSPDKL	MYCOBACTERIUM BOVIS MYCOCEROSIC ACID SYNTHASE GENE; M95808	3,26634584
MNNLALWSRPVWDVE	CAA17343.1; HEAT SHOCK PROTEIN HSP	2,562033013
EMWAQDAAVMYSYEG	PPE FAMILY PROTEIN CAE55334	2,222215455
DMVAACLKWEGGYVW	CAA17111.1; PROBABLE ISOCITRATE DEHYDROGENASE	2,218562879
WSDVDVSEWPGIDWP	HYPOTHETICAL PROTEIN RV2623 NP_217139	2,053792971
WDINTPAFEWYYQSG	CAB10044.1; SECRETED ANTIGEN 85-B FBPB (85B)	2,007198631
WLADLVRRSPWGEMF	MYCOBACTERIUM BOVIS MYCOCEROSIC ACID SYNTHASE GENE; M95808	2,005375219
PKLVANNTRLWVYCG	CAB10044.1; SECRETED ANTIGEN 85-B FBPB (85B)	1,964679335
WNQALEDLVRAYQSM	CAA16104.1; SECRETED ESAT-6 LIKE PROTEIN ESXR	1,8281652
GELDNRGSQFYLAMY	PROBABLE ISOCITRATE DEHYDROGENASE CAA16247	1,750472915
LTAANTKKVAMAAPM	POSSIBLE GLYCOSYL TRANSFERASE CAB05419	1,708539359
AFDLDRLLFKRNICH	POSSIBLE GLYCOSYL TRANSFERASE CAB05419	1,671740675
RFGEMECWAMQAYGA	RNA POLYMERASE BETA-SUBUNIT AAA21416	1,66637506
AVQRSFNRTLLSLMD	PPE FAMILY PROTEIN CAE55489	1,606072581
SLGWDLCRIFTDGDY	POSSIBLE GLYCOSYL TRANSFERASE CAB05418	1,588060524
LKMCWPAAPRSVGVP	POSSIBLE HEMOLYSIN-LIKE PROTEIN CAA17201	1,540856346
QDWQAQWNQAMEALA	CAA16102.1; ESAT-6 LIKE PROTEIN ESXQ	1,51236425
RSLHWLRPITPTFRP	PUTATIVE CYCLOPROPANE-FATTY-ACYL-PHOSPHOLIPID	1,505408928

Peptides	Protein	Average Index
	SYNTHASE UFAA1 NP_854118	
AQYMREHDIGALPIC	HYPOTHETICAL PROTEIN RV2626C NP_217142	1,480169913
TRLMRLEDEMKEGRY	CAA17245.1; HEAT SHOCK PROTEIN HSPX	1,468845376
GGGGMGMPMGAAHQG	MTB48 AAK31576	1,453001213
EGGYVWACKNYDGDV	CAA17111.1; PROBABLE ISOCITRATE DEHYDROGENASE	1,4405722
VMPVGGQSSFYSDWY	CAB10044.1; SECRETED ANTIGEN 85-B FBPB (85B)	1,43745771
QTYSQQFDWRYPPSP	CAA17582.1; PROBABLE SERINE PROTEASE PEPD	1,433031983
FSNGVLWRGDYEGLW	YP_177963; PPE FAMILY PROTEIN	1,430193096
YPYVVPLIDLEMRRP	CONSERVED HYPOTHETICAL PROTEIN CAB08634	1,403330528
KGLEKIPLLGHLWSW	CAB06237.1; CONSERVED HYPOTHETICAL PROTEIN	1,376440711
LKWEGGYVWACKNYD	CAA17111.1; PROBABLE ISOCITRATE DEHYDROGENASE	1,375228971
GLRGLLRVISSEHPM	MYCOBACTERIUM BOVIS MYCOCEROSIC ACID SYNTHASE GENE; M95808	1,362188474
SEWPGIDWPATQSMA	HYPOTHETICAL PROTEIN RV2623 NP_217139	1,299074225
YYLDLALMSVTQPDR	MYCOBACTERIUM BOVIS MYCOCEROSIC ACID SYNTHASE GENE; M95808	1,272307009
QQIPKLVANNTRLWV	CAB10044.1; SECRETED ANTIGEN 85-B FBPB (85B)	1,265963324
VVSKPAALVHGMFVA	CAE55335.1; PE FAMILY PROTEIN	1,248651058
LRPASCRFVPTCSQY	POSSIBLE HEMOLYSIN CAA16235	1,213761468
TKNTILKAYDGMFKD	CAA17111.1; PROBABLE ISOCITRATE DEHYDROGENASE	1,206367428
TTFGYHEFFHACTAV	POSSIBLE HEMOLYSIN-LIKE PROTEIN CAA17201	1,186669796
WQGDTGITYQGWQTQ	CAA16104.1; SECRETED ESAT-6 LIKE PROTEIN ESXR	1,171808961
YCGKYKRVRFKGIIC	RNA POLYMERASE BETA'-SUBUNIT AAA21417	1,171792063
MANNHMSMTNSGVSM	CAE55371.1; PPE FAMILY PROTEIN	1,16782544
FYLAMYWAQELAAQT	PROBABLE ISOCITRATE DEHYDROGENASE CAA16247	1,098541134
HQAIVRDVLAAGDFW	PUTATIVE ESAT-6 LIKE PROTEIN ESXN YP_177838	1,081287603
MAQQAEEYGSHDKTF	PROBABLE ISOCITRATE DEHYDROGENASE CAA16247	1,069689239
YRLERPLLFALQCMP	POSSIBLE GLYCOSYL TRANSFERASE CAB05415	1,028215409
MSKKALCDFYEEQMQ	PROBABLE ISOCITRATE DEHYDROGENASE CAA16247	1,015397967
GMAKNPAERFMSAGD	TRANSMEMBRANE SERINE/THREONINE-PROTEIN KINASE D PKND NP_215446	1,010234531
NMREYLAAGAKERQR	MTB48 AAK31576	1,000968051
VPSVTIPALTAARAV	YP_177963; PPE FAMILY PROTEIN	0,988313348
PIAVPSVTIPALTAA	YP_177963; PPE FAMILY PROTEIN	0,983977029
PNSALVSWLPFYHDM	MYCOBACTERIUM BOVIS ACYL-COA SYNTHASE GENE; U75685	0,970511955
SGNFSNGILWRGNFE	YP_177963; PPE FAMILY PROTEIN	0,956473172
RMRKFARSNHGTCAN	RNA POLYMERASE BETA-SUBUNIT AAA21416	0,949330455
INYRTLKPEKDGLFC	RNA POLYMERASE BETA'-SUBUNIT AAA21417	0,89783364
WQGDTGITYQAWQAQ	CAA17363.1; LOW MOLECULAR WEIGHT PROTEIN ANTIGEN 7 ESXH	0,864149273
GGVHTANATVYMIDT	CAA98350.1; CELL SURFACE LIPOPROTEIN MPT83	0,86062945
QSAWQGDTGITYQAW	CAA17363.1; LOW MOLECULAR WEIGHT PROTEIN ANTIGEN 7 ESXH	0,853230392
SRTGSIYIVKPKMHG	MTB81	0,851057246
SGSSSGFKVRPSFSF	YP_177963; PPE FAMILY PROTEIN	0,845312037
VVLGMYNFKESIRDF	CAA17111.1; PROBABLE ISOCITRATE DEHYDROGENASE	0,838356034
DTGITYQAWQAQWNQ	CAA17363.1; LOW MOLECULAR WEIGHT PROTEIN ANTIGEN 7 ESXH	0,837086074
GGQRFGEMECWAMQA	RNA POLYMERASE BETA-SUBUNIT AAA21416	0,832210895
EPVARRQRILFVGEA	POSSIBLE GLYCOSYL TRANSFERASE CAB05418	0,822173608
AFISGNFSNGVLWRG	YP_177963; PPE FAMILY PROTEIN	0,794657787
PEFSELFAAFPSFAG	CAA17245.1; HEAT SHOCK PROTEIN HSPX	0,781468818
RQRVAAAGFGHRVEI	PUTATIVE CYCLOPROPANE-FATTY-ACYL-PHOSPHOLIPID SYNTHASE UFAA1 NP_854118	0,780106244
AVLDPDHDGMRVQVR	MYCOBACTERIUM BOVIS MYCOCEROSIC ACID SYNTHASE GENE; M95808	0,772117409
VTIPALTAARAVLDM	YP_177963; PPE FAMILY PROTEIN	0,753253794
FTLPGRSLMFVRNVG	MTB81 (publication)	0,747045608
TEGLDIQIMSQVRSM	PROBABLE ISOCITRATE DEHYDROGENASE CAA16247	0,742036585
NGQFFVEMRMIDGTS	TRANSMEMBRANE SERINE/THREONINE-PROTEIN KINASE D PKND NP_215446	0,7379895
PWDGRNHYLAAVYRT	CAA16030.1; PROBABLE MOLYBDOPTERIN-GUANINE DINUCLEOTIDE BIOSYNTHESIS PROTEIN	0,7306786
LSDYPYVVPLIDLEM	CONSERVED HYPOTHETICAL PROTEIN CAB08634	0,729554033
ITYDTLWQADTDPLP	CAA98382.1; IMMUNOGENIC PROTEIN MPT64	0,726597459
SSAWQGDTGITYQGW	CAA16104.1; SECRETED ESAT-6 LIKE PROTEIN ESXR	0,724510446
FARMWELYLAYSEAG	PUTATIVE CYCLOPROPANE-FATTY-ACYL-PHOSPHOLIPID SYNTHASE UFAA1 NP_854118	0,711716681

Peptides	Protein	Average Index
MIDGTSLRALLKQYG	TRANSMEMBRANE SERINE/THREONINE-PROTEIN KINASE D PKND NP_215446	0,711622953
SPAMVAANRTRLASL	PPE FAMILY PROTEIN CAE55504	0,704835942
KYKRVRFKGIICERC	RNA POLYMERASE BETA'-SUBUNIT AAA21417	0,6861387
QAYRKPITYDTLWQA	CAA98382.1; IMMUNOGENIC PROTEIN MPT64	0,669819446
FGTSQSATIATFEMR	PPE FAMILY PROTEIN CAE55504	0,639239593
MVQTKRYAGLTAANT	POSSIBLE GLYCOSYL TRANSFERASE CAB05419	0,633077817
PPSLVAANRSRLMSL	PPE FAMILY PROTEIN CAE55334	0,612606852
VIGMGCRLPGGINSP	MYCOBACTERIUM BOVIS MYCOCEROSIC ACID SYNTHASE GENE; M95808	0,595728291
AGAGTGPPTPGAARW	TRANSMEMBRANE SERINE/THREONINE-PROTEIN KINASE D PKND NP_215446	0,595358349
FGGGTAIPISVGPIT	YP_177963; PPE FAMILY PROTEIN	0,59274134
AAMYQGTIKTWDDPQ	PERIPLASMIC PHOSPHATE-BINDING LIPOPROTEIN PSTS1 YP_177770	0,592656519
QRVAVRVSQLHAGPR	CONSERVED HYPOTHETICAL PROTEIN CAB08634	0,584962702
ECKDKDMTYAAPLFV	RNA POLYMERASE BETA-SUBUNIT AAA21416	0,581746056
NGMSRDMASTFTIVA	CAB10947.1; PROBABLE LIPOPROTEIN LPRJ	0,580865314
RIHTRYSSAYELEGA	SECRETED L-ALANINE DEHYDROGENASE ALD CAA15575	0,576214642
EGKQSLTKLAAAWGG	CAE55648	0,569384157
AFAAPAQANPVDDAF	CONSERVED HYPOTHETICAL PROTEIN CAA15739	0,567080825
AATIKRFADRFARFN	MYCOBACTERIUM BOVIS ACYL-COA SYNTHASE GENE; U75685	0,56130746
VYVTSEGMYGRVVKL	TRANSMEMBRANE SERINE/THREONINE-PROTEIN KINASE D PKND NP_215446	0,535838967
LLEQAAAVEEASDTA	CAE55371.1; PPE FAMILY PROTEIN	0,534029413
AIAELRHLNPLNIVN	YP_177963; PPE FAMILY PROTEIN	0,533275689
GMWTMTELMADMVET	MTB81 (publication)	0,507311465
ERCGVEVTRAKVRRE	RNA POLYMERASE BETA'-SUBUNIT AAA21417	0,503148776
GGATSLISYMLPRSP	MYCOBACTERIUM BOVIS ACYL-COA SYNTHASE GENE; U75685	0,502526455
EYELMWAADVAAMAG	YP_177963; PPE FAMILY PROTEIN	0,50059474
WCAHSGWKVDAAKGV	RNA POLYMERASE BETA-SUBUNIT AAA21416	0,496960479
TELAAAQRRKIDRLL	PUTATIVE CYCLOPROPANE-FATTY-ACYL-PHOSPHOLIPID SYNTHASE UFAA1 NP_854118	0,49503797
DDLAAALADIAPMTP	MYCOBACTERIUM BOVIS MYCOCEROSIC ACID SYNTHASE GENE; M95808	0,494057505
EYLAAGAKERQRLAT	MTB48 AAK31576	0,487664725
AYETAYGLTVPPPVI	CAE55371.1; PPE FAMILY PROTEIN	0,48236027
GLAARRRRILFVAEA	POSSIBLE GLYCOSYL TRANSFERASE CAB05415	0,482250977
GSWLTMIRLAKCGPW	POSSIBLE HEMOLYSIN CAA16235	0,481869898
MASTFTIVAIGTYCP	CAB10947.1; PROBABLE LIPOPROTEIN LPRJ	0,480224657
RLARQRVAAAGFGHR	PUTATIVE CYCLOPROPANE-FATTY-ACYL-PHOSPHOLIPID SYNTHASE UFAA1 NP_854118	0,473056479
RRERMGHIELAAPVT	RNA POLYMERASE BETA'-SUBUNIT AAA21417	0,472974122
GLSSLGWDLCRIFTD	POSSIBLE GLYCOSYL TRANSFERASE CAB05418	0,472443167
LLPDAQSIQAAAAGF	PERIPLASMIC PHOSPHATE-BINDING LIPOPROTEIN PSTS1 YP_177770	0,464600604
EAVPIAGFAQMRIVM	3-OXOACYL-[ACYL-CARRIER PROTEIN] SYNTHASE 2 KASB CAA94642	0,464399457
LPGLPSLPDIIDGLW	CAB06237.1; CONSERVED HYPOTHETICAL PROTEIN	0,461585425
CGKAGCQTYKWETFL	CAB10044.1; SECRETED ANTIGEN 85-B FBPB (85B)	0,460006499
DRMFLAEKTVKNYVS	TWO COMPONENT TRANSCRIPTIONAL REGULATORY PROTEIN DEVR NP_217649	0,451972015
AQDAAVMYSYEGASA	PPE FAMILY PROTEIN CAE55334	0,445702162
IIDGLWPPKLPDIPI	CAB06237.1; CONSERVED HYPOTHETICAL PROTEIN	0,435440298
RVICRRSKLMTRIAG	MYCOBACTERIUM BOVIS MYCOCEROSIC ACID SYNTHASE GENE; M95808	0,419623385
GTGPPTPGAARWSPG	TRANSMEMBRANE SERINE/THREONINE-PROTEIN KINASE D PKND NP_215446	0,41092685
ETGSTLLYPLFNLWG	PERIPLASMIC PHOSPHATE-BINDING LIPOPROTEIN PSTS1 YP_177770	0,40093857
KNPAERFMSAGDLAI	TRANSMEMBRANE SERINE/THREONINE-PROTEIN KINASE D PKND NP_215446	0,390432193
GSGQTYGVDVVGYDR	CAB09453.1; PROBABLE SERINE PROTEASE PEPA	0,38495356
SQYAVDALTEYGLLR	POSSIBLE HEMOLYSIN CAA16235	0,383266246
DPDSFWAGVDKVVAD	MTB81 (publication)	0,361767223

Peptides	Protein	Average Index
AAAAYETAYGLTVPP	CAE55371.1; PPE FAMILY PROTEIN	0,357891451
SAIQGNVTSIHSLLD	CAE55648	0,352664166
VYADAAPDLFDFLDS	MCE-FAMILY PROTEIN MCE1AYP_177701	0,350836735
TAASNNPMLSTLTSA	CAA98350.1; CELL SURFACE LIPOPROTEIN MPT83	0,344527157
SEFDVILEAAGDKKI	CAB07109.1; PROBABLE 50S RIBOSOMAL PROTEIN L7/L12 RPLL	0,343812792
TATAGELARDSIYYV	HYPOTHETICAL PROTEIN RV2626C NP_217142	0,342053367
IKRFADRFARFNLQE	MYCOBACTERIUM BOVIS ACYL-COA SYNTHASE GENE; U75685	0,332854806
LDEEEIWDLTSAEFT	CONSERVED HYPOTHETICAL PROTEIN CAB08634	0,331245012
GKAFPYVVVTGIAMT	3-OXOACYL-[ACYL-CARRIER PROTEIN] SYNTHASE 2 KASB CAA94642	0,323320449
ELTMTDTVGQVVLGW	CAB06500.1; IMMUNOGENIC PROTEIN MPT63	0,322988589
PPTPGAARWSPGDSA	TRANSMEMBRANE SERINE/THREONINE-PROTEIN KINASE D PKND NP_215446	0,315088634
IKEFFGTSQLSQFMD	RNA POLYMERASE BETA-SUBUNIT AAA21416	0,314683832
MAKVNIKPLEDKILV	CAB01005.1; 10 KDA CHAPERONIN GROES	0,310450721
IAAAAKPPLGSPPPK	CAA17582.1; PROBABLE SERINE PROTEASE PEPD	0,309443137
HGMFVALPAATADAY	CAE55335.1; PE FAMILY PROTEIN	0,302314498
MMGNAFLTALTNAGI	CAB10947.1; PROBABLE LIPOPROTEIN LPRJ	0,30172851
NGIELCRDLLSRMPD	TWO COMPONENT TRANSCRIPTIONAL REGULATORY PROTEIN DEVR NP_217649	0,301209095
APAIAAAEAEYEQIW	PPE FAMILY PROTEIN CAE55504	0,298781285
RRSGSSRAPTESTRR	MYCOBACTERIUM BOVIS MYCOCEROSIC ACID SYNTHASE GENE; M95808	0,293576286
SGVTPGTGMPAAPMV	MTB48 AAK31576	0,290134627
QHTTEEAIRMNCRAV	CONSERVED HYPOTHETICAL PROTEIN CAB08634	0,283404228
QVVAALNATDPGAAA	LOW MOLECULAR WEIGHT T-CELL ANTIGEN TB8.4 NP_215690	0,275613704
NHVIAGATDINAFSV	CAB09453.1; PROBABLE SERINE PROTEASE PEPA	0,273918174
ENYIAQTRDKFLSAA	CAA98382.1; IMMUNOGENIC PROTEIN MPT64	0,271710266
IAGATDINAFSVGSG	CAB09453.1; PROBABLE SERINE PROTEASE PEPA	0,265587341
AGAATEVELKERKHR	60 kDa chaperonin 2 P0A521	0,2637488
VEAEYELMWAADVAA	YP_177963; PPE FAMILY PROTEIN	0,262071031
PGYPVAGQVWEATAT	CAB06500.1; IMMUNOGENIC PROTEIN MPT63	0,251914991
KMSALVDASDALRIV	CAA16030.1; PROBABLE MOLYBDOPTERIN-GUANINE DINUCLEOTIDE BIOSYNTHESIS PROTEIN	0,251593228
LYTEDRAWTEAVIGN	MTB48 AAK31576	0,238518101
AFAMTVPPSLVAANR	PPE FAMILY PROTEIN CAE55334	0,234180297
VAASAQGMLSSTGRC	MYCOBACTERIUM BOVIS MYCOCEROSIC ACID SYNTHASE GENE; M95808	0,232518259
TTLALVSAPAGGRAA	PROBABLE CUTINASE PRECURSOR CFP21 NP_216500	0,231089257
VIKAGAATEVELKER	60 kDa chaperonin 2 P0A521	0,227026684
GQMLGGLPVGQMGAR	CAE55371.1; PPE FAMILY PROTEIN	0,226301828
WDLCRIFTDGDYTLY	POSSIBLE GLYCOSYL TRANSFERASE CAB05418	0,225658628
GNNMAQTDSAVGSSW	PUTATIVE ESAT-6 LIKE PROTEIN ESXN YP_177838	0,223051183
VDSAGNVYVTSEGMY	TRANSMEMBRANE SERINE/THREONINE-PROTEIN KINASE D PKND NP_215446	0,221641764
DFTPAAEIVKDGDDA	CAA17343.1; HEAT SHOCK PROTEIN HSP	0,22128926
AAAAGFASKTPANQA	PERIPLASMIC PHOSPHATE-BINDING LIPOPROTEIN PSTS1 YP_177770	0,220408085
TQTGTAVGTYNYMAP	TRANSMEMBRANE SERINE/THREONINE-PROTEIN KINASE D PKND NP_215446	0,217518017
TDAKLLSSILTYHVI	CAA98350.1; CELL SURFACE LIPOPROTEIN MPT83	0,217074823
STGMSGGFVTAPTQG	YP_177963; PPE FAMILY PROTEIN	0,216550228
RFVPTCSQYAVDALT	POSSIBLE HEMOLYSIN CAA16235	0,212820518
GLFSYSYSLDVPRIT	YP_177963; PPE FAMILY PROTEIN	0,209620102
KEDEYPSTAYLQYTS	MYCOBACTERIUM BOVIS ACYL-COA SYNTHASE GENE; U75685	0,208506266
YGRLRHGQILFTFLH	SECRETED L-ALANINE DEHYDROGENASE ALD CAA15575	0,20539649
AGCQTYKWETFLTSE	CAB10044.1; SECRETED ANTIGEN 85-B FBPB (85B)	0,201704797
AALQSAWQGDTGITY	CAA17363.1; LOW MOLECULAR WEIGHT PROTEIN ANTIGEN 7 ESXH	0,198940985
QAVPGAAQYIGLVES	LOW MOLECULAR WEIGHT T-CELL ANTIGEN TB8.4 NP_215690	0,198803055
ENPSARDQILPVYAE	MTB48 AAK31576	0,193151716
AAGAKERQRLATSLR	MTB48 AAK31576	0,193050363
VVLPFTDITAPWGIA	TRANSMEMBRANE SERINE/THREONINE-PROTEIN KINASE D PKND NP_215446	0,192738109
NYEGLFSYSYSLDVP	YP_177963; PPE FAMILY PROTEIN	0,19230008
RRTQAAVFATELKRS	TWO COMPONENT TRANSCRIPTIONAL REGULATORY PROTEIN	0,190063453

Peptides	Protein	Average Index
	DEVR NP_217649	
LDAPNGYTFKEDEYP	MYCOBACTERIUM BOVIS ACYL-COA SYNTHASE GENE; U75685	0,182877614
RTLKPEKDGLFCEKI	RNA POLYMERASE BETA'-SUBUNIT AAA21417	0,180402548
WTSSGAIGPTWYSVG	YP_177963; PPE FAMILY PROTEIN	0,175840254
ETIQEITRGRCAAIS	MYCOBACTERIUM BOVIS ACYL-COA SYNTHASE GENE; U75685	0,175378538
KTQTVLPFTGLNDPD	TRANSMEMBRANE SERINE/THREONINE-PROTEIN KINASE D PKND NP_215446	0,172994436
DTPRVATAGEPNFMD	MTB48 AAK31576	0,172414333
LVDADGKAMLFDGRS	RNA POLYMERASE BETA-SUBUNIT AAA21416	0,171218907
GAAFGGLTTAHTPRR	MYCOBACTERIUM BOVIS MYCOCEROSIC ACID SYNTHASE GENE; M95808	0,168453397
EDMPFLADGTPVDII	RNA POLYMERASE BETA-SUBUNIT AAA21416	0,166228421
RPSFSFFAVGPDGMP	YP_177963; PPE FAMILY PROTEIN	0,16567856
YRQYQAGKPTSTNPI	CAA17111.1; PROBABLE ISOCITRATE DEHYDROGENASE	0,160089961
SARDQILPVYAEYQQ	MTB48 AAK31576	0,159044559
VNADIKATTVFGGKY	MCE-FAMILY PROTEIN MCE1AYP_177701	0,155823513
AYVITSVDEEMRHNE	RNA POLYMERASE BETA'-SUBUNIT AAA21417	0,155327059
DQEYDDFWDEDGEVM	PPE FAMILY PROTEIN CAE55489	0,155321
WQADTDPLPVVFPIV	CAA98382.1; IMMUNOGENIC PROTEIN MPT64	0,148995939
VAAMFGYHSAASAVA	PPE FAMILY PROTEIN CAE55504	0,148315995
SIQAAAAGFASKTPA	PERIPLASMIC PHOSPHATE-BINDING LIPOPROTEIN PSTS1 YP_177770	0,147378265
TLGLHGPAMTFDTAC	MYCOBACTERIUM BOVIS MYCOCEROSIC ACID SYNTHASE GENE; M95808	0,140466519
DVTVSRRHAEFRLEN	CAB01474.1; CONSERVED HYPOTHETICAL PROTEIN CFP17	0,136731381
TVFMGDFPMMTEKGT	RNA POLYMERASE BETA-SUBUNIT AAA21416	0,135808968
SSGFKVRPSFSFFAV	YP_177963; PPE FAMILY PROTEIN	0,131811491
ISYMLPRSPIVRIVD	MYCOBACTERIUM BOVIS ACYL-COA SYNTHASE GENE; U75685	0,128363526
TPGTGMPAAPMVPPT	MTB48 AAK31576	0,128048634
AAGLTYEHRLIDDMV	CAA17111.1; PROBABLE ISOCITRATE DEHYDROGENASE	0,123497887
PDLFDFLDSSVTTAR	MCE-FAMILY PROTEIN MCE1AYP_177701	0,119279282
LILTSYTSDEAMLDA	TWO COMPONENT TRANSCRIPTIONAL REGULATORY PROTEIN DEVR NP_217649	0,117752886
VGGRSIGVYAVNYPA	PROBABLE CUTINASE PRECURSOR CFP21 NP_216500	0,117377013
TFLTSELPQWLSANR	CAB10044.1; SECRETED ANTIGEN 85-B FBPB (85B)	0,114567921
GVEVTRAKVRRERMG	RNA POLYMERASE BETA'-SUBUNIT AAA21417	0,113644372
EGLPPGSALLVVKRG	CAB01474.1; CONSERVED HYPOTHETICAL PROTEIN CFP17	0,110945275
FKEMTLLELSDFVKK	CAB07109.1; PROBABLE 50S RIBOSOMAL PROTEIN L7/L12 RPLL	0,110724075
DIASEQAVLSSAWQG	CAA16104.1; SECRETED ESAT-6 LIKE PROTEIN ESXR	0,110561279
FAAVDHTGADVKLYQ	CONSERVED HYPOTHETICAL PROTEIN CAB08634	0,106267478
TSVFRADFLSELDAP	CAB01474.1; CONSERVED HYPOTHETICAL PROTEIN CFP17	0,104161343
PGTNWIGQAAEAYLN	CAB06237.1; CONSERVED HYPOTHETICAL PROTEIN	0,10231926
NNQVVLPFTDITAPW	TRANSMEMBRANE SERINE/THREONINE-PROTEIN KINASE D PKND NP_215446	0,101549487
AGKRRATIEQLLTIP	MTB81 (publication)	0,101059926
VAVTPEQRPLYRRDG	MYCOBACTERIUM BOVIS MYCOCEROSIC ACID SYNTHASE GENE; M95808	0,098910474
TFTIVAIGTYCPAVI	CAB10947.1; PROBABLE LIPOPROTEIN LPRJ	0,098761361
SQFGPYQLLRLLGRG	TRANSMEMBRANE SERINE/THREONINE-PROTEIN KINASE D PKND NP_215446	0,095039253
TWDDPQIAALNPGVN	PERIPLASMIC PHOSPHATE-BINDING LIPOPROTEIN PSTS1 YP_177770	0,091014436
ATDINAFSVGSGQTY	CAB09453.1; PROBABLE SERINE PROTEASE PEPA	0,089123793
RSCPGYTLDYNANGS	PERIPLASMIC PHOSPHATE-BINDING LIPOPROTEIN PSTS2 YP_177769	0,089021303
DVVVLLESMKMEIPV	CAB08316.1; BIOTINYLATED PROTEIN TB7.3	0,085806525
KVIATNKTARALAQY	MYCOBACTERIUM BOVIS MYCOCEROSIC ACID SYNTHASE GENE; M95808	0,08201149
YQAGKPTSTNPIASI	CAA17111.1; PROBABLE ISOCITRATE DEHYDROGENASE	0,079379647
RRAVQTDAEVVLPWD	CAA16030.1; PROBABLE MOLYBDOPTERIN-GUANINE DINUCLEOTIDE BIOSYNTHESIS PROTEIN	0,078087847
SNVPRLVPGWTKPIV	CAA17111.1; PROBABLE ISOCITRATE DEHYDROGENASE	0,071191478
VALFGEPSSGFSSML	PROBABLE CUTINASE PRECURSOR CFP21 NP_216500	0,071086281
IYEQANAHGQKVQAA	PUTATIVE ESAT-6 LIKE PROTEIN ESXN YP_177838	0,068735617
SYDAGVLTVRVAGAY	CAA17343.1; HEAT SHOCK PROTEIN HSP	0,066132977

Peptides	Protein	Average Index
PLTPARAVAIVRQIA	TRANSMEMBRANE SERINE/THREONINE-PROTEIN KINASE D PKND NP_215446	0,051326161
VANMPASVPKTSTYA	SECRETED L-ALANINE DEHYDROGENASE ALD CAA15575	0,050796812
VPDSSVPIIIVPPTP	YP_177963; PPE FAMILY PROTEIN	0,050313308
VQTDAEVVLPWDGRN	CAA16030.1; PROBABLE MOLYBDOPTERIN-GUANINE DINUCLEOTIDE BIOSYNTHESIS PROTEIN	0,049032747
YGEVKKPETINYRTL	RNA POLYMERASE BETA'-SUBUNIT AAA21417	0,047429912
FFPFTGLNSLTYDVS	PPE FAMILY PROTEIN CAE55504	0,046854498
LIKAILSLRHGVVPP	MYCOBACTERIUM BOVIS MYCOCEROSIC ACID SYNTHASE GENE; M95808	0,045866773
GPKGRNVVLEKKWGA	60 kDa chaperonin 2 P0A521	0,045855679
SLPAHLRPGLDELSF	PPE FAMILY PROTEIN CAE55504	0,044248467
ELAVDLAVPMIGMLK	CONSERVED HYPOTHETICAL PROTEIN TB16.3CAD97060	0,044083554
DYEGLWGLSGGSTIP	YP_177963; PPE FAMILY PROTEIN	0,043690161
LPDFLGGFAGLPSLG	CAB06237.1; CONSERVED HYPOTHETICAL PROTEIN	0,038123923
INSVLMYSGAGSSPL	YP_177963; PPE FAMILY PROTEIN	0,033661654
LFCTIRNFYDADPLA	MCE-FAMILY PROTEIN MCE1AYP_177701	0,032342608
IQQLAALGDVYADAA	MCE-FAMILY PROTEIN MCE1AYP_177701	0,030311271
EAPLKQIAFNSGLEP	60 kDa chaperonin 2 P0A521	0,029146264
VLLKRLPDALRDGNR	MYCOBACTERIUM BOVIS MYCOCEROSIC ACID SYNTHASE GENE; M95808	0,025868202
DTLFYCVANMPASVP	SECRETED L-ALANINE DEHYDROGENASE ALD CAA15575	0,02491999
LAENLKQRAEQLAAS	CONSERVED HYPOTHETICAL PROTEIN TB18.5CAD93033	0,024330846
FQDAYNAAGGHNAVF	CAB10044.1; SECRETED ANTIGEN 85-B FBPB (85B)	0,023933612
LDEHGTVLLAVRGLR	MYCOBACTERIUM BOVIS MYCOCEROSIC ACID SYNTHASE GENE; M95808	0,023776005
GFSEIMRSTLEKDNT	RNA POLYMERASE BETA-SUBUNIT AAA21416	0,023274973
RITILDAHFTGAFGP	YP_177963; PPE FAMILY PROTEIN	0,022796671
FPEIPANADGELYVI	YP_177963; PPE FAMILY PROTEIN	0,022483593
PSQFFPFTGLNSLTY	PPE FAMILY PROTEIN CAE55504	0,021905379
PGGTFESITSRMAEI	CAB10947.1; PROBABLE LIPOPROTEIN LPRJ	0,020019992
LVAAAQMWDSVASDL	CAE55371.1; PPE FAMILY PROTEIN	0,017471774
YDRTQDVAVLQLRGA	CAB09453.1; PROBABLE SERINE PROTEASE PEPA	0,016914847
QAAVFATELKRSRPP	TWO COMPONENT TRANSCRIPTIONAL REGULATORY PROTEIN DEVR NP_217649	0,016854755
AAQVAVRWAARDAEL	HYPOTHETICAL PROTEIN RV2623 NP_217139	0,01399293
LYLLLGWVAVWYTAT	POSSIBLE HEMOLYSIN-LIKE PROTEIN CAA17201	0,013364597
YELEGAVKRADLVIG	SECRETED L-ALANINE DEHYDROGENASE ALD CAA15575	0,012714761
PHWGPFGGAAPVSAG	PPE FAMILY PROTEIN CAE55334	0,009721193
VRERMTTQDVEAITP	RNA POLYMERASE BETA-SUBUNIT AAA21416	0,008406688
LPVYAEYQQRSEKVL	MTB48 AAK31576	0,005851981
CSSTKPVSQDTSPKP	CAA98350.1; CELL SURFACE LIPOPROTEIN MPT83	0,003327369
CGGVSTANATVYMID	CAA98373.1; MAJOR SECRETED IMMUNOGENIC PROTEIN	0,00247953
ELTAAQVRVAAAAYE	CAE55371.1; PPE FAMILY PROTEIN	0,001467407
GDLVGPGCAEYAAAN	CAA98373.1; MAJOR SECRETED IMMUNOGENIC PROTEIN	0,000943683
FATELKRSRPPGDGP	TWO COMPONENT TRANSCRIPTIONAL REGULATORY PROTEIN DEVR NP_217649	-0,004231027
MQRRDGLAHLGFDEV	PUTATIVE CYCLOPROPANE-FATTY-ACYL-PHOSPHOLIPID SYNTHASE UFAA1 NP_854118	-0,005901913
TPEQRPLYRRDGSYI	MYCOBACTERIUM BOVIS MYCOCEROSIC ACID SYNTHASE GENE; M95808	-0,005997758
GKSPGFGTTVDFPAV	PERIPLASMIC PHOSPHATE-BINDING LIPOPROTEIN PSTS1 YP_177770	-0,008749944
ADLSLLGKARKVVVT	60 kDa chaperonin 2 P0A521	-0,009309334
PQIQALNSGTDLPPT	PERIPLASMIC PHOSPHATE-BINDING LIPOPROTEIN PSTS2 YP_177769	-0,010820714
GNTTTVPSSGFFNTG	YP_177963; PPE FAMILY PROTEIN	-0,013153174
AAAGDLVGPGCAEYA	CAA98373.1; MAJOR SECRETED IMMUNOGENIC PROTEIN	-0,014070845
VVPLIDLEMRRPTYV	CONSERVED HYPOTHETICAL PROTEIN CAB08634	-0,015608476
MFRELVGLPAGSPEF	ALTERNATE RNA POLYMERASE SIGMA FACTOR SIGF CAB07069	-0,017821416
PAADLIGRGCAQYAA	CAA98350.1; CELL SURFACE LIPOPROTEIN MPT83	-0,019440838
FQVIYEQANAHGQKV	PUTATIVE ESAT-6 LIKE PROTEIN ESXN YP_177838	-0,019442092
EEDVLTSIHIEEHEI	RNA POLYMERASE BETA-SUBUNIT AAA21416	-0,022214855
DLSNDLFAAFLDETM	PUTATIVE CYCLOPROPANE-FATTY-ACYL-PHOSPHOLIPID SYNTHASE UFAA1 NP_854118	-0,023592002

Peptides	Protein	Average Index
VTGCAETPGCVAYIG	PERIPLASMIC PHOSPHATE-BINDING LIPOPROTEIN PSTS1 YP_177770	-0,026531245
ATIATFEMRYLQSLP	PPE FAMILY PROTEIN CAE55504	-0,029812475
ILTVSVAVSEGKPTE	CAA17245.1; HEAT SHOCK PROTEIN HSPX	-0,02995089
DPIPLNFDVLSSAGP	YP_177963; PPE FAMILY PROTEIN	-0,030851211
RMTSGSSSGFKVRPS	YP_177963; PPE FAMILY PROTEIN	-0,034464359
EPIDVPAVVLDIHET	YP_177963; PPE FAMILY PROTEIN	-0,034598722
ASPVAQSYLRNFLAA	LOW MOLECULAR WEIGHT T-CELL ANTIGEN TB8.4 NP_215690	-0,035832642
EPSDLVYGDVIMRST	POSSIBLE GLYCOSYL TRANSFERASE CAB05419	-0,036822196
EVTVETTSVFRADFL	CAB01474.1; CONSERVED HYPOTHETICAL PROTEIN CFP17	-0,038745964
GLAVDTQGAVYVADR	TRANSMEMBRANE SERINE/THREONINE-PROTEIN KINASE D PKND NP_215446	-0,040285395
QRHRDKIVQRCLPLA	ALTERNATE RNA POLYMERASE SIGMA FACTOR SIGF CAB07069	-0,041854845
FLDDVTVSRRHAEFR	CAB01474.1; CONSERVED HYPOTHETICAL PROTEIN CFP17	-0,041892878
AKERQRLATSLRNAA	MTB48 AAK31576	-0,044071686
TVTTGIVSALNRPVS	CAA17582.1; PROBABLE SERINE PROTEASE PEPD	-0,045205768
LIVEPWMGPAAAAMA	PPE FAMILY PROTEIN CAE55334	-0,045283687
DAQSIQAAAAGFASK	PERIPLASMIC PHOSPHATE-BINDING LIPOPROTEIN PSTS1 YP_177770	-0,045295093
VEQASVILRRTIDAD	MYCOBACTERIUM BOVIS MYCOCEROSIC ACID SYNTHASE GENE; M95808	-0,045367602
SARMYAGPGSASLVA	CAE55371.1; PPE FAMILY PROTEIN	-0,045482531
YIGISFLDQASQRGL	PERIPLASMIC PHOSPHATE-BINDING LIPOPROTEIN PSTS1 YP_177770	-0,045622083
IPANADGELYVIAGD	YP_177963; PPE FAMILY PROTEIN	-0,046545368
ELAIPMSGIAMAVVG	CAB06237.1; CONSERVED HYPOTHETICAL PROTEIN	-0,047850197
TSYTSDEAMLDAILA	TWO COMPONENT TRANSCRIPTIONAL REGULATORY PROTEIN DEVR NP_217649	-0,048217872
QNTMKIGIMDEERRT	MTB81 (publication)	-0,048398898
AMLARDGAEAAKWGG	CAA16104.1; SECRETED ESAT-6 LIKE PROTEIN ESXR	-0,050528795
GSFASVTTGLAGDAW	PPE FAMILY PROTEIN CAE55504	-0,050659567
ANIPAEFLENFVRSS	CAB10044.1; SECRETED ANTIGEN 85-B FBPB (85B)	-0,052448584
QVALAATMEQTYGVR	MYCOBACTERIUM BOVIS MYCOCEROSIC ACID SYNTHASE GENE; M95808	-0,053977182
AWGAWGEVGRATFLA	MYCOBACTERIUM BOVIS MYCOCEROSIC ACID SYNTHASE GENE; M95808	-0,054201565
ILVQANEAETTTASG	CAB01005.1; 10 KDA CHAPERONIN GROES	-0,055922523
VRPVSILYRLYRPLI	POSSIBLE GLYCOSYL TRANSFERASE CAB05418	-0,057132727
AAMGAFTIPQIDIPA	YP_177963; PPE FAMILY PROTEIN	-0,05728475
TRHTQTWIQKYIFPG	PUTATIVE CYCLOPROPANE-FATTY-ACYL-PHOSPHOLIPID SYNTHASE UFAA1 NP_854118	-0,057352198
AVVRVQGVSGLTPIS	CAA17582.1; PROBABLE SERINE PROTEASE PEPD	-0,057443827
GGKAQFGGQRFGEME	RNA POLYMERASE BETA-SUBUNIT AAA21416	-0,058227684
GSVVSTLIVEPWMGP	PPE FAMILY PROTEIN CAE55334	-0,058274288
DVDKDVNVELDPGQP	CAA17343.1; HEAT SHOCK PROTEIN HSP	-0,058778695
LTEPHIVPIHDYGEI	TRANSMEMBRANE SERINE/THREONINE-PROTEIN KINASE D PKND NP_215446	-0,059645627
IATVESGKMTKDLAI	CAA17111.1; PROBABLE ISOCITRATE DEHYDROGENASE	-0,060327547
YPEGLAVDTQGAVYV	TRANSMEMBRANE SERINE/THREONINE-PROTEIN KINASE D PKND NP_215446	-0,063677203
EPGVPGRSVSRWGGF	MYCOBACTERIUM BOVIS MYCOCEROSIC ACID SYNTHASE GENE; M95808	-0,063918218
PIPEGLTTGRSCQTD	POSSIBLE HEMOLYSIN CAA16235	-0,064740073
SSRYGHWWPAVAVGC	MYCOBACTERIUM BOVIS MYCOCEROSIC ACID SYNTHASE GENE; M95808	-0,065053871
RVSVGNLRIARVLYD	MTB81 (publication)	-0,067364096
TWKLLLDRQSGIRTL	3-OXOACYL-[ACYL-CARRIER PROTEIN] SYNTHASE 2 KASB CAA94642	-0,067840306
FPQHVENALRLMPEN	POSSIBLE GLYCOSYL TRANSFERASE CAB05415	-0,068356056
IEDAVRNAKAAVEEG	60 kDa chaperonin 2 P0A521	-0,069138783
MRPSKTFNAALEAVQ	PROBABLE ISOCITRATE DEHYDROGENASE CAA16247	-0,069167901
RRGLTFYYLDLALMS	MYCOBACTERIUM BOVIS MYCOCEROSIC ACID SYNTHASE GENE; M95808	-0,069360565
DSIYYVDANASIQEM	HYPOTHETICAL PROTEIN RV2626C NP_217142	-0,069727511
TCVGEHETLTAAAQY	HYPOTHETICAL PROTEIN RV2626C NP_217142	-0,069792544

Peptides	Protein	Average Index
NPPKPPPAIKIDPPP	MTB48 AAK31576	-0,070777858
AERFMSAGDLAIAAH	TRANSMEMBRANE SERINE/THREONINE-PROTEIN KINASE D PKND NP_215446	-0,072361233
EADLRVLDEIAPDLV	POSSIBLE GLYCOSYL TRANSFERASE CAB05415	-0,072634562
TGMELRAAIDAATSS	RNA POLYMERASE BETA-SUBUNIT AAA21416	-0,072983254
GRAGGGAALGGGGMG	MTB48 AAK31576	-0,073933201
HESNTMAMLARDGAE	CAA16104.1; SECRETED ESAT-6 LIKE PROTEIN ESXR	-0,074016454
RQAVPLVRSEAPLVG	RNA POLYMERASE BETA-SUBUNIT AAA21416	-0,074873616
DISVAARILAEFPDY	PROBABLE ISOCITRATE DEHYDROGENASE CAA16247	-0,078294731
ARDADMLSELGEPVD	CONSERVED HYPOTHETICAL PROTEIN CAB08634	-0,0787349
ANATVYMIDSVLMPP	CAA98373.1; MAJOR SECRETED IMMUNOGENIC PROTEIN	-0,079265066
DLFVGGNIISGIPTL	YP_177963; PPE FAMILY PROTEIN	-0,079452154
PRREPRRCWPRSRCP	MYCOBACTERIUM BOVIS MYCOCEROSIC ACID SYNTHASE GENE; M95808	-0,080070107
VMQQGELFAKQEQLF	CONSERVED HYPOTHETICAL PROTEIN TB18.5CAD93033	-0,081310769
INIRPVVAAIKEFFG	RNA POLYMERASE BETA-SUBUNIT AAA21416	-0,08134969
YLKDHDTEGLDIQIM	PROBABLE ISOCITRATE DEHYDROGENASE CAA16247	-0,082534993
SPRWRESAAERGDVN	RNA POLYMERASE BETA-SUBUNIT AAA21416	-0,082686603
FTAHTKHYRVVSKPA	CAE55335.1; PE FAMILY PROTEIN	-0,082946976
IAASYDAGVLTVRVA	CAA17343.1; HEAT SHOCK PROTEIN HSP	-0,083632452
ASNSTGMSGGFVTAP	YP_177963; PPE FAMILY PROTEIN	-0,084841509
FQEAANKQKQELDEI	CAA17966.1; 10 KDA CULTURE FILTRATE ANTIGEN ESXB	-0,086572623
LSSLGWNLCRIFTDG	POSSIBLE GLYCOSYL TRANSFERASE CAB05415	-0,088058909
RKIPLTLVHAVSPEV	HYPOTHETICAL PROTEIN RV2623 NP_217139	-0,088232675
TKVDDRPINSADALV	CAA17582.1; PROBABLE SERINE PROTEASE PEPD	-0,089465709
TRGRCAAISVPGDRR	MYCOBACTERIUM BOVIS ACYL-COA SYNTHASE GENE; U75685	-0,090174764
KNVPANAFVADYLPG	POSSIBLE GLYCOSYL TRANSFERASE CAB05418	-0,091497454
NRANEVEAPMADPPT	MTB48 AAK31576	-0,092137708
RRDPRWEADLDVLDE	MYCOBACTERIUM BOVIS MYCOCEROSIC ACID SYNTHASE GENE; M95808	-0,093269203
FGFGNSGLAAGLAGM	YP_177963; PPE FAMILY PROTEIN	-0,093747878
AEAEYEQIWAQDVAA	PPE FAMILY PROTEIN CAE55504	-0,094304556
SLSAGHAKPCAGGGA	MYCOBACTERIUM BOVIS ACYL-COA SYNTHASE GENE; U75685	-0,095064813
TVASCPNTRIVLGGY	PROBABLE CUTINASE PRECURSOR CFP21 NP_216500	-0,095218522
FTDGDYTLYADVPEL	POSSIBLE GLYCOSYL TRANSFERASE CAB05418	-0,096144215
TNDGVSIAKEIELED	60 kDa chaperonin 2 P0A521	-0,096735389
HSGWKVDAAKGVPDW	RNA POLYMERASE BETA-SUBUNIT AAA21416	-0,096899365
PGAAQYIGLVESVAG	LOW MOLECULAR WEIGHT T-CELL ANTIGEN TB8.4 NP_215690	-0,099226827
VVWGLTVGSWIGSSA	CAE55371.1; PPE FAMILY PROTEIN	-0,099287706
GFHMVAPDPNGERAG	3-OXOACYL-[ACYL-CARRIER PROTEIN] SYNTHASE 2 KASB CAA94642	-0,099297075
FAVQVALAATMEQTY	MYCOBACTERIUM BOVIS MYCOCEROSIC ACID SYNTHASE GENE; M95808	-0,100160942
SELATAIAFDEASRR	HYPOTHETICAL PROTEIN RV2623 NP_217139	-0,101369903
HLRWDSLGEFLALGA	PROBABLE ISOCITRATE DEHYDROGENASE CAA16247	-0,101537906
VGDLRISLSVSARLA	POSSIBLE GLYCOSYL TRANSFERASE CAB05415	-0,101898927
GGNIMAHVSYVQSGM	PROBABLE CUTINASE PRECURSOR CFP21 NP_216500	-0,101917471
RGEPRDDLIQVARVG	ALTERNATE RNA POLYMERASE SIGMA FACTOR SIGF CAB07069	-0,102936807
MTYAAPLFVTAEFIN	RNA POLYMERASE BETA-SUBUNIT AAA21416	-0,103648401
LTYHVIAGQASPSRI	CAA98350.1; CELL SURFACE LIPOPROTEIN MPT83	-0,10389026
SSAGLMVAAASPYVA	CAE55371.1; PPE FAMILY PROTEIN	-0,105586496
IFGEKAREVRDTSLK	RNA POLYMERASE BETA-SUBUNIT AAA21416	-0,105978606
PGAARWSPGDSATVA	TRANSMEMBRANE SERINE/THREONINE-PROTEIN KINASE D PKND NP_215446	-0,106558293
LLKGAKEVETKEQIA	60 kDa chaperonin 2 P0A521	-0,108180039
TIKTWDDPQIAALNP	PERIPLASMIC PHOSPHATE-BINDING LIPOPROTEIN PSTS1 YP_177770	-0,109619753
YVIAGDIPLINIPPT	YP_177963; PPE FAMILY PROTEIN	-0,111653732
DVILEAAGDKKIGVI	CAB07109.1; PROBABLE 50S RIBOSOMAL PROTEIN L7/L12 RPLL	-0,111888089
NQSFPVTVNWSTPAV	YP_177963; PPE FAMILY PROTEIN	-0,113173935
MNFPVLPPEINSVLM	YP_177963; PPE FAMILY PROTEIN	-0,113387986
SSDLRVGQPVLAIGS	CAA17582.1; PROBABLE SERINE PROTEASE PEPD	-0,113566083
SYNTGIGNSGTGSTG	YP_177963; PPE FAMILY PROTEIN	-0,113806704
WSPTQASGQTVLPFT	TRANSMEMBRANE SERINE/THREONINE-PROTEIN KINASE D PKND NP_215446	-0,114304346

Peptides	Protein	Average Index
LSFTLTGNPNRPDGG	PPE FAMILY PROTEIN CAE55504	-0,114775244
NTGGFNPGSTNTGWF	YP_177963; PPE FAMILY PROTEIN	-0,116290228
APIQADMMGNAFLTA	CAB10947.1; PROBABLE LIPOPROTEIN LPRJ	-0,118131858
TDIDPDSFWAGVDKV	MTB81 (publication)	-0,118267936
LQQLAQPTQGTTPSS	CAE55371.1; PPE FAMILY PROTEIN	-0,118460521
IKKHGVGVKCATITP	CAA17111.1; PROBABLE ISOCITRATE DEHYDROGENASE	-0,118769214
MLDVNFFDELRIGLA	RNA POLYMERASE BETA'-SUBUNIT AAA21417	-0,118774235
KGASETFNGGVGVGA	PERIPLASMIC PHOSPHATE-BINDING LIPOPROTEIN PSTS2 YP_177769	-0,118858236
GGINSPDKLWESLLR	MYCOBACTERIUM BOVIS MYCOCEROSIC ACID SYNTHASE GENE; M95808	-0,120195121
TMTELMADMVETKIA	MTB81 (publication)	-0,122277649
LLRYAATNRLGSHRS	CONSERVED HYPOTHETICAL PROTEIN CAB08634	-0,123385066
RAAAEAFGTAFAMTV	PPE FAMILY PROTEIN CAE55334	-0,124875653
PINSADALVAAVRSK	CAA17582.1; PROBABLE SERINE PROTEASE PEPD	-0,126022613
LSQFMDQNNPLSGLT	RNA POLYMERASE BETA-SUBUNIT AAA21416	-0,126067928
DPQIAALNPGVNLPG	PERIPLASMIC PHOSPHATE-BINDING LIPOPROTEIN PSTS1 YP_177770	-0,126468248
LLAKSGKTIVLKPEV	PROBABLE ISOCITRATE DEHYDROGENASE CAA16247	-0,127275906
LTFYYLDLALMSVTQ	MYCOBACTERIUM BOVIS MYCOCEROSIC ACID SYNTHASE GENE; M95808	-0,127899824
DNWIVGASNSTGMSG	YP_177963; PPE FAMILY PROTEIN	-0,128680841
LAILIGPEQDWLNSE	CAA17111.1; PROBABLE ISOCITRATE DEHYDROGENASE	-0,128897185
IRLDYYDLGIEHRDA	CAA17111.1; PROBABLE ISOCITRATE DEHYDROGENASE	-0,128950472
PAEVAFTCELFSRVE	MTB81 (publication)	-0,129407613
QIADRMFLAEKTVKN	TWO COMPONENT TRANSCRIPTIONAL REGULATORY PROTEIN DEVR NP_217649	-0,131253556
YMAGEWSSKELTRVL	PUTATIVE CYCLOPROPANE-FATTY-ACYL-PHOSPHOLIPID SYNTHASE UFAA1 NP_854118	-0,13282891
APAQAAPPALSQDRF	CAB09453.1; PROBABLE SERINE PROTEASE PEPA	-0,132930422
VLNFGSGVSGLYNTG	YP_177963; PPE FAMILY PROTEIN	-0,132986596
ANYGFAVPDIPIPAI	YP_177963; PPE FAMILY PROTEIN	-0,134441257
LTVLALRDQVIPPTL	3-OXOACYL-[ACYL-CARRIER PROTEIN] SYNTHASE 2 KASB CAA94642	-0,134672946
KASGSTAQANAMTRF	PERIPLASMIC PHOSPHATE-BINDING LIPOPROTEIN PSTS3 YP_177768	-0,134712349
TNQDGRTETLTMPSE	MYCOBACTERIUM BOVIS MYCOCEROSIC ACID SYNTHASE GENE; M95808	-0,136252138
SGGSGGSGQPSIIDA	YP_177963; PPE FAMILY PROTEIN	-0,136781068
LVNIGPFRIEPIDVP	YP_177963; PPE FAMILY PROTEIN	-0,137853759
DTGALMSGNFSNGIL	YP_177963; PPE FAMILY PROTEIN	-0,137921707
DEMTRVIWKLIKDML	CAA17111.1; PROBABLE ISOCITRATE DEHYDROGENASE	-0,138017744
PAHAAHHLVEGVARV	POSSIBLE HEMOLYSIN-LIKE PROTEIN CAA17201	-0,138038762
FRPSWPDHSRDQARR	PUTATIVE CYCLOPROPANE-FATTY-ACYL-PHOSPHOLIPID SYNTHASE UFAA1 NP_854118	-0,139942488
AIVDTDGSEVFEGIM	MTB81 (publication)	-0,140070359
YNTAIVGLGTPALVS	YP_177963; PPE FAMILY PROTEIN	-0,140133856
NTGAGGGSGFGNFGA	YP_177963; PPE FAMILY PROTEIN	-0,141266293
PESSPGDAEVGPRLF	MYCOBACTERIUM BOVIS MYCOCEROSIC ACID SYNTHASE GENE; M95808	-0,142320364
GLSNTIVDKEFLKRL	POSSIBLE GLYCOSYL TRANSFERASE CAB05419	-0,142657535
VSSFGMSGTNVHAIV	MYCOBACTERIUM BOVIS MYCOCEROSIC ACID SYNTHASE GENE; M95808	-0,142868919
SEAIAASYDAGVLTV	CAA17343.1; HEAT SHOCK PROTEIN HSP	-0,143275654
VLMVVGCLGSGRWPG	HYPOTHETICAL PROTEIN RV2623 NP_217139	-0,143954308
GGVSGFGNFGAGSSG	YP_177963; PPE FAMILY PROTEIN	-0,144927959
ATDEPEPHWGPFGGA	PPE FAMILY PROTEIN CAE55334	-0,145015179
VNIGASDAYLSEGDM	PERIPLASMIC PHOSPHATE-BINDING LIPOPROTEIN PSTS1 YP_177770	-0,145576163
KDVDIMVRDGQLTIK	CAA17245.1; HEAT SHOCK PROTEIN HSPX	-0,146383806
QTYGVDVVGYDRTQD	CAB09453.1; PROBABLE SERINE PROTEASE PEPA	-0,147848034
TTVALGRSVCPMVVA	CAB10947.1; PROBABLE LIPOPROTEIN LPRJ	-0,14890895
SVPKTSTYALTNATM	SECRETED L-ALANINE DEHYDROGENASE ALD CAA15575	-0,148953071
ITGTRIDPIPLNFDV	YP_177963; PPE FAMILY PROTEIN	-0,149292252
DMGVEHVYDSRSVEF	MYCOBACTERIUM BOVIS MYCOCEROSIC ACID SYNTHASE GENE;	-0,149299443

Peptides	Protein	Average Index
	M95808	
WTKPIVIGRHAFGDQ	CAA17111.1; PROBABLE ISOCITRATE DEHYDROGENASE	-0,149463323
ANLGRAASVGSLSVP	CAE55371.1; PPE FAMILY PROTEIN	-0,149600602
VFRARMQREADTAGR	TRANSMEMBRANE SERINE/THREONINE-PROTEIN KINASE D PKND NP_215446	-0,150458274
TIPLAKELAWAPDEI	MTB81 (publication)	-0,151760685
LSSMLKGFAPAAAAQ	CAE55371.1; PPE FAMILY PROTEIN	-0,151875534
GESGKVIGIRVFSRE	RNA POLYMERASE BETA-SUBUNIT AAA21416	-0,152576476
EVFGEAAEVRDITFE	MYCOBACTERIUM BOVIS MYCOCEROSIC ACID SYNTHASE GENE; M95808	-0,153835072
SGVIEEVSADYITVM	RNA POLYMERASE BETA-SUBUNIT AAA21416	-0,153889286
GPAGPAAAAAATQAA	PPE FAMILY PROTEIN CAE55334	-0,15509305
RIVMADSRPLTNVNS	CAA16030.1; PROBABLE MOLYBDOPTERIN-GUANINE DINUCLEOTIDE BIOSYNTHESIS PROTEIN	-0,156195863
AYSEAGFRSGYLDVY	PUTATIVE CYCLOPROPANE-FATTY-ACYL-PHOSPHOLIPID SYNTHASE UFAA1 NP_854118	-0,15623861
GLANAGSYNTGSLNA	YP_177963; PPE FAMILY PROTEIN	-0,156315684
KLTADGVLTAPQCTH	MYCOBACTERIUM BOVIS MYCOCEROSIC ACID SYNTHASE GENE; M95808	-0,15637674
QADMMGNAFLTALTN	CAB10947.1; PROBABLE LIPOPROTEIN LPRJ	-0,157709716
GLFGLNVGITIPEFP	YP_177963; PPE FAMILY PROTEIN	-0,157870238
QPGSYPIVLATYEIV	PERIPLASMIC PHOSPHATE-BINDING LIPOPROTEIN PSTS2 YP_177769	-0,159017115
ASCRFVPTCSQYAVD	POSSIBLE HEMOLYSIN CAA16235	-0,159064901
TSEIEPFIVWTSSGA	YP_177963; PPE FAMILY PROTEIN	-0,160340663
QPVVLAAATGDLPTI	CAE55335.1; PE FAMILY PROTEIN	-0,160357476
LSGCSSNKSTTGSGE	LIPOPROTEIN LPQH PRECURSOR P0A5J0	-0,161103986
FSTPPITIDRIPLNL	YP_177963; PPE FAMILY PROTEIN	-0,161117124
RREFKARAAEKPAPS	MTB81 (publication)	-0,161285709
SPLLAAAAAWDGLAE	YP_177963; PPE FAMILY PROTEIN	-0,162224602
DSVAAVDAADKVLGY	MTB81 (publication)	-0,16283228
LIPGFLMPPSDGSGV	MTB48 AAK31576	-0,163437319
NLFPGLPGLGDLLPG	CAB06237.1; CONSERVED HYPOTHETICAL PROTEIN	-0,164056206
LGQNTPAIAVNEAEY	CAE55371.1; PPE FAMILY PROTEIN	-0,164206229
GPLYSSKTINLCAPD	PROBABLE CUTINASE PRECURSOR CFP21 NP_216500	-0,165432565
WISEYKEVEILEADD	CONSERVED HYPOTHETICAL PROTEIN TB16.3CAD97060	-0,166510769
DSLPTDRPIVYATLG	POSSIBLE GLYCOSYL TRANSFERASE CAB05415	-0,166576605
AVQQGLAGKRRATIE	MTB81 (publication)	-0,166590266
ELGRLTQLPDTNIIK	PROBABLE ISOCITRATE DEHYDROGENASE CAA16247	-0,167558673
GPYLQRGVADLVPTA	MCE-FAMILY PROTEIN MCE1AYP_177701	-0,167625355
LLVVGNVGLSTIAGR	CAB08889.1; IRON-REGULATED CONSERVED HYPOTHETICAL PROTEIN	-0,168572449
MKEGRYEVRAELPGV	CAA17245.1; HEAT SHOCK PROTEIN HSPX	-0,168729832
SEGCAMVLLKRLPDA	MYCOBACTERIUM BOVIS MYCOCEROSIC ACID SYNTHASE GENE; M95808	-0,168876205
NAMTRFVNVFEQACP	PERIPLASMIC PHOSPHATE-BINDING LIPOPROTEIN PSTS3 YP_177768	-0,169401079
EVHFACDPRFNKLLG	POSSIBLE GLYCOSYL TRANSFERASE CAB05418	-0,169702202
ENILVTASDFAYLVD	TRANSMEMBRANE SERINE/THREONINE-PROTEIN KINASE D PKND NP_215446	-0,169783762
TDEQAITAATAHATA	MYCOBACTERIUM BOVIS MYCOCEROSIC ACID SYNTHASE GENE; M95808	-0,170048529
LPTVTIPTITTSPIP	YP_177963; PPE FAMILY PROTEIN	-0,170839498
TVIGGTSDTVRDLIA	MYCOBACTERIUM BOVIS MYCOCEROSIC ACID SYNTHASE GENE; M95808	-0,171381836
IVISNVPRLVPGWTK	CAA17111.1; PROBABLE ISOCITRATE DEHYDROGENASE	-0,171425604
EFRLENNEFNVVDVG	CAB01474.1; CONSERVED HYPOTHETICAL PROTEIN CFP17	-0,171844343
AGGLASPYVVTGLNN	MYCOBACTERIUM BOVIS MYCOCEROSIC ACID SYNTHASE GENE; M95808	-0,172067191
VVVFGTSQSATIATF	PPE FAMILY PROTEIN CAE55504	-0,172913822
DADPLAKAASGGGNG	MCE-FAMILY PROTEIN MCE1AYP_177701	-0,173514756
ANPMSPVNKSFEIEV	LIPOPROTEIN LPQH PRECURSOR P0A5J0	-0,174224316
EYKEVEILEADDEGY	CONSERVED HYPOTHETICAL PROTEIN TB16.3CAD97060	-0,174495455
TGMPAAPMVPPTGSP	MTB48 AAK31576	-0,174749564
GDMAGYAGTLQSLGA	CAA16104.1; SECRETED ESAT-6 LIKE PROTEIN ESXR	-0,17477305

Peptides	Protein	Average Index
AADVAAMAGYHSGAS	YP_177963; PPE FAMILY PROTEIN	-0,174823066
GQVVGMNTAASDNFQ	CAB09453.1; PROBABLE SERINE PROTEASE PEPA	-0,17513399
SAAAFAAPAQANPVD	CONSERVED HYPOTHETICAL PROTEIN CAA15739	-0,175311239
TSDEAMLDAILAGAS	TWO COMPONENT TRANSCRIPTIONAL REGULATORY PROTEIN DEVR NP_217649	-0,175793812
NFQKYLDGASNGAWG	PERIPLASMIC PHOSPHATE-BINDING LIPOPROTEIN PSTS2 YP_177769	-0,175944873
PDGNGIELCRDLLSR	TWO COMPONENT TRANSCRIPTIONAL REGULATORY PROTEIN DEVR NP_217649	-0,176625491
DPTSDIAVVRVQGVS	CAA17582.1; PROBABLE SERINE PROTEASE PEPD	-0,176840191
IKERYARCLGSAVNP	PROBABLE ISOCITRATE DEHYDROGENASE CAA16247	-0,177253053
EIVKDGDDAVVRLEL	CAA17343.1; HEAT SHOCK PROTEIN HSP	-0,177530568
NPQVGSAQPIVIPTP	PPE FAMILY PROTEIN CAE55334	-0,177829373
TAAQNGVRAMSSLGS	CAE55371.1; PPE FAMILY PROTEIN	-0,178355057
FFNTGGGGGSGIANF	YP_177963; PPE FAMILY PROTEIN	-0,178787115
PAVFAVQVALAATME	MYCOBACTERIUM BOVIS MYCOCEROSIC ACID SYNTHASE GENE; M95808	-0,179001881
PGLPDFKWPPTPGSP	CAB06237.1; CONSERVED HYPOTHETICAL PROTEIN	-0,179074005
GLGLATAPAQAAPPA	CAB09453.1; PROBABLE SERINE PROTEASE PEPA	-0,180793078
EKAIKERYARCLGSA	PROBABLE ISOCITRATE DEHYDROGENASE CAA16247	-0,181214217
WAGRLRRLLVEQASV	MYCOBACTERIUM BOVIS MYCOCEROSIC ACID SYNTHASE GENE; M95808	-0,181441312
EAGDIWRMCIVKDAP	PROBABLE ISOCITRATE DEHYDROGENASE CAA16247	-0,18173021
RNFLAAPPPQRAAMA	LOW MOLECULAR WEIGHT T-CELL ANTIGEN TB8.4 NP_215690	-0,182207518
IVKVALEAPLKQIAF	60 kDa chaperonin 2 P0A521	-0,182383221
IPIGQAMAIAGQIRS	CAB09453.1; PROBABLE SERINE PROTEASE PEPA	-0,182519184
AGRNHLKNVPANAFV	POSSIBLE GLYCOSYL TRANSFERASE CAB05418	-0,183243348
YNAARIANGMGATVT	SECRETED L-ALANINE DEHYDROGENASE ALD CAA15575	-0,183301284
PGLTQTGTAVGTYNY	TRANSMEMBRANE SERINE/THREONINE-PROTEIN KINASE D PKND NP_215446	-0,183776686
AASLLTVDMDVQVTM	CONSERVED HYPOTHETICAL PROTEIN TB18.5CAD93033	-0,183850828
GLWGLSGGSTIPAIP	YP_177963; PPE FAMILY PROTEIN	-0,184513211
MVIPPIVFLPSMTIG	YP_177963; PPE FAMILY PROTEIN	-0,184748421
LPPDLASGVVQPVSS	PPE FAMILY PROTEIN CAE55504	-0,185283477
PIPLKIDVSGGLPAF	YP_177963; PPE FAMILY PROTEIN	-0,186766311
HEGQTTMTVPGGVEV	RNA POLYMERASE BETA-SUBUNIT AAA21416	-0,187270215
TRVIWKLIKDMLILP	CAA17111.1; PROBABLE ISOCITRATE DEHYDROGENASE	-0,188179001
MAAPGQPLPTLPVPV	CAA16030.1; PROBABLE MOLYBDOPTERIN-GUANINE DINUCLEOTIDE BIOSYNTHESIS PROTEIN	-0,188253178
LSLSVSARLAGIPYI	POSSIBLE GLYCOSYL TRANSFERASE CAB05418	-0,189259247
WPRAQGLPVSAIAWG	MYCOBACTERIUM BOVIS MYCOCEROSIC ACID SYNTHASE GENE; M95808	-0,190488101
AALRACACLQPHDPA	MYCOBACTERIUM BOVIS ACYL-COA SYNTHASE GENE; U75685	-0,191227682
DELLDAFKEMTLLEL	CAB07109.1; PROBABLE 50S RIBOSOMAL PROTEIN L7/L12 RPLL	-0,193620056
VLKPEVPLDDGDVID	PROBABLE ISOCITRATE DEHYDROGENASE CAA16247	-0,193934774
KGAGKSFQGGVGEGA	PERIPLASMIC PHOSPHATE-BINDING LIPOPROTEIN PSTS3 YP_177768	-0,194237471
WESLLRGDDLVTEIP	MYCOBACTERIUM BOVIS MYCOCEROSIC ACID SYNTHASE GENE; M95808	-0,194665763
LKQIAFNSGLEPGVV	60 kDa chaperonin 2 P0A521	-0,194715257
RTLNVARELSRCGST	MYCOBACTERIUM BOVIS ACYL-COA SYNTHASE GENE; U75685	-0,195633881
QKRKISDGDKLAGRH	RNA POLYMERASE BETA-SUBUNIT AAA21416	-0,196560051
GTAVSARNTAVCAPT	CAE55335.1; PE FAMILY PROTEIN	-0,197187132
ALPLTSLTSAAERGP	CAE55371.1; PPE FAMILY PROTEIN	-0,197536381
PILDDLAAALADIAP	MYCOBACTERIUM BOVIS MYCOCEROSIC ACID SYNTHASE GENE; M95808	-0,197789918
AHTPRREPRRCWPRS	MYCOBACTERIUM BOVIS MYCOCEROSIC ACID SYNTHASE GENE; M95808	-0,19882437
HPHAVNGTAMRESFA	MYCOBACTERIUM BOVIS MYCOCEROSIC ACID SYNTHASE GENE; M95808	-0,199295443
RYTCERLVRGLDTIA	PROBABLE ISOCITRATE DEHYDROGENASE CAA16247	-0,19955348
TTTAPSSGFFNSGDG	YP_177963; PPE FAMILY PROTEIN	-0,199773377
PFTDITAPWGIAVDE	TRANSMEMBRANE SERINE/THREONINE-PROTEIN KINASE D PKND NP_215446	-0,20006095
LDELSFTLTGNPNRP	PPE FAMILY PROTEIN CAE55504	-0,20110747

Peptides	Protein	Average Index
TVGQVVLGWKVSDLK	CAB06500.1; IMMUNOGENIC PROTEIN MPT63	-0,201293271
VSPLTVQKYMPNGAA	3-OXOACYL-[ACYL-CARRIER PROTEIN] SYNTHASE 2 KASB CAA94642	-0,201359334
SVPANVSRRAKVDVL	CAB08889.1; IRON-REGULATED CONSERVED HYPOTHETICAL PROTEIN	-0,203571537
MALGKNLLVAIMPWE	RNA POLYMERASE BETA-SUBUNIT AAA21416	-0,204735963
TSELPQWLSANRAVK	CAB10044.1; SECRETED ANTIGEN 85-B FBPB (85B)	-0,204910453
VSVAVSEGKPTEKHI	CAA17245.1; HEAT SHOCK PROTEIN HSPX	-0,205233162
AGLAPGDIDHVNAHA	3-OXOACYL-[ACYL-CARRIER PROTEIN] SYNTHASE 2 KASB CAA94642	-0,205358515
RVVRVTGRASARGLI	POSSIBLE HEMOLYSIN CAA16235	-0,206236058
VPLVRSEAPLVGTGM	RNA POLYMERASE BETA-SUBUNIT AAA21416	-0,206407597
HEVVRRGLVDLLGAD	TWO COMPONENT TRANSCRIPTIONAL REGULATORY PROTEIN DEVR NP_217649	-0,206627714
VGADEDDIKATYDKG	CAA17245.1; HEAT SHOCK PROTEIN HSPX	-0,207249684
TIPAIPIGLELNGGV	YP_177963; PPE FAMILY PROTEIN	-0,207728964
NDKSPSRKTGELDNR	PROBABLE ISOCITRATE DEHYDROGENASE CAA16247	-0,207797022
IQLAGLAPGDIDHVN	3-OXOACYL-[ACYL-CARRIER PROTEIN] SYNTHASE 2 KASB CAA94642	-0,207867449
APSLFIHRPDALARR	PUTATIVE CYCLOPROPANE-FATTY-ACYL-PHOSPHOLIPID SYNTHASE UFAA1 NP_854118	-0,208719588
DADRSFIEYGLDSLG	MYCOBACTERIUM BOVIS MYCOCEROSIC ACID SYNTHASE GENE; M95808	-0,208725463
AGQAMASTEGNVTGM	CAE55648	-0,209246103
DRTVYVADRGNDRVV	TRANSMEMBRANE SERINE/THREONINE-PROTEIN KINASE D PKND NP_215446	-0,211283699
LATAPAQAAPPALSQ	CAB09453.1; PROBABLE SERINE PROTEASE PEPA	-0,211530699
GLNYPEGLAVDTQGA	TRANSMEMBRANE SERINE/THREONINE-PROTEIN KINASE D PKND NP_215446	-0,211564209
ARASSRYGHWWPAVA	MYCOBACTERIUM BOVIS MYCOCEROSIC ACID SYNTHASE GENE; M95808	-0,211931303
AHLRPGLDELSFTLT	PPE FAMILY PROTEIN CAE55504	-0,212362108
AGSWLLIDTSNAVDT	MYCOBACTERIUM BOVIS MYCOCEROSIC ACID SYNTHASE GENE; M95808	-0,212741112
GVDVVGYDRTQDVAV	CAB09453.1; PROBABLE SERINE PROTEASE PEPA	-0,212767299
RYFAALEQLVRPGGP	PUTATIVE CYCLOPROPANE-FATTY-ACYL-PHOSPHOLIPID SYNTHASE UFAA1 NP_854118	-0,213181742
EPVVAMGNSGGQGGT	CAB09453.1; PROBABLE SERINE PROTEASE PEPA	-0,213997114
AENRAELMILIATNL	CAE55371.1; PPE FAMILY PROTEIN	-0,214607924
ADEEQQQALSSQMGF	CAA17966.1; 10 KDA CULTURE FILTRATE ANTIGEN ESXB	-0,21510826
AVKVTLGPKGRNVVL	60 kDa chaperonin 2 P0A521	-0,21528552
ASIFAWTRGLQHRGK	CAA17111.1; PROBABLE ISOCITRATE DEHYDROGENASE	-0,216001118
NGGEYTVFAPTNAAF	CAA98350.1; CELL SURFACE LIPOPROTEIN MPT83	-0,216039621
VEAITPQTLINIRPV	RNA POLYMERASE BETA-SUBUNIT AAA21416	-0,216066448
EFPAIPGFPALPGLP	CAB06237.1; CONSERVED HYPOTHETICAL PROTEIN	-0,216548678
MDFGLLPPEVNSSRM	PPE FAMILY PROTEIN CAE55334	-0,217292024
PDALRDGNRIFAVVR	MYCOBACTERIUM BOVIS MYCOCEROSIC ACID SYNTHASE GENE; M95808	-0,219385373
IPSRGAWLEFDVDKR	RNA POLYMERASE BETA-SUBUNIT AAA21416	-0,219801599
MRHTIGQAEQAAMSA	CONSERVED HYPOTHETICAL PROTEIN TB9.8 CAD93159	-0,220094395
PGLIPGVIPTMTPPP	CAA17582.1; PROBABLE SERINE PROTEASE PEPD	-0,22030787
HQVRRVPVISEHRLV	HYPOTHETICAL PROTEIN RV2626C NP_217142	-0,220430411
ALGQTVQASDSLTGA	CAB09453.1; PROBABLE SERINE PROTEASE PEPA	-0,222185544
LVGVDGSSASELATA	HYPOTHETICAL PROTEIN RV2623 NP_217139	-0,222239673
VVATTLALVSAPAGG	PROBABLE CUTINASE PRECURSOR CFP21 NP_216500	-0,22251863
LPDIPIPGLPDIPGL	CAB06237.1; CONSERVED HYPOTHETICAL PROTEIN	-0,222610509
DIVIPAFGGGTAIPI	YP_177963; PPE FAMILY PROTEIN	-0,222994227
VFGGKYVSLTTPKNP	MCE-FAMILY PROTEIN MCE1AYP_177701	-0,223178456
VLSRRLWENAGSPEV	3-OXOACYL-[ACYL-CARRIER PROTEIN] SYNTHASE 2 KASB CAA94642	-0,223933739
TTVLPFNGLYQPQGL	TRANSMEMBRANE SERINE/THREONINE-PROTEIN KINASE D PKND NP_215446	-0,224769015
TSSSQESGVIEEVSA	RNA POLYMERASE BETA-SUBUNIT AAA21416	-0,224876475
AATTDWYRPVAGDFT	CAA17343.1; HEAT SHOCK PROTEIN HSP	-0,225605795
ATQSMAEQVLAERLA	HYPOTHETICAL PROTEIN RV2623 NP_217139	-0,225651955

Peptides	Protein	Average Index
ATSIDPQQRLLLETS	MYCOBACTERIUM BOVIS MYCOCEROSIC ACID SYNTHASE GENE; M95808	-0,227023063
ITTTAGPQLVVPVLN	MTB81 (publication)	-0,227154238
NRVSFAKLREPLEVP	RNA POLYMERASE BETA-SUBUNIT AAA21416	-0,227465034
SYLRNFLAAPPPQRA	LOW MOLECULAR WEIGHT T-CELL ANTIGEN TB8.4 NP_215690	-0,228768923
RNTAVCAPTTGVLPP	CAE55335.1; PE FAMILY PROTEIN	-0,229201156
ARWSPGDSATVAGPL	TRANSMEMBRANE SERINE/THREONINE-PROTEIN KINASE D PKND NP_215446	-0,229627227
YLNQNIAQQLRAQVM	CAB06237.1; CONSERVED HYPOTHETICAL PROTEIN	-0,230275818
TAEARTPAHAAHHLV	POSSIBLE HEMOLYSIN-LIKE PROTEIN CAA17201	-0,230397368
PVDDAFIAALNNAGV	CONSERVED HYPOTHETICAL PROTEIN CAA15739	-0,231120165
LPDIIDGLWPPKLPD	CAB06237.1; CONSERVED HYPOTHETICAL PROTEIN	-0,231248692
VAQANSPIDADGRFV	RNA POLYMERASE BETA-SUBUNIT AAA21416	-0,232101532
RDYLTDLFPILELGT	PROBABLE ISOCITRATE DEHYDROGENASE CAA16247	-0,232280859
LNDLAQIKAGDKVLI	MYCOBACTERIUM BOVIS MYCOCEROSIC ACID SYNTHASE GENE; M95808	-0,233009767
FPVTVNWSTPAVTIF	YP_177963; PPE FAMILY PROTEIN	-0,233157146
ADGKAMLFDGRSGEP	RNA POLYMERASE BETA-SUBUNIT AAA21416	-0,234203903
FNAGDTNTGGFNPGS	YP_177963; PPE FAMILY PROTEIN	-0,234863847
SYSYSLDVPRITILD	YP_177963; PPE FAMILY PROTEIN	-0,235029022
DAAAMFGYAAATATA	CAE55371.1; PPE FAMILY PROTEIN	-0,235163618
GRMCPIETPEGPNIG	RNA POLYMERASE BETA-SUBUNIT AAA21416	-0,236026235
LTRLVDAADPEVVFV	CONSERVED HYPOTHETICAL PROTEIN CAB08634	-0,236233194
SIPNLFPGLPGLGDL	CAB06237.1; CONSERVED HYPOTHETICAL PROTEIN	-0,236315736
LYRLYRPLIFALYCL	POSSIBLE GLYCOSYL TRANSFERASE CAB05418	-0,236497837
HVTSEAIAASYDAGV	CAA17343.1; HEAT SHOCK PROTEIN HSP	-0,236822325
DDLMVNNAGLVCGGV	CAA98350.1; CELL SURFACE LIPOPROTEIN MPT83	-0,236889271
TVTGIDKVQPAVFAV	MYCOBACTERIUM BOVIS MYCOCEROSIC ACID SYNTHASE GENE; M95808	-0,236996091
LFNAGGFTTGLANAG	YP_177963; PPE FAMILY PROTEIN	-0,237457877
PSEDAQVAVYRAALA	MYCOBACTERIUM BOVIS MYCOCEROSIC ACID SYNTHASE GENE; M95808	-0,237474881
AAAQRRKIDRLLDVA	PUTATIVE CYCLOPROPANE-FATTY-ACYL-PHOSPHOLIPID SYNTHASE UFAA1 NP_854118	-0,237701458
GGGLPADTAAQLTSA	MTB48 AAK31576	-0,238045595
GLTVGSWIGSSAGLM	CAE55371.1; PPE FAMILY PROTEIN	-0,23811435
LNAQTGVYEDLLAAG	60 kDa chaperonin 2 P0A521	-0,238948815
LNYTANGSGAGISEF	PERIPLASMIC PHOSPHATE-BINDING LIPOPROTEIN PSTS3 YP_177768	-0,23980766
VELPAKQVNSELMAR	MYCOBACTERIUM BOVIS MYCOCEROSIC ACID SYNTHASE GENE; M95808	-0,240016635
GGAPNPYTYPENLPR	MCE-FAMILY PROTEIN MCE1AYP_177701	-0,240132256
LVAANRSRLMSLVAA	PPE FAMILY PROTEIN CAE55334	-0,241389277
VIRLSDYPYVVPLID	CONSERVED HYPOTHETICAL PROTEIN CAB08634	-0,242062147
DIYALACVLGECLTG	TRANSMEMBRANE SERINE/THREONINE-PROTEIN KINASE D PKND NP_215446	-0,242133179
VSAATATGLPLRGVL	MYCOBACTERIUM BOVIS MYCOCEROSIC ACID SYNTHASE GENE; M95808	-0,242867123
VQGMSQDPVAVAASN	CAA98373.1; MAJOR SECRETED IMMUNOGENIC PROTEIN	-0,243496944
TLAQEAGNFERISGD	CAA17966.1; 10 KDA CULTURE FILTRATE ANTIGEN ESXB	-0,244390068
LAGDAWHGPASLAMT	PPE FAMILY PROTEIN CAE55504	-0,245109377
PRQMVSVATAMIPFL	RNA POLYMERASE BETA-SUBUNIT AAA21416	-0,245934551
STGTTVLPFNGLYQP	TRANSMEMBRANE SERINE/THREONINE-PROTEIN KINASE D PKND NP_215446	-0,246196511
PSASELAAELGMDRA	ALTERNATE RNA POLYMERASE SIGMA FACTOR SIGF CAB07069	-0,246326212
VLLAVRGLRMGTGTS	MYCOBACTERIUM BOVIS MYCOCEROSIC ACID SYNTHASE GENE; M95808	-0,246456172
LGWKVSDLKSSTAVI	CAB06500.1; IMMUNOGENIC PROTEIN MPT63	-0,246921954
DAPIRDWVKLAVTRA	PROBABLE ISOCITRATE DEHYDROGENASE CAA16247	-0,247108732
LAVQATPTFSSSAGA	PPE FAMILY PROTEIN CAE55334	-0,247744
DVSGGLPAFTLFPGG	YP_177963; PPE FAMILY PROTEIN	-0,247851784
TPREAPYELNITSAT	CAA98382.1; IMMUNOGENIC PROTEIN MPT64	-0,247869174
MGTGTSERDERDRLV	MYCOBACTERIUM BOVIS MYCOCEROSIC ACID SYNTHASE GENE; M95808	-0,248056831
PRRMNIGQILETHLG	RNA POLYMERASE BETA-SUBUNIT AAA21416	-0,248642978

Peptides	Protein	Average Index
LPLPGGTTTLVEHMV	CAA16030.1; PROBABLE MOLYBDOPTERIN-GUANINE DINUCLEOTIDE BIOSYNTHESIS PROTEIN	-0,249012513
RRPTYVFAAVDHTGA	CONSERVED HYPOTHETICAL PROTEIN CAB08634	-0,249189207
AELVKEVAKKTDDVA	60 kDa chaperonin 2 P0A521	-0,249263037
FMSAGDLAIAAHDAL	TRANSMEMBRANE SERINE/THREONINE-PROTEIN KINASE D PKND NP_215446	-0,250673541
IRGTFKSVAVKAPGF	60 kDa chaperonin 2 P0A521	-0,250865877
IIPTLNVAAVLPACL	POSSIBLE GLYCOSYL TRANSFERASE CAB05419	-0,250946124
NAGSYNTGSLNAGNT	YP_177963; PPE FAMILY PROTEIN	-0,251392723
GLYNTGGLPPGTPAV	YP_177963; PPE FAMILY PROTEIN	-0,251437181
YTSGSTRTPAGVVMS	MYCOBACTERIUM BOVIS ACYL-COA SYNTHASE GENE; U75685	-0,25189353
DVFDRGGPYLQRGVA	MCE-FAMILY PROTEIN MCE1AYP_177701	-0,252797117
AHLTGNAAMGAFTIP	YP_177963; PPE FAMILY PROTEIN	-0,253412638
TPALNIHQIPLGFQV	YP_177963; PPE FAMILY PROTEIN	-0,253710301
DRVDTLVGAGERKMS	CAA16030.1; PROBABLE MOLYBDOPTERIN-GUANINE DINUCLEOTIDE BIOSYNTHESIS PROTEIN	-0,253975274
QAIFYRRGLFGTIGP	POSSIBLE GLYCOSYL TRANSFERASE CAB05419	-0,25426139
LATYAFLPIVRAFAE	PROBABLE ISOCITRATE DEHYDROGENASE CAA16247	-0,255248357
PGIDWPATQSMAEQV	HYPOTHETICAL PROTEIN RV2623 NP_217139	-0,255254707
LEGTYRLAPKGSGTE	CONSERVED HYPOTHETICAL PROTEIN TB16.3CAD97060	-0,255337141
RVTGRASARGLIFVI	POSSIBLE HEMOLYSIN CAA16235	-0,25609627
PTDRPIVYATLGTSG	POSSIBLE GLYCOSYL TRANSFERASE CAB05415	-0,256259343
HQIPLGFQVPGSIDA	YP_177963; PPE FAMILY PROTEIN	-0,256392468
ALAAAAEVFGEAAEV	MYCOBACTERIUM BOVIS MYCOCEROSIC ACID SYNTHASE GENE; M95808	-0,256860815
MAKLARVVGLVQEEQ	CAA17582.1; PROBABLE SERINE PROTEASE PEPD	-0,25692159
SAAIGLSMAGSSAMI	CAB10044.1; SECRETED ANTIGEN 85-B FBPB (85B)	-0,257104423
LPVSAIAWGAWGEVG	MYCOBACTERIUM BOVIS MYCOCEROSIC ACID SYNTHASE GENE; M95808	-0,257136838
KTLKASGSTAQANAM	PERIPLASMIC PHOSPHATE-BINDING LIPOPROTEIN PSTS3 YP_177768	-0,257378278
RLLAKLGMERRTQAA	TWO COMPONENT TRANSCRIPTIONAL REGULATORY PROTEIN DEVR NP_217649	-0,257460193
DHHGLVGFSYSIEIT	YP_177963; PPE FAMILY PROTEIN	-0,257875604
MPPGSVEQVAAKVVP	CAA17582.1; PROBABLE SERINE PROTEASE PEPD	-0,258045382
FLNNETDFAGSDVPL	PERIPLASMIC PHOSPHATE-BINDING LIPOPROTEIN PSTS2 YP_177769	-0,258048323
DVDWAEVAADLQQGA	PPE FAMILY PROTEIN CAE55504	-0,258893838
IKGMELARAVKDVGA	TWO COMPONENT TRANSCRIPTIONAL REGULATORY PROTEIN DEVR NP_217649	-0,259081588
SVVATGAASLLTVDM	CONSERVED HYPOTHETICAL PROTEIN TB18.5CAD93033	-0,259349902
HGMLTDRDIVIKGLA	HYPOTHETICAL PROTEIN RV2626C NP_217142	-0,25981802
VRVAAAAYETAYGLT	CAE55371.1; PPE FAMILY PROTEIN	-0,26050592
TIMDFEDSVAAVDAA	MTB81 (publication)	-0,26080098
FGYPIPLFPAVGADV	YP_177963; PPE FAMILY PROTEIN	-0,260803709
GIRLTPKVIATNKTA	MYCOBACTERIUM BOVIS MYCOCEROSIC ACID SYNTHASE GENE; M95808	-0,260883665
GFDNWEGDAATACEA	MTB48 AAK31576	-0,261047313
RRLKELHLRLGTATA	ALTERNATE RNA POLYMERASE SIGMA FACTOR SIGF CAB07069	-0,262089809
EYLQVPSPSMGRDIK	CAB10044.1; SECRETED ANTIGEN 85-B FBPB (85B)	-0,262186568
LGDVYADAAPDLFDF	MCE-FAMILY PROTEIN MCE1AYP_177701	-0,262249045
SEIRMTSGSSSGFKV	YP_177963; PPE FAMILY PROTEIN	-0,262505674
QLVRSPGVYFDETID	RNA POLYMERASE BETA-SUBUNIT AAA21416	-0,262537047
APNAAPEPVARRQRI	POSSIBLE GLYCOSYL TRANSFERASE CAB05418	-0,262688822
LDEIAPDLVVGDLRI	POSSIBLE GLYCOSYL TRANSFERASE CAB05415	-0,263761019
SQLASMGSQQAQLIS	CAB06237.1; CONSERVED HYPOTHETICAL PROTEIN	-0,264325695
TIPLNIHQTFSLGPL	YP_177963; PPE FAMILY PROTEIN	-0,265290733
VFATELKRSRPPGDG	TWO COMPONENT TRANSCRIPTIONAL REGULATORY PROTEIN DEVR NP_217649	-0,26598899
DWNPASKRGERESFV	POSSIBLE HEMOLYSIN CAA16235	-0,266579314
SRRNVDLVALHAWSD	HYPOTHETICAL PROTEIN RV2623 NP_217139	-0,266658606
TSSTPREAPYELNIT	CAA98382.1; IMMUNOGENIC PROTEIN MPT64	-0,267914552
WLLSVLAAVGLGLAT	CAB09453.1; PROBABLE SERINE PROTEASE PEPA	-0,268031066
YTLTDEAPLLATYAF	PROBABLE ISOCITRATE DEHYDROGENASE CAA16247	-0,26856781
IEPFIVWTSSGAIGP	YP_177963; PPE FAMILY PROTEIN	-0,268622766

Peptides	Protein	Average Index
YILLVSSKVSTVKDL	60 kDa chaperonin 2 P0A521	-0,26913761
QPGTPGYAQGQQQTY	CAA17582.1; PROBABLE SERINE PROTEASE PEPD	-0,269518943
SVTPAVSQFNARTAD	CAB06500.1; IMMUNOGENIC PROTEIN MPT63	-0,270189119
QQVSIAPNAGLDPVN	CAA98382.1; IMMUNOGENIC PROTEIN MPT64	-0,27062532
KSNMGHSTASAGTVG	MYCOBACTERIUM BOVIS MYCOCEROSIC ACID SYNTHASE GENE; M95808	-0,271361886
EVAGAGSGFANFGSL	YP_177963; PPE FAMILY PROTEIN	-0,271691592
TGIAMTTALATDAET	3-OXOACYL-[ACYL-CARRIER PROTEIN] SYNTHASE 2 KASB CAA94642	-0,27176684
LHALHYHQVDVAAVQ	MTB81 (publication)	-0,272001328
CVAYIGISFLDQASQ	PERIPLASMIC PHOSPHATE-BINDING LIPOPROTEIN PSTS1 YP_177770	-0,272806694
SDPAYNINISLPSYY	CAA98382.1; IMMUNOGENIC PROTEIN MPT64	-0,273437498
RDLWPAPYLVMDTGA	MCE-FAMILY PROTEIN MCE1AYP_177701	-0,273451842
IRDWVKLAVTRARIS	PROBABLE ISOCITRATE DEHYDROGENASE CAA16247	-0,274052349
MNLRRHQTLTLRLLA	CONSERVED HYPOTHETICAL PROTEIN CAA15739	-0,274164873
VLLESMKMEIPVLAE	CAB08316.1; BIOTINYLATED PROTEIN TB7.3	-0,27426449
RAGAGVLLRTERLKS	POSSIBLE GLYCOSYL TRANSFERASE CAB05415	-0,274640316
GAKEVETKEQIAATA	60 kDa chaperonin 2 P0A521	-0,274748475
AIPPRGTQAVVLKVY	CAA98382.1; IMMUNOGENIC PROTEIN MPT64	-0,275055044
AQGLPVSAIAWGAWG	MYCOBACTERIUM BOVIS MYCOCEROSIC ACID SYNTHASE GENE; M95808	-0,275167435
PPQEQGLIPGFLMPP	MTB48 AAK31576	-0,275252122
PTTYDHPTFAVHDTL	SECRETED L-ALANINE DEHYDROGENASE ALD CAA15575	-0,275389024
VSVGDVIQAGDLIAV	CAB08316.1; BIOTINYLATED PROTEIN TB7.3	-0,275567743
ANTVLDDLNSRMPQS	MCE-FAMILY PROTEIN MCE1AYP_177701	-0,276253547
TGNAAMGAFTIPQID	YP_177963; PPE FAMILY PROTEIN	-0,277051017
PIDADGRFVEPRVLV	RNA POLYMERASE BETA-SUBUNIT AAA21416	-0,277174225
PLNFDVLSSAGPINI	YP_177963; PPE FAMILY PROTEIN	-0,278105051
LHFPGFTIPTGPIHI	YP_177963; PPE FAMILY PROTEIN	-0,278447769
TEAVVADKPEKEKAS	60 kDa chaperonin 2 P0A521	-0,278490491
RPIVYATLGTSGGRN	POSSIBLE GLYCOSYL TRANSFERASE CAB05415	-0,279764604
IPGVIPTMTPPPGMV	CAA17582.1; PROBABLE SERINE PROTEASE PEPD	-0,280108334
SWPDHSRDQARRNIA	PUTATIVE CYCLOPROPANE-FATTY-ACYL-PHOSPHOLIPID SYNTHASE UFAA1 NP_854118	-0,281073177
PQPSQLRPGRVPPAL	TRANSMEMBRANE SERINE/THREONINE-PROTEIN KINASE D PKND NP_215446	-0,281319292
PLADHIARRFEGRGE	ALTERNATE RNA POLYMERASE SIGMA FACTOR SIGF CAB07069	-0,281373659
MRESFAERGVTLGAA	MYCOBACTERIUM BOVIS MYCOCEROSIC ACID SYNTHASE GENE; M95808	-0,281519369
NAGSTNVGLANMGVG	YP_177963; PPE FAMILY PROTEIN	-0,282070699
STLTEEDVVATIEYL	RNA POLYMERASE BETA-SUBUNIT AAA21416	-0,282478319
HVENALRLMPENRPR	POSSIBLE GLYCOSYL TRANSFERASE CAB05415	-0,282562859
DDTVGRVKVYEAIVK	RNA POLYMERASE BETA-SUBUNIT AAA21416	-0,283308746
YAGLTAANTKKVAMA	POSSIBLE GLYCOSYL TRANSFERASE CAB05419	-0,284479961
NSGTDLPPTPISVIF	PERIPLASMIC PHOSPHATE-BINDING LIPOPROTEIN PSTS2 YP_177769	-0,284509956
LMSVTQPDRVRELLA	MYCOBACTERIUM BOVIS MYCOCEROSIC ACID SYNTHASE GENE; M95808	-0,284597395
QSGSIGLGFAIPVDQ	CAA17582.1; PROBABLE SERINE PROTEASE PEPD	-0,285275951
DDLGVNVNNGLSDLY	PROBABLE ISOCITRATE DEHYDROGENASE CAA16247	-0,285677857
YAFLPIVRAFAEPAG	PROBABLE ISOCITRATE DEHYDROGENASE CAA16247	-0,285828488
GLWKTVSPHRSPISN	CAE55371.1; PPE FAMILY PROTEIN	-0,28618684
ASKTPANQAISMIDG	PERIPLASMIC PHOSPHATE-BINDING LIPOPROTEIN PSTS1 YP_177770	-0,286668692
ISTTPDAAPGLAARR	POSSIBLE GLYCOSYL TRANSFERASE CAB05415	-0,28697696
VGATSEIEPFIVWTS	YP_177963; PPE FAMILY PROTEIN	-0,287526272
GLREVADGDALYDAA	MYCOBACTERIUM BOVIS MYCOCEROSIC ACID SYNTHASE GENE; M95808	-0,287560729
ADMLSELGEPVDRVA	CONSERVED HYPOTHETICAL PROTEIN CAB08634	-0,289522384
QTYGVRPGAVVGHSM	MYCOBACTERIUM BOVIS MYCOCEROSIC ACID SYNTHASE GENE; M95808	-0,289883486
VPDNLAELGRLTQLP	PROBABLE ISOCITRATE DEHYDROGENASE CAA16247	-0,290057251
QAAPPALSQDRFADF	CAB09453.1; PROBABLE SERINE PROTEASE PEPA	-0,290226698
EDRQSLSWTLESSSL	CONSERVED HYPOTHETICAL PROTEIN TB16.3CAD97060	-0,290311014

Peptides	Protein	Average Index
NEFNVVDVGSLNGTY	CAB01474.1; CONSERVED HYPOTHETICAL PROTEIN CFP17	-0,290351938
FKVRPSFSFFAVGPD	YP_177963; PPE FAMILY PROTEIN	-0,290613773
APKHVKQLVEENHLR	PROBABLE ISOCITRATE DEHYDROGENASE CAA16247	-0,290874209
PPEVNSARIFAGAGL	PPE FAMILY PROTEIN CAE55504	-0,291100018
TPEVIDFAHKLESVV	CAA17111.1; PROBABLE ISOCITRATE DEHYDROGENASE	-0,291696502
VDMANPMSPVNKSFE	LIPOPROTEIN LPQH PRECURSOR P0A5J0	-0,29184029
AGQASPSRIDGTHQT	CAA98350.1; CELL SURFACE LIPOPROTEIN MPT83	-0,291964307
NATVYMIDSVLMPPA	CAA98373.1; MAJOR SECRETED IMMUNOGENIC PROTEIN	-0,292458831
KGLMNIALAISAQQV	PERIPLASMIC PHOSPHATE-BINDING LIPOPROTEIN PSTS1 YP_177770	-0,292758238
STNNDDPAGACRPFD	3-OXOACYL-[ACYL-CARRIER PROTEIN] SYNTHASE 2 KASB CAA94642	-0,292919162
MREHDIGALPICGDD	HYPOTHETICAL PROTEIN RV2626C NP_217142	-0,293141725
AQTEVAGAGSGFANF	YP_177963; PPE FAMILY PROTEIN	-0,294237372
ISVTWQTKSGGTRTG	CAB09453.1; PROBABLE SERINE PROTEASE PEPA	-0,294268483
QEITRGRCAAISVPG	MYCOBACTERIUM BOVIS ACYL-COA SYNTHASE GENE; U75685	-0,294407147
LDPSQGMGPSLIGLA	CAB10044.1; SECRETED ANTIGEN 85-B FBPB (85B)	-0,294498924
DIKATYDKGILTVSV	CAA17245.1; HEAT SHOCK PROTEIN HSPX	-0,294623427
LRAAGADIVVECGNI	MYCOBACTERIUM BOVIS MYCOCEROSIC ACID SYNTHASE GENE; M95808	-0,294767397
AGVTCVGEHETLTAA	HYPOTHETICAL PROTEIN RV2626C NP_217142	-0,295690683
LRDGNRIFAVVRGTA	MYCOBACTERIUM BOVIS MYCOCEROSIC ACID SYNTHASE GENE; M95808	-0,296226529
PGSIDAITLFPGGLT	YP_177963; PPE FAMILY PROTEIN	-0,296466059
IQMSDPAYNINISLP	CAA98382.1; IMMUNOGENIC PROTEIN MPT64	-0,29667051
AESNNQVVLPFTDIT	TRANSMEMBRANE SERINE/THREONINE-PROTEIN KINASE D PKND NP_215446	-0,297098607
VLIAFGRFPIIDDRE	MYCOBACTERIUM BOVIS MYCOCEROSIC ACID SYNTHASE GENE; M95808	-0,297325731
DAYRQFLTEIGYLLP	MTB81 (publication)	-0,297380696
NDDPAGACRPFDRDR	3-OXOACYL-[ACYL-CARRIER PROTEIN] SYNTHASE 2 KASB CAA94642	-0,297851906
EIVPAAAVPTLVDMS	HYPOTHETICAL PROTEIN RV2623 NP_217139	-0,298532608
GEVMKAIADIEAYPQ	CONSERVED HYPOTHETICAL PROTEIN TB16.3CAD97060	-0,298597724
LRVGQPVLAIGSPLG	CAA17582.1; PROBABLE SERINE PROTEASE PEPD	-0,298959825
DAMARLGAIKREVTS	MYCOBACTERIUM BOVIS ACYL-COA SYNTHASE GENE; U75685	-0,299126203
AGVPVIGLPSNMDQH	POSSIBLE GLYCOSYL TRANSFERASE CAB05418	-0,299995465
LGGGVGAFLNALFAG	YP_177963; PPE FAMILY PROTEIN	-0,300304817
FNAASQAFNTPALNI	YP_177963; PPE FAMILY PROTEIN	-0,300905128
VTSIHSLLDEGKQSL	CAE55648	-0,301434299
ADMVETKIAQPRAGA	MTB81 (publication)	-0,301628129
TVPANAFVADYLPGE	POSSIBLE GLYCOSYL TRANSFERASE CAB05415	-0,30167776
ARGANILARIMGASI	3-OXOACYL-[ACYL-CARRIER PROTEIN] SYNTHASE 2 KASB CAA94642	-0,301949212
GSDSSMRAVDRAAQI	CAB08889.1; IRON-REGULATED CONSERVED HYPOTHETICAL PROTEIN	-0,302032813
LLPFEEAPEMTSAGG	CAE55371.1; PPE FAMILY PROTEIN	-0,302550158
RWLDPRHAGPATAAD	CAA16102.1; ESAT-6 LIKE PROTEIN ESXQ	-0,302685519
VKEVAKKTDDVAGDG	60 kDa chaperonin 2 P0A521	-0,302818206
VHYDLSNDLFAAFLD	PUTATIVE CYCLOPROPANE-FATTY-ACYL-PHOSPHOLIPID SYNTHASE UFAA1 NP_854118	-0,303521925
LFVGEAATLAHVVRP	POSSIBLE GLYCOSYL TRANSFERASE CAB05418	-0,30352505
NGDEVQIGKFRLVFL	CAB01474.1; CONSERVED HYPOTHETICAL PROTEIN CFP17	-0,303707812
TQLAPIQEGLQQQLQ	PPE FAMILY PROTEIN CAE55504	-0,303827731
VTLGPKGRNVVLEKK	60 kDa chaperonin 2 P0A521	-0,304320798
LYQGATISSTKIDGV	CONSERVED HYPOTHETICAL PROTEIN CAB08634	-0,304505221
QAARRLAEAFGPDFA	POSSIBLE GLYCOSYL TRANSFERASE CAB05418	-0,304959639
MPPSDGSGVTPGTGM	MTB48 AAK31576	-0,305090189
NITSATYQSAIPPRG	CAA98382.1; IMMUNOGENIC PROTEIN MPT64	-0,305987146
GKYVSLTTPKNPTKR	MCE-FAMILY PROTEIN MCE1AYP_177701	-0,306005592
DKRDTVGVRIDRKRR	RNA POLYMERASE BETA-SUBUNIT AAA21416	-0,306892903
ASIAGLFLTTEAVVA	60 kDa chaperonin 2 P0A521	-0,307104114
MTVPPSLVAANRSRL	PPE FAMILY PROTEIN CAE55334	-0,307209293
VDAVINTTCNYGQVV	LOW MOLECULAR WEIGHT T-CELL ANTIGEN TB8.4 NP_215690	-0,307606723
IAGAKIMGQGNDLVL	PERIPLASMIC PHOSPHATE-BINDING LIPOPROTEIN PSTS2	-0,307675972

Peptides	Protein	Average Index
	YP_177769	
KLLAGSTTSTVLPFT	TRANSMEMBRANE SERINE/THREONINE-PROTEIN KINASE D PKND NP_215446	-0,307804265
DETLDIANIFAPNLG	POSSIBLE GLYCOSYL TRANSFERASE CAB05419	-0,308127651
AGFGHRVEIDLCDYR	PUTATIVE CYCLOPROPANE-FATTY-ACYL-PHOSPHOLIPID SYNTHASE UFAA1 NP_854118	-0,309083993
YRQAAARLADAFGRD	POSSIBLE GLYCOSYL TRANSFERASE CAB05415	-0,309166664
ERAGLEVRDVHPSHY	RNA POLYMERASE BETA-SUBUNIT AAA21416	-0,309334996
AFLGLGVVDNNGNGA	CAB09453.1; PROBABLE SERINE PROTEASE PEPA	-0,30959663
SADALVAAVRSKAPG	CAA17582.1; PROBABLE SERINE PROTEASE PEPD	-0,309909785
GRIAVPLSVPQGGVT	MYCOBACTERIUM BOVIS ACYL-COA SYNTHASE GENE; U75685	-0,31028006
RVQGVSGLTPISLGS	CAA17582.1; PROBABLE SERINE PROTEASE PEPD	-0,310353426
AKRIADELISTGKAS	CAA17582.1; PROBABLE SERINE PROTEASE PEPD	-0,310364935
VAVSMSSINFADVLI	MYCOBACTERIUM BOVIS MYCOCEROSIC ACID SYNTHASE GENE; M95808	-0,310559615
GVYAVNYPASDDYRA	PROBABLE CUTINASE PRECURSOR CFP21 NP_216500	-0,31085694
GSEVFEGIMDALFTG	MTB81 (publication)	-0,311051358
GDMAGYTGTTQSLGA	CAA16102.1; ESAT-6 LIKE PROTEIN ESXQ	-0,31139554
RLTQLPDTNIIKLPN	PROBABLE ISOCITRATE DEHYDROGENASE CAA16247	-0,312064589
PANAFVADYLPGEAA	POSSIBLE GLYCOSYL TRANSFERASE CAB05418	-0,312491382
NGEEYLILSARDVLA	CAB01005.1; 10 KDA CHAPERONIN GROES	-0,312915914
VVGGALLYLTIMTLM	CAB06237.1; CONSERVED HYPOTHETICAL PROTEIN	-0,31348225
MNFAVLPPEVNSARI	PPE FAMILY PROTEIN CAE55504	-0,313607824
GTGLGSAEELVFSYD	3-OXOACYL-[ACYL-CARRIER PROTEIN] SYNTHASE 2 KASB CAA94642	-0,313627094
RSVGVPLYLLLGWVA	POSSIBLE HEMOLYSIN-LIKE PROTEIN CAA17201	-0,313742456
AGQIRSGGGSPTVHI	CAB09453.1; PROBABLE SERINE PROTEASE PEPA	-0,314864584
LAAPPPQRAAMAAQL	LOW MOLECULAR WEIGHT T-CELL ANTIGEN TB8.4 NP_215690	-0,314976089
IAITYNIKGVSTLNL	PERIPLASMIC PHOSPHATE-BINDING LIPOPROTEIN PSTS2 YP_177769	-0,315371944
GFAIPIGQAMAIAGQ	CAB09453.1; PROBABLE SERINE PROTEASE PEPA	-0,315908083
HEANTMAMMARDTAE	CAA17363.1; LOW MOLECULAR WEIGHT PROTEIN ANTIGEN 7 ESXH	-0,316341861
AGILFLHSGLIALPP	PPE FAMILY PROTEIN CAE55504	-0,316421221
SPGDSATVAGPLAAD	TRANSMEMBRANE SERINE/THREONINE-PROTEIN KINASE D PKND NP_215446	-0,316747796
ITPVHPGYTATFLET	PPE FAMILY PROTEIN CAE55504	-0,317102105
SGIAGAGTGPPTPGA	TRANSMEMBRANE SERINE/THREONINE-PROTEIN KINASE D PKND NP_215446	-0,317117294
DGWNTFNLTLQGDVK	MTB48 AAK31576	-0,317249536
SAGVGHAALVGALSV	PPE FAMILY PROTEIN CAE55334	-0,31726059
SPIEDFSGSMSLSFS	RNA POLYMERASE BETA-SUBUNIT AAA21416	-0,317263109
GRNLLQLVLNALAEL	POSSIBLE GLYCOSYL TRANSFERASE CAB05415	-0,317309225
DDQQLAEHFASLADV	PROBABLE ISOCITRATE DEHYDROGENASE CAA16247	-0,317372642
DKLWESLLRGDDLVT	MYCOBACTERIUM BOVIS MYCOCEROSIC ACID SYNTHASE GENE; M95808	-0,317748081
GRAASVGSLSVPQAW	CAE55371.1; PPE FAMILY PROTEIN	-0,317939763
FGPVVVPPIPVLAIN	YP_177963; PPE FAMILY PROTEIN	-0,318302235
EKVTETLLKGAKEVE	60 kDa chaperonin 2 P0A521	-0,318716937
ADAIRAMSNAEHTGK	MYCOBACTERIUM BOVIS MYCOCEROSIC ACID SYNTHASE GENE; M95808	-0,319251221
TLLAVLTAAPLLLAA	PERIPLASMIC PHOSPHATE-BINDING LIPOPROTEIN PSTS1 YP_177770	-0,319309267
TGNMISNQAKYVSDT	CAB06237.1; CONSERVED HYPOTHETICAL PROTEIN	-0,319549482
ASAYLPQHEDARAAD	CAB08889.1; IRON-REGULATED CONSERVED HYPOTHETICAL PROTEIN	-0,319811041
EAPYELNITSATYQS	CAA98382.1; IMMUNOGENIC PROTEIN MPT64	-0,31990998
LFNAGSFNTGLANAG	YP_177963; PPE FAMILY PROTEIN	-0,319927709
LGWTSEQIVERFGFS	RNA POLYMERASE BETA-SUBUNIT AAA21416	-0,319972494
VFVMAAPGQPLPTLP	CAA16030.1; PROBABLE MOLYBDOPTERIN-GUANINE DINUCLEOTIDE BIOSYNTHESIS PROTEIN	-0,320249258
ADFPALPLDPSAMVA	CAB09453.1; PROBABLE SERINE PROTEASE PEPA	-0,320483185
PDTAKEKPQEGTVVA	CAB01005.1; 10 KDA CHAPERONIN GROES	-0,32064721
SPAVPNALQASLGHA	TRANSMEMBRANE SERINE/THREONINE-PROTEIN KINASE D PKND NP_215446	-0,320724516
LYGTDVIPETDGAEK	MTB81 (publication)	-0,3210012

Peptides	Protein	Average Index
TVLPIQILPTIPLNI	YP_177963; PPE FAMILY PROTEIN	-0,321076829
LGEGIPDPGDIFNTG	CAB06237.1; CONSERVED HYPOTHETICAL PROTEIN	-0,321109584
PETINYRTLKPEKDG	RNA POLYMERASE BETA'-SUBUNIT AAA21417	-0,321138829
TIPAITFPEIPANAD	YP_177963; PPE FAMILY PROTEIN	-0,321269519
AMFGYAAATATATAT	CAE55371.1; PPE FAMILY PROTEIN	-0,321328084
IAVVSAGIGGAAASL	CAA17582.1; PROBABLE SERINE PROTEASE PEPD	-0,321515781
VPDYPADPKTDQEKA	PROBABLE ISOCITRATE DEHYDROGENASE CAA16247	-0,321543539
SPGDAEVGPRLFMLS	MYCOBACTERIUM BOVIS MYCOCEROSIC ACID SYNTHASE GENE; M95808	-0,322466257
VLMYSGAGSSPLLAA	YP_177963; PPE FAMILY PROTEIN	-0,322601516
AVKPTGSAAIGLSMA	CAB10044.1; SECRETED ANTIGEN 85-B FBPB (85B)	-0,322685088
AVNGTAMRESFAERG	MYCOBACTERIUM BOVIS MYCOCEROSIC ACID SYNTHASE GENE; M95808	-0,322754178
IAEAMDKVGNEGVIT	60 kDa chaperonin 2 P0A521	-0,323269501
FDSMTQTQIAERVGI	ALTERNATE RNA POLYMERASE SIGMA FACTOR SIGF CAB07069	-0,323323866
DTDGSEVFEGIMDAL	MTB81 (publication)	-0,323858187
LTKNEDVIVRELTEV	PROBABLE ISOCITRATE DEHYDROGENASE CAA16247	-0,324565856
VFAPTNAAFDKLPAA	CAA98350.1; CELL SURFACE LIPOPROTEIN MPT83	-0,325357981
RPSWSPTQASGQTVL	TRANSMEMBRANE SERINE/THREONINE-PROTEIN KINASE D PKND NP_215446	-0,325457245
TATILHNAGVTALVL	POSSIBLE HEMOLYSIN-LIKE PROTEIN CAA17201	-0,325617948
AWGKGASETFNGGVG	PERIPLASMIC PHOSPHATE-BINDING LIPOPROTEIN PSTS2 YP_177769	-0,325656012
FALLALPAHDGRVVL	POSSIBLE HEMOLYSIN-LIKE PROTEIN CAA17201	-0,325915869
ELAWAPDEIREEVDN	MTB81 (publication)	-0,32605642
GLERHAKAGVMTPVS	3-OXOACYL-[ACYL-CARRIER PROTEIN] SYNTHASE 2 KASB CAA94642	-0,326132765
HACVAASAQGMLSST	MYCOBACTERIUM BOVIS MYCOCEROSIC ACID SYNTHASE GENE; M95808	-0,326295996
PGNVNTGWLNTGNTN	YP_177963; PPE FAMILY PROTEIN	-0,326337066
NNLGIGLTGDNQTGF	YP_177963; PPE FAMILY PROTEIN	-0,326367772
FVRNVGHLMTNDAIV	MTB81 (publication)	-0,326407841
LTIPGLSLDAAVSEI	YP_177963; PPE FAMILY PROTEIN	-0,326642526
DPRFNKLLGPLPFPH	POSSIBLE GLYCOSYL TRANSFERASE CAB05418	-0,3267025
EAYQGVQQKWDATAT	CAE55648	-0,327428099
VDQAKRIADELISTG	CAA17582.1; PROBABLE SERINE PROTEASE PEPD	-0,327512504
DRARNVRMELLAKSG	PROBABLE ISOCITRATE DEHYDROGENASE CAA16247	-0,327528003
NGISMSEVVPIDVSV	YP_177963; PPE FAMILY PROTEIN	-0,327950001
EFTPKTQLTMLSARA	MCE-FAMILY PROTEIN MCE1AYP_177701	-0,327984684
PVLPPEINSVLMYSG	YP_177963; PPE FAMILY PROTEIN	-0,328010466
AVEEASDTAAANQLM	CAE55371.1; PPE FAMILY PROTEIN	-0,328030119
LAKETATQARAAAEA	PPE FAMILY PROTEIN CAE55334	-0,328633073
EIVCSKYPDSQVGTA	PERIPLASMIC PHOSPHATE-BINDING LIPOPROTEIN PSTS3 YP_177768	-0,32914225
TQIAERVGISQMHVS	ALTERNATE RNA POLYMERASE SIGMA FACTOR SIGF CAB07069	-0,329307937
ALSGLGLPPPWQPAL	PPE FAMILY PROTEIN CAE55504	-0,329734964
IVDAGDRVEAGQVIA	RNA POLYMERASE BETA-SUBUNIT AAA21416	-0,329747237
TIFPGGFTIDPLPLS	YP_177963; PPE FAMILY PROTEIN	-0,330227401
HGTVLLAVRGLRMGT	MYCOBACTERIUM BOVIS MYCOCEROSIC ACID SYNTHASE GENE; M95808	-0,330287571
VIWTRLFGVRLVKLL	POSSIBLE GLYCOSYL TRANSFERASE CAB05415	-0,330769481
GARAGGGLSGVLRVP	CAE55371.1; PPE FAMILY PROTEIN	-0,330862773
TVFKLTADGVLTAPQ	MYCOBACTERIUM BOVIS MYCOCEROSIC ACID SYNTHASE GENE; M95808	-0,33098188
EYVVAFLGALQAGRI	MYCOBACTERIUM BOVIS ACYL-COA SYNTHASE GENE; U75685	-0,331175009
SLTALSAGVGAVAMS	LOW MOLECULAR WEIGHT T-CELL ANTIGEN TB8.4 NP_215690	-0,331387365
YGSFRRSFRLPAHVT	CAA17343.1; HEAT SHOCK PROTEIN HSP	-0,332068636
SEGGCWRTFLTCDAN	MYCOBACTERIUM BOVIS MYCOCEROSIC ACID SYNTHASE GENE; M95808	-0,332130677
GHAGDMAGYAGTLQS	CAA17363.1; LOW MOLECULAR WEIGHT PROTEIN ANTIGEN 7 ESXH	-0,332219395
RELTEVQGEPVDIGG	PROBABLE ISOCITRATE DEHYDROGENASE CAA16247	-0,33258598
TPVFDGAQEAELQGL	RNA POLYMERASE BETA-SUBUNIT AAA21416	-0,332853232
RCWPRSRCPRRSGSS	MYCOBACTERIUM BOVIS MYCOCEROSIC ACID SYNTHASE GENE; M95808	-0,333020086
NMQRQAVPLVRSEAP	RNA POLYMERASE BETA-SUBUNIT AAA21416	-0,333035859

Peptides	Protein	Average Index
AFGRFPIIDDREPQL	MYCOBACTERIUM BOVIS MYCOCEROSIC ACID SYNTHASE GENE; M95808	-0,333189048
FGNLLSFASLPTVGQ	CAB06237.1; CONSERVED HYPOTHETICAL PROTEIN	-0,333374737
EPNFMDLKEAARKLE	MTB48 AAK31576	-0,333474886
AVRNAKAAVEEGIVA	60 kDa chaperonin 2 P0A521	-0,333727446
ASVTTGLAGDAWHGP	PPE FAMILY PROTEIN CAE55504	-0,333828184
SGIPTLSTTGPVHAV	YP_177963; PPE FAMILY PROTEIN	-0,33417148
ATFLETPSQFFPFTG	PPE FAMILY PROTEIN CAE55504	-0,334250261
REPIVISNVPRLVPG	CAA17111.1; PROBABLE ISOCITRATE DEHYDROGENASE	-0,334406098
ALPEVGDGEAGSWLL	MYCOBACTERIUM BOVIS MYCOCEROSIC ACID SYNTHASE GENE; M95808	-0,334421515
VIPPTLNLVNLDPEI	3-OXOACYL-[ACYL-CARRIER PROTEIN] SYNTHASE 2 KASB CAA94642	-0,334506866
SFSDPRFDDVKAPVD	RNA POLYMERASE BETA-SUBUNIT AAA21416	-0,334512898
ANRSRLMSLVAANIL	PPE FAMILY PROTEIN CAE55334	-0,33451464
MLSSTSSDALRQTAR	MYCOBACTERIUM BOVIS MYCOCEROSIC ACID SYNTHASE GENE; M95808	-0,334621348
GTAIPISVGPITISP	YP_177963; PPE FAMILY PROTEIN	-0,334658107
LTQSPIINLGLADVG	YP_177963; PPE FAMILY PROTEIN	-0,334898689
GWWNQAHTALAGAGS	YP_177963; PPE FAMILY PROTEIN	-0,335469864
RRCRRALRQIGVLER	CAA16102.1; ESAT-6 LIKE PROTEIN ESXQ	-0,335607625
EIQLAVQATPTFSSS	PPE FAMILY PROTEIN CAE55334	-0,335608403
ATLGSSGGKNLLQVV	POSSIBLE GLYCOSYL TRANSFERASE CAB05418	-0,335797932
VDEAGTVYVTEHNTN	TRANSMEMBRANE SERINE/THREONINE-PROTEIN KINASE D PKND NP_215446	-0,335931754
NISVPDSSVPIIIVP	YP_177963; PPE FAMILY PROTEIN	-0,336253955
LPGTDIDPDSFWAGV	MTB81 (publication)	-0,336316982
AGSNNQTVLPFDGLN	TRANSMEMBRANE SERINE/THREONINE-PROTEIN KINASE D PKND NP_215446	-0,337112845
TNTLSSMLKGFAPAA	CAE55371.1; PPE FAMILY PROTEIN	-0,337553428
FVAAAAKVNTLLDVA	CONSERVED HYPOTHETICAL PROTEIN TB9.8 CAD93159	-0,337646121
RVATAGEPNFMDLKE	MTB48 AAK31576	-0,337759664
TGKIYFDVTGPSPTI	CAB06500.1; IMMUNOGENIC PROTEIN MPT63	-0,337810722
GSRFLLDQAITSAGR	CAB01474.1; CONSERVED HYPOTHETICAL PROTEIN CFP17	-0,338386739
PGGLNIPQNAIPLTI	YP_177963; PPE FAMILY PROTEIN	-0,338566127
HGVPRRMNIGQILET	RNA POLYMERASE BETA-SUBUNIT AAA21416	-0,339219125
MTDMNPDIEKDQTSD	CAB01474.1; CONSERVED HYPOTHETICAL PROTEIN CFP17	-0,339292928
LHAVDQGLQRRDPRW	MYCOBACTERIUM BOVIS MYCOCEROSIC ACID SYNTHASE GENE; M95808	-0,340075892
RPTQPGSYPIVLATY	PERIPLASMIC PHOSPHATE-BINDING LIPOPROTEIN PSTS2 YP_177769	-0,34018939
KGETELTPEERLLRA	RNA POLYMERASE BETA-SUBUNIT AAA21416	-0,340797122
NNQTVLPFDGLNYPE	TRANSMEMBRANE SERINE/THREONINE-PROTEIN KINASE D PKND NP_215446	-0,341183765
MIRAQAASLEAEHQA	PUTATIVE ESAT-6 LIKE PROTEIN ESXN YP_177838	-0,341341983
TSDEVTVETTSVFRA	CAB01474.1; CONSERVED HYPOTHETICAL PROTEIN CFP17	-0,341650394
MRLSLTALSAGVGAV	LOW MOLECULAR WEIGHT T-CELL ANTIGEN TB8.4 NP_215690	-0,341694982
PALAKGLSTHEGALL	SECRETED L-ALANINE DEHYDROGENASE ALD CAA15575	-0,341708479
STAYLQYTSGSTRTP	MYCOBACTERIUM BOVIS ACYL-COA SYNTHASE GENE; U75685	-0,341749171
GTIRNILGGTIFREP	CAA17111.1; PROBABLE ISOCITRATE DEHYDROGENASE	-0,341759277
EVADGDALYDAAVGH	MYCOBACTERIUM BOVIS MYCOCEROSIC ACID SYNTHASE GENE; M95808	-0,341881652
GRCHSFDADADGFVR	MYCOBACTERIUM BOVIS MYCOCEROSIC ACID SYNTHASE GENE; M95808	-0,342107622
CAPVFVMAAPGQPLP	CAA16030.1; PROBABLE MOLYBDOPTERIN-GUANINE DINUCLEOTIDE BIOSYNTHESIS PROTEIN	-0,343048718
LPNISASVPQLVAAI	PROBABLE ISOCITRATE DEHYDROGENASE CAA16247	-0,34305481
LTSILTYHVVAGQTS	CAA98373.1; MAJOR SECRETED IMMUNOGENIC PROTEIN	-0,343123153
SMTNSGVSMTNTLSS	CAE55371.1; PPE FAMILY PROTEIN	-0,343334245
AAIDAATSSSQESGV	RNA POLYMERASE BETA-SUBUNIT AAA21416	-0,343341049
TGASLAPYNHMEVGS	MCE-FAMILY PROTEIN MCE1AYP_177701	-0,343972889
GDQYRATNFKVDQPG	CAA17111.1; PROBABLE ISOCITRATE DEHYDROGENASE	-0,34397389
TYRLAPKGSGTEVTY	CONSERVED HYPOTHETICAL PROTEIN TB16.3CAD97060	-0,344394443
NALAELPVTVIAATA	POSSIBLE GLYCOSYL TRANSFERASE CAB05415	-0,344671757
MAAIATFAAPVALAA	CAB06500.1; IMMUNOGENIC PROTEIN MPT63	-0,344713219

Peptides	Protein	Average Index
QQFIYAGSLSALLDP	CAB10044.1; SECRETED ANTIGEN 85-B FBPB (85B)	-0,344866026
AAARLADAFGRDRVG	POSSIBLE GLYCOSYL TRANSFERASE CAB05415	-0,344962539
EAEHQAIVRDVLAAG	PUTATIVE ESAT-6 LIKE PROTEIN ESXN YP_177838	-0,345197586
QRVAGAVMQVISRSE	POSSIBLE GLYCOSYL TRANSFERASE CAB05415	-0,345209574
ESMLAAAAAWDGVAA	PPE FAMILY PROTEIN CAE55334	-0,345265072
GAYHLMRTQGGRGVL	SECRETED L-ALANINE DEHYDROGENASE ALD CAA15575	-0,345284329
KVGNEGVITVEESNT	60 kDa chaperonin 2 P0A521	-0,345463035
FEETFEVTAAAPVAV	CAB07109.1; PROBABLE 50S RIBOSOMAL PROTEIN L7/L12 RPLL	-0,345655947
LDQVHFQPLPPAVVK	PERIPLASMIC PHOSPHATE-BINDING LIPOPROTEIN PSTS1 YP_177770	-0,346498363
RLADAFGRDRVGFPQ	POSSIBLE GLYCOSYL TRANSFERASE CAB05415	-0,346499995
QSESGIAGAGTGPPT	TRANSMEMBRANE SERINE/THREONINE-PROTEIN KINASE D PKND NP_215446	-0,346526352
PSIIDAAIAELRHLN	YP_177963; PPE FAMILY PROTEIN	-0,346891684
TLPVPVLRDELPGLG	CAA16030.1; PROBABLE MOLYBDOPTERIN-GUANINE DINUCLEOTIDE BIOSYNTHESIS PROTEIN	-0,346891854
NRDFTLPGERIHVVF	PERIPLASMIC PHOSPHATE-BINDING LIPOPROTEIN PSTS3 YP_177768	-0,346966009
NADGELYVIAGDIPL	YP_177963; PPE FAMILY PROTEIN	-0,347630923
RQLDAEFCGRIHTRY	SECRETED L-ALANINE DEHYDROGENASE ALD CAA15575	-0,348003823
EAPMADPPTDVPITP	MTB48 AAK31576	-0,34804104
AITTIMDFEDSVAAV	MTB81 (publication)	-0,348201092
VKVIPSRGAWLEFDV	RNA POLYMERASE BETA-SUBUNIT AAA21416	-0,348236534
VPVLRDELPGLGPLP	CAA16030.1; PROBABLE MOLYBDOPTERIN-GUANINE DINUCLEOTIDE BIOSYNTHESIS PROTEIN	-0,348459634
PEVATWLEVPLPPGV	HYPOTHETICAL PROTEIN RV2623 NP_217139	-0,348621406
SAKVDCGGKKTLKAS	PERIPLASMIC PHOSPHATE-BINDING LIPOPROTEIN PSTS3 YP_177768	-0,348796016
VAGLSGCSSNKSTTG	LIPOPROTEIN LPQH PRECURSOR P0A5J0	-0,349159758
ATAWYGLNDLAQIKA	MYCOBACTERIUM BOVIS MYCOCEROSIC ACID SYNTHASE GENE; M95808	-0,349479402
MKTDAATLAQEAGNF	CAA17966.1; 10 KDA CULTURE FILTRATE ANTIGEN ESXB	-0,349492673
VAAAGYLVLRPSWSP	TRANSMEMBRANE SERINE/THREONINE-PROTEIN KINASE D PKND NP_215446	-0,349742254
ALQASLGHAVPPAGN	TRANSMEMBRANE SERINE/THREONINE-PROTEIN KINASE D PKND NP_215446	-0,350231925
LARGRRHRPVRTAVV	MYCOBACTERIUM BOVIS MYCOCEROSIC ACID SYNTHASE GENE; M95808	-0,350570433
GSERVQAATIKRFAD	MYCOBACTERIUM BOVIS ACYL-COA SYNTHASE GENE; U75685	-0,350958305
SHVRLTEEPERHVWQ	MYCOBACTERIUM BOVIS MYCOCEROSIC ACID SYNTHASE GENE; M95808	-0,351032985
SRPQSSSNNSVPGAP	RNA POLYMERASE BETA-SUBUNIT AAA21416	-0,351041787
GQYTVFAPTNAAFSK	CAA98373.1; MAJOR SECRETED IMMUNOGENIC PROTEIN	-0,351054462
LGALQAGRIAVPLSV	MYCOBACTERIUM BOVIS ACYL-COA SYNTHASE GENE; U75685	-0,351074264
RWAARDAELRKIPLT	HYPOTHETICAL PROTEIN RV2623 NP_217139	-0,351089587
PTPIGELDVIALYIA	PPE FAMILY PROTEIN CAE55334	-0,351109001
PVKVTRSALQNAASI	60 kDa chaperonin 2 P0A521	-0,351469193
PPLAGASRTDMEGTF	3-OXOACYL-[ACYL-CARRIER PROTEIN] SYNTHASE 2 KASB CAA94642	-0,351885436
GLPPGMGNIGLGNAG	YP_177963; PPE FAMILY PROTEIN	-0,35218738
GEAQLGNSSGNFLLP	PERIPLASMIC PHOSPHATE-BINDING LIPOPROTEIN PSTS1 YP_177770	-0,35224665
STLNPQVGSAQPIVI	PPE FAMILY PROTEIN CAE55334	-0,352401525
PLGFQVPGSIDAITL	YP_177963; PPE FAMILY PROTEIN	-0,352532915
GQRCAPVFVMAAPGQ	CAA16030.1; PROBABLE MOLYBDOPTERIN-GUANINE DINUCLEOTIDE BIOSYNTHESIS PROTEIN	-0,352924106
MSAQAFHQGESSAAF	CONSERVED HYPOTHETICAL PROTEIN TB9.8 CAD93159	-0,352962522
ADLDERGIVRIGAEV	RNA POLYMERASE BETA-SUBUNIT AAA21416	-0,353002095
QLNPFNVNLKLQFLH	YP_177963; PPE FAMILY PROTEIN	-0,353464976
GRGCAQYAAQNPTGP	CAA98350.1; CELL SURFACE LIPOPROTEIN MPT83	-0,353551944
VKTYLKDHDTEGLDI	PROBABLE ISOCITRATE DEHYDROGENASE CAA16247	-0,353761567
VLANGDEVQIGKFRL	CAB01474.1; CONSERVED HYPOTHETICAL PROTEIN CFP17	-0,353767786
TLPPGLTDEQAITAA	MYCOBACTERIUM BOVIS MYCOCEROSIC ACID SYNTHASE GENE; M95808	-0,353831835
VNSSRMYSGPGPESM	PPE FAMILY PROTEIN CAE55334	-0,353876864

Peptides	Protein	Average Index
EEHQDCVAASDLAYT	MYCOBACTERIUM BOVIS MYCOCEROSIC ACID SYNTHASE GENE; M95808	-0,353898032
SLERMAPLVDRQNAG	MTB81 (publication)	-0,353902591
CRRSKLMTRIAGAGA	MYCOBACTERIUM BOVIS MYCOCEROSIC ACID SYNTHASE GENE; M95808	-0,354215686
LFVAEAVTLAHVVRP	POSSIBLE GLYCOSYL TRANSFERASE CAB05415	-0,354438432
TKDGSHYKITGTATG	LIPOPROTEIN LPQH PRECURSOR P0A5J0	-0,354638444
HANGVTHRDVKPENI	TRANSMEMBRANE SERINE/THREONINE-PROTEIN KINASE D PKND NP_215446	-0,354679048
AELPGVDPDKDVDIM	CAA17245.1; HEAT SHOCK PROTEIN HSPX	-0,354715248
MADKTTQTIYIDADP	CONSERVED HYPOTHETICAL PROTEIN TB16.3CAD97060	-0,355052463
LHASTTIGPVSLPIF	YP_177963; PPE FAMILY PROTEIN	-0,355194376
DNSWSVKVPRRLKEL	ALTERNATE RNA POLYMERASE SIGMA FACTOR SIGF CAB07069	-0,355414728
ITIDSVGQTIAGATI	PERIPLASMIC PHOSPHATE-BINDING LIPOPROTEIN PSTS3 YP_177768	-0,355453563
CAQYAAQNPTGPGSV	CAA98350.1; CELL SURFACE LIPOPROTEIN MPT83	-0,355622722
TFESITSRMAEINGM	CAB10947.1; PROBABLE LIPOPROTEIN LPRJ	-0,355721396
ERQILIDNNALGQFT	PPE FAMILY PROTEIN CAE55489	-0,355808158
WAPDEIREEVDNNCQ	MTB81 (publication)	-0,356288181
SGIANFGAGVSGLLN	YP_177963; PPE FAMILY PROTEIN	-0,356558202
RAGAALDYSALYPAG	MYCOBACTERIUM BOVIS MYCOCEROSIC ACID SYNTHASE GENE; M95808	-0,356621496
GLGLSGGTPPFDLPT	YP_177963; PPE FAMILY PROTEIN	-0,357076033
YSLDVPRITILDAHF	YP_177963; PPE FAMILY PROTEIN	-0,357641739
DLEKIIYFAAYVITS	RNA POLYMERASE BETA'-SUBUNIT AAA21417	-0,357731967
PSDVIVDASMPAMIR	PROBABLE ISOCITRATE DEHYDROGENASE CAA16247	-0,357931838
GLPSNMDQHLNMEAL	POSSIBLE GLYCOSYL TRANSFERASE CAB05418	-0,358440275
VDGVVSDEIVYLTAD	RNA POLYMERASE BETA-SUBUNIT AAA21416	-0,358596787
SALLDPSQGMGPSLI	CAB10044.1; SECRETED ANTIGEN 85-B FBPB (85B)	-0,358711522
LGYRNWLGLNKGDLA	MTB81 (publication)	-0,35874932
FLAEKTVKNYVSRLL	TWO COMPONENT TRANSCRIPTIONAL REGULATORY PROTEIN DEVR NP_217649	-0,358853774
IGKFRLVFLTGPKQG	CAB01474.1; CONSERVED HYPOTHETICAL PROTEIN CFP17	-0,358863349
GFGNSTELPSSGFFN	YP_177963; PPE FAMILY PROTEIN	-0,359044188
RHATAALRAAEDDCP	MYCOBACTERIUM BOVIS MYCOCEROSIC ACID SYNTHASE GENE; M95808	-0,359575751
SPHPLLTHAVEQTGR	MYCOBACTERIUM BOVIS MYCOCEROSIC ACID SYNTHASE GENE; M95808	-0,359715398
LFNVGGFNTGVANVG	YP_177963; PPE FAMILY PROTEIN	-0,360959025
TSVAGDPGPDAGTST	POSSIBLE GLYCOSYL TRANSFERASE CAB05418	-0,361109083
TINAQQAELDSALLA	MCE-FAMILY PROTEIN MCE1AYP_177701	-0,361502473
VSGLGLKEAKDLVDG	CAB07109.1; PROBABLE 50S RIBOSOMAL PROTEIN L7/L12 RPLL	-0,361642332
WSFAQAQHLTMANIV	PERIPLASMIC PHOSPHATE-BINDING LIPOPROTEIN PSTS3 YP_177768	-0,361740648
IDIDGQIDGFSTPPI	YP_177963; PPE FAMILY PROTEIN	-0,361846408
GVETRIEAVPIAGFA	3-OXOACYL-[ACYL-CARRIER PROTEIN] SYNTHASE 2 KASB CAA94642	-0,362018143
PRLFVVTRQAQIVKP	MYCOBACTERIUM BOVIS MYCOCEROSIC ACID SYNTHASE GENE; M95808	-0,36222258
AGCGRIVLTARSQPN	MYCOBACTERIUM BOVIS MYCOCEROSIC ACID SYNTHASE GENE; M95808	-0,362235669
VSARNTAVCAPTTGV	CAE55335.1; PE FAMILY PROTEIN	-0,362618944
AAIANRLLRRTATHL	PUTATIVE CYCLOPROPANE-FATTY-ACYL-PHOSPHOLIPID SYNTHASE UFAA1 NP_854118	-0,362713043
DIMVRDGQLTIKAER	CAA17245.1; HEAT SHOCK PROTEIN HSPX	-0,363007331
VATGEVLLTENVEAG	PROBABLE ISOCITRATE DEHYDROGENASE CAA16247	-0,363148876
EKEKASVPGGGDMGG	60 kDa chaperonin 2 P0A521	-0,363170557
SLGSSGLGGGVAANL	CAE55371.1; PPE FAMILY PROTEIN	-0,363239444
HLMRTQGGRGVLMGG	SECRETED L-ALANINE DEHYDROGENASE ALD CAA15575	-0,363726838
DVGSLNGTYVNREPV	CAB01474.1; CONSERVED HYPOTHETICAL PROTEIN CFP17	-0,363889466
VQFVKAICSPMALAS	HYPOTHETICAL PROTEIN RV2626C NP_217142	-0,364136297
TKIDGVGYPVHKPVT	CONSERVED HYPOTHETICAL PROTEIN CAB08634	-0,364328521
YQPQGLAVDGAGTVY	TRANSMEMBRANE SERINE/THREONINE-PROTEIN KINASE D PKND NP_215446	-0,364504103
LPDELLEAHANAIVS	RNA POLYMERASE BETA-SUBUNIT AAA21416	-0,365080015

Peptides	Protein	Average Index
YIASIATGSIALAIT	PPE FAMILY PROTEIN CAE55334	-0,365328096
SMSEVVPIDVSVDIP	YP_177963; PPE FAMILY PROTEIN	-0,365533737
QSSSNNSVPGAPNRV	RNA POLYMERASE BETA-SUBUNIT AAA21416	-0,366004643
YSIEITGSTLVDINE	YP_177963; PPE FAMILY PROTEIN	-0,366139089
VAAICHYIAMWFVVF	POSSIBLE HEMOLYSIN-LIKE PROTEIN CAA17201	-0,366306408
SASIGTLGFTIPGFT	YP_177963; PPE FAMILY PROTEIN	-0,36631177
NNTGEIKSQTVFMGD	RNA POLYMERASE BETA-SUBUNIT AAA21416	-0,366333734
LPLPNIPLLEYAARF	PPE FAMILY PROTEIN CAE55504	-0,367427651
WLEFDVDKRDTVGVR	RNA POLYMERASE BETA-SUBUNIT AAA21416	-0,367677518
TARTINAQQAELDSA	MCE-FAMILY PROTEIN MCE1AYP_177701	-0,367962356
GTAFLRVLNRDRNYT	MTB81 (publication)	-0,368543139
KVLTEYNNKAALEPV	MTB48 AAK31576	-0,368810809
LGYNNAVGAGTGIVI	CAB09453.1; PROBABLE SERINE PROTEASE PEPA	-0,368863491
CFEGSRPTTYDHPTF	SECRETED L-ALANINE DEHYDROGENASE ALD CAA15575	-0,368971602
AVSEGKPTEKHIQIR	CAA17245.1; HEAT SHOCK PROTEIN HSPX	-0,369063093
VASPQSTVIGGTSDT	MYCOBACTERIUM BOVIS MYCOCEROSIC ACID SYNTHASE GENE; M95808	-0,369181887
TTSEQHQATTILRRG	TRANSMEMBRANE SERINE/THREONINE-PROTEIN KINASE D PKND NP_215446	-0,369200718
VKKFEETFEVTAAAP	CAB07109.1; PROBABLE 50S RIBOSOMAL PROTEIN L7/L12 RPLL	-0,369274022
ALNGMPAGLLSGMAL	PPE FAMILY PROTEIN CAE55334	-0,369300067
AFELAARRTTDDDMA	MYCOBACTERIUM BOVIS ACYL-COA SYNTHASE GENE; U75685	-0,369527572
DVVCGGVSTANATVY	CAA98373.1; MAJOR SECRETED IMMUNOGENIC PROTEIN	-0,369813738
DSVMPHPQQAPVLVG	HYPOTHETICAL PROTEIN RV2623 NP_217139	-0,36993164
NIAVHYDLSNDLFAA	PUTATIVE CYCLOPROPANE-FATTY-ACYL-PHOSPHOLIPID SYNTHASE UFAA1 NP_854118	-0,369987933
TQVLVPRSAIDSMLA	CAA98382.1; IMMUNOGENIC PROTEIN MPT64	-0,370140033
YRTDLADRVDTLVGA	CAA16030.1; PROBABLE MOLYBDOPTERIN-GUANINE DINUCLEOTIDE BIOSYNTHESIS PROTEIN	-0,370188357
FSVGSGQTYGVDVVG	CAB09453.1; PROBABLE SERINE PROTEASE PEPA	-0,370331089
EEPERHVWQGDVGTS	MYCOBACTERIUM BOVIS MYCOCEROSIC ACID SYNTHASE GENE; M95808	-0,370333562
HRRRREFKARAAEKP	MTB81 (publication)	-0,370460667
DAYLSEGDMAAHKGL	PERIPLASMIC PHOSPHATE-BINDING LIPOPROTEIN PSTS1 YP_177770	-0,370623428
QSDTVAQGYGSLGLM	CAA17111.1; PROBABLE ISOCITRATE DEHYDROGENASE	-0,370796472
TIFPNGISIPNNPLA	YP_177963; PPE FAMILY PROTEIN	-0,370973558
VITSADVRASLERMA	MTB81 (publication)	-0,371089175
PLTLVHAVSPEVATW	HYPOTHETICAL PROTEIN RV2623 NP_217139	-0,371139634
SRMAEINGMSRDMAS	CAB10947.1; PROBABLE LIPOPROTEIN LPRJ	-0,371310398
AAAIVLVAIVAAAGY	TRANSMEMBRANE SERINE/THREONINE-PROTEIN KINASE D PKND NP_215446	-0,371438802
VGALSVPHSWTTAAP	PPE FAMILY PROTEIN CAE55334	-0,373024295
QLLASEPVFAATIAK	MYCOBACTERIUM BOVIS MYCOCEROSIC ACID SYNTHASE GENE; M95808	-0,37303527
MGHSTASAGTVGLIK	MYCOBACTERIUM BOVIS MYCOCEROSIC ACID SYNTHASE GENE; M95808	-0,373205154
IGISLLSPLALATNG	MCE-FAMILY PROTEIN MCE1AYP_177701	-0,373269108
WYGLNDLAQIKAGDK	MYCOBACTERIUM BOVIS MYCOCEROSIC ACID SYNTHASE GENE; M95808	-0,374049546
AATQAAGAGAVADAQ	PPE FAMILY PROTEIN CAE55334	-0,374195392
NQNTVLDAIQTDAAI	CAA17582.1; PROBABLE SERINE PROTEASE PEPD	-0,374198994
NWLRHGVITSADVRA	MTB81 (publication)	-0,374387602
ITPCELTAAKNAAQQ	MTB48 AAK31576	-0,374471654
KGSGTEVTYELAVDL	CONSERVED HYPOTHETICAL PROTEIN TB16.3CAD97060	-0,374818527
FNSGTGNIGFFNSGT	YP_177963; PPE FAMILY PROTEIN	-0,37525351
VRAMSSLGSSLGSSG	CAE55371.1; PPE FAMILY PROTEIN	-0,375482339
MKIGIMDEERRTTVN	MTB81 (publication)	-0,375620018
VKRADLVIGAVLVPG	SECRETED L-ALANINE DEHYDROGENASE ALD CAA15575	-0,375649944
ARLFIDDDGQEQRAQ	MYCOBACTERIUM BOVIS MYCOCEROSIC ACID SYNTHASE GENE; M95808	-0,376055083
LLSALGITGQFRFGP	YP_177963; PPE FAMILY PROTEIN	-0,37612178
GRSGEPFPYPVTVGY	RNA POLYMERASE BETA-SUBUNIT AAA21416	-0,376644125
LNNAGVNYGDPVDAK	CONSERVED HYPOTHETICAL PROTEIN CAA15739	-0,376717107
LVLSADNMREYLAAG	MTB48 AAK31576	-0,377081398

Peptides	Protein	Average Index
AAGQAAQAAGQARLA	PPE FAMILY PROTEIN CAE55504	-0,377163617
SDGSGVTPGTGMPAA	MTB48 AAK31576	-0,377317675
SGSMSLSFSDPRFDD	RNA POLYMERASE BETA-SUBUNIT AAA21416	-0,377458332
DFKAAGAQLVGTADQ	SECRETED L-ALANINE DEHYDROGENASE ALD CAA15575	-0,377521305
SSHGLSVADLVLVAP	MYCOBACTERIUM BOVIS ACYL-COA SYNTHASE GENE; U75685	-0,377774476
VGSWIGSSAGLMVAA	CAE55371.1; PPE FAMILY PROTEIN	-0,377827754
TEADIARHLPEHAIV	HYPOTHETICAL PROTEIN RV2626C NP_217142	-0,378244231
NVTGGGATTGQASAK	PERIPLASMIC PHOSPHATE-BINDING LIPOPROTEIN PSTS3 YP_177768	-0,378341873
AGVNELVRVYVAQKR	RNA POLYMERASE BETA-SUBUNIT AAA21416	-0,378662298
SSGLGGGVAANLGRA	CAE55371.1; PPE FAMILY PROTEIN	-0,378734019
AFNTPALNIHQIPLG	YP_177963; PPE FAMILY PROTEIN	-0,378916412
VPAARPDVAVLDVRL	TWO COMPONENT TRANSCRIPTIONAL REGULATORY PROTEIN DEVR NP_217649	-0,379812346
YYVDANASIQEMLNV	HYPOTHETICAL PROTEIN RV2626C NP_217142	-0,379847458
DYSALYPAGRLVDAP	MYCOBACTERIUM BOVIS MYCOCEROSIC ACID SYNTHASE GENE; M95808	-0,38038202
NFAGIEAAASAIQGN	CAE55648	-0,38055098
RWEQRDVMAREVAVD	MYCOBACTERIUM BOVIS MYCOCEROSIC ACID SYNTHASE GENE; M95808	-0,380597166
FVASDRVPPGPGQIE	MYCOBACTERIUM BOVIS MYCOCEROSIC ACID SYNTHASE GENE; M95808	-0,380647286
RVFADVSPHPLLTHA	MYCOBACTERIUM BOVIS MYCOCEROSIC ACID SYNTHASE GENE; M95808	-0,381164884
EDFSGSMSLSFSDPR	RNA POLYMERASE BETA-SUBUNIT AAA21416	-0,38130597
DGAYWVDNLRNTVQF	MYCOBACTERIUM BOVIS MYCOCEROSIC ACID SYNTHASE GENE; M95808	-0,381349609
PAFSPPAQALGGGVG	YP_177963; PPE FAMILY PROTEIN	-0,381436041
IVLVAIVAAAGYLVL	TRANSMEMBRANE SERINE/THREONINE-PROTEIN KINASE D PKND NP_215446	-0,381993108
DEERRTTVNLKACIK	MTB81 (publication)	-0,382081899
QTFTSIAISMYCPSV	CONSERVED HYPOTHETICAL PROTEIN CAA15739	-0,382136841
ARAGLVMDPGSKVTY	MCE-FAMILY PROTEIN MCE1AYP_177701	-0,382195289
AAAGAAPAGAAVEAA	CAB07109.1; PROBABLE 50S RIBOSOMAL PROTEIN L7/L12 RPLL	-0,382230581
VVAPGGTFESITSRM	CAB10947.1; PROBABLE LIPOPROTEIN LPRJ	-0,382238284
TRINQSFPVTVNWST	YP_177963; PPE FAMILY PROTEIN	-0,382510616
AILSLRHGVVPPLLH	MYCOBACTERIUM BOVIS MYCOCEROSIC ACID SYNTHASE GENE; M95808	-0,38252194
FGGRFVEIGKADVYG	MYCOBACTERIUM BOVIS MYCOCEROSIC ACID SYNTHASE GENE; M95808	-0,383573024
QAHAALAGAGSGVLN	YP_177963; PPE FAMILY PROTEIN	-0,383617308
DEDELPAGVNELVRV	RNA POLYMERASE BETA-SUBUNIT AAA21416	-0,383726058
LADLIQPDLRVLVEL	PPE FAMILY PROTEIN CAE55504	-0,383757447
GAAHQGQGGAKSKGS	MTB48 AAK31576	-0,383859556
ACPGQTLNYTANGSG	PERIPLASMIC PHOSPHATE-BINDING LIPOPROTEIN PSTS3 YP_177768	-0,38414471
AAATPYVGWLAATAA	PPE FAMILY PROTEIN CAE55334	-0,384199268
YDITALLQAHPHAVN	MYCOBACTERIUM BOVIS MYCOCEROSIC ACID SYNTHASE GENE; M95808	-0,384359664
GQMGARAGGGLSGVL	CAE55371.1; PPE FAMILY PROTEIN	-0,384814855
CSKYPDATTGTAVRA	PERIPLASMIC PHOSPHATE-BINDING LIPOPROTEIN PSTS2 YP_177769	-0,384819464
PGVDPDKDVDIMVRD	CAA17245.1; HEAT SHOCK PROTEIN HSPX	-0,3848777
WAQELAAQTDDQQLA	PROBABLE ISOCITRATE DEHYDROGENASE CAA16247	-0,384957443
RPAVYAPKSALGHSV	3-OXOACYL-[ACYL-CARRIER PROTEIN] SYNTHASE 2 KASB CAA94642	-0,385398727
IVGLERLYAENPSAR	MTB48 AAK31576	-0,385451324
TAVVLLCCSGVATAA	CAA98382.1; IMMUNOGENIC PROTEIN MPT64	-0,386321556
GMLAIGAVTIAVVSA	CAA17582.1; PROBABLE SERINE PROTEASE PEPD	-0,386420454
MAEMKTDAATLAQEA	CAA17966.1; 10 KDA CULTURE FILTRATE ANTIGEN ESXB	-0,386618415
VGAGERKMSALVDAS	CAA16030.1; PROBABLE MOLYBDOPTERIN-GUANINE DINUCLEOTIDE BIOSYNTHESIS PROTEIN	-0,386856538
QNQALLNARDELQAQ	MTB81 (publication)	-0,386918498
GIGGAAASLVGFNRA	CAA17582.1; PROBABLE SERINE PROTEASE PEPD	-0,387015973
DSIAFLAAQELILSG	MTB81 (publication)	-0,387021902

Peptides	Protein	Average Index
EPRRCWPRSRCPRRS	MYCOBACTERIUM BOVIS MYCOCEROSIC ACID SYNTHASE GENE; M95808	-0,387130363
DPDKDVDIMVRDGQL	CAA17245.1; HEAT SHOCK PROTEIN HSPX	-0,387628053
IAGATISGVGNDLVL	PERIPLASMIC PHOSPHATE-BINDING LIPOPROTEIN PSTS3 YP_177768	-0,387631856
ADGTPVDIILNTHGV	RNA POLYMERASE BETA-SUBUNIT AAA21416	-0,38809775
QALNSGTDLPPTPIS	PERIPLASMIC PHOSPHATE-BINDING LIPOPROTEIN PSTS2 YP_177769	-0,388284407
LTEEQRVPDNLAELG	PROBABLE ISOCITRATE DEHYDROGENASE CAA16247	-0,388563908
AAEALTGLGDKFGES	MCE-FAMILY PROTEIN MCE1AYP_177701	-0,388608871
SGTGNFGLFNSGSFN	YP_177963; PPE FAMILY PROTEIN	-0,388719251
QSGLSIVMPVGGQSS	CAB10044.1; SECRETED ANTIGEN 85-B FBPB (85B)	-0,388783081
TGTATGVDMANPMSP	LIPOPROTEIN LPQH PRECURSOR P0A5J0	-0,389020101
PFTPPVQGTGPAGPA	PPE FAMILY PROTEIN CAE55334	-0,389143105
LDDVAGFDAEFFGIS	MYCOBACTERIUM BOVIS MYCOCEROSIC ACID SYNTHASE GENE; M95808	-0,389304348
FAAPVALAAYPITGK	CAB06500.1; IMMUNOGENIC PROTEIN MPT63	-0,389324846
VELKERKHRIEDAVR	60 kDa chaperonin 2 P0A521	-0,389416821
QMRIVMSTNNDDPAG	3-OXOACYL-[ACYL-CARRIER PROTEIN] SYNTHASE 2 KASB CAA94642	-0,389864422
FNNRVVTLAAGSNNQ	TRANSMEMBRANE SERINE/THREONINE-PROTEIN KINASE D PKND NP_215446	-0,389893015
SKIESLPASQRDEII	PROBABLE ISOCITRATE DEHYDROGENASE CAA16247	-0,390033974
LGNHVSGVSIDGLLA	YP_177963; PPE FAMILY PROTEIN	-0,390057084
MNCRAVADHLTRLVD	CONSERVED HYPOTHETICAL PROTEIN CAB08634	-0,390124381
TCGDFELVLVDGGST	POSSIBLE GLYCOSYL TRANSFERASE CAB05419	-0,390164586
RPGSYPIVLATYEIV	PERIPLASMIC PHOSPHATE-BINDING LIPOPROTEIN PSTS3 YP_177768	-0,39079239
MAMRALAMSNLLGQN	YP_177963; PPE FAMILY PROTEIN	-0,391180819
NIISGIPTLSTTGPV	YP_177963; PPE FAMILY PROTEIN	-0,391252519
LVLNALAELPVTVIA	POSSIBLE GLYCOSYL TRANSFERASE CAB05415	-0,391459824
EAHANAIVSTPVFDG	RNA POLYMERASE BETA-SUBUNIT AAA21416	-0,391673872
TPSPGTPEGPWLRTG	MYCOBACTERIUM BOVIS ACYL-COA SYNTHASE GENE; U75685	-0,391819545
PAIHIDFDGAADAGF	YP_177963; PPE FAMILY PROTEIN	-0,392178151
AALVGALSVPHSWTT	PPE FAMILY PROTEIN CAE55334	-0,392348579
ASTGQRSRGPSKFRM	MYCOBACTERIUM BOVIS MYCOCEROSIC ACID SYNTHASE GENE; M95808	-0,392640803
QNAASIAGLFLTTEA	60 kDa chaperonin 2 P0A521	-0,392856747
LAQPTQGTTPSSKLG	CAE55371.1; PPE FAMILY PROTEIN	-0,392927474
AALPAFSPPAQALGG	YP_177963; PPE FAMILY PROTEIN	-0,392934498
ADVLTKNEDVIVREL	PROBABLE ISOCITRATE DEHYDROGENASE CAA16247	-0,393384193
RKTGELDNRGSQFYL	PROBABLE ISOCITRATE DEHYDROGENASE CAA16247	-0,393608028
AAFQAAHARFVAAAA	CONSERVED HYPOTHETICAL PROTEIN TB9.8 CAD93159	-0,393828697
MKIRLHTLLAVLTAA	PERIPLASMIC PHOSPHATE-BINDING LIPOPROTEIN PSTS1 YP_177770	-0,394034246
AAGFGNTTADVFDRG	MCE-FAMILY PROTEIN MCE1AYP_177701	-0,394168358
YHSAASAVATQLAPI	PPE FAMILY PROTEIN CAE55504	-0,394507413
DRPIIYATLGSSGGK	POSSIBLE GLYCOSYL TRANSFERASE CAB05418	-0,3949459
ARLGAIKREVTSALS	MYCOBACTERIUM BOVIS ACYL-COA SYNTHASE GENE; U75685	-0,394960097
QSGGNNSPAVYLLDG	CAB10044.1; SECRETED ANTIGEN 85-B FBPB (85B)	-0,395416675
TTVARDADMLSELGE	CONSERVED HYPOTHETICAL PROTEIN CAB08634	-0,395796038
AWAGPVVMQLMEAAK	PPE FAMILY PROTEIN CAE55489	-0,39596761
VLRQGNSDRRAPKAV	PROBABLE ISOCITRATE DEHYDROGENASE CAA16247	-0,396050862
AVQTAAQNGVRAMSS	CAE55371.1; PPE FAMILY PROTEIN	-0,396080939
DRGGPYLQRGVADLV	MCE-FAMILY PROTEIN MCE1AYP_177701	-0,396177421
FPANSLLNLDVFVGT	YP_177963; PPE FAMILY PROTEIN	-0,39620904
LVAVSWAVGSAKAGL	POSSIBLE HEMOLYSIN-LIKE PROTEIN CAA17201	-0,396488082
IKDLLIVYGRNHSPD	MYCOBACTERIUM BOVIS ACYL-COA SYNTHASE GENE; U75685	-0,396706422
KVNTLLDVAQANLGE	CONSERVED HYPOTHETICAL PROTEIN TB9.8 CAD93159	-0,396739452
LVEGLREVADGDALY	MYCOBACTERIUM BOVIS MYCOCEROSIC ACID SYNTHASE GENE; M95808	-0,39685827
AISMYCPSVMADVAS	CONSERVED HYPOTHETICAL PROTEIN CAA15739	-0,397228384
DHSRDQARRNIAVHY	PUTATIVE CYCLOPROPANE-FATTY-ACYL-PHOSPHOLIPID SYNTHASE UFAA1 NP_854118	-0,397553938
PGIGNIGLGNAGSNN	YP_177963; PPE FAMILY PROTEIN	-0,397626779

Peptides	Protein	Average Index
RTATHLPLRLVYSDG	PUTATIVE CYCLOPROPANE-FATTY-ACYL-PHOSPHOLIPID SYNTHASE UFAA1 NP_854118	-0,397890359
RRSPWGEMFASTGQR	MYCOBACTERIUM BOVIS MYCOCEROSIC ACID SYNTHASE GENE; M95808	-0,398326295
INIPPTPGIGNTTTV	YP_177963; PPE FAMILY PROTEIN	-0,398379639
TSAGGDPVAITIDSV	PERIPLASMIC PHOSPHATE-BINDING LIPOPROTEIN PSTS3 YP_177768	-0,398475338
RQRPRAGMLAIGAVT	CAA17582.1; PROBABLE SERINE PROTEASE PEPD	-0,399130949
EFEGELPRLFVVTRQ	MYCOBACTERIUM BOVIS MYCOCEROSIC ACID SYNTHASE GENE; M95808	-0,3994902
GSLALTLPTVTIPTI	YP_177963; PPE FAMILY PROTEIN	-0,399552718
QGEPVDIGGYYAPDS	PROBABLE ISOCITRATE DEHYDROGENASE CAA16247	-0,399938504
AAHDALTTSEQHQAT	TRANSMEMBRANE SERINE/THREONINE-PROTEIN KINASE D PKND NP_215446	-0,400021491
LTNNHVIAGATDINA	CAB09453.1; PROBABLE SERINE PROTEASE PEPA	-0,400179594
TYNIKGVSTLNLDGP	PERIPLASMIC PHOSPHATE-BINDING LIPOPROTEIN PSTS2 YP_177769	-0,400434598
ELTRRGHEVLIQAGA	SECRETED L-ALANINE DEHYDROGENASE ALD CAA15575	-0,400541565
ASQRGLGEAQLGNSS	PERIPLASMIC PHOSPHATE-BINDING LIPOPROTEIN PSTS1 YP_177770	-0,400590592
SEVDYMDVSPRQMVS	RNA POLYMERASE BETA-SUBUNIT AAA21416	-0,400885534
HIDFDGAADAGFTAP	YP_177963; PPE FAMILY PROTEIN	-0,401103314
FVVTRQAQIVKPHDS	MYCOBACTERIUM BOVIS MYCOCEROSIC ACID SYNTHASE GENE; M95808	-0,401460104
MPNGAAAAVGLERHA	3-OXOACYL-[ACYL-CARRIER PROTEIN] SYNTHASE 2 KASB CAA94642	-0,401519604
PKTQLTMLSARAGLV	MCE-FAMILY PROTEIN MCE1AYP_177701	-0,401639963
YAAQNPTGPGSVAGM	CAA98350.1; CELL SURFACE LIPOPROTEIN MPT83	-0,401680097
LASTLTDALKSHGPQ	MYCOBACTERIUM BOVIS MYCOCEROSIC ACID SYNTHASE GENE; M95808	-0,401824233
LARDSIYYVDANASI	HYPOTHETICAL PROTEIN RV2626C NP_217142	-0,401887493
ELHRAGAALDYSALY	MYCOBACTERIUM BOVIS MYCOCEROSIC ACID SYNTHASE GENE; M95808	-0,402080634
SVPGAPNRVSFAKLR	RNA POLYMERASE BETA-SUBUNIT AAA21416	-0,402569364
VVSQLVRSPGVYFDE	RNA POLYMERASE BETA-SUBUNIT AAA21416	-0,402761096
DQIDKGDVVVLLESM	CAB08316.1; BIOTINYLATED PROTEIN TB7.3	-0,40282194
DSDYDREKLQERLAK	60 kDa chaperonin 2 P0A521	-0,402906395
EPDDFTITTSGVDAE	MTB81 (publication)	-0,403233649
GSDVPLNPSTGQPDR	PERIPLASMIC PHOSPHATE-BINDING LIPOPROTEIN PSTS2 YP_177769	-0,40328269
PFDGLNYPEGLAVDT	TRANSMEMBRANE SERINE/THREONINE-PROTEIN KINASE D PKND NP_215446	-0,403402731
TTVPSSGFFNTGAGG	YP_177963; PPE FAMILY PROTEIN	-0,403592079
TTPKNPTKRRITPKD	MCE-FAMILY PROTEIN MCE1AYP_177701	-0,403597922
LRHGQILFTFLHLAA	SECRETED L-ALANINE DEHYDROGENASE ALD CAA15575	-0,404068367
VAAFEAALAATVDPA	YP_177963; PPE FAMILY PROTEIN	-0,404169637
NRIAPLDGVGALLRY	CONSERVED HYPOTHETICAL PROTEIN CAB08634	-0,404188112
IKVSGPVVELDGDEM	CAA17111.1; PROBABLE ISOCITRATE DEHYDROGENASE	-0,404384206
TLGQPLDWFCLFSSG	MYCOBACTERIUM BOVIS MYCOCEROSIC ACID SYNTHASE GENE; M95808	-0,404589033
YHSGASAAAAALPAF	YP_177963; PPE FAMILY PROTEIN	-0,40466635
AIAVGAALVRDDNRI	CONSERVED HYPOTHETICAL PROTEIN CAB08634	-0,404781523
RVIRPSYGLAEATVY	MYCOBACTERIUM BOVIS ACYL-COA SYNTHASE GENE; U75685	-0,404785503
VFIAGSYTPFALLAL	POSSIBLE HEMOLYSIN-LIKE PROTEIN CAA17201	-0,404947405
VVLKVYQNAGGTHPT	CAA98382.1; IMMUNOGENIC PROTEIN MPT64	-0,405072488
LAIPGTNWIGQAAEA	CAB06237.1; CONSERVED HYPOTHETICAL PROTEIN	-0,405080621
GNSGVASTGLVNAGG	YP_177963; PPE FAMILY PROTEIN	-0,405351172
TETLTMPSEDAQVAV	MYCOBACTERIUM BOVIS MYCOCEROSIC ACID SYNTHASE GENE; M95808	-0,405394133
RLAGWQERYPNVAIT	HYPOTHETICAL PROTEIN RV2623 NP_217139	-0,405597171
EKPAPSDRAGDDAAR	MTB81 (publication)	-0,405601605
GSAPAASLGISTGDV	CAB09453.1; PROBABLE SERINE PROTEASE PEPA	-0,405840241
QIAATAAISAGDQSI	60 kDa chaperonin 2 P0A521	-0,40596474
YSGPGPESMLAAAAA	PPE FAMILY PROTEIN CAE55334	-0,406054107
NNVPQALQQLAQPTQ	CAE55371.1; PPE FAMILY PROTEIN	-0,406235147

Peptides	Protein	Average Index
DADLLLKVKEPIAAE	SECRETED L-ALANINE DEHYDROGENASE ALD CAA15575	-0,406266494
GPILINGVNIPATPG	YP_177963; PPE FAMILY PROTEIN	-0,406555248
ATVYMIDTVLMPPAQ	CAA98350.1; CELL SURFACE LIPOPROTEIN MPT83	-0,406620127
GTAVRAFMQAAIGPG	PERIPLASMIC PHOSPHATE-BINDING LIPOPROTEIN PSTS2 YP_177769	-0,406828371
DEFKSRLSTAVNAIA	PERIPLASMIC PHOSPHATE-BINDING LIPOPROTEIN PSTS3 YP_177768	-0,406877998
TRFVNVFEQACPGQT	PERIPLASMIC PHOSPHATE-BINDING LIPOPROTEIN PSTS3 YP_177768	-0,407076451
AGMAQDPVATAASNN	CAA98350.1; CELL SURFACE LIPOPROTEIN MPT83	-0,407548144
SSQLLANWLRHGVIT	MTB81 (publication)	-0,407565151
TLAATVSPAMVAANR	PPE FAMILY PROTEIN CAE55504	-0,407946607
TVLLKALGWTSEQIV	RNA POLYMERASE BETA-SUBUNIT AAA21416	-0,408196943
KLESVVIATVESGKM	CAA17111.1; PROBABLE ISOCITRATE DEHYDROGENASE	-0,408350786
ASITSDGFHMVAPDP	3-OXOACYL-[ACYL-CARRIER PROTEIN] SYNTHASE 2 KASB CAA94642	-0,408378694
PVAGQVWEATATVNA	CAB06500.1; IMMUNOGENIC PROTEIN MPT63	-0,40858965
MLEMRTHVETETGIR	MYCOBACTERIUM BOVIS MYCOCEROSIC ACID SYNTHASE GENE; M95808	-0,408606458
LMDAWAGPVVMQLME	PPE FAMILY PROTEIN CAE55489	-0,408893635
DGTRTWRTGRQATTL	CAB10947.1; PROBABLE LIPOPROTEIN LPRJ	-0,409245424
HPMLRTTLIDVDEHT	MYCOBACTERIUM BOVIS MYCOCEROSIC ACID SYNTHASE GENE; M95808	-0,409753727
IPVLAEAAGTVSKVA	CAB08316.1; BIOTINYLATED PROTEIN TB7.3	-0,409795401
NPELTTLTAALSGQL	CAA98373.1; MAJOR SECRETED IMMUNOGENIC PROTEIN	-0,410410214
TGEQVLVNEHLIGPP	CONSERVED HYPOTHETICAL PROTEIN CAB08634	-0,411145848
TIGQAEQAAMSAQAF	CONSERVED HYPOTHETICAL PROTEIN TB9.8 CAD93159	-0,411472133
VYVTDTDNNRVVKLE	TRANSMEMBRANE SERINE/THREONINE-PROTEIN KINASE D PKND NP_215446	-0,411475445
AEAERAPIDAGTAAS	CAB06237.1; CONSERVED HYPOTHETICAL PROTEIN	-0,412014624
FGMSGTNVHAIVEEA	MYCOBACTERIUM BOVIS MYCOCEROSIC ACID SYNTHASE GENE; M95808	-0,412770542
AQQTVTGIDKVQPAV	MYCOBACTERIUM BOVIS MYCOCEROSIC ACID SYNTHASE GENE; M95808	-0,41286932
FLHLAASRACTDALL	SECRETED L-ALANINE DEHYDROGENASE ALD CAA15575	-0,413051759
MGSVELPAKQVNSEL	MYCOBACTERIUM BOVIS MYCOCEROSIC ACID SYNTHASE GENE; M95808	-0,413424063
VDAHGAMIRAQAASL	PUTATIVE ESAT-6 LIKE PROTEIN ESXN YP_177838	-0,4134322
VVAGGAAANAGVPKG	CAA17582.1; PROBABLE SERINE PROTEASE PEPD	-0,413486051
MYAGPGSASLVAAAQ	CAE55371.1; PPE FAMILY PROTEIN	-0,413588515
SSSAGADPTALNGMP	PPE FAMILY PROTEIN CAE55334	-0,413635746
DSIARQTCGDFELVL	POSSIBLE GLYCOSYL TRANSFERASE CAB05419	-0,413911471
HVLEIGTGWGELCIR	PUTATIVE CYCLOPROPANE-FATTY-ACYL-PHOSPHOLIPID SYNTHASE UFAA1 NP_854118	-0,414212258
NRDGDVLVDADGKAM	RNA POLYMERASE BETA-SUBUNIT AAA21416	-0,414585351
GELPRLFVVTRQAQI	MYCOBACTERIUM BOVIS MYCOCEROSIC ACID SYNTHASE GENE; M95808	-0,414725045
HQVDVAAVQQGLAGK	MTB81 (publication)	-0,415413566
LSKQDPEGWGKSPGF	PERIPLASMIC PHOSPHATE-BINDING LIPOPROTEIN PSTS1 YP_177770	-0,415549518
TLIDVDEHTDVERVA	MYCOBACTERIUM BOVIS MYCOCEROSIC ACID SYNTHASE GENE; M95808	-0,415576062
GTRPGLIPGVIPTMT	CAA17582.1; PROBABLE SERINE PROTEASE PEPD	-0,415982781
LSTDELLDAFKEMTL	CAB07109.1; PROBABLE 50S RIBOSOMAL PROTEIN L7/L12 RPLL	-0,416184903
QDAGDKDGRTLREIR	CAA17343.1; HEAT SHOCK PROTEIN HSP	-0,416807267
VDMDVQVTMPVPEPM	CONSERVED HYPOTHETICAL PROTEIN TB18.5CAD93033	-0,416822332
KLRGAAEKQDPLSGL	TWO COMPONENT TRANSCRIPTIONAL REGULATORY PROTEIN DEVR NP_217649	-0,417089478
GGCAVLLEPHACVAA	MYCOBACTERIUM BOVIS MYCOCEROSIC ACID SYNTHASE GENE; M95808	-0,417282148
DGYRVFADVSPHPLL	MYCOBACTERIUM BOVIS MYCOCEROSIC ACID SYNTHASE GENE; M95808	-0,418263581
TGQANYGFAVPDIPI	YP_177963; PPE FAMILY PROTEIN	-0,418282976
LTGDNQTGFGGLNSG	YP_177963; PPE FAMILY PROTEIN	-0,418296207
PQALQQLAQPTQGTT	CAE55371.1; PPE FAMILY PROTEIN	-0,418541005

Peptides	Protein	Average Index
NWEGDAATACEASLD	MTB48 AAK31576	-0,418640305
QGYGSLGLMTSVLMT	CAA17111.1; PROBABLE ISOCITRATE DEHYDROGENASE	-0,418647747
GIDKVQPAVFAVQVA	MYCOBACTERIUM BOVIS MYCOCEROSIC ACID SYNTHASE GENE; M95808	-0,4188852
GSKPPSGSPETGAGA	PERIPLASMIC PHOSPHATE-BINDING LIPOPROTEIN PSTS1 YP_177770	-0,418980292
RSTLEKDNTVGTDEA	RNA POLYMERASE BETA-SUBUNIT AAA21416	-0,419148737
PSQTGHSPAVPNALQ	TRANSMEMBRANE SERINE/THREONINE-PROTEIN KINASE D PKND NP_215446	-0,419181098
KAVEKVTETLLKGAK	60 kDa chaperonin 2 P0A521	-0,419252794
ELNGGVGPITVLPIQ	YP_177963; PPE FAMILY PROTEIN	-0,419270695
GNVDTGALMSGNFSN	YP_177963; PPE FAMILY PROTEIN	-0,41935669
ISASVPQLVAAIKEL	PROBABLE ISOCITRATE DEHYDROGENASE CAA16247	-0,419603967
GGAATGIAAVLTDGN	LIPOPROTEIN LPQH PRECURSOR P0A5J0	-0,419767758
IGIGITGNGQIGFGK	PPE FAMILY PROTEIN CAE55504	-0,420129536
LKLEGDEATGANIVK	60 kDa chaperonin 2 P0A521	-0,420308257
VEERPIVGAPVDALV	CAB08889.1; IRON-REGULATED CONSERVED HYPOTHETICAL PROTEIN	-0,420373328
NLNPTTLHFPGFTIP	YP_177963; PPE FAMILY PROTEIN	-0,420584707
ARVLYDFVNNEALPG	MTB81 (publication)	-0,4207375
LRAAEDDCPPPGYDI	MYCOBACTERIUM BOVIS MYCOCEROSIC ACID SYNTHASE GENE; M95808	-0,420833925
AAVGLERHAKAGVMT	3-OXOACYL-[ACYL-CARRIER PROTEIN] SYNTHASE 2 KASB CAA94642	-0,42109619
MDYEQDWDGVAITLT	MYCOBACTERIUM BOVIS ACYL-COA SYNTHASE GENE; U75685	-0,421396976
KSLENYIAQTRDKFL	CAA98382.1; IMMUNOGENIC PROTEIN MPT64	-0,421503473
IGVLERPVGDSSDCG	CAA16102.1; ESAT-6 LIKE PROTEIN ESXQ	-0,421716117
AGADPTALNGMPAGL	PPE FAMILY PROTEIN CAE55334	-0,421802866
SNPALVTRYMHVVVA	POSSIBLE GLYCOSYL TRANSFERASE CAB05419	-0,42184787
AGVVMSHQNVRVNFE	MYCOBACTERIUM BOVIS ACYL-COA SYNTHASE GENE; U75685	-0,421893163
ATTLLSALGITGQFR	YP_177963; PPE FAMILY PROTEIN	-0,421927247
VTQFLNNETDFAGSD	PERIPLASMIC PHOSPHATE-BINDING LIPOPROTEIN PSTS2 YP_177769	-0,422055431
AEAMARVPAARPDVA	TWO COMPONENT TRANSCRIPTIONAL REGULATORY PROTEIN DEVR NP_217649	-0,422354135
ETATQARAAAEAFGT	PPE FAMILY PROTEIN CAE55334	-0,422458034
VGPGCAEYAAANPTG	CAA98373.1; MAJOR SECRETED IMMUNOGENIC PROTEIN	-0,422632379
GVTNLHTAIMAQLAA	PPE FAMILY PROTEIN CAE55504	-0,423257204
GVSGLYNTGGLPPGT	YP_177963; PPE FAMILY PROTEIN	-0,423498997
TNFKVDQPGTVTLTF	CAA17111.1; PROBABLE ISOCITRATE DEHYDROGENASE	-0,424383813
GLQRRDPRWEADLDV	MYCOBACTERIUM BOVIS MYCOCEROSIC ACID SYNTHASE GENE; M95808	-0,425166169
LSAAAEALTGLGDKF	MCE-FAMILY PROTEIN MCE1AYP_177701	-0,425227909
ALPGIVGGAPNPYTY	MCE-FAMILY PROTEIN MCE1AYP_177701	-0,425507463
QAAMSAQAFHQGESS	CONSERVED HYPOTHETICAL PROTEIN TB9.8 CAD93159	-0,425644737
IVAIGTYCPAVIAPL	CAB10947.1; PROBABLE LIPOPROTEIN LPRJ	-0,425703183
VDGGSTDETLDIANI	POSSIBLE GLYCOSYL TRANSFERASE CAB05419	-0,425926156
CAMVLLKRLPDALRD	MYCOBACTERIUM BOVIS MYCOCEROSIC ACID SYNTHASE GENE; M95808	-0,426146515
VSWAVGSAKAGLTTL	POSSIBLE HEMOLYSIN-LIKE PROTEIN CAA17201	-0,426510186
AAAAPYAGWLGSVAA	YP_177963; PPE FAMILY PROTEIN	-0,426532959
ERLIAAHLMDPAPQP	TRANSMEMBRANE SERINE/THREONINE-PROTEIN KINASE D PKND NP_215446	-0,426716291
SRQSCGRVVRVTGRA	POSSIBLE HEMOLYSIN CAA16235	-0,426970474
REVRDTSLKVPHGES	RNA POLYMERASE BETA-SUBUNIT AAA21416	-0,427332098
ADGVLTAPQCTHYPL	MYCOBACTERIUM BOVIS MYCOCEROSIC ACID SYNTHASE GENE; M95808	-0,427491471
GDRRKAMLQDMAILT	60 kDa chaperonin 2 P0A521	-0,427714015
TPANQAISMIDGPAP	PERIPLASMIC PHOSPHATE-BINDING LIPOPROTEIN PSTS1 YP_177770	-0,428553732
GNWPSQTGHSPAVPN	TRANSMEMBRANE SERINE/THREONINE-PROTEIN KINASE D PKND NP_215446	-0,4293466
TSQAATFAANRLDHA	PROBABLE CUTINASE PRECURSOR CFP21 NP_216500	-0,42969896
KSQPWILAYEDHNVD	MTB81 (publication)	-0,429939678
VGAALVRDDNRIAPL	CONSERVED HYPOTHETICAL PROTEIN CAB08634	-0,430011713

Peptides	Protein	Average Index
VKTGSDFVSFAVPTI	ALTERNATE RNA POLYMERASE SIGMA FACTOR SIGF CAB07069	-0,430095837
MTVPGGVEVPVETDD	RNA POLYMERASE BETA-SUBUNIT AAA21416	-0,430192234
DGEMALGKNLLVAIM	RNA POLYMERASE BETA-SUBUNIT AAA21416	-0,430274464
DAPLPAWTHARLFID	MYCOBACTERIUM BOVIS MYCOCEROSIC ACID SYNTHASE GENE; M95808	-0,431082006
FTVAPINLNPTTLHF	YP_177963; PPE FAMILY PROTEIN	-0,431522639
EIAVEQAALQSAWQG	CAA17363.1; LOW MOLECULAR WEIGHT PROTEIN ANTIGEN 7 ESXH	-0,432044813
QVPSPSMGRDIKVQF	CAB10044.1; SECRETED ANTIGEN 85-B FBPB (85B)	-0,43206872
AADILKDESYKVTGT	CAB08889.1; IRON-REGULATED CONSERVED HYPOTHETICAL PROTEIN	-0,432171862
SLLGKARKVVVTKDE	60 kDa chaperonin 2 P0A521	-0,432270642
MGDAGGYKAADMWGP	CAB10044.1; SECRETED ANTIGEN 85-B FBPB (85B)	-0,432552722
GQTYTIPLDTPPAPA	YP_177963; PPE FAMILY PROTEIN	-0,432612296
PALDQVIAKGMAKNP	TRANSMEMBRANE SERINE/THREONINE-PROTEIN KINASE D PKND NP_215446	-0,432871595
NSGAGNLGLFNSGTG	YP_177963; PPE FAMILY PROTEIN	-0,432906169
LLTELHRAGAALDYS	MYCOBACTERIUM BOVIS MYCOCEROSIC ACID SYNTHASE GENE; M95808	-0,433086527
VALPDKSTGLANPGQ	MTB81 (publication)	-0,433342656
GAALGIGNIGLGNAG	YP_177963; PPE FAMILY PROTEIN	-0,433357873
LAGALPGIVGGAPNP	MCE-FAMILY PROTEIN MCE1AYP_177701	-0,433381559
IKSDDTVGRVKVYEA	RNA POLYMERASE BETA-SUBUNIT AAA21416	-0,433405402
KVPRRLKELHLRLGT	ALTERNATE RNA POLYMERASE SIGMA FACTOR SIGF CAB07069	-0,434404091
MGTVPNVGLMAQQAE	PROBABLE ISOCITRATE DEHYDROGENASE CAA16247	-0,434783599
VPGAKAPKLVSNSLV	SECRETED L-ALANINE DEHYDROGENASE ALD CAA15575	-0,435386881
NMEAVERAGAGVLLR	POSSIBLE GLYCOSYL TRANSFERASE CAB05415	-0,435433277
TAAPKTYCEELKGTD	CAA98382.1; IMMUNOGENIC PROTEIN MPT64	-0,435996354
ALVSGLGNVGDHLSG	YP_177963; PPE FAMILY PROTEIN	-0,436063024
RVEDVLGLPQNTMKI	MTB81 (publication)	-0,436377351
GVRPGAVVGHSMGES	MYCOBACTERIUM BOVIS MYCOCEROSIC ACID SYNTHASE GENE; M95808	-0,436810721
ESAAKFILDVDPRYI	MCE-FAMILY PROTEIN MCE1AYP_177701	-0,437122828
VLNSLTGAAQRAGLE	MYCOBACTERIUM BOVIS MYCOCEROSIC ACID SYNTHASE GENE; M95808	-0,437280219
TSDGFHMVAPDPNGE	3-OXOACYL-[ACYL-CARRIER PROTEIN] SYNTHASE 2 KASB CAA94642	-0,437794325
PLALLASASIGTLGF	YP_177963; PPE FAMILY PROTEIN	-0,43793773
GDRVVISAPQGLEYV	MYCOBACTERIUM BOVIS ACYL-COA SYNTHASE GENE; U75685	-0,438056584
GNDLVLDTSSFYRPT	PERIPLASMIC PHOSPHATE-BINDING LIPOPROTEIN PSTS2 YP_177769	-0,438061583
DGDKLAGRHGNKGVI	RNA POLYMERASE BETA-SUBUNIT AAA21416	-0,438187967
GNVNTGALITGNFSN	YP_177963; PPE FAMILY PROTEIN	-0,438231233
AGLDPNTATAGELAR	HYPOTHETICAL PROTEIN RV2626C NP_217142	-0,438350778
IATFAAPVALAAYPI	CAB06500.1; IMMUNOGENIC PROTEIN MPT63	-0,438481893
LQAGRIAVPLSVPQG	MYCOBACTERIUM BOVIS ACYL-COA SYNTHASE GENE; U75685	-0,439195146
FIDDDGQEQRAQGAC	MYCOBACTERIUM BOVIS MYCOCEROSIC ACID SYNTHASE GENE; M95808	-0,439498504
SFWAGVDKVVADLTP	MTB81 (publication)	-0,439531655
PVSTTGEAGNQNTVL	CAA17582.1; PROBABLE SERINE PROTEASE PEPD	-0,439570291
PTLSTTGPVHAVFNA	YP_177963; PPE FAMILY PROTEIN	-0,440091071
VPPAGNKRKVWAVVG	TRANSMEMBRANE SERINE/THREONINE-PROTEIN KINASE D PKND NP_215446	-0,440284314
IGDRNLGIGNTGNWN	PPE FAMILY PROTEIN CAE55504	-0,440415879
VAAAGFGHRVEIDLC	PUTATIVE CYCLOPROPANE-FATTY-ACYL-PHOSPHOLIPID SYNTHASE UFAA1 NP_854118	-0,440819576
VIVDASMPAMIRAGG	PROBABLE ISOCITRATE DEHYDROGENASE CAA16247	-0,440983133
VPLNPSTGQPDRSAE	PERIPLASMIC PHOSPHATE-BINDING LIPOPROTEIN PSTS2 YP_177769	-0,441100394
PMVPPTGSPGGGLPA	MTB48 AAK31576	-0,4414346
TQTWIQKYIFPGGLL	PUTATIVE CYCLOPROPANE-FATTY-ACYL-PHOSPHOLIPID SYNTHASE UFAA1 NP_854118	-0,44235695
GDKDGRTLREIRYGS	CAA17343.1; HEAT SHOCK PROTEIN HSP	-0,442386107
DVVVSVVASPQSTVI	MYCOBACTERIUM BOVIS MYCOCEROSIC ACID SYNTHASE GENE; M95808	-0,442652271
VAFLGALQAGRIAVP	MYCOBACTERIUM BOVIS ACYL-COA SYNTHASE GENE; U75685	-0,442925985

Peptides	Protein	Average Index
KYLDGASNGAWGKGA	PERIPLASMIC PHOSPHATE-BINDING LIPOPROTEIN PSTS2 YP_177769	-0,443194668
TPYRKVVDGVVSDEI	RNA POLYMERASE BETA-SUBUNIT AAA21416	-0,443305246
VAPVSPSTASSRTDT	PPE FAMILY PROTEIN CAE55489	-0,443490493
EVIEGLLAGSSYHTL	ALTERNATE RNA POLYMERASE SIGMA FACTOR SIGF CAB07069	-0,443519941
AYTLQELLTIKSDDT	RNA POLYMERASE BETA-SUBUNIT AAA21416	-0,443771691
ADLVIGAVLVPGAKA	SECRETED L-ALANINE DEHYDROGENASE ALD CAA15575	-0,443978653
DLVYGDVIMRSTNFR	POSSIBLE GLYCOSYL TRANSFERASE CAB05419	-0,444172323
NNLGIGLTGDNQTGI	YP_177963; PPE FAMILY PROTEIN	-0,444482284
TTTATVLAQALVREG	60 kDa chaperonin 2 P0A521	-0,444816058
RLLVEQASVILRRTI	MYCOBACTERIUM BOVIS MYCOCEROSIC ACID SYNTHASE GENE; M95808	-0,445484355
QFRFGPITVSNVQLN	YP_177963; PPE FAMILY PROTEIN	-0,446415892
DAAPDLFDFLDSSVT	MCE-FAMILY PROTEIN MCE1AYP_177701	-0,447146203
GAADPRAPSLFIHRP	PUTATIVE CYCLOPROPANE-FATTY-ACYL-PHOSPHOLIPID SYNTHASE UFAA1 NP_854118	-0,4473313
AVGEPVVAMGNSGGQ	CAB09453.1; PROBABLE SERINE PROTEASE PEPA	-0,44751555
GVLNVGSGISGLYNT	YP_177963; PPE FAMILY PROTEIN	-0,447875314
DDRPINSADALVAAV	CAA17582.1; PROBABLE SERINE PROTEASE PEPD	-0,447927254
TSVLMTADGKTVEAE	CAA17111.1; PROBABLE ISOCITRATE DEHYDROGENASE	-0,448150777
GIPTETKNNEFRVAI	SECRETED L-ALANINE DEHYDROGENASE ALD CAA15575	-0,448213252
EFGGLSNTIVDKEFL	POSSIBLE GLYCOSYL TRANSFERASE CAB05419	-0,448274642
ETVGVVEAHGTGTPI	MYCOBACTERIUM BOVIS MYCOCEROSIC ACID SYNTHASE GENE; M95808	-0,448373307
TLVDMSKDAVLMVVG	HYPOTHETICAL PROTEIN RV2623 NP_217139	-0,448435311
DANASIQEMLNVMEE	HYPOTHETICAL PROTEIN RV2626C NP_217142	-0,449203207
ARIFAGAGLGPMLAA	PPE FAMILY PROTEIN CAE55504	-0,449478992
EALERAGAGVLLRTE	POSSIBLE GLYCOSYL TRANSFERASE CAB05418	-0,449856131
GSLGLMTSVLMTADG	CAA17111.1; PROBABLE ISOCITRATE DEHYDROGENASE	-0,449944193
RREQPLPHGLRGLLT	MYCOBACTERIUM BOVIS MYCOCEROSIC ACID SYNTHASE GENE; M95808	-0,450151159
EVLLKIAQGRLFYNT	POSSIBLE GLYCOSYL TRANSFERASE CAB05418	-0,450410866
ALVDASDALRIVMAD	CAA16030.1; PROBABLE MOLYBDOPTERIN-GUANINE DINUCLEOTIDE BIOSYNTHESIS PROTEIN	-0,450777854
TVLPFTGIDFRLSPS	TRANSMEMBRANE SERINE/THREONINE-PROTEIN KINASE D PKND NP_215446	-0,451449775
DKVIAYARKFLDDSV	MTB81 (publication)	-0,45156774
AGDQSIGDLIAEAMD	60 kDa chaperonin 2 P0A521	-0,451646295
PKPATSPAAPVTTAA	CAA98350.1; CELL SURFACE LIPOPROTEIN MPT83	-0,451655723
AVVLPGLVGLAGGAA	CAB10044.1; SECRETED ANTIGEN 85-B FBPB (85B)	-0,451893278
ATVIDLSTSAMPPAV	PROBABLE CUTINASE PRECURSOR CFP21 NP_216500	-0,452476706
KGTFIINGTERVVVS	RNA POLYMERASE BETA-SUBUNIT AAA21416	-0,452619638
GLVDLLGADPELDVV	TWO COMPONENT TRANSCRIPTIONAL REGULATORY PROTEIN DEVR NP_217649	-0,452639041
PAAPMVPPTGSPGGG	MTB48 AAK31576	-0,45265782
PMLSTLTSALSGKLN	CAA98350.1; CELL SURFACE LIPOPROTEIN MPT83	-0,453053769
GAFTIPQIDIPALNP	YP_177963; PPE FAMILY PROTEIN	-0,453216
QNPTGPGSVAGMAQD	CAA98350.1; CELL SURFACE LIPOPROTEIN MPT83	-0,453376988
NTAASDNFQLSQGGQ	CAB09453.1; PROBABLE SERINE PROTEASE PEPA	-0,453391308
QLRGRDGVVIVYGPR	MYCOBACTERIUM BOVIS MYCOCEROSIC ACID SYNTHASE GENE; M95808	-0,453496483
ERGLNALADAVKVTL	60 kDa chaperonin 2 P0A521	-0,453527383
VRWVDQGVGCSKVPD	MTB81 (publication)	-0,453586825
VNMNAQLVGVNSAIA	CAA17582.1; PROBABLE SERINE PROTEASE PEPD	-0,453588068
NIGIGNIGDRNLGIG	PPE FAMILY PROTEIN CAE55504	-0,454116678
QLARTPVIVARESLT	HYPOTHETICAL PROTEIN RV2623 NP_217139	-0,45452003
NEDVIVRELTEVQGE	PROBABLE ISOCITRATE DEHYDROGENASE CAA16247	-0,45528904
RLLDVAGVQQGSHVL	PUTATIVE CYCLOPROPANE-FATTY-ACYL-PHOSPHOLIPID SYNTHASE UFAA1 NP_854118	-0,455441619
RDGETTRHATAALRA	MYCOBACTERIUM BOVIS MYCOCEROSIC ACID SYNTHASE GENE; M95808	-0,455666774
IPAFGGGTAIPISVG	YP_177963; PPE FAMILY PROTEIN	-0,456285775
APKIKVSGPVVELDG	CAA17111.1; PROBABLE ISOCITRATE DEHYDROGENASE	-0,456407856
IVDVATGEVLLTENV	PROBABLE ISOCITRATE DEHYDROGENASE CAA16247	-0,456507307
PHSWTTAAPEIQLAV	PPE FAMILY PROTEIN CAE55334	-0,457348754

Peptides	Protein	Average Index
HGVGVKCATITPDEA	CAA17111.1; PROBABLE ISOCITRATE DEHYDROGENASE	-0,457445039
TKRYAGLTAANTKKV	POSSIBLE GLYCOSYL TRANSFERASE CAB05419	-0,457655521
GVLNSGVDISGMFNV	YP_177963; PPE FAMILY PROTEIN	-0,457740534
GDTNTGGFNPGSTNT	YP_177963; PPE FAMILY PROTEIN	-0,458281376
FFGTSQLSQFMDQNN	RNA POLYMERASE BETA-SUBUNIT AAA21416	-0,45834549
VVRLELPGIDVDKDV	CAA17343.1; HEAT SHOCK PROTEIN HSP	-0,45851791
GQVTSGLTAGVWQGA	YP_177963; PPE FAMILY PROTEIN	-0,458634572
VNSAAGLHAPMQPGR	CAA16030.1; PROBABLE MOLYBDOPTERIN-GUANINE DINUCLEOTIDE BIOSYNTHESIS PROTEIN	-0,458765012
LIALPPDLASGVVQP	PPE FAMILY PROTEIN CAE55504	-0,459330007
RPIVGAPVDALVNLA	CAB08889.1; IRON-REGULATED CONSERVED HYPOTHETICAL PROTEIN	-0,459661412
SVGPITISPITLFPA	YP_177963; PPE FAMILY PROTEIN	-0,460445094
NGGSPTTQQALAAGV	POSSIBLE GLYCOSYL TRANSFERASE CAB05418	-0,460497108
VGWLNTAAGQAAQAA	PPE FAMILY PROTEIN CAE55504	-0,460664187
SGAGGVSGFGNFGAG	YP_177963; PPE FAMILY PROTEIN	-0,46076614
LHSVKVIPSRGAWLE	RNA POLYMERASE BETA-SUBUNIT AAA21416	-0,460860608
GALLRYAATNRLGSH	CONSERVED HYPOTHETICAL PROTEIN CAB08634	-0,460877836
LALLAGVFGGAASCA	CAB10947.1; PROBABLE LIPOPROTEIN LPRJ	-0,461138003
FRRSFRLPAHVTSEA	CAA17343.1; HEAT SHOCK PROTEIN HSP	-0,461333076
SLGFGNLLSFASLPT	CAB06237.1; CONSERVED HYPOTHETICAL PROTEIN	-0,461362752
LSRMPDLRCLILTSY	TWO COMPONENT TRANSCRIPTIONAL REGULATORY PROTEIN DEVR NP_217649	-0,46165991
PGQFAGYTGAAESPT	MTB81 (publication)	-0,461879829
VTIPTITTSPIPLKI	YP_177963; PPE FAMILY PROTEIN	-0,461948253
YKITGTATGVDMANP	LIPOPROTEIN LPQH PRECURSOR P0A5J0	-0,462145469
SFYRPTQPGSYPIVL	PERIPLASMIC PHOSPHATE-BINDING LIPOPROTEIN PSTS2 YP_177769	-0,462310681
IPEPGIPESFKVLLK	RNA POLYMERASE BETA-SUBUNIT AAA21416	-0,462451649
GFAQMRIVMSTNNDD	3-OXOACYL-[ACYL-CARRIER PROTEIN] SYNTHASE 2 KASB CAA94642	-0,46274693
LIGSPRWRESAAERG	RNA POLYMERASE BETA-SUBUNIT AAA21416	-0,462756459
DKDGTAFLRVLNRDR	MTB81 (publication)	-0,463570756
LFSRVEDVLGLPQNT	MTB81 (publication)	-0,463972314
SRQSKTAASPSPSRP	RNA POLYMERASE BETA-SUBUNIT AAA21416	-0,464968485
PTITTSPIPLKIDVS	YP_177963; PPE FAMILY PROTEIN	-0,465484147
LVLTTTAGGLASPYV	MYCOBACTERIUM BOVIS MYCOCEROSIC ACID SYNTHASE GENE; M95808	-0,46580825
CELTAAKNAAQQLVL	MTB48 AAK31576	-0,466618146
AILVAGLSGCSSNKS	LIPOPROTEIN LPQH PRECURSOR P0A5J0	-0,466795301
LFLHSGLIALPPDLA	PPE FAMILY PROTEIN CAE55504	-0,467593046
PNIPLLEYAARFITP	PPE FAMILY PROTEIN CAE55504	-0,467888179
VTYELAVDLAVPMIG	CONSERVED HYPOTHETICAL PROTEIN TB16.3CAD97060	-0,469891081
VGNLRIARVLYDFVN	MTB81 (publication)	-0,469923575
AATALDNDGEGTVQA	MTB48 AAK31576	-0,470090457
AGSYTPFALLALPAH	POSSIBLE HEMOLYSIN-LIKE PROTEIN CAA17201	-0,470662438
AQAGTESAVSGVEGL	CAB01474.1; CONSERVED HYPOTHETICAL PROTEIN CFP17	-0,470887604
LNGHHPGDVISVTWQ	CAB09453.1; PROBABLE SERINE PROTEASE PEPA	-0,470955806
PVDECKDKDMTYAAP	RNA POLYMERASE BETA-SUBUNIT AAA21416	-0,47166183
QGSHVLEIGTGWGEL	PUTATIVE CYCLOPROPANE-FATTY-ACYL-PHOSPHOLIPID SYNTHASE UFAA1 NP_854118	-0,471966246
LLASNVGQNPITIVN	YP_177963; PPE FAMILY PROTEIN	-0,473576052
VAARILAEFPDYLTE	PROBABLE ISOCITRATE DEHYDROGENASE CAA16247	-0,473709184
RAVADHLTRLVDAAD	CONSERVED HYPOTHETICAL PROTEIN CAB08634	-0,474408747
ASNNPELTTLTAALS	CAA98373.1; MAJOR SECRETED IMMUNOGENIC PROTEIN	-0,474418539
DKDMTYAAPLFVTAE	RNA POLYMERASE BETA-SUBUNIT AAA21416	-0,474600207
QAAHARFVAAAAKVN	CONSERVED HYPOTHETICAL PROTEIN TB9.8 CAD93159	-0,474956724
DAKQELIDSLEEAVR	CONSERVED HYPOTHETICAL PROTEIN CAB08634	-0,475109143
ALPPFTPPVQGTGPA	PPE FAMILY PROTEIN CAE55334	-0,475185056
TLTGNPNRPDGGILT	PPE FAMILY PROTEIN CAE55504	-0,475231272
HDIGALPICGDDDRL	HYPOTHETICAL PROTEIN RV2626C NP_217142	-0,475244198
EQRVPDNLAELGRLT	PROBABLE ISOCITRATE DEHYDROGENASE CAA16247	-0,476749649
ILPTIPLNIHQTFSL	YP_177963; PPE FAMILY PROTEIN	-0,477001997
ATADAYATTEAVNVV	CAE55335.1; PE FAMILY PROTEIN	-0,477450595
KVVREIVSGLGLKEA	CAB07109.1; PROBABLE 50S RIBOSOMAL PROTEIN L7/L12 RPLL	-0,47810553

Peptides	Protein	Average Index
AIPSIHLGIDPTFDV	YP_177963; PPE FAMILY PROTEIN	-0,478110597
VNNGLSDLYSKIESL	PROBABLE ISOCITRATE DEHYDROGENASE CAA16247	-0,478315304
IDAITLFPGGLTFPA	YP_177963; PPE FAMILY PROTEIN	-0,478736017
IQRTVASCPNTRIVL	PROBABLE CUTINASE PRECURSOR CFP21 NP_216500	-0,479569044
TLQGASVTVTGQGNS	CAA98373.1; MAJOR SECRETED IMMUNOGENIC PROTEIN	-0,479600214
LGEATVVTGKARTTV	CONSERVED HYPOTHETICAL PROTEIN CAB08634	-0,479947992
IAFNSGLEPGVVAEK	60 kDa chaperonin 2 P0A521	-0,480792719
PFVRWLTDLCVQLSE	PPE FAMILY PROTEIN CAE55489	-0,481350443
LNIPQNAIPLTIDAS	YP_177963; PPE FAMILY PROTEIN	-0,48161709
VLCNGGSPTTQQALA	POSSIBLE GLYCOSYL TRANSFERASE CAB05418	-0,481684849
DLAAAVDKDGTAFLR	MTB81 (publication)	-0,481920542
PQNAIPLTIDASGVL	YP_177963; PPE FAMILY PROTEIN	-0,483459834
RSNHGTCANQCPIVD	RNA POLYMERASE BETA-SUBUNIT AAA21416	-0,484210419
GLSDLYSKIESLPAS	PROBABLE ISOCITRATE DEHYDROGENASE CAA16247	-0,485869731
VAFTCELFSRVEDVL	MTB81 (publication)	-0,485956583
NVHAIVEEAPAEASA	MYCOBACTERIUM BOVIS MYCOCEROSIC ACID SYNTHASE GENE; M95808	-0,48596588
MSNAEHTGKLVLDVP	MYCOBACTERIUM BOVIS MYCOCEROSIC ACID SYNTHASE GENE; M95808	-0,486091583
ATVDPAAVAVNRMAM	YP_177963; PPE FAMILY PROTEIN	-0,486230669
TGKLVLDVPRSGRRS	MYCOBACTERIUM BOVIS MYCOCEROSIC ACID SYNTHASE GENE; M95808	-0,486350773
SGISGLYNTAIVGLG	YP_177963; PPE FAMILY PROTEIN	-0,487488567
AIAGILLKMCWPAAP	POSSIBLE HEMOLYSIN-LIKE PROTEIN CAA17201	-0,487769389
LTNNHVIAAAAKPPL	CAA17582.1; PROBABLE SERINE PROTEASE PEPD	-0,487966593
RLNTEGVAAAVKQVL	POSSIBLE GLYCOSYL TRANSFERASE CAB05418	-0,488156925
VGSFRGRWLDPRHAG	CAA16102.1; ESAT-6 LIKE PROTEIN ESXQ	-0,488564591
RRVPVISEHRLVGIV	HYPOTHETICAL PROTEIN RV2626C NP_217142	-0,48894731
QSLSWTLESSSLLKS	CONSERVED HYPOTHETICAL PROTEIN TB16.3CAD97060	-0,489275909
GTGTPIGDPIEYRSL	MYCOBACTERIUM BOVIS MYCOCEROSIC ACID SYNTHASE GENE; M95808	-0,491310547
EAEDTRKHRVVALKL	TRANSMEMBRANE SERINE/THREONINE-PROTEIN KINASE D PKND NP_215446	-0,491704166
TTSPIPLKIDVSGGL	YP_177963; PPE FAMILY PROTEIN	-0,491837903
ITNVAQQFEAEIGRG	CONSERVED HYPOTHETICAL PROTEIN CAB08634	-0,492307589
QATPTFSSSAGADPT	PPE FAMILY PROTEIN CAE55334	-0,492760294
TVGLIKAILSLRHGV	MYCOBACTERIUM BOVIS MYCOCEROSIC ACID SYNTHASE GENE; M95808	-0,493407964
EADTLARVAAFIGEH	POSSIBLE GLYCOSYL TRANSFERASE CAB05419	-0,493586452
PVPEPMVKMLLNNVL	CONSERVED HYPOTHETICAL PROTEIN TB18.5CAD93033	-0,493660432
VGMNTAASDNFQLSQ	CAB09453.1; PROBABLE SERINE PROTEASE PEPA	-0,495455821
VTATMGQLQQLVAAG	CAB06237.1; CONSERVED HYPOTHETICAL PROTEIN	-0,495819422
ATIIPPIHIPSIPLG	YP_177963; PPE FAMILY PROTEIN	-0,496227786
SLGISTGDVITAVDG	CAB09453.1; PROBABLE SERINE PROTEASE PEPA	-0,496315032
LLSFASLPTVGQVTA	CAB06237.1; CONSERVED HYPOTHETICAL PROTEIN	-0,496539749
SGQTVLPFTGIDFRL	TRANSMEMBRANE SERINE/THREONINE-PROTEIN KINASE D PKND NP_215446	-0,49703719
IDTSNAVDTPDMLAS	MYCOBACTERIUM BOVIS MYCOCEROSIC ACID SYNTHASE GENE; M95808	-0,497752742
ARAVKDVGAGRSLLD	TWO COMPONENT TRANSCRIPTIONAL REGULATORY PROTEIN DEVR NP_217649	-0,497821857
MLSARAGLVMDPGSK	MCE-FAMILY PROTEIN MCE1AYP_177701	-0,498944993
QELIDSLEEAVRDSR	CONSERVED HYPOTHETICAL PROTEIN CAB08634	-0,499628669
GNPLADLIQPDLRVL	PPE FAMILY PROTEIN CAE55504	-0,499985451
SGAEFRQAARRLAEA	POSSIBLE GLYCOSYL TRANSFERASE CAB05418	-0,502098398
VLAIGSPLGLEGTVT	CAA17582.1; PROBABLE SERINE PROTEASE PEPD	-0,502974105
DIARHLPEHAIVQFV	HYPOTHETICAL PROTEIN RV2626C NP_217142	-0,503335871
PVALAAYPITGKLGS	CAB06500.1; IMMUNOGENIC PROTEIN MPT63	-0,504289731
EYGSHDKTFEIPEDG	PROBABLE ISOCITRATE DEHYDROGENASE CAA16247	-0,504478414
TGTAPIYEILHDAKE	CAB08889.1; IRON-REGULATED CONSERVED HYPOTHETICAL PROTEIN	-0,508969903
EHTQDAGDKDGRTLR	CAA17343.1; HEAT SHOCK PROTEIN HSP	-0,510336538
GKSFQGGVGEGARGN	PERIPLASMIC PHOSPHATE-BINDING LIPOPROTEIN PSTS3 YP_177768	-0,512072913
RAQGACTITVHPLLG	MYCOBACTERIUM BOVIS MYCOCEROSIC ACID SYNTHASE GENE;	-0,514164657

Peptides	Protein	Average Index
	M95808	
LIKDMLILPYLDIRL	CAA17111.1; PROBABLE ISOCITRATE DEHYDROGENASE	-0,514926182
SERVATDLGVPFTEP	SECRETED L-ALANINE DEHYDROGENASE ALD CAA15575	-0,51617529
YRAALAAAGVQPETV	MYCOBACTERIUM BOVIS MYCOCEROSIC ACID SYNTHASE GENE; M95808	-0,517236555
FLQSTIGAGQSGLGD	PERIPLASMIC PHOSPHATE-BINDING LIPOPROTEIN PSTS3 YP_177768	-0,517386614
DNRLSLSVSARLAGI	POSSIBLE GLYCOSYL TRANSFERASE CAB05418	-0,518317919
PIAHSTVLVAPVSPS	PPE FAMILY PROTEIN CAE55489	-0,518384738
VTRSALQNAASIAGL	60 kDa chaperonin 2 P0A521	-0,518700243
AITAQQTVTGIDKVQ	MYCOBACTERIUM BOVIS MYCOCEROSIC ACID SYNTHASE GENE; M95808	-0,520766726
LLAQPTPAWTELAAA	PUTATIVE CYCLOPROPANE-FATTY-ACYL-PHOSPHOLIPID SYNTHASE UFAA1 NP_854118	-0,522593704
IPIPAIHIDFDGAAD	YP_177963; PPE FAMILY PROTEIN	-0,523206842
TDLGRQSEEGSGIIL	CAA17582.1; PROBABLE SERINE PROTEASE PEPD	-0,523412359
NATNLRGILGRLIEM	CAB06237.1; CONSERVED HYPOTHETICAL PROTEIN	-0,523603931
NAFLTALTNAGIAYD	CAB10947.1; PROBABLE LIPOPROTEIN LPRJ	-0,524132996
DRKILNEIAPDVVVG	POSSIBLE GLYCOSYL TRANSFERASE CAB05418	-0,524288392
RAIPLLGNPLADLIQ	PPE FAMILY PROTEIN CAE55504	-0,528380844
ARVYGAGTPCALGSA	MYCOBACTERIUM BOVIS MYCOCEROSIC ACID SYNTHASE GENE; M95808	-0,529834144
ATTGSLALTLPTVTI	YP_177963; PPE FAMILY PROTEIN	-0,530040409
HSPQVDPILDDLAAA	MYCOBACTERIUM BOVIS MYCOCEROSIC ACID SYNTHASE GENE; M95808	-0,531080296
DPLSGLTDQERTLLG	TWO COMPONENT TRANSCRIPTIONAL REGULATORY PROTEIN DEVR NP_217649	-0,532169984
ATGFTVQDGQLVVAL	MTB81 (publication)	-0,535840048
GREAAALSGDVAVKA	MTB48 AAK31576	-0,536103708
SAAPNFAFELAARRT	MYCOBACTERIUM BOVIS ACYL-COA SYNTHASE GENE; U75685	-0,536871639
ILSGSERVQAATIKR	MYCOBACTERIUM BOVIS ACYL-COA SYNTHASE GENE; U75685	-0,541198663
YAGEKSMTLDRARNV	PROBABLE ISOCITRATE DEHYDROGENASE CAA16247	-0,541523747
AAKPFVRWLTDLCVQ	PPE FAMILY PROTEIN CAE55489	-0,541766222
VVIDRIPVELHASTT	YP_177963; PPE FAMILY PROTEIN	-0,542841778
DSLLPLPNIPLLEYA	PPE FAMILY PROTEIN CAE55504	-0,543618292
LGDVGEAFVDSLTSQ	PROBABLE CUTINASE PRECURSOR CFP21 NP_216500	-0,543925235
GFTIPGGTLIPQLPL	YP_177963; PPE FAMILY PROTEIN	-0,544148862
QIENREVLRPLLEAL	ALTERNATE RNA POLYMERASE SIGMA FACTOR SIGF CAB07069	-0,546293135
VVGDNRLSLSVSARL	POSSIBLE GLYCOSYL TRANSFERASE CAB05418	-0,546564451
PPTKESAQTLLENLF	RNA POLYMERASE BETA-SUBUNIT AAA21416	-0,547349912
NKGDLAAAVDKDGTA	MTB81 (publication)	-0,548389968
AARALPLTSLTSAAE	CAE55371.1; PPE FAMILY PROTEIN	-0,549355139
PVGGLEEVLYELSPI	RNA POLYMERASE BETA-SUBUNIT AAA21416	-0,551921429
IPIDIPPIDIPASTI	YP_177963; PPE FAMILY PROTEIN	-0,553269072
DQHLNMEALERAGAG	POSSIBLE GLYCOSYL TRANSFERASE CAB05418	-0,558933009
DAYATTEAVNVVATG	CAE55335.1; PE FAMILY PROTEIN	-0,559109979
LLANWLRHGVITSAD	MTB81 (publication)	-0,560507385
VNLKLQFLHDAFPNE	YP_177963; PPE FAMILY PROTEIN	-0,562429303
VTIQPVVLAAATGDL	CAE55335.1; PE FAMILY PROTEIN	-0,562562212
EHFASLADVLTKNED	PROBABLE ISOCITRATE DEHYDROGENASE CAA16247	-0,562596844
RVQRVVGSAPAASLG	CAB09453.1; PROBABLE SERINE PROTEASE PEPA	-0,56861691
TLFQTLTSIAEKVDP	MCE-FAMILY PROTEIN MCE1AYP_177701	-0,571792683
ASLEAEHQAIVRDVL	PUTATIVE ESAT-6 LIKE PROTEIN ESXN YP_177838	-0,57228867
AALEQLVRPGGPVAI	PUTATIVE CYCLOPROPANE-FATTY-ACYL-PHOSPHOLIPID SYNTHASE UFAA1 NP_854118	-0,573846442
KKIGVIKVVREIVSG	CAB07109.1; PROBABLE 50S RIBOSOMAL PROTEIN L7/L12 RPLL	-0,574981242
PRAGMLAIGAVTIAV	CAA17582.1; PROBABLE SERINE PROTEASE PEPD	-0,575350245
LNIHQIPLGFQVPGS	YP_177963; PPE FAMILY PROTEIN	-0,576134807
AALAGMRREQPLPHG	MYCOBACTERIUM BOVIS MYCOCEROSIC ACID SYNTHASE GENE; M95808	-0,5792977
AEINGMSRDMASTFT	CAB10947.1; PROBABLE LIPOPROTEIN LPRJ	-0,58434934
LVNAAVRFDVKTGSD	ALTERNATE RNA POLYMERASE SIGMA FACTOR SIGF CAB07069	-0,59046939
LAGVFGGAASCAAPI	CAB10947.1; PROBABLE LIPOPROTEIN LPRJ	-0,59124871
DVSVLTAARFTAHTK	CAE55335.1; PE FAMILY PROTEIN	-0,592915012
QIDIPALNPNVTGSV	YP_177963; PPE FAMILY PROTEIN	-0,596016156

Peptides	Protein	Average Index
DELKTNSSLLTSILT	CAA98373.1; MAJOR SECRETED IMMUNOGENIC PROTEIN	-0,596611332
PGAVVGHSMGESAAA	MYCOBACTERIUM BOVIS MYCOCEROSIC ACID SYNTHASE GENE; M95808	-0,597983199
QLVVALPDKSTGLAN	MTB81 (publication)	-0,602101234
AIVGLGTPALVSGAG	YP_177963; PPE FAMILY PROTEIN	-0,602916048
AAARGAHIRSVTLSV	PUTATIVE CYCLOPROPANE-FATTY-ACYL-PHOSPHOLIPID SYNTHASE UFAA1 NP_854118	-0,603666942
ASEPVFAATIAKLEP	MYCOBACTERIUM BOVIS MYCOCEROSIC ACID SYNTHASE GENE; M95808	-0,607288782
TEGVAAAVKQVLSGA	POSSIBLE GLYCOSYL TRANSFERASE CAB05418	-0,607830219
RAYPQCPLLLHAVDQ	MYCOBACTERIUM BOVIS MYCOCEROSIC ACID SYNTHASE GENE; M95808	-0,609771995
DGKMFIIGRIKDLLI	MYCOBACTERIUM BOVIS ACYL-COA SYNTHASE GENE; U75685	-0,609962493
ATNKTARALAQYLAD	MYCOBACTERIUM BOVIS MYCOCEROSIC ACID SYNTHASE GENE; M95808	-0,619946612
ADHLTRLVDAADPEV	CONSERVED HYPOTHETICAL PROTEIN CAB08634	-0,620221773
DVRAEIVASVLEVVV	CAB08316.1; BIOTINYLATED PROTEIN TB7.3	-0,621564329
AIPIGLELNGGVGPI	YP_177963; PPE FAMILY PROTEIN	-0,62413237
AGDIPLINIPPTPGI	YP_177963; PPE FAMILY PROTEIN	-0,633342714
MGPSLIGLAMGDAGG	CAB10044.1; SECRETED ANTIGEN 85-B FBPB (85B)	-0,64740066
RAELMILIATNLLGQ	CAE55371.1; PPE FAMILY PROTEIN	-0,651125279
RAKLLGKTLDAAIGK	PROBABLE ISOCITRATE DEHYDROGENASE CAA16247	-0,657436644
VFGGAASCAAPIQAD	CAB10947.1; PROBABLE LIPOPROTEIN LPRJ	-0,658447949
SITSRMAEINGMSRD	CAB10947.1; PROBABLE LIPOPROTEIN LPRJ	-0,675078091
VAQSYLRNFLAAPPP	LOW MOLECULAR WEIGHT T-CELL ANTIGEN TB8.4 NP_215690	-0,699561648
DTADRLVSAATATGL	MYCOBACTERIUM BOVIS MYCOCEROSIC ACID SYNTHASE GENE; M95808	-0,702277187
NVLEHLAENLKQRAE	CONSERVED HYPOTHETICAL PROTEIN TB18.5CAD93033	-0,705313583
SRDMASTFTIVAIGT	CAB10947.1; PROBABLE LIPOPROTEIN LPRJ	-0,709452938
SSILTYHVIAGQASP	CAA98350.1; CELL SURFACE LIPOPROTEIN MPT83	-0,718239167
GFTIPAAPLPLTIDI	YP_177963; PPE FAMILY PROTEIN	-0,728513338
AANDVSVLTAARFTA	CAE55335.1; PE FAMILY PROTEIN	-0,744665219
LPSLPDIIDGLWPPK	CAB06237.1; CONSERVED HYPOTHETICAL PROTEIN	-0,751621634
PVLAINAHLTGNAAM	YP_177963; PPE FAMILY PROTEIN	-0,770758114
IIPDTTILPPIHLGL	YP_177963; PPE FAMILY PROTEIN	-0,77255184
CVLGECLTGAPPYRA	TRANSMEMBRANE SERINE/THREONINE-PROTEIN KINASE D PKND NP_215446	-0,800689217
LIDGALKDLKKRVEG	CONSERVED HYPOTHETICAL PROTEIN TB16.3CAD97060	-0,812672106
PSEEVLLKIAQGRLF	POSSIBLE GLYCOSYL TRANSFERASE CAB05418	-0,813940256
LTDRDIVIKGLAAGL	HYPOTHETICAL PROTEIN RV2626C NP_217142	-0,820897343
TVELIEDLARRAVQT	CAA16030.1; PROBABLE MOLYBDOPTERIN-GUANINE DINUCLEOTIDE BIOSYNTHESIS PROTEIN	-0,826108055
QLVAAIKELQDKGYA	PROBABLE ISOCITRATE DEHYDROGENASE CAA16247	-0,833011504
VPEMFRELVGLPAGS	ALTERNATE RNA POLYMERASE SIGMA FACTOR SIGF CAB07069	-0,847381209
LENADLSLLGKARKV	60 kDa chaperonin 2 P0A521	-0,848108173
GYGVDIVLNSLTGAA	MYCOBACTERIUM BOVIS MYCOCEROSIC ACID SYNTHASE GENE; M95808	-0,848203845
DAAIGKLLDNDKSPS	PROBABLE ISOCITRATE DEHYDROGENASE CAA16247	-0,863757443
SKVSTVKDLLPLLEK	60 kDa chaperonin 2 P0A521	-0,874279169
LQQTIDAIELPAISF	YP_177963; PPE FAMILY PROTEIN	-0,881866384
LLGKTLDAAIGKLLD	PROBABLE ISOCITRATE DEHYDROGENASE CAA16247	-0,899734236
LPELVEGLREVADGD	MYCOBACTERIUM BOVIS MYCOCEROSIC ACID SYNTHASE GENE; M95808	-0,905163319
GPYQLLRLLGRGGMG	TRANSMEMBRANE SERINE/THREONINE-PROTEIN KINASE D PKND NP_215446	-0,910251542
VGLSTIAGRLLGSVP	CAB08889.1; IRON-REGULATED CONSERVED HYPOTHETICAL PROTEIN	-0,912872514
LVSSKVSTVKDLLPL	60 kDa chaperonin 2 P0A521	-0,951999699
AGKPLLIIAEDVEGE	60 kDa chaperonin 2 P0A521	-0,991330499
GILGRLIEMLTTLPK	CAB06237.1; CONSERVED HYPOTHETICAL PROTEIN	-1,135545356
LPPAANDVSVLTAAR	CAE55335.1; PE FAMILY PROTEIN	-1,147225742
YFKGVPSRLGYLLDL	RNA POLYMERASE BETA'-SUBUNIT AAA21417	0,178853331
SGLTDQERTLLGLLS	TWO COMPONENT TRANSCRIPTIONAL REGULATORY PROTEIN DEVR NP_217649	-0,254510782
TSGGRNLLQLVLNAL	POSSIBLE GLYCOSYL TRANSFERASE CAB05415	-0,258911429

Peptides	Protein	Average Index
LGGANIPAEFLENFV	CAB10044.1; SECRETED ANTIGEN 85-B FBPB (85B)	-0,432912877
RQEAVLEDPYILLVS	60 kDa chaperonin 2 P0A521	-0,473624042
MAEDVRAEIVASVLE	CAB08316.1; BIOTINYLATED PROTEIN TB7.3	-1,170759902

Supplementary Table 6c. Peptides recognized only by IgA and not by IgG

Peptides	Protein	Average Index
RMCIVKDAPIRDWVK	PROBABLE ISOCITRATE DEHYDROGENASE CAA16247	1,78007277
IMYNYPAMMAHAGDM	CAA16104.1; SECRETED ESAT-6 LIKE PROTEIN ESXR	1,688376874
GSGFGNFGANMSGWW	YP_177963; PPE FAMILY PROTEIN	1,490983745
LQRPARWMHLMASDF	MYCOBACTERIUM BOVIS ACYL-COA SYNTHASE GENE; U75685	1,294760404
ERPLLFALQCMPLNW	POSSIBLE GLYCOSYL TRANSFERASE CAB05415	1,256942606
YNGWDINTPAFEWYY	CAB10044.1; SECRETED ANTIGEN 85-B FBPB (85B)	1,215241303
EMFASTGQRSRGPSK	MYCOBACTERIUM BOVIS MYCOCEROSIC ACID SYNTHASE GENE; M95808	1,164785023
GGTHPTTTYKAFDWD	CAA98382.1; IMMUNOGENIC PROTEIN MPT64	1,047504081
SPACGKAGCQTYKWE	CAB10044.1; SECRETED ANTIGEN 85-B FBPB (85B)	1,024058672
VIMRSTNFRWGGAFD	POSSIBLE GLYCOSYL TRANSFERASE CAB05419	0,925754789
GFSSMLWGGGSLPTI	PROBABLE CUTINASE PRECURSOR CFP21 NP_216500	0,755000782
HHRMLATRHTQTWIQ	PUTATIVE CYCLOPROPANE-FATTY-ACYL-PHOSPHOLIPID SYNTHASE UFAA1 NP_854118	0,722282932
TTYKAFDWDQAYRKP	CAA98382.1; IMMUNOGENIC PROTEIN MPT64	0,713282246
LKKMWLSPNGTIRNI	CAA17111.1; PROBABLE ISOCITRATE DEHYDROGENASE	0,632979924
HMVGILGQRCAPVFV	CAA16030.1; PROBABLE MOLYBDOPTERIN-GUANINE DINUCLEOTIDE BIOSYNTHESIS PROTEIN	0,422205503
SFSYGLNAKWPVYLS	CAA17111.1; PROBABLE ISOCITRATE DEHYDROGENASE	0,407767066
LHDAKERAHNAGAKN	CAB08889.1; IRON-REGULATED CONSERVED HYPOTHETICAL PROTEIN	0,396315331
LTEGMRFDKGYISGY	60 kDa chaperonin 2 P0A521	0,355902737
ELCRDLLSRMPDLRC	TWO COMPONENT TRANSCRIPTIONAL REGULATORY PROTEIN DEVR NP_217649	0,355083166
LYRRTLNVARELSRC	MYCOBACTERIUM BOVIS ACYL-COA SYNTHASE GENE; U75685	0,347223973
LVPRSLHWLRPITPT	PUTATIVE CYCLOPROPANE-FATTY-ACYL-PHOSPHOLIPID SYNTHASE UFAA1 NP_854118	0,336462173
HAKARGANILARIMG	3-OXOACYL-[ACYL-CARRIER PROTEIN] SYNTHASE 2 KASB CAA94642	0,321228196
VAIQAITMPHHRMLA	PUTATIVE CYCLOPROPANE-FATTY-ACYL-PHOSPHOLIPID SYNTHASE UFAA1 NP_854118	0,286487386
LHSSGSTAQENAMEQ	PERIPLASMIC PHOSPHATE-BINDING LIPOPROTEIN PSTS2 YP_177769	0,27460646
GLPQNTMKIGIMDEE	MTB81 (publication)	0,207822065
VDLAVPMIGMLKRKA	CONSERVED HYPOTHETICAL PROTEIN TB16.3CAD97060	0,177200498
AAAAMAAAAAPYAGW	YP_177963; PPE FAMILY PROTEIN	0,175466394
RRRYAEITNVAQQFE	CONSERVED HYPOTHETICAL PROTEIN CAB08634	0,173636167
APWGIAVDEAGTVYV	TRANSMEMBRANE SERINE/THREONINE-PROTEIN KINASE D PKND NP_215446	0,170802063
ASLADVLTKNEDVIV	PROBABLE ISOCITRATE DEHYDROGENASE CAA16247	0,168923473
GNIGHGNVGFGNSGL	YP_177963; PPE FAMILY PROTEIN	0,163427776
GNMGVGNIGFGNTGT	YP_177963; PPE FAMILY PROTEIN	0,163389901
WTRFFGVRPVSILYR	POSSIBLE GLYCOSYL TRANSFERASE CAB05418	0,160020344
SLRNAAKAYGEVDEE	MTB48 AAK31576	0,133716167
GTGNIGFFNSGTGNF	YP_177963; PPE FAMILY PROTEIN	0,129615059
FLENFVRSSNLKFQD	CAB10044.1; SECRETED ANTIGEN 85-B FBPB (85B)	0,125545256
LDVIALYIASIATGS	PPE FAMILY PROTEIN CAE55334	0,116756224
QRDEIIEDLHRCHEH	PROBABLE ISOCITRATE DEHYDROGENASE CAA16247	0,106426342
NIGFFNTGTGNWGLF	YP_177963; PPE FAMILY PROTEIN	0,087061854
VCNGGSLTTQQALVA	POSSIBLE GLYCOSYL TRANSFERASE CAB05415	0,083969388
RAHNAGAKNVEERPI	CAB08889.1; IRON-REGULATED CONSERVED HYPOTHETICAL PROTEIN	0,078652353
VHNVAALPGAAYCEM	MYCOBACTERIUM BOVIS MYCOCEROSIC ACID SYNTHASE GENE; M95808	0,07641947
NSATAMADALNGHHP	CAB09453.1; PROBABLE SERINE PROTEASE PEPA	0,073734394
DKKEDEEGVAEAERA	CAB06237.1; CONSERVED HYPOTHETICAL PROTEIN	0,0731842
IGLSMAGSSAMILAA	CAB10044.1; SECRETED ANTIGEN 85-B FBPB (85B)	0,070124924
ANLGVGNIGFANTGS	YP_177963; PPE FAMILY PROTEIN	0,064905876
PLDPSAMVAQVGPQV	CAB09453.1; PROBABLE SERINE PROTEASE PEPA	0,060005742
NVGNYNVGLGNVGIF	YP_177963; PPE FAMILY PROTEIN	0,059154434

Peptides	Protein	Average Index
DDYYDPEPGVPGRSV	MYCOBACTERIUM BOVIS MYCOCEROSIC ACID SYNTHASE GENE; M95808	0,056488007
FLGADDSLYEADTLA	POSSIBLE GLYCOSYL TRANSFERASE CAB05419	0,056074631
DTKLGAEEITRDIPN	RNA POLYMERASE BETA-SUBUNIT AAA21416	0,054634259
YSAGTAVLAGASLVA	POSSIBLE HEMOLYSIN-LIKE PROTEIN CAA17201	0,049220808
CPIETPEGPNIGLIG	RNA POLYMERASE BETA-SUBUNIT AAA21416	0,04712074
EYVWGRQVGDNTINP	MCE-FAMILY PROTEIN MCE1AYP_177701	0,046020521
GIFNLGAANVGAQNL	YP_177963; PPE FAMILY PROTEIN	0,04406468
LDQASQRGLGEAQLG	PERIPLASMIC PHOSPHATE-BINDING LIPOPROTEIN PSTS1 YP_177770	0,043484825
ADLTVIGARDDLMVN	CAA98350.1; CELL SURFACE LIPOPROTEIN MPT83	0,039928437
LQLGNLNIDTRINQS	YP_177963; PPE FAMILY PROTEIN	0,037154078
SETFNGGVGVGASGN	PERIPLASMIC PHOSPHATE-BINDING LIPOPROTEIN PSTS2 YP_177769	0,035845469
STNIRQAGVQYSRAD	CAA17966.1; 10 KDA CULTURE FILTRATE ANTIGEN ESXB	0,034240818
INTGWFNTGNANTGV	YP_177963; PPE FAMILY PROTEIN	0,032366362
VQRSEEAQLVVVGSR	HYPOTHETICAL PROTEIN RV2623 NP_217139	0,029909141
GNLGHGNVGFGNSGL	YP_177963; PPE FAMILY PROTEIN	0,029013584
GGFSEGGCWRTFLTC	MYCOBACTERIUM BOVIS MYCOCEROSIC ACID SYNTHASE GENE; M95808	0,028451818
MARGIDDVVVSVVAS	MYCOBACTERIUM BOVIS MYCOCEROSIC ACID SYNTHASE GENE; M95808	0,027825825
QTPSAAIDSDRWPAV	PUTATIVE CYCLOPROPANE-FATTY-ACYL-PHOSPHOLIPID SYNTHASE UFAA1 NP_854118	0,021093094
GSSTAVFTGLTHEDY	MYCOBACTERIUM BOVIS MYCOCEROSIC ACID SYNTHASE GENE; M95808	0,020125022
VGLGNVGIFNLGAAN	YP_177963; PPE FAMILY PROTEIN	0,017066441
FNSGTGNIGFFNTGT	YP_177963; PPE FAMILY PROTEIN	0,012037167
HAVFNAASQAFNTPA	YP_177963; PPE FAMILY PROTEIN	0,009875688
SPAWDLPTVFGPIAI	PERIPLASMIC PHOSPHATE-BINDING LIPOPROTEIN PSTS2 YP_177769	0,007999217
NIDTRINQSFPVTVN	YP_177963; PPE FAMILY PROTEIN	0,007761387
IPISVGPITISPITL	YP_177963; PPE FAMILY PROTEIN	0,00688814
DLATGTWLLFLGADD	POSSIBLE GLYCOSYL TRANSFERASE CAB05419	0,006667501
ALAISAQQVNYNLPG	PERIPLASMIC PHOSPHATE-BINDING LIPOPROTEIN PSTS1 YP_177770	0,003888854
QNLGLGNLGDGNVGF	YP_177963; PPE FAMILY PROTEIN	0,001228866
VIFRSDKSGTSDNFQ	PERIPLASMIC PHOSPHATE-BINDING LIPOPROTEIN PSTS2 YP_177769	0,001100688
AAVEEGIVAGGGVTL	60 kDa chaperonin 2 P0A521	-0,000398969
PGSTNTGWFNTGNAN	YP_177963; PPE FAMILY PROTEIN	-0,000610216
AGADIVVECGNIAEP	MYCOBACTERIUM BOVIS MYCOCEROSIC ACID SYNTHASE GENE; M95808	-0,000645648
GIDDVVVSVVASPQS	MYCOBACTERIUM BOVIS MYCOCEROSIC ACID SYNTHASE GENE; M95808	-0,001031727
PILHRRRREFKARAA	MTB81 (publication)	-0,003504892
SSTGRCHSFDADADG	MYCOBACTERIUM BOVIS MYCOCEROSIC ACID SYNTHASE GENE; M95808	-0,005254835
NYNVGLGNVGIFNLG	YP_177963; PPE FAMILY PROTEIN	-0,005505278
NLTLQGDVKRFRGFD	MTB48 AAK31576	-0,00574212
GESIVNANTVLDDLN	MCE-FAMILY PROTEIN MCE1AYP_177701	-0,006974907
AVIGNRRRQDSKESK	MTB48 AAK31576	-0,007870634
HFADGWNTFNLTLQG	MTB48 AAK31576	-0,0086001
YGLANLGVGNIGFAN	YP_177963; PPE FAMILY PROTEIN	-0,009416497
GSVACQEFITQLGRN	PUTATIVE ESAT-6 LIKE PROTEIN ESXN YP_177838	-0,011145626
AGTVYVTEHNTNQVV	TRANSMEMBRANE SERINE/THREONINE-PROTEIN KINASE D PKND NP_215446	-0,011221653
ATPADTGLSQSESGI	TRANSMEMBRANE SERINE/THREONINE-PROTEIN KINASE D PKND NP_215446	-0,014465115
FGGKIVTPSPGTPEG	MYCOBACTERIUM BOVIS ACYL-COA SYNTHASE GENE; U75685	-0,016356913
SAKAGLTTLAYTAAT	POSSIBLE HEMOLYSIN-LIKE PROTEIN CAA17201	-0,018593176
ARRNIAVHYDLSNDL	PUTATIVE CYCLOPROPANE-FATTY-ACYL-PHOSPHOLIPID SYNTHASE UFAA1 NP_854118	-0,019546912
LYDFVNNEALPGTDI	MTB81 (publication)	-0,021510044
PGSINTGWFNTGNAN	YP_177963; PPE FAMILY PROTEIN	-0,024359273
MKRGLTVAVAGAAIL	LIPOPROTEIN LPQH PRECURSOR P0A5J0	-0,024756112
HLNPLNIVNLGNVGS	YP_177963; PPE FAMILY PROTEIN	-0,025783132
VSAGIGGAAASLVGF	CAA17582.1; PROBABLE SERINE PROTEASE PEPD	-0,026903461
VNVGFGNVGDFNLGA	YP_177963; PPE FAMILY PROTEIN	-0,026969147
NANVGFGNIGHGNVG	YP_177963; PPE FAMILY PROTEIN	-0,028713017
NQAHTALAGAGSGIA	YP_177963; PPE FAMILY PROTEIN	-0,028790937
GGALYSIGGILYAVR	POSSIBLE HEMOLYSIN-LIKE PROTEIN CAA17201	-0,029063401
TRLGIRLVIVLVRRW	POSSIBLE GLYCOSYL TRANSFERASE CAB05419	-0,029272125
VHIGPTAFLGLGVVD	CAB09453.1; PROBABLE SERINE PROTEASE PEPA	-0,030343907
FVPQAVTPWPNGNDH	MYCOBACTERIUM BOVIS MYCOCEROSIC ACID SYNTHASE GENE; M95808	-0,031416085
TGVANAGNVNTGALI	YP_177963; PPE FAMILY PROTEIN	-0,031631245
PQSRHDIQQLAALGD	MCE-FAMILY PROTEIN MCE1AYP_177701	-0,031866518

Peptides	Protein	Average Index
KPKMHGPAEVAFTCE	MTB81 (publication)	-0,037762727
GTASTGLFNVGGFNT	YP_177963; PPE FAMILY PROTEIN	-0,038659419
IGLANVGNGNVGLGN	YP_177963; PPE FAMILY PROTEIN	-0,038958561
EADLDVLDEHGTVLL	MYCOBACTERIUM BOVIS MYCOCEROSIC ACID SYNTHASE GENE; M95808	-0,039833899
DPGAAAQFNASPVAQ	LOW MOLECULAR WEIGHT T-CELL ANTIGEN TB8.4 NP_215690	-0,042576811
NVGDVNLGAGNLGNL	YP_177963; PPE FAMILY PROTEIN	-0,043863529
ATTVFGGKYVSLTTP	MCE-FAMILY PROTEIN MCE1AYP_177701	-0,045888217
IALYIASIATGSIAL	PPE FAMILY PROTEIN CAE55334	-0,046868415
GGDPVAITIDSVGQT	PERIPLASMIC PHOSPHATE-BINDING LIPOPROTEIN PSTS3 YP_177768	-0,047705698
PDVLVVGNGGPGVTA	PPE FAMILY PROTEIN CAE55504	-0,047740387
GKVIGIRVFSREDED	RNA POLYMERASE BETA-SUBUNIT AAA21416	-0,048538976
LLHFNRLPDELSDVE	MYCOBACTERIUM BOVIS MYCOCEROSIC ACID SYNTHASE GENE; M95808	-0,048856577
PVAITIDSVGQTIAG	PERIPLASMIC PHOSPHATE-BINDING LIPOPROTEIN PSTS3 YP_177768	-0,051128624
AGPTQVLVPRSAIDS	CAA98382.1; IMMUNOGENIC PROTEIN MPT64	-0,051236594
AAGGHNAVFNFPPNG	CAB10044.1; SECRETED ANTIGEN 85-B FBPB (85B)	-0,052533148
ANMGVGNIGFANTGT	YP_177963; PPE FAMILY PROTEIN	-0,052563511
FNSGTGNVGLFNSGT	YP_177963; PPE FAMILY PROTEIN	-0,054017439
VAQGVTNLHTAIMAQ	PPE FAMILY PROTEIN CAE55504	-0,05418693
GVWQGAAAAAMAAAA	YP_177963; PPE FAMILY PROTEIN	-0,05451461
KAPAETQAQRIAITK	CAA17343.1; HEAT SHOCK PROTEIN HSP	-0,054671614
GASLVAVSWAVGSAK	POSSIBLE HEMOLYSIN-LIKE PROTEIN CAA17201	-0,056110531
VATAMIPFLEHDDAN	RNA POLYMERASE BETA-SUBUNIT AAA21416	-0,056489318
ETGAGAGTVATTPAS	PERIPLASMIC PHOSPHATE-BINDING LIPOPROTEIN PSTS1 YP_177770	-0,060261359
AKNVEERPIVGAPVD	CAB08889.1; IRON-REGULATED CONSERVED HYPOTHETICAL PROTEIN	-0,060428811
VHPLLGSHVRLTEEP	MYCOBACTERIUM BOVIS MYCOCEROSIC ACID SYNTHASE GENE; M95808	-0,060440027
REEVDNNCQSILGYV	MTB81 (publication)	-0,060719892
GLAAGLAGVGNIGLG	YP_177963; PPE FAMILY PROTEIN	-0,061182062
PRDDLIQVARVGLVN	ALTERNATE RNA POLYMERASE SIGMA FACTOR SIGF CAB07069	-0,062043534
LFVGGALYSIGGILY	POSSIBLE HEMOLYSIN-LIKE PROTEIN CAA17201	-0,062076714
VVPDIVIPAFGGGTA	YP_177963; PPE FAMILY PROTEIN	-0,063204947
FNTGVANPGSYNTGS	YP_177963; PPE FAMILY PROTEIN	-0,063584088
SPVTLAETGSTLLYP	PERIPLASMIC PHOSPHATE-BINDING LIPOPROTEIN PSTS1 YP_177770	-0,063974856
WGGPPVASEVRGDAA	POSSIBLE GLYCOSYL TRANSFERASE CAB05415	-0,064756397
DSLTSQVGGRSIGVY	PROBABLE CUTINASE PRECURSOR CFP21 NP_216500	-0,065314386
TNTGWFNTGNANTGV	YP_177963; PPE FAMILY PROTEIN	-0,066367192
GGNLGGQNLGLGNLG	YP_177963; PPE FAMILY PROTEIN	-0,066677883
RQTIEGLRAAGADIV	MYCOBACTERIUM BOVIS MYCOCEROSIC ACID SYNTHASE GENE; M95808	-0,067168809
QELDEISTNIRQAGV	CAA17966.1; 10 KDA CULTURE FILTRATE ANTIGEN ESXB	-0,067170483
SRWGGFLDDVAGFDA	MYCOBACTERIUM BOVIS MYCOCEROSIC ACID SYNTHASE GENE; M95808	-0,067775736
DANLAVTLPPGLTDE	MYCOBACTERIUM BOVIS MYCOCEROSIC ACID SYNTHASE GENE; M95808	-0,068422308
IVEVVAGGAAANAGV	CAA17582.1; PROBABLE SERINE PROTEASE PEPD	-0,069143445
STIGAGQSGLGDNGY	PERIPLASMIC PHOSPHATE-BINDING LIPOPROTEIN PSTS3 YP_177768	-0,07015227
HEVLIQAGAGEGSAI	SECRETED L-ALANINE DEHYDROGENASE ALD CAA15575	-0,070399804
RRILFVAEAVTLAHV	POSSIBLE GLYCOSYL TRANSFERASE CAB05415	-0,07185028
ADALNGHHPGDVISV	CAB09453.1; PROBABLE SERINE PROTEASE PEPA	-0,072173031
PEAAGPTQVLVPRSA	CAA98382.1; IMMUNOGENIC PROTEIN MPT64	-0,072206766
VGVLAAGALVLSACG	PERIPLASMIC PHOSPHATE-BINDING LIPOPROTEIN PSTS3 YP_177768	-0,072573497
GADGKLKDTKAVNPE	PROBABLE ISOCITRATE DEHYDROGENASE CAA16247	-0,072851137
TDTDNNRVVKLEAES	TRANSMEMBRANE SERINE/THREONINE-PROTEIN KINASE D PKND NP_215446	-0,073012618
AAAWGGSGSEAYQGV	CAE55648	-0,073115281
ATTGQASAKVDCGGK	PERIPLASMIC PHOSPHATE-BINDING LIPOPROTEIN PSTS3 YP_177768	-0,07570331
EGLTTGRSCQTDVDG	POSSIBLE HEMOLYSIN CAA16235	-0,076474667
SPSRKTGELDNRGSQ	PROBABLE ISOCITRATE DEHYDROGENASE CAA16247	-0,077357188
IVRIVDSDTCIECPD	MYCOBACTERIUM BOVIS ACYL-COA SYNTHASE GENE; U75685	-0,077492567
QGLEYVVAFLGALQA	MYCOBACTERIUM BOVIS ACYL-COA SYNTHASE GENE; U75685	-0,079507215
VLTACGGGTNSSSSG	PERIPLASMIC PHOSPHATE-BINDING LIPOPROTEIN PSTS2 YP_177769	-0,080074312
AGAGSGVLNVGTLNS	YP_177963; PPE FAMILY PROTEIN	-0,080756219
GNWGLFNSGSFNTGI	YP_177963; PPE FAMILY PROTEIN	-0,081362342
WIGSSAGLMVAAASP	CAE55371.1; PPE FAMILY PROTEIN	-0,082471726
VVASYNEFGGLSNTI	POSSIBLE GLYCOSYL TRANSFERASE CAB05419	-0,082831157
YNLGFANVGDVNLGA	YP_177963; PPE FAMILY PROTEIN	-0,083184244
LQPFTVAPINLNPTT	YP_177963; PPE FAMILY PROTEIN	-0,083261527
NETDFAGSDVPLNPS	PERIPLASMIC PHOSPHATE-BINDING LIPOPROTEIN PSTS2 YP_177769	-0,083560136

Peptides	Protein	Average Index
QYSRADEEQQQALSS	CAA17966.1; 10 KDA CULTURE FILTRATE ANTIGEN ESXB	-0,0850419
ANRAVKPTGSAAIGL	CAB10044.1; SECRETED ANTIGEN 85-B FBPB (85B)	-0,085168708
PNGNDHTPKRVAVSS	MYCOBACTERIUM BOVIS MYCOCEROSIC ACID SYNTHASE GENE; M95808	-0,085238688
DVAYRPMAPNFDDSI	MTB81 (publication)	-0,085672516
GYPAVLTSPVSFLQR	MYCOBACTERIUM BOVIS ACYL-COA SYNTHASE GENE; U75685	-0,086618578
ASGNLGSGNVGVGNI	PPE FAMILY PROTEIN CAE55504	-0,087404461
TLGYTSGTGQGNASA	LIPOPROTEIN LPQH PRECURSOR P0A5J0	-0,087698792
PIINYEYAIVNNRQK	PERIPLASMIC PHOSPHATE-BINDING LIPOPROTEIN PSTS1 YP_177770	-0,088807903
ITTSGVDAEITTTAG	MTB81 (publication)	-0,09007481
NGSGAGVTQFLNNET	PERIPLASMIC PHOSPHATE-BINDING LIPOPROTEIN PSTS2 YP_177769	-0,090446532
DGFVFGEGGALLLIE	3-OXOACYL-[ACYL-CARRIER PROTEIN] SYNTHASE 2 KASB CAA94642	-0,090625872
LSWSVQDTPPNDQAG	MYCOBACTERIUM BOVIS MYCOCEROSIC ACID SYNTHASE GENE; M95808	-0,090950595
NGDWYVARLTPSPLG	MYCOBACTERIUM BOVIS MYCOCEROSIC ACID SYNTHASE GENE; M95808	-0,091075676
SGNNGTSALLQTTDG	PERIPLASMIC PHOSPHATE-BINDING LIPOPROTEIN PSTS2 YP_177769	-0,091569753
AATAGRNHLKNVPAN	POSSIBLE GLYCOSYL TRANSFERASE CAB05418	-0,091671887
VGDVIQAGDLIAVIS	CAB08316.1; BIOTINYLATED PROTEIN TB7.3	-0,091802362
LNSVSSLNLDGPTLA	PERIPLASMIC PHOSPHATE-BINDING LIPOPROTEIN PSTS3 YP_177768	-0,092263374
GVAVDNSGNVYVTDT	TRANSMEMBRANE SERINE/THREONINE-PROTEIN KINASE D PKND NP_215446	-0,092363728
RIGAEVRDGDILVGK	RNA POLYMERASE BETA-SUBUNIT AAA21416	-0,092574985
NTRLGLFPFRRGLTF	MYCOBACTERIUM BOVIS MYCOCEROSIC ACID SYNTHASE GENE; M95808	-0,092592434
VAADLQQGAVQGVND	PPE FAMILY PROTEIN CAE55504	-0,09314742
DVVGEAGSVAEAMAR	TWO COMPONENT TRANSCRIPTIONAL REGULATORY PROTEIN DEVR NP_217649	-0,093369942
QPNPKARQTIEGLRA	MYCOBACTERIUM BOVIS MYCOCEROSIC ACID SYNTHASE GENE; M95808	-0,093396268
LVAGVPVIGVAGNLD	POSSIBLE GLYCOSYL TRANSFERASE CAB05415	-0,09370187
SGFSNSGSGMSGVLN	YP_177963; PPE FAMILY PROTEIN	-0,094815
TGQGNSLKVGNADVV	CAA98373.1; MAJOR SECRETED IMMUNOGENIC PROTEIN	-0,095104118
DQAITSAGRHPDSDI	CAB01474.1; CONSERVED HYPOTHETICAL PROTEIN CFP17	-0,097145624
VHPGYTATFLETPSQ	PPE FAMILY PROTEIN CAE55504	-0,097196198
SPIINLGLADVGNYN	YP_177963; PPE FAMILY PROTEIN	-0,097886731
IKAGDKVLIHSATGG	MYCOBACTERIUM BOVIS MYCOCEROSIC ACID SYNTHASE GENE; M95808	-0,097927132
VGFGNLGHGNVGFGN	YP_177963; PPE FAMILY PROTEIN	-0,098142203
AEATVYVATSKPGQP	MYCOBACTERIUM BOVIS ACYL-COA SYNTHASE GENE; U75685	-0,098255998
TLLDVAQANLGEAAG	CONSERVED HYPOTHETICAL PROTEIN TB9.8 CAD93159	-0,098432504
TKSGGTRTGNVTLAE	CAB09453.1; PROBABLE SERINE PROTEASE PEPA	-0,098580056
GRDGVVIVYGPRVGD	MYCOBACTERIUM BOVIS MYCOCEROSIC ACID SYNTHASE GENE; M95808	-0,099482892
MSQDMAQTFTSIAIS	CONSERVED HYPOTHETICAL PROTEIN CAA15739	-0,099936523
VAALPGAAYCEMALA	MYCOBACTERIUM BOVIS MYCOCEROSIC ACID SYNTHASE GENE; M95808	-0,100032724
QMLLLDEQTPIDAVA	MYCOBACTERIUM BOVIS MYCOCEROSIC ACID SYNTHASE GENE; M95808	-0,100187394
TIPGFTIPGGTLIPQ	YP_177963; PPE FAMILY PROTEIN	-0,100365757
LLSEGLTNKQIADRM	TWO COMPONENT TRANSCRIPTIONAL REGULATORY PROTEIN DEVR NP_217649	-0,100558272
AVGSAKAGLTTLAYT	POSSIBLE HEMOLYSIN-LIKE PROTEIN CAA17201	-0,101270292
QAAAGTVNIGASDAY	PERIPLASMIC PHOSPHATE-BINDING LIPOPROTEIN PSTS1 YP_177770	-0,101452603
QSVCPILAEPGGSFN	CONSERVED HYPOTHETICAL PROTEIN CAA15739	-0,102022931
LNAGLGNVGNYNVGL	YP_177963; PPE FAMILY PROTEIN	-0,102175388
FRLPLLFVNALGDNW	YP_177963; PPE FAMILY PROTEIN	-0,102445405
SLAGSSTAVFTGLTH	MYCOBACTERIUM BOVIS MYCOCEROSIC ACID SYNTHASE GENE; M95808	-0,102548978
EVGDGEAGSWLLIDT	MYCOBACTERIUM BOVIS MYCOCEROSIC ACID SYNTHASE GENE; M95808	-0,102631435
QLGNSSGNFLLPDAQ	PERIPLASMIC PHOSPHATE-BINDING LIPOPROTEIN PSTS1 YP_177770	-0,10278039
REVAVDVASHSPQVD	MYCOBACTERIUM BOVIS MYCOCEROSIC ACID SYNTHASE GENE; M95808	-0,102884629
RAAGGSASRANEYAD	ALTERNATE RNA POLYMERASE SIGMA FACTOR SIGF CAB07069	-0,103119564
NVDAGLAAGFSGRAQ	MTB81 (publication)	-0,105134662
MSYEWPEDRQSLSWT	CONSERVED HYPOTHETICAL PROTEIN TB16.3CAD97060	-0,106038748
DARDTKLGAEEITRD	RNA POLYMERASE BETA-SUBUNIT AAA21416	-0,106056173
SADVRASLERMAPLV	MTB81 (publication)	-0,106062226
VGVGNIGNDNIGNAN	PPE FAMILY PROTEIN CAE55504	-0,106098687
ANVGNGNVGLGNIGN	YP_177963; PPE FAMILY PROTEIN	-0,106723311
AGDLAIAAHDALTTS	TRANSMEMBRANE SERINE/THREONINE-PROTEIN KINASE D PKND NP_215446	-0,107232369
TPALVSGAGNVGQQL	YP_177963; PPE FAMILY PROTEIN	-0,107254223
NRAERQILIDNNALG	PPE FAMILY PROTEIN CAE55489	-0,107673239
VTLGAAFGGLTTAHT	MYCOBACTERIUM BOVIS MYCOCEROSIC ACID SYNTHASE GENE; M95808	-0,107678518
DALTTSEQHQATTIL	TRANSMEMBRANE SERINE/THREONINE-PROTEIN KINASE D PKND	-0,108010439

Peptides	Protein	Average Index
	NP_215446	
LKALGWTSEQIVERF	RNA POLYMERASE BETA-SUBUNIT AAA21416	-0,108333118
AARGTTGGGGTRSGT	PPE FAMILY PROTEIN CAE55334	-0,108818947
SRMPQSRHDIQQLAA	MCE-FAMILY PROTEIN MCE1AYP_177701	-0,10888443
RTVGELIQNQIRVGM	RNA POLYMERASE BETA-SUBUNIT AAA21416	-0,109092024
AALAGAGSGVLNVGT	YP_177963; PPE FAMILY PROTEIN	-0,109358567
VVDNNGNGARVQRVV	CAB09453.1; PROBABLE SERINE PROTEASE PEPA	-0,109702926
PGKLNKARVPPYKTA	MCE-FAMILY PROTEIN MCE1AYP_177701	-0,110003935
TACSSGLMAVHLACR	MYCOBACTERIUM BOVIS MYCOCEROSIC ACID SYNTHASE GENE; M95808	-0,110923365
LDAILAGASGYVVKD	TWO COMPONENT TRANSCRIPTIONAL REGULATORY PROTEIN DEVR NP_217649	-0,111018335
IIVGIDDSPAAQVAV	HYPOTHETICAL PROTEIN RV2623 NP_217139	-0,111313912
YCGNGTPNELGGANI	CAB10044.1; SECRETED ANTIGEN 85-B FBPB (85B)	-0,112266004
GVPSAPLGSAIGGAE	MTB48 AAK31576	-0,112681112
QEIENSDSDYDREKL	60 kDa chaperonin 2 P0A521	-0,113677548
VHLACRSLHDGERDL	MYCOBACTERIUM BOVIS MYCOCEROSIC ACID SYNTHASE GENE; M95808	-0,113985899
RWSWLLSVLAAVGLG	CAB09453.1; PROBABLE SERINE PROTEASE PEPA	-0,114095674
LASPYVVTGLNNSVA	MYCOBACTERIUM BOVIS MYCOCEROSIC ACID SYNTHASE GENE; M95808	-0,114399131
NIGNANIGFGNRGDA	PPE FAMILY PROTEIN CAE55504	-0,114672487
RGWIHVYSAGTAVLA	POSSIBLE HEMOLYSIN-LIKE PROTEIN CAA17201	-0,114830203
LAKLAGGVAVIKAGA	60 kDa chaperonin 2 P0A521	-0,115051895
VVGHSMGESAAAVVA	MYCOBACTERIUM BOVIS MYCOCEROSIC ACID SYNTHASE GENE; M95808	-0,115102422
VNVFEQACPGQTLNY	PERIPLASMIC PHOSPHATE-BINDING LIPOPROTEIN PSTS3 YP_177768	-0,115282041
AAPVAVAAAGAAPAG	CAB07109.1; PROBABLE 50S RIBOSOMAL PROTEIN L7/L12 RPLL	-0,115311343
TGHSPAVPNALQASL	TRANSMEMBRANE SERINE/THREONINE-PROTEIN KINASE D PKND NP_215446	-0,115468468
NLGSGNVGVGNIGND	PPE FAMILY PROTEIN CAE55504	-0,115733024
VGSVGETVAQLARTP	HYPOTHETICAL PROTEIN RV2623 NP_217139	-0,116011147
NIGLIGSLSVYARVN	RNA POLYMERASE BETA-SUBUNIT AAA21416	-0,116890348
QGAAAAAMAAAAAPY	YP_177963; PPE FAMILY PROTEIN	-0,117531951
PANVVGTRQTLQGAS	CAA98373.1; MAJOR SECRETED IMMUNOGENIC PROTEIN	-0,117565879
FGKPANPDVLVVGNG	PPE FAMILY PROTEIN CAE55504	-0,11790677
WGGSGSEAYQGVQQK	CAE55648	-0,117913684
HPRYSPPPQQPGTPG	CAA17582.1; PROBABLE SERINE PROTEASE PEPD	-0,118319063
GLSVADLVLVAPGSI	MYCOBACTERIUM BOVIS ACYL-COA SYNTHASE GENE; U75685	-0,11834566
NGVTLGYTSGTGQGN	LIPOPROTEIN LPQH PRECURSOR P0A5J0	-0,118370804
VDCGGKKTLKASGST	PERIPLASMIC PHOSPHATE-BINDING LIPOPROTEIN PSTS3 YP_177768	-0,118386183
AKAPKLVSNSLVAHM	SECRETED L-ALANINE DEHYDROGENASE ALD CAA15575	-0,118601623
GNIGDVNLGFGNIGG	YP_177963; PPE FAMILY PROTEIN	-0,118680095
FADTDGIPPPNSALV	MYCOBACTERIUM BOVIS ACYL-COA SYNTHASE GENE; U75685	-0,121999361
WMSVTAGQAELTAAQ	CAE55371.1; PPE FAMILY PROTEIN	-0,122064629
SDVPLSKDEAAAAQR	PERIPLASMIC PHOSPHATE-BINDING LIPOPROTEIN PSTS3 YP_177768	-0,12228096
GIMDALFTGLIAIHG	MTB81 (publication)	-0,122344982
HGAMIRAQAASLEAE	PUTATIVE ESAT-6 LIKE PROTEIN ESXN YP_177838	-0,122525937
PPVASEVRGDAAIST	POSSIBLE GLYCOSYL TRANSFERASE CAB05415	-0,122603641
NIGNANVGFGNIGHG	YP_177963; PPE FAMILY PROTEIN	-0,122930709
MAGYHSGASAAAAAL	YP_177963; PPE FAMILY PROTEIN	-0,123571443
DNATLLATPADTGLS	TRANSMEMBRANE SERINE/THREONINE-PROTEIN KINASE D PKND NP_215446	-0,12428819
SSGAALLGSPGQGAY	MYCOBACTERIUM BOVIS MYCOCEROSIC ACID SYNTHASE GENE; M95808	-0,124817347
DIKVQFQSGGNNSPA	CAB10044.1; SECRETED ANTIGEN 85-B FBPB (85B)	-0,125229582
LDSSVTTARTINAQQ	MCE-FAMILY PROTEIN MCE1AYP_177701	-0,126157782
GNVGDHLSGLLASNV	YP_177963; PPE FAMILY PROTEIN	-0,126513753
RGDSDQDAMARLGAI	MYCOBACTERIUM BOVIS ACYL-COA SYNTHASE GENE; U75685	-0,127445645
AQLASGNLGSGNVGV	PPE FAMILY PROTEIN CAE55504	-0,128116115
PISVIFRSDKSGTSD	PERIPLASMIC PHOSPHATE-BINDING LIPOPROTEIN PSTS2 YP_177769	-0,128228167
DGNPPEVKSVGLGNV	LIPOPROTEIN LPQH PRECURSOR P0A5J0	-0,128567874
PAGIKIEASDISVAA	PROBABLE ISOCITRATE DEHYDROGENASE CAA16247	-0,128674924
LAVTLPPGLTDEQAI	MYCOBACTERIUM BOVIS MYCOCEROSIC ACID SYNTHASE GENE; M95808	-0,12994407
PNGYTEPILHRRRRE	MTB81 (publication)	-0,130436128
SGSEAYQGVQQKWDA	CAE55648	-0,130618607
NNIGIGLTGDNLTGI	YP_177963; PPE FAMILY PROTEIN	-0,130649363
GWFNTGNANTGVANA	YP_177963; PPE FAMILY PROTEIN	-0,13068398
NGSITQWNNPAIQAL	PERIPLASMIC PHOSPHATE-BINDING LIPOPROTEIN PSTS3 YP_177768	-0,130904559

Peptides	Protein	Average Index
LSEGDMAAHKGLMNI	PERIPLASMIC PHOSPHATE-BINDING LIPOPROTEIN PSTS1 YP_177770	-0,131356405
VVLEKKWGAPTITND	60 kDa chaperonin 2 P0A521	-0,131755035
IYNNRAERQILIDNN	PPE FAMILY PROTEIN CAE55489	-0,131762821
GDMAAHKGLMNIALA	PERIPLASMIC PHOSPHATE-BINDING LIPOPROTEIN PSTS1 YP_177770	-0,131978597
AIPGFPALPGLPSIP	CAB06237.1; CONSERVED HYPOTHETICAL PROTEIN	-0,132006213
LYGNAGGLGPTQGHP	PPE FAMILY PROTEIN CAE55334	-0,132337877
LNPGVNLPGTAVVPL	PERIPLASMIC PHOSPHATE-BINDING LIPOPROTEIN PSTS1 YP_177770	-0,132574675
ITGQFRFGPITVSNV	YP_177963; PPE FAMILY PROTEIN	-0,132847914
AGTAASQRGQEGTVL	CAB06237.1; CONSERVED HYPOTHETICAL PROTEIN	-0,133416602
ALATNGAAIGIGLVA	MCE-FAMILY PROTEIN MCE1AYP_177701	-0,133715758
LAAGTLVLTACGGGT	PERIPLASMIC PHOSPHATE-BINDING LIPOPROTEIN PSTS2 YP_177769	-0,133805408
QGNSDRRAPKAVKEY	PROBABLE ISOCITRATE DEHYDROGENASE CAA16247	-0,134004055
INIDKLRQLDAEFCG	SECRETED L-ALANINE DEHYDROGENASE ALD CAA15575	-0,134215152
LDIHETMVIPPIVFL	YP_177963; PPE FAMILY PROTEIN	-0,134355053
FPGGLLPSTQAIIDI	PUTATIVE CYCLOPROPANE-FATTY-ACYL-PHOSPHOLIPID SYNTHASE UFAA1 NP_854118	-0,134768309
ADTGLSQSESGIAGA	TRANSMEMBRANE SERINE/THREONINE-PROTEIN KINASE D PKND NP_215446	-0,135349276
AAASAIQGNVTSIHS	CAE55648	-0,135590958
NIGFGNTGTNNLGIG	YP_177963; PPE FAMILY PROTEIN	-0,135591973
DDALKVVEQASLRAG	HYPOTHETICAL PROTEIN RV2623 NP_217139	-0,13563172
SFAERGVTLGAAFGG	MYCOBACTERIUM BOVIS MYCOCEROSIC ACID SYNTHASE GENE; M95808	-0,135751614
NNGNGARVQRVVGSA	CAB09453.1; PROBABLE SERINE PROTEASE PEPA	-0,136124541
GRALIATGEQVLVNE	CONSERVED HYPOTHETICAL PROTEIN CAB08634	-0,136437708
GEKRIPLDVAEGDTV	CAB01005.1; 10 KDA CHAPERONIN GROES	-0,136620178
AFVDSLTSQVGGRSI	PROBABLE CUTINASE PRECURSOR CFP21 NP_216500	-0,136781366
ANGMGATVTVLDINI	SECRETED L-ALANINE DEHYDROGENASE ALD CAA15575	-0,136870502
PLGHEERRTAVLDPD	MYCOBACTERIUM BOVIS MYCOCEROSIC ACID SYNTHASE GENE; M95808	-0,137038306
FGPITVSNVQLNPFN	YP_177963; PPE FAMILY PROTEIN	-0,137765691
PVNYQNFAVTNDGVI	CAA98382.1; IMMUNOGENIC PROTEIN MPT64	-0,137874703
SPPAQALGGGVGAFL	YP_177963; PPE FAMILY PROTEIN	-0,137888861
ARTISEAGQAMASTE	CAE55648	-0,138211116
AAAARSAVVVCNGGS	POSSIBLE GLYCOSYL TRANSFERASE CAB05415	-0,138685703
TVSKVAVSVGDVIQA	CAB08316.1; BIOTINYLATED PROTEIN TB7.3	-0,138932983
AEPDTADRLVSAATA	MYCOBACTERIUM BOVIS MYCOCEROSIC ACID SYNTHASE GENE; M95808	-0,139445217
FPKYPLNVFATANAI	PPE FAMILY PROTEIN CAE55504	-0,139492397
STLGSAPAVISGFGN	YP_177963; PPE FAMILY PROTEIN	-0,139765803
GEAAEVRDITFEQML	MYCOBACTERIUM BOVIS MYCOCEROSIC ACID SYNTHASE GENE; M95808	-0,13979172
AGTVNIGASDAYLSE	PERIPLASMIC PHOSPHATE-BINDING LIPOPROTEIN PSTS1 YP_177770	-0,139818243
IVLGGYSQGATVIDL	PROBABLE CUTINASE PRECURSOR CFP21 NP_216500	-0,139830902
AVLLEPHACVAASAQ	MYCOBACTERIUM BOVIS MYCOCEROSIC ACID SYNTHASE GENE; M95808	-0,140259748
AKEKPQEGTVVAVGP	CAB01005.1; 10 KDA CHAPERONIN GROES	-0,140458222
ISAQQVNYNLPGVSE	PERIPLASMIC PHOSPHATE-BINDING LIPOPROTEIN PSTS1 YP_177770	-0,14061412
TPYVGWLAATAALAK	PPE FAMILY PROTEIN CAE55334	-0,14078034
YVAADAAAASTYTGF	CONSERVED HYPOTHETICAL PROTEIN TB9.8 CAD93159	-0,141167914
GNFGLFNSGSFNTGI	YP_177963; PPE FAMILY PROTEIN	-0,141435078
EYGLLRGSWLTMIRL	POSSIBLE HEMOLYSIN CAA16235	-0,141567374
AAGAQLVGTADQVWA	SECRETED L-ALANINE DEHYDROGENASE ALD CAA15575	-0,141912976
NARDELQAQIDKWHR	MTB81 (publication)	-0,142558533
VDQGLQRRDPRWEAD	MYCOBACTERIUM BOVIS MYCOCEROSIC ACID SYNTHASE GENE; M95808	-0,143046173
QPLGGKAQFGGQRFG	RNA POLYMERASE BETA-SUBUNIT AAA21416	-0,143339431
PIDAGTAASQRGQEG	CAB06237.1; CONSERVED HYPOTHETICAL PROTEIN	-0,143359944
VYATLGTSGGRNLLQ	POSSIBLE GLYCOSYL TRANSFERASE CAB05415	-0,143540252
SGYFADTDGIPPPNS	MYCOBACTERIUM BOVIS ACYL-COA SYNTHASE GENE; U75685	-0,143917417
GYHEFFHACTAVAAI	POSSIBLE HEMOLYSIN-LIKE PROTEIN CAA17201	-0,144096138
EDEEGVAEAERAPID	CAB06237.1; CONSERVED HYPOTHETICAL PROTEIN	-0,144276731
LPVRIGGHLLEEFDH	3-OXOACYL-[ACYL-CARRIER PROTEIN] SYNTHASE 2 KASB CAA94642	-0,14450529
STGLFNAGNFNTGVA	YP_177963; PPE FAMILY PROTEIN	-0,144623456
GILSAAAFAAPAQAN	CONSERVED HYPOTHETICAL PROTEIN CAA15739	-0,144655231
GVADLVPTATLLDTY	MCE-FAMILY PROTEIN MCE1AYP_177701	-0,145027233
NTGNANTGVANSGNV	YP_177963; PPE FAMILY PROTEIN	-0,145205874
SGIRTLDDPFVEEFD	3-OXOACYL-[ACYL-CARRIER PROTEIN] SYNTHASE 2 KASB CAA94642	-0,145387326
GPVHAVFNAASQAFN	YP_177963; PPE FAMILY PROTEIN	-0,145728112
PNGYTFKEDEYPSTA	MYCOBACTERIUM BOVIS ACYL-COA SYNTHASE GENE; U75685	-0,145756358

Peptides	Protein	Average Index
TSGKVRRGACVEQYR	MYCOBACTERIUM BOVIS ACYL-COA SYNTHASE GENE; U75685	-0,146267325
VAANILGQNSAAIAA	PPE FAMILY PROTEIN CAE55334	-0,146472508
GDVITAVDGAPINSA	CAB09453.1; PROBABLE SERINE PROTEASE PEPA	-0,14649955
AGDIAGLGQGRAGGG	MTB48 AAK31576	-0,146824298
VSSPDVLTTYILLPS	PPE FAMILY PROTEIN CAE55504	-0,146846528
TSTAPNAAPEPVARR	POSSIBLE GLYCOSYL TRANSFERASE CAB05418	-0,147328495
QANAHGQKVQAAGNN	PUTATIVE ESAT-6 LIKE PROTEIN ESXN YP_177838	-0,147794105
TANGSGAGISEFNGN	PERIPLASMIC PHOSPHATE-BINDING LIPOPROTEIN PSTS3 YP_177768	-0,148369335
DVSPRQMVSVATAMI	RNA POLYMERASE BETA-SUBUNIT AAA21416	-0,148418571
ANAGNVNTGALITGN	YP_177963; PPE FAMILY PROTEIN	-0,148440964
GFLMPPSDGSGVTPG	MTB48 AAK31576	-0,149596083
ARTPAHAAHHLVEGV	POSSIBLE HEMOLYSIN-LIKE PROTEIN CAA17201	-0,149624333
GWLNTGNTNTGIANS	YP_177963; PPE FAMILY PROTEIN	-0,149842336
DQNVTGSVVCTTAAG	LIPOPROTEIN LPQH PRECURSOR P0A5J0	-0,150037272
QASSPMVGTLSGLGN	YP_177963; PPE FAMILY PROTEIN	-0,150228784
TAVVPLHRSDGSGDT	PERIPLASMIC PHOSPHATE-BINDING LIPOPROTEIN PSTS1 YP_177770	-0,150301544
GEADAAICGGVETRI	3-OXOACYL-[ACYL-CARRIER PROTEIN] SYNTHASE 2 KASB CAA94642	-0,150309891
VDPVKLNLTLSAAAE	MCE-FAMILY PROTEIN MCE1AYP_177701	-0,150569316
SPLALATNGAAIGIG	MCE-FAMILY PROTEIN MCE1AYP_177701	-0,150593753
GAAPAGAAVEAAEEQ	CAB07109.1; PROBABLE 50S RIBOSOMAL PROTEIN L7/L12 RPLL	-0,15093326
AKIMGQGNDLVLDTS	PERIPLASMIC PHOSPHATE-BINDING LIPOPROTEIN PSTS2 YP_177769	-0,152272411
ARMQREADTAGRLTE	TRANSMEMBRANE SERINE/THREONINE-PROTEIN KINASE D PKND NP_215446	-0,15242811
VVPVLNARFALNAAN	MTB81 (publication)	-0,15271346
WPGRLLGSVSSGLLR	HYPOTHETICAL PROTEIN RV2623 NP_217139	-0,152754188
PHIVPIHDYGEINGQ	TRANSMEMBRANE SERINE/THREONINE-PROTEIN KINASE D PKND NP_215446	-0,15301679
VVVTKVDDRPINSAD	CAA17582.1; PROBABLE SERINE PROTEASE PEPD	-0,153402863
AVVVCNGGSLTTQQA	POSSIBLE GLYCOSYL TRANSFERASE CAB05415	-0,1534697
QRDVMAREVAVDVAS	MYCOBACTERIUM BOVIS MYCOCEROSIC ACID SYNTHASE GENE; M95808	-0,153609057
VTVGPILINGVNIPA	YP_177963; PPE FAMILY PROTEIN	-0,15377046
AGVQYSRADEEQQQA	CAA17966.1; 10 KDA CULTURE FILTRATE ANTIGEN ESXB	-0,153780467
ASLRAGPPTVHSEIV	HYPOTHETICAL PROTEIN RV2623 NP_217139	-0,154388961
MFGYHSAASAVATQL	PPE FAMILY PROTEIN CAE55504	-0,154727107
PAAPVTTAAMADPAA	CAA98350.1; CELL SURFACE LIPOPROTEIN MPT83	-0,155135148
VDFDTESLSAGHAKP	MYCOBACTERIUM BOVIS ACYL-COA SYNTHASE GENE; U75685	-0,155488219
ACQIQMSDPAYNINI	CAA98382.1; IMMUNOGENIC PROTEIN MPT64	-0,155585469
TRGLQHRGKLDGTPE	CAA17111.1; PROBABLE ISOCITRATE DEHYDROGENASE	-0,155650973
TVAGPLAADSRGGNW	TRANSMEMBRANE SERINE/THREONINE-PROTEIN KINASE D PKND NP_215446	-0,155736549
TDAIAGRVAQIRQEI	60 kDa chaperonin 2 P0A521	-0,155897569
ITVWNDPQIQALNSG	PERIPLASMIC PHOSPHATE-BINDING LIPOPROTEIN PSTS2 YP_177769	-0,155904103
VALEAPLKQIAFNSG	60 kDa chaperonin 2 P0A521	-0,155982326
AARLPDELLEAHANA	RNA POLYMERASE BETA-SUBUNIT AAA21416	-0,156109294
RQFLTEIGYLLPEPD	MTB81 (publication)	-0,156250054
PGERIHVVFRSDESG	PERIPLASMIC PHOSPHATE-BINDING LIPOPROTEIN PSTS3 YP_177768	-0,156677109
VSILQPFTVAPINLN	YP_177963; PPE FAMILY PROTEIN	-0,156853772
GRIVLTARSQPNPKA	MYCOBACTERIUM BOVIS MYCOCEROSIC ACID SYNTHASE GENE; M95808	-0,156875678
SVRPAGAGDIAGLGQ	MTB48 AAK31576	-0,156977182
PFTGLNDPDGVAVDN	TRANSMEMBRANE SERINE/THREONINE-PROTEIN KINASE D PKND NP_215446	-0,157000245
GQNSAAIAATQAEYA	PPE FAMILY PROTEIN CAE55334	-0,157101811
QREADTAGRLTEPHI	TRANSMEMBRANE SERINE/THREONINE-PROTEIN KINASE D PKND NP_215446	-0,157181758
KMEIPVLAEAAGTVS	CAB08316.1; BIOTINYLATED PROTEIN TB7.3	-0,157397333
ELPSSGFFNTGGGGG	YP_177963; PPE FAMILY PROTEIN	-0,157760609
QTTMTVPGGVEVPVE	RNA POLYMERASE BETA-SUBUNIT AAA21416	-0,157892817
LTTAHTPRREPRRCW	MYCOBACTERIUM BOVIS MYCOCEROSIC ACID SYNTHASE GENE; M95808	-0,157918102
RHAEFRLENNEFNVV	CAB01474.1; CONSERVED HYPOTHETICAL PROTEIN CFP17	-0,158220641
GPTYNKVRGDKVIAY	MTB81 (publication)	-0,158512798
SYNTGVGNAGTASTG	YP_177963; PPE FAMILY PROTEIN	-0,158803081
PDKSTGLANPGQFAG	MTB81 (publication)	-0,158807036
GDVLVDADGKAMLFD	RNA POLYMERASE BETA-SUBUNIT AAA21416	-0,158814971
RALAMSNLLGQNAAA	YP_177963; PPE FAMILY PROTEIN	-0,15916758
FNSGDGGVSGFGNFG	YP_177963; PPE FAMILY PROTEIN	-0,159729494

Peptides	Protein	Average Index
RYKVNKKLGLHVGEP	RNA POLYMERASE BETA-SUBUNIT AAA21416	-0,159813284
PVCDGAYWVDNLRNT	MYCOBACTERIUM BOVIS MYCOCEROSIC ACID SYNTHASE GENE; M95808	-0,159866261
NLKFQDAYNAAGGHN	CAB10044.1; SECRETED ANTIGEN 85-B FBPB (85B)	-0,159896506
ADTLAEEQAAAPAAS	MYCOBACTERIUM BOVIS MYCOCEROSIC ACID SYNTHASE GENE; M95808	-0,160133407
EYPSTAYLQYTSGST	MYCOBACTERIUM BOVIS ACYL-COA SYNTHASE GENE; U75685	-0,160320323
EQSTGKIYFDVTGPS	CAB06500.1; IMMUNOGENIC PROTEIN MPT63	-0,160671095
GLSQSESGIAGAGTG	TRANSMEMBRANE SERINE/THREONINE-PROTEIN KINASE D PKND NP_215446	-0,160800792
GSTAQANAMTRFVNV	PERIPLASMIC PHOSPHATE-BINDING LIPOPROTEIN PSTS3 YP_177768	-0,160972243
ELKGTDTGQACQIQM	CAA98382.1; IMMUNOGENIC PROTEIN MPT64	-0,161056457
AMNRGVDLATGTWLL	POSSIBLE GLYCOSYL TRANSFERASE CAB05419	-0,161100481
GVVAEKVRNLPAGHG	60 kDa chaperonin 2 P0A521	-0,162246394
TVAQLARTPVIVARE	HYPOTHETICAL PROTEIN RV2623 NP_217139	-0,162411059
DTAAQLTSAGREAAA	MTB48 AAK31576	-0,162495465
VAANLLGQNAPAIAA	PPE FAMILY PROTEIN CAE55504	-0,162587448
SRSVEFAEQIRRDTD	MYCOBACTERIUM BOVIS MYCOCEROSIC ACID SYNTHASE GENE; M95808	-0,162749234
AASAAIANRLLRRTA	PUTATIVE CYCLOPROPANE-FATTY-ACYL-PHOSPHOLIPID SYNTHASE UFAA1 NP_854118	-0,16276827
AEDDCPPPGYDITAL	MYCOBACTERIUM BOVIS MYCOCEROSIC ACID SYNTHASE GENE; M95808	-0,162946063
VGETVAQLARTPVIV	HYPOTHETICAL PROTEIN RV2623 NP_217139	-0,16335628
PWEGHNYEDAIILSN	RNA POLYMERASE BETA-SUBUNIT AAA21416	-0,163373724
EITGSTLVDINETLN	YP_177963; PPE FAMILY PROTEIN	-0,163666766
ALVTRYMHVVVASYN	POSSIBLE GLYCOSYL TRANSFERASE CAB05419	-0,164140148
GAGTVYVTDFNNRVV	TRANSMEMBRANE SERINE/THREONINE-PROTEIN KINASE D PKND NP_215446	-0,164182955
WVPSPTAATLHALHY	MTB81 (publication)	-0,164380349
AELPVTVIAATAGRS	POSSIBLE GLYCOSYL TRANSFERASE CAB05415	-0,164764006
VTVTGQGNSLKVGNA	CAA98373.1; MAJOR SECRETED IMMUNOGENIC PROTEIN	-0,165195577
HDMGLVIGICAPILG	MYCOBACTERIUM BOVIS ACYL-COA SYNTHASE GENE; U75685	-0,165375005
TDGAEKGPTYNKVRG	MTB81 (publication)	-0,165450526
LIQAGAGEGSAITDA	SECRETED L-ALANINE DEHYDROGENASE ALD CAA15575	-0,165535247
ISFLDQASQRGLGEA	PERIPLASMIC PHOSPHATE-BINDING LIPOPROTEIN PSTS1 YP_177770	-0,165855897
DKLRQLDAEFCGRIH	SECRETED L-ALANINE DEHYDROGENASE ALD CAA15575	-0,165906086
TTTAGGLASPYVVTG	MYCOBACTERIUM BOVIS MYCOCEROSIC ACID SYNTHASE GENE; M95808	-0,166064423
ATISGVGNDLVLDTD	PERIPLASMIC PHOSPHATE-BINDING LIPOPROTEIN PSTS3 YP_177768	-0,166205292
PAVGQRGRALIATGE	CONSERVED HYPOTHETICAL PROTEIN CAB08634	-0,166650523
AAHHLVEGVARVLTK	POSSIBLE HEMOLYSIN-LIKE PROTEIN CAA17201	-0,16682181
EAKAKLEAAGATVTV	CAB07109.1; PROBABLE 50S RIBOSOMAL PROTEIN L7/L12 RPLL	-0,167679504
FDELRIGLATAEDIR	RNA POLYMERASE BETA'-SUBUNIT AAA21417	-0,168201686
AGVPKGVVVTKVDDR	CAA17582.1; PROBABLE SERINE PROTEASE PEPD	-0,16850789
GSIPITTSGKVRRGA	MYCOBACTERIUM BOVIS ACYL-COA SYNTHASE GENE; U75685	-0,16851624
AGSKTQTVLPFTGLN	TRANSMEMBRANE SERINE/THREONINE-PROTEIN KINASE D PKND NP_215446	-0,168875206
DEEAATALDNDGEGT	MTB48 AAK31576	-0,169842232
HQGESSAAFQAAHAR	CONSERVED HYPOTHETICAL PROTEIN TB9.8 CAD93159	-0,169918513
TQYLSKQDPEGWGKS	PERIPLASMIC PHOSPHATE-BINDING LIPOPROTEIN PSTS1 YP_177770	-0,17014789
ALGPGGLSRERAGLE	RNA POLYMERASE BETA-SUBUNIT AAA21416	-0,170419999
GRGSGLAAQGLAEVC	CONSERVED HYPOTHETICAL PROTEIN CAB08634	-0,170522327
VGSVNVGFGNVGDFN	YP_177963; PPE FAMILY PROTEIN	-0,172040934
SRMYSGPGPESMLAA	PPE FAMILY PROTEIN CAE55334	-0,17213996
QVLVNEHLIGPPPAT	CONSERVED HYPOTHETICAL PROTEIN CAB08634	-0,172233058
DVILESAITTIMDFE	MTB81 (publication)	-0,172312376
ATANAIAGILFLHSG	PPE FAMILY PROTEIN CAE55504	-0,172618421
PKARQTIEGLRAAGA	MYCOBACTERIUM BOVIS MYCOCEROSIC ACID SYNTHASE GENE; M95808	-0,17307117
LVLVFALVVALVYLQ	MCE-FAMILY PROTEIN MCE1AYP_177701	-0,173154198
NISGIEIKDLAGNVT	YP_177963; PPE FAMILY PROTEIN	-0,17329711
DGASNGAWGKGASET	PERIPLASMIC PHOSPHATE-BINDING LIPOPROTEIN PSTS2 YP_177769	-0,173513482
FTGLNSLTYDVSVAQ	PPE FAMILY PROTEIN CAE55504	-0,173632312
SPSMGRDIKVQFQSG	CAB10044.1; SECRETED ANTIGEN 85-B FBPB (85B)	-0,173686107
QIRQEIENSDSDYDR	60 kDa chaperonin 2 P0A521	-0,173714186
EGVAEAERAPIDAGT	CAB06237.1; CONSERVED HYPOTHETICAL PROTEIN	-0,174089635
NGPLINSRTGSIYIV	MTB81 (publication)	-0,174329577
TLPQRVAVRVSQLHA	CONSERVED HYPOTHETICAL PROTEIN CAB08634	-0,174382013
AGVGAVAMSLTVGAG	LOW MOLECULAR WEIGHT T-CELL ANTIGEN TB8.4 NP_215690	-0,174667565
GTPPFDLPTVVIDRI	YP_177963; PPE FAMILY PROTEIN	-0,175322473

Peptides	Protein	Average Index
VGLANMGVGNIGFAN	YP_177963; PPE FAMILY PROTEIN	-0,175552847
QAVAVAGQARAAVAA	YP_177963; PPE FAMILY PROTEIN	-0,176007652
FIINGTERVVVSQLV	RNA POLYMERASE BETA-SUBUNIT AAA21416	-0,176379147
AQANAMTRFVNVFEQ	PERIPLASMIC PHOSPHATE-BINDING LIPOPROTEIN PSTS3 YP_177768	-0,176452313
VNREPVDSAVLANGD	CAB01474.1; CONSERVED HYPOTHETICAL PROTEIN CFP17	-0,176719411
VTALVLLFVGGALYS	POSSIBLE HEMOLYSIN-LIKE PROTEIN CAA17201	-0,177355028
VVTKDETTIVEGAGD	60 kDa chaperonin 2 P0A521	-0,17778984
PNDQAGLEKLGSQLR	MYCOBACTERIUM BOVIS MYCOCEROSIC ACID SYNTHASE GENE; M95808	-0,178096027
TLIVGELGEATVVTG	CONSERVED HYPOTHETICAL PROTEIN CAB08634	-0,17816478
DVPRITILDAHFTGA	YP_177963; PPE FAMILY PROTEIN	-0,178168608
GLGNAGSTNVGLANM	YP_177963; PPE FAMILY PROTEIN	-0,178247557
TFEQMLLLDEQTPID	MYCOBACTERIUM BOVIS MYCOCEROSIC ACID SYNTHASE GENE; M95808	-0,178678408
GIGLTGDNQTGFGGL	YP_177963; PPE FAMILY PROTEIN	-0,179362769
KRGPNAGSRFLLDQA	CAB01474.1; CONSERVED HYPOTHETICAL PROTEIN CFP17	-0,179376465
EKKWGAPTITNDGVS	60 kDa chaperonin 2 P0A521	-0,180000181
APTNAAFSKLPASTI	CAA98373.1; MAJOR SECRETED IMMUNOGENIC PROTEIN	-0,180242336
AALDAAHANGVTHRD	TRANSMEMBRANE SERINE/THREONINE-PROTEIN KINASE D PKND NP_215446	-0,180454063
DSVPLSSGSFGDATG	MTB81 (publication)	-0,181167505
EAAEEQSEFDVILEA	CAB07109.1; PROBABLE 50S RIBOSOMAL PROTEIN L7/L12 RPLL	-0,181259416
NGARVQRVVGSAPAA	CAB09453.1; PROBABLE SERINE PROTEASE PEPA	-0,181431772
CEMALAAAAEVFGEA	MYCOBACTERIUM BOVIS MYCOCEROSIC ACID SYNTHASE GENE; M95808	-0,181748304
SGIGGLNSGTGNIGL	YP_177963; PPE FAMILY PROTEIN	-0,181944102
QPARQLVQRSEEAQL	HYPOTHETICAL PROTEIN RV2623 NP_217139	-0,182018663
LHGPAMTFDTACSSG	MYCOBACTERIUM BOVIS MYCOCEROSIC ACID SYNTHASE GENE; M95808	-0,182697404
GRVVKLATGSTGTTV	TRANSMEMBRANE SERINE/THREONINE-PROTEIN KINASE D PKND NP_215446	-0,183194815
VPGDRRTEKLVAIIE	MYCOBACTERIUM BOVIS ACYL-COA SYNTHASE GENE; U75685	-0,183312397
GNSSLGIIVGIDDSP	HYPOTHETICAL PROTEIN RV2623 NP_217139	-0,183336069
ASDNFQLSQGGQGFA	CAB09453.1; PROBABLE SERINE PROTEASE PEPA	-0,183746451
QIAAALDAAHANGVT	TRANSMEMBRANE SERINE/THREONINE-PROTEIN KINASE D PKND NP_215446	-0,183981199
SYPIVLATYEIVCSK	PERIPLASMIC PHOSPHATE-BINDING LIPOPROTEIN PSTS2 YP_177769	-0,184063983
MSQDPVAVAASNNPE	CAA98373.1; MAJOR SECRETED IMMUNOGENIC PROTEIN	-0,184308118
IVLGEADAAICGGVE	3-OXOACYL-[ACYL-CARRIER PROTEIN] SYNTHASE 2 KASB CAA94642	-0,184557393
GVGCSKVPDIHDVAL	MTB81 (publication)	-0,184646413
LGGLPVGQMGARAGG	CAE55371.1; PPE FAMILY PROTEIN	-0,185189268
SGLGAALGIGNIGLG	YP_177963; PPE FAMILY PROTEIN	-0,185317316
GAGNVGQQLSGVLAA	YP_177963; PPE FAMILY PROTEIN	-0,185699577
HQGQGGAKSKGSQQE	MTB48 AAK31576	-0,186306672
EDKILVQANEAETTT	CAB01005.1; 10 KDA CHAPERONIN GROES	-0,187082519
VIDVRSVTTEINTLF	MCE-FAMILY PROTEIN MCE1AYP_177701	-0,18717068
RNHSPDDIEETIQEI	MYCOBACTERIUM BOVIS ACYL-COA SYNTHASE GENE; U75685	-0,187767166
DLALMSVTQPDRVRE	MYCOBACTERIUM BOVIS MYCOCEROSIC ACID SYNTHASE GENE; M95808	-0,188954778
SQVRLDTAVQGIEGT	CONSERVED HYPOTHETICAL PROTEIN TB18.5CAD93033	-0,188976824
NLGGGNLGGQNLGLG	YP_177963; PPE FAMILY PROTEIN	-0,189049876
AVAIVRQIAAALDAA	TRANSMEMBRANE SERINE/THREONINE-PROTEIN KINASE D PKND NP_215446	-0,189141473
SVASGRIAHTLGLHG	MYCOBACTERIUM BOVIS MYCOCEROSIC ACID SYNTHASE GENE; M95808	-0,189362527
ERLRWLVAAEGPFAS	CONSERVED HYPOTHETICAL PROTEIN CAB08634	-0,189386175
GNSGTGSTGLFNAGG	YP_177963; PPE FAMILY PROTEIN	-0,189806468
GTLVLTACGGGTNSS	PERIPLASMIC PHOSPHATE-BINDING LIPOPROTEIN PSTS2 YP_177769	-0,190279654
LNRPVSTTGEAGNQN	CAA17582.1; PROBABLE SERINE PROTEASE PEPD	-0,190605308
NVGLGNVGGGNVGFG	YP_177963; PPE FAMILY PROTEIN	-0,19087812
TSGSVHCGGKKELHS	PERIPLASMIC PHOSPHATE-BINDING LIPOPROTEIN PSTS2 YP_177769	-0,190900029
ALGDNWIVGASNSTG	YP_177963; PPE FAMILY PROTEIN	-0,191137569
NVGSGISGLYNTAIV	YP_177963; PPE FAMILY PROTEIN	-0,191363902
GRAPSASELAAELGM	ALTERNATE RNA POLYMERASE SIGMA FACTOR SIGF CAB07069	-0,191716453
PASSPVTLAETGSTL	PERIPLASMIC PHOSPHATE-BINDING LIPOPROTEIN PSTS1 YP_177770	-0,191960965
AVAGQARAAVAAFEA	YP_177963; PPE FAMILY PROTEIN	-0,192398528
TVIGARDDLMVNNAG	CAA98350.1; CELL SURFACE LIPOPROTEIN MPT83	-0,192963455
FDFLDSSVTTARTIN	MCE-FAMILY PROTEIN MCE1AYP_177701	-0,19325808
RSIGVYAVNYPASDD	PROBABLE CUTINASE PRECURSOR CFP21 NP_216500	-0,193421756
NIGDANFGFGNSGLA	YP_177963; PPE FAMILY PROTEIN	-0,193984792
NDDNVTGGGATTGQA	PERIPLASMIC PHOSPHATE-BINDING LIPOPROTEIN PSTS3 YP_177768	-0,194112633

Peptides	Protein	Average Index
LVDAADPEVVFVSGE	CONSERVED HYPOTHETICAL PROTEIN CAB08634	-0,194210922
PGPDAGTSTAPNAAP	POSSIBLE GLYCOSYL TRANSFERASE CAB05418	-0,194432593
AAESPTSVLLINHGL	MTB81 (publication)	-0,194488549
AGWNGYGDFQHTTEE	CONSERVED HYPOTHETICAL PROTEIN CAB08634	-0,194797179
GGNRPAVYAPKSALG	3-OXOACYL-[ACYL-CARRIER PROTEIN] SYNTHASE 2 KASB CAA94642	-0,194933864
GLDSLGMLEMRTHVE	MYCOBACTERIUM BOVIS MYCOCEROSIC ACID SYNTHASE GENE; M95808	-0,195298368
AVGKQLNMAQIITSA	PERIPLASMIC PHOSPHATE-BINDING LIPOPROTEIN PSTS2 YP_177769	-0,195492888
TGQHTGLRIVDAASL	PUTATIVE CYCLOPROPANE-FATTY-ACYL-PHOSPHOLIPID SYNTHASE UFAA1 NP_854118	-0,195668808
SEFAYGSFVRTVSLP	CAA17245.1; HEAT SHOCK PROTEIN HSPX	-0,195697659
QVGPQVVNINTKLGY	CAB09453.1; PROBABLE SERINE PROTEASE PEPA	-0,195969943
CSKVPDIHDVALMED	MTB81 (publication)	-0,196403741
RAGHAITRAIQLAGL	3-OXOACYL-[ACYL-CARRIER PROTEIN] SYNTHASE 2 KASB CAA94642	-0,19704933
LPFNGLYQPQGLAVD	TRANSMEMBRANE SERINE/THREONINE-PROTEIN KINASE D PKND NP_215446	-0,197055451
LNLGASVTVGPILIN	YP_177963; PPE FAMILY PROTEIN	-0,197522397
SDTRDVLRAMKKMID	CAB06237.1; CONSERVED HYPOTHETICAL PROTEIN	-0,197886951
NPGNSGGALVNMNAQ	CAA17582.1; PROBABLE SERINE PROTEASE PEPD	-0,197889885
VHKPVTAGWNGYGDF	CONSERVED HYPOTHETICAL PROTEIN CAB08634	-0,198164543
ANAFVADYLPGEAAA	POSSIBLE GLYCOSYL TRANSFERASE CAB05415	-0,198587808
DLIGRGCAQYAAQNP	CAA98350.1; CELL SURFACE LIPOPROTEIN MPT83	-0,19868501
KDETTIVEGAGDTDA	60 kDa chaperonin 2 P0A521	-0,199091359
TFEVTAAAPVAVAAA	CAB07109.1; PROBABLE 50S RIBOSOMAL PROTEIN L7/L12 RPLL	-0,199326778
GNIGLGNAGSTNVGL	YP_177963; PPE FAMILY PROTEIN	-0,199616561
IPETDGAEKGPTYNK	MTB81 (publication)	-0,200256229
RSNPALAKGLSTHEG	SECRETED L-ALANINE DEHYDROGENASE ALD CAA15575	-0,200688932
QFEAAGLTYEHRLID	CAA17111.1; PROBABLE ISOCITRATE DEHYDROGENASE	-0,200783355
QRRKIDRLLDVAGVQ	PUTATIVE CYCLOPROPANE-FATTY-ACYL-PHOSPHOLIPID SYNTHASE UFAA1 NP_854118	-0,201313996
LPAHVTSEAIAASYD	CAA17343.1; HEAT SHOCK PROTEIN HSP	-0,20139037
GFEDIGIKTGNERAK	PROBABLE ISOCITRATE DEHYDROGENASE CAA16247	-0,201585673
VIGAGKPLLIIAEDV	60 kDa chaperonin 2 P0A521	-0,202139991
RIYNAGDLFVGGNII	YP_177963; PPE FAMILY PROTEIN	-0,202408466
QQGGQQHATLVSDKK	CAB06237.1; CONSERVED HYPOTHETICAL PROTEIN	-0,202433603
RTNSEILSGIGISLL	MCE-FAMILY PROTEIN MCE1AYP_177701	-0,20252313
GDGGVSGFGNFGAGS	YP_177963; PPE FAMILY PROTEIN	-0,202531194
LTGAPPYRADSVERL	TRANSMEMBRANE SERINE/THREONINE-PROTEIN KINASE D PKND NP_215446	-0,20314981
GPIIIPDTTILPPIH	YP_177963; PPE FAMILY PROTEIN	-0,203178905
SAAAKNTPGSITYNE	PERIPLASMIC PHOSPHATE-BINDING LIPOPROTEIN PSTS3 YP_177768	-0,203607282
GSVAEAMARVPAARP	TWO COMPONENT TRANSCRIPTIONAL REGULATORY PROTEIN DEVR NP_217649	-0,203613672
VSGLLNQASSPMVGT	YP_177963; PPE FAMILY PROTEIN	-0,203946653
FAAIGHIGPISIPNI	YP_177963; PPE FAMILY PROTEIN	-0,204010804
GNRIFAVVRGTATNQ	MYCOBACTERIUM BOVIS MYCOCEROSIC ACID SYNTHASE GENE; M95808	-0,204070681
AFIGEHEPSDLVYGD	POSSIBLE GLYCOSYL TRANSFERASE CAB05419	-0,204591158
AAVRSKAPGATVALT	CAA17582.1; PROBABLE SERINE PROTEASE PEPD	-0,204896626
APVLVGVDGSSASEL	HYPOTHETICAL PROTEIN RV2623 NP_217139	-0,20580806
DLIQVARVGLVNAAV	ALTERNATE RNA POLYMERASE SIGMA FACTOR SIGF CAB07069	-0,205969151
MLQDMAILTGGQVIS	60 kDa chaperonin 2 P0A521	-0,206320055
DIEKDQTSDEVTVET	CAB01474.1; CONSERVED HYPOTHETICAL PROTEIN CFP17	-0,206345846
SGAEAIARAWQQIVL	3-OXOACYL-[ACYL-CARRIER PROTEIN] SYNTHASE 2 KASB CAA94642	-0,206559738
STAGSLQGQWRGAAG	CAA17966.1; 10 KDA CULTURE FILTRATE ANTIGEN ESXB	-0,206759292
IVAGGGVTLLQAAPT	60 kDa chaperonin 2 P0A521	-0,206853614
MKFARSGAAVSLLAA	PERIPLASMIC PHOSPHATE-BINDING LIPOPROTEIN PSTS2 YP_177769	-0,206989369
DGVGYPVHKPVTAGW	CONSERVED HYPOTHETICAL PROTEIN CAB08634	-0,207012946
EVTRDGESAAKFILD	MCE-FAMILY PROTEIN MCE1AYP_177701	-0,207334144
GTPALVSGLGNVGDH	YP_177963; PPE FAMILY PROTEIN	-0,207358249
ADIERYPEWNEGVKG	CONSERVED HYPOTHETICAL PROTEIN TB18.5CAD93033	-0,207497725
IHSLLDEGKQSLTKL	CAE55648	-0,208000981
AGSGIANVGTLHSGV	YP_177963; PPE FAMILY PROTEIN	-0,208247175
AAGTYVAADAAAAST	CONSERVED HYPOTHETICAL PROTEIN TB9.8 CAD93159	-0,208530638
AAKAGLMRHTIGQAE	CONSERVED HYPOTHETICAL PROTEIN TB9.8 CAD93159	-0,209047552
DRAGVKDVILESAIT	MTB81 (publication)	-0,209101714
GGAAANAGVPKGVVV	CAA17582.1; PROBABLE SERINE PROTEASE PEPD	-0,209154154

Peptides	Protein	Average Index
PGQPLPTLPVPVLRD	CAA16030.1; PROBABLE MOLYBDOPTERIN-GUANINE DINUCLEOTIDE BIOSYNTHESIS PROTEIN	-0,210373721
TVLPFDGLNYPEGLA	TRANSMEMBRANE SERINE/THREONINE-PROTEIN KINASE D PKND NP_215446	-0,211207648
PKTYCEELKGTDTGQ	CAA98382.1; IMMUNOGENIC PROTEIN MPT64	-0,211391828
SLLSPLALATNGAAI	MCE-FAMILY PROTEIN MCE1AYP_177701	-0,211690617
YLQFRGEFTPKTQLT	MCE-FAMILY PROTEIN MCE1AYP_177701	-0,211737674
RVAQQLLSGSEEDET	MYCOBACTERIUM BOVIS MYCOCEROSIC ACID SYNTHASE GENE; M95808	-0,211848917
LAAGALVLSACGNDD	PERIPLASMIC PHOSPHATE-BINDING LIPOPROTEIN PSTS3 YP_177768	-0,212072533
NTFNLTLQGDVKRFR	MTB48 AAK31576	-0,212171744
RDRNYTAPGGGQFTL	MTB81 (publication)	-0,212389039
LAGCSSTKPVSQDTS	CAA98350.1; CELL SURFACE LIPOPROTEIN MPT83	-0,212822113
ASDPGLTQTGTAVGT	TRANSMEMBRANE SERINE/THREONINE-PROTEIN KINASE D PKND NP_215446	-0,213143528
VNEHLIGPPPATVIR	CONSERVED HYPOTHETICAL PROTEIN CAB08634	-0,213340979
LGEPVDRVARADEAL	CONSERVED HYPOTHETICAL PROTEIN CAB08634	-0,213418173
RVFSREDEDELPAGV	RNA POLYMERASE BETA-SUBUNIT AAA21416	-0,21345037
LAGNVTLQLGNLNID	YP_177963; PPE FAMILY PROTEIN	-0,213598911
NVGQQLSGVLAAGTA	YP_177963; PPE FAMILY PROTEIN	-0,214836941
PYVVTGLNNSVASGR	MYCOBACTERIUM BOVIS MYCOCEROSIC ACID SYNTHASE GENE; M95808	-0,214959612
LKKRGDSDQDAMARL	MYCOBACTERIUM BOVIS ACYL-COA SYNTHASE GENE; U75685	-0,215240245
VTDFNNRVVTLAAGS	TRANSMEMBRANE SERINE/THREONINE-PROTEIN KINASE D PKND NP_215446	-0,215242237
DAASIMAIVADIERY	CONSERVED HYPOTHETICAL PROTEIN TB18.5CAD93033	-0,215554636
AAHGTVTRHYRQYQA	CAA17111.1; PROBABLE ISOCITRATE DEHYDROGENASE	-0,215827968
DPNTATAGELARDSI	HYPOTHETICAL PROTEIN RV2626C NP_217142	-0,21660966
TRQTLQGASVTVTGQ	CAA98373.1; MAJOR SECRETED IMMUNOGENIC PROTEIN	-0,216884125
QGTGPAGPAAAAAAT	PPE FAMILY PROTEIN CAE55334	-0,217063785
PLRLVYSDGTATGAA	PUTATIVE CYCLOPROPANE-FATTY-ACYL-PHOSPHOLIPID SYNTHASE UFAA1 NP_854118	-0,217316646
AVLTAAPLLLAAAGC	PERIPLASMIC PHOSPHATE-BINDING LIPOPROTEIN PSTS1 YP_177770	-0,217460051
IILSAEGLILTNNHV	CAA17582.1; PROBABLE SERINE PROTEASE PEPD	-0,218321114
EIPPDRWDADDYYDP	MYCOBACTERIUM BOVIS MYCOCEROSIC ACID SYNTHASE GENE; M95808	-0,218792497
AAVSEIRMTSGSSSG	YP_177963; PPE FAMILY PROTEIN	-0,219124153
GTRTGNVTLAEGPPA	CAB09453.1; PROBABLE SERINE PROTEASE PEPA	-0,220203785
RRALRQIGVLERPVG	CAA16102.1; ESAT-6 LIKE PROTEIN ESXQ	-0,22041543
PRYASRMPVLSKTVE	CONSERVED HYPOTHETICAL PROTEIN TB18.5CAD93033	-0,220964846
LADSRQSKTAASPSP	RNA POLYMERASE BETA-SUBUNIT AAA21416	-0,222218095
PFTGIDFRLSPSGVA	TRANSMEMBRANE SERINE/THREONINE-PROTEIN KINASE D PKND NP_215446	-0,222355579
TNGAAIGIGLVAGLI	MCE-FAMILY PROTEIN MCE1AYP_177701	-0,222681946
QNGVRAMSSLGSSLG	CAE55371.1; PPE FAMILY PROTEIN	-0,222785585
AISVPGDRRTEKLVA	MYCOBACTERIUM BOVIS ACYL-COA SYNTHASE GENE; U75685	-0,222879386
SALLVVKRGPNAGSR	CAB01474.1; CONSERVED HYPOTHETICAL PROTEIN CFP17	-0,223060848
DDGQEQRAQGACTIT	MYCOBACTERIUM BOVIS MYCOCEROSIC ACID SYNTHASE GENE; M95808	-0,223764426
EQQRLARQRVAAAGF	PUTATIVE CYCLOPROPANE-FATTY-ACYL-PHOSPHOLIPID SYNTHASE UFAA1 NP_854118	-0,224318696
ENSDSDYDREKLQER	60 kDa chaperonin 2 P0A521	-0,224395891
WGQLLLLGEGIPDPG	CAB06237.1; CONSERVED HYPOTHETICAL PROTEIN	-0,224420974
TAGPQLVVPVLNARF	MTB81 (publication)	-0,224883915
YGLAEATVYVATSKP	MYCOBACTERIUM BOVIS ACYL-COA SYNTHASE GENE; U75685	-0,225722775
SGGGSDDDARAITDT	ALTERNATE RNA POLYMERASE SIGMA FACTOR SIGF CAB07069	-0,225943357
SRACTDALLDSGTTS	SECRETED L-ALANINE DEHYDROGENASE ALD CAA15575	-0,22599492
AGGFNTGVANAGSYN	YP_177963; PPE FAMILY PROTEIN	-0,22631958
GNLNLGGGNLGGQNL	YP_177963; PPE FAMILY PROTEIN	-0,226828428
QAAQAAGQARLAASA	PPE FAMILY PROTEIN CAE55504	-0,22715873
QVVLGWKVSDLKSST	CAB06500.1; IMMUNOGENIC PROTEIN MPT63	-0,227519707
GDAATACEASLDQQR	MTB48 AAK31576	-0,227552878
GQILFTFLHLAASRA	SECRETED L-ALANINE DEHYDROGENASE ALD CAA15575	-0,227605481
GFAVPDIPIPAIHID	YP_177963; PPE FAMILY PROTEIN	-0,227807345
RRTAVLDPDHDGMRV	MYCOBACTERIUM BOVIS MYCOCEROSIC ACID SYNTHASE GENE; M95808	-0,228240731
DLDVVAGEPRPGNYR	3-OXOACYL-[ACYL-CARRIER PROTEIN] SYNTHASE 2 KASB CAA94642	-0,22851024
LGNIGNLNLGAANIG	YP_177963; PPE FAMILY PROTEIN	-0,228925231
NVGLGNIGNLNLGAA	YP_177963; PPE FAMILY PROTEIN	-0,229187085
DGRTAPFTVVGADPT	CAA17582.1; PROBABLE SERINE PROTEASE PEPD	-0,229878601

Peptides	Protein	Average Index
GHGNVGFGNSGLGAA	YP_177963; PPE FAMILY PROTEIN	-0,230562794
QRPLYRRDGSYIITG	MYCOBACTERIUM BOVIS MYCOCEROSIC ACID SYNTHASE GENE; M95808	-0,230565304
PGFTIPTGPIHIGLP	YP_177963; PPE FAMILY PROTEIN	-0,230891642
GVLDPITIFPGGFTI	YP_177963; PPE FAMILY PROTEIN	-0,231123662
LRWQQDHGRHLIDDA	HYPOTHETICAL PROTEIN RV2623 NP_217139	-0,231129164
PSSGFFNTGAGGGSG	YP_177963; PPE FAMILY PROTEIN	-0,231238235
GFSYSIEITGSTLVD	YP_177963; PPE FAMILY PROTEIN	-0,231464918
VAAVALFGEPSSGFS	PROBABLE CUTINASE PRECURSOR CFP21 NP_216500	-0,231808427
RPVVAAIKEFFGTSQ	RNA POLYMERASE BETA-SUBUNIT AAA21416	-0,231848771
ARSQPNPKARQTIEG	MYCOBACTERIUM BOVIS MYCOCEROSIC ACID SYNTHASE GENE; M95808	-0,23195329
VDSDRTVYVADRGND	TRANSMEMBRANE SERINE/THREONINE-PROTEIN KINASE D PKND NP_215446	-0,232270826
PENLPRVNARGGPGG	MCE-FAMILY PROTEIN MCE1AYP_177701	-0,233473844
LNLDGPTTAKIFNGT	PERIPLASMIC PHOSPHATE-BINDING LIPOPROTEIN PSTS2 YP_177769	-0,233690477
ATAGRSDLKTVPANA	POSSIBLE GLYCOSYL TRANSFERASE CAB05415	-0,23375119
TNDAIVDTDGSEVFE	MTB81 (publication)	-0,23431226
LPVTVIAATAGRNHL	POSSIBLE GLYCOSYL TRANSFERASE CAB05418	-0,234951842
VTSALSSSHGLSVAD	MYCOBACTERIUM BOVIS ACYL-COA SYNTHASE GENE; U75685	-0,234977354
GAGAVADAQATLAQL	PPE FAMILY PROTEIN CAE55334	-0,235491845
VVFARGTHQASGLGD	PROBABLE CUTINASE PRECURSOR CFP21 NP_216500	-0,235654828
ADDSLYEADTLARVA	POSSIBLE GLYCOSYL TRANSFERASE CAB05419	-0,236361949
SNGAWGKGAGKSFQG	PERIPLASMIC PHOSPHATE-BINDING LIPOPROTEIN PSTS3 YP_177768	-0,236533877
AAGVQPETVGVVEAH	MYCOBACTERIUM BOVIS MYCOCEROSIC ACID SYNTHASE GENE; M95808	-0,236587559
RTLDDPFVEEFDLPV	3-OXOACYL-[ACYL-CARRIER PROTEIN] SYNTHASE 2 KASB CAA94642	-0,236588495
NYTAPGGGQFTLPGR	MTB81 (publication)	-0,237043113
PAFHERYPNVTITAQ	PERIPLASMIC PHOSPHATE-BINDING LIPOPROTEIN PSTS1 YP_177770	-0,237071095
LNALADLPVTVIAAT	POSSIBLE GLYCOSYL TRANSFERASE CAB05418	-0,237167711
AIAIAFLAGCSSTKP	CAA98350.1; CELL SURFACE LIPOPROTEIN MPT83	-0,237382373
GHGNVGFGNSGLGAL	YP_177963; PPE FAMILY PROTEIN	-0,238571511
EIMRSTLEKDNTVGT	RNA POLYMERASE BETA-SUBUNIT AAA21416	-0,238942915
KLREPLEVPGLLDVQ	RNA POLYMERASE BETA-SUBUNIT AAA21416	-0,239390142
PPVQGTGPAGPAAAA	PPE FAMILY PROTEIN CAE55334	-0,240503424
SGFFNSGDGGVSGFG	YP_177963; PPE FAMILY PROTEIN	-0,24121047
HERYPNVTITAQGTG	PERIPLASMIC PHOSPHATE-BINDING LIPOPROTEIN PSTS1 YP_177770	-0,241414658
RRLRTVGELIQNQIR	RNA POLYMERASE BETA-SUBUNIT AAA21416	-0,241829536
KIDRLLDVAGVQQGS	PUTATIVE CYCLOPROPANE-FATTY-ACYL-PHOSPHOLIPID SYNTHASE UFAA1 NP_854118	-0,242325506
GFGNDTTAPSSGFFN	YP_177963; PPE FAMILY PROTEIN	-0,242647743
SQGMGPSLIGLAMGD	CAB10044.1; SECRETED ANTIGEN 85-B FBPB (85B)	-0,243782329
RAAANLGINLSRNES	RNA POLYMERASE BETA-SUBUNIT AAA21416	-0,244340399
TGTQVGDLAEGRAIN	3-OXOACYL-[ACYL-CARRIER PROTEIN] SYNTHASE 2 KASB CAA94642	-0,24435533
QLNAMKGDLQSSLGA	CAB10044.1; SECRETED ANTIGEN 85-B FBPB (85B)	-0,245178137
QQDHGRHLIDDALKV	HYPOTHETICAL PROTEIN RV2623 NP_217139	-0,246621713
DFAGSDVPLNPSTGQ	PERIPLASMIC PHOSPHATE-BINDING LIPOPROTEIN PSTS2 YP_177769	-0,246697423
LVVALVYLQFRGEFT	MCE-FAMILY PROTEIN MCE1AYP_177701	-0,246862535
DGRTETLTMPSEDAQ	MYCOBACTERIUM BOVIS MYCOCEROSIC ACID SYNTHASE GENE; M95808	-0,246882344
WLLFLGADDSLYEAD	POSSIBLE GLYCOSYL TRANSFERASE CAB05419	-0,247747788
EFLKRLPMSTRLGIR	POSSIBLE GLYCOSYL TRANSFERASE CAB05419	-0,247898151
DMAGRDLGNILTILS	MYCOBACTERIUM BOVIS ACYL-COA SYNTHASE GENE; U75685	-0,248617057
KARTTVARDADMLSE	CONSERVED HYPOTHETICAL PROTEIN CAB08634	-0,249056071
FPDPTISVQIQVAIP	YP_177963; PPE FAMILY PROTEIN	-0,250168473
DSVGQTIAGATISGV	PERIPLASMIC PHOSPHATE-BINDING LIPOPROTEIN PSTS3 YP_177768	-0,250424609
LRTTLIDVDEHTDVE	MYCOBACTERIUM BOVIS MYCOCEROSIC ACID SYNTHASE GENE; M95808	-0,250731617
LTSGLLGIASTLNPQ	PPE FAMILY PROTEIN CAE55334	-0,250923494
DVDTLIVGELGEATV	CONSERVED HYPOTHETICAL PROTEIN CAB08634	-0,25223226
DPRHAGPATAADAGD	CAA16102.1; ESAT-6 LIKE PROTEIN ESXQ	-0,252246713
AEKGPTYNKVRGDKV	MTB81 (publication)	-0,252633791
HADPCSDIAVVFARG	PROBABLE CUTINASE PRECURSOR CFP21 NP_216500	-0,253804567
LSLTIPGFTIPGGTL	YP_177963; PPE FAMILY PROTEIN	-0,253868535
PTLNLVNLDPEIDLD	3-OXOACYL-[ACYL-CARRIER PROTEIN] SYNTHASE 2 KASB CAA94642	-0,254032023
TRHYRQYQAGKPTST	CAA17111.1; PROBABLE ISOCITRATE DEHYDROGENASE	-0,255182477
DANFGLGNAGLAAGL	YP_177963; PPE FAMILY PROTEIN	-0,25572256
APINLNPTTLHFPGF	YP_177963; PPE FAMILY PROTEIN	-0,257487933
YEAIVKGENIPEPGI	RNA POLYMERASE BETA-SUBUNIT AAA21416	-0,25749877

Peptides	Protein	Average Index
NVELDPGQPVSRLVI	CAA17343.1; HEAT SHOCK PROTEIN HSP	-0,257789324
GVSIAKEIELEDPYE	60 kDa chaperonin 2 P0A521	-0,257978052
TSDNFQKYLDGASNG	PERIPLASMIC PHOSPHATE-BINDING LIPOPROTEIN PSTS2 YP_177769	-0,258342494
NANTGVANSGNVDTG	YP_177963; PPE FAMILY PROTEIN	-0,25885151
FAHKLESVVIATVES	CAA17111.1; PROBABLE ISOCITRATE DEHYDROGENASE	-0,258962115
TQGILIHTGPSSATT	YP_177963; PPE FAMILY PROTEIN	-0,259104705
NNLGIGLTGDNQSGI	YP_177963; PPE FAMILY PROTEIN	-0,259920871
KVDQPGTVTLTFTPA	CAA17111.1; PROBABLE ISOCITRATE DEHYDROGENASE	-0,260013399
LLKELQSLCLNVEVL	RNA POLYMERASE BETA-SUBUNIT AAA21416	-0,260693181
NSGVDISGMFNVSTL	YP_177963; PPE FAMILY PROTEIN	-0,261139719
PIPLFPAVGADVTGD	YP_177963; PPE FAMILY PROTEIN	-0,26358494
FVSFAVPTIMGEVRR	ALTERNATE RNA POLYMERASE SIGMA FACTOR SIGF CAB07069	-0,264049927
GTALTQSPIINLGLA	YP_177963; PPE FAMILY PROTEIN	-0,264340008
DLLGADPELDVVGEA	TWO COMPONENT TRANSCRIPTIONAL REGULATORY PROTEIN DEVR NP_217649	-0,264402577
TAARAVLDMAASVGA	YP_177963; PPE FAMILY PROTEIN	-0,264416478
VSNSLVAHMKPGAVL	SECRETED L-ALANINE DEHYDROGENASE ALD CAA15575	-0,26544382
SSGAGGTSGSVHCGG	PERIPLASMIC PHOSPHATE-BINDING LIPOPROTEIN PSTS2 YP_177769	-0,265560598
YDEEARRGLERGLNA	60 kDa chaperonin 2 P0A521	-0,265780813
TRDIPNISDEVLADL	RNA POLYMERASE BETA-SUBUNIT AAA21416	-0,266242123
GDVISVTWQTKSGGT	CAB09453.1; PROBABLE SERINE PROTEASE PEPA	-0,266281636
LVEHMVGILGQRCAP	CAA16030.1; PROBABLE MOLYBDOPTERIN-GUANINE DINUCLEOTIDE BIOSYNTHESIS PROTEIN	-0,266342897
TTSNFIWGQLLLLGE	CAB06237.1; CONSERVED HYPOTHETICAL PROTEIN	-0,266418141
NSGLEPGVVAEKVRN	60 kDa chaperonin 2 P0A521	-0,266687415
VVVIREQPPPGNPPR	PPE FAMILY PROTEIN CAE55334	-0,26673411
PDVVVGDNRLSLSVS	POSSIBLE GLYCOSYL TRANSFERASE CAB05418	-0,266926179
KPPVVVIREQPPPGN	PPE FAMILY PROTEIN CAE55334	-0,266956548
NAGSSNYGLANLGVG	YP_177963; PPE FAMILY PROTEIN	-0,267041872
PIQILPTIPLNIHQT	YP_177963; PPE FAMILY PROTEIN	-0,267417053
LVLDVPRSGRRSVAV	MYCOBACTERIUM BOVIS MYCOCEROSIC ACID SYNTHASE GENE; M95808	-0,267658148
AEKVRNLPAGHGLNA	60 kDa chaperonin 2 P0A521	-0,268544715
DSSDCGTIRVGSFRG	CAA16102.1; ESAT-6 LIKE PROTEIN ESXQ	-0,269422644
LRNVAAGANPLGLKR	60 kDa chaperonin 2 P0A521	-0,269525338
IGPVSLPIFGFGGAP	YP_177963; PPE FAMILY PROTEIN	-0,27106608
GDLLPGVGDLGKLPT	CAB06237.1; CONSERVED HYPOTHETICAL PROTEIN	-0,27135556
RGNDGTSAAAKNTPG	PERIPLASMIC PHOSPHATE-BINDING LIPOPROTEIN PSTS3 YP_177768	-0,273795209
FGNLGNHVSGVSIDG	YP_177963; PPE FAMILY PROTEIN	-0,274140655
VSRRAKVDVLIVHTT	CAB08889.1; IRON-REGULATED CONSERVED HYPOTHETICAL PROTEIN	-0,276911525
PTITNDGVSIAKEIE	60 kDa chaperonin 2 P0A521	-0,277579369
ESMKMEIPVLAEAAG	CAB08316.1; BIOTINYLATED PROTEIN TB7.3	-0,27865641
VLNRDRNYTAPGGGQ	MTB81 (publication)	-0,279548314
PYEALGGTRPGLIPG	CAA17582.1; PROBABLE SERINE PROTEASE PEPD	-0,281595756
KIIYFAAYVITSVDE	RNA POLYMERASE BETA'-SUBUNIT AAA21417	-0,282027922
CSSNKSTTGSGETTT	LIPOPROTEIN LPQH PRECURSOR P0A5J0	-0,282179042
LFPILELGTSAKMLS	PROBABLE ISOCITRATE DEHYDROGENASE CAA16247	-0,282225084
NWNIGIGITGNGQIG	PPE FAMILY PROTEIN CAE55504	-0,282641076
YSLRTNSEILSGIGI	MCE-FAMILY PROTEIN MCE1AYP_177701	-0,282702864
AGLAAGFSGRAQVGK	MTB81 (publication)	-0,284947393
FSIPQLGFTLSGATP	PPE FAMILY PROTEIN CAE55504	-0,285715336
ISSTKIDGVGYPVHK	CONSERVED HYPOTHETICAL PROTEIN CAB08634	-0,286602907
FNVSTLGSAPAVISG	YP_177963; PPE FAMILY PROTEIN	-0,287291072
RLLGSVSSGLLRHAH	HYPOTHETICAL PROTEIN RV2623 NP_217139	-0,289252291
TETLLKGAKEVETKE	60 kDa chaperonin 2 P0A521	-0,289714417
SEHRLVGIVTEADIA	HYPOTHETICAL PROTEIN RV2626C NP_217142	-0,290368727
HQDVPSPFGLFPDVD	PPE FAMILY PROTEIN CAE55504	-0,290891601
SLAAIAIAFLAGCSS	CAA98350.1; CELL SURFACE LIPOPROTEIN MPT83	-0,290976517
NAGGLGPTQGHPLSS	PPE FAMILY PROTEIN CAE55334	-0,292448831
FLRVLNRDRNYTAPG	MTB81 (publication)	-0,293127662
LKVGNADVVCGGVST	CAA98373.1; MAJOR SECRETED IMMUNOGENIC PROTEIN	-0,294521559
ISTGDVITAVDGAPI	CAB09453.1; PROBABLE SERINE PROTEASE PEPA	-0,296554342
LEPGVVAEKVRNLPA	60 kDa chaperonin 2 P0A521	-0,297100507
DFLSELDAPAQAGTE	CAB01474.1; CONSERVED HYPOTHETICAL PROTEIN CFP17	-0,297649723
FRLVFLTGPKQGEDD	CAB01474.1; CONSERVED HYPOTHETICAL PROTEIN CFP17	-0,298233321

Peptides	Protein	Average Index
VDISGMFNVSTLGSA	YP_177963; PPE FAMILY PROTEIN	-0,298572594
ADRGNNRVVKLAAGS	TRANSMEMBRANE SERINE/THREONINE-PROTEIN KINASE D PKND NP_215446	-0,298930406
GTTDRAGVKDVILES	MTB81 (publication)	-0,299591192
MSPVNKSFEIEVTCS	LIPOPROTEIN LPQH PRECURSOR P0A5J0	-0,29961902
AAVEAAEEQSEFDVI	CAB07109.1; PROBABLE 50S RIBOSOMAL PROTEIN L7/L12 RPLL	-0,300901232
SATYQSAIPPRGTQA	CAA98382.1; IMMUNOGENIC PROTEIN MPT64	-0,301166226
IAKLEPVIAAESGFS	MYCOBACTERIUM BOVIS MYCOCEROSIC ACID SYNTHASE GENE; M95808	-0,301512671
LTVGAGVASADPVDA	LOW MOLECULAR WEIGHT T-CELL ANTIGEN TB8.4 NP_215690	-0,302237221
IEEHEIDARDTKLGA	RNA POLYMERASE BETA-SUBUNIT AAA21416	-0,305059374
IAALNPGVNLPGTAV	PERIPLASMIC PHOSPHATE-BINDING LIPOPROTEIN PSTS1 YP_177770	-0,308466997
VAITPAGVAELTRRG	SECRETED L-ALANINE DEHYDROGENASE ALD CAA15575	-0,308799277
LELPGIDVDKDVNVE	CAA17343.1; HEAT SHOCK PROTEIN HSP	-0,309312105
LIQPDLRVLVELGYD	PPE FAMILY PROTEIN CAE55504	-0,309472203
EIGTGWGELCIRAAA	PUTATIVE CYCLOPROPANE-FATTY-ACYL-PHOSPHOLIPID SYNTHASE UFAA1 NP_854118	-0,310339388
LARVVGLVQEEQPSD	CAA17582.1; PROBABLE SERINE PROTEASE PEPD	-0,310485449
AAGTAAQAAVVRFQE	CAA17966.1; 10 KDA CULTURE FILTRATE ANTIGEN ESXB	-0,313914482
PVATAASNNPMLSTL	CAA98350.1; CELL SURFACE LIPOPROTEIN MPT83	-0,315898357
KQKQELDEISTNIRQ	CAA17966.1; 10 KDA CULTURE FILTRATE ANTIGEN ESXB	-0,324960335
VNIKPLEDKILVQAN	CAB01005.1; 10 KDA CHAPERONIN GROES	-0,32587626
LHVGEPITSSTLTEE	RNA POLYMERASE BETA-SUBUNIT AAA21416	-0,330583347
GLTVPPPVIAENRAE	CAE55371.1; PPE FAMILY PROTEIN	-0,333141888
LSELGEPVDRVARAD	CONSERVED HYPOTHETICAL PROTEIN CAB08634	-0,333407473
VLVAPVSPSTASSRT	PPE FAMILY PROTEIN CAE55489	-0,336240473
GRQSEEGSGIILSAE	CAA17582.1; PROBABLE SERINE PROTEASE PEPD	-0,336531638
GAGVSGLLNQASSPM	YP_177963; PPE FAMILY PROTEIN	-0,34771685
PALTAARAVLDMAAS	YP_177963; PPE FAMILY PROTEIN	-0,351552056
NVARELSRCGSTGDR	MYCOBACTERIUM BOVIS ACYL-COA SYNTHASE GENE; U75685	-0,353023131
AAAWDGLAEELGSAA	YP_177963; PPE FAMILY PROTEIN	-0,362995126
AQEAELQGLLSCTLP	RNA POLYMERASE BETA-SUBUNIT AAA21416	-0,366421509
TEEHAKARGANILAR	3-OXOACYL-[ACYL-CARRIER PROTEIN] SYNTHASE 2 KASB CAA94642	-0,367179535
IVSTPVFDGAQEAEL	RNA POLYMERASE BETA-SUBUNIT AAA21416	-0,372112454
VSARLAGIPYIAIAN	POSSIBLE GLYCOSYL TRANSFERASE CAB05415	-0,373672075
GLTHKRRLSALGPGG	RNA POLYMERASE BETA-SUBUNIT AAA21416	-0,377898616
GRHGNKGVIGKILPV	RNA POLYMERASE BETA-SUBUNIT AAA21416	-0,384967081
GVVNISGIEIKDLAG	YP_177963; PPE FAMILY PROTEIN	-0,38607622
YVSRLLAKLGMERRT	TWO COMPONENT TRANSCRIPTIONAL REGULATORY PROTEIN DEVR NP_217649	-0,386874925
IPQLPLGLGLSGGTP	YP_177963; PPE FAMILY PROTEIN	-0,39296585
VHEMVSIPEDGGVVL	CAA17111.1; PROBABLE ISOCITRATE DEHYDROGENASE	-0,395192255
LPTVVIDRIPVELHA	YP_177963; PPE FAMILY PROTEIN	-0,40335159
VVGTRQTLQGASVTV	CAA98373.1; MAJOR SECRETED IMMUNOGENIC PROTEIN	-0,424812268
SVQIQVAIPLTSATL	YP_177963; PPE FAMILY PROTEIN	-0,44194934
DTTILPPIHLGLTGQ	YP_177963; PPE FAMILY PROTEIN	-0,442488384
TLHSGVLNLGSGLSG	YP_177963; PPE FAMILY PROTEIN	-0,453699372
FEAALAATVDPAAVA	YP_177963; PPE FAMILY PROTEIN	-0,455817492
AAAAALPAFSPPAQA	YP_177963; PPE FAMILY PROTEIN	-0,458665758
VAAVLPACLDSIARQ	POSSIBLE GLYCOSYL TRANSFERASE CAB05419	-0,46019168
LKVVEQASLRAGPPT	HYPOTHETICAL PROTEIN RV2623 NP_217139	-0,462208415
HLIGPPPATVIRLSD	CONSERVED HYPOTHETICAL PROTEIN CAB08634	-0,464400191
VELGYDRTAHQDVPS	PPE FAMILY PROTEIN CAE55504	-0,495205498
MAKLSTDELLDAFKE	CAB07109.1; PROBABLE 50S RIBOSOMAL PROTEIN L7/L12 RPLL	-0,508461187
GLLGIASTLNPQVGS	PPE FAMILY PROTEIN CAE55334	-0,509050419
VYVADRGNDRVVKLT	TRANSMEMBRANE SERINE/THREONINE-PROTEIN KINASE D PKND NP_215446	-0,521859632
DVQVTMPVPEPMVKM	CONSERVED HYPOTHETICAL PROTEIN TB18.5CAD93033	-0,541909454
LLGLLSEGLTNKQIA	TWO COMPONENT TRANSCRIPTIONAL REGULATORY PROTEIN DEVR NP_217649	-0,547093757
TRLASLVAANLLGQN	PPE FAMILY PROTEIN CAE55504	-0,58142395
DTNIIKLPNISASVP	PROBABLE ISOCITRATE DEHYDROGENASE CAA16247	-0,612020171
SIIEVDLLDLDAPNG	MYCOBACTERIUM BOVIS ACYL-COA SYNTHASE GENE; U75685	-0,642602812
VVFPIVQGELSKQTG	CAA98382.1; IMMUNOGENIC PROTEIN MPT64	-0,656512179
KRRQPVTVLLKALGW	RNA POLYMERASE BETA-SUBUNIT AAA21416	-0,701395061
LNALADAVKVTLGPK	60 kDa chaperonin 2 P0A521	-0,737675477

Peptides	Protein	Average Index
APLDGVGALLRYAAT	CONSERVED HYPOTHETICAL PROTEIN CAB08634	-0,772298387
ADPLTSGLLGIASTL	PPE FAMILY PROTEIN CAE55334	-0,831953838
LRVLDEIAPDLVVGD	POSSIBLE GLYCOSYL TRANSFERASE CAB05415	-0,871855689
STVKDLLPLLEKVIG	60 kDa chaperonin 2 P0A521	-1,006236593
MKLNRFGAAVGVLAA	PERIPLASMIC PHOSPHATE-BINDING LIPOPROTEIN PSTS3 YP_177768	0,111501131
NAAFSKLPASTIDEL	CAA98373.1; MAJOR SECRETED IMMUNOGENIC PROTEIN	-0,424477996
