# Supplementary material for: Pattern Recognition in Pulmonary Tuberculosis Defined by High Content Peptide Microarray Chip Analysis Representing 61 Proteins from M. tuberculosis
Source: PLoS One. 2008 Dec 9;3(12):e3840. doi: 10.1371/journal.pone.0003840 (PMC2588537; doi:10.1371/journal.pone.0003840)
Supplement: Table S7 — Blast search of the top 12 peptide exclusively recognized in healthy, PPD-, Quantiferon-negative individuals. Blast search of the top 12 peptides exclusively recognized in TB-negative individuals. The peptide amino acid sequence and peptide number as well as the protein ID and Rv numbers are provided. The search allowed for at most two amino acids variation from the query peptide except in some few cases highlighted with a star (greater variation as compared with the query peptide). If greater variation with the query peptides was allowed concerning the peptide length, more matches with peptides derived from non-mycobacterial species were obtained. Amino acid differences are marked in red. A detailed blast search covering all possible permutations of these peptides, followed by targeted amino acid substitutions and subsequent serum recognition analysis will aid to define immunogenicity. (0.02 MB PDF) [file pone.0003840.s007.pdf]

Supplementary Table 7: Blast search of the top 12 peptide exclusively recognized in healthy, PPD-, Quantiferon-negative individuals.

YNGWDINTPAFEWYY-030 (CAB10044, Rv1886c)

YNGWDINTPAFEWYY	M.tuberculosis
YNGWDINTPAFEWYY	M.marinum
YNGWDINTPAFEWYY	M.ulcerans
YNGWDINTPAFEWYY	M.bovis
YNGWDINTPAFEWYY	M.avium
YNGWDINTPAFEWYY	M.intracellulare
YNGWDINTPAFEWFY	M.smegmatis
YNGWDINTPAFEWFY	M.vanbaalenii

SPACGKAGCQTYKWE-042 (CAB10044, Rv1886c)

SPACGKAGCQTYKWE	M. tuberculosis
SPACGKAGCQTYKWE	M. bovis
SPACGKAGCQTYKWE	M. leprae
SPACGKAGCSTYKWE	M. gilvum
-PACGKAGCTTYKWE	M. marinum
-PACGKAGCTTYKWE	M. avium
-PACGKAGCSTYKWE	M. vanbaalenii
-PACGKAGCSTYKWE	M. flavescens

PLIFALYCLPLNWLR-063 (CAB05418, Rv2958c)

PLIFALYCLPLNWLR	M. tuberculosis
PLIFALYCLPLNWLR	M. bovis
PLIFAIYCLPLNWVR	M.leprae
LYCLPLHWM*	Infectious hypodermal & hematopoieticnecrosis virus

TVFKLTADGVLTAPQ-056 (M95808)

TVFKLTADGVLTAPQ	M. tuberculosis
TVFKLTADGVLTAPQ	M. bovis
TVFNLTADGVLTAPE	M. leprae
KLTADGVSLTAKQ*	Caenorhabditis elegans
TVFKLSLDGVL*	Parabacteroides merdae
KLTSDGVLT*	Plasmodium vivax
ADGVLTAP*	Methylobacterium sp.

SSGAALLGSPGQGAY-063 (M95808)

SSGAALLGSPGQGAY	M. tuberculosis
SSGAALLGSPGQGAY	M. leprae
SSGAALLGSPGQGAY	M. bovis
SSGAALMGSPGQGAY	M. marinum
SSGAALMGSPGQGAY	M. ulcerans
SSSSALLGSPGQGAY	Streptomyces fradiae
SSAAGLLGSPGQGAY	Streptomyces sp
SSAAALVGSPGQGAY	M.smegmatis
SSAAALVGSPGQGAY	M. avium

VHPLLGSHVRLTEEP-029 (M95808)

```
VHPLLGSHVRLTEEP    M. tuberculosis
VHPLLGSHVRLTEEP    M. bovis
VHPLLGSHVRLPEEP    M. avium
VHPLLGAHVRLAEEP    M. abscessus
VHPLLGSHVRLLEEP    M. leprae
VHPLLGSHVVLPQEP    M. smegmatis
 HPLLGSVVRLAE*     Streptomyces hygroscopicus
```

AVDALTEYGLLRGSW -018 (CAA16235, Rv3922c)

```
AVDALTEYGLLRGSW    M. tuberculosis
AVDALTEYGLIRGTW    M. smegmatis
AVDALSEYGLIRGSW    M. ulcerans
AVDALDEYGLIRGSW    M. avium
       YGLLRGSW*   Novosphingobium aromaticivorans
       YGLLRGSW*   Rhodopseudomonas palustris
 VDGLTESGLLRG*     Saccharopolyspora erythraea NRRL 2338
```

AAAAMAAAAAPYAGW-021 (YP_177963, Rv3347c)

```
AAAAMAAAAAPYAGW    M. tuberculosis
AAAAMAAAAAPYAGW    M. marinum
AAAAMAAAAAPYAGW    M.bovis
AAAAMAAAAAPYAGW    M.ulcerans
```

FRLPLLFVNALGDNW-578 (YP_177963, Rv3347c)

```
FRLPLLFVNALGDNW    M.tuberculosis
FRLPLLFVNALGDNW    M. bovis
FR-PLLFVNAL*       Marinobacter algicola
FKLPLLFVIAL*       Lactococcus lactis
FRVPLLFVAAL*       Aspergillus clavatus
FR-PLLFINAL*       Marinobacter aquaeolei
```

NIGNANVGFGNIGHG-674 (YP_177963, Rv3347c)

```
NIGNANVGFGNIGHG    M.tuberculosis
NIGNANVGFGNIGHG    M.bovis
```

NRTLLSLMDAWAGPV-016 (CAE55489, Rv2430c)

```
NRTLLSLMDAWAGPV    M.tuberculosis
NRTLLSLMDAWAGPV    M.bovis
  TLLDLMDAWA*      Akkermansia muciniphila
```

AMTRAASPYVGWLNT-022 (CAE55489, Rv2430c)

```
AMTRAASPYVGWLNT    M.tuberculosis
AMTRAASPYVGWLNT    M. bovis
```
